# Experimental and Theoretical Study of the OH-Initiated Degradation of Piperidine under Simulated Atmospheric Conditions

**DOI:** 10.1021/acs.jpca.3c08415

**Published:** 2024-03-29

**Authors:** Wen Tan, Liang Zhu, Tomas Mikoviny, Claus J. Nielsen, Armin Wisthaler, Barbara D’Anna, Simen Antonsen, Yngve Stenstrøm, Naomi J. Farren, Jacqueline F. Hamilton, Graham A. Boustead, Trevor Ingham, Dwayne E. Heard

**Affiliations:** † Section for Environmental Sciences, Department of Chemistry, University of Oslo, P.O.Box. 1033 Blindern, NO-0315 Oslo, Norway; ‡ Aix-Marseille University, CNRS, LCE, UMR 7376, Marseille 13331, France; § Faculty of Chemistry, Biotechnology and Food Science, Norwegian University of Life Sciences, P.O. Box 5003, N-1432 Ås, Norway; ∥ Wolfson Atmospheric Chemistry Laboratories, Department of Chemistry, University of York, YO10 5DD York, U.K.; ⊥ School of Chemistry, University of Leeds, LS2 9JT Leeds, U.K.

## Abstract

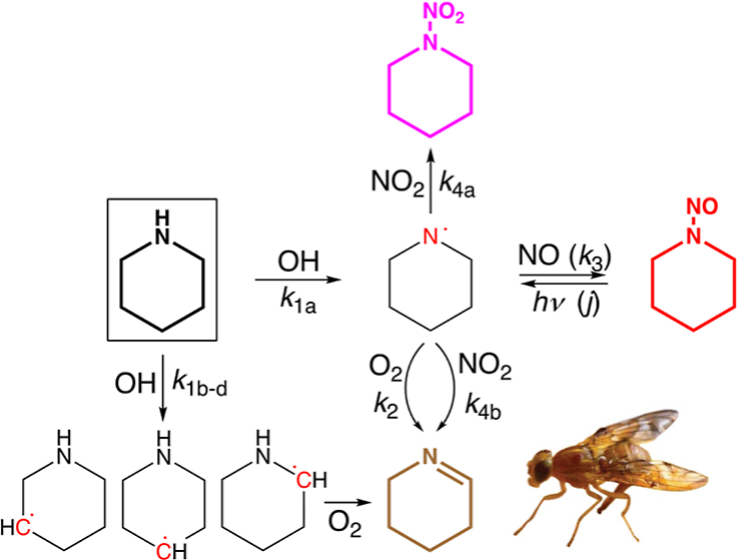

The OH-initiated photo-oxidation of piperidine and the photolysis of 1-nitrosopiperidine were investigated in a large atmospheric simulation chamber and in theoretical calculations based on CCSD(T*)-F12a/aug-cc-pVTZ//M062X/aug-cc-pVTZ quantum chemistry results and master equation modeling of the pivotal reaction steps. The rate coefficient for the reaction of piperidine with OH radicals was determined by the relative rate method to be *k*_OH-piperidine_ = (1.19 ± 0.27) × 10^–10^ cm^3^ molecule^–1^ s^–1^ at 304 ± 2 K and 1014 ± 2 hPa. Product studies show the piperidine + OH reaction to proceed via H-abstraction from both CH_2_ and NH groups, resulting in the formation of the corresponding imine (2,3,4,5-tetrahydropyridine) as the major product and in the nitramine (1-nitropiperidine) and nitrosamine (1-nitrosopiperidine) as minor products. Analysis of 1-nitrosopiperidine photolysis experiments under natural sunlight conditions gave the relative rates *j*_rel_ = *j*_1-nitrosoperidine_/*j*_NO_2__ = 0.342 ± 0.007, *k*_3_/*k*_4a_ = 0.53 ± 0.05 and *k*_2_/*k*_4a_ = (7.66 ± 0.18) × 10^–8^ that were subsequently employed in modeling the piperidine photo-oxidation experiments, from which the initial branchings between H-abstraction from the NH and CH_2_ groups, *k*_N–H_/*k*_tot_ = 0.38 ± 0.08 and *k*_C^2^–H_/*k*_tot_ = 0.49 ± 0.19, were derived. All photo-oxidation experiments were accompanied by particle formation that was initiated by the acid–base reaction of piperidine with nitric acid. Primary photo-oxidation products including both 1-nitrosopiperidine and 1-nitropiperidine were detected in the particles formed. Quantum chemistry calculations on the OH initiated atmospheric photo-oxidation of piperidine suggest the branching in the initial H-abstraction routes to be ∼35% N^1^, ∼50% C^2^, ∼13% C^3^, and ∼2% C^4^. The theoretical study produced an atmospheric photo-oxidation mechanism, according to which H-abstraction from the C^2^ position predominantly leads to 2,3,4,5-tetrahydropyridine and H-abstraction from the C^3^ position results in ring opening followed by a complex autoxidation, of which the first few steps are mapped in detail. H-abstraction from the C^4^ position is shown to result mainly in the formation of piperidin-4-one and 2,3,4,5-tetrahydropyridin-4-ol, whereas H-abstraction from N^1^ under atmospheric conditions primarily leads to 2,3,4,5-tetrahydropyridine and in minor amounts of 1-nitrosopiperidine and 1-nitropiperidine. The calculated rate coefficient for the piperidine + OH reaction agrees with the experimental value within 35%, and aligning the theoretical numbers to the experimental value results in *k*(T) = 2.46 × 10^–12^ × exp(486 K/T) cm^3^ molecule^–1^ s^–1^ (200–400 K).

## 1 Introduction

Piperidine (PIP) is on the OECD list of high production volume chemicals;^1^ the compound is not considered persistent, bioaccumulative, and inherently toxic to aquatic organisms.^2^ It is, however, classified as hazardous with a workplace exposure limit of 1 ppm time weighted average. Piperidine is used as an intermediate for pharmaceuticals and for plant protection agents, as a vulcanization accelerator in rubber manufacture, and as an oil or fuel additive.^3^ The compound has been reported in waste gas emissions from agricultural operations and food industries^4^ and detected in outdoor particle and air samples collected in the Zonguldak province (Northern Turkey at the Black sea coast), where coal mining and associated activities are economic drivers.^5,6^ Piperidine has also been detected in many samples of source water to major cities in China with concentrations of up to 2.35 μg L^–1^.^7^

The corresponding nitrosamine, 1-nitrosopiperidine (PIP-NO), was reported in the atmospheric particulate matter (PM_2.5_) in central London^8^ and later in PM_2.5_ and PM_10_ samples^9,10^ collected at Seoul, Korea, and recently in PM_2.5_ samples collected at Urumqi, China.^11^ A comprehensive examination of nitrosamines in a Korean water system, including sewage treatment plants, river water, and seawater reported PIP-NO concentrations of ∼100 ng L^–1^ in the sewage treatment plants influent and ∼5 ng L^–1^ in the effluent, whereas the concentrations in the river water and seawater was reported to be <1 and ∼2 ng L^–1^, respectively.^12^ More recently, PIP-NO was reported with an average of 22 ng L^–1^ in 4 of 5 drinking water samples collected from 13 cities in the state of São Paulo, Brazil,^13^ and in 47 of 117 samples of treated water throughout China (average 2.9 ng L^–1^).^14^ To the best of our knowledge, neither 1-nitropiperidine (PIP-NO_2_) nor the piperidine imine, 2,3,4,5-tetrahydropyridine (PIP-IM), have ever been reported in environmental samples. Intriguingly, PIP-IM is an attractor to the Mexican fruit fly.^15^

The Henry’s law solubility of PIP is small, *H*^cp^ = 2.8 mol m^–3^ Pa^–1^.^16^ Consequently, on a global scale, the major atmospheric sinks will therefore be gas phase reaction with OH radicals during daytime and NO_3_ radicals during night-time. A preliminary project report from analysis of the PIP + OH kinetics suggested the reaction to be fast, ∼7 × 10^–11^ cm^3^ molecule^–1^ s^–1^ at 298 K; the report also included preliminary results from 1-nitrosopiperidine (PIP-NO) photolysis experiments.^17^ A subsequent quality control of the project data showed nonlinearity effects in the PTR-ToF-MS microchannel plate (MCP) detector warranting reanalysis of the data.^18^

PIP-NO and PIP-NO_2_ are both carcinogenic^19^ and produced in the gas phase by competing aminyl radical reactions with O_2_, NO, and NO_2_.^20^ Although the O_2_ reaction with aminyl radicals in general is expected to be around 6 orders of magnitude slower than the corresponding NO and NO_2_ reactions,^20^ it will still dominate under most atmospheric conditions, and PIP-NO and PIP-NO_2_ are thus only expected as minor products in the natural atmospheric photo-oxidation of PIP.

Piperidine was studied as part of the Norwegian “CO_2_ and Amines Screening Study for Environmental Risks”^21^ because of its structural relationship to two CCS relevant compounds: piperazine and morpholine. In the present communication, we first report results from quantum chemistry-based calculations of the OH reaction kinetics and evaluations of the major routes in the OH initiated photo-oxidations of PIP under atmospheric conditions. We then present results from a series of PIP + OH reaction kinetics studies and from PIP-NO photolysis experiments carried out in the large volume EUPHORE atmospheric simulation chamber. We finally present results from PIP photo-oxidation experiments at EUPHORE and from analyses of the particles formed in these experiments.

## 2 Methods

### 2.1 Experimental Methods and Chemicals

A series of experiments were carried out in chamber B of the EUPHORE facility at CEAM in Valencia, Spain. The facility and its standard analytical methods have been reported in detail^22^—special online instrumentation employed in the present study includes a PTR-TOF 8000 instrument (Ionicon Analytik GmbH, Innsbruck, Austria),^23^ a prototype CHARON inlet^24,25^ interfaced to a second PTR-TOF 8000, a compact time-of-flight Aerosol Mass Spectrometer (C-ToF-AMS, Aerodyne Research Inc., Billerica, MA, U.S.A.),^26,27^ and a FAGE (Fluorescence Assay by Gas Expansion) apparatus.^28−30^ Additional experimental information specific to the present work including chemicals used and the synthesis of PIP-NO^31^ and PIP-NO_2_^32^ is given in the Supporting Information.

### 2.2 Computational Methods

Optimized geometries of stationary points on the potential energy surfaces of the atmospheric degradation of PIP were obtained in density functional theory (DFT) calculations using the global-hybrid *meta*-GGA functional M06-2X^33^ in combination with the aug-cc-pVTZ^34,35^ basis set. Pre- and postreaction complexes were located by following the intrinsic reaction coordinate (IRC)^36−38^ from the saddle points. Electronic energies of selected stationary points were improved by explicitly correlated coupled cluster calculations with scaled triples contributions, denoted CCSD(T*)-F12a.^39,40^ Additional calculations on the initial step in the PIP + OH reaction were carried out employing ωB97XD^41^ (a range-separated hybrid density functional with atom–atom dispersion correction), BMK^42^ (a hybrid *meta*-GGA functional developed for kinetics), and the second-order Møller–Plesset wave function method, MP2.^43^

Nontrivial conformational analyses, including initial DFT calculations, were carried out employing a Mac-version of SPARTAN’20/Q-Chem.^44,45^ First, the conformational space was mapped in molecular mechanics calculations (MMFF94),^46^ and the structures of the (up to 500) lowest energy conformers were optimized in semiempirical AM1 calculations.^47^ Second, based on energy and focal interatomic distances relevant to internal H-transfer reactions, up to 100 conformers were selected and their structures optimized in B3LYP/3-21G* calculations.^48,49^ Third, the lowest energy conformers relevant to internal H-transfer reaction were refined in M062*X*/6-31+G(d,p) calculations on a mainframe computer before final selection for large basis set calculations.

Reaction enthalpies and proton affinities were calculated using the CBS-QB3 multilevel correlation single-reference method,^50,51^ based on B3LYP/6-311G(2d,d,p) structures, which was reported^51^ with a mean absolute deviation of 4.5 kJ mol^–1^ on the G2/97 test set.^52,53^ The CBS-QB3 composite method has also been tested against the BH28 data set of barrier heights^54^ and was reported with a mean unsigned error of 5.9 kJ mol^–1^.^55^ Additional calculations of selected barrier heights were carried out employing the multilevel correlation single-reference G3X-K method;^56^ the G3X-K method, parametrized using the DBH24/08 thermochemical kinetics database^57^ as training set, was reported with a mean unsigned error of 2.1 kJ mol^–1^ for the barrier heights.^56^

Dipole moments and isotropic polarizabilities, serving as input to prediction of ion–molecule reaction rate coefficients,^58^ were obtained in M062X/aug-cc-pVTZ and B3LYP/aug-cc-pVTZ calculations; see Table S1 in the Supporting Information. The M06-2X, B3LYP, ωB97XD, BMK, MP2, CBS-QB3, and G3X-K calculations were performed in Gaussian 09^59^ and Gaussian 16;^60^ the CCSD(T*)-F12a calculations were carried out employing Molpro 2022.3 and 2023.2.^61^

Master equation calculations were carried out using the program MESMER 5.1^62^ (Master Equation Solver for Multi-Energy-well Reactions) to simulate the reactions under atmospheric conditions. The energy range spanned by grains of 100 cm^–1^ was set to 25 kT, and the required input parameters for molecules, intermediate species and products were obtained from the ab initio calculations. Association (prereaction complex formation) and unimolecular dissociation steps were treated employing ILT (Inverse Laplace Transform), whereas unimolecular isomerization and H-transfer steps were treated by standard RRKM theory. Unless noted explicitly, the MESMER calculations reported employed the Lennard-Jones parameters for cyclohexane (*e* = 297 K, *s* = 6.18 Å),^63^ an exponential down energy transfer parameter ⟨ΔE⟩_down_ = 250 cm^–1^, and a one-dimensional asymmetric Eckart potential^64^ for describing tunneling in H-transfer reactions.

## 3 Results

### 3.1 Computational Results

**Figure 1:**
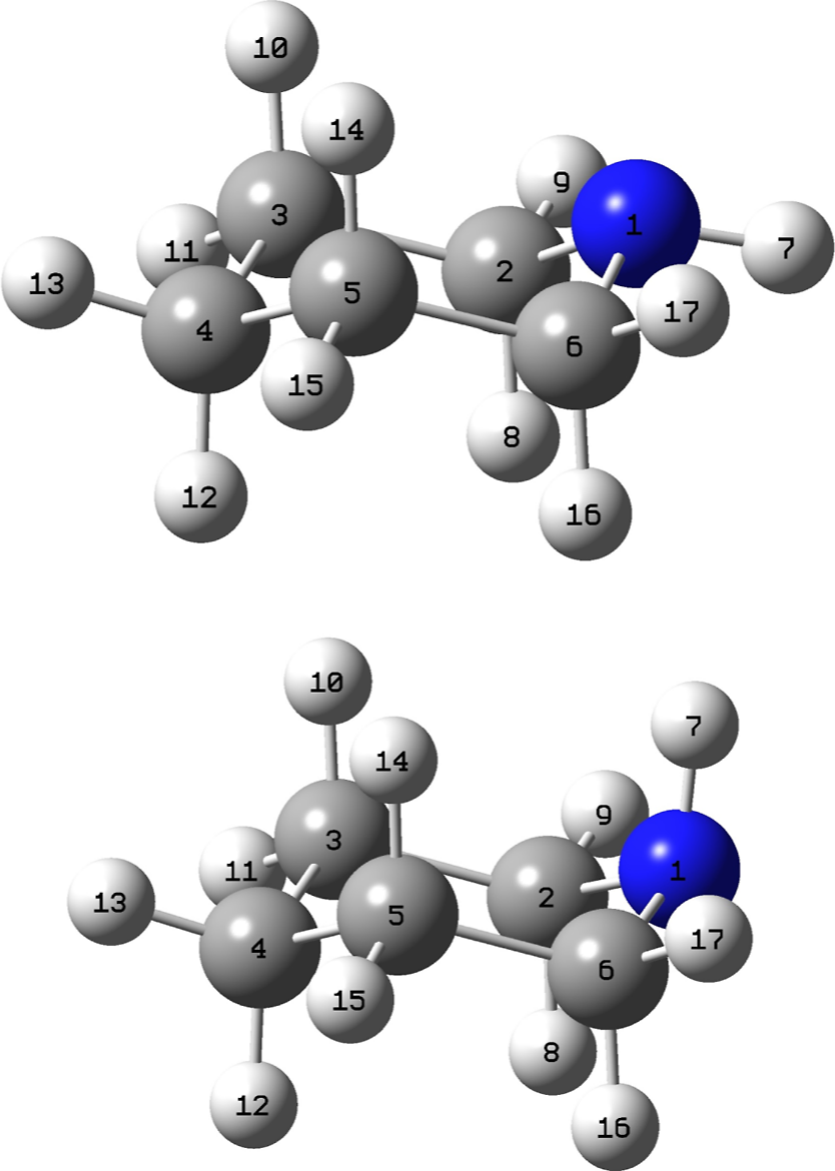
Numbering of the atoms in piperidine. Top: equatorial conformer. Bottom: axial conformer.

Piperidine (PIP) exists in two chair conformations (*C*_s_ symmetry) in which the >NH group is either equatorial (eq) or axial (ax); each conformer has four pairs of equivalent and three nonequivalent hydrogen atoms, as shown in Figure 1. The electronic structure results from M06-2X/aug-cc-pVTZ calculations agree with the equilibrium structures of the two chair conformers derived from microwave spectroscopic data in combination with ab initio results.^65^ The average absolute differences are 0.07 pm for distances, 0.13° for bond angles, and 0.53° for the dihedral angles. Also, the dipole moments compare well with the experiment (calculated values in parentheses): μ_a_ = 0.178 ± 0.007 (0.174) D and μ_c_ = 0.800 ± 0.020 (0.867) D for the equatorial form, and μ_a_ = 1.069 ± 0.015 (1.098) and μ_c_ = 0.521 ± 0.007 (0.550) D for the axial form.^66^

The energy difference between the two chair conformers is relatively small; results from CCSD(T*)-F12a/aug-cc-pVTZ//M06-2X/aug-cc-pVTZ calculations (hereafter abbreviated as CC//M062X) place Δ*H*_ax–eq_ = 2.72 and Δ*G*_ax–eq_ = 2.43 kJ mol^–1^ at 298 K, which compares to the experimental results Δ*H* = 2.22 ± 0.13 kJ mol^–1^ from van’t Hoff analyses of intensity variations in gas phase infrared absorption bands over the 50–210 °C range,^67^ and Δ*G* = 3.07 ± 0.30 kJ mol^–1^ from relative intensity measurements of rotational transitions by microwave spectroscopy at 293 and 239 K.^66^ The barrier to eq–ax inversion of the >NH group is calculated to be Δ*E*_*v*=0_^⧧^ = 19.65 kJ mol^–1^ (Δ*G*^⧧^ = 19.54 kJ mol^–1^), which compares to 25.5 ± 0.8 kJ mol^–1^ from a ^13^C NMR study of piperidine dissolved in a 1:1 blend of CHFCl_2_ and CHF_2_Cl.^68^ The conformational pathways in piperidine are excellently treated by Stortz^69^ and will not be reiterated here; in addition to the two chair forms, there are six skew, pairwise pseudoenantiomeric conformations (denoted ^3^S_1_, ^5^S_1_, and ^2^S_N_) having ∼25 kJ mol^–1^ higher energies linked by boat forms (^3,N^B, B_1,4_, and B_3,N_); the barriers between the chair forms and the skew forms (^N^H_1_, ^3^H_4_/^3^E, E_3_, and E_N_) are ∼50 kJ mol^–1^. The present results from CC//M062X calculations of stationary points on the piperidine conformational space are found in Table S2 (energies, Cartesian coordinates, vibrational frequencies, rotational constants, and T_1_^70^ and D_1_^71,72^ diagnostics values); the results obtained in the present high-level calculations do not differ markedly from the previous results obtained using relatively modest B3LYP calculations.^69^

The conformational distribution in piperidine is calculated to be ∼82% eq and 18% ax at 200 K, ∼73% eq and 27% ax et 298 K, and ∼67% ax and 33% eq at 400 K; the skew conformations are not relevant in relation to the initial reaction with OH radicals at temperatures below 700 K. An RRKM calculation places the rate of conversion between the two chair isomers >10^8^ s^–1^ under atmospheric conditions, which is orders of magnitude faster than any bimolecular reaction with atmospheric oxidants. That is, the piperidine eq–ax equilibrium is maintained at all times during the atmospheric photo-oxidation.

#### 3.1.1 Kinetics and Branching in the Piperidine + OH Reaction

The PIP + OH reaction proceeds via H-abstraction
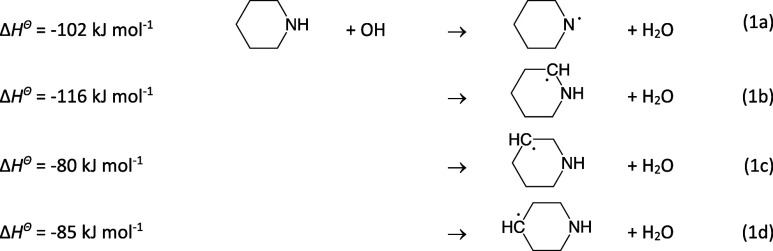
1a

All reaction enthalpies given are derived from CBS-QB3 calculations (298 K) referring to the lowest energy conformation of the species in question; all relative energies and barrier heights presented originate from CC//M062X calculations except when noted. In the following, the 1-, 2-, 3-, and 4-piperidinyl radicals will be acronymized PIPṄ, PIPĊ^2^, PIPĊ^3^, and PIPĊ^4^, respectively.

##### 3.1.1.1 Potential Energy Surfaces of the OH Radical Reaction with Piperidine

**Figure 2:**
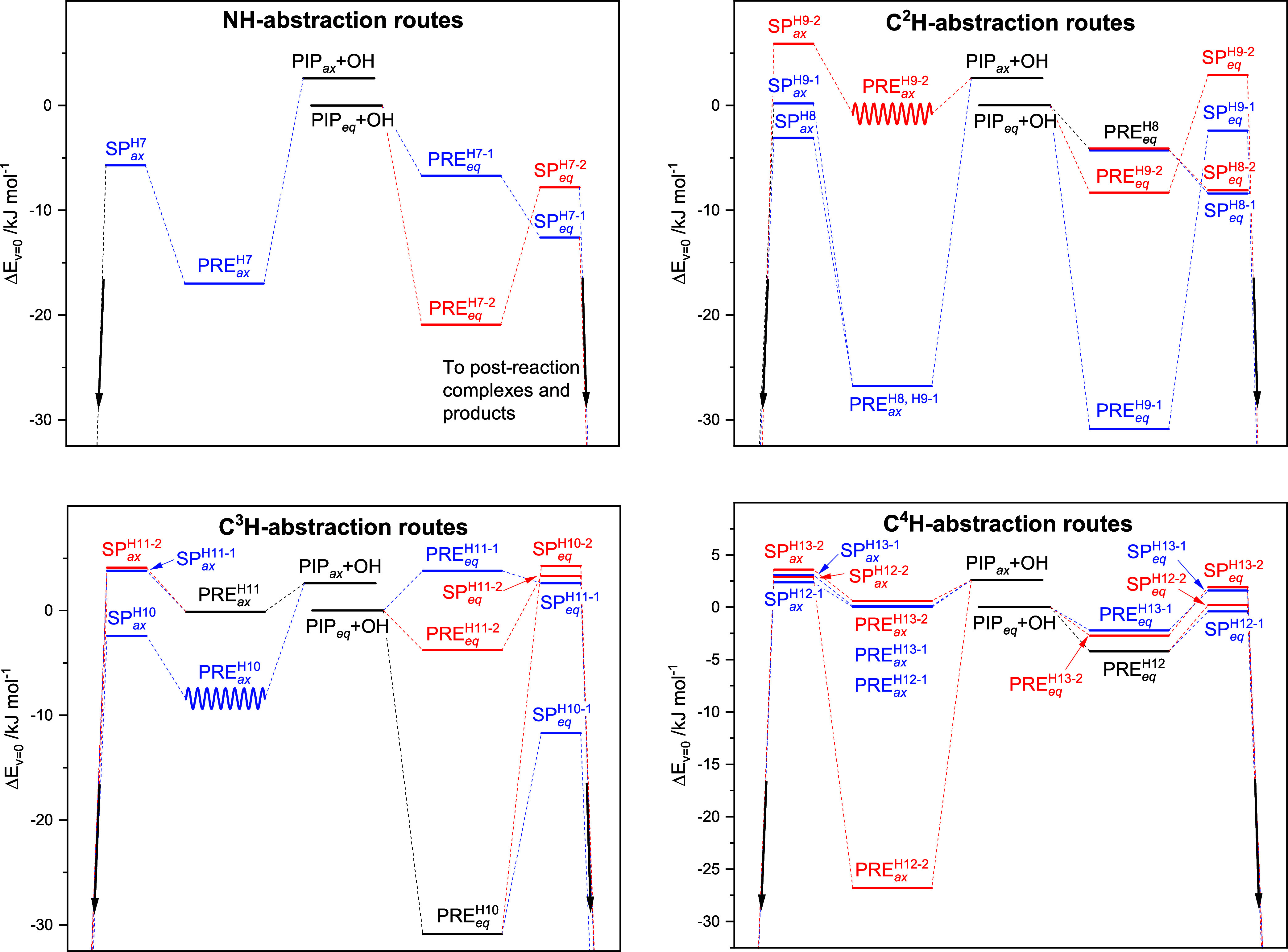
Relative energies (Δ*E*_*v*=0_ in kJ mol^–1^) of stationary points on the potential energy surface of the OH radical reaction with piperidine. Results from CCSD(T*)-F12a/aug-cc-pVTZ//M06-2X/aug-cc-pVTZ calculations. See Figure 1 for definition of atom numbering. The sinusoidal curves signify that the stationary point has not been structure optimized, see text.

The stationary points on the entrance side of the potential energy surface (PES) of the PIP + OH reaction are interrelated in Figure 2; the labeling tracks the atom number of the hydrogen being abstracted (see Figure 1) with sub labels increasing with the energy of the saddle point. On the exit side, the reaction proceeds via postreaction H-bonded H_2_O complexes (POST). The underlying quantum chemistry results are documented in Tables S3 (equatorial conformation) and S4 (axial conformation), in Figure S1, demonstrating the vastly different saddle point energies as a function of the OH radical rotation around the XH···OH axes, and in Figure S2, depicting the structures of the prereaction complexes/adducts (PRE) and the saddle points (SP) to H-abstraction.

The M062X-description of the PES gives a picture of gentle sloped saddle points with imaginary frequencies below 1000 cm^–1^ (average ∼600 cm^–1^). The majority of saddle points to H-abstraction are located with energies below the entrance energy of reactants, and 6 of the 25 first order saddle points located are linked to strongly H-bonded prereaction complexes involving the nitrogen lone pair. The other saddle points are linked to the reactants via van der Waals-type prereaction adducts resulting from electrostatic interactions, Debye forces and London dispersion interactions, of which the latter is a recognized problem in DFT theory. We note that adding Grimme’s empirical dispersion^73^ does not result in any significant improvements. The saddle points to the H-abstraction reactions can be described as “early” or “loose” with average N–H/C-H elongations relative to their respective reactants of only 5.0 pm correlating well with the imaginary frequencies of the saddle points (Table S5); the average XH···OH distances in the saddle points are consistently long with an average of 154.3 pm. The two first order saddle points to abstraction of H^8^/H^16^ in the equatorial form (see Figures 1, S1, and S2) stand out with submerged barriers of around −8 kJ mol^–1^, C–H elongations of only ∼2.5 pm, CH···OH distances of 172–174 pm, imaginary frequencies of ∼260 cm^–1^, and a barrier of only ∼2 kJ mol^–1^ to rotation of the OH radical around the CH···OH axis. IRC calculations locate a van der Waals-type prereaction adduct with slightly lower electronic energy (∼1 kJ mol^–1^ of which 80% can be ascribed to basis set superposition error at the M062X/aug-cc-pVTZ level), in which the OH radical soars on top of the axial H atoms (H^8^, H^12^, and H^16^; see Figure 1) with a rotational barrier of only ∼1.5 kJ mol^–1^. The single point CC//M062X calculations swap the relative electronic energy differences in PRE_eq_^H8–1^, PRE_eq_^H8–2^, SP_eq_^H8–1^, and SP_eq_^H8–2^ from −1 to +1 kJ mol^–1^.

The saddle point to H-abstraction from the NH group in the equatorial form, SP_eq_^H7–1^, appears with a submerged barrier of −12.6 kJ mol^–1^. In this case, IRC calculations locate a prereaction adduct with 1.6 kJ mol^–1^ lower electronic energy at the M062X/aug-cc-pVTZ level, but the single point CC/M062X electronic energy differences between PRE_eq_^H7–1^ and SP_eq_^H7–1^ changes from −1.6 to +2.1 kJ mol^–1^. Further, the N–H bond lengths are almost unchanged in SP_eq_^H7–1^ and SP_eq_^H7–2^, whereas the NH···OH distances are remarkably long, 169.4 and 177.5 pm, respectively. The NH bond is even shortened in the saddle point structure SP_eq_^7^, in which the NH···OH distance is extraordinary long, 191.7 pm.

Other worrisome issues include the prereaction adducts to the saddle points SP_ox_^H9–2^ and SP_ox_^H10^ in the axial conformer. IRC calculations place nearly identical prereaction adducts, PRE_ox_^H9–2^ and PRE_ox_^H10^, with the OH radical floating over H^10^, H^9^, and H^7^ of the amine group, and any attempts on structural refinements of PRE_ox_^H9–2^ and PRE_ox_^H10^, resulting as the end points of the IRCs, lead to direct abstraction of H^7^ and formation of a postreaction complex between H_2_O and PIPṄ. These two prereaction adducts are outlined as sinusoidal curves in Figure 2.

The above-mentioned oddities in the M06-2X description of the piperidine + OH reaction prompted an additional examination of selected PES regions employing two other commonly used functionals—ωB97XD,^41^ and BMK^42^—as well as by the MP2 wave function method.^43^ The results from these comparative studies are included in Table S5.

The ωB97XD calculations locate the saddle points to H-abstraction with systematically lower energies relative to that of the reactants than found by M06-2X (average 7.9 kJ mol^–1^lower), and the saddle points show even gentler inclines than in the M06-2X description (average imaginary frequency ∼280 cm^–1^). Single point CC//ωB97XD calculations place the relative saddle point energies systematically higher compared to the CC//M062X results by an average of 2.6 kJ mol^–1^. The C–H bond elongations are slightly lesser and the CH···OH distances slightly longer in the saddle points than those obtained in the M06-2X calculations. Despite meticulous efforts, it was not possible to locate any saddle points to H^8^/H^16^ abstraction in the equatorial conformation. Apparently, this H-abstraction route proceeds without any electronic barrier in the ωB97XD/aug-cc-pVTZ description of the reaction. Also SP_ox_^H13–2^ evaded finding. It is worth noting that the N–H bond lengths are shortened in all saddle points to H-abstraction from the amino group and the corresponding NH···OH distances are extremely long, ∼190 pm.

The BMK results also show gentle sloped saddle points (average imaginary frequency ∼400 cm^–1^), but the average N–H/C–H bond elongations in the saddle points are slightly longer (average 5.9 pm), and the CH···OH distances slightly shorter (average 146.7 pm) than found in the M06-2X description of the reaction. The saddle points to H^8^/H^16^ abstraction in the equatorial conformation could only be located in small basis set calculations, whereas BMK/aug-cc-pVTZ calculations portray this H-abstraction route without any electronic barrier. In addition, the saddle points SP_ox_^H9–2^ and SP_ox_^H13–2^ could not be located. The other relative saddle point energies differ from those of the M06-2X calculations by an average of −0.6 kJ mol^–1^; the single point CC//BMK calculations place the relative saddle point energies systematically higher compared to the CC//M062X results by an average of 2.2 kJ mol^–1^. The BMK calculations also render the N–H bond lengths shortened in all saddle points to H-abstraction from the amino group and the corresponding NH···OH distances as particularly long.

As expected, the MP2 calculations locate steeper sloped saddle points (average imaginary frequency ∼1270 cm^–1^) with relative energies higher by 8.6 kJ mol^–1^ on the average than forecast by the M062X calculations. The C–H bond elongations are, also expectedly, found to be longer in the saddle points than in the DFT calculations, and the corresponding CH···OH distances are significantly shorter (average 141.6 pm). The saddle point to H-abstraction from the NH group in the axial form could not be located with certainty–energy mappings from calculations of the OH radical approaching the axial N–H invariably resulted in >N–H inversion prior to H-transfer.

It is obvious from the above that the PIP + OH reaction presents a challenge to quantum chemistry. None of the DFTs employed give an impeccable description of the PES. In particular, the ωB97XD and BMK functionals are apparently not able to describe the potential for the dominant C^2^ H-abstraction route. The computationally expensive MP2 wave function method gives, with one exception, an intuitively appealing description of the reaction routes, the exception being the H-abstraction from the NH group in the axial conformation that could not be located with certainty. Although there are a few peculiarities, the M06-2X functional provides a reasonable characterization of PIP + OH reaction.

##### 3.1.1.2 Rate Coefficient and Branching in the OH Radical Reaction with Piperidine

The kinetics of reaction 1 was simulated in a master equation model based on the PES illustrated in part in Figure 2 (all vibrational modes were treated as harmonic oscillators). Spin–orbit coupling in the OH radical (139.7 cm^–1^)^74^ was included in the model by lowering the energy of the OH radical with half of the splitting and including the ^2^Π_3/2_ and ^2^Π_1/2_ spin–orbit states in the electronic partition function; it was assumed that spin–orbit coupling could be neglected in prereaction adducts and in the saddle points. The formation of prereaction complexes and dissociation of postreaction complexes were treated as reversible reactions with rate coefficients approximated by *k*_association_ from long–range transition state theory (LRTST).^75^ The different dipole moments of the equatorial and axial forms of piperidine results in slightly different values, *k*_association_ = 2.3 and 2.7 × 10^–10^ × (T/298 K)^−1/6^ cm^3^ molecule^–1^ s^–1^ for the equatorial and axial forms, respectively. Tunneling was included using a one-dimensional asymmetric Eckart potential.^64^

**Figure 3:**
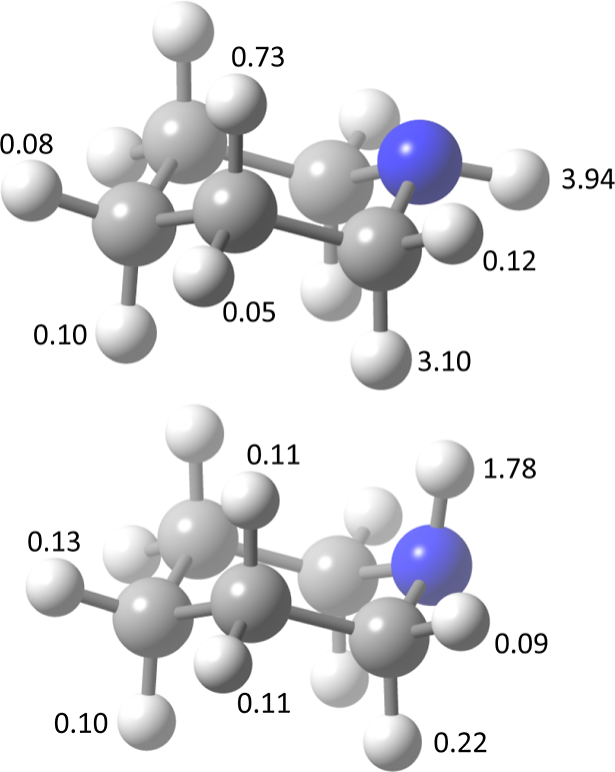
Calculated site-specific rate coefficients at 298 K (in 10^–11^ cm^3^ molecule^–1^ s^–1^) in the piperidine + OH reaction. Top: Equatorial conformer. Bottom: Axial conformer. Results from MESMER models based on CCSD(T*)/aug-cc-pVTZ//M062X/aug-cc-pVTZ calculations.

The calculations forecast remarkably different rate coefficients for the equatorial and axial conformers—*k*_PIPeq+OH_ = 1.21 × 10^–10^ and *k*_PIPax+OH_ = 0.31 × 10^–10^ cm^3^ molecule^–1^ s^–1^ at 298 K. The branching in the H-abstraction reaction is indeed different for the two conformations—32, 53, 13, and 2% from the N^1^, C^2^, C^3^, and C^4^ positions in the equatorial form and 58, 20, 14, and 7% in the axial form. The conformational Boltzmann weighted rate coefficient, *k*_PIP+OH_ = 9.77 × 10^–11^ cm^3^ molecule^–1^ s^–1^ at 298 K, shows modest pressure dependence in the 1–1000 hPa region and displays a negative temperature dependence, as shown in Figure S3. The individual rate coefficients at 1013 hPa for all routes specified in Figure 2 are summarized in Table S6, whereas the rate coefficients per H atom are presented in Figure 3.

A sensitivity analysis, based on varying the Lennard-Jones parameters, ⟨Δ*E*⟩_down_ and the LRTST values by a factor of 2 in the master equations, shows that the model results are essentially neutral to variations in the Lennard-Jones parameters and ⟨Δ*E*⟩_down_—the largest changes amount to a few percent. The sensitivity of the calculated rate coefficient and the branching to variations in the saddle point energies was examined by varying all barrier heights by ±1 kJ mol^–1^ in the master equations. The analysis shows that changing all barriers ±1 kJ mol^–1^ results in a ∓ 29% change in the calculated overall rate coefficient at 298 K. The influence of the prereaction complex/adduct energies was examined in a similar way by varying their relative energies by ±1 kJ mol^–1^. In general, the calculated rate coefficients are far less sensitive to variation in the PRE-energies than in the SP energies (barrier heights), the most noteworthy exception being PRE_eq_^H7–1^. As mentioned above, PRE_eq_^H7–1^ is located with 1.6 kJ mol^–1^ lower electronic energy than SP_eq_^H7–1^ in M062X/aug-cc-pVTZ calculations, but at +2.1 kJ mol^–1^ higher energy in the single point CC/M062X calculation. Lowering the PRE_eq_^H7–1^ energy by 2.1 kJ mol^–1^ increases the calculated rate coefficient for this route by 37%, but lowering it further by additional 1.6 kJ mol^–1^ brings the calculated rate coefficient back to its original value.

In summary, the branching between H-abstraction from N^1^, C^2^, C^3^, and C^4^ position in PIP is projected to be 35(+19–8): 50(+11–20): 13(+4–5): 2(+1–1) at 298 K, where the attributed limits are estimated from variations in all barriers by ±1 kJ mol^–1^.

#### 3.1.2 Atmospheric Photo-Oxidation

The following sections detail the quantum chemistry and master equation modeling of the atmospheric chemistry of PIP. Peroxy-radical reaction routes, such as ROȮ + ROȮ →···, ROȮ + HOȮ →··· and ROȮ + NO → RONO_2_, are not considered in the present investigation.

The master equation modeling often assumes rate coefficients for several bimolecular reactions, i.e., >ĊH + O_2_ → > CHOȮ, >CHOȮ + NȮ → >CHȮ + NO_2_ and >CHȮ + O_2_ → >C=O + HO_2_. For cyclic radicals, these rate coefficients have been approximated by the experimental values (in cm^3^ molecule^–1^ s^–1^) for the similar reactions of the cyclohexyl- (*k*_>ĊH+O_2_→>CHOȮ_ = 1.3 × 10^–11^),^76^ cyclohexyloxy- (*k*_>CHȮ+O_2_→>C=O+HO_2__ = 1.8 × 10^–14^),^77^ and cyclohexylperoxy radicals (*k*_>CHOȮ+NO→>CHȮ+NO_2__ = 6.7 × 10^–12^).^76^ The average NO*x* level in rural Europe was 2.5 ppbV in 2019,^78^ and an NO level of 1 ppbV has been taken as standard for unpretentious estimations of ROȮ + NO → RȮ + NO_2_ reaction rates under atmospheric conditions.

There is only one chair form of the PIPṄ and PIPĊ^2^ radicals, whereas the PIPĊ^3^ and PIPĊ^4^ radicals exist in both eq and ax chair conformations. The enthalpy of the ax-form is ∼1 kJ mol^–1^ lower than the eq-form in PIPĊ^3^, whereas the ax-form has ∼1 kJ mol^–1^ higher enthalpy than the eq-form in PIPĊ^4^. The barriers to ax–eq conversion in the PIPĊ^3^ and PIPĊ^4^ radicals are nearly the same as in PIP itself, that is, ∼20 kJ mol^–1^, and the ax–eq conformational equilibrium will therefore be established before the primary PIP radicals experience any bimolecular reaction. Cartesian coordinates, vibrational frequencies, rotational constants, energies, and T_1_^70^ and D_1_^71,72^ diagnostics values of stationary points on the eq–ax potential energy surfaces of the PIPĊ^3^ and PIPĊ^4^ radicals are found in Table S7.

The conformational pathways of the PIPṄ radical resemble those of tetrahydropyran with two pairs of pseudoenantiomeric skew conformations, located with 17–18 kJ mol^–1^ higher energy than the chair form.^69^ The PIPĊ^2^ radical has four skew conformation pairs having 17–21 kJ mol^–1^ higher energies than the chair conformation. The PIPĊ^3^ radical is also found with four pairs of skew conformations having energies between 13 and 18 kJ mol^–1^ above that of PIPĊ_ox_^3^, but the PIPĊ^4^ radical is only located with three pairs of skew conformations, having energies 19–21 kJ mol^–1^ above that of PIPĊ_eq_^4^. The conformational pathways of the PIPṄ, PIPĊ^2^, PIPĊ^3^ and PIPĊ^4^ radicals have not been mapped in detail; the skew conformers, however, will clearly not contribute notably to the radical reactivities under atmospheric conditions.

##### 3.1.2.1 Atmospheric Fate of the 1-Piperidinyl Radical

Under atmospheric conditions, aminyl radicals may react with O_2_, NO, NO_2_, and O_3_. The dominant reaction is normally H-abstraction from the carbon atom in α-position by O_2_ leading to the corresponding imine, whereas reactions with NO, NO_2_, and O_3_ constitute minor loss routes.

We first address the reaction between PIPṄ and O_2_
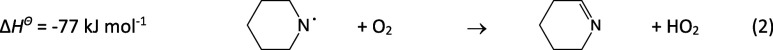
2

Tang and Nielsen^79^ investigated the CH_3_ṄH, (CH_3_)_2_Ṅ, CH_3_CH_2_ṄH and (CH_3_CH_2_)_2_Ṅ reactions with O_2_ in B3LYP, MP2 and G4 calculations and showed that they proceed via the R^1^R^2^NOȮ radical on the entrance side and a post reaction adduct between the corresponding imine (here, PIP-IM) and HO_2_ on the exit side. They also identified two additional H-abstraction routes having significantly higher barriers and therefore of less importance under atmospheric conditions.

The 1-piperidinyl peroxy-radical (PIPNOȮ) exists in both equatorial and axial forms having approximately equal energies and being connected via a barrier around 15 kJ mol^–1^ above the entrance energy of reactants. The formation of PIPNOȮ is calculated to be slightly endergonic with Δ*G*^Θ^ = 5.4 kJ mol^–1^ at 298 K, and the PIPNOȮ radical lifetime with respect to back-dissociation is estimated to be less than 1 μs. Consequently, only unimolecular reactions, such as eq–ax conversion and internal H-transfer reactions, may compete with back-dissociation under atmospheric conditions.

Alam et al. studied the CH_3_ṄH + O_2_ reaction in G3X-K calculations and reported a small barrier of ∼10 kJ mol^–1^ to the nitroperoxy radical formation, CH_3_ṄH + O_2_ → CH_3_NHOȮ.^80^ This entry barrier is, however, an artifact of applying a single-reference methodology to the generic multireference problem of a radical–radical reaction and is disclosed by the unmistakable accompanying spin-contamination, exemplified in Figure S4 exposing the PIPNOȮ_eq_ → PIPṄ + O_2_ dissociation.

In principle, there are two possible 1,4-H transfer reactions within the eq conformational subspace (transfer of either H_eq_ or H_ax_), but only one within the ax subspace (H_eq_). However, the minimum energy path to H_eq_-transfer starting from PIPNOȮ_eq_ shows that this route is a two-step process, the first being a conformational change from PIPNOȮ_eq_ to PIPNOȮ_ax_. Further, the 1,5-H transfer from C^3^ is only possible within the conformational subspace of the ax-form. Finally, the H atoms in C^4^ position are too far away in PIPNOȮ for H-transfer, and the possible 1,6-H transfer route has been disregarded here. In addition to the two chair forms of the PIPNOȮ radical, three pairs of pseudoenantiomeric skew conformers, linked by boat like saddle points, have been identified. The total population of these conformers, having from 22 to 29 kJ mol^–1^ higher energies than the chair forms, will be less than 0.02% at 298 K. The saddle point energies of reaction 2, linked to the skew forms, are slightly higher in energy than those linked to the chair forms, and the skew conformations will therefore not contribute notably to the kinetics of reaction 2.
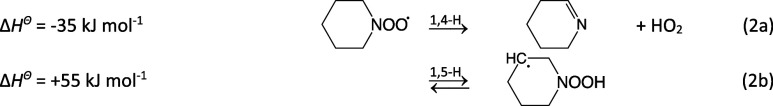
2a

**Figure 4:**
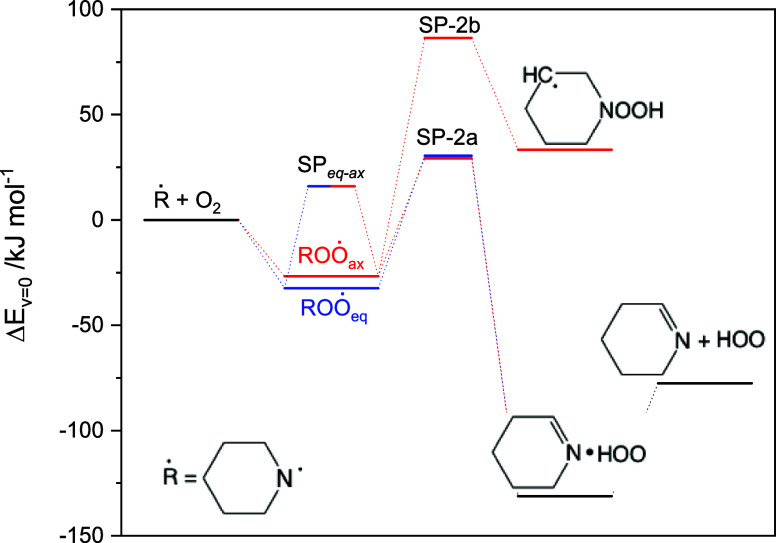
Relative energies of stationary points on the potential energy surface relevant to the 1-piperidinyl radical reaction with O_2_. Results from CCSD(T*)-F12a/aug-cc-pVTZ//M062X/aug-cc-pVTZ calculations. The underlying quantum chemistry data are summarized in Table S8.

Figure 4 illustrates the links between stationary points on the PES relevant to reaction 2. Not only is the barrier to internal H-transfer from C^3^ ∼ 90 kJ mol^–1^ above the entrance energy of reactants, the reversible H-transfer is also quite endothermic and this route will consequently be of little importance under atmospheric conditions; the barriers to imine formation are calculated to be lower, ∼30 kJ mol^–1^ above the entrance energy of reactants, and of similar magnitude as the corresponding barriers found for the 1-piperazinyl + O_2_ reaction.^81^ Unexpectedly, however, the saddle points energies obtained in CC//M062X calculations are ∼30 kJ mol^–1^ lower than the values obtained in M06-2X/aug-cc-pVTZ calculations, and the T_1_/D_1_ diagnostic values for the coupled cluster calculations are arguably large −0.026/0.151 and 0.033/0.192 for SP_ox_^2a^ and SP_eq_^2a^, respectively, indicating inadequacy of the adapted single-reference electron correlation methodology. The multireference nature of reaction 2a is also reflected in the concurrent spin-contamination in the DFT results along the IRC from reactant (PIPNOȮ) to intermediate product (PIP-IM*HOȮ). In the ax form, the spin-contamination maximizes before the saddle point to H-transfer (Figure S5)—the signature of a late transition state. According to the M06-2X description, the corresponding PIPNOȮ_eq_ reaction proceeds in two steps: (1) the formation of an intermediate PIPṄ*O_2_ complex via a barrier of around 60 kJ mol^–1^, as shown in Figure S4, and (2) H-abstraction/H-transfer to the PIP-IM*HOȮ, as shown in Figure S5. The spin-contamination remains high from there on (*S*^2^ = 1.16 before annihilation in the saddle point) and even well into the product side of the reaction; that is, this saddle point region can only be described correctly in multireference calculations. The multireference nature of reaction 2a also leaves its mark on the interatomic distances in the two saddle point structures: *r*_C–H_ = 124.4/123.9, *r*_O–H_ = 142.0/141.0 and *r*_N–O_ = 247.9/202.2 pm in the eq/ax forms, respectively.

The PES of reaction 2a was investigated further employing the ωB97XD and BMK functionals, as well as the multilevel correlation single-reference methods CBS-QB3 (B3LYP reference structure) and G3X-K (M06-2X reference structure). The saddle point structures and the spin-contaminations obtained in the ωB97XD and BMK calculations are virtually the same as those of M06-2X and are not documented further here; the saddle point energies to reaction 2a are slightly lower in the ωB97XD than in the M06-2X description, but the other energies are ∼12 kJ mol^–1^ higher. The CC//ωB97XD, CC/BMK, and CC/M062X energies are essentially identical. The CBS-QB3 calculations find a slightly lower barrier of 9 kJ mol^–1^ to the PIPNOȮ equatorial-axial conversion, and slightly higher barriers to imine formation of ∼45 kJ mol^–1^ above the entrance energy of reactants (Table S8). Interestingly, the G3X-K calculations seem to catch the saddle points multiconfigurational calamities and locate SP_eq_^2a^ at ∼50 and SP_ox_^2a^ at ∼25 kJ mol^–1^ above the entrance energy of reactants, as shown in Table S8. While the present paper was in preparation, spin-unrestricted open-shell coupled cluster calculations using UHF-orbitals became available in Molpro (UHF-UCCSD(T)-F12a; acronym: UCC). As UHF-orbitals are expected to form a better basis for UCC calculations, additional UCC//M062X calculations were carried out for reaction 2a. The UCC results, included in Table S8, show more acceptable, but still of concern, T_1_/D_1_ diagnostic values (0.021/0.122 and 0.027/0.141 for SP_ox_^2a^ and SP_eq_^2a^, respectively) and barriers of ∼35 kJ mol^–1^ above the entrance energy of reactants to imine formation.

Reaction 2 was investigated in a master equation model based on the PES illustrated in Figure 4; the PIPṄ + O_2_ association was treated as a reversible reaction and the post reaction complex, PIP-IM*HO_2_, was replaced by the products in the master equation (treating dissociation of the postreaction complex explicitly makes little difference to the outcome of the kinetic modeling). The rate coefficient for the association reaction was approximated by a typical value of 2 × 10^–10^ × (T/298 K)^1/6^ cm^3^ molecule^–1^ s^–1^ from Long-range transition state theory^75^ (this is likely an overestimate of the true association rate coefficient, but a lowering of the value by 50% has no impact on the model results). The barriers to rotation of the -OȮ moiety in PIPNOȮ are quite different in the eq and ax conformations; the potentials obtained in M06-2X/6-31+G(d,p) calculations, shown in Figure S6, warrant that the CNOȮ torsional mode should be treated as a hindered rotor in the model. Treating the CNOȮ torsional mode as a hindered rotor results in an increase in the calculated rate coefficients by a factor of ∼2 at all temperatures. Figure S7 shows the calculated rate coefficient for reaction 2 as a function of temperature stemming from master equation calculations based on the CC//M062X, UCC//M062X, G3X-K, and CCS-QB3 quantum chemistry results; the calculated rate coefficients are essentially independent of pressure in the relevant [*p*,*T*] range. Of concern, the results span 5 orders of magnitude at 298 K, *k*_PIPṄ+O_2_→PIP-IM+HO_2__ = 2.3 × 10^–16^ (CC//M062X), 3.9 × 10^–17^ (UCC//M062X), 2.7 × 10^–18^ (G3X-K), and 8.0 × 10^–21^ (CBS-QB3) cm^3^ molecule^–1^ s^–1^. Disregarding the results based on CC//M062X and CBS-QB3 model chemistry, the atmospheric lifetime of the 1-piperidinyl radical with respect to reaction with O_2_ is then estimated to fall in the range between 5 and 75 ms at 298 K.

We next consider the 1-piperidinyl radical reaction with NO. The reaction occurs without electronic barrier resulting in a vibrationally excited nitrosamine (PIP-NO). We note that there is only one chair-conformation of PIP-NO.
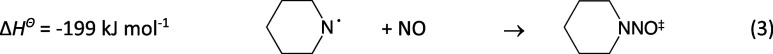
3

Tang et al.^82^ characterized the (CH_3_)_2_Ṅ + NO reaction in B3LYP, MP2, and G4 calculations and showed that the possible internal H-transfer reactions of the highly excited nitrosamine all have barriers of >50 kJ mol^–1^ above the entrance energy of reactants. These routes have similar barriers in PIP-NO and are therefore of little importance under atmospheric conditions. The similar reaction routes involving the higher energy skew conformations of PIP-NO (three pairs of pseudoenantiomeric skew conformations have been located with 14 to 22 kJ mol^–1^ higher energies than the chair forms and will have a relative population of less than 0.6% in total at 298 K) will not affect the thermal stability of PIP-NO. Accordingly, PIP-NO is expected to be thermally stable in the atmosphere.

Nitrosamines, having a characteristic *n* → π* transition in the UV-A region, undergo rapid photolysis in natural sunlight. CAS-SCF calculations on H_2_NNO show that the excited S_1_ state is repulsive, leading to swift dissociation following excitation.^83^ The same is expected for organic nitrosamines, and the quantum yield to photodissociation of (CH_3_)_2_NNO was, conformingly, reported to be 1.03 ± 0.10, following S_0_ → S_1_ (*n*π*) excitation at 363.5 nm.^84^ Hazeldine and Jander reported very comparable UV-spectral data for 1-nitrosopiperidine and dimethyl-, diethyl-, dipropyl-, dipentyl-, and dihexylnitrosamine.^85^ Further, simple TD-DFT calculations (B3LYP/aug-cc-pVTZ) place the first two vertical singlet excitations in H_2_NNO and PIP-NO at, respectively, 372 (oscillator strength *f* = 0.004) and 227 nm (*f* = 0.0112) and 363 (*f* = 0.008) and 237 nm (*f* = 0.026), which are virtually the same values calculated for (CH_3_)_2_NNO^82^ and 1-nitroso-piperazine.^81^ PIP-NO is therefore expected undergo photolysis as fast as (CH_3_)_2_NNO, the threshold wavelength being ∼600 nm
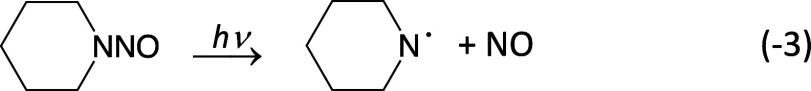
3a

The PIPṄ radical reaction with NO_2_ leads to a vibrationally excited nitramine (PIP-NO_2_^⧧^) that may initiate internal H-transfer from one of the α-CH_2_ groups leading to the corresponding imine (PIP-IM). We note that there is only one chair-conformation of PIP-NO_2_.
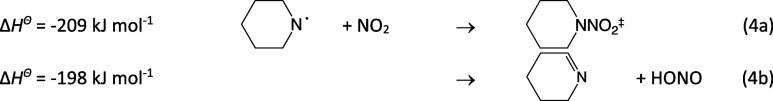
4

The barrier to reaction 4b is found to be 1.3 kJ mol^–1^ below the entrance energy of the initial reactants indicating a small yield under atmospheric conditions.

Lazarou et al. found an additional route in the (CH_3_)_2_Ṅ radical reaction with NO_2_ leading to the (CH_3_)_2_NȮ radical in their very low pressure experiments (∼10^–5^ atm).^86^ This was later corroborated by theoretical calculations of the CH_3_ṄH, (CH_3_)_2_Ṅ, CH_3_CH_2_ṄH and (CH_3_CH_2_)_2_Ṅ reactions with NO_2_ showing that the aminyl radical reaction with NO_2_ can also proceed via a metastable *N*-nitroso-oxy compound.^79^ The present quantum chemistry calculations show that the vibrationally excited *N*-nitroso-oxy piperidine will dissociate without any additional electronic barrier.
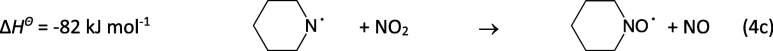
4c

The atmospheric fate of the PIPNȮ radical will be addressed later.

**Figure 5:**
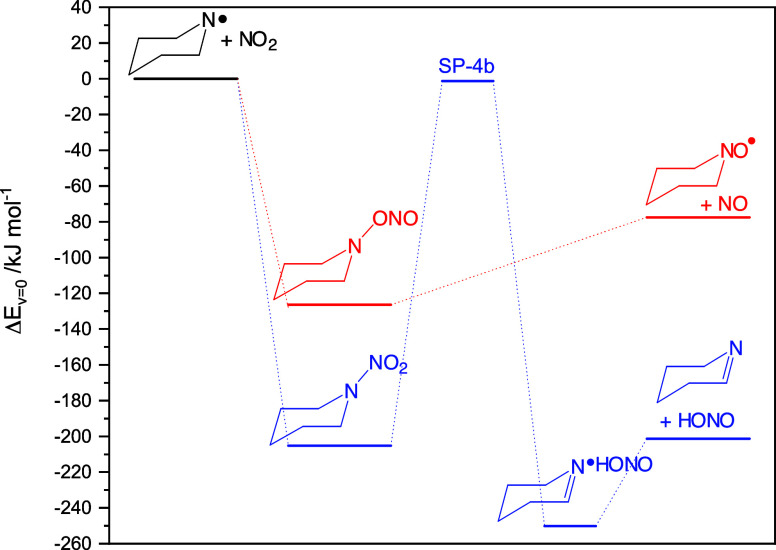
Relative energies of stationary points on the potential energy surface relevant to the 1-piperidinyl radical reaction with NO_2_. Results from CCSD(T*)-F12a/aug-cc-pVTZ//M062X/aug-cc-pVTZ calculations. The underlying quantum chemistry data are included in Table S9.

The energetics of the PIPṄ + NO_2_ reaction are illustrated in Figure 5. Master equation calculations based on the PES shown in Figure 5 signal that reaction 4b is of little importance under atmospheric conditions with ≪1% yield of PIP-IM. Even lowering the barrier to reaction 4b by 50 kJ mol^–1^ will not make any significant difference to the imine-yield, and it will require a lowering of the barrier to reaction 4b by as much as 75 kJ mol^–1^ to have a 1% imine yield. Concerning the possible importance of PIP-NO_2_ skew conformations, three pairs of pseudoenantiomeric skew forms, having from 17 to 21 kJ mol^–1^ higher energies than the chair form, were located. They will be populated by less than 0.2% at 298 K, and they will consequently not contribute notably to the imine yield in reaction 4a

We finally address the PIPṄ + O_3_ reaction. There is no experimental information available on organic aminyl radical reactions with O_3_, but the ṄH_2_ radical is reported to react relatively fast with O_3_ (*k* = 1.7 × 10^–13^ cm^3^ molecule^–1^ s^–1^, extensive review^87^). Peiró–García et al.^88^ investigated the ṄH_2_ + O_3_ reaction in MP2, QCISD, QCISD(T), CCSD(T), CASSCF, and CASPT2 calculations with various basis sets corroborating that the reaction is an oxygen atom transfer from O_3_: ṄH_2_ + O_3_ → H_2_NȮ + O_2_ (^3^Σ_g_ and ^1^Δ_g_).

All attempts to carry out single-reference calculations on the PIPṄ + O_3_ system similar to those presented for ṄH_2_ + O_3_ were unsuccessful (the electronic structures of O_3_ and, in particular, the saddle point of the O_3_ reaction are not described adequately in any single reference quantum chemistry method). The simpler CH_3_ṄH + O_3_ system can, however, be described reasonably well at the MP2 level. The present CH_3_ṄH + O_3_ calculations imply a ∼7 kJ mol^–1^ lower barrier than that calculated for the ṄH_2_ + O_3_ reaction at the CCSD(T)/6-311G(3df,2p)//MP2(Full)/6-311+G(d,p) level,^88^ as shown in Table S10. The CH_3_ṄH + O_3_ reaction is therefore expected to be an order of magnitude faster than the ṄH_2_ + O_3_ reaction, and we suggest that this will also apply to the PIPṄ + O_3_ reaction.
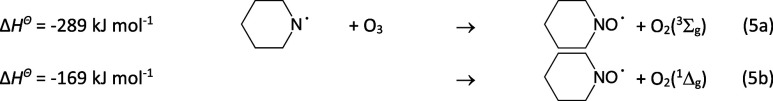
5

Relevant loss processes for the PIPNȮ radical includes reaction with O_3_. The experimental study on the ṄH_2_ + O_3_ reaction by Bulatov et al.^123^ also included the best estimate for the H_2_NȮ + O_3_ reaction: *k*_H_2_NȮ+O3_→ _ṄH_2_+2O_2__ = (2.0 ± 1.5) × 10^–14^ cm^3^ molecule^–1^ s^–1^ at room temperature in agreement with the upper limit of 5 × 10^–14^ cm^3^ molecule^–1^ s^–1^ deducted in experiments by Patrick and Golden.^124^ The computational problems related to the PIPṄ + O_3_ reaction are even more severe for the PIPNȮ + O_3_ reaction. All the same, we boldly suggest that the PIPNȮ reaction with O_3_ will be considerably faster than that of H_2_NȮ.
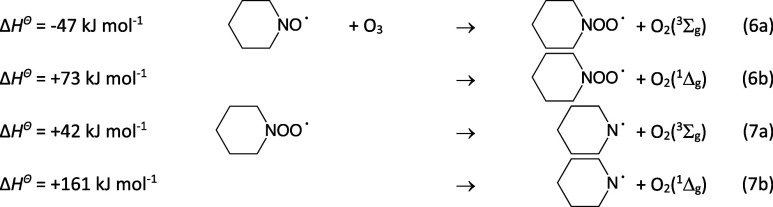
6

The barrier to imine formation (reaction 2a) is around 30 kJ mol^–1^ higher in energy than the dissociation channel (reaction 7) and no significant amount of imine will therefore result directly following reaction 6. We confidently conclude from the above that the PIPṄ radical and the corresponding nitroxide radical (PIPNȮ) will simply react with O_3_ in a cyclic manner regenerating the PIPṄ radical under atmospheric conditions: PIPṄ + O_3_ → PIPNȮ + O_2_, PIPNȮ + O_3_ → PIPṄ +2 O_2_.

**Scheme 1:**
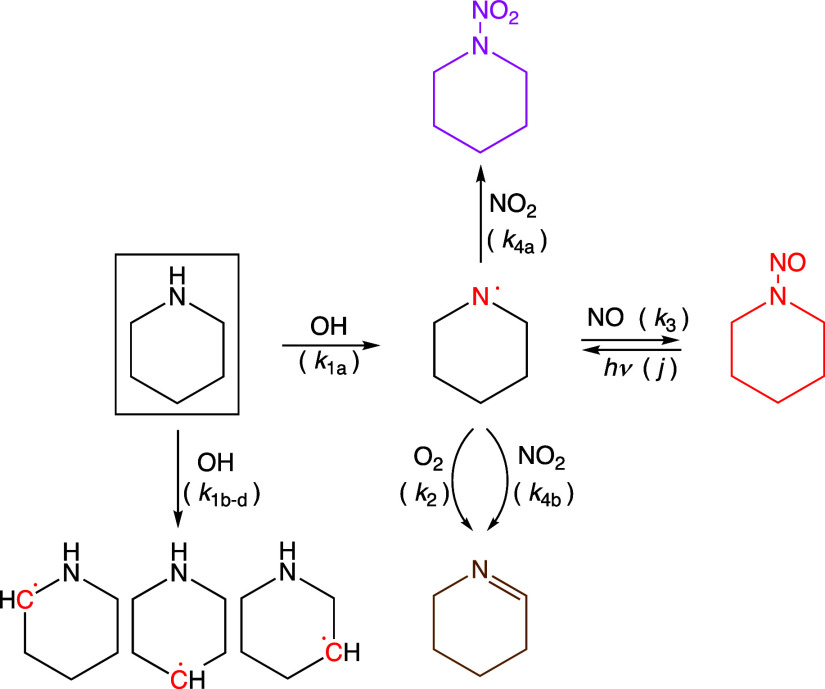
Piperidine Reactions Leading to Nitrosamine and Nitramine Formation in the Atmosphere Note that the present theoretical calculations on the 1-piridinyl radical reactions predict *k*_4b_/*k*_4a_ < 0.01.

In summary, under atmospheric conditions, an initial hydrogen abstraction from the amino group in PIP leads to imine, nitrosamine, and nitramine formation according to Scheme 1 adapted from the generic mechanism proposed by Lindley et al.^89^ for the (CH_3_)_2_Ṅ radical reactions with NO, NO_2_ and O_2_.

##### 3.1.2.2 Atmospheric Fate of the 2-Piperidinyl Radical

The 2-piperidinyl radical, PIPĊ^2^, predicted with a 50% yield in reaction 1, will add O_2_, forming a vibrationally excited peroxy radical, PIPC^2^OȮ^⧧^
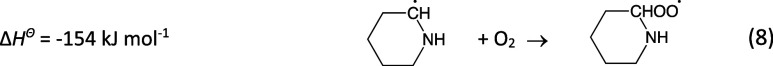
8

There are four distinct chair forms of PIPC^2^OȮ that are labeled ee, ea, ae, and aa according to the equatorial (*e*) or axial (*a*) orientations of the N–H and –OȮ groups, respectively. The ea form has the lowest energy, followed by ee (+16 kJ mol^–1^) and ae and aa (both +22 kJ mol^–1^). In addition, eight pairs of pseudoenantiomeric skew conformations have been located with 18–50 kJ mol^–1^ higher energy than the ea chair form.

**Figure 6:**
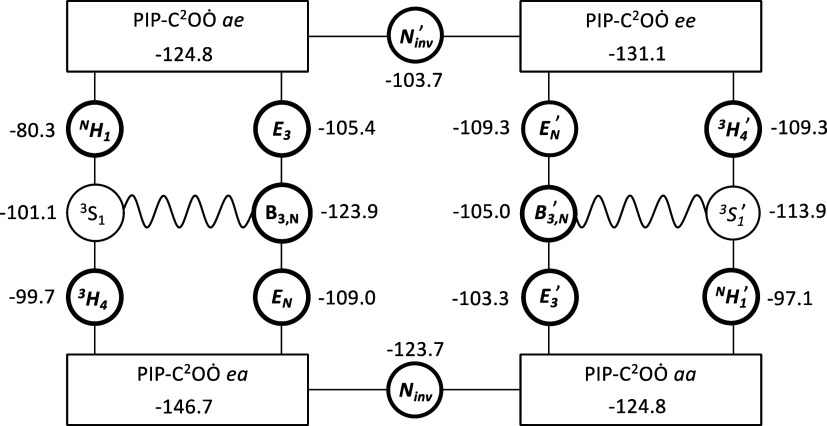
Conformational pathways in 2-piperidinyl peroxy radicals and relative energies in kJ mol^–1^ of stationary points on the potential energy surface relative to that of the initial reactants, PIPĊ^2^ + O_2_. Saddle points are highlighted in bold font; the sine wave represents the pseudorotation consisting of alternate skew and boat conformations. Results from CCSD(T*)-F12a/aug-cc-pVTZ//M062X/aug-cc-pVTZ calculations; the underlying quantum chemistry data are collected in Table S11.

The aa conformer turns out to be metastable with a barrier to N-inversion of only 1.4 kJ mol^–1^ to the minimum energy ea conformer. The ae and ee and the ea and aa conformer pairs are connected via relatively low barriers of ∼20 kJ mol^–1^ to *N*-inversion, whereas the ea and ae and the aa and ee conformers are connected via higher barriers of around 40 kJ mol^–1^ involving a series of linked skew and boat conformers constituting internal pseudorotation in PIPC^2^OȮ, as shown in Figure 6.

The vibrationally excited PIPC^2^OȮ^⧧^ may initiate internal H-transfer reactions before being quenched by collisions and reaction with NO. There are five possible internal H-transfer reactions, of which reaction 9a, leading to the imine (PIP-IM), and reaction 9b, leading to 1,2,3,4-tetrahydropyridine, can be characterized as irreversible, concerted 1,4 H-transfer/C–O bond scission reactions proceeding via HO_2_ complexes on the exit sides. The reaction enthalpies presented refer to the ea conformation.
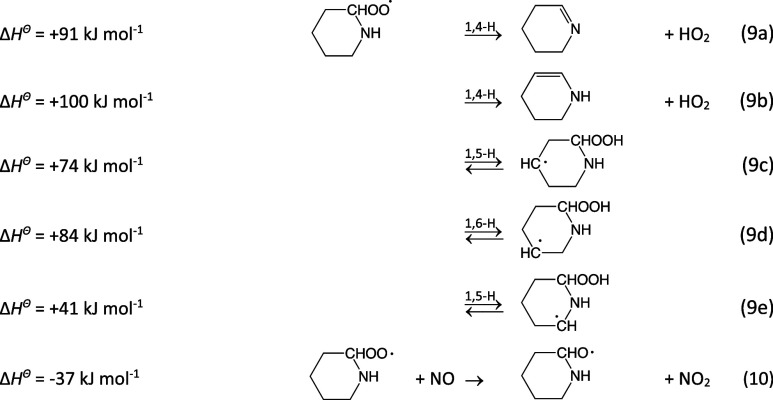
9a

**Figure 7:**
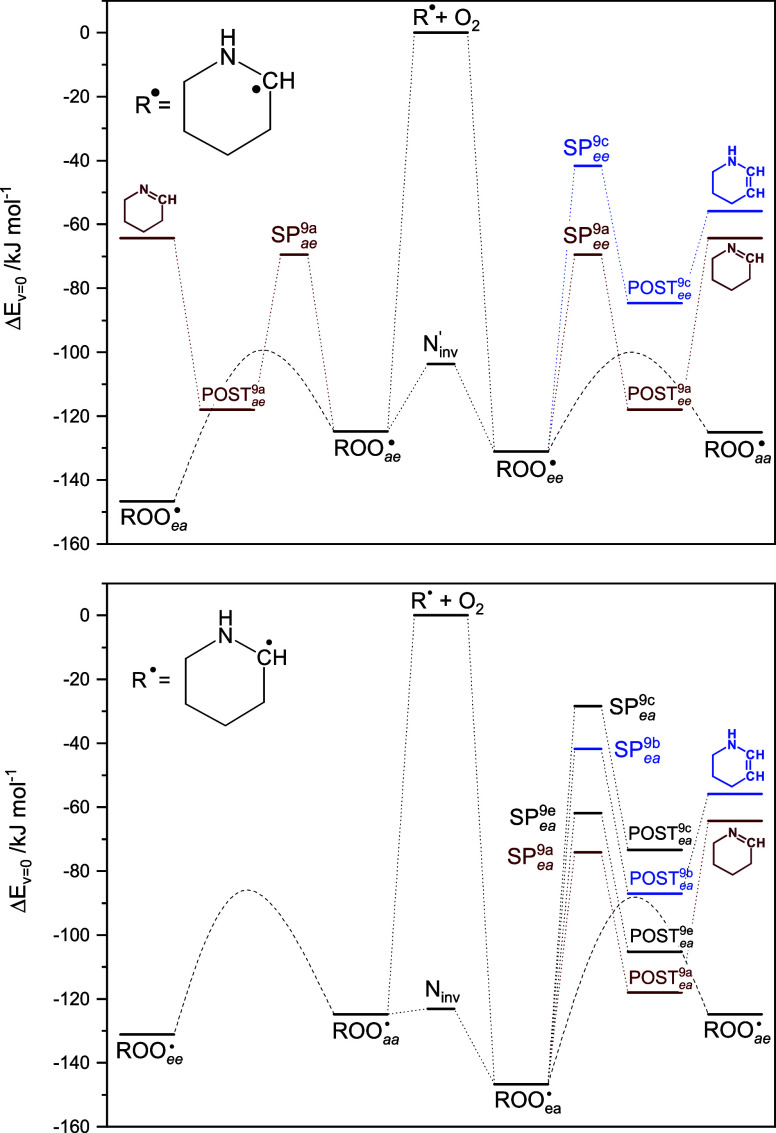
Stationary points on the potential energy surface relevant to the internal H-transfer reactions in the 2-piperidinyl peroxy radical. The high-barrier route 9d as well as routes 9b and 9c within the ae conformational have been left out for the sake of clarity. Top: ea and aa manifold. Bottom: ae and ee manifold. The dashed curves mimic the complex conformational links illustrated in Figure 6. Results from CCSD(T*)-F12a/aug-cc-pVTZ//M062X/aug-cc-pVTZ calculations. The underlying quantum chemistry data for all routes in reaction 9 are included in Table S11.

The barriers to routes 9a–9e are calculated to be 73, 105, 118, 140, and 85 kJ mol^–1^, respectively, in the PIPC^2^OȮ ea conformation. Not all routes of reaction 9 are possible within the three stable conformational subspaces of the PIPC^2^OȮ radical. Reactions 9a and 9e are only realistic in the ea conformation, and the 140 kJ mol^–1^ barrier to reaction 9d is a result of the long distance between the (O)Ȯ and H atoms in C^5^ position. In general, the barriers to H-transfer are substantially higher than the barriers between the conformations. Consequently, the PIPC^2^OȮ conformers will exist in equilibrium throughout the reaction, and the complex conformational pathways illustrated in Figure 6 may therefore be modeled by simple equilibria. Figure 7 presents a simplified outline of the links between stationary points on the PES of the unimolecular PIPC^2^OȮ radical reactions, in which the conformational interchange processes are indicated by dashed curves and routes 9d, having high barriers, have been omitted for simplicity. The same applies to routes 9b and 9c within the ae conformational subspace, as these routes have high barriers of 137 and 152 kJ mol^–1^, respectively.

A total of 8 pairs of pseudoenantiomeric skew PIPC^2^OȮ conformations were located with 18–50 kJ mol^–1^ higher energy than the ea chair form. The relative population of these conformations is <0.1%, and the linked saddle point energies to reactions 9a–e are all located at higher energies than the corresponding saddle points linked to the chair forms. It can therefore be concluded that the skew conformations will only have minute impact on the kinetics of the internal H-transfer reactions in PIPC^2^OȮ.

The reaction sequence 8–10 was simulated in master equation calculations based on the PESes sketched in Figures 6 and 7, including O_2_-sinks for the intermediate alkyl radicals formed in 9c–9e. The rate coefficient of reaction 8 is in the order of 10^–11^ cm^3^ molecule^–1^ s^–1^, and the PIPĊ^2^ radical will consequently not be fully thermalized at the time of peroxy radical formation. Assuming equipartitioning of the reaction enthalpy in reaction 1b and ∼10 quenching collisions before reaction, PIPĊ^2^ could bring as much as an additional ∼60 kJ mol^–1^ into reaction 8.

Assuming *k*_PIPC^2^+O_2__ = 1.3 × 10^–11^, *k*_PIPC^2^OO+NO_ = 6.7 × 10^–12^ cm^3^ molecule^–1^ s^–1^ and that PIPĊ^2^ is vibrationally quenched at the time of reaction, the calculations indicate a PIP-IM yield of >99% for a mixing ratio of NO = 1 ppb; the PIP-IM yield will be slightly lower (still >97%) if the PIPĊ^2^ is vibrationally excited by +60 kJ mol^–1^ at the time of reaction. A concurrent increase in the barriers to imine formation and decrease in the barriers to 1,2,3,4-tetrahydropyridine formation by 4 kJ mol^–1^ lowers the PIP-Im yield to ∼98.5%, which leads to the conclusion that PIP-IM is the by far dominating product in the PIP-C^2^ + O_2_ reaction under atmospheric conditions. In chamber experiments with high-NO*x* conditions of, e.g., 50 ppb NO, the PIP-IM yield is lowered to ∼92%, and the upper limits for PIPC^2^Ȯ and 1,2,3,4-tetrahydropyridine formation are estimated to be 7 and 1%, respectively.

##### 3.1.2.3 Atmospheric Fate of the 3-Piperidinyl Radical

The theoretical calculations predict a 13% yield of the 3-piperidinyl radical (PIPĊ^3^) in reaction 1. Under atmospheric conditions, the PIPĊ^3^ radical will add O_2_ forming a vibrationally excited peroxy radical, PIPC^3^OȮ^⧧^, which may initiate several internal H-transfer reactions before being quenched by collisions and reaction with NO. There are four distinct chair forms of the PIPC^3^OȮ peroxy radical (ee, ea, ae, and aa); the aa form has the lowest energy followed by ee (+1.6 kJ mol^–1^), ae (+3.5 kJ mol^–1^), and aa (+3.7 kJ mol^–1^). The ae and ee and the ea and aa conformers are connected via relatively low barriers of ∼20 kJ mol^–1^ to *N*-inversion, whereas the ea and ae and the aa and ee conformers are connected via higher barriers of around 40 kJ mol^–1^ involving a series of linked skew and boat conformers; internal pseudorotation in PIPC^3^OȮ is similar to the setting outlined in Figure 6 for PIPC^2^OȮ. The energies and conformational pathways involving skew and boat forms of PIPC^3^OȮ were not investigated in detail, but a total of 12 pairs of pseudoenantiomeric skew PIPC^3^OȮ conformations were located with 21–32 kJ mol^–1^ higher energy than the aa chair form. The relative population of these conformations is <0.1% in total, and it is presumed that internal H-transfer reactions involving the PIPC^3^OȮ skew conformers can be omitted in modeling the atmospheric fate of the PIPC^3^OȮ radical.

Excluding the high barrier 1,6-H transfer reactions from C^6^ position (cf. Section 3.1.2.2) involving a skew conformation, the possible routes are
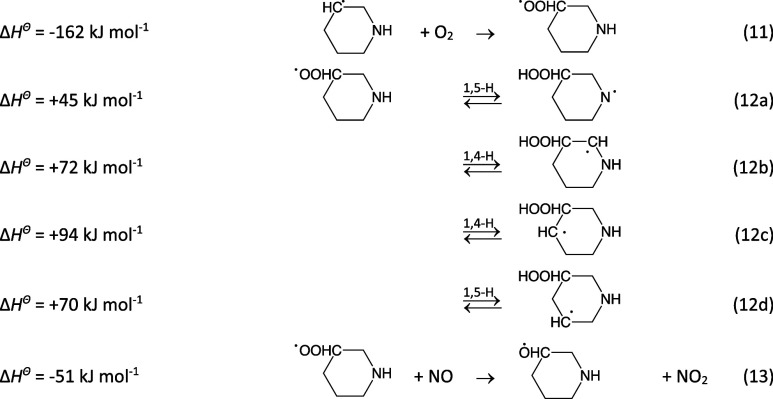
11

**Figure 8:**
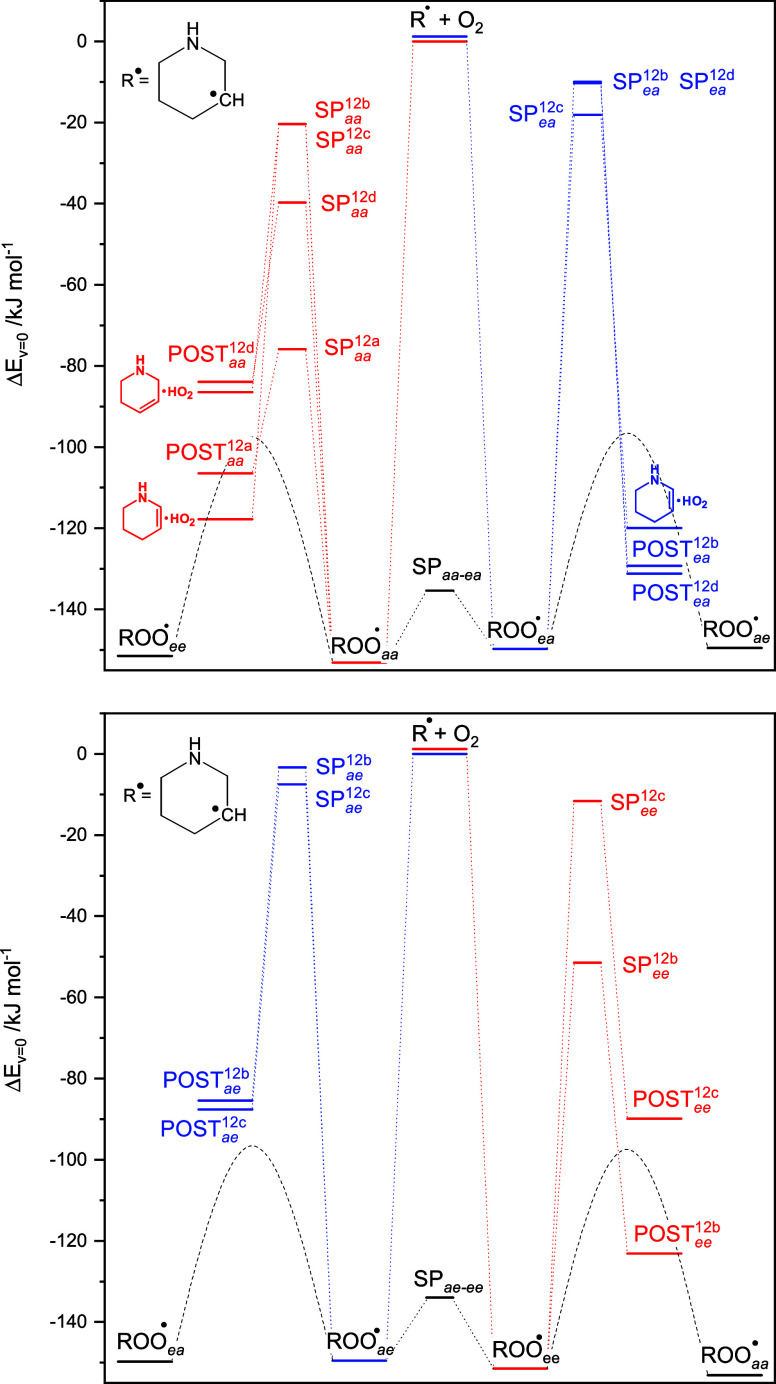
Stationary points on the potential energy surface relevant to the internal H-transfer reactions in the 3-piperidinyl peroxy radical. Top: ea and aa manifold. Bottom: ae and ee manifold. The dashed curves mimic the complicated conformational links between the ea and ae and the ee and aa conformers. Results from CCSD(T*)-F12a/aug-cc-pVTZ//M062X/aug-cc-pVTZ calculations. The underlying quantum chemistry data are collected in Table S12.

Not all routes of reaction 12 are feasible within each of the four conformational subspaces of the PIPC^3^OȮ radical. Route 12a is only possible in the aa conformation, and route 12d is only accessible in the aa and ea conformations. The aa conformation routes 12b and 12c and the ea conformation route 12c can be characterized as irreversible, concerted 1,4 H-transfer/C–O bond scission reactions proceeding via HO_2_ complexes with tetrahydropyridines on the exit sides. The QOOH radical formed in the ee conformer route 12b has an additional barrier of around 60 kJ mol^–1^ to HOO ejection; the latter detail has not been included in Figure 8 illustrating the energetics of the PIPC^3^OȮ radical H-transfer reactions. The complex conformational pathways linking the ea and ae conformers, and the aa and ee conformers are represented by dashed parabolic curves.

The reaction sequence 11−13 was simulated in master equation calculations based on the PES sketched in Figure 8, including O_2_-sinks (*k*_QOOH+O_2__ = 1.3 × 10^–11^ cm^3^ molecule^–1^ s^–1^) for the intermediate alkyl radicals formed in 12b−12d and an aminyl radical O_2_ sink (*k*_RṄ+O_2__ = 10^–17^ – 10^–18^ cm^3^ molecule^–1^ s^–1^, see Section 3.1.2.1). The rate coefficient of reaction 11 is in the order of 10^–11^ cm^3^ molecule^–1^ s^–1^, and the PIPĊ^3^ radical will consequently not be fully thermalized at the time of peroxy radical formation. Assuming equipartitioning of the reaction enthalpy in reaction 1c and ∼10 quenching collisions before reaction, PIPĊ^3^ could bring as much as an additional ∼40 kJ mol^–1^ into reaction 11.

A quick perusal of Figure 8 hints that only routes 12a and 12d for the aa conformer and route 12b for the ee conformer may compete with the PIPC^3^OȮ + NO sink (reaction 13). Assuming *k*_13_ = 6.7 × 10^–12^ cm^3^ molecule^–1^ s^–1^ (see above) and that PIPĊ^3^ is vibrationally quenched at the time of reaction, the calculations indicate a PIPC^3^Ȯ yield of 57, 36% of 1,2,3,4-tetrahydropyridine and 7% of the 
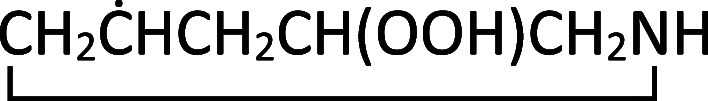
 radical for a mixing ratio of NO = 20 ppt. In the case that PIPĊ^3^ carries 40 kJ mol^–1^ additional internal energy into reaction 11, the branching becomes completely different: 13% PIPC^3^Ȯ, 85% 1,2,3,4-tetrahydropyridine, and 2% 
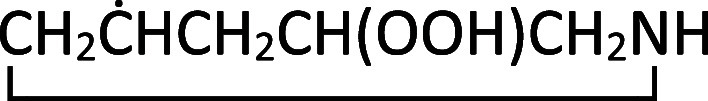
. The branching is only slightly sensitive to ⟨Δ*E*⟩_down_—a decrease of ⟨Δ*E*⟩_down_ from 250 to 150 cm^–1^ only changes the above-mentioned yields to 52, 41, and 6%. A simultaneous decrease of all barriers to H-transfer by 4 kJ mol^–1^ lowers the PIPC^3^Ȯ yield to 45%, whereas 1,2,3,4-tetrahydropyridine and the 
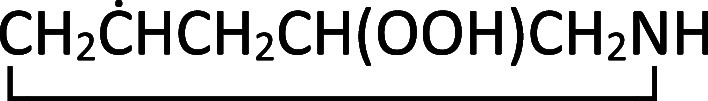
 radical yields increase to 18 and 47%, respectively.

Under atmospheric conditions with NO ≥ 1 ppb, the yields become >98% PIPC^3^Ȯ and <2% 1,2,3,4-tetrahydropyridine when PIPĊ^3^ is vibrationally quenched at the time of reaction with O_2_, and ∼87% PIPC^3^Ȯ and ∼13% 1,2,3,4-tetrahydropyridine when an additional 40 kJ mol^–1^ internal energy is carried over into the reaction with O_2_. In summary, the product distribution of the reaction sequence 11−13 is very dependent on the NO level and the extent of collisional quenching prior to the PIPĊ^3^ + O_2_ reaction. Considering the uncertainties involved in the reaction modeling, we tentatively suggest that the reaction sequence 11−13 results in 85 ± 10% PIPC^3^Ȯ and 15 ± 10% 1,2,3,4-tetrahydropyridine.

The PIPC^3^Ȯ radical also exists in four distinct chair forms; the aa form has the lowest energy, followed by ae (+0.5 kJ mol^–1^), ee (+3.4 kJ mol^–1^), and ea (+11.3 kJ mol^–1^), the latter being metastable with a barrier of only 5.8 kJ mol^–1^ (M062X/aug-cc-pVTZ) to the aa form. The CC//M062X calculations place the saddle point 5.6 kJ mol^–1^ lower in energy than the ea form, but, as the diagnostic values of the CCSD(T) calculation are rather high (*T*_1_ = 0.026, *D*_1_ = 0.148), the single-reference-based method will likely not give highly accurate results. In any case, the PIPC^3^Ȯ ea conformer will spontaneously rearrange to the aa conformer. The ae and ee conformers are connected via a relatively low barrier of ∼20 kJ mol^–1^ to *N*-inversion, whereas the ea and ae and the aa and ee conformers are, presumably, connected via higher barriers of around 40 kJ mol^–1^ involving a series of linked skew and boat conformers constituting internal pseudorotation in PIPC^3^Ȯ resembling the situation in PIPC^3^OȮ (12 pairs of pseudoenantiomeric skew PIPC^3^Ȯ conformations were located with 21–40 kJ mol^–1^ higher energy than the aa chair form).

The PIPC^3^Ȯ radical will either undergo H-abstraction of the α-hydrogen atom by O_2_, ring rupture, or internal H-transfer reactions. The enthalpies of reaction given below refer to the aa conformation.
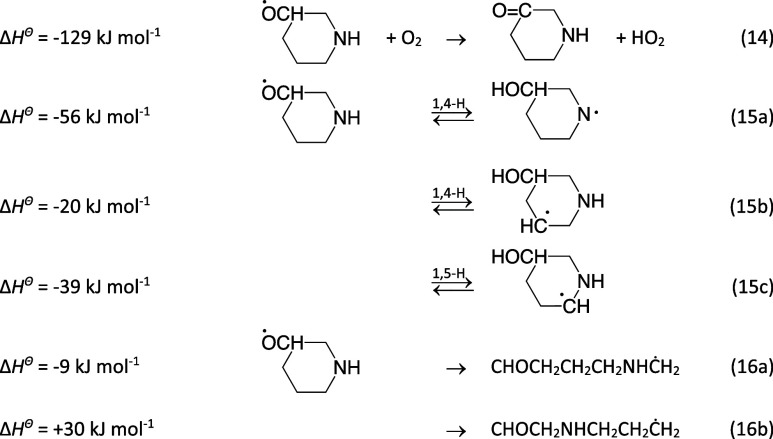
14

**Figure 9:**
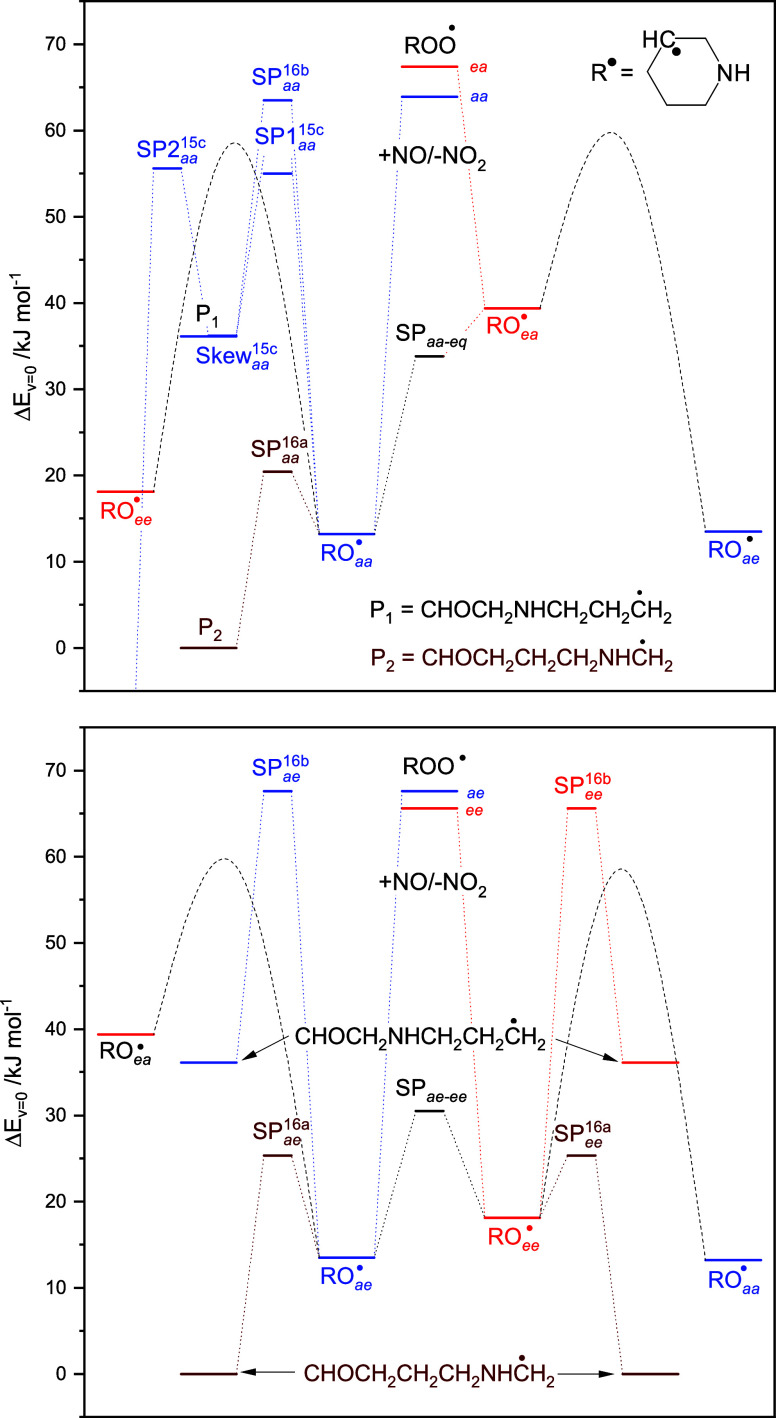
Stationary points on the potential energy surface relevant to the unimolecular reactions of the 3-piperidinyl oxy radical, PIPC^3^Ȯ. For the sake of simplicity, the high-barrier 1,4-H transfer reactions 15a and 15b have not been included in the figure. Top: ea and aa manifold. Bottom: ae and ee manifold. The dashed curves mimic the complicated conformational links between the ea and ae and the ee and aa conformers. Results from CCSD(T*)-F12a/aug-cc-pVTZ//M062X/aug-cc-pVTZ calculations. The underlying quantum chemistry data are collected in Table S13.

Not all routes in reaction 15 are feasible within each of the four conformational subspaces of the PIPC^3^Ȯ radical. Route 15a is only possible in the aa conformation, and route 15b is only accessible in the aa and ea conformations. The barriers to routes 15a and 15b are calculated to be well above the entrance energy of reactants, but the barriers to 15c are below; the 1,5-H shift reaction occurs via envelope-like saddle points (SP1^15c^) linking the chair conformations of PIPC^3^Ȯ to a skew conformer (Skew^15c^) and a boat conformer (SP2^15c^). Importantly, the barriers to C^2^–C^3^ scission (route 16a) are very low −7.2 (aa), 11.8 (ae), and 7.1 kJ mol^–1^ (ee)—whereas the barriers to C^3^–C^4^ scission (route 16b) are much higher: 50.3 (aa), 54.1 (ae), and 47.5 kJ mol^–1^ (ee). Figure 9 illustrates the links between stationary points on the PES of the unimolecular PIPC^3^Ȯ radical reactions; the complex conformational pathways linking the ea and ae conformers and the aa and ee conformers are represented by dashed parabolic curves.

A quick perusal of Figure 9 leaves little doubt that route 16a will dominate the atmospheric fate of PIPC^3^Ȯ. To confirm, the competition between reactions 14−16 was simulated in master equation calculations based on the PES sketched in Figure 9, including O_2_ sinks for radicals formed in reaction 15, *k*_>CH+O_2__ = 1.3 × 10^–11^ cm^3^ molecule^–1^ s^–1^ and an O_2_ sink for the 1-aminyl radical, *k*_>N+O_2__ = 1.0 × 10^–17^ cm^3^ molecule^–1^ s^–1^; see Section 3.1.2.1. Assuming *k*_14_ = 1.8 × 10^–14^ cm^3^ molecule^–1^ s^–1^ and equipartition of the enthalpy in reaction 13, the calculations show >99.9% yield of the CHOCH_2_CH_2_CH_2_NHĊH_2_ radical under atmospheric conditions. It makes essentially no difference to the yields if all the available enthalpy in reaction 13 is placed in the oxy-radical.

The CHOCH_2_CH_2_CH_2_NHĊH_2_ radical will add O_2_, resulting in a vibrationally excited peroxy radical that may initiate internal H-transfer reactions before being quenched by collision and reaction with NO. There are five possible internal H-transfer reactions, of which the 1,4-H transfer route turns out to be irreversible and results in imine formation via a post reaction complex with HO_2_
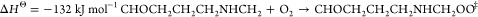
17
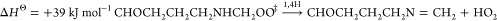
18a
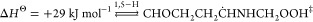
18b
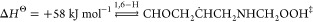
18c
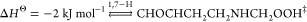
18d
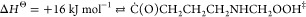
18e
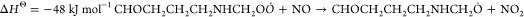
19

Considering the internal energy available in CHOCH_2_CH_2_CH_2_NHCH_2_OȮ^⧧^, there is a plethora of conformations accessible, and the lowest barriers to reactions 18a through 18e were found to be 77, 91, 102, 83, and 64 kJ mol^–1^ (the rotational barriers linking the various conformations are lower and can be disregarded). The links between stationary points on the PES of reactions 17–18 are sketched in Figure S8, and the underlying quantum chemistry data are collected in Table S14.

The competition between reactions 18 and 19 was modeled in master equation calculations including O_2_ sinks with typical values of 1 × 10^–11^ for the alkyl^90^ and 5 × 10^–12^ cm^3^ molecule^–1^ s^–1^ for the acyl radicals formed in reactions 18b through 18e. Taking *k*_19_ = 1 × 10^–11^ cm^3^ molecule^–1^ s^–1^, the calculations show less than 1% yield of the oxy radical for NO levels below 40 ppb and a branching of 26:1:0:1:72 in reaction 18. Changing *k*_18e_ by a factor of 2 has virtually no impact on the branching, but setting ⟨Δ*E*⟩_down_ = 150 cm^–1^ changes the branching to 64:1:0:1:34. The barrier to imine formation, reaction 18a, is of the same height as in CH_3_NHCH_2_OȮ,^91^ and, although the 1,8-H transfer reaction has the lowest barrier, it is somewhat surprising finding this channel being predominant despite the entropic penalty that accompanies formation of a nine-member cyclic transition state.^92^ Regarding the barrier heights, the branching is essentially only sensitive to the barrier to imine formation in route 18a. Assuming an uncertainty in this barrier of ±4 kJ mol^–1^ and ⟨Δ*E*⟩_down_ to be somewhere in the region 150–250 cm^–1^ confines the predicted branching 18a: 18e to the rather wide range 13–64:84–34. There are obviously additional saddle point conformers to each reaction route. However, it makes virtually no difference to the calculated branching if the second lowest energy saddle point conformers are included in the calculations. Considering the assumptions and approximations involved in the quantum chemistry and the master equation model, we conclude that the imine and acyl radical yields in reactions 17–19 are around 40 and 60 ± 20%, respectively, under atmospheric conditions.

The Ċ(O)CH_2_CH_2_CH_2_NHCH_2_OOH acyl radical is expected to add O_2_ forming a highly vibrationally excited peroxy acyl radical that may induce internal H-transfer reactions before being quenched and reacting with NO or NO_2_. Of the six possible internal H-transfer reactions, the 1,8-H transfer turns out to be irreversible and resulting in OH regeneration and amide formation
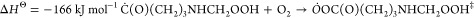
20
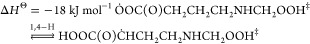
21a
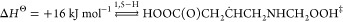
21b
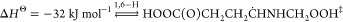
21c
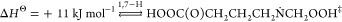
21d
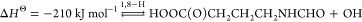
21e
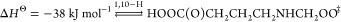
21f
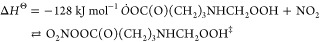
22
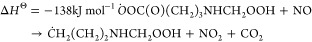
23

Again, there is a plethora of ȮOC(O)(CH_2_)_3_NHCH_2_OOH conformations accessible, and the lowest barriers to reactions 21a through 21f are found to be 113, 86, 53, 76, 61, and 52 kJ mol^–1^ at the M062X/aug-cc-pVTZ level of theory. Although energy results obtained at this level of theory should be viewed with some caution (CC//M062X calculations on these species are very memory and CPU time demanding), it is clear that routes 21c and 21f will be dominant. Consequently, modeling the atmospheric fate of Ċ(O)(CH_2_)_3_NHCH_2_OOH requires inclusion of the additional four possible internal H-transfer reactions that the intermediate peroxy radical, HOOC(O)CH_2_CH_2_CH_2_NHCH_2_OȮ^⧧^, formed in 21f, may initiate before quenching, and reaction with NO. It turns out that the 1,4-H shift leading to imine formation is irreversible and proceeding via a complex with HO_2_ on the exit side.
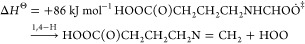
24a
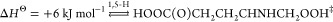
24b
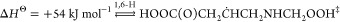
24c
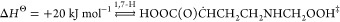
24d
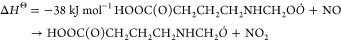
25

The barriers to reactions 24a–24d are calculated to be 98, 79, 106, and 84 kJ mol^–1^, respectively; route 24b, having the lowest barrier, leads to the same radical as 21c.

The links between the stationary points on the PES of reactions 20–24 are sketched in Figure S9, while the quantum chemistry data are collected in Table S15. The competition between reactions 20–25 was estimated in master equation calculations including O_2_ sinks with typical values of 1 × 10^–11^ cm^3^ molecule^–1^ s^–1^ for the alkyl radicals formed in reactions 21 and 24, and *k*_22_ = 1 × 10^–11^ and *k*_23_ = 2 × 10^–11^ cm^3^ molecule^–1^ s^–1^ (the values for the peroxyacetyl radical),^90^ and a typical value of 9 × 10^–12^ cm^3^ molecule^–1^ s^–1^ for *k*_25_.^90^Reaction 22 was initially treated as irreversible to estimate the maximum amount of the PAN-like compound that could possibly be formed; the model shows that reactions 22 and 23 can be ignored for NO and NO_2_ levels below 100 ppb.

The importance of reaction 25 is subject to the assumed level of NO. For remote areas with NO levels of 20 ppt, the model predicts a small yield of ∼2% oxy radical formation, whereas the sum of routes 21b and 24c accounts for ∼84% and route 23d for ∼14%. In urban areas with NO levels of 1–10 ppb, the oxy radical yield is predicted to fall in the region 37–60% with the sum of routes 21b and 24c accounting for ∼38% and route 24d for only ∼1%.

**Scheme 2:**
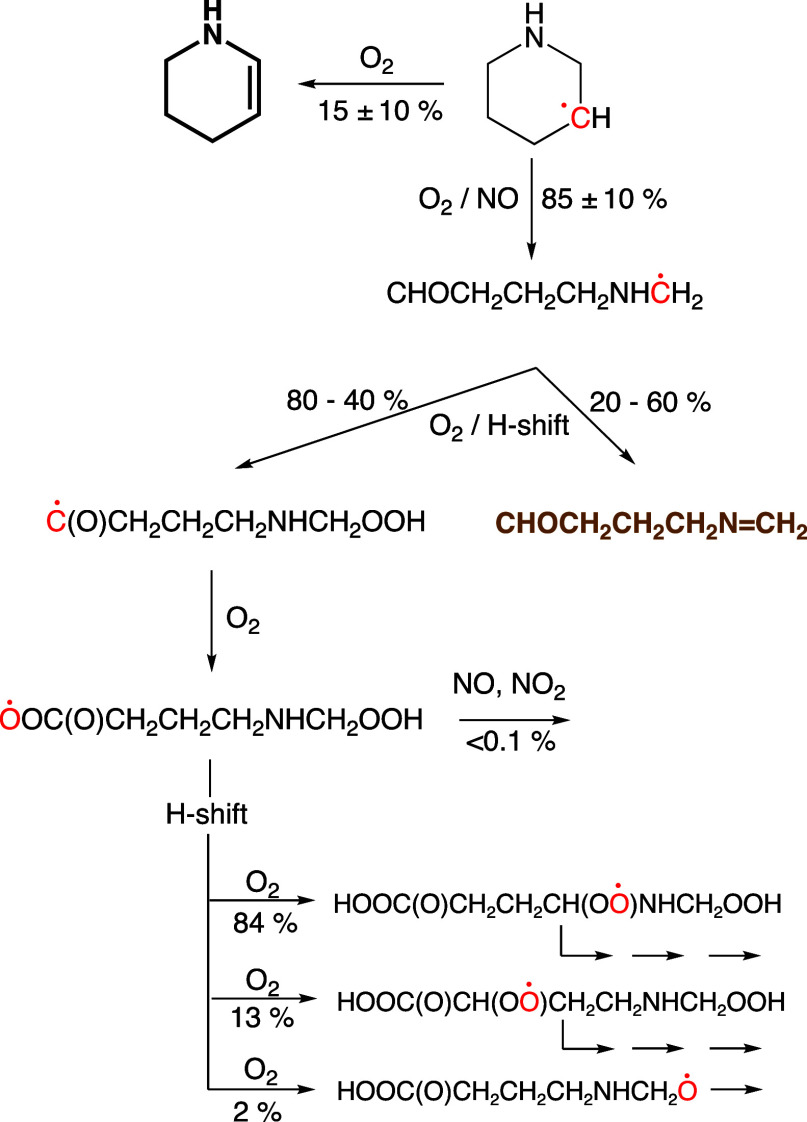
Atmospheric Reactions of the 3-Piperidinyl Radical (PIPĊ^3^) Thermally stable products are typeset in bold font; radical sites are indicated in red.

The QOOH radicals formed in reactions 21 and 24 will add O_2_, resulting in a highly vibrationally excited peroxy radicals that each may induce further internal H-transfer reactions before being quenched and/or reacting with NO. Also, the alkoxy radical formed in reaction 25 may initiate internal H-transfer reactions, in particular H-transfer from the peroxyacid group, competing with dissociation and H-abstraction by O_2_. These autoxidation reactions will, however, not be pursued further here. The first steps in the atmospheric reactions the PIPĊ^3^ radical are outlined in Scheme 2.

##### 3.1.2.4 Atmospheric Fate of the 4-Piperidinyl Radical

The theoretical calculations predict around 2% yield of the 4-piperidinyl radical (PIPĊ^4^) in reaction 1; details on the atmospheric reactions of PIPĊ^4^ are placed and illustrated in Figures S10–S11 in the Supporting Information (underlying quantum chemistry data are collected in Tables S16–S17). In summary, the major products following H-abstraction from C^4^ are projected to be ∼20% CHOCH_2_CH_2_NHCH_2_ĊH_2_, ∼30% 2,3,4,5-tetrahydropyridin-4-ol and ∼50% piperidin-4-one. The alkyl radical, CHOCH_2_CH_2_NHCH_2_ĊH_2_, is expected to undergo autoxidation reactions similar to those outlined for CHOCH_2_CH_2_CH_2_NHĊH_2_ in Section 3.1.2.3.

##### 3.1.2.5 Photo-Oxidation Mechanism

**Scheme 3:**
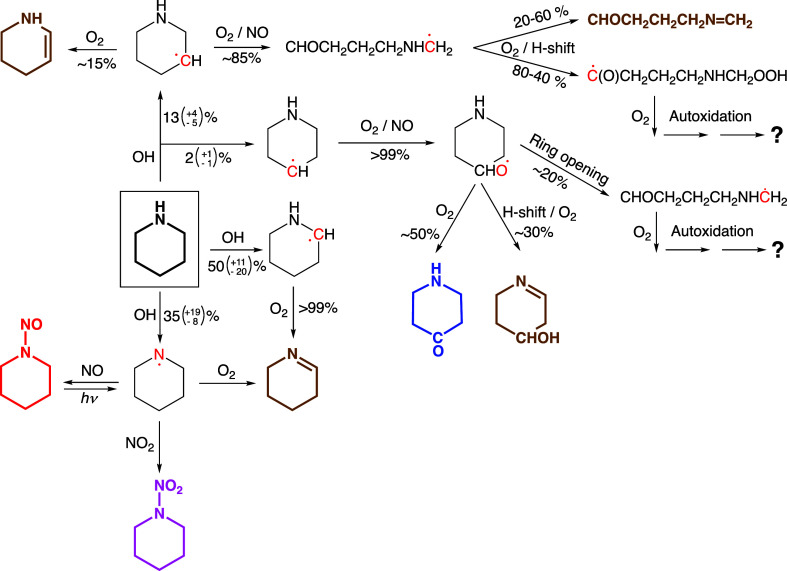
Major Routes for the OH Initiated Photo-Oxidation of Piperidine under Atmospheric Conditions as Resulting From Theoretical Calculations Conservative limits to estimated branchings are given in parentheses; thermally stable products are typeset in bold font; radical sites are indicated in red.

Scheme 3 summarizes the major atmospheric degradation routes of PIP obtained from master equation calculations based on quantum chemistry results detailed in the previous sections. Tentative limit estimates to the calculated branching in the initial PIP + OH reaction are given in parentheses.

Scheme 3 shows that the four routes in the PIP + OH reaction result in different products, and it should therefore, in principle, be possible to quantify the initial branching in photo-oxidation experiments. The two dominating H-abstraction routes (C^2^ and N^1^) are estimated to account for 85% of the PIP reactivity toward OH, both result in 2,3,4,5-tetrahydropyridine (PIP-IM) as the major primary product. The products resulting in the autoxidation following H-abstraction from C^3^ are highly functionalized, and they are therefore very likely to partition into the particle phase.

### 3.2 Experimental Results

We first report results from kinetic studies of the PIP + OH reaction carried out in the EUPHORE atmospheric simulation chamber B. We then focus on results from PIP-NO photolysis experiments before addressing the atmospheric photo-oxidation of PIP and the initial branching between H-abstraction from the NH and CH_2_ groups in the PIP + OH reaction. Finally, we present results from studies of the particles formed during the PIP photo-oxidation experiments.

#### 3.2.1 Piperidine + OH Reaction Kinetics

Two relative rate experiments were carried out on 2016.07.19 employing three reference compounds: styrene, 1,2,3-trimethylbenzene, and isoprene. Acetonitrile was added as an inert tracer to monitor the apparent dilution by purified air that was constantly added to compensate for leakage and continuous sampling by the air monitors (*k*_OH+CH_3_CN_ = 2.2 × 10^–14^ cm^3^ molecule^–1^ s^–1^ at 298 K).^90^ OH radicals were generated employing 2-propylnitrite (IPN) as the precursor: CH_3_CH(ONO)CH_3_ + *h*ν → CH_3_CH(Ȯ)CH_3_ + NO; CH_3_CH(Ȯ)CH_3_ + O_2_ → CH_3_C(O)CH_3_ + HO_2_; HO_2_ + NO → OH + NO_2_.

**Figure 10:**
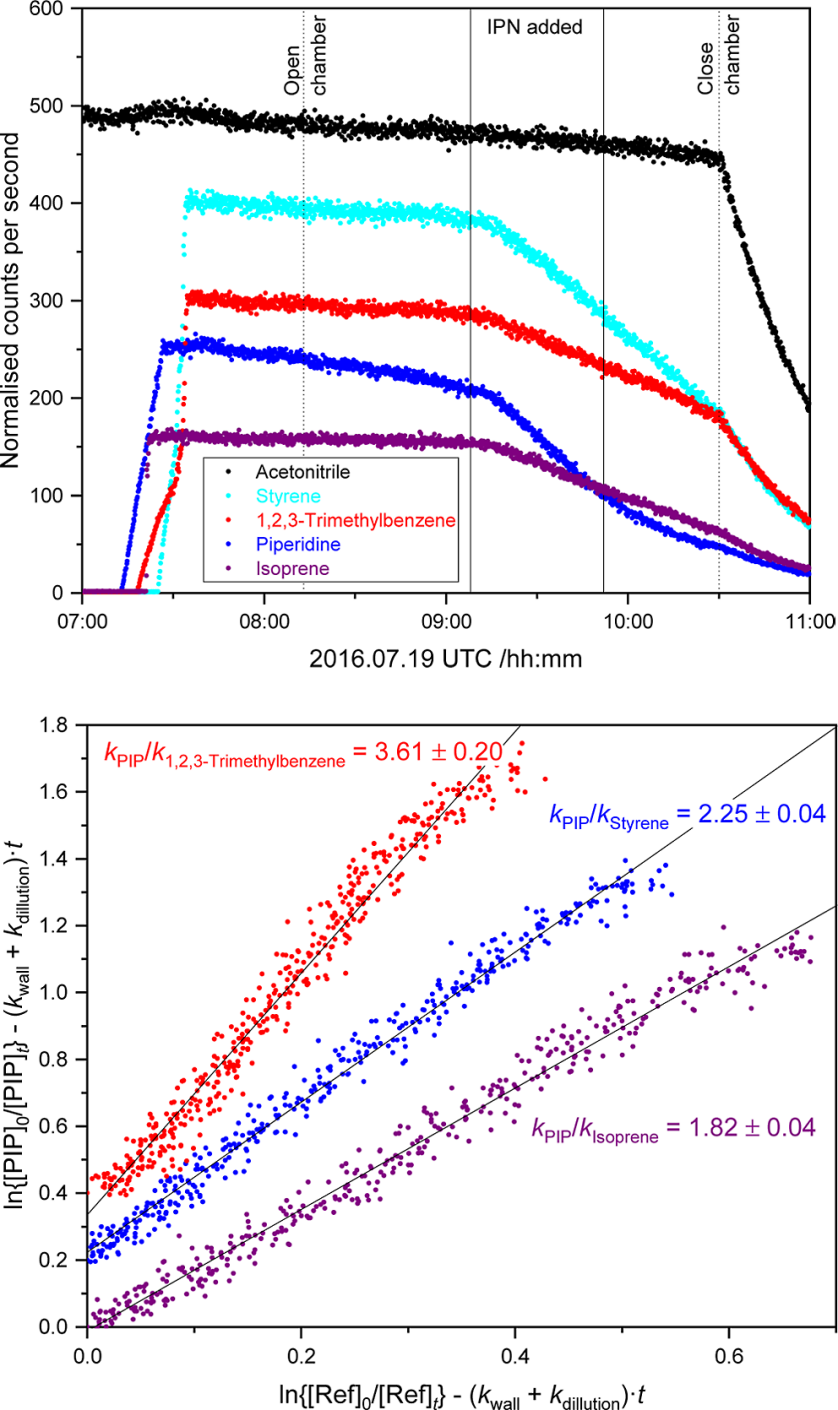
Top panel: Time evolution of acetonitrile, styrene, 1,2,3-trimethylbenzene, piperidine (PIP), and isoprene during the first kinetic experiment on 2016.07.19. Bottom panel: relative rate plots showing the decays of piperidine, styrene, and 1,2,3-trimethylbenzene in the presence OH radicals. For the sake of clarity, the data have been displaced along the abscissa. The data have been corrected for dilution due to chamber air replenishment and wall loss. Uncertainties given are 3 times the standard error of the least-squares fit.

Figure 10 displays the time profiles of acetonitrile, styrene, 1,2,3-trimethylbenzene, piperidine, and isoprene (detected at *m*/*z* 42.034, 105.070, 121.102, 86.095, and 69.070, respectively) during the first of the two kinetic experiments (1014 ± 2 hPa, 304 ± 2 K). The apparent dilution rate, due to air replenishment, was 8.0 × 10^–6^ s^–1^, and the PIP wall loss rate, derived from the decay prior to adding IPN, was around 3 times larger (*k*_dillution_ + *k*_wall_ = 3.4 × 10^–5^ s^–1^). As can be seen, the reference compounds wall losses are practically negligible. Results from the second kinetic experiment are presented in Figure S12.

Preliminary findings from a kinetic experiment, in which the OH reactivities of PIP, dimethyl- and methylpropylamine were compared relative to tetrahydrofuran and 1,3,5-trimethylbenzene, have appeared as part of the “Atmospheric Degradation of Amines” (ADA) project summary report.^17^ The data have been reanalyzed, and the results are presented in Figure S13.

Table S18 summarizes the kinetic results and includes the currently recommended values for the absolute OH rate coefficients for the reference compounds.^93^ The relative rate data obtained with isoprene as reference results in an almost 50% larger absolute rate coefficient for the PIP + OH reaction than the average of all data. We tentatively suggest that there is an interference in the *m*/*z* 69.070 ion signal, attributed to isoprene, and offer the average value of (1.19 ± 0.27) × 10^–10^ cm^3^ molecule^–1^ s^–1^ from the other reference compounds as a best estimate for the piperidine + OH rate coefficient at 1014 ± 2 hPa and 304 ± 2 K.

There was a moderate particle formation during the kinetic experiments (see also Section 3.2.4 later). The particle formation was not characterized in detail, and the kinetic data have therefore not been corrected for gas-particle transfer as was done in our previous piperazine study,^81^ where information on the particle composition was available. Inclusion of gas-particle transfer in the analyses of the kinetic data for piperazine resulted in a lowering of the rate coefficient by ∼7%. Since the particle formation in the piperidine experiments is much smaller than in the corresponding piperazine experiments, we are confident that derived piperidine rate coefficient is not severely affected by particle formation.

#### 3.2.2 1-Nitrosopiperidine Photolysis Studies

***Warning**. N-Nitrosopiperidine should be handled with utmost caution; it is reasonably anticipated to be a human carcinogen based on sufficient evidence of carcinogenicity from studies in experimental animals.*(94)

Three photolysis experiments were carried out in the EUPHORE chamber B under different NO*x* conditions in 2011; preliminary findings have appeared as part of the “Atmospheric Degradation of Amines” (ADA) project summary report that also contains information on monitor and *j*_NO_2__ calibration.^17^ A subsequent quality control of the project data showed nonlinearity effects in the PTR-ToF-MS microchannel plate (MCP) detector warranting reanalysis of the PIP-NO photolysis data.^18^

In the photolysis experiments, SF_6_ and varying amounts of NO, NO_2_ (NO + O_3_) were injected into the chamber prior to adding the nitrosamine in a stream of N_2_. Excess cyclohexane (∼2 ppm) was included in order to quantify the amount of OH radicals formed following PIP-NO photolysis (PIP-NO + *h*ν → PIPṄ + NO; PIPṄ + O_2_ → PIP-IM + HO_2_; HO_2_ + NO → OH + NO_2_). When the substances were well mixed, the chamber canopy was opened to natural sunlight and the nitrosamine was largely photolyzed within 30 min. Figures S14 and S15 illustrates *p*, *T*, *j*_NO_2__, NO, NO_2_, and O_3_ during the experiments.

**Figure 11:**
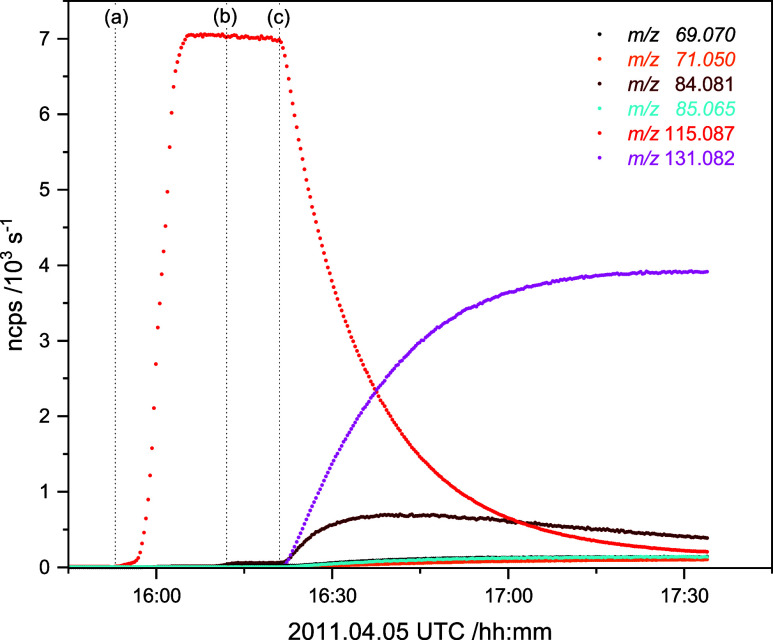
PTR-TOF-MS ion signals (ncps) observed during the 1-nitrosopiperidine photolysis experiment on 2011.04.05. (a) Start injection of PIP-NO, (b) start injection of cyclohexane, and (c) open chamber canopy to solar irradiation.

Figure 11 shows the time profiles of PIP-NO (*m*/*z* 115.087, C_5_H_11_N_2_O^+^), PIP-NO_2_ (*m*/*z* 131.082, C_5_H_11_N_2_O^+^), and PIP-IM (*m*/*z* 84.081, C_5_H_10_N^+^) during the photolysis experiment on 2011.04.05. In addition to signals obviously related to cyclohexanone (see below), only three ion signals were growing in with intensities above 1% of the intensity loss in the PIP-NO ion signal: *m*/*z* 69.070 (C_5_H_9_^+^), 71.050 (C_4_H_7_O^+^), and 85.065 (C_5_H_9_O^+^). It should be noted that the three additional signals all grow with intensities below 2% of the intensity loss in the PIP-NO ion signal, and we abstain from presenting a definite molecular interpretation. An inspection of the ion signals observed in the time period before opening the chamber canopy to sunlight reveals that [PIP-NO]H^+^ hardly fragments at the instrumental settings employed (*E*/*N* = 88 Td): 99.7% *m*/*z* 115.087 (protonated molecule), ∼0.1% *m*/*z* 84.081 (HNO ejection), and ∼0.2% *m*/*z* 85.089 (NO ejection). PIP-NO_2_ calibration experiments show that [PIP-NO_2_]H^+^ fragments slightly more: ∼97% *m*/*z* 131.082 (protonated molecule), ∼2% *m*/*z* 84.081 (HNO_2_ ejection), and ∼1% *m*/*z* 85.089 (NO_2_ ejection).

**Figure 12:**
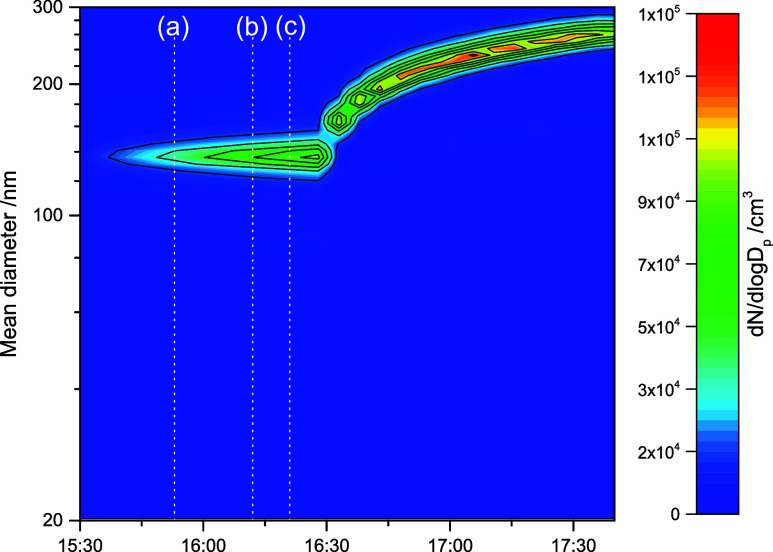
Particle number concentration and particle size distribution from SMPS measurements during the 2011.04.05 photolysis experiment in the EUPHORE atmospheric simulation chamber B. (a) Start injection of PIP-NO, (b) start injection of cyclohexane, and (c) open chamber canopy to solar irradiation.

It is obvious that the temporal imine signal at *m*/*z* 84.082 cannot possibly be due to reaction with OH radicals alone, but that particle formation must be involved. Figure 12 shows the particle number concentration and particle size distribution from scanning mobility particle sizer (SMPS) measurements during the experiment. Assuming that the particle formation is a result of PIP-IM salt formation with HNO_3_ (from NO_2_ + OH), the observed particle mass growth corresponds to a transfer of ∼30 ppb PIP-IM from the gas to the particle phase. It is therefore clear that the temporal gas-phase profile of PIP-IM should be interpreted with caution. The two other experiments were carried out under NO*x* conditions suppressing imine formation to the extent that the instrument signal of cyclohexane (*m*/*z* 84.094, C_6_H_12_^+^) was interfering with the PIP-IM signal (*m*/*z* 84.082) impeding its quantification. On the other hand, the particle formation and gas-phase mass transfer were much smaller in these experiments, as shown in Figure S12.

The photolysis experiments were modeled according to the generic reactions in Scheme 1, with the constraint *k*_4b_/*k*_4a_ < 0.01 from our theoretical study of the atmospheric PIPṄ radical reactions; see Section 3.1.2.1. The model takes NO, NO_2_, *j*_NO_2__, and the OH-field as input; the monitor values for NO and NO_2_ were cross-calibrated against results from FTIR.^17^ In addition to the photolysis and OH reactions, all compounds also experience wall loss and apparent loss due to air replenishment compensating for air drawn from the chamber by instruments and other sampling. The dilution loss rate coefficient, *k*_dilution_, was determined by monitoring the inert tracer, SF_6_, by FTIR. The first order wall loss of PIP-NO, *k*_wall_, was obtained from the apparent PIP-NO loss before opening of the chamber canopy; the photolysis model assumes that PIP-NO_2_ and PIP-IM face the same wall loss as PIP-NO.

The OH radical density was retrieved from the temporal profile of cyclohexanone formed in the cyclohexane + OH reaction (C_6_H_12_ + OH → C_6_H_11_ + H_2_O; C_6_H_11_ + O_2_ → C_6_H_11_OȮ; C_6_H_11_OȮ + NO → C_6_H_11_Ȯ + NO_2_; C_6_H_11_Ȯ + O_2_ → C_6_H_10_O + HO_2_) presuming the cyclohexane concentration and the rate coefficients for OH reaction with cyclohexane and cyclohexanone (6.7 and 6.4 × 10^–12^ cm^3^ molecule^–1^ s^–1^ at 298 K).^93^ The cyclohexane mixing ratio was quantified by FTIR and the cyclohexanone mixing ratio by PTR-TOF-MS; the derived OH radical density was found to be as high as 6 × 10^6^ cm^–3^ during the initial phase and continuously decreasing throughout the photolysis experiments, and OH radical reactions could potentially represent significant loss processes. However, the nitroso group has been found to reduce the OH reactivity of (CH_3_)_2_NNO by an order of magnitude^95,96^ vis-à-vis that of the parent amine,^93^ and the same is the case for the nitro group.^97^ Also imines are less reactive than their amine analogues.^98−100^ In accordance with the experimental evidence showing insignificant additional compounds being formed in the photolysis experiments (Figure 11), the OH reactivities of PIP-NO, PIP-NO_2_, and PIP-IM were constrained to be an order of magnitude smaller than that of piperidine itself (1.2 × 10^–10^ cm^3^ molecule^–1^ s^–1^, see Section 3.2.1).

**Figure 13:**
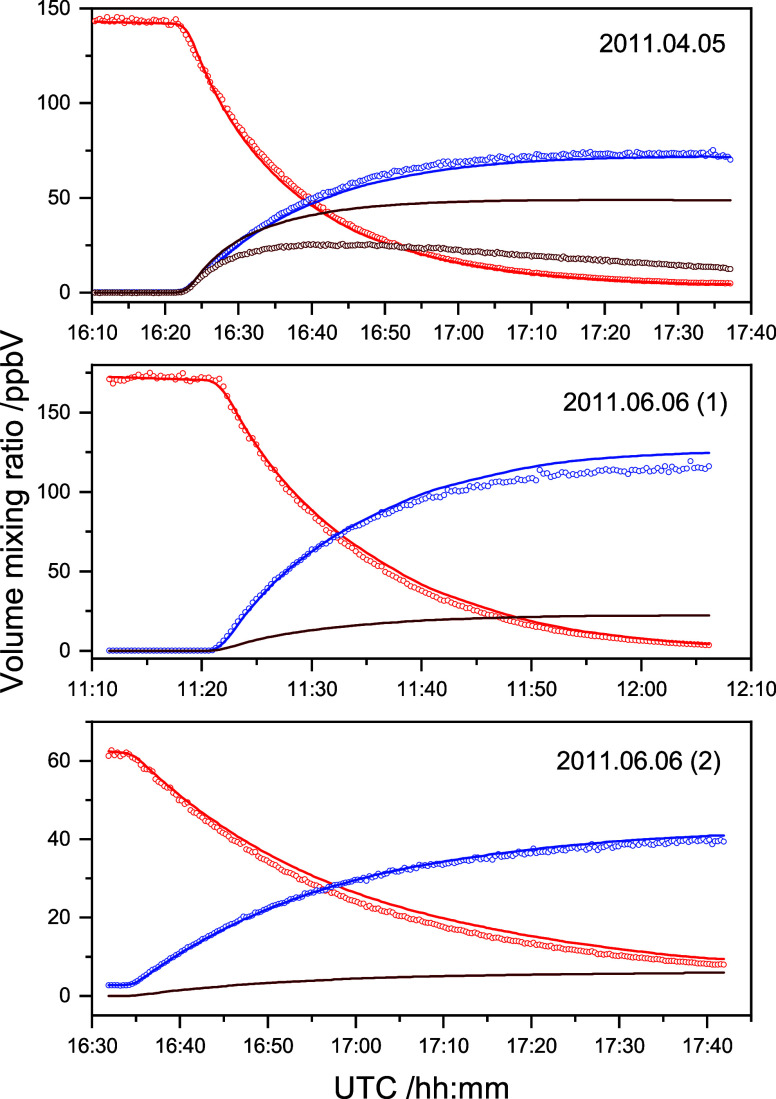
Observed and calculated mixing ratios of 1-nitroso piperidine, 1-nitropiperidine, and 2,3,4,5-tetrahydropyridine during the 1-nitrosopiperidine photolysis experiments on 2011.04.05 and 2011.06.06.

Figure 13 illustrates the quality of PIP-NO photolysis modeling under natural sunlight conditions. The agreement between experiment and model (r.m.s. 2.5 ppbV) is very pleasing considering the model constraints, and the inherent uncertainties in the monitor values for NO*x* and the actinic flux. The derived parameters − *j*_rel_ = 0.342 ± 0.007, *k*_3_/*k*_4a_ = 0.53 ± 0.05, and *k*_2_/*k*_4a_ = (7.66 ± 0.18) × 10^–8^ (3σ error limits) – are only slightly correlated (largest element [*j*_rel_, *k*_3_/*k*_4a_] = 0.88) and fall in the range reported from other nitrosamine photolysis studies.^20^ Disregarding the theoretical upper limit of *k*_4b_/*k*_4a_ < 0.01 and including *k*_4b_/*k*_4a_ in the nonlinear least-squares fitting procedure results in a slightly better fit with an r.m.s. = 1.491 ppbV and slightly different parameters: *j*_rel_ = 0.340 ± 0.004, *k*_3_/*k*_4a_ = 0.464 ± 0.030, *k*_4b_/*k*_4a_ = 0.119 ± 0.008 and *k*_2_/*k*_4a_ = (5.58 ± 0.15) × 10^–8^ (3σ error limits). The associated correlation matrix has two significant elements: [*j*_rel_, *k*_3_/*k*_4a_] = 0.87 and [*k*_4b_/*k*_4a_, *k*_3_/*k*_4a_] = −0.80. However, a value of *k*_4b_/*k*_4a_ = 0.119 is in utter disagreement with the theoretical results (Section 3.1.2.1) and must be dismissed as a fitting artifact.

Concerning OH-initiated oxidation during the experiments, the model simulations imply that up to 6% of the PIP-NO, PIP-NO_2_, and PIP-IM gas phase losses during the experiments were due to reaction with OH-radicals, cf. the additional weak ion signals observed in the experiments.

#### 3.2.3 Piperidine Photo-Oxidation Studies

Four PIP photo-oxidation experiments were carried out under sunny conditions in July 2016; IPN was slowly injected to the chamber during three of the experiments to boost the photo-oxidation relative to competing gas phase loss processes in the chamber. The experiments were carried out under diverse NO*x* conditions (Table S19) attempting to enhance the three PIPṄ radical reaction routes selectively; see Scheme 1.

Calibration experiments show that PIP fragments significantly at the instrument settings employed; at *E*/*N* = 105 Td, the fragmentation is 87% *m*/*z* 86.097 (C_5_H_12_N^+^, protonated molecule) and 13% *m*/*z* 84.082 (C_5_H_10_N^+^, H_2_-loss), the latter coinciding with the ion signal of protonated PIP-IM. At *E*/*N* = 65 Td, the fragmentation is less: 93% *m*/*z* 86.097 and 7% *m*/*z* 84.082. PIP-NO_2_ and PIP-NO both exhibit minor, but insignificant fragmentation in the present context; see Section 3.2.2.

**Figure 14:**
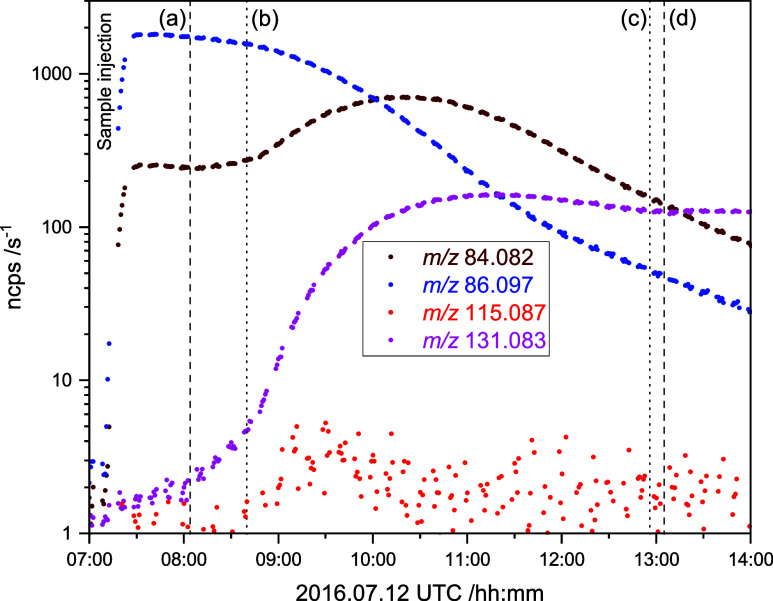
Normalized counts per second (ncps) for *m*/*z* 86.097 (protonated piperidine, PIPH^+^), *m*/*z* 84.082 (protonated 2,3,4,5-tetrahydropyridine, [PIP-IM]H^+^, as well as fragment of PIPH^+^), *m*/*z* 115.094 (protonated 1-nitrosopiperidine, [PIP-NO]H^+^), and *m*/*z* 131.083 (protonated 1-nitropiperidine, [PIP-NO_2_]H^+^) observed during the piperidine photo-oxidation experiment on 2016.07.12. (a) Chamber canopy opened to solar radiation. (b) IPN injection started. (c) IPN injection stopped. (d) Chamber canopy closed.

Figure 14 exemplifies the observed time evolution of the focal molecules ion signals recorded during a photo-oxidation experiment; the time profiles of other ion signals, increasing by more than 1% of the decrease in the PIP *m*/*z* 86.097 signal during the photo-oxidation experiments, are displayed in Figure S17. The temporal variation in the NO, NO_2_, and O_3_ mixing ratios and in *j*_NO_2__ are recorded in Figure S18. Note that *j*_NO_2__ is a factor of 2 larger during the photo-oxidation experiments than in the PIP-NO photolysis experiments (see above) and that the lifetime of PIP-NO in the photo-oxidation experiments is estimated to be less than 6 min. The mass peaks pertinent to the PIP photo-oxidation experiments are summarized in Table 1; a more complete list of observed ion signals including our interpretation is compiled in Table S20, from which one may note that the average difference between observed and exact *m*/*z* is < 0.002.

**Table 1:** Major PTR-TOF-MS Ion Signals Observed during OH Initiated Piperidine Photo-Oxidation Experiments^a^

exact *m*/*z*	ion sum formula	interpretation and comments
71.050	C_4_H_7_O^+^	unidentified product, also observed in PIPNO photolysis experiments
74.024	C_2_H_4_NO_2_^+^	unidentified product from autoxidation following H-abstraction from C^3^
84.081	C_5_H_10_N^+^	2,3,4,5-tetrahydropyridine (PIP-IM), [PIPH]^+^ fragment
86.097	C_5_H_12_N^+^	piperidine (PIP)
98.061	C_5_H_8_NO^+^	secondary product: 4,5-dihydropyridin-2(3*H*)-one
100.076	C_5_H_10_NO^+^	piperidin-4-one (PIPC^4^=O), CHOCH_2_CH_2_CH_2_*N* = CH_2_, 2,3,4,5-tetrahydropyridin-4-ol
114.092	C_6_H_12_NO^+^	piperidine-1-carbaldehyde, condensation product of PIP and HCOOH
115.087	C_5_H_11_N_2_O^+^	1-nitrosopiperidine (PIP-NO)
131.082	C_5_H_11_N_2_O_2_^+^	1-nitropiperidine (PIP-NO2)

a Only ion signals increasing by more than 2% of the *m*/*z* 86.097 ion signal decrease during the time of photo-oxidation are included. Signals due to isotopes and established chamber artifacts are not included.

Figure 14 establishes that PIP has a recognizable surface affinity, and that there was only a minor additional drop in the *m*/*z* 86.097 ion signal ascribable to reaction with OH radicals during the first 30 min after opening the chamber canopy to solar radiation. Upon the injection of IPN (0.1 μL min^–1^), the PIP gas phase loss increased and the ion signals associated with the primary products—PIP-IM (*m*/*z* 84.081) PIP-NO (*m*/*z* 115.087), and PIP-NO_2_ (*m*/*z* 131.082)—developed at a faster rate. It is also clear from the graph that PIP-IM is removed from the gas phase quite rapidly during the experiment.

The observed ion signals can be grouped according to their time evolution: (1) signals that grow initially and then decrease during the photo-oxidation experiments (reactive primary and secondary products, reactive compounds desorbing from chamber walls by displacement, and products with high affinity to the particle phase) and (2) signals that apparently grow steadily after opening the chamber canopy (secondary products, chamber artifacts and products released from the particle phase). Most of the ion signals observed fall into category 2; in fact, a meticulous inspection of the PTR-TOF-MS spectra shows relatively few ion signals increasing their intensity by more than 1% of the decrease in the PIP *m*/*z* 86.097 signal during the experiments. Further, the majority of these ion signals can either be attributed to established chamber artifacts, molecules from previous experiments desorbing from the chamber walls, or generic molecule ion fragments.

The temporal shape of the PIP-IM ion signal suggests that the imine reacts quite readily with OH, although not as fast as PIP. However, there are very few ion signals that can be attributed to secondary products. In fact, only *m*/*z* 98.061 (C_5_H_8_NO^+^) shows the correct temporal profile, and the ion sum formula suggests the incorporation of a carbonyl functionality into PIP-IM. Results from a recent theoretical study of the atmospheric oxidation of 1,2,3,6-tetrahydropyrazine (the imine of piperazine)^100^ indicates that the major reaction route in the PIP-IM + OH reaction should be H-abstraction from C^2^, and we tentatively assign the *m*/*z* 98.061 ion signal to protonated 4,5-dihydropyridin-2(3*H*)-one, 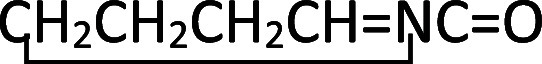.

One of the ions signals observed, *m*/*z* 114.092 (C_6_H_12_NO^+^), corresponds to a compound containing more carbon atoms than PIP, and it is attributed to piperidine-1-carbaldehyde resulting from condensation of PIP and formic acid, either in the particle phase or on the chamber walls. In 3 of the 4 experiments, the *m*/*z* 114.092 ion signal is an order of magnitude larger than the *m*/*z* 115.087 PIP-NO signal, and, the ^13^C isotopic signal of C_6_H_12_NO^+^, in particular, hampers the analysis and the quantification of PIP-NO.

Two, uncorrelated ion signals remain unexplained: *m*/*z* 71.050 and 74.024. The first was also observed in the PIP-NO photolysis experiments and could potentially stem from a photo-oxidation product of PIP-NO, PIP-NO_2_, or PIP-IM. The latter ion signal corresponds to a sum formula that has no obvious relationship PIP photo-oxidation and is tentatively attributed to a product in the autoxidation following H-abstraction from C^3^; see Section 3.1.2.3.

#### 3.2.4 Particle Analysis during the Piperidine + OH Reaction

**Figure 15:**
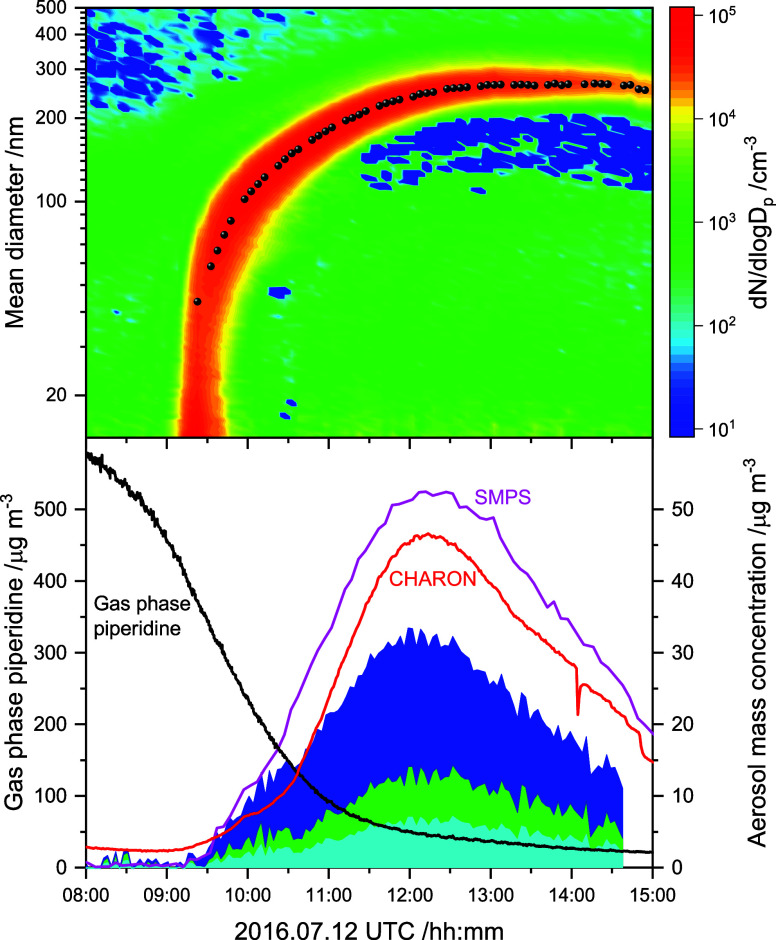
Top panel: Time evolution of particle size distribution during the piperidine photo-oxidation experiment on July 12, 2016. The chamber was opened to solar radiation 8:00; slow injection of IPN started at 8:40. Bottom panel: Time evolution of gas phase piperidine (black curve) and total particle mass loading from SMPS (magenta curve) and CHARON PTR-ToF-MS (red curve), and cumulative plots of the major chemical components from c-ToF-AMS: organic-water fragments (cyan), nitrate (green), and organic fragments (blue).

The piperidine photo-oxidation experiments were accompanied by minor particle formation, as exemplified in the top panel of Figure 15, displaying the temporal profile of the particle size distribution obtained by a scanning mobility particle sizer (SMPS). Particles were already present in the chamber before it was exposed sunlight at 8:00 UTC; these particles were formed in the PIP–HNO_3_ acid–base reaction (HNO_3_ initially being an impurity in the NO used and later resulting from the NO_2_ reaction with OH). As already mentioned, the OH radical precursor IPN was slowly injected to the chamber during the experiments to boost the photo-oxidation relative to competing gas phase loss processes in the chamber. At around 13:00 UTC, when the chamber was closed, the SMPS results showed a total particle mass loading of ∼50 μg m^–3^ and a particle number concentration of ∼7.0 × 10^3^ cm^–3^ having a mean mobility diameter of ∼260 nm. Assuming the particles to be pure PIP·HNO_3_ salt provides a rough estimate of around 5% of the initial gas phase PIP being transferred to the particle phase.

The bottom panel in Figure 15 shows the temporal profiles of gas phase piperidine, the total particle mass loading derived from SMPS, CHARON PTR-ToF-MS, and the particle composition (nitrate, organics, and water), as monitored online by C-ToF AMS. It is evident that AMS underestimates the organics fraction. This is related to the instrument relative ion efficiency, which was derived using pure piperidinium nitrate (there is a good agreement between the AMS and CHARON PTR-ToF-MS results for nitrate).

**Figure 16:**
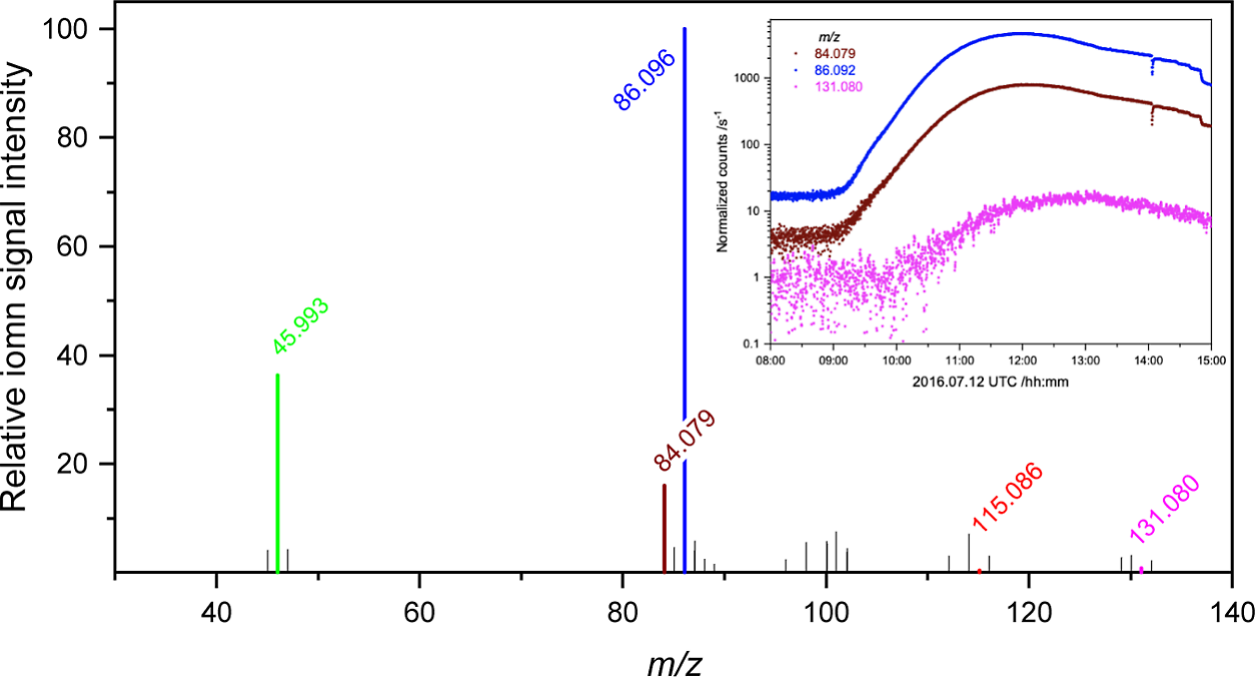
Particle mass spectrum measured by CHARON PTR-ToF-MS at 12:00 UTC on July 12, 2016: *m*/*z* 45.993 (NO_2_^+^), 84.079 (protonated PIP-IM), 86.096 (protonated PIP), 115.086 (protonated PIP-NO), and 131.080 (protonated PIP-NO_2_). Inset: time evolutions of *m*/*z* 84.079, *m*/*z* 86.092, and *m*/*z* 131.080. All ion signals are listed in Table S20.

Both AMS and CHARON PTR-ToF-MS data show that a considerable part of the total aerosol mass is due to piperidinium nitrate, but the data also clearly show that around half of the organic fraction of the particle mass is composed of organics other than PIP. A mass spectrum of the particle phase obtained by CHARON PTR-ToF-MS, as shown in Figure 16, clearly shows the dominating peaks being due to PIP (C_5_H_12_N^+^, *m*/*z* 86.092), nitrate (NO_2_^+^, *m*/*z* 45.993), and PIP-IM (C_5_H_10_N^+^, *m*/*z* 84.079), but around 20 other ion signals are observed with intensities above 2% of that of protonated PIP. Table S20 summarizes the ion signals observed together with our tentative interpretation. Considering the highly oxygenated products that eventually will be formed in the autoxidation upon ring-opening we refrain from offering molecular interpretation of all the ion signals observed.

PIP-NO_2_ was unambiguously detected in small amounts in the particle phase; see the inset in Figure 16. As described above in Section 3.2.3, quantification of PIP-NO by CHARON PTR-ToF-MS was hampered by interference from piperidine-1-carbaldehyde isotope signals. The results from filter sampling, collected in Tables S21 and S22, confirm both PIP-NO and PIP-NO_2_ in the particle phase.

#### 3.2.5 N–H/C–H Branching in the Piperidine + OH Reaction

The theoretical study predicts a branching between H-abstraction from the N^1^, C^2^, C^3^, and C^4^ positions in PIP to be 35(+19–8): 50(+11–20): 13(+4–5): 2(+1–1) at 298 K (Section 3.1.1). Scheme 3 shows that the initial branching *k*_NH_/*k*_tot_ in the PIP + OH reaction can be extracted from the temporal profiles of PIP and PIP-NO_2_ during the photo-oxidation experiments (as already mentioned, the PIP-NO gas phase profiles should be considered with caution).

The gas phase chemistry model puts the relative rate coefficients, obtained in the PIP-NO photolysis experiments (Section 3.2.2), to use, taking NO, NO_2_, and *j*_NO_2__ from the chamber monitors as input, as shown in Figure S18. The apparent dilution rate, due to air replenishment compensating for leakage and continuous instrument sampling, was obtained from FTIR spectra of SF_6_ added as an inert tracer. The PIP wall loss rate was, as previously mentioned, derived from its decay prior to adding IPN, and the wall losses of PIP-IM, PIP-NO, and PIP-NO_2_ were assumed to be the same as that of PIP. The model does not include transfer from gas to the particle phase as this is less than 5% of the total gas phase loss, as mentioned above. The OH concentration was extracted from the temporal PIP profiles using the experimental rate coefficient, *k*_PIP+OH_ = 1.2 × 10^–10^ cm^3^ molecule^–1^ s^–1^. There is a very good agreement between the temporal shape of the OH profiles measured directly by Fluorescence Assay by Gas Expansion (FAGE), as shown in Figure S19, and those derived from the decay of PIP in the early phase of the experiments, although there is an explicable difference of 50% between the absolute concentrations; see notes to Figure S19.^101,102^ In the later parts of the photo-oxidation experiments, where the OH concentration derived from the temporal PIP profiles is highly unreliable, scaled FAGE results, were used in the model.

**Figure 17:**
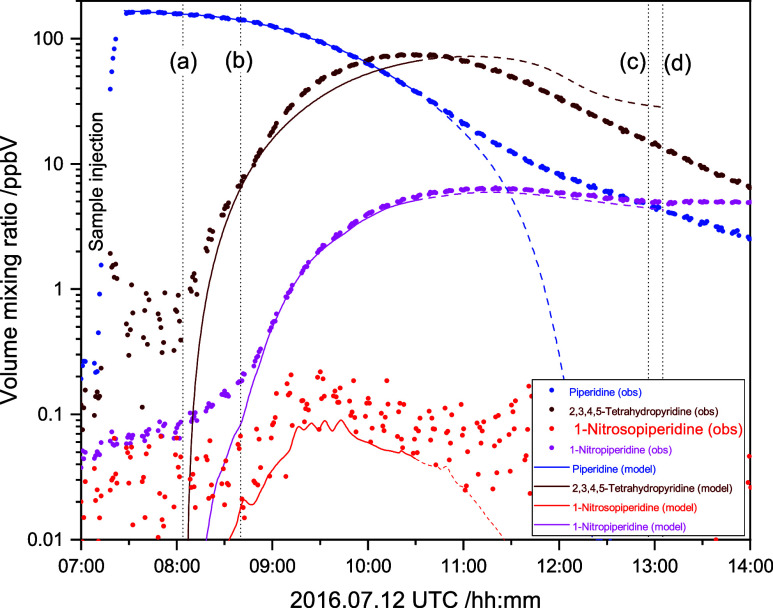
Observed and modeled volume mixing ratios of piperidine (PIP), 2,3,4,5-tetrahydropyridine (PIP-IM), and 1-nitropiperidine (PIP-NO_2_) observed during the piperidine photo-oxidation experiment on 2016.07.12. (a) Chamber canopy opened to solar radiation. (b) IPN injection started. (c) IPN injection stopped. (d) Chamber canopy closed.

Figure 17 illustrates the results from analysis of the PIP photo-oxidation experiment on 2016.07.12. According to the analysis, ∼130 of the ∼160 ppbV PIP present in the chamber at the start of the photo-oxidation was removed from the gas phase by reaction with OH. The PIP-NO_2_ profile can be reproduced reasonably well with *k*_N–H_/*k*_tot_ = 0.35 ± 0.05; to obtain a passable agreement between the experimental and modeled PIP-IM profile requires a slight increase in the C^2^ H-abstraction route to *k*_C^2^–H_/*k*_tot_ = 0.55 ± 0.05. Results from analyses of the other photo-oxidation experiments are visualized in Figure S20, which also includes the values of *k*_N–H_/*k*_tot_ and *k*_C^2^–H_/*k*_tot_ drawn from the experiments.

Figure 17 displays several eye-catching mis-matches. First and foremost, the PIP decay deviates significantly from the model results from around 11:00 and onward when the OH radical concentration is at its highest and when most of PIP has been removed from the gas phase. We attribute this mis-match to evaporation of particles in the heated sampling lines and, in particular, in the drift tube of the PTR-MS analyzer.^103^ A more pronounced example of particle evaporation in the PTR-MS analyzer was reported in AMP (2-amino-2-methyl-1-propanol) photo-oxidation experiments.^104^ Also, the (noisy) PIP-NO profile displays a time profile in the later part of the experiment, which conflicts the established gas phase chemistry of amines.^20^

Considering the inherent uncertainties in the monitor values for NO, NO_2_, and *j*_NO_2__, and in the derived volume mixing ratios from PTR measurements, the span in the extracted branching is remarkably modest, and the average results, *k*_N–H_/*k*_tot_ = 0.38 ± 0.08 and *k*_C^2^–H_/*k*_tot_ = 0.49 ± 0.19, are in disturbing agreement with the theoretical predictions abstaining us from drawing firm conclusions.

### 3.3 Synthesis of Experimental and Theoretical Results

The present quantum chemistry calculations narrow the branching in the initial H-abstraction reactions from PIP by OH down to 35(_–8_^+19^) % from N, 50(_–20_^+11^) % from C^2^, 13(_–5_^+4^) % from C^3^, and 2(_–1_^+1^) % from C^4^ at 298 K, which by providence agrees with the experimental results, 38 ± 8% from N and 49 ± 19% from C^2^. The quantum chemistry-based kinetic model for the PIP + OH reaction also forecasts a rate coefficient quite close to the experimental value, *k*_theo_(PIP + OH) = 0.948 and *k*_exp_(PIP + OH) = (1.19 ± 0.27) × 10^–10^ cm^3^ molecule^–1^ s^–1^ at 1014 ± 2 hPa and 304 ± 2 K. The sensitivity study of the theoretical PIP + OH kinetics results (Section 3.1.1) indicated that the calculated branching primarily depends on the saddle point energies and less on energies of the pre reaction complexes/adducts. Considering the calculated saddle point energies being associated with conservative uncertainties of ±4 kJ mol^–1^, it is therefore straightforward to reproduce the experimental rate coefficient by naively lowering all barriers by a merely ∼0.8 kJ mol^–1^. This leaves the calculated branching in the initial PIP + OH reaction essentially unchanged and in agreement with the experimental branching.

**Figure 18:**
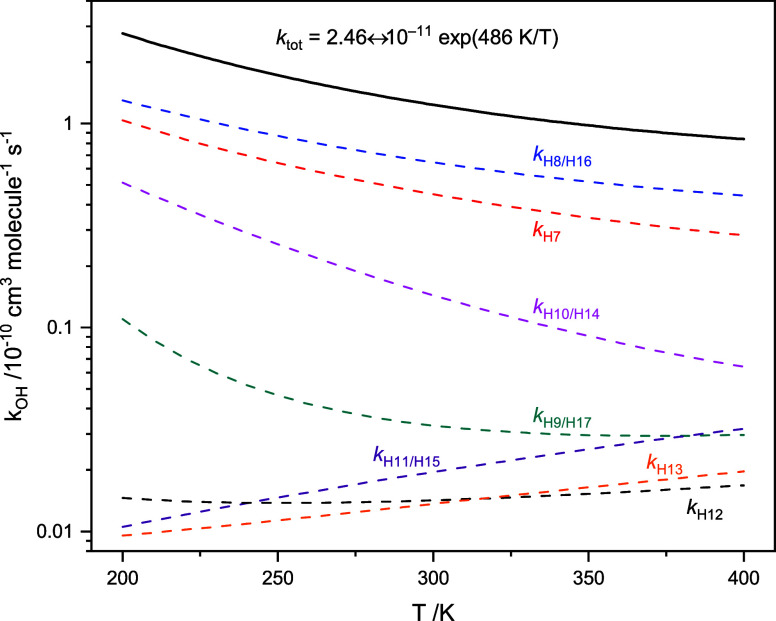
Theoretical rate coefficient *k*(T) for the piperidine + OH reaction at 1013 hPa aligned to reproduce the experimental rate coefficient and branching in the reaction at 304 K; see text. Contributions from the individual H-abstraction routes are shown as dashed curves.

Figure 18 shows the theoretical PIP + OH rate coefficient, scaled to match the experimental value at 304 K, as well as the individual contributions from the seven nonequivalent H-abstraction sites. The temperature dependence of the rate coefficient at 1013 hPa can be described quite accurately by the Arrhenius equation in the 200 to 400 K region: *k*(T) = 2.46 × 10^–11^ × exp(486 K/T) cm^3^ molecule^–1^ s^–1^.

The theoretical study of the PIPṄ + NO/NO_2_/O_2_ reactions (Section 3.1.2.1) concluded that multireference calculations are necessary to produce trustworthy ab initio values for the PIPṄ + O_2_ rate coefficient. The PIP-NO photolysis experiments (Section 3.2.2) provided a robust set of relative rate coefficients for the PIPṄ radical reactions with NO, NO_2_, and O_2_: *k*_3_/*k*_4a_ = *k*_PIPṄ+NO→PIP-NO_/*k*_PIPṄ+NO_2_→PIP-NO_2__ = 0.53 ± 0.05 and *k*_2_/*k*_4a_ = *k*_PIPṄ+O_2_→PIP-IM_/*k*_PIPṄ+NO_2_→PIP-NO_2__ = (7.66 ± 0.18) × 10^–8^.

Radical–radical reactions, such as PIPṄ + NO and PIPṄ + NO_2_, are expected to proceed with rate coefficients in the range 10^–11^ to 10^–10^ cm^3^ molecule^–1^ s^–1^ under atmospheric conditions; *k*_NH2+NO→products_ = 1.6 × 10^–11^ cm^3^ molecule^–1^ s^–1^ at 298 K (extensive review^87^) and *k*_NH_2_+NO_2_→products_ = 2.8 × 10^–11^ cm^3^ molecule^–1^ s^–1^ (high pressure value at 298 K^105^). The results from the PIP-NO photolysis experiments therefore imply that *k*_PIPṄ+O_2_→PIP-IM_ should fall in the region 8 × 10^–19^ to 8 × 10^–18^ cm^3^ molecule^–1^ s^–1^ at 298 K, which is consistent with the results from the multilevel correlation single-reference G3X-K calculations. The CC/M062X and UCC/M062X calculations results in rate coefficients being 1–2 orders of magnitude too large, whereas the results based on CBS-QB3 calculations are 2–3 orders of magnitude too small.

We note that Liu et al.^106^ examined the O_2_ reaction with 24 aminyl radicals, including the 1-piperidinyl radical, in CCSD(T)/6-311+G(2df,2p)//MP2/6-31+G(d,p) calculations and reported *k* = 1.4 × 10^–20^ cm^3^ molecule^–1^ s^–1^ at 298 K from canonical transition state theory including tunneling correction. This result is also deemed 2–3 orders of magnitude too small.

## 4 Discussion and Conclusions

The PIP + OH reaction rate coefficient was determined to be (1.19 ± 0.27) × 10^–10^ cm^3^ molecule^–1^ s^–1^ at 1014 ± 2 hPa and 304 ± 2 K, which is roughly half of sum of *k*_OH_ for piperazine^81,107^ and cyclohexane.^93^ The branching in the PIP + OH reaction was determined to be *k*_N–H_/*k*_tot_ = 0.38 ± 0.08, which apparently does not compare well with the experimental values offered for the branching in the piperazine + OH reaction: 0.09 ± 0.06,^107^ and 0.18 ± 0.04.^81^ According to the present theoretical study, the branching in the OH reaction is quite different for the two PIP conformations (*k*_N–H_/*k*_tot_ = 0.32 for eq and 0.58 for ax), and piperazine exists in three low-energy chair-conformations that are distributed 55% eq–eq, 42% eq–ax, and 3% ax–ax at 298 K;^81^ unfortunately, the only existing theoretical analysis of the kinetics and branching in the piperazine + OH reaction just considers the eq–eq conformer.^108^ Branching data exist for two other secondary amines: (CH_3_)_2_NH (*k*_N–H_/*k*_tot_ = 0.37 ± 0.05,^89^ 0.42 ± 0.05,^109^ and 0.41 ± 0.07^110^) and (CH_3_CH_2_)_2_NH (*k*_N–H_/*k*_tot_ = 0.60 ± 0.10).^17^

To the best of our knowledge, there are no estimations of the magnitude of PIP emissions to the atmosphere. Once in the atmosphere, PIP will partition between the gas phase and the solid/deliquescent particle phase. There are no kinetic transfer parameters available for piperidine, but assuming the experimental uptake coefficients for methylamines on 59–82 wt % sulfuric acid (γ ∼ 2 × 10^–2^)^111^ establishes amine uptake on deliquescent particles in general, the implication is that the aqueous particle uptake of PIP will be diffusion controlled under atmospheric conditions.

With *k*_PIP+OH_ ≈ 1.2 × 10^–10^ cm^3^ molecule^–1^ s^–1^, the atmospheric lifetime of PIP with respect to gas phase reaction with OH during daytime will typically be 2–3 h. The night-time chemistry of PIP is expected to be dominated by the NO_3_ radical. There is no experimental value for *k*_PIP+NO_3__, but the empirical correlation between OH and NO_3_ rate coefficients for reaction with amines implies a very fast reaction, *k*_PIP+NO_3__ ≈ 5 × 10^–12^ cm^3^ molecule^–1^ s^–1^ at 298 K.^20^ The average night-time NO_3_ concentration has been suggested to be around 5 × 10^8^ cm^–3^,^112,113^ bringing the estimated lifetime of PIP during night to less than 10 min. Note that there is no information available in the literature on the branching between N–H and C–H H-abstraction in amines by the NO_3_ radical.

The experimental Henry’s law solubility constant for PIP is *H*^cp^ = 2.8 mol m^–3^ Pa^–1^ at 298 K.^16^ Under nonreactive equilibrium conditions and assuming the liquid water content in clouds, fog, and urban aerosol to be, respectively, 3, 0.2 and 10^–4^ cm^3^ m^–3^,^114^ PIP will partition roughly 2, 0.14, and ≪0.01% to the aqueous particle phase in the three cases. There are no experimental data for the aqueous phase PIP reactivity, but *k*_OH,aq_ = 8.24 × 10^9^ M^–1^ s^–1^ is predicted by the group contribution method of Minakata et al.^115^ Taking typical average values for the OH concentration in maritime, remote and urban clouds (2 × 10^–12^, 2.2 × 10^–14^, 3.5 × 10^–15^ M^–1^) and in deliquescent particles (1 × 10^–13^, 3 × 10^–12^, 4.4 × 10^–13^ M^–1^),^116^ the estimated lifetime of PIP in clouds will be around 1 min in maritime and 2–10 h in remote and urban environments. The lifetime of PIP in deliquescent particles is predicted to be short, ∼20 min in maritime, but only 1–5 min in remote and urban environments, and the high reactivity in maritime clouds and in deliquescent aerosol will consequently drive additional uptake. Note that there are no experimental results from mechanistic studies of aqueous phase piperidine reactions.

The major product in the atmospheric degradation of PIP, PIP-IM, is expected to react an order of magnitude slower with OH than PIP, and PIP-IM is therefore to a large extent likely to undergo hydrolysis in aqueous particles, resulting in ring opening and the formation of CHOCH_2_CH_2_CH_2_CH_2_NH_2_ (5-aminopentanal).

Regarding the photo-oxidation products of health concern, PIP-NO and PIP-NO_2_, the former will primarily undergo very fast photolysis in the gas phase, *j*_PIP-NO_ ≈ 0.35·*j*_NO_2__. Taking the annual average *j*_NO_2__ at 60°N (1.3 × 10^–3^ s^–1^)^117^ leads to an annual average gas phase photolysis lifetime of <40 min. Both PIP-NO and PIP-NO_2_ are expected to react an order of magnitude slower with OH than PIP does; their atmospheric lifetimes with respect to gas phase reaction with OH are consequently estimated to be 1–2 days.

The Henry’s law solubility constant of PIP-NO is ∼4 times larger than that of PIP.^118^ There are no data for the Henry’s law solubility constants for nitramines, but to a first approximation, they are expected to be the same as those of the nitrosamines. Phase transfer will therefore be important for these compounds, and with aqueous phase OH rate coefficients of 2.98 × 10^9^ and 2.8 × 10^9^ M^–1^ s^–1^, for PIP-NO^119^ and PIP-NO_2_,^120^ respectively, their estimated lifetimes with respect to reaction with OH radicals in deliquescent particles will be nearly as short as that of PIP, driving additional phase transfer.

The aqueous phase photolysis speed of PIP-NO has been shown to be comparable to that of the gas phase,^121^ implying that the lifetime of PIP-NO in droplets and deliquescent particles will also be comparable that of the gas phase.

The present results permit implementation of a detailed gas phase degradation mechanism accounting for more than 85% of PIP in emission dispersion modeling; the remaining 15% is expected to undergo autoxidation resulting in yet unknown compounds. A simple box model (parameters given in Table S23(89,93,95,96,110)), based on average atmospheric NO*x* conditions in the Oslo region (⟨*j*_NO_2__⟩_annual_ = 1.3 × 10^–3^ s^–1^,^117^ ⟨NO⟩_annual_ ∼ 6 ppb and ⟨NO_2_⟩_annual_ ∼ 10 ppb^122^), demonstrates similarities and differences in the formation of nitrosamines and nitramines, resulting in dimethylamine and PIP emissions from a point source. The results show that 1 h after emission, the mixing ratios of PIP-NO and PIP-NO_2_ will be around 10, respectively 5 times larger than those of nitrosodimethylamine (NDMA) and dimethylnitramine (DMNM) per unit of amine emitted, respectively, as shown in Figure S21. Although the actinic flux and NO*x*-conditions in London, Seoul, and Urumqi differ substantially from those characteristic of Oslo, it is obvious that the major part of nitrosamines reported in the atmospheric particulate matter in central London,^8^ Seoul,^9,10^ and in Urumqi,^11^ cannot originate in gas phase photo-oxidation of the corresponding amines.

## Supporting Information Available

Details on instrumentation and experimental methodologies, including chemical synthesis, documentation of quantum chemistry calculations, and experimental conditions and results (PDF)
