# Supplementary material for: Experimental and Theoretical Study of the OH-Initiated Degradation of Piperidine under Simulated Atmospheric Conditions
Source: J Phys Chem A. 2024 Mar 29;128(14):2789–814. doi: 10.1021/acs.jpca.3c08415 (PMC11017256; doi:10.1021/acs.jpca.3c08415)
Supplement: Supplementary file 1 — jp3c08415_si_001.pdf [file jp3c08415_si_001.pdf]

Experimental and Theoretical Study of the OH-Initiated Degradation of Piperidine under Simulated Atmospheric Conditions

Wen Tan,[1] Liang Zhu,[1§] Tomas Mikoviny,[1] Claus J. Nielsen,[1*] Armin Wisthaler,[1] Barbara D´Anna,[2] Simen Antonsen,[3] Yngve Stenstrøm,[3] Naomi J. Farren,[4] Jacqueline F. Hamilton,[4] Graham A. Boustead,[5] Trevor Ingham[5] and Dwayne E. Heard[5]

[1] Section for Environmental Sciences, Department of Chemistry, University of Oslo, P.O.Box. 1033 Blindern, NO-0315 Oslo, Norway. [2] Aix Marseille Université, CNRS, LCE, UMR 7376, 13331, Marseille, France. [3] Faculty of Chemistry, Biotechnology and Food Science, Norwegian University of Life Sciences, P.O. Box 5003, N-1432 Ås, Norway. [4] Wolfson Atmospheric Chemistry Laboratories, Department of Chemistry, University of York, York, YO10 5DD, United Kingdom. [5] School of Chemistry, University of Leeds, Leeds, LS2 9JT, United Kingdom.

Supporting Information

EXPERIMENTAL METHODS AND CHEMICALS 3
European Photoreactor (EUPHORE). 3
Proton-Transfer-Reaction Time-of-Flight Mass Spectrometry (PTR-ToF-MS). 3
Chemical Analysis of Aerosol Online (CHARON) 3
Aerosol Mass Spectrometry 4
Aerosol filter sample collection, extraction and analysis 4
Fluorescence Assay by Gas Expansion (FAGE) 6
Chemicals 7
Safety precautions in the syntheses of 1-nitrosopiperidine and 1-nitropiperidine 7
1-nitrosopiperidine 7
1-nitropiperidine 7

COMPUTATIONAL RESULTS 8
Table S1. Dipole moments, isotropic polarizabilities and PTR rate coefficients. 8
Table S2. QCC results for piperidine conformational pathways 8
Kinetics and branching in the piperidine + OH reaction 15
Table S3. QCC results for the piperidine (eq) reaction with OH radicals 15
Table S4. QCC results for the piperidine (ax) reaction with OH radicals 30
Figure S1. Rotation potential energies of the CH/NH-abstraction saddle points in the piperidine + OH reaction. 48
Figure S2. Structures of pre-reaction complexes/adducts and saddle points in the piperidine + OH reaction 54
Table S5. Descriptors of saddle points in the piperidine + OH reaction. 55
Figure S3. Calculated rate coefficients (200 – 400 K) for the piperidine + OH reaction 56
Table S6. Site-specific rate coefficients for the OH reaction with piperidine 57
Atmospheric fate of the 1-piperidinyl radical 60
Table S7. QCC results for the eq-ax equilibria in the piperidine C^3 and C^4 radicals 60
Figure S4. Spin contamination in the PIPNOȮ$_{eq}$ dissociation reaction 62
Figure S5. Spin contamination in the PIPNOȮ $\rightarrow$ PIP-IM + HOO reaction 63
Table S8. QCC results for the 1-piperidinyl radical reactions with O_2 64
Figure S6. CNOO torsional potentials in PIPNOO. 70
Figure S7. Calculated k(T) for the PIPṄ + O_2 $\rightarrow$ PIP-Im + HO_2 reaction 71
Table S9. QCC results for the 1-piperidinyl radical reactions with NO and NO_2 71
Table S10. QCC results for the NH_2 and CH_3NH radical reactions with O_3 75
Atmospheric fate of the 2-piperidinyl radical 76
Table S11. QCC results for the PIP-C^2OȮ conformational pathways and internal H-transfer reactions 76
Atmospheric fate of the 3-piperidinyl radical 93
Table S12. QCC results for the PIPC^{3}OȮ conformational pathways and internal H-transfer reactions 93
Table S13. QCC results the PIP-C^3Ȯ conformational pathways and reactions 107
Figure S8. PES of the $CHOCH_2CH_2CH_2NH\dot{C}H_2$ + O_2 reaction. 121
Table S14. QCC results for the $CHOCH_2CH_2CH_2NHCH_2O\dot{O}$ internal H-transfer reactions 121
Figure S9. PES of the $C(O)CH_2CH_2CH_2NHCH_2OOH$ + O_2 reaction 127
Table S15. QCC results for the $\dot{O}OC(O)CH_2CH_2CH_2NHCH_2OOH$ internal H-transfer reactions 128

ATMOSPHERIC FATE OF THE 4-PIPERIDINYL RADICAL 139
Figure S10. PES of the PIPĊ⁴ + O_2 reaction. *140*
Figure S11. PES of PIPC⁴Ȯ reactions. *142*
Table S16. QCC results for the PIP-C⁴OȮ conformational pathways and internal H-transfer reactions *143*
Table S17. QCC results for the PIP-C⁴Ȯ conformational pathways and internal H-transfer reactions *152*

EXPERIMENTAL RESULTS 162

PIPERIDINE + OH REACTION KINETICS 162
Figure S12. Piperidine + OH relative rate plot, 2016.07.19 experiment 2. .... *162*
Figure S13. Piperidine + OH relative rate plot, 2011.06.08. .... *163*
Table S18. Summary of relative rate results for the piperidine + OH reaction. *163*
1-NITROSOPIPERIDINE PHOTOLYSIS STUDIES 164
Figure S14. SMPS results 2011.06.06. .... *167*
Figure S15. p, T, j_{NO2}, NO, NO_2 and O_3 during the PIP-NO photolysis experiment on 2011.04.05. .... *165*
Figure S16. p, T, j_{NO2}, NO, NO_2 and O_3 during the PIP-NO photolysis experiment on2011.04.05 .... *166*
PIPERIDINE PHOTO-OXIDATION STUDIES 168
Table S19. Initial experimental conditions in piperidine photo-oxidation experiments. *168*
Figure S17. Small ion signals observed by PTR-TOF-MS during the experiment on 2016.07.12. .... *169*
Table S20. Ion signals observed by PTR-TOF-MS during the experiment on 2016.07.12. .... *170*
Figure S18. j_{NO2}, NO, NO_2 and O_3 mixing ratios during the 2016.07.12 photo-oxidation experiment. *171*
Figure S19. OH and HO_2 concentrations during the 2016.07.12 photo-oxidation experiment. *172*
PARTICLE ANALYSIS DURING THE PIPERIDINE + OH REACTION 173
Table S21. Results from filter sampling *173*
Table S22. Results from filter sampling *174*
N-H/C-H BRANCHING IN THE PIPERIDINE + OH REACTION 175
Figure S20. Observed and modelled PIP, PIP-IM, PIP-NO and PIP-NO_2 during photo-oxidation experiments. *176*
SYNTHESIS OF EXPERIMENTAL AND THEORETICAL RESULTS 176

DISCUSSION & CONCLUSIONS 176

Table S23. Parameters for box-modelling of nitrosamine and nitramine formation *176*
Figure S21. Box model results for nitrosamine and nitramine formation from $(CH_3)_2NH$ and PIP emissions *177*

Experimental Methods and Chemicals

European Photoreactor (EUPHORE).

A series of experiments was carried out in chamber B of the EUPHORE facility in Valencia, Spain. The 200 m^3 polytetrafluoroethylene (PTFE) atmosphere simulation chamber has been described in detail elsewhere[22] and only the details pertinent to this work are given here. A syringe pump was used to inject known amounts of piperidine (PIP) into the chamber via a heated (120 °C) transfer line made of passivated stainless steel. The line was flushed with nitrogen during and after injection. Nitric oxide (NO) was injected using a gas-tight syringe. A basic scrubber was inserted into the injection line for removing trace nitric acid. Nitrogen dioxide (NO_2) was generated from NO via addition of ozone (O_3). After ~1 hour of chamber conditioning the chamber canopy was opened to sunlight radiation. In some experiments isopropyl nitrite (IPN) was continuously added to the chamber in a flow of nitrogen as an efficient OH radical precursor. Acetonitrile was used as a virtually inert dilution tracer in the kinetic study. The EUPHORE facility is equipped with standard monitors for pressure (p), temperature (T), relative humidity, NO, NO_2, O_3, NO_2 photolysis frequency (j_{NO2}) and sub-micrometer particle size distribution. The latter was measured using a Scanning Mobility Particle Sizer (SMPS) consisting of an electrostatic classifier (Model 3080, impactor: 0.0457 cm) with a long differential mobility analyzer (DMA, Model 3081), and a condensation particle counter (CPC, Model 3022A). For the experiments described herein, we deployed a series of additional analyzers/analytical methods, which are described in more detail below.

(22) K. H. Becker, *The European Photoreactor EUPHORE: Design and Technical Development of the European Photoreactor and First Experimental Results: Final Report of the EC-Project: Contract EV5V-CT92-0059: Funding Period, January 1993-December 1995*, 1996.

Proton-Transfer-Reaction Time-of-Flight Mass Spectrometry (PTR-ToF-MS).

A PTR-TOF 8000 instrument (Ionicon Analytik GmbH, Innsbruck, Austria) was used for measuring PIP and its photochemical oxidation products in the gas phase. The instrument has been described in detail elsewhere[23] and thus only the details pertinent to this study are described here. The drift tube was kept at a temperature of 100 °C and a pressure of 2.30 mbar. The electric field applied to the drift tube was periodically switched in 80 s intervals, i.e. measurements were performed at alternating E/N-values of 65 and 105 Td (1 Td = 10^{-17} V cm^{-2} $molecule^{-1}$), respectively. The PTR-TOF 8000 instrument was interfaced to the chamber using Siltek®/Sulfinert®-treated stainless steel tubing (total length: 143 cm, 60 cm extending into the chamber, ID: 4.57 mm, temperature: 100 °C, flow: 20 lpm). The flow to the instrument was subsampled through PEEK® (polyetheretherketone) capillary tubing (OD: 1.59 mm). The instrument was regularly calibrated against a gaseous reference standard containing 13 hydrocarbons and oxygenated hydrocarbons. The instrument was further calibrated for piperidine, 1-nitrosopiperidine (PIP-NO) and 1-nitro-piperidine (PIP-NO_2) using a commercial liquid calibration unit (LCU; Ionicon Analytik GmbH, Innsbruck, Austria) for evaporation of gravimetrically prepared aqueous standards in nitrogen. The fragmentation patterns of PIP, PIP-NO and PIP-NO_2 were taken from the calibration measurements. For other analytes reported herein, we used theoretically derived instrumental response factors summarized in Table S1.

(23) A. Jordan, S. Haidacher, G. Hanel, E. Hartungen, L. Märk, H. Seehauser, R. Schottkowsky, P. Sulzer and T. D. Märk, A high resolution and high sensitivity proton-transfer-reaction time-of-flight mass spectrometer (PTR-TOF-MS). *Int. J. Mass Spectrom.*, 2009, **286**, 122-128. DOI: 10.1016/j.ijms.2009.07.005

Chemical Analysis of Aerosol Online (CHARON)

A prototype CHARON inlet[24,25] was interfaced to a second PTR-TOF 8000 instrument (Ionicon Analytik GmbH, Innsbruck, Austria) for measuring particle chemical composition. The CHARON inlet strips off gas-phase analytes, enriches the particle concentration in the PTR-ToF-MS subsampling flow and vaporizes the particles prior to ionization and mass spectrometric analysis. In this study, the vaporization temperature was set to 140 °C. The CHARON inlet was interfaced to the EUPHORE chamber using Siltek®/Sulfinert®-treated stainless steel tubing (total length: 415 cm, 40 cm extending into the chamber, ID: 4.57 mm). The PTR-ToF-MS drift tube was kept at a voltage of 350 V, a temperature of 130 °C and a pressure of 2.40 mbar (100 Td). The instrument was regularly calibrated against a gaseous reference standard containing 13 pure and oxygenated hydrocarbons. A reference mass spectrum was obtained from the PZ-aminium nitrate salt.

(24) P. Eichler, M. Muller, B. D'Anna and A. Wisthaler, A Novel Inlet System for Online Chemical Analysis of Semi-Volatile Submicron Particulate Matter. *Atmos. Meas. Tech.*, 2015, **8**, 1353-1360. DOI: 10.5194/amt-8-1353-2015
(25) P. Eichler, M. Müller, C. Rohmann, B. Stengel, J. r. Orasche, R. Zimmermann and A. Wisthaler, A Novel Inlet System for Online Chemical Analysis of Semi-Volatile Submicron Particulate Matter. *Environ. Sci. Technol. Lett.*, 2017, **4**, 54-58. DOI: 10.1021/acs.estlett.6b00488

Aerosol Mass Spectrometry

A compact time-of-flight Aerosol Mass Spectrometer (C-ToF-AMS, Aerodyne Research Inc., Billerica, MA, U.S.A.)[26] was used for measuring total particle mass loading and particle chemical composition. A reference spectrum was obtained from the piperidinium nitrate salt. The cToF-AMS data set was processed using a cumulative peak fitting analysis which allows to separate multiple isobaric peaks that are not taken into account in the traditional analysis of unit mass resolution data. [27] Uncertainties in the major chemical species from the cToF-AMS are typically of the order of ±30%. [27]

(26) F. Drewnick, S. S. Hings, P. DeCarlo, J. T. Jayne, M. Gonin, K. Fuhrer, S. Weimer, J. L. Jimenez, K. L. Demerjian, S. Borrmann and D. R. Worsnop, *Aerosol Sci. Technol.*, 2005, **39**, 637-658. DOI: 10.1021/acs.estlett.6b00488
(27) M. Müller, C. George and B. D'Anna, Enhanced spectral analysis of C-TOF Aerosol Mass Spectrometer data: Iterative residual analysis and cumulative peak fitting. *Int. J. Mass Spectrom.*, 2011, **306**, 1-8. DOI: 10.1016/j.ijms.2011.04.007

Aerosol filter sample collection, extraction and analysis

Filters were collected at appropriate intervals throughout the photo-oxidation experiments, in order to obtain time-resolved measurements of aerosol formation and composition.

When the chamber canopy was open the total flow rate available for aerosol filter sampling was 10 L min^{-1}; there were several instruments operating and so flow rates were restricted to avoid excessive chamber dilution. However, when the chamber canopy was closed there were fewer instruments operating and so higher flow rates were allowed for the aerosol sampling. As a result, aerosol sampling flow rates varied throughout the experiment as detailed in below, allowing for the best compromise between aerosol mass, time resolution and chamber dilution to be achieved.

Aerosol filter sampling during the July 2016 photo-oxidation experiments.

Stage of experiment	Sampling flow rate (L min^{-1})	Sampling time (min)
Chamber background – canopy closed, no reagents added	*ca.* 48	30
Photo oxidation – canopy open, all reagents added	10 (2 × 5)	30 - 120
Remaining aerosol – chamber closed, end of experiment	*ca.* 48	60

For the samples collected using the high-volume sampling pump (*ca*. 48 L min^{-1}), the inlet to the 47 mm filter holder (Cole-Parmer, London, U.K.) was connected to the chamber using a 40 cm length of ¼” PFA tubing (Swagelok, Teesside, U.K.) with an in-line ¼” stainless steel tap installed 10 cm above the filter holder inlet. A further 15 cm of ¼” of PFA tubing was used to connect the filter holder outlet to a mass flow meter (0-50 L min^{-1}, Alicat, Cambridge, U.K.). The mass flow meter outlet was connected to the high volume pump *via* 1.5 m of ½” PFA tubing. A diagram of the high-volume filter sampling set up is shown in Figure 4.3. The low-volume filter sampling was carried out at 10 L min^{-1}, and the air flow was split equally between two 47 mm filter holders, as shown below. The connections between the chamber, filter holders and mass flow controllers were made using ¼” PFA tubing. After collection, all of the filter samples were stored at -18 °C until analysis. The high-volume filters were divided into two parts; ¼ for analysis by IC and ¾ for analysis by GC×GC. One filter from each pair of low-volume filters was used for analysis by IC and the other one was used for analysis by GC×GC.

Diagrams showing the filter sampling set up for the collection of both the high-volume samples (left) and the low-volume samples (right).

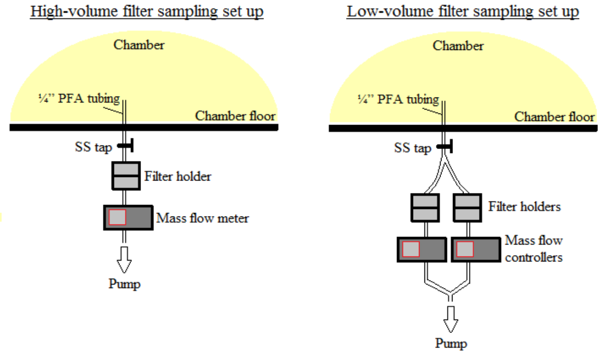

*Preparation for GC×GC-NCD analysis***:** each sample was extracted using an accelerated solvent extraction system (ASE 350, Dionex, CA, USA). The base of each 5 mL stainless steel extraction cell was lined with two glass microfibre filter papers (Fisher Scientific, Loughborough, U.K.) and then packed with the sample. Extractions were carried out in EtOAc (HPLC grade, >99.5% purity) at 80 °C and 1500 psi for three consecutive 5 min cycles. A 50% flush volume and 60 s purge time were used. Extracts obtained were kept in an ice bath whilst they were evaporated under nitrogen to a final volume of 1 mL.

Preparation for IC analysis: each sample was dissolved in 2 mL ultrapure milli-Q water (18 MΩ cm^{-1}) and sonicated at RT for 30 mins. The extract was filtered using a Millex-GP 33 mm diameter hydrophilic syringe filter with a pore size of 0.22 µm (Millipore UK Limited, Watford, U.K.).

The amount of filter sample used for the extraction depended on whether a high- or low-volume sample was being extracted, as outlined below.

Summary of the extraction method and analytical technique used for each type of sample collected at EUPHORE.

Sample type	Analytical technique	Extraction method	Filter paper size
High volume	GCxGC-NCD	Accelerated solvent extraction	¾ filter (47 mm diameter)
High volume	IC	Aqueous sonication	¼ filter (47 mm diameter)
Low volume	GCxGC-NCD	Accelerated solvent extraction	Whole filter (47 mm diameter)
Low volume	IC	Aqueous sonication	Whole filter (47 mm diameter)

Chromatographic analysis – GCxGC-NCD: Chromatographic analysis was carried out on a GC×GC-NCD system comprised of an Agilent 7890 gas chromatograph and an Agilent 255 NCD system (Palo Alto, CA, United States). The first column was a non-polar Ultra Inert DB5 (30 m × 0.32 mm i.d. × 0.25 µm film thickness) and the second column a mid-polarity DB-17 (3 m × 0.10 m i.d. × 0.10 µm film thickness). Both columns were purchased from Agilent Technologies Ltd., Stockport, U.K. The initial temperature of the first dimension column was 40 °C for 2 min, followed by a heating rate of 7 °C min^{-1} to 100 °C for 8 min and then further heating at 7 °C min^{-1} until 270 °C was reached and held isothermally for a further 5 min. A temperature offset of 30 °C was applied to the second dimension column throughout the GC temperature program. A liquid nitrogen two-stage cold jet modulation system was used, with a modulation period of 5 s and a +15 °C offset from the secondary GC oven temperature. Data was collected at 200 Hz over the entire course of the analysis, and hydrogen was used as a carrier gas at 1.4 mL min^{-1}. Injections of 1 µL were performed in splitless mode at an injection temperature of 200 °C using an automated liquid injector (Gerstel, Mülheim an der Ruhr, Germany). Pyrolysis of the analytes in the dual plasma burner was carried out at 900 °C under a hydrogen flow rate of 4 mL min^{-1} and an oxygen flow rate of 10 mL min^{-1}.

Chromatographic analysis – IC: Chromatographic analysis was carried out using an ICS-110 integrated IC system equipped with an AS-DV autosampler (Dionex Corporation, CA, United States). The column configuration used for anion exchange consisted of an IonPac AG14A guard column (4 × 50 mm) and an IonPac AS14A analytical column (4 × 250 mm). Cation exchange chromatography was performed using an IonPac CG12A guard column (4 × 50 mm) and an IonPac CS12A analytical column (4 × 250 mm). AERS 500 and CSRS 300 self-regenerating suppressors (4 mm) were used for anion and cation exchange respectively. All columns and suppressors were supplied from Dionex Corporation. The system used a DS6 heated conductivity cell for ion detection. All anion-exchange experiments were carried out in isocratic mode but some cation-exchange experiments relied on gradient elution. Gradient elution was achieved using a Dionex reagent free controller (RFC-30) in combination with a Dionex EGC 111 MSA eluent generator cartridge and a continuously regenerated trap column. A summary of the separation methods for anion and cation chromatography is provided below

Method parameters used for both anion exchange and cation exchange chromatography.

method parameter	anion exchange	cation exchange	
run time	18 min	15 min	24-27 min
flow type	isocratic	isocratic	gradient
flow rate	1 mL/min	1 mL/min	1 mL/min
injection volume	100 μL	100 μL	100 μL
data collection rate	5 Hz	5 Hz	5 Hz
column temperature	30 °C	30 °C	30 °C
suppressor current	45 mA	59 mA	59 – 293 mA

Additional details of the gradient elution system can be found below. All data obtained was analysed using the Chromeleon v7.1 software package.

Gradient methods employed for the analysis of $[C_5H_{12}N]^+$ and $[C_4H_{12}N_2]^{2+}$.

Gradient method for protonated piperidine, $[C_5H_{12}N]^+$		
Rate / mM min^{-1}	Target concentration / mM	Duration / min
Initial	20	10
4.00	60	10
Final	20	4

Fluorescence Assay by Gas Expansion (FAGE)

A FAGE (Fluorescence Assay by Gas Expansion) apparatus was used for the measurement of OH radicals during the 2016 ACA campaign. FAGE is a method based on laser-induced fluorescence (LIF) spectroscopy at low pressure, and has been widely used for the measurement of OH concentrations both in the field [28] and in the laboratory. The OH radical is excited with a wavelength-tunable pulsed laser at 308 nm (via the $A^2\Sigma$ v'=0 ← $X^2\ \Pi_i$ v''=0 electronic transition), after which on-resonance fluorescence around 308 nm is detected.[29] FAGE uses a low pressure fluorescence detection cell (pressure held at ~2 Torr or less) to extend the fluorescence lifetime of OH to beyond that of the excitation laser pulse (~10 ns), and gated photon counting is then used to separate the small fluorescence signal (which is detected) from the much larger signal from laser-scattered light.[29]

The laser and detection cells used in the ACA campaign both follow the same design as those used in the Leeds aircraft FAGE instrument which is discussed in detail in Commane et al.[30] The 308 nm laser light was generated by tripling the 924 nm output from a Nd:YAG pumped Ti:Sapphire laser (Photonics Industries Inc). The 308 nm light was generated at a pulse repetition frequency of 5000 Hz and with a typical laser power of 5-15 mW (corresponding to 1-3 μJ per pulse) and delivered to the fluorescence cell via a fibre optic cable (Oz optics). The fluorescence signal was detected using a multi-channel plate (MCP) detector (PMT325/Q/BI/G, Photek Ltd.) equipped with a gating unit (GM10-50, Photek) and fast pre-amplifier (PA200-10, Photek), with the output sent to a PMS-400A photon counting card (Becker & Hickl GmbH). The photon counts are integrated over a one second period, which consists of 5000 excitation laser pulses.

As the FAGE technique does not provide an absolute measurement, a calibration is required in order to relate the fluorescence signal to a concentration. The calibration of the instrument for OH utilises a turbulent reactor,[30] which produces OH following the photolysis of water vapour at 185 nm using a mercury pen-lamp. With knowledge of the concentration of water vapour (determined using a chilled mirror hygrometer), the flux of the lamp at 185 nm and the photolysis time (the product of which is determined using a N_2O chemical actinometer), and the absorption cross-section of water vapour and the OH quantum yield at 185 nm, the OH concentration can be calculated.[30] By changing the flux of the radiation from the pen-lamp at 185 nm, a range of known OH concentrations could be generated and were used to calibrate the fluorescence signal. From the calibration a limit of detection for OH of ~ 1 x 10^6 molecule cm^{-3} was derived for a signal-to-noise ratio of 1 and an averaging time of 2 minutes.

(28) D. Stone, L. K. Whalley and D. E. Heard, Tropospheric OH and HO_2 radicals: field measurements and model comparisons. *Chem. Soc. Rev.*, 2012, **41**, 6348-6404.
(29) D. E. Heard, Atmospheric Field Measurements of the Hydroxyl Radical using Laser-Induced Fluorescence Spectroscopy. *Annu. Rev. Phys. Chem.*, 2006, **57**, 191-216.
(30) R. Commane, C. F. A. Floquet, T. Ingham, D. Stone, M. J. Evans and D. E. Heard, Observations of OH and HO_2 radicals over West Africa. *Atmos. Chem. Phys.*, 2010, **10**, 8783-8801.

Chemicals

PIP (Sigma-Aldrich, ReagentPlus®, 99%), acetonitrile (Sigma-Aldrich, LiChrosolv®, ≥99.8%), styrene (Sigma-Aldrich, ReagentPlus® 99.9 %), 1,2,3-trimethylbenzene (Sigma-Aldrich, 98 %), 1,3,5-trimethylbenzene (Sigma-Aldrich, 98 %), and isoprene (Sigma-Aldrich, analytical standard) were used without further purification. 2-propyl nitrite (isopropyl nitrite, IPN) was synthesized from isopropanol, hydrochloric acid and sodium nitrite, and purified by repeated washing with ice water. The 1:1 nitric acid salt of PIP was prepared by adding a small excess of diluted nitric acid (HNO_3) to diluted PIP followed by rotary evaporation to dryness at 80 °C.

Standards for GCxGC-NCD: N-nitrosopiperidine was commercially available as a 5000 µg mL^{-1} solution in methanol (Sigma-Aldrich Ltd., Dorset, U.K.). N-nitropiperidine was synthesised at the Norwegian University of Life Sciences in Oslo, Norway.

Standards for IC: Ultrapure milli-Q water from an ELGA LabWater purification system (18 MΩ cm^{-1}) was used to prepare the required eluents and analytical standards. A solution of 8 mM Na_2CO_3 /1 mM $NaHCO_3$ (Sigma-Aldrich Ltd., Dorset, U.K.) was used as the eluent for anion-exchange chromatography. Methanesulfonic acid, also available from Sigma-Aldrich Ltd., was used to prepare the eluent for cation-exchange chromatography at concentrations ranging from 20 to 100 mM.

Safety precautions in the syntheses of 1-nitrosopiperidine and 1-nitropiperidine

All reactions have been done in an inert atmosphere (N_2) in a well-ventilated fume hood. Usual precautions have been implemented when working with these compounds, i.e. use of gloves, laboratory coat and safety goggles. Those compounds specially suspected to be cancer promoting or explosive is treated wet and destroyed according to standard laboratory practice. All chemical waste is sealed in special flasks and sent to authorized companies for destruction of such materials.

1-nitrosopiperidine

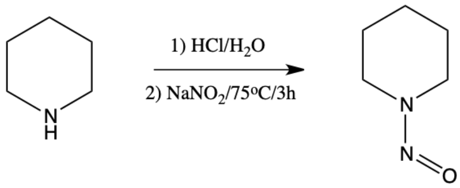

Piperidine (12.88 g; 151.3 mmol) was dissolved in water (5 mL). The reaction mixture was acidified with 6M HCl (≈25 mL). The mixture was stirred vigorously and heated to ≈ 75°C, when $NaNO_2$ (8.98 g; 169.4 mmol; 1.12 eq) dissolved in water (15 mL) was slowly added by means of a dropping funnel. The pH of the reaction mixture was tested regularly and small volumes of 6 M HCl was added to ensure acidity. When all $NaNO_2$ was added the mixture was stirred for another 2 h at this temperature. The aqueous phase was saturated with NaCl and extracted with ether (3 x 30 mL). Drying (K_2CO_3) and distillation using a Claisen apparatus gave the pure compound. Yield of pure nitrosamine as a pale-yellow liquid was 12.53g (73%). Bp. 88-9°C/9mmHg. Spectral data are in accordance to published data.[31]

(31) Zhang, Y.; Zou, P.; Han, Y.; Geng, Y.; Luo, J.; Zhou, B., A combined experimental and DFT mechanistic study for the unexpected nitrosolysis of N-hydroxymethyldialkylamines in fuming nitric acid. *RSC Advances* **2018,** *8* (34), 19310-19316.

1-nitropiperidine

Remarks: A modified version of the method described by Mezyk et al was used.[32] *Following the above procedure yielded only ca. 75% conversion and gave a severe separation problem. Following the modified procedure full conversion was achieved giving pure product according to NMR.*

30% H_2O_2 (15.3g, 135mmol) was dissolved in 25ml DCM was cooled to 0*C. Trifluoro acetic anhydride (27g, 18ml, 135mmol) was added. The solution was stirred for 10 minutes and heated to room temperature. Piperidine-NO (2.0g, 17mmol) was added dropwise. The mixture was reacted at reflux for 4h. The solution was cooled to room temperature and washed with water (2 x 25ml), saturated aqueous solution

of $NaHCO3$ (1 x 10ml) and water (1 x 10ml). Organic phase was dried ($MgSO_4$) and solvent evaporated to give 87% yield of pure nitramine, piperidin-NO2 (2.1g, 14.8mmol).

(32) Mezyk, S. P.; Ewing, D. B.; Kiddle, J. J.; Madden, K. P., Kinetics and Mechanisms of the Reactions of Hydroxyl Radicals and Hydrated Electrons with Nitrosamines and Nitramines in Water. *J. Phys. Chem. A* **2006,** *110* (14), 4732-4737

Computational Results

Table S1. Dipole moments, isotropic polarizabilities and PTR rate coefficients.
Dipole moments (μ /Debye) and isotropic polarizabilities (α /Å^3) obtained in B3LYP/aug-cc-pVTZ and M06-2X/aug-cc-pVTZ calculations.

Molecule	QCC[a]	μ	α	k [b] /10^{-9} cm^3 molecule^{-1} s^{-1}		
				E/N = 65	E/N = 85	E/N = 105
OH	B3L	1.650	1.113			
	M06	1.692	1.063			
$(CH_3)_2CO$	B3L	3.079	6.338	3.569	3.433	3.319
	M06	3.100	6.115	3.568	3.428	3.311
Cyclohexanone	B3L	3.417	10.859	3.877	3.755	3.628
	M06	3.419	10.543	3.856	3.734	3.609
Piperidine (ax)	B3L	1.179	10.279	2.293	2.189	2.116
	M06	1.227	11.712	2.311	2.194	2.112
Piperidine (eq)	B3L	0.825	10.369	2.072	2.029	1.998
	M06	4.660	9.942	2.075	2.022	1.985
1-Nitosopiperidine	B3L	4.705	12.159	4.927	4.711	4.535
	M06	5.065	11.781	4.872	4.657	4.481
1-Nitropiperidine	B3L	4.954	12.628	5.090	4.859	4.671
	M06	4.795	12.112	4.942	4.721	4.539
2,3,4,5-Tetrahydropyridine	B3L	2.247	10.537	3.061	2.940	2.776
	M06	2.923	10.144	3.042	2.925	2.765
Piperidin-4-one (eq)	B3L	3.425	10.554	3.924	3.795	3.674
	M06	3.371	10.171	3.859	3.732	3.613
Piperidin-4-one (ax)	B3L	2.990	10.537	3.613	3.507	3.387
	M06	2.923	10.144	3.538	3.434	3.316

[a] B3L, B3LYP/aug-cc-pVTZ; M06, M06-2X/aug-cc-pVTZ. [b] Collisional rate coefficients at 100 °C (H_3O^+ - analyte molecule) used for determining the PTR-ToF-MS response factors.

Table S2. QCC results for piperidine conformational pathways
Energies (/Hartree) of piperidine conformations and connecting saddle points, and energy differences between these (/kJ mol^{-1}).

Species [a]	M06-2X/aTZ		CCSD(T*)-F12a/aTZ	
	E_{Elec}	E_{ZPE}	E_{Elec}	$\Delta E_{v=0}$
$^4C_1$; Piperidine, *eq*	-251.87635	0.16021	-251.56425	0.0
SP *eq-ax*	-251.86837	0.15866	-251.55521	19.7
$^1C_4$; Piperidine, *ax*	-251.87515	0.16002	-251.56307	2.6
$^{3,N}B$	-251.86446	0.15991	-251.55255	29.9

$^{3}S_1$	-251.86599	0.16009	-251.55395	26.7
$B_{1,4}$	-251.86337	0.15975	-251.55131	32.8
$^{5}S_1$	-251.86513	0.15993	-251.55287	29.1
$^{2,5}B$	-251.86349	0.15978	-251.55137	32.7
$^{2}S_N$	-251.86606	0.16005	-251.55398	26.6
$B_{3,N}$	-251.86436	0.15999	-251.55248	30.3
$^{N}H_1$	-251.85703	0.15999	-251.54502	49.9
$^{3}H_4/^{3}E$	-251.85730	0.15942	-251.54468	49.3
E_3	-251.85694	0.15907	-251.54393	50.3
E_N	-251.85826	0.16024	-251.54657	46.5

[a] Nomenclature according to Ref. 69. Stortz, C. A. Conformational pathways of simple six-membered rings. *J. Phys. Org. Chem.* **2010,** *23*, 1173-1186. DOI: https://doi.org/10.1002/poc.1689

Table S2, continued.
T_1 and D_1 diagnostic values, vibrational frequencies (cm^{-1}), Rotational constants (GHz) and Cartesian coordinates of the species listed above. Results from CCSD(T*)-F12a/aug-cc-pVTZ//M06-2X/aug-cc-pVTZ calculations.

$^{1}C_4$; Piperidine, *eq*	C	0.745800	-1.207902	0.207212
	N	1.373475	-0.000000	-0.314988
T_1= 0.00980401, D_1= 0.02483925	C	0.745801	1.207901	0.207212
	C	-0.712203	1.254865	-0.230204
$\tilde{\nu}$: 250.9, 255.5, 410.2, 435.8, 452.7, 551.9, 775.3, 825.6, 839.9, 878.9, 898.0, 921.2, 983.9, 1061.7, 1070.3, 1076.5, 1158.7, 1173.2, 1174.4, 1196.8, 1291.2, 1299.7, 1320.6, 1352.0, 1366.6, 1384.2, 1385.7, 1426.2, 1475.5, 1485.9, 1487.6, 1495.0, 1503.1, 1514.6, 2948.2, 2949.6, 3040.3, 3060.4, 3060.4, 3098.8, 3100.8, 3102.5, 3106.4, 3110.1, 3569.1	C	-1.446992	0.000000	0.235978
	C	-0.712204	-1.254865	-0.230204
	H	0.784648	-1.245302	1.308636
	H	1.286778	-2.074559	-0.172231
	H	2.362451	-0.000000	-0.106717
	H	1.286779	2.074558	-0.172232
	H	0.784649	1.245302	1.308635
B: 4.567659, 4.476888, 2.564242	H	-1.189887	2.151042	0.168001
	H	-0.745709	1.315028	-1.320333
	H	-2.475621	0.000000	-0.126174
	H	-1.494031	0.000000	1.329696
	H	-1.189887	-2.151041	0.168001
	H	-0.745709	-1.315028	-1.320333
SP *eq-ax*	C	-0.023304	-0.774824	1.230585
	N	-0.572670	-1.272749	-0.000000
T_1= 0.00995196, D_1= 0.02700059	C	-0.023304	-0.774824	-1.230585
	C	-0.023304	0.753883	-1.259134
$\tilde{\nu}$: -613.1, 233.3, 247.2, 387.1, 442.7, 458.0, 576.9, 822.5, 836.3, 859.7, 892.6, 921.5, 969.0, 1037.6, 1056.6, 1076.6, 1155.5, 1174.2, 1177.1, 1237.8, 1284.1, 1306.6, 1348.4, 1353.9, 1375.2, 1379.5, 1389.1, 1417.9, 1456.3, 1483.1, 1486.8, 1490.8, 1490.9, 1504.7, 2956.1, 2957.1, 3034.7, 3050.6, 3051.4, 3096.1, 3097.5, 3101.5, 3110.4, 3111.2, 3745.1	C	0.647892	1.302021	0.000000
	C	-0.023304	0.753883	1.259134
	H	1.015510	-1.119994	1.354845
	H	-0.595122	-1.177725	2.065498
	H	-1.357847	-1.888905	-0.000000
	H	-0.595122	-1.177725	-2.065498

B: 4.509424, 4.492802, 2.545915

H	1.015510	-1.119994	-1.354845
H	0.490164	1.111931	-2.153408
H	-1.056880	1.106816	-1.302560
H	0.629503	2.392446	0.000000
H	1.701631	1.002808	0.000000
H	0.490164	1.111931	2.153408
H	-1.056880	1.106816	1.302560

$^{1}C_{4}$; Piperidine, *ax*

T_1= 0.00976284, D_1= 0.02478778

$\tilde{\nu}$: 245.1, 247.0, 398.8, 438.6, 458.2, 559.3, 761.4, 813.2, 833.6, 871.0, 891.4, 927.8, 953.4, 1026.9, 1060.1, 1076.6, 1126.5, 1159.1, 1190.5, 1232.8, 1284.7, 1300.3, 1344.2, 1346.5, 1375.0, 1387.2, 1387.6, 1401.3, 1475.4, 1486.8, 1487.9, 1492.3, 1495.7, 1507.3, 3039.7, 3041.5, 3045.1, 3047.1, 3049.6, 3099.0, 3100.3, 3106.3, 3113.7, 3114.7, 3545.6

B: 4.533099, 4.438014, 2.559028

C	-1.211992	-0.753426	0.225764
N	-0.000002	-1.463006	-0.178599
C	1.211989	-0.753430	0.225764
C	1.257950	0.708409	-0.233513
C	0.000002	1.451225	0.226230
C	-1.257947	0.708413	-0.233513
H	-1.258108	-0.788081	1.323559
H	-2.078568	-1.308648	-0.146778
H	-0.000003	-1.593716	-1.187618
H	2.078563	-1.308655	-0.146778
H	1.258106	-0.788085	1.323559
H	2.160424	1.197177	0.151651
H	1.318409	0.737768	-1.330748
H	0.000004	2.481205	-0.145979
H	0.000003	1.509975	1.324038
H	-2.160420	1.197185	0.151651
H	-1.318406	0.737772	-1.330748

$^{3,N}B$

T_1= 0.00984168, D_1= 0.02631158

$\tilde{\nu}$: -105.7, 237.1, 316.0, 459.6, 504.5, 623.0, 757.3, 782.8, 820.5, 858.5, 891.7, 933.3, 968.1, 992.4, 1067.3, 1082.9, 1154.6, 1160.3, 1181.6, 1234.4, 1278.6, 1301.9, 1343.8, 1355.5, 1356.1, 1363.4, 1386.7, 1393.2, 1485.9, 1493.1, 1495.7, 1502.3, 1503.9, 1516.6, 3050.1, 3053.8, 3057.2, 3065.7, 3065.7, 3095.8, 3096.3, 3107.5, 3119.4, 3121.5, 3555.3

B: 4.463407, 4.416972, 2.621656

C	-0.207362	-0.795997	1.206909
C	-0.207362	0.757181	1.249694
C	0.453620	1.333599	0.000000
C	-0.207362	0.757181	-1.249694
C	-0.207362	-0.795997	-1.206909
N	0.378196	-1.366584	-0.000000
H	-1.231875	-1.168610	1.272090
H	0.328358	-1.203288	2.063217
H	-1.233493	1.127317	1.301093
H	0.299387	1.114443	2.146339
H	1.519807	1.094204	0.000000
H	0.383236	2.421200	0.000000
H	-1.233493	1.127317	-1.301093
H	0.299387	1.114443	-2.146339
H	-1.231875	-1.168610	-1.272090
H	0.328358	-1.203288	-2.063217
H	1.379797	-1.224846	-0.000000

$^{3}S_{1}$

T_1= 0.00977563, D_1= 0.02527939

$\tilde{\nu}$: 128.7, 251.5, 297.7, 446.1, 496.5, 611.9, 770.4, 771.9, 828.2, 872.2, 900.2, 945.3, 953.4, 1006.2, 1059.9, 1082.1, 1148.2, 1155.7, 1184.8, 1230.9, 1275.0, 1295.0, 1339.3, 1342.9, 1359.7, 1373.0, 1384.9, 1397.6, 1486.0, 1494.0, 1496.1, 1503.2, 1505.6, 1517.8, 3045.8, 3051.7, 3055.7, 3061.0, 3063.9, 3092.9, 3094.7, 3110.1, 3113.5, 3117.6, 3554.1

B: 4.581605, 4.351365, 2.660009

C	0.745529	-1.136492	0.357472
C	1.491385	0.173641	0.030460
C	0.499558	1.269001	-0.388987
C	-0.803438	1.104023	0.384721
C	-1.466233	-0.234192	0.001176
N	-0.507966	-1.279512	-0.372916
H	0.498639	-1.171119	1.420991
H	1.376801	-2.001501	0.157451
H	2.056013	0.498900	0.906770
H	2.216901	0.004186	-0.766771
H	0.287497	1.188999	-1.458364
H	0.931662	2.256759	-0.232816
H	-0.584083	1.120424	1.455188

H	-1.490416	1.928332	0.193326
H	-2.071837	-0.598808	0.834234
H	-2.141946	-0.086204	-0.841691
H	-0.324273	-1.239270	-1.366960

$B_{1,4}$

T_1= 0.00977563, D_1= 0.02527939

$\tilde{\nu}$: -116.3, 223.6, 314.9, 453.8, 474.2, 577.7, 711.9, 783.0, 830.4, 869.4, 896.3, 915.7, 984.2, 1028.1, 1049.9, 1098.8, 1164.7, 1167.7, 1177.6, 1247.8, 1272.7, 1295.2, 1310.3, 1348.6, 1367.4, 1383.6, 1390.1, 1409.2, 1482.1, 1491.1, 1494.0, 1509.3, 1517.8, 1529.2, 3027.1, 3051.6, 3055.8, 3061.6, 3067.9, 3076.4, 3090.9, 3108.6, 3109.8, 3119.6, 3582.0

B: 4.539534, 4.343376, 2.607030

C	0.563150	-1.202624	0.422266
C	1.460593	-0.140534	-0.213710
C	0.771606	1.245468	-0.211589
C	-0.617767	1.183558	0.421720
C	-1.451807	0.078307	-0.215857
N	-0.785799	-1.233981	-0.132567
H	0.494006	-1.033420	1.498624
H	0.998629	-2.193788	0.298920
H	1.683395	-0.444690	-1.238211
H	2.414616	-0.102242	0.314469
H	0.666207	1.606565	-1.236396
H	1.384555	1.979121	0.311297
H	-1.133800	2.136355	0.301687
H	-0.536525	1.008311	1.496046
H	-1.629890	0.344802	-1.262595
H	-2.429465	0.024170	0.268550
H	-0.765787	-1.672362	-1.041402

$^{5}S_1$

T_1= 0.00964001, D_1= 0.02353829

$\tilde{\nu}$: 131.2, 267.2, 291.0, 452.8, 468.0, 561.3, 716.3, 781.3, 839.0, 879.3, 897.1, 919.2, 992.2, 1046.3, 1052.2, 1093.8, 1152.6, 1159.0, 1183.9, 1232.5, 1260.3, 1292.1, 1316.5, 1344.2, 1368.3, 1380.6, 1387.6, 1405.8, 1480.9, 1487.7, 1496.0, 1504.0, 1518.9, 1531.1, 2979.2, 3033.2, 3057.7, 3064.6, 3069.9, 3086.1, 3095.8, 3109.5, 3111.5, 3122.8, 3580.4

B: 4.631633, 4.323304, 2.650219

C	0.668984	1.165591	0.399564
C	-0.635642	1.210526	-0.389620
C	-1.505258	-0.003715	-0.021576
C	-0.633452	-1.205201	0.381852
C	0.689635	-1.175477	-0.367453
N	1.474251	-0.022114	0.086929
H	0.435917	1.169255	1.468720
H	1.275171	2.050958	0.211831
H	-0.399308	1.196231	-1.456083
H	-1.169486	2.141344	-0.196568
H	-2.138154	-0.272322	-0.868508
H	-2.174158	0.253538	0.800611
H	-1.155375	-2.141562	0.188732
H	-0.416939	-1.175206	1.451526
H	0.478450	-1.142094	-1.445693
H	1.261838	-2.084449	-0.179339
H	2.176675	0.208753	-0.600332

${}^{2,5}B$

T_1= 0.00963380, D_1= 0.02341760

$\tilde{\nu}$: -115.5, 243.4, 327.6, 449.0, 476.7, 544.5, 706.7, 784.3, 835.7, 880.2, 895.4, 914.9, 1002.3, 1051.6, 1060.6, 1103.3, 1145.4, 1170.7, 1193.1, 1234.7, 1267.5, 1293.6, 1316.7, 1350.0, 1370.4, 1378.0, 1385.3, 1421.4, 1480.2, 1484.3, 1497.7, 1509.4, 1525.1, 1534.6, 2975.0, 3024.6, 3060.0, 3063.3, 3075.1, 3076.3, 3095.4, 3099.6, 3110.6, 3124.3, 3597.6

B: 4.553428, 4.370062, 2.609208

C	0.175133	-1.327113	0.389542
C	1.345269	-0.571747	-0.226175
C	1.123346	0.959068	-0.172224
C	-0.233827	1.309448	0.436990
C	-1.341996	0.521035	-0.251236
N	-1.060872	-0.925243	-0.272371
H	0.160471	-1.157616	1.475520
H	0.305118	-2.398776	0.238628
H	1.446130	-0.896417	-1.261864
H	2.264578	-0.848477	0.289960
H	1.165337	1.370949	-1.181819
H	1.918294	1.444076	0.393998
H	-0.432623	2.376499	0.334053

H	-0.232656	1.091824	1.506682
H	-1.435461	0.881436	-1.280607
H	-2.297397	0.716556	0.240348
H	-1.843236	-1.447495	0.090312

$^{2}S_{N}$

T_1= 0.00967029, D_1= 0.02352092

$\tilde{\nu}$: 125.8, 247.9, 307.4, 441.9, 486.9, 578.6, 762.4, 784.6, 843.2, 896.4, 906.3, 924.4, 976.3, 1042.3, 1075.9, 1088.2, 1153.3, 1174.4, 1195.4, 1208.8, 1277.3, 1293.0, 1310.5, 1345.5, 1361.8, 1368.4, 1386.1, 1420.1, 1473.2, 1486.0, 1501.2, 1502.6, 1515.0, 1528.3, 2963.7, 2988.4, 3058.8, 3065.2, 3074.5, 3076.4, 3101.9, 3113.6, 3114.6, 3123.7, 3583.7

B: 4.638467, 4.364253, 2.663741

C	-0.534518	-1.237382	0.350029
C	-1.505675	-0.096973	0.011598
C	-0.728025	1.168147	-0.383905
C	0.587528	1.222838	0.385618
C	1.485650	0.041708	-0.014723
N	0.663050	-1.083844	-0.465513
H	-0.302279	-1.225030	1.425655
H	-0.988437	-2.203275	0.134048
H	-2.159324	-0.397860	-0.806961
H	-2.140256	0.105117	0.876423
H	-0.512465	1.146234	-1.452581
H	-1.326073	2.058825	-0.193007
H	1.114113	2.160118	0.209785
H	0.371740	1.180748	1.455734
H	2.139408	0.334305	-0.838029
H	2.125514	-0.228042	0.835881
H	1.206947	-1.934260	-0.480060

$B_{3,N}$

T_1= 0.00969305, D_1= 0.02390277

$\tilde{\nu}$: -109.4, 228.7, 325.1, 455.6, 491.3, 601.0, 774.9, 805.0, 836.2, 892.6, 900.2, 927.8, 978.7, 1024.4, 1081.5, 1092.9, 1167.4, 1173.3, 1198.4, 1219.6, 1276.5, 1308.8, 1324.8, 1337.4, 1354.4, 1368.5, 1389.9, 1421.1, 1474.6, 1490.9, 1495.4, 1504.4, 1516.8, 1528.6, 2962.5, 2965.2, 3065.6, 3067.1, 3078.9, 3095.7, 3096.2, 3113.5, 3115.2, 3127.6, 3572.1

B: 4.507984, 4.447623, 2.630913

C	0.200350	-0.790520	1.200072
C	0.200350	0.755728	1.254166
C	-0.437552	1.349969	-0.000000
C	0.200350	0.755728	-1.254166
C	0.200350	-0.790520	-1.200072
N	-0.490995	-1.245611	0.000000
H	-0.311635	-1.204710	2.067486
H	1.236996	-1.158159	1.226598
H	-0.327475	1.104505	2.141254
H	1.226430	1.118586	1.336853
H	-1.505239	1.135494	-0.000000
H	-0.322575	2.434191	-0.000000
H	-0.327475	1.104505	-2.141254
H	1.226430	1.118586	-1.336853
H	-0.311635	-1.204710	-2.067486
H	1.236996	-1.158159	-1.226598
H	-0.566939	-2.253157	0.000000

$^{N}H_{1}$

T_1= 0.00971881, D_1= 0.02482964

$\tilde{\nu}$: -233.0, 75.1, 329.8, 458.7, 481.8, 674.9, 734.3, 778.8, 801.2, 850.0, 900.0, 944.8, 978.4, 996.1, 1078.7, 1138.5, 1162.1, 1172.6, 1190.0, 1265.5, 1294.9, 1306.2, 1343.0, 1353.4, 1357.3, 1385.4, 1386.5, 1403.6, 1482.3, 1491.7, 1500.4, 1503.4, 1511.6, 1528.5, 3046.7, 3051.8, 3054.0, 3073.7, 3085.9, 3090.0, 3096.6, 3100.9, 3114.0, 3129.5, 3526.2

B: 4.438646, 4.320241, 2.487633

C	-0.975750	1.112069	0.132744
C	1.485406	0.177048	-0.095162
C	0.531545	1.393089	-0.112502
C	0.810264	-1.106192	0.370816
C	-1.442804	-0.366418	0.055594
H	-1.551791	1.703235	-0.578147
H	1.859453	-0.005398	-1.105371
H	0.862275	2.112796	0.634823
H	0.571387	-1.056352	1.437153
H	-1.803637	-0.674060	1.039360
H	-1.243504	1.496529	1.116193
H	2.360737	0.390633	0.519668
H	0.641732	1.901120	-1.069221

H	1.477771	-1.957345	0.232721
H	-2.288530	-0.458670	-0.624256
N	-0.438268	-1.345263	-0.339168
H	-0.269986	-1.293231	-1.337692

$^{3}H_{4}/^{3}E$

T_1= 0.00966829, D_1= 0.02383924

$\tilde{\nu}$: -251.2, 27.5, 321.6, 424.7, 476.6, 510.7, 687.5, 805.7, 808.3, 884.7, 902.4, 914.2, 980.6, 1007.6, 1081.9, 1092.8, 1164.9, 1169.9, 1200.9, 1271.9, 1289.1, 1304.5, 1317.8, 1353.8, 1378.2, 1390.8, 1394.4, 1433.1, 1493.4, 1495.1, 1506.1, 1510.8, 1530.8, 1536.7, 3026.4, 3030.2, 3042.8, 3048.4, 3058.5, 3061.7, 3066.9, 3104.7, 3106.0, 3109.4, 3652.0

B: 4.476149, 4.364099, 2.514150

C	1.341833	-0.663496	0.034265
C	1.159280	0.835052	0.279191
C	-0.073925	1.349670	-0.447249
C	-1.283387	0.696687	0.204580
C	-1.233116	-0.835821	0.087553
N	0.097204	-1.426250	-0.088609
H	1.962900	-1.082888	0.831004
H	1.906651	-0.784991	-0.893765
H	1.033158	1.029977	1.347118
H	2.066630	1.353735	-0.030801
H	-0.021800	1.088977	-1.508783
H	-0.142979	2.435458	-0.381156
H	-1.291183	0.988020	1.257174
H	-2.219044	1.055042	-0.224972
H	-1.719486	-1.269665	0.965632
H	-1.837066	-1.134126	-0.771468
H	0.117679	-1.988343	-0.919757

E_3

T_1= 0.00964590, D_1= 0.02331506

$\tilde{\nu}$: -244.3, 50.5, 315.3, 335.4, 465.6, 475.0, 680.1, 815.2, 825.3, 889.6, 909.0, 910.3, 992.6, 1024.3, 1084.5, 1094.7, 1149.4, 1172.9, 1209.8, 1258.7, 1280.2, 1307.1, 1325.0, 1353.1, 1367.6, 1385.1, 1387.8, 1436.6, 1490.4, 1494.0, 1511.0, 1517.0, 1538.9, 1545.1, 3011.5, 3014.7, 3027.2, 3027.3, 3051.9, 3056.3, 3062.0, 3107.2, 3109.4, 3112.9, 3645.3

B: 4.514293, 4.367290, 2.510352

C	0.135913	-0.738415	1.297749
C	0.135913	0.790040	1.225327
C	-0.619833	1.285285	-0.000000
C	0.135913	0.790040	-1.225327
C	0.135913	-0.738415	-1.297749
N	-0.003529	-1.392876	0.000000
H	-0.707692	-1.049255	1.922156
H	1.042589	-1.068099	1.814978
H	-0.285425	1.187158	2.148390
H	1.160977	1.162402	1.157125
H	-1.643768	0.901819	-0.000000
H	-0.675491	2.373945	-0.000000
H	-0.285425	1.187158	-2.148390
H	1.160977	1.162402	-1.157125
H	-0.707692	-1.049255	-1.922156
H	1.042589	-1.068099	-1.814978
H	0.380142	-2.321266	0.000000

E_N

T_1= 0.00969305, D_1= 0.02390277

$\tilde{\nu}$: -223.3, 43.9, 383.4, 464.4, 469.6, 663.6, 734.8, 802.9, 807.0, 869.2, 908.7, 934.5, 1025.8, 1025.9, 1107.9, 1129.4, 1147.7, 1188.8, 1225.9, 1240.3, 1301.6, 1311.7, 1331.2, 1350.6, 1361.1, 1386.0, 1390.4, 1433.6, 1477.4, 1489.4, 1498.0, 1514.1, 1518.6, 1529.8, 2954.9, 2957.2, 3071.2, 3075.7, 3091.5, 3096.7, 3100.5, 3107.2, 3115.4, 3134.9, 3565.8

B: 4.436083, 4.396454, 2.494895

C	1.181663	0.806184	0.214180
C	1.329513	-0.708378	0.050096
C	0.000105	-1.479611	-0.147893
C	-1.329409	-0.708565	0.050113
C	-1.181784	0.806026	0.214156
N	-0.000084	1.273406	-0.495943
H	2.063668	1.308044	-0.181802
H	1.113589	1.058628	1.284044
H	1.959297	-0.898393	-0.818124
H	1.868879	-1.100986	0.911964
H	0.000126	-1.870272	-1.162883
H	0.000169	-2.354319	0.500812
H	-1.959191	-0.898700	-0.818083
H	-1.868692	-1.101231	0.912007

H	-2.063850	1.307747	-0.181864
H	-1.113778	1.058508	1.284015
H	-0.000153	2.283189	-0.542397

Kinetics and branching in the piperidine + OH reaction

Table S3. QCC results for the piperidine (*eq*) reaction with OH radicals
Electronic energies of reactants, intermediates and products (/Hartree), and relative energies including Zero Point Energies, Δ (/kJ mol^{-1}), of stationary points on the potential energy surface of the piperidine + OH reaction, Figure 2 in the main text.

Species	M06-2X/aTZ		CCSD(T*)-F12a/aTZ	
	E_{Elec}	E_{ZPE}	E_{Elec}	$\Delta E_{v=0}$
Piperidine, *eq* ($^4C_1$)	-251.87635	0.16021	-251.56425	
OH	-75.73381	0.00859	-75.67069	
Sum reactants	-327.61016	0.16880	-327.23494	0.0
$PRE_{eq}^{7\text{-}1}$	-327.62130	0.17118	-327.24529	-20.9
$SP_{eq}^{7\text{-}1}$	-327.61577	0.17022	-327.23933	-7.8
$PRE_{eq}^{7\text{-}2}$	-327.61626	0.17046	-327.23917	-6.7
$SP_{eq}^{7\text{-}2}$	-327.61564	0.16906	-327.23998	-12.6
$POST_{eq}^{7}$	-327.65782	0.17026	-327.28386	-124.6
PIPṄ	-251.21565	0.14592	-250.90064	
H_2O	-76.43011	0.02155	-76.37098	
Sum products	-327.64576	0.16748	-327.27163	-99.8
$PRE_{eq}^{8\text{-}1}$	-327.61466	0.16993	-327.23769	-4.3
$SP_{eq}^{8\text{-}1}$	-327.61428	0.16875	-327.23809	-8.4
$PRE_{eq}^{8\text{-}2}$	-327.61466	0.16993	-327.23767	-4.2
$SP_{eq}^{8\text{-}2}$	-327.61428	0.16889	-327.23810	-8.1
$POST_{eq}^{8}$	-327.66298	0.17081	-327.28655	-130.2
$PRE_{eq}^{9\text{-}1}$	-327.62652	0.17203	-327.24995	-30.9
$SP_{eq}^{9\text{-}1}$	-327.61035	0.16781	-327.23480	-2.2
$PRE_{eq}^{9\text{-}2}$	-327.61455	0.17003	-327.23764	-3.9
$SP_{eq}^{9\text{-}2}$	-327.60722	0.16698	-327.23200	2.9
$POST_{eq}^{9}$	-327.66315	0.17098	-327.28791	-133.3
PIPĊ^2	-251.22262	0.14655	-250.90654	
H_2O	-76.43011	0.02155	-76.37098	
Sum products	-327.65272	0.16810	-327.27752	-113.6
PRE_{eq}^{10} ($PRE_{eq}^{9\text{-}1}$)	-327.62652	0.17203	-327.24995	-30.9
$SP_{eq}^{10\text{-}1}$	-327.61414	0.16790	-327.23848	-11.7

$SP^{10\text{-}2}_{eq}$	-327.60664	0.16687	-327.23131	4.5
$POST^{10}_{eq}$	-327.65274	0.17025	-327.27565	-103.1
$PRE^{11\text{-}1}_{eq}$	-327.61373	0.16998	-327.23467	3.8
$SP^{11\text{-}1}_{eq}$	-327.60701	0.16692	-327.23206	2.6
$PRE^{11\text{-}2}_{eq}$	-327.61465	0.17007	-327.23767	-3.8
$SP^{11\text{-}2}_{eq}$	-327.60701	0.16690	-327.23179	3.3
$POST^{11}_{eq}$	-327.64360	0.16903	-327.26749	-84.9
PIPĊ^{3}	-251.20842	0.14522	-250.89186	
H_2O	-76.43011	0.02155	-76.37098	
Sum products	-327.63853	0.16677	-327.26284	-78.6
$PRE^{12\text{-}1}_{eq}$	-327.61466	0.16993	-327.23767	-4.2
$SP^{12\text{-}1}_{eq}$	-327.60869	0.16730	-327.23358	-0.4
$POST^{12\text{-}1}_{eq}$	-327.64789	0.16925	-327.27123	-94.1
$PRE^{12\text{-}2}_{eq}$	-327.61465	0.17008	-327.23767	-3.8
$SP^{12\text{-}2}_{eq}$	-327.60883	0.16703	-327.23309	0.2
$POST^{12\text{-}2}_{eq}$	-327.64733	0.16902	-327.27033	-92.3
$PRE^{13\text{-}1}_{eq}$	-327.61352	0.17002	-327.23700	-2.2
$SP^{13\text{-}1}_{eq}$	-327.60742	0.16689	-327.23243	1.6
$PRE^{13\text{-}2}_{eq}$	-327.61403	0.17020	-327.23735	-2.7
$SP^{13\text{-}2}_{eq}$	-327.60767	0.16697	-327.23239	1.9
$POST^{13}_{eq}$	-327.64550	0.16888	-327.26951	-90.6
PIPĊ^{4}	-251.21082	0.14546	-250.89424	
H_2O	-76.43011	0.02155	-76.37098	
Sum products	-327.64092	0.16701	-327.26522	-84.2

Table S3, continued.
T_1 and D_1 diagnostic values, vibrational frequencies (cm^{-1}), Rotational constants (GHz) and Cartesian coordinates of the species listed above. Results from CCSD(T*)-F12a/aug-cc-pVTZ//M06-2X/aug-cc-pVTZ calculations.

Piperidine *eq*; ($^1C_4$)	C	0.745800	-1.207902	0.207212
	N	1.373475	-0.000000	-0.314988
T_1= 0.00980401, D_1= 0.02483925	C	0.745801	1.207901	0.207212
	C	-0.712203	1.254865	-0.230204
$\tilde{\nu}$: 250.9, 255.5, 410.2, 435.8, 452.7, 551.9, 775.3, 825.6, 839.9,	C	-1.446992	0.000000	0.235978
878.9, 898.0, 921.2, 983.9, 1061.7, 1070.3, 1076.5, 1158.7,	C	-0.712204	-1.254865	-0.230204
1173.2, 1174.4, 1196.8, 1291.2, 1299.7, 1320.6, 1352.0, 1366.6,	H	0.784648	-1.245302	1.308636

1384.2, 1385.7, 1426.2, 1475.5, 1485.9, 1487.6, 1495.0, 1503.1, 1514.6, 2948.2, 2949.6, 3040.3, 3060.4, 3060.4, 3098.8, 3100.8, 3102.5, 3106.4, 3110.1, 3569.1

B: 4.567659 4.476888 2.564242

H	1.286778	-2.074559	-0.172231
H	2.362451	-0.000000	-0.106717
H	1.286779	2.074558	-0.172232
H	0.784649	1.245302	1.308635
H	-1.189887	2.151042	0.168001
H	-0.745709	1.315028	-1.320333
H	-2.475621	0.000000	-0.126174
H	-1.494031	0.000000	1.329696
H	-1.189887	-2.151041	0.168001
H	-0.745709	-1.315028	-1.320333

OH

T_1= 0.00799671 , D_1= 0.01451825

$\tilde{\nu}$: 3769.4

B: 0.000000 564.286338 564.286338

O	-0.000000	-0.000000	0.107992
H	0.000000	0.000000	-0.863937

PRE^{7-1}_{eq}

T_1= 0.01814158, D_1= 0.09861294

$\tilde{\nu}$: 78.6, 86.0, 135.1, 136.1, 255.1, 262.9, 401.3, 424.6, 449.4, 455.7, 572.9, 726.7, 825.5, 838.6, 869.9, 897.4, 918.0, 978.8, 1054.6, 1062.3, 1076.7, 1163.3, 1168.1, 1177.5, 1192.8, 1287.8, 1301.1, 1326.5, 1354.5, 1368.3, 1382.7, 1382.9, 1415.2, 1468.2, 1482.8, 1487.9, 1494.3, 1495.2, 1509.7, 2921.5, 2922.0, 3038.9, 3063.0, 3063.1, 3101.5, 3109.6, 3112.4, 3115.2, 3116.3, 3525.6, 3771.2

B: 4.081088 1.696689 1.366433

C	-0.358178	0.897816	0.965455
N	-0.765266	-0.470251	0.676401
C	0.352428	-1.388788	0.519350
C	1.232669	-0.930820	-0.636331
C	1.696761	0.507030	-0.414208
C	0.500494	1.425523	-0.175082
H	0.216329	0.945576	1.903731
H	-1.256669	1.500528	1.080194
H	-1.454906	-0.806371	1.331939
H	-0.039077	-2.389567	0.338025
H	0.961739	-1.429913	1.436132
H	2.085202	-1.603298	-0.737119
H	0.654804	-0.987974	-1.562315
H	2.282695	0.854042	-1.265631
H	2.355719	0.536976	0.459549
H	0.833047	2.438025	0.055668
H	-0.120755	1.470703	-1.071371
O	-2.450473	0.047855	-0.859005
H	-2.102522	-0.684379	-1.386661

SP^{7-1}_{eq}

T_1= 0.02881765, D_1= 0.17491830

$\tilde{\nu}$: -317.6, 45.0, 98.1, 109.6, 230.0, 252.7, 351.0, 398.6, 455.3, 481.2, 588.7, 646.9, 823.8, 838.0, 868.8, 897.0, 915.7, 978.5, 1044.7, 1059.4, 1077.0, 1159.8, 1166.9, 1176.3, 1195.2, 1286.1, 1299.6, 1321.2, 1352.9, 1365.0, 1382.7, 1383.8, 1399.9, 1452.6, 1482.4, 1488.4, 1494.8, 1495.0, 1510.1, 2942.9, 2952.2, 3039.5, 3064.1, 3064.1, 3103.1, 3111.5, 3113.8, 3123.8, 3123.8, 3195.0, 3803.9

B: 4.271363 1.736422 1.370145

C	0.220480	-1.215391	-0.020702
N	0.712750	-0.000001	-0.638764
C	0.220485	1.215389	-0.020699
C	-1.300419	1.257042	-0.138253
C	-1.914053	0.000002	0.475203
C	-1.300425	-1.257038	-0.138257
H	0.489095	-1.260727	1.047301
H	0.681595	-2.069894	-0.512099
H	1.714195	-0.000003	-0.850728
H	0.681603	2.069891	-0.512094
H	0.489104	1.260721	1.047302
H	-1.680970	2.152642	0.353916
H	-1.567879	1.318772	-1.195242
H	-2.996301	0.000005	0.343524
H	-1.724643	0.000000	1.553371
H	-1.680978	-2.152638	0.353909
H	-1.567883	-1.318764	-1.195246
O	3.004148	0.000009	0.246756
H	2.584214	-0.000091	1.119634

$PRE_{eq}^{7\text{-}2}$

T_1= 0.02194332, D_1= 0.12619825

$\tilde{\nu}$: 46.3, 77.7, 128.3, 147.4, 260.6, 271.8, 411.0, 440.3, 454.6, 502.8, 567.9, 748.4, 824.1, 839.3, 875.5, 899.6, 914.7, 979.2, 1059.3, 1064.4, 1076.7, 1159.4, 1170.2, 1174.0, 1200.0, 1287.3, 1300.4, 1322.2, 1353.0, 1367.8, 1384.8, 1385.8, 1418.8, 1476.5, 1486.4, 1488.4, 1493.9, 1498.7, 1510.1, 2962.0, 2966.1, 3039.6, 3056.5, 3070.4, 3101.7, 3103.4, 3108.1, 3118.4, 3128.6, 3597.3, 3821.3

B: 3.254309 2.062302 1.759755

C	-0.358178	0.897816	0.965455
N	-0.765266	-0.470251	0.676401
C	0.352428	-1.388788	0.519350
C	1.232669	-0.930820	-0.636331
C	1.696761	0.507030	-0.414208
C	0.500494	1.425523	-0.175082
H	0.216329	0.945576	1.903731
H	-1.256669	1.500528	1.080194
H	-1.454906	-0.806371	1.331939
H	-0.039077	-2.389567	0.338025
H	0.961739	-1.429913	1.436132
H	2.085202	-1.603298	-0.737119
H	0.654804	-0.987974	-1.562315
H	2.282695	0.854042	-1.265631
H	2.355719	0.536976	0.459549
H	0.833047	2.438025	0.055668
H	-0.120755	1.470703	-1.071371
O	-2.450473	0.047855	-0.859005
H	-2.102522	-0.684379	-1.386661

$SP_{eq}^{7\text{-}2}$

T_1= 0.03808854, D_1= 0.23674759

$\tilde{\nu}$: -487.1, 100.5, 119.1, 184.0, 224.3, 251.7, 327.2, 402.6, 447.2, 461.3, 555.1, 610.6, 813.5, 836.6, 862.9, 897.0, 915.2, 965.0, 1033.5, 1054.7, 1080.0, 1161.6, 1166.2, 1176.3, 1231.1, 1286.7, 1305.0, 1339.8, 1354.5, 1372.5, 1379.0, 1387.2, 1404.9, 1472.3, 1477.7, 1484.4, 1488.9, 1494.0, 1500.8, 2996.5, 2998.2, 3036.7, 3061.6, 3073.1, 3102.5, 3109.6, 3121.3, 3123.6, 3141.1, 3504.3, 3823.3

B: 3.111222 2.252329 1.927509

C	-0.216026	1.165770	0.833625
N	-0.817622	-0.144956	0.878554
C	0.036842	-1.295509	0.727183
C	0.973143	-1.166818	-0.474593
C	1.706806	0.171572	-0.434523
C	0.710948	1.326982	-0.369310
H	0.363465	1.312505	1.755158
H	-1.014270	1.903430	0.814376
H	-1.817730	-0.231897	0.750982
H	-0.587374	-2.184532	0.654563
H	0.648418	-1.395347	1.634166
H	1.678381	-1.998912	-0.473278
H	0.387137	-1.229429	-1.394395
H	2.352246	0.274418	-1.306987
H	2.356606	0.198548	0.446661
H	1.232719	2.282059	-0.298332
H	0.095334	1.336951	-1.269573
O	-2.169001	0.038189	-0.967543
H	-1.889852	-0.770593	-1.417167

$POST_{eq}^{7}$

T_1= 0.01383997, D_1= 0.06178438

$\tilde{\nu}$: 24.6, 62.4, 88.2, 155.0, 226.7, 276.9, 344.4, 413.6, 443.7, 460.5, 564.7, 625.8, 817.7, 841.7, 858.4, 888.6, 900.1, 978.5, 983.6, 1036.7, 1076.3, 1100.8, 1145.7, 1146.6, 1249.4, 1250.2, 1286.4, 1295.9, 1330.0, 1333.4, 1350.5, 1384.4, 1385.7, 1485.7, 1488.2, 1497.2, 1501.3, 1522.4, 1625.4, 2992.6, 3001.0, 3045.3, 3068.4, 3069.7, 3102.0, 3112.6, 3114.2, 3118.5, 3120.7, 3609.8, 3932.3

B: 2.847972 1.892448 1.798524

C	-0.326331	-1.193421	0.922725
N	0.459852	-0.009523	1.153084
C	-0.315534	1.183782	0.934915
C	-0.707500	1.255962	-0.553998
C	-1.492920	0.010022	-0.948682
C	-0.718526	-1.246776	-0.566889
H	-1.242511	-1.171244	1.528619
H	0.253921	-2.073159	1.196543
H	2.088542	-0.006541	0.112533
H	0.272812	2.055347	1.217437
H	-1.231757	1.163766	1.540820
H	-1.285233	2.164273	-0.726000
H	0.207262	1.321105	-1.146469
H	-1.696826	0.016460	-2.019916
H	-2.459685	0.011609	-0.436432
H	-1.304097	-2.148170	-0.748508

H	0.195785	-1.313607	-1.159879
O	2.602803	0.000497	-0.713239
H	3.525261	-0.014576	-0.452841

PIPṄ

T_1= 0.01389069, D_1= 0.05612892

$\tilde{\nu}$: 217.5, 237.5, 364.2, 445.3, 454.7, 562.1, 811.8, 840.0, 857.9, 891.7, 894.9, 971.2, 980.6, 1037.3, 1075.9, 1106.1, 1137.2, 1140.5, 1243.1, 1251.0, 1284.1, 1294.9, 1332.0, 1336.1, 1351.9, 1383.7, 1387.6, 1482.5, 1488.7, 1494.0, 1499.4, 1517.9, 2978.7, 2987.6, 3047.0, 3063.0, 3063.4, 3100.3, 3106.9, 3108.8, 3110.7, 3113.8

B: 4.652185 4.648684 2.646002

C	-0.031830	-0.829670	1.186394
N	-0.715167	-1.280712	0.000000
C	-0.031830	-0.829670	-1.186394
C	-0.031830	0.706571	-1.250223
C	0.620061	1.286733	-0.000000
C	-0.031830	0.706571	1.250223
H	1.011884	-1.176624	1.173678
H	-0.520082	-1.249767	2.064543
H	-0.520082	-1.249767	-2.064543
H	1.011884	-1.176624	-1.173678
H	0.483802	1.030011	-2.154836
H	-1.065924	1.050895	-1.321138
H	0.545041	2.374817	-0.000000
H	1.685317	1.037933	-0.000000
H	0.483802	1.030011	2.154836
H	-1.065924	1.050895	1.321138

H_2O

T_1= 0.00895900, D_1= 0.01776535

$\tilde{\nu}$: 3971.4, 3868.5, 1619.7

B: 834.0336966 430.9641048 284.1416681

0.000000	0.000000	0.114963
0.000000	0.762576	-0.466991
0.000000	-0.762576	-0.466991

$PRE^{8\text{-}1}_{eq}$

T_1= 0.01404010, D_1= 0.06441524

$\tilde{\nu}$: 36.0, 62.7, 108.0, 110.3, 260.0, 267.8, 341.2, 432.1, 443.3, 455.5, 556.6, 761.6, 821.8, 840.4, 869.4, 898.4, 923.2, 974.6, 1061.8, 1070.4, 1078.2, 1160.6, 1164.8, 1170.7, 1201.1, 1285.6, 1291.5, 1313.7, 1346.9, 1364.4, 1380.6, 1384.7, 1421.2, 1475.3, 1485.8, 1486.8, 1494.8, 1498.5, 1510.5, 2834.5, 2914.9, 3040.0, 3061.5, 3062.1, 3100.3, 3103.3, 3104.6, 3109.0, 3113.9, 3574.2, 3759.0

B: 2.694695 1.856712 1.778555

C	0.370728	-0.258297	1.246475
N	-0.527696	-1.277175	0.732570
C	-0.768469	-1.132910	-0.693368
C	-1.425631	0.212292	-0.972368
C	-0.565000	1.351076	-0.430047
C	-0.234903	1.123157	1.042983
H	1.355824	-0.288472	0.736129
H	0.554830	-0.449152	2.303422
H	-0.181083	-2.201753	0.946802
H	-1.406423	-1.950112	-1.028752
H	0.172720	-1.196220	-1.270820
H	-1.590756	0.328896	-2.044334
H	-2.401632	0.226022	-0.482552
H	-1.066906	2.309236	-0.567435
H	0.370395	1.403112	-0.996966
H	0.458459	1.883944	1.402185
H	-1.148484	1.192864	1.637938
O	2.769271	0.005936	-0.686633
H	2.162410	-0.137541	-1.432594

$SP^{8\text{-}1}_{eq}$

T_1= 0.02110095, D_1= 0.11958180

$\tilde{\nu}$: -263.6, 41.3, 77.4, 87.2, 248.8, 260.0, 394.9, 438.3, 451.2, 516.9, 588.9, 744.0, 823.7, 841.2, 869.0, 898.3, 924.8, 973.9, 1062.2, 1070.1, 1077.9, 1150.8, 1165.2, 1170.4, 1202.5, 1275.7, 1286.7, 1309.2, 1345.7, 1361.9, 1379.6, 1385.0, 1421.7, 1465.3, 1475.8, 1486.5, 1494.3, 1497.0, 1509.2, 2305.7, 2947.4, 3033.6, 3060.2, 3063.1, 3100.9, 3104.4, 3108.0, 3111.0, 3114.4, 3577.4,

C	0.587406	0.026244	1.119373
N	-0.201869	-1.162542	0.922106
C	-0.708889	-1.286800	-0.436469
C	-1.603003	-0.098158	-0.762785
C	-0.842121	1.210814	-0.565816
C	-0.232806	1.274929	0.832369
H	1.484495	0.032785	0.434301
H	0.986976	0.036066	2.133234
H	0.308496	-1.989397	1.198537
H	-1.264082	-2.220355	-0.519413

3778.7

B: 2.841610 1.916245 1.775620

H	0.118767	-1.324784	-1.164926
H	-1.969099	-0.181478	-1.786702
H	-2.468685	-0.120627	-0.097335
H	-1.495765	2.066107	-0.738548
H	-0.040698	1.277197	-1.309721
H	0.397036	2.158156	0.939386
H	-1.031815	1.341742	1.574780
O	2.643931	0.006333	-0.836062
H	2.032480	0.249546	-1.549867

$PRE_{eq}^{8\text{-}2}$

T_1= 0.01451566, D_1= 0.06904026

$\tilde{\nu}$: 22.0, 64.5, 104.6, 126.1, 249.2, 263.0, 399.7, 417.4, 440.3, 454.0, 551.2, 761.7, 822.3, 839.7, 871.1, 897.0, 922.0, 978.0, 1063.0, 1069.5, 1076.8, 1161.9, 1166.7, 1175.1, 1199.0, 1289.4, 1300.4, 1320.3, 1351.1, 1363.5, 1383.8, 1384.9, 1425.9, 1475.7, 1477.5, 1485.2, 1492.6, 1497.4, 1510.1, 2822.5, 2965.2, 3045.4, 3055.9, 3060.8, 3096.5, 3098.8, 3103.1, 3105.8, 3111.3, 3570.8, 3764.0

B: 2.693056 1.883128 1.796461

C	0.371954	-0.411701	1.202033
N	-0.597886	-1.319126	0.620597
C	-0.842998	-1.029126	-0.787835
C	-1.432060	0.368558	-0.923213
C	-0.488743	1.399627	-0.307637
C	-0.144587	1.019338	1.131203
H	1.342138	-0.470053	0.663984
H	0.560947	-0.704289	2.234924
H	-0.302636	-2.278220	0.739840
H	-1.531384	-1.775765	-1.182508
H	0.088462	-1.078872	-1.374082
H	-1.611919	0.594067	-1.974928
H	-2.394053	0.394413	-0.406356
H	-0.929175	2.396578	-0.344202
H	0.428965	1.430755	-0.902958
H	0.599779	1.702171	1.545468
H	-1.040045	1.093056	1.752553
O	2.674463	-0.113633	-0.821546
H	2.797018	0.758932	-0.410855

$SP_{eq}^{8\text{-}2}$

T_1= 0.02119195, D_1= 0.12054724

$\tilde{\nu}$: -258.0, 39.2, 58.0, 86.0, 241.5, 257.3, 411.6, 433.5, 451.7, 526.2, 554.5, 737.2, 821.0, 840.9, 864.2, 897.9, 923.0, 973.5, 1063.3, 1069.9, 1078.0, 1156.5, 1166.1, 1177.8, 1203.2, 1288.1, 1299.9, 1319.4, 1349.8, 1361.1, 1384.6, 1386.8, 1425.0, 1447.2, 1477.3, 1485.9, 1493.4, 1496.3, 1507.9, 2358.4, 2977.7, 3047.7, 3055.5, 3061.6, 3098.5, 3100.7, 3104.8, 3108.8, 3112.7, 3577.1, 3777.5

B: 2.827250 1.943062 1.792211

C	0.567656	-0.295969	1.095583
N	-0.403905	-1.290644	0.721755
C	-0.907953	-1.109561	-0.635299
C	-1.593451	0.245331	-0.748360
C	-0.623883	1.360346	-0.363217
C	-0.034513	1.099838	1.021429
H	1.454456	-0.332575	0.399760
H	0.949713	-0.513550	2.092966
H	-0.040604	-2.223943	0.854375
H	-1.607808	-1.914869	-0.855169
H	-0.088972	-1.156401	-1.369016
H	-1.958495	0.389049	-1.765767
H	-2.456588	0.259607	-0.078932
H	-1.120866	2.330476	-0.389897
H	0.185542	1.390611	-1.098722
H	0.727833	1.841694	1.265875
H	-0.821662	1.176495	1.775468
O	2.561133	-0.106369	-0.921976
H	2.668591	0.838957	-0.728234

$POST_{eq}^{8}$

T_1= 0.01519514, D_1= 0.07104162

$\tilde{\nu}$: - 34.4, 81.6, 142.4, 146.9, 226.5, 265.2, 327.8, 392.5, 423.8, 450.9, 485.4, 551.2, 721.4, 728.2, 834.3, 847.1, 907.1, 922.7,

C	0.260693	1.022366	0.665937
N	0.415227	-0.281660	1.104067
C	-0.081160	-1.356089	0.248705
C	-1.497856	-1.050455	-0.212585
C	-1.549243	0.312359	-0.897105
C	-1.057686	1.391947	0.066630

934.5, 1051.5, 1076.0, 1084.9, 1110.8, 1178.0, 1193.2, 1258.7, 1283.1, 1307.7, 1353.4, 1370.7, 1385.8, 1387.5, 1461.7, 1472.8, 1486.9, 1495.6, 1511.7, 1525.4, 1616.9, 2968.9, 3008.0, 3049.7, 3058.3, 3103.1, 3106.3, 3111.4, 3113.2, 3182.4, 3597.2, 3706.6, 3933.6

B: 3.340129 1.716875 1.495544

H	2.063925	0.538635	-0.569506
H	0.802117	1.760644	1.243593
H	1.327546	-0.476259	1.489693
H	-0.049366	-2.284860	0.816301
H	0.571686	-1.470404	-0.626185
H	-1.833051	-1.839029	-0.886545
H	-2.165150	-1.048961	0.653109
H	-2.561194	0.536468	-1.234782
H	-0.904052	0.295284	-1.780104
H	-0.976566	2.356828	-0.433472
H	-1.808816	1.511419	0.861995
O	2.843955	-0.029988	-0.652306
H	3.426203	0.411008	-1.273609

$PRE_{eq}^{9\text{-}1}$

T_1= 0.02074069, D_1= 0.11489445

$\tilde{\nu}$: 52.2, 84.5, 177.6, 261.7, 278.0, 416.1, 435.9, 455.4, 555.9, 659.6, 753.1, 827.5, 833.1, 841.5, 894.0, 896.9, 925.1, 981.6, 1061.1, 1074.8, 1076.4, 1152.3, 1176.4, 1178.4, 1194.0, 1295.6, 1300.9, 1324.2, 1352.0, 1367.2, 1386.3, 1391.4, 1426.2, 1475.5, 1486.8, 1490.6, 1499.6, 1502.5, 1516.6, 2981.0, 2984.9, 3040.2, 3052.2, 3062.5, 3105.7, 3106.8, 3108.2, 3108.5, 3113.4, 3239.7, 3552.7

B: 2.739856 2.045640 1.950161

C	-0.076540	0.824588	1.228861
N	-0.573580	-0.536075	0.998801
C	0.467432	-1.414017	0.455973
C	0.940120	-0.877666	-0.888502
C	1.448305	0.554617	-0.741486
C	0.383367	1.433228	-0.089564
H	0.764150	0.822489	1.937606
H	-0.882464	1.415265	1.663380
H	-0.933240	-0.920733	1.863009
H	0.051428	-2.415040	0.346564
H	1.325231	-1.477572	1.141339
H	1.721322	-1.526565	-1.285208
H	0.102429	-0.898775	-1.589739
H	1.735571	0.959593	-1.711719
H	2.346289	0.555051	-0.115365
H	0.766205	2.438364	0.088913
H	-0.477525	1.526147	-0.757897
O	-2.448543	0.052590	-1.013839
H	-1.892098	-0.270918	-0.253477

$SP_{eq}^{9\text{-}1}$

T_1= 0.02074069, D_1= 0.11489445

$\tilde{\nu}$: -867.9, 73.9, 97.9, 210.6, 233.0, 326.2, 351.9, 443.1, 450.8, 550.0, 738.5, 809.1, 837.5, 852.0, 882.5, 902.7, 923.7, 964.0, 1049.2, 1062.2, 1071.1, 1109.5, 1158.2, 1167.4, 1175.4, 1259.8, 1288.6, 1301.2, 1342.2, 1362.3, 1366.7, 1382.6, 1383.7, 1416.8, 1476.1, 1487.6, 1490.5, 1497.0, 1512.6, 1775.6, 2970.2, 2979.0, 3044.9, 3062.9, 3065.0, 3104.2, 3107.8, 3112.6, 3119.5, 3555.4, 3756.0

B: 4.161519 1.702946 1.352847

C	-0.735442	0.184055	0.642493
N	-0.351580	-1.074232	0.042212
C	1.085427	-1.337415	0.148153
C	1.868309	-0.228582	-0.538161
C	1.506555	1.125135	0.066279
C	-0.005955	1.343747	-0.001552
H	-0.636784	0.199257	1.738647
H	-1.870885	0.310076	0.448979
H	-0.874844	-1.829555	0.466280
H	1.291110	-2.300638	-0.317386
H	1.405955	-1.398411	1.199486
H	2.937202	-0.421913	-0.441936
H	1.623811	-0.231807	-1.602768
H	2.028149	1.932010	-0.448894
H	1.829412	1.150580	1.111507
H	-0.289938	2.275262	0.487198
H	-0.316974	1.409081	-1.046770
O	-3.054068	0.045361	-0.348374
H	-2.545979	-0.458847	-1.006109

$PRE_{eq}^{9\text{-}2}$

C	-0.372981	1.052331	-0.385214
N	-0.472630	-0.245989	-1.037355

T_1= 0.01006637, D_1= 0.02572743

$\tilde{\nu}$: 37.5,67.6, 120.4, 142.8, 239.7, 254.9, 279.2, 414.6, 438.6, 454.6, 558.3, 787.9, 826.6, 840.2, 879.4, 898.3, 920.5, 980.4, 1060.7, 1069.0, 1076.8, 1159.8, 1169.2, 1172.9, 1199.0, 1289.3, 1301.3, 1321.1, 1355.4, 1369.7, 1383.3, 1385.1, 1425.9, 1477.7, 1487.1, 1488.2, 1494.7, 1500.5, 1508.5, 2887.1, 2896.4, 3038.1, 3062.1, 3062.2, 3099.9, 3103.9, 3109.3, 3111.0, 3112.3, 3571.5, 3744.2

B: 3.612217 1.642682 1.390033

C	0.023901	-1.320366	-0.193855
C	1.500614	-1.099573	0.107170
C	1.710025	0.264944	0.759967
C	1.087324	1.369188	-0.091480
H	-0.935236	1.080853	0.566983
H	-0.810188	1.806226	-1.038416
H	-1.428805	-0.421746	-1.314546
H	-0.128446	-2.270849	-0.704381
H	-0.513209	-1.375775	0.773834
H	1.871952	-1.897127	0.751899
H	2.053235	-1.145749	-0.833638
H	2.771787	0.454686	0.919715
H	1.235406	0.264936	1.746696
H	1.163670	2.333177	0.412718
H	1.621914	1.445562	-1.040911
O	-3.096501	0.058134	0.605491
H	-2.514964	-0.616491	0.998086

$SP^{9\text{-}2}_{eq}$

T_1= 0.01006637, D_1= 0.02572743

$\tilde{\nu}$: -539.1, 38.4, 70.2, 118.1, 235.1, 265.5, 381.2, 435.4, 443.2, 552.7, 640.7, 776.7, 834.5, 840.6, 879.1, 889.3, 930.7, 986.5, 1020.1, 1065.2, 1075.9, 1127.0, 1154.8, 1170.6, 1175.9, 1259.2, 1287.2, 1306.3, 1323.7, 1351.7, 1368.4, 1371.8, 1386.1, 1426.2, 1475.2, 1486.9, 1489.4, 1496.4, 1511.0, 1771.1, 2952.5, 2956.4, 3042.9, 3063.0, 3069.3, 3102.0, 3106.3, 3111.2, 3119.0, 3572.6, 3784.8

B: 4.439973 1.516272 1.210968

C	-0.752771	0.115792	0.351204
N	-0.214843	-1.106654	-0.191349
C	1.182397	-1.317266	0.182584
C	2.031898	-0.167816	-0.340005
C	1.503639	1.163904	0.186690
C	0.019461	1.318540	-0.153964
H	-0.805263	0.121237	1.452530
H	-1.836499	0.183968	-0.011840
H	-0.786749	-1.895549	0.077661
H	1.512725	-2.264230	-0.242911
H	1.305542	-1.379896	1.275862
H	3.071298	-0.313724	-0.043427
H	1.992094	-0.171597	-1.431359
H	2.074467	1.997180	-0.224010
H	1.628081	1.195369	1.273606
H	-0.385683	2.232458	0.280631
H	-0.102083	1.378058	-1.237150
O	-3.309935	-0.031948	-0.230778
H	-3.592296	0.239968	0.657018

$POST^{9}_{eq}$

T_1= 0.01398710, D_1= 0.06082895

$\tilde{\nu}$: 35.3, 88.1, 136.1, 149.7, 234.3, 260.8, 375.9, 386.6, 450.1, 465.4, 525.0, 594.8, 674.1, 769.4, 839.1, 844.0, 905.7, 916.5, 934.6, 1053.9, 1072.0, 1082.5, 1112.8, 1177.1, 1195.0, 1234.2, 1286.1, 1309.2, 1350.8, 1368.0, 1379.8, 1392.5, 1449.0, 1470.5, 1489.7, 1491.2, 1501.0, 1513.3, 1637.8, 2974.8, 3008.3, 3051.3, 3068.0, 3105.7, 3108.6, 3109.9, 3117.3, 3197.2, 3587.9, 3639.4, 3930.8

B: 2.773657 1.930941 1.857470

C	0.304852	1.198334	0.906453
N	-0.425873	0.047333	1.225551
C	0.187750	-1.235993	0.861293
C	0.630818	-1.213327	-0.594444
C	1.570966	-0.037814	-0.845882
C	0.873277	1.270830	-0.470724
H	-0.018916	2.099855	1.407514
H	-3.302230	0.477642	-0.679158
H	-0.760762	0.050822	2.178060
H	-0.547577	-2.022133	1.028400
H	1.057339	-1.432732	1.500658
H	1.118408	-2.159798	-0.829137
H	-0.248874	-1.124735	-1.235925
H	1.881717	-0.014674	-1.890248
H	2.471172	-0.155136	-0.236248
H	1.558888	2.114914	-0.539028
H	0.067495	1.450165	-1.198142
O	-2.472435	0.047035	-0.891489

H	-1.922046	0.116018	-0.093869

PIP$\dot{\mathrm{C}}^{2}$

T_1= 0.01546620, D_1= 0.06541086

$\tilde{\nu}$: 225.7, 250.6, 385.8, 437.5, 470.4, 514.8, 664.8, 709.2, 834.7, 846.5, 906.5, 921.2, 932.4, 1050.9, 1075.6, 1083.3, 1107.6, 1176.0, 1190.0, 1249.7, 1281.5, 1306.3, 1351.8, 1367.8, 1383.7, 1385.6, 1455.8, 1470.8, 1485.8, 1493.4, 1504.4, 1516.8, 2955.2, 2987.8, 3051.5, 3058.1, 3102.9, 3104.7, 3107.1, 3110.2, 3200.6, 3612.8

B: 4.759784 4.575280 2.582603

C	0.045985	1.434030	0.153552
N	-1.151463	0.809575	-0.174262
C	-1.297427	-0.595290	0.189626
C	-0.086883	-1.385542	-0.284854
C	1.195420	-0.774716	0.273452
C	1.301676	0.689127	-0.156205
H	0.034614	2.512875	0.086339
H	-1.968971	1.362092	0.030685
H	-2.210747	-0.976190	-0.265778
H	-1.387035	-0.705684	1.279169
H	-0.191718	-2.425725	0.024970
H	-0.055717	-1.365900	-1.377244
H	2.066897	-1.335560	-0.065128
H	1.172832	-0.828722	1.365278
H	2.147892	1.176537	0.327464
H	1.499568	0.713601	-1.239349

PRE$_{eq}^{10}$ (see PRE$_{eq}^{9\text{-}1}$)

SP$_{eq}^{10\text{-}1}$

T_1= 0.02105550, D_1= 0.11545777

$\tilde{\nu}$: -964.4, 116.9, 150.0, 245.4, 270.2, 402.1, 438.0, 454.2, 529.0, 550.6, 740.5, 797.0, 835.6, 868.0, 894.7, 908.0, 943.0, 977.9, 1018.4, 1063.6, 1076.0, 1092.0, 1157.0, 1164.7, 1192.2, 1220.3, 1251.4, 1303.9, 1318.2, 1344.4, 1350.3, 1373.8, 1388.6, 1421.2, 1479.3, 1480.3, 1491.6, 1495.7, 1512.4, 1577.1, 2955.3, 2967.2, 3023.5, 3072.1, 3106.3, 3107.6, 3108.5, 3115.9, 3119.0, 3565.6, 3663.9

B: 2.884137 2.273691 2.034933

C	-0.300621	-0.900578	1.100485
N	0.309108	-1.359403	-0.146239
C	1.497804	-0.575702	-0.483328
C	1.105292	0.877277	-0.718495
C	0.386340	1.447207	0.503619
C	-0.748325	0.537238	0.935633
H	0.407085	-0.957091	1.943768
H	-1.152091	-1.539335	1.332481
H	0.543686	-2.340504	-0.078496
H	1.949648	-0.998481	-1.379955
H	2.246663	-0.620239	0.322657
H	1.994410	1.465857	-0.945683
H	0.440162	0.929174	-1.582128
H	0.015729	2.451184	0.298655
H	1.097057	1.531879	1.334350
H	-1.289403	0.903497	1.807389
H	-1.548851	0.519851	0.064346
O	-2.130675	0.062244	-1.110048
H	-1.465389	-0.640567	-1.240813

SP$_{eq}^{10\text{-}2}$

T_1= 0.02018874, D_1= 0.11089443

$\tilde{\nu}$: -549.7, 59.8, 73.5, 128.6, 249.3, 265.1, 404.4, 436.5, 442.6, 536.2, 697.2, 765.9, 839.2, 854.6, 873.3, 898.0, 922.9, 963.8, 1056.7, 1069.3, 1073.8, 1119.2, 1164.3, 1172.5, 1198.3, 1217.3, 1294.3, 1312.3, 1330.1, 1343.2, 1351.3, 1375.8, 1387.7, 1422.7, 1478.1, 1484.9, 1488.9, 1495.5, 1510.4, 1633.6, 2922.0, 2947.7, 3027.0, 3068.3, 3098.4, 3102.4, 3105.7, 3108.0, 3116.9, 3575.8, 3785.3

B: 3.138192 1.896329 1.662541

C	0.300080	-1.362458	0.575890
N	1.033878	-1.030294	-0.633761
C	1.732041	0.244342	-0.527130
C	0.717805	1.362395	-0.326110
C	-0.116503	1.100181	0.925266
C	-0.730028	-0.288343	0.871741
H	0.962842	-1.438660	1.457035
H	-0.184090	-2.329394	0.443224
H	1.675802	-1.772711	-0.872991
H	2.300285	0.405829	-1.442563
H	2.444205	0.253726	0.315016
H	1.230566	2.321663	-0.249123
H	0.058881	1.397588	-1.195690
H	-0.894315	1.855847	1.037688

H	0.525876	1.165207	1.811048
H	-1.304589	-0.526584	1.767110
H	-1.473857	-0.295534	-0.011172
O	-2.591062	-0.029681	-0.936187
H	-3.270622	0.075831	-0.251692

POST$^{10}_{eq}$

T_1= 0.00895900, D_1= 0.01776535

$\tilde{\nu}$: 54.5, 72.8, 160.3, 188.9, 198.9, 242.0, 365.3, 387.6, 408.9, 460.5, 492.9, 652.2, 687.9, 803.4, 842.4, 881.3, 900.3, 927.0, 950.2, 1032.4, 1071.2, 1095.5, 1117.5, 1168.6, 1192.6, 1206.5, 1275.9, 1301.6, 1342.9, 1363.8, 1366.9, 1401.9, 1422.7, 1471.6, 1480.2, 1486.9, 1492.8, 1510.1, 1659.8, 2898.2, 2961.9, 2970.1, 3071.7, 3101.4, 3101.6, 3111.3, 3120.0, 3207.3, 3558.9, 3578.8, 3913.0

B: 2.715812 1.948415 1.904725

C	0.048805	-0.392346	1.452058
N	0.362480	0.900040	0.833397
C	-0.743340	1.396487	0.014118
C	-0.976452	0.447509	-1.152963
C	-1.304713	-0.957670	-0.638469
C	-0.335891	-1.386025	0.409688
H	-0.781863	-0.286764	2.175732
H	0.920572	-0.735052	2.009252
H	0.596116	1.573400	1.551212
H	-0.482280	2.389594	-0.349097
H	-1.670282	1.485105	0.601731
H	-1.788188	0.820906	-1.777273
H	-0.070383	0.413457	-1.760348
H	-1.335278	-1.675607	-1.457892
H	-2.318282	-0.939560	-0.207622
H	-0.197959	-2.436370	0.622904
H	2.221855	-1.009468	-0.909459
O	2.545624	-0.110309	-0.820721
H	1.873173	0.334819	-0.273747

PRE$^{11\text{-}1}_{eq}$

T_1= 0.00983672, D_1= 0.02510933

$\tilde{\nu}$: -500.5, 38.4, 60.8, 100.0, 246.4, 264.7, 407.7, 431.2, 442.4, 544.8, 654.9, 770.2, 832.8, 876.7, 880.5, 907.6, 927.5, 989.5, 1039.9, 1066.9, 1080.6, 1125.0, 1160.2, 1172.8, 1186.8, 1252.9, 1295.6, 1305.9, 1327.8, 1346.0, 1360.4, 1374.7, 1379.9, 1424.6, 1476.2, 1482.7, 1492.8, 1501.2, 1512.4, 1602.2, 2956.2, 2969.9, 3052.2, 3063.9, 3088.2, 3103.7, 3109.0, 3110.9, 3116.6, 3567.8, 3787.7

B: 2.827250 1.943062 1.792211

C	0.083107	-1.264589	0.247939
N	-1.359066	-1.175778	0.049473
C	-1.728928	-0.012795	-0.747355
C	-1.313297	1.258707	-0.018756
C	0.186708	1.234973	0.269514
C	0.562778	-0.042252	1.018829
H	0.631274	-1.312904	-0.706663
H	0.304570	-2.175457	0.803243
H	-1.706567	-2.021382	-0.381398
H	-2.807118	-0.025455	-0.905441
H	-1.245342	-0.024423	-1.738170
H	-1.576971	2.132297	-0.616244
H	-1.864418	1.318148	0.922269
H	0.484780	2.120013	0.833662
H	0.719204	1.262428	-0.688446
H	1.641682	-0.108442	1.178305
H	0.086672	-0.038024	2.001987
O	3.147800	-0.098195	-0.637803
H	2.921083	0.844937	-0.568017

SP$^{11\text{-}1}_{eq}$

T_1= 0.02004102, D_1= 0.11004883

$\tilde{\nu}$: -500.5, 38.4, 60.8, 100.0, 246.4, 264.7, 407.7, 431.2, 442.4, 544.8, 654.9, 770.2, 832.8, 876.7, 880.5, 907.6, 927.5, 989.5, 1039.9, 1066.9, 1080.6, 1125.0, 1160.2, 1172.8, 1186.8, 1252.9, 1295.6, 1305.9, 1327.8, 1346.0, 1360.4, 1374.7, 1379.9, 1424.6, 1476.2, 1482.7, 1492.8, 1501.2, 1512.4, 1602.2, 2956.2, 2969.9, 3052.2, 3063.9, 3088.2, 3103.7, 3109.0, 3110.9, 3116.6, 3567.8,

C	-0.165400	-1.196008	0.089687
N	1.246902	-1.239367	-0.276125
C	1.997466	-0.151949	0.340924
C	1.469758	1.183503	-0.165996
C	-0.029611	1.309612	0.123283
C	-0.759510	0.099378	-0.431194
H	-0.313734	-1.236348	1.179967
H	-0.672738	-2.054922	-0.347653
H	1.646191	-2.133438	-0.025010
H	3.050475	-0.266062	0.084715

3787.7

B: 2.827250 1.943062 1.792211

H	1.915473	-0.170622	1.439842
H	2.014464	2.003488	0.303602
H	1.640968	1.240295	-1.242911
H	-0.429033	2.232180	-0.296998
H	-0.183246	1.352711	1.205299
H	-1.854713	0.143673	-0.081845
H	-0.770571	0.103078	-1.522660
O	-3.281412	0.016051	0.266844
H	-3.596774	-0.134083	-0.638449

PRE$_{eq}^{11\text{-}2}$

T_1= 0.01450744, D_1= 0.06895543

$\tilde{\nu}$: 21.9, 64.5, 104.6, 126.3, 249.2, 263.0, 399.7, 417.4, 440.3, 454.0, 551.2, 761.7, 822.3, 839.7, 871.1, 897.0, 922.0, 978.0, 1063.0, 1069.5, 1076.8, 1161.9, 1166.7, 1175.1, 1199.0, 1289.4, 1300.4, 1320.3, 1351.1, 1363.5, 1383.8, 1384.9, 1425.9, 1475.7, 1477.5, 1485.2, 1492.6, 1497.4, 1510.1, 2822.5, 2965.2, 3045.4, 3055.9, 3060.8, 3096.5, 3098.8, 3103.1, 3105.8, 3111.3, 3570.8, 3764.0

B: 2.693092 1.883129 1.796443

C	0.083107	-1.264589	0.247939
N	-1.359066	-1.175778	0.049473
C	-1.728928	-0.012795	-0.747355
C	-1.313297	1.258707	-0.018756
C	0.186708	1.234973	0.269514
C	0.562778	-0.042252	1.018829
H	0.631274	-1.312904	-0.706663
H	0.304570	-2.175457	0.803243
H	-1.706567	-2.021382	-0.381398
H	-2.807118	-0.025455	-0.905441
H	-1.245342	-0.024423	-1.738170
H	-1.576971	2.132297	-0.616244
H	-1.864418	1.318148	0.922269
H	0.484780	2.120013	0.833662
H	0.719204	1.262428	-0.688446
H	1.641682	-0.108442	1.178305
H	0.086672	-0.038024	2.001987
O	3.147800	-0.098195	-0.637803
H	2.921083	0.844937	-0.568017

SP$_{eq}^{11\text{-}2}$

POST$_{eq}^{11}$

T_1= 0.01158307, D_1= 0.03761254

$\tilde{\nu}$: 33.2, 39.4, 100.3, 111.0, 188.0, 226.4, 232.0, 315.8, 346.5, 429.3, 448.1, 486.5, 636.2, 781.5, 847.0, 883.7, 894.7, 929.4, 956.8, 1012.9, 1057.5, 1116.4, 1150.4, 1158.8, 1180.3, 1203.9, 1299.7, 1304.5, 1328.1, 1334.8, 1357.0, 1388.2, 1422.6, 1474.6, 1482.1, 1487.8, 1502.6, 1510.1, 1616.4, 2949.2, 2955.5, 3016.0, 3066.3, 3104.3, 3112.7, 3116.7, 3117.4, 3199.8, 3553.0, 3790.4, 3938.1

B: 3.570066 1.514627 1.287758

C	0.084955	-1.226762	0.354899
N	-1.361011	-1.217255	0.085184
C	-1.731097	-0.101904	-0.779768
C	-1.436371	1.216340	-0.076540
C	0.056984	1.295579	0.295719
C	0.453410	0.056646	1.024539
H	0.661648	-1.322032	-0.580661
H	0.320107	-2.083934	0.983059
H	-1.631550	-2.093858	-0.341785
H	-2.794245	-0.178193	-1.006730
H	-1.181255	-0.123715	-1.734696
H	-1.706580	2.053754	-0.721082
H	-2.041862	1.273940	0.829365
H	0.265112	2.191811	0.878128
H	0.631708	1.367762	-0.636103
H	2.550996	0.024760	0.109771
H	0.599548	0.075757	2.095556
O	3.159920	-0.045591	-0.635008
H	4.006794	0.260079	-0.304144

PIP$\dot{C}^3$
T_1= 0.01158722, D_1= 0.03427274

C	0.080678	-1.360242	0.251426
N	1.208510	-0.638732	-0.324674
C	1.253492	0.751762	0.126371

$\tilde{\nu}$: 170.5, 223.5, 340.2, 383.4, 441.7, 480.6, 631.5, 775.8, 844.2, 872.7, 900.5, 929.3, 944.9, 1028.0, 1069.4, 1099.3, 1117.0, 1171.4, 1196.0, 1202.8, 1273.9, 1298.4, 1341.3, 1359.8, 1364.9, 1399.6, 1423.1, 1471.4, 1481.8, 1485.3, 1492.4, 1509.1, 2867.4, 2943.6, 2956.1, 3068.8, 3097.0, 3098.3, 3103.2, 3116.4, 3207.8, 3562.6

B: 4.801706 4.507795 2.582316

C	-0.034145	1.439805	-0.167637
C	-1.299811	0.727183	0.161043
C	-1.220548	-0.747406	-0.246535
H	0.090318	-1.328586	1.354252
H	0.148003	-2.405458	-0.049290
H	2.076223	-1.101190	-0.089303
H	2.081801	1.258210	-0.368710
H	1.446977	0.807519	1.217362
H	-0.033856	2.511469	-0.303826
H	-2.155638	1.216279	-0.304496
H	-1.474150	0.771893	1.248055
H	-2.072133	-1.297730	0.154941
H	-1.245112	-0.827887	-1.334275

PRE$_{eq}^{12\text{-}1}$ (see PRE$_{eq}^{8\text{-}2}$)

SP$_{eq}^{12\text{-}1}$

T_1= 0.01979598, D_1= 0.10846800

$\tilde{\nu}$: -502.0, 49.0, 60.8, 137.8, 250.3, 256.4, 407.8, 432.5, 455.4, 542.9, 682.1, 783.9, 819.6, 839.4, 901.0, 908.7, 919.4, 975.7, 1059.1, 1070.0, 1075.5, 1145.2, 1168.1, 1169.9, 1195.0, 1245.9, 1279.5, 1321.5, 1346.6, 1353.3, 1365.1, 1375.4, 1388.4, 1434.5, 1464.3, 1478.0, 1484.9, 1505.4, 1516.2, 1650.4, 2975.5, 2976.4, 3049.1, 3050.9, 3097.4, 3104.0, 3105.0, 3107.4, 3108.4, 3566.5, 3780.9

B: 3.018263 1.925041 1.737308

C	-0.887691	-1.207349	-0.491675
N	-1.706650	0.000009	-0.522012
C	-0.887680	1.207359	-0.491664
C	-0.115846	1.262510	0.820370
C	0.711402	-0.000010	0.997712
C	-0.115858	-1.262520	0.820358
H	-0.165366	-1.235563	-1.321745
H	-1.539837	-2.075395	-0.583116
H	-2.294563	0.000016	-1.344211
H	-1.539817	2.075412	-0.583096
H	-0.165355	1.235574	-1.321734
H	0.523015	2.145827	0.843045
H	-0.828619	1.340969	1.646360
H	1.278710	-0.000017	1.928663
H	1.485214	-0.000009	0.152129
H	0.522993	-2.145844	0.843026
H	-0.828633	-1.340979	1.646347
O	2.500011	0.000001	-0.974404
H	3.272762	-0.000006	-0.386955

POST$_{eq}^{12\text{-}1}$

T_1= 0.01169635, D_1= 0.03877471

$\tilde{\nu}$: 28.6, 71.7, 104.3, 111.8, 211.6, 236.6, 243.8, 348.9, 393.6, 426.6, 459.8, 495.8, 654.5, 799.4, 806.4, 844.6, 907.4, 921.2, 1006.8, 1048.8, 1065.6, 1094.8, 1099.2, 1180.7, 1188.6, 1204.4, 1257.4, 1326.5, 1339.3, 1353.1, 1377.2, 1397.8, 1428.0, 1456.4, 1465.1, 1482.9, 1509.9, 1519.2, 1616.9, 2981.2, 2981.3, 2992.1, 2992.8, 3094.1, 3094.5, 3108.7, 3109.4, 3182.8, 3569.3, 3769.7, 3931.5

B: 2.681154 1.815215 1.753122

C	0.672729	1.204901	-0.639219
N	1.441729	-0.000028	-0.931446
C	0.672704	-1.204930	-0.639178
C	0.409483	-1.278967	0.864509
C	-0.167408	0.000029	1.371754
C	0.409511	1.278994	0.864466
H	-0.290292	1.215435	-1.170133
H	1.245258	2.074535	-0.959455
H	1.728625	-0.000047	-1.900669
H	1.245214	-2.074587	-0.959384
H	-0.290317	-1.215463	-1.170093
H	-0.242706	-2.122523	1.094439
H	1.369401	-1.467463	1.365550
H	-0.700662	0.000050	2.313815
H	-2.074924	0.000011	0.070953
H	-0.242660	2.122572	1.094368
H	1.369433	1.467486	1.365499
O	-2.702326	-0.000002	-0.662694
H	-3.571979	0.000048	-0.257211

$PRE_{eq}^{12\text{-}2}$

T_1= 0.01450641, D_1= 0.06894671

$\tilde{\nu}$: 21.4, 65.0, 104.6, 128.9, 249.3, 263.1, 400.0, 417.4, 440.3, 454.1, 551.2, 761.6, 822.3, 839.7, 871.2, 897.1, 922.0, 978.0, 1063.0, 1069.5, 1076.9, 1162.0, 1166.7, 1175.1, 1199.0, 1289.4, 1300.5, 1320.4, 1351.1, 1363.6, 1383.8, 1385.0, 1425.9, 1475.7, 1477.5, 1485.3, 1492.6, 1497.4, 1510.1, 2822.5, 2965.1, 3045.3, 3055.9, 3060.8, 3096.4, 3098.8, 3103.1, 3105.8, 3111.3, 3570.7, 3764.1

B: 2.693625 1.882852 1.795948

C	-0.372194	0.414593	1.200781
N	0.599137	1.319817	0.618405
C	0.844993	1.027033	-0.789317
C	1.432469	-0.371601	-0.921747
C	0.487383	-1.400414	-0.305100
C	0.142656	-1.017200	1.132824
H	-1.341970	0.473135	0.662010
H	-0.561488	0.709231	2.233038
H	0.304976	2.279485	0.735753
H	1.534605	1.772140	-1.184737
H	-0.085945	1.076866	-1.376408
H	1.612917	-0.599197	-1.972909
H	2.394000	-0.397706	-0.404050
H	0.926554	-2.397995	-0.339618
H	-0.429931	-1.431397	-0.901056
H	-0.602855	-1.698394	1.547742
H	1.037562	-1.090944	1.754966
O	-2.674643	0.113699	-0.822309
H	-2.797089	-0.758003	-0.409740

$SP_{eq}^{12\text{-}2}$

T_1= 0.02016796, D_1= 0.11058014

$\tilde{\nu}$: -570.7, 74.3, 96.7, 112.6, 238.5, 262.7, 403.6, 429.4, 457.5, 535.4, 706.6, 776.7, 817.0, 838.3, 892.1, 900.6, 921.3, 974.3, 1056.8, 1069.0, 1081.1, 1143.9, 1155.5, 1173.1, 1197.5, 1243.9, 1269.5, 1314.6, 1343.5, 1354.7, 1368.4, 1375.6, 1403.8, 1434.9, 1464.5, 1477.5, 1485.2, 1503.8, 1513.1, 1576.8, 2936.0, 2942.5, 3052.1, 3053.9, 3100.9, 3105.9, 3106.9, 3110.8, 3111.8, 3570.5, 3780.6

B: 2.987268 1.971296 1.783994

C	0.855508	1.207946	-0.488566
N	1.672812	0.003170	-0.528509
C	0.863822	-1.206270	-0.483014
C	0.098648	-1.261777	0.832609
C	-0.722795	0.000078	1.033612
C	0.092427	1.265143	0.828089
H	0.129358	1.241259	-1.318871
H	1.504377	2.077189	-0.589235
H	2.265982	0.003255	-1.346524
H	1.518028	-2.072160	-0.578241
H	0.140109	-1.249561	-1.315585
H	-0.542644	-2.143047	0.857612
H	0.821574	-1.349833	1.648462
H	-1.251655	0.000003	1.986970
H	-1.574482	-0.001062	0.258839
H	-0.553660	2.142907	0.849965
H	0.815593	1.359895	1.643007
O	-2.530107	0.001077	-0.891998
H	-1.867067	-0.070378	-1.597229

$POST_{eq}^{12\text{-}2}$

T_1= 0.01172525, D_1= 0.03848531

$\tilde{\nu}$: 18.2, 57.5, 103.3, 105.0, 195.5, 226.0, 242.4, 351.6, 395.4, 432.2, 460.3, 498.7, 649.1, 786.8, 806.9, 846.0, 908.9, 922.1, 1005.8, 1048.3, 1065.3, 1090.8, 1103.9, 1180.5, 1192.0, 1205.5, 1256.6, 1322.0, 1334.1, 1351.8, 1377.8, 1400.6, 1422.4, 1458.2, 1467.0, 1481.7, 1504.8, 1513.6, 1619.9, 2952.5, 2953.8, 2991.0, 2992.1, 3099.4, 3099.7, 3110.5, 3111.2, 3188.4, 3570.7, 3782.4, 3932.9

B: 2.758653 1.710910 1.626779

C	0.760213	1.204760	-0.613571
N	1.544099	-0.000892	-0.842644
C	0.759215	-1.205686	-0.612447
C	0.363726	-1.278807	0.861510
C	-0.255173	0.000859	1.312587
C	0.364805	1.279582	0.860324
H	-0.155984	1.220972	-1.226229
H	1.357670	2.073324	-0.887064
H	1.925946	-0.001490	-1.778181
H	1.355965	-2.074994	-0.885117
H	-0.156985	-1.221722	-1.225101
H	-0.307960	-2.120661	1.032568
H	1.276072	-1.469430	1.443535
H	-0.887105	0.001533	2.190840
H	-2.179831	0.000551	0.025939

H	-0.306161	2.122164	1.030620
H	1.277325	1.469968	1.442153
O	-2.928645	0.000176	-0.582313
H	-2.535206	0.000378	-1.457371

$PRE_{eq}^{13\text{-}1}$

T_1= 0.01012843, D_1= 0.02481417

$\tilde{\nu}$: 9.8, 58.4, 103.6, 120.9, 246.8, 260.5, 280.6, 413.7, 441.0, 453.1, 553.7, 772.5, 826.7, 840.2, 883.7, 898.6, 920.8, 984.2, 1062.7, 1070.8, 1074.8, 1157.7, 1171.6, 1176.1, 1196.9, 1291.0, 1300.9, 1321.9, 1353.2, 1364.7, 1378.7, 1385.2, 1427.4, 1473.5, 1484.9, 1486.2, 1494.4, 1503.5, 1515.7, 2953.2, 2957.3, 3004.0, 3058.3, 3061.2, 3079.2, 3102.2, 3105.3, 3109.2, 3111.9, 3566.0, 3761.8

B: 4.473113 1.485259 1.195930

C	-0.441617	-1.417363	-0.016317
N	-1.744476	-0.777080	-0.157764
C	-1.632926	0.569259	-0.705519
C	-0.822109	1.441301	0.244280
C	0.552294	0.819602	0.484432
C	0.411848	-0.623855	0.963526
H	0.095207	-1.477044	-0.977130
H	-0.590127	-2.434736	0.344577
H	-2.351392	-1.341321	-0.736360
H	-2.634780	0.977830	-0.835934
H	-1.139715	0.570853	-1.691420
H	-0.724009	2.446588	-0.167162
H	-1.358821	1.517837	1.192543
H	1.121243	1.414973	1.202030
H	1.106340	0.829397	-0.462697
H	1.389765	-1.096539	1.061962
H	-0.073152	-0.638452	1.942146
O	3.204257	-0.061702	-0.713297
H	3.331775	0.430119	0.115768

$SP_{eq}^{13\text{-}1}$

T_1= 0.01995502, D_1= 0.10962017

$\tilde{\nu}$: -469.2, -25.7, 61.3, 61.7, 252.8, 263.7, 409.8, 428.6, 451.6, 546.6, 642.0, 775.3, 828.8, 844.6, 898.0, 904.9, 948.5, 982.1, 1057.5, 1066.4, 1066.5, 1138.5, 1169.7, 1171.1, 1192.8, 1255.1, 1290.7, 1308.6, 1326.2, 1327.9, 1351.2, 1376.2, 1383.7, 1425.2, 1476.4, 1483.1, 1491.4, 1502.2, 1513.6, 1661.7, 2954.0, 2954.7, 3057.8, 3071.2, 3073.6, 3103.3, 3104.2, 3119.4, 3122.1, 3569.7, 3790.9

B: 4.473113 1.485259 1.195930

C	-1.393587	1.208524	0.168148
N	-1.968622	0.000006	-0.406782
C	-1.393599	-1.208517	0.168149
C	0.100725	-1.257985	-0.144785
C	0.769730	-0.000006	0.380979
C	0.100737	1.257979	-0.144787
H	-1.526998	1.248271	1.261569
H	-1.896632	2.075287	-0.259702
H	-2.973419	0.000011	-0.298375
H	-1.896652	-2.075275	-0.259699
H	-1.527010	-1.248261	1.261571
H	0.547461	-2.149165	0.296030
H	0.229006	-1.315035	-1.227026
H	1.864433	-0.000012	0.041852
H	0.789557	-0.000005	1.474746
H	0.547481	2.149155	0.296026
H	0.229018	1.315026	-1.227028
O	3.334874	-0.000008	-0.223202
H	3.611083	0.000059	0.706893

$POST_{eq}^{13\text{-}1}$

T_1= 0.01200801, D_1= 0.04265116

$\tilde{\nu}$: 28.0, 43.6, 55.1, 107.9, 184.0, 230.2, 242.5, 335.2, 347.6, 427.4, 451.3, 490.8, 605.8, 774.1, 820.6, 852.2, 904.7, 915.4, 1007.5, 1031.1, 1055.3, 1108.1, 1127.3, 1171.8, 1187.9, 1207.2, 1279.4, 1315.7, 1320.3, 1342.5, 1348.0, 1396.3, 1414.0, 1471.5, 1478.3, 1482.8, 1501.7, 1513.3, 1619.5, 2962.9, 2963.4, 3034.6, 3036.1, 3102.7, 3104.6, 3117.8, 3120.2, 3196.3, 3572.8, 3785.9, 3938.2

C	-1.483507	1.167861	-0.077906
N	-1.698455	-0.076603	-0.802176
C	-1.360167	-1.249297	-0.007896
C	0.141367	-1.218486	0.318330
C	0.479138	0.084942	0.960305
C	0.012705	1.307690	0.244920
H	-2.050665	1.202005	0.865232
H	-1.812104	1.999277	-0.701049
H	-2.652616	-0.134691	-1.129679
H	-1.600470	-2.144048	-0.581504
H	-1.924573	-1.286347	0.936730

B: 3.699456 1.462639 1.243915

H	0.407338	-2.062131	0.953357
H	0.682109	-1.321449	-0.628624
H	2.559240	0.033195	0.066528
H	0.705276	0.127008	2.017022
H	0.189064	2.210132	0.828661
H	0.542655	1.406586	-0.708585
O	3.265659	-0.067234	-0.582657
H	3.981433	0.488295	-0.268117

$\mathrm{PRE}_{eq}^{13\text{-}2}$

T_1= 0.00997335, D_1= 0.10983463

$\tilde{\nu}$: 51.3, 63.7, 104.2, 163.2, 250.0, 254.3, 270.5, 413.5, 438.8, 453.0, 551.4, 776.6, 821.5, 838.9, 879.8, 898.4, 920.5, 982.8, 1058.5, 1070.5, 1075.9, 1159.2, 1171.1, 1175.5, 1196.2, 1291.6, 1297.8, 1320.7, 1351.0, 1365.6, 1385.6, 1385.6, 1426.0, 1474.3, 1486.2, 1486.4, 1497.0, 1504.1, 1516.2, 2951.9, 2953.2, 3039.1, 3051.6, 3052.2, 3082.1, 3104.0, 3105.2, 3107.2, 3109.3, 3569.7, 3753.7

B: 3.633106 1.570012 1.336046

C	1.343779	-1.207026	-0.052386
N	1.636935	0.000004	-0.815897
C	1.343775	1.207029	-0.052381
C	-0.145423	1.259162	0.260798
C	-0.577200	-0.000004	1.010203
C	-0.145419	-1.259166	0.260793
H	1.904957	-1.237154	0.895928
H	1.641022	-2.074828	-0.640523
H	2.601787	0.000006	-1.116790
H	1.641014	2.074835	-0.640516
H	1.904953	1.237156	0.895933
H	-0.375167	2.149959	0.846116
H	-0.689580	1.343764	-0.683911
H	-1.656204	-0.000006	1.180812
H	-0.105818	-0.000005	1.997936
H	-0.375158	-2.149966	0.846108
H	-0.689577	-1.343767	-0.683915
O	-3.219277	-0.000003	-0.576474
H	-2.423623	0.000041	-1.136260

$\mathrm{SP}_{eq}^{13\text{-}2}$

T_1= 0.02001287, D_1= 0.03568433

$\tilde{\nu}$: -628.6, 32.0, 73.0, 80.3, 233.7, 253.3, 387.4, 420.1, 452.6, 535.5, 686.9, 781.3, 827.8, 845.9, 899.0, 899.7, 930.0, 979.2, 1050.1, 1066.0, 1068.3, 1142.1, 1150.2, 1170.3, 1192.9, 1257.1, 1289.8, 1309.1, 1329.9, 1352.1, 1371.6, 1386.7, 1409.1, 1420.2, 1474.9, 1482.8, 1487.4, 1502.5, 1513.2, 1642.9, 2957.7, 2958.3, 3056.5, 3061.4, 3068.6, 3104.5, 3105.3, 3117.0, 3119.1, 3568.7, 3786.1

B: 4.324771 1.563450 1.258290

C	1.376027	-1.208564	0.109113
N	1.877591	-0.000093	-0.532140
C	1.376158	1.208489	0.109008
C	-0.144366	1.257614	-0.023151
C	-0.747715	0.000098	0.576627
C	-0.144503	-1.257533	-0.023047
H	1.638571	-1.246755	1.178602
H	1.825154	-2.075649	-0.374371
H	2.888200	-0.000148	-0.543565
H	1.825378	2.075483	-0.374552
H	1.638712	1.246744	1.178493
H	-0.537810	2.148136	0.466569
H	-0.395363	1.317668	-1.084816
H	-1.884438	0.000162	0.406652
H	-0.646402	0.000134	1.665830
H	-0.538048	-2.147972	0.466743
H	-0.395494	-1.317645	-1.084711
O	-3.240007	0.000037	-0.193975
H	-2.935147	-0.000423	-1.115402

$\mathrm{POST}_{eq}^{13\text{-}2}$

T_1= 0.01150825, D_1= 0.03582035

$\tilde{\nu}$: 46.1, 77.6, 148.3, 156.5, 216.9, 243.7, 371.3, 404.5, 438.1, 459.4, 511.8, 640.0, 739.6, 815.0, 838.6, 851.2, 907.7, 919.6, 1008.4, 1050.5, 1067.0, 1094.4, 1103.3, 1178.4, 1187.5, 1205.2,

C	-0.443636	-1.212123	0.873898
N	0.311982	-0.000025	1.202627
C	-0.443611	1.212099	0.873938
C	-0.676897	1.274936	-0.634994
C	-1.268710	0.000029	-1.129422
C	-0.676924	-1.274906	-0.635035
H	-1.414301	-1.222455	1.388697

1268.9, 1324.7, 1341.5, 1348.9, 1380.7, 1398.0, 1419.5, 1465.6, 1474.4, 1477.1, 1499.5, 1512.0, 1651.4, 2986.9, 2987.8, 2999.3, 2999.5, 3102.4, 3102.7, 3107.9, 3108.5, 3206.1, 3482.6, 3552.8, 3930.2

B: 2.651266 2.018675 1.956058

H	0.128496	-2.077928	1.205770
H	0.541880	-0.000044	2.188058
H	0.128539	2.077881	1.205838
H	-1.414276	1.222434	1.388736
H	-1.314861	2.125684	-0.874655
H	0.294068	1.447321	-1.119851
H	3.336285	-0.000056	-0.321510
H	-1.868087	0.000050	-2.028257
H	-1.314905	-2.125633	-0.874724
H	0.294038	-1.447294	-1.119899
O	2.455759	0.000003	-0.700036
H	1.831839	-0.000013	0.053385

PIPĊ^{4}

T_1= 0.01174001, D_1= 0.03582035

$\tilde{\nu}$: 186.8, 235.2, 359.2, 400.3, 456.8, 475.4, 621.5, 783.8, 808.6, 848.0, 909.1, 921.4, 1007.4, 1048.9, 1063.5, 1084.9, 1106.3, 1181.1, 1189.4, 1204.6, 1257.2, 1322.5, 1333.9, 1347.1, 1372.1, 1400.3, 1420.3, 1460.2, 1469.2, 1482.9, 1501.1, 1511.5, 2962.9, 2964.1, 2985.5, 2986.6, 3099.6, 3100.0, 3103.9, 3104.7, 3204.6, 3568.2

B: 4.726136 4.595880 2.592119

C	0.032376	-0.756088	1.206027
N	-0.666044	-1.183023	0.000000
C	0.032376	-0.756088	-1.206027
C	0.032376	0.770965	-1.275732
C	0.534491	1.353904	-0.000000
C	0.032376	0.770965	1.275732
H	1.075361	-1.108655	1.219522
H	-0.476591	-1.173859	2.074134
H	-0.781580	-2.187055	0.000000
H	-0.476591	-1.173859	-2.074134
H	1.075361	-1.108655	-1.219522
H	0.626670	1.109947	-2.124600
H	-1.002757	1.094780	-1.454394
H	1.014558	2.321835	-0.000000
H	0.626670	1.109947	2.124600
H	-1.002757	1.094780	1.454394

Table S4. QCC results for the piperidine (*ax*) reaction with OH radicals

Electronic energies of reactants, intermediates and products (/Hartree), and relative energies including Zero Point Energies, Δ (/kJ mol^{-1}), of stationary points on the potential energy surface of the piperidine + OH reaction, Figure 2 in the main text.

Species	M06-2X/aTZ		CCSD(T*)-F12a/aTZ	
	E_{Elec}	E_{ZPE}	E_{Elec}	$\Delta E_{v=0}$
Piperidine, *ax* ($^1C_4$)	-251.87515	0.16002	-251.56307	
OH	-75.73381	0.00859	-75.67069	
Sum reactants	-327.60896	0.16860	-327.23376	0.0
PRE^{7}_{ax}	-327.61953	0.17096	-327.24357	-19.6
SP^{7}_{ax}	-327.61469	0.17015	-327.23845	-8.3
$POST^{7}_{ax}$	-327.65782	0.17026	-327.28376	-126.9
PIPṄ	-251.21565	0.14592	-250.90064	
H_2O	-76.43011	0.02155	-76.37098	
Sum products	-327.64576	0.16748	-327.27163	-102.4
PRE^{8}_{ax}	-327.62419	0.17178	-327.24812	-29.4

SP_{ax}^{8}	-327.61070	0.16764	-327.23497	-5.7
$POST_{ax}^{8}$	-327.66298	0.17081	-327.28644	-132.5
$PRE_{ax}^{9\text{-}1}$	-327.62419	0.17178	-327.24812	-29.4
$SP_{ax}^{9\text{-}1}$	-327.60892	0.16745	-327.23350	-2.4
$PRE_{ax}^{9\text{-}2}$	-327.61264	0.16970	-327.23613	-3.4
$SP_{ax}^{9\text{-}2}$	-327.60627	0.16692	-327.23082	3.3
$POST_{ax}^{9}$	-327.64600	0.16975	-327.26971	-91.4
$PIP\dot{C}^{2}$	-251.22262	0.14655	-250.90654	
H_2O	-76.43011	0.02155	-76.37098	
Sum products	-327.65272	0.16810	-327.27752	-116.2
PRE_{ax}^{10}	-327.61350	0.16993	-327.23703	-5.1
SP_{ax}^{10}	-327.60827	0.16700	-327.23291	-2.0
$POST_{ax}^{10}$	-327.64745	0.16937	-327.27044	-94.3
$PRE_{ax}^{11\text{-}1}$	-327.61264	0.16996	-327.23613	-2.7
$SP_{ax}^{11\text{-}1}$	-327.60670	0.16692	-327.23160	1.2
$PRE_{ax}^{11\text{-}2}$	-327.61237	0.16961	-327.23595	-3.1
$SP_{ax}^{11\text{-}2}$	-327.60651	0.16684	-327.23140	1.5
$POST_{ax}^{11}$	-327.64411	0.16901	-327.26839	-89.8
$PIP\dot{C}^{3}$	-251.20842	0.14522	-250.89186	
H_2O	-76.43011	0.02155	-76.37098	
Sum products	-327.63853	0.16677	-327.26284	-82.4
$PRE_{ax}^{12\text{-}1}$	-327.61195	0.16964	-327.23579	-2.6
$SP_{ax}^{12\text{-}1}$	-327.60739	0.16706	-327.23229	-0.2
$PRE_{ax}^{12\text{-}2}$	-327.62419	0.17178	-327.24812	-29.4
$SP_{ax}^{12\text{-}2}$	-327.60747	0.16673	-327.23176	0.3
$POST_{ax}^{12}$	-327.64673	0.16897	-327.27008	-94.4
$PRE_{ax}^{13\text{-}1}$	-327.61264	0.17005	-327.23616	-2.5
$SP_{ax}^{13\text{-}1}$	-327.60713	0.16709	-327.23205	0.5
$PRE_{ax}^{13\text{-}2}$	-327.61277	0.17012	-327.23604	-2.0
$SP_{ax}^{13\text{-}2}$	-327.60708	0.16703	-327.23182	1.0

$POST^{13}_{ax}$	-327.64636	0.16936	-327.27037	-94.1
PIPĊ[4]	-251.21082	0.14546	-250.89424	
H_2O	-76.43011	0.02155	-76.37098	
Sum products	-327.64092	0.16701	-327.26522	-86.8

Table S4, continued.
T_1 and D_1 diagnostic values, vibrational frequencies (cm^{-1}), Rotational constants (GHz) and Cartesian coordinates of the species listed above. Results from CCSD(T*)-F12a/aug-cc-pVTZ//M06-2X/aug-cc-pVTZ calculations.

Piperidine, *ax* ($^1C_4$)	C	0.745800	-1.207902	0.207212
	N	1.373475	-0.000000	-0.314988
T_1= 0.00980401, D_1= 0.02483925	C	0.745801	1.207901	0.207212
	C	-0.712203	1.254865	-0.230204
$\tilde{\nu}$: 250.9, 255.5, 410.2, 435.8, 452.7, 551.9, 775.3, 825.6, 839.9,	C	-1.446992	0.000000	0.235978
878.9, 898.0, 921.2, 983.9, 1061.7, 1070.3, 1076.5, 1158.7,	C	-0.712204	-1.254865	-0.230204
1173.2, 1174.4, 1196.8, 1291.2, 1299.7, 1320.6, 1352.0, 1366.6,	H	0.784648	-1.245302	1.308636
1384.2, 1385.7, 1426.2, 1475.5, 1485.9, 1487.6, 1495.0, 1503.1,	H	1.286778	-2.074559	-0.172231
1514.6, 2948.2, 2949.6, 3040.3, 3060.4, 3060.4, 3098.8, 3100.8,	H	2.362451	-0.000000	-0.106717
3102.5, 3106.4, 3110.1, 3569.1	H	1.286779	2.074558	-0.172232
	H	0.784649	1.245302	1.308635
B: 4.5600000, 0.0076587, 4.4768877	H	-1.189887	2.151042	0.168001
	H	-0.745709	1.315028	-1.320333
	H	-2.475621	0.000000	-0.126174
	H	-1.494031	0.000000	1.329696
	H	-1.189887	-2.151041	0.168001
	H	-0.745709	-1.315028	-1.320333
OH	O	0.00000	0.00000	0.107992
T_1= 0.0080, D_1= 0.0145	H	0.00000	0.00000	-0.863937
$\tilde{\nu}$: 3769.6				
B: 0.0000000, 564.2863408, 564.2863408				
PRE^{7}_{ax}	C	-0.334480	1.074989	0.274964
	N	-0.830087	-0.236776	-0.125708
T_1= 0.01939542, D_1= 0.10819154	C	0.069151	-1.322819	0.246750
	C	1.495193	-1.117300	-0.264585
$\tilde{\nu}$: 44.9, 50.8, 129.9, 147.3, 242.6, 261.5, 399.2, 428.6, 457.8,	C	2.031309	0.239820	0.186793
469.5, 574.9, 777.3, 815.3, 835.0, 872.3, 893.4, 925.2, 955.7,	C	1.079011	1.358313	-0.231949
1024.8, 1055.1, 1075.8, 1129.8, 1150.3, 1189.9, 1231.4, 1285.2,	H	-0.338747	1.104909	1.369216
1302.0, 1343.0, 1350.9, 1373.8, 1383.0, 1384.8, 1399.0, 1474.4,	H	-1.044363	1.824216	-0.070837
1488.2, 1490.5, 1493.4, 1497.1, 1507.6, 3037.8, 3038.4, 3042.0,	H	-1.023963	-0.254610	-1.119000
3047.1, 3049.3, 3096.9, 3097.6, 3101.7, 3111.6, 3126.2, 3558.4,	H	-0.348200	-2.260613	-0.118870
3823.9	H	0.084304	-1.376762	1.339836
	H	2.137038	-1.926114	0.088783
B: 4.469459 1.593417 1.261920	H	1.490178	-1.162428	-1.357780
	H	3.029218	0.412126	-0.217322
	H	2.125853	0.240948	1.277394
	H	1.423365	2.322506	0.145544
	H	1.057395	1.428597	-1.323699
	O	-3.214151	0.040638	-0.138930
	H	-3.109363	-0.418462	0.706284

SP$^{7}_{ax}$

T_1= 0.03267199, D_1= 0.20045604

$\tilde{\nu}$: -392.5, 69.3, 107.2, 125.9, 216.8, 248.0, 298.7, 408.4, 453.5, 457.5, 575.2, 604.9, 821.9, 837.3, 863.4, 896.9, 916.8, 972.1, 1031.9, 1056.7, 1078.3, 1162.4, 1170.3, 1171.0, 1223.0, 1284.9, 1300.5, 1331.9, 1352.0, 1372.8, 1381.5, 1384.0, 1398.1, 1464.1, 1486.9, 1488.5, 1493.6, 1495.1, 1508.3, 2968.5, 3009.7, 3041.1, 3060.1, 3061.2, 3103.9, 3108.8, 3111.6, 3120.9, 3139.1, 3634.0, 3818.8

B: 4.251460 1.830648 1.415668

C	0.197814	-1.253225	-0.012836
N	0.756019	-0.041270	-0.553362
C	0.279846	1.205820	-0.008054
C	-1.238955	1.285099	-0.148870
C	-1.892060	0.046648	0.464192
C	-1.324558	-1.232243	-0.150403
H	0.448695	-1.339563	1.055377
H	0.633320	-2.107130	-0.528829
H	1.575569	-0.063620	-1.135292
H	0.779411	2.024111	-0.520828
H	0.553379	1.269701	1.052736
H	-1.603475	2.191223	0.336319
H	-1.497883	1.348723	-1.208395
H	-2.973816	0.081442	0.333673
H	-1.701326	0.039417	1.542054
H	-1.747474	-2.113951	0.332644
H	-1.583745	-1.276524	-1.210623
O	2.866045	0.083478	0.274774
H	2.764335	-0.745358	0.762334

POST$^{7}_{ax}$

T_1= 0.01384121, D_1= 0.06179489

$\tilde{\nu}$: 24.6, 62.4, 88.1, 155.0, 226.7, 276.9, 344.4, 413.6, 443.7, 460.5, 564.7, 625.8, 817.7, 841.7, 858.4, 888.6, 900.1, 978.5, 983.6, 1036.7, 1076.3, 1100.8, 1145.7, 1146.6, 1249.4, 1250.2, 1286.4, 1295.9, 1330.0, 1333.4, 1350.5, 1384.4, 1385.7, 1485.7, 1488.2, 1497.2, 1501.3, 1522.4, 1625.4, 2992.6, 3001.0, 3045.3, 3068.4, 3069.7, 3102.0, 3112.6, 3114.2, 3118.5, 3120.7, 3609.8, 3932.3

B: 2.847974 1.892444 1.798520

C	0.326344	1.193430	0.922712
N	-0.459853	0.009543	1.153083
C	0.315520	-1.183772	0.934926
C	0.707491	-1.255973	-0.553985
C	1.492923	-0.010044	-0.948680
C	0.718540	1.246765	-0.566902
H	1.242523	1.171249	1.528608
H	-0.253899	2.073177	1.196521
H	-2.088547	0.006571	0.112532
H	-0.272837	-2.055328	1.217456
H	1.231741	-1.163761	1.540835
H	1.285217	-2.164290	-0.725974
H	-0.207270	-1.321114	-1.146458
H	1.696830	-0.016496	-2.019914
H	2.459687	-0.011635	-0.436428
H	1.304121	2.148151	-0.748530
H	-0.195770	1.313598	-1.159894
O	-2.602808	-0.000485	-0.713240
H	-3.525267	0.014528	-0.452841

PIPṄ

T_1= 0.01389069, D_1= 0.05612892

$\tilde{\nu}$: 217.5, 237.5, 364.2, 445.3, 454.7, 562.1, 811.8, 840.0, 857.9, 891.7, 894.9, 971.2, 980.6, 1037.3, 1075.9, 1106.1, 1137.2, 1140.5, 1243.1, 1251.0, 1284.1, 1294.9, 1332.0, 1336.1, 1351.9, 1383.7, 1387.6, 1482.5, 1488.7, 1494.0, 1499.4, 1517.9, 2978.7, 2987.6, 3047.0, 3063.0, 3063.4, 3100.3, 3106.9, 3108.8, 3110.7, 3113.8

B: 4.652185 4.648684 2.646002

C	-0.031830	-0.829670	1.186394
N	-0.715167	-1.280712	0.000000
C	-0.031830	-0.829670	-1.186394
C	-0.031830	0.706571	-1.250223
C	0.620061	1.286733	-0.000000
C	-0.031830	0.706571	1.250223
H	1.011884	-1.176624	1.173678
H	-0.520082	-1.249767	2.064543
H	-0.520082	-1.249767	-2.064543
H	1.011884	-1.176624	-1.173678
H	0.483802	1.030011	-2.154836
H	-1.065924	1.050895	-1.321138
H	0.545041	2.374817	-0.000000
H	1.685317	1.037933	-0.000000
H	0.483802	1.030011	2.154836
H	-1.065924	1.050895	1.321138

H_2O

T_1= 0.00895900, D_1= 0.01776535

$\tilde{\nu}$: 3971.4, 3868.5, 1619.7

B: 834.0336966 430.9641048 284.1416681

	0.000000	0.000000	0.114963
	0.000000	0.762576	-0.466991
	0.000000	-0.762576	-0.466991

PRE^{8}_{ax}

T_1= 0.01358441, D_1= 0.06272627

$\tilde{\nu}$: 37.8, 44.8, 177.6, 242.8, 269.2, 401.3, 446.0, 457.0, 561.8, 621.6, 761.1, 814.2, 833.4, 852.2, 887.2, 900.2, 933.7, 954.4, 1032.9, 1059.6, 1075.7, 1130.5, 1146.5, 1195.3, 1225.8, 1289.8, 1304.0, 1348.3, 1354.4, 1377.4, 1386.2, 1389.9, 1407.1, 1471.8, 1489.3, 1490.2, 1493.9, 1495.1, 1510.2, 3038.3, 3040.0, 3045.6, 3050.4, 3053.8, 3100.3, 3101.6, 3105.5, 3111.5, 3112.7, 3240.5, 3530.7

B: 4.083587 1.570814 1.283170

C	-0.073772	-1.280531	0.049493
N	-0.730337	-0.138413	0.692968
C	-0.294056	1.140214	0.121790
C	1.221271	1.317061	0.117049
C	1.895192	0.138814	-0.582352
C	1.448668	-1.179400	0.045767
H	-0.442950	-1.321028	-0.979649
H	-0.407252	-2.191685	0.545321
H	-0.536818	-0.149215	1.688363
H	-0.785488	1.944309	0.668931
H	-0.670731	1.174797	-0.905971
H	1.482448	2.258295	-0.368636
H	1.576084	1.378999	1.150535
H	2.980093	0.236806	-0.540704
H	1.615145	0.143434	-1.640161
H	1.874601	-2.028778	-0.490203
H	1.813052	-1.233892	1.076388
O	-3.265628	0.007402	-0.457213
H	-2.444625	-0.119310	0.092229

SP^{8}_{ax}

T_1= 0.02128877, D_1= 0.11971965

$\tilde{\nu}$: -687.3, 69.4, 108.6, 219.0, 248.8, 323.1, 389.6, 442.8, 455.7, 554.4, 575.0, 696.5, 813.2, 835.3, 871.7, 895.6, 925.5, 949.9, 1028.4, 1057.5, 1076.8, 1094.8, 1144.6, 1179.2, 1211.8, 1268.2, 1285.4, 1316.5, 1345.2, 1356.8, 1382.4, 1386.5, 1391.6, 1415.3, 1465.7, 1482.7, 1488.4, 1490.5, 1503.0, 1816.5, 3026.9, 3039.8, 3050.9, 3053.5, 3099.4, 3102.9, 3105.8, 3110.7, 3114.4, 3558.3, 3760.4

B: 3.124879 1.982895 1.731705

C	0.676628	0.565244	0.829221
N	0.388481	-0.836569	1.005942
C	-0.408896	-1.419713	-0.070967
C	-1.655277	-0.611604	-0.422935
C	-1.261681	0.825990	-0.752068
C	-0.503536	1.441428	0.419449
H	1.452584	0.636954	-0.017932
H	1.182763	0.945670	1.715501
H	-0.021765	-1.017204	1.912902
H	-0.663943	-2.440752	0.210204
H	0.231534	-1.474413	-0.956230
H	-2.169838	-1.075851	-1.265600
H	-2.346751	-0.619116	0.425381
H	-2.139770	1.424924	-0.994063
H	-0.615634	0.826136	-1.634857
H	-0.150235	2.444146	0.177063
H	-1.175855	1.532664	1.279991
O	2.507284	0.075040	-0.938263
H	2.555846	-0.735572	-0.404045

$POST^{8}_{ax}$

T_1= 0.01519518, D_1= 0.07104167

$\tilde{\nu}$: 34.4, 81.6, 142.4, 146.9, 226.5, 265.2, 327.8, 392.5, 423.8, 450.9, 485.4, 551.2, 721.4, 728.2, 834.3, 847.1, 907.1, 922.7, 934.5, 1051.5, 1076.0, 1084.9, 1110.8, 1178.0, 1193.2, 1258.7, 1283.1, 1307.7, 1353.4, 1370.7, 1385.8, 1387.5, 1461.7, 1472.8,

C	0.260695	1.022366	0.665932
N	0.415231	-0.281661	1.104062
C	-0.081161	-1.356089	0.248701
C	-1.497858	-1.050456	-0.212581
C	-1.549250	0.312359	-0.897101
C	-1.057687	1.391947	0.066632
H	2.063928	0.538637	-0.569510
H	0.802124	1.760643	1.243582

1486.9, 1495.6, 1511.7, 1525.4, 1616.9, 2968.9, 3008.0, 3049.7, 3058.3, 3103.1, 3106.3, 3111.4, 3113.2, 3182.4, 3597.2, 3706.6, 3933.6

B: 3.340142, 1.716872, 1.495540

H	1.327553	-0.476260	1.489682
H	-0.049364	-2.284860	0.816297
H	0.571682	-1.470403	-0.626191
H	-1.833057	-1.839029	-0.886540
H	-2.165149	-1.048962	0.653116
H	-2.561203	0.536468	-1.234772
H	-0.904064	0.295285	-1.780103
H	-0.976569	2.356828	-0.433471
H	-1.808813	1.511419	0.862001
O	2.843962	-0.029984	-0.652296
H	3.426183	0.410972	-1.273653

$PRE_{ax}^{9\text{-}1}$ see PRE_{ax}^{8}

$SP_{ax}^{9\text{-}1}$

T_1= 0.02095020, D_1= 0.11654167

$\tilde{\nu}$: -685.4, 64.4, 100.4, 228.4, 247.3, 301.1, 395.2, 431.1, 457.2, 543.6, 652.1, 810.4, 817.2, 839.5, 880.7, 914.0, 934.0, 945.8, 1018.4, 1032.5, 1071.2, 1096.2, 1143.1, 1160.4, 1195.1, 1264.0, 1286.9, 1313.2, 1333.2, 1349.1, 1362.9, 1369.5, 1384.0, 1390.1, 1474.3, 1487.0, 1490.7, 1496.0, 1503.3, 1758.1, 3040.4, 3045.7, 3052.4, 3052.9, 3069.2, 3096.1, 3102.1, 3111.9, 3112.7, 3509.0, 3766.8

B: 4.338064, 1.633254, 1.288360

C	-0.732280	0.278560	0.476798
N	-0.423712	-1.048129	-0.014173
C	0.986030	-1.377950	0.210342
C	1.937599	-0.341106	-0.386998
C	1.596247	1.060591	0.115168
C	0.117288	1.383128	-0.129593
H	-0.660873	0.276089	1.567843
H	-1.846744	0.477885	0.233896
H	-0.615842	-1.076416	-1.011176
H	1.179652	-2.369938	-0.196706
H	1.139150	-1.436162	1.291720
H	2.970258	-0.595806	-0.142363
H	1.849554	-0.367869	-1.477452
H	2.229788	1.806964	-0.365043
H	1.797623	1.115487	1.189195
H	-0.146252	2.350405	0.298628
H	-0.076856	1.434276	-1.204533
O	-3.159835	0.070620	-0.224605
H	-3.004097	-0.862313	-0.002252

$PRE_{ax}^{9\text{-}2}$

T_1= 0.01022875, D_1= 0.02552982

$\tilde{\nu}$: 62.2, 77.0, 110.1, 259.2, 272.0, 279.7, 328.7, 420.8, 444.9, 463.9, 578.1, 783.7, 826.8, 830.4, 879.7, 883.6, 931.1, 965.5, 1023.5, 1057.0, 1070.7, 1134.5, 1148.0, 1195.6, 1228.2, 1290.0, 1304.8, 1344.4, 1355.8, 1375.6, 1385.3, 1386.8, 1405.0, 1478.2, 1488.1, 1492.5, 1494.0, 1499.1, 1509.8, 2989.0, 2989.5, 2995.5, 2999.6, 3005.0, 3030.0, 3045.4, 3052.1, 3056.3, 3064.5, 3489.1, 3709.1

B: 3.544679 1.647016 1.382061

C	-0.559724	0.006930	1.007314
N	-0.186138	-1.236807	0.345211
C	1.236420	-1.276050	0.017409
C	1.727304	-0.061391	-0.780206
C	1.351220	1.238372	-0.062226
C	-0.151570	1.266801	0.233194
H	-0.069419	0.018505	1.991756
H	-1.640178	-0.015941	1.197461
H	-0.752218	-1.358870	-0.491868
H	1.442332	-2.205884	-0.521853
H	1.788574	-1.328797	0.966920
H	2.811624	-0.121158	-0.930241
H	1.261621	-0.074592	-1.775644
H	1.646958	2.109620	-0.656346
H	1.902785	1.298358	0.886867
H	-0.419071	2.167523	0.799262
H	-0.701884	1.296689	-0.718618
O	-3.053617	-0.127366	-0.629479
H	-3.161121	0.843160	-0.621245

$SP_{ax}^{9\text{-}2}$

C	-0.719615	-0.069891	0.603388
N	-0.138714	-1.262906	0.036518

T_1= 0.02087965, D_1= 0.11660695

$\tilde{\nu}$: -669.1, 52.0, 85.0, 120.5, 231.2, 247.4, 375.3, 435.9, 453.6, 533.3, 658.6, 775.7, 809.8, 839.5, 867.3, 898.0, 933.7, 942.1, 1018.6, 1063.3, 1067.8, 1131.3, 1139.0, 1164.3, 1220.3, 1283.1, 1289.0, 1317.1, 1332.3, 1348.3, 1366.5, 1370.3, 1383.1, 1393.8, 1473.4, 1487.2, 1489.8, 1494.1, 1502.5, 1732.4, 3039.2, 3039.3, 3046.1, 3049.0, 3057.7, 3094.7, 3095.7, 3101.9, 3112.0, 3526.0, 3783.2

B: 4.208350 1.624381 1.292792

C	1.321034	-1.243103	0.126521
C	1.942060	0.012419	-0.487219
C	1.321379	1.270019	0.117925
C	-0.205266	1.228232	-0.001372
H	-0.571569	-0.081851	1.687588
H	-1.853638	-0.152114	0.439180
H	-0.427712	-1.348468	-0.932089
H	1.709114	-2.144901	-0.346002
H	1.583749	-1.296111	1.187411
H	3.023216	0.007623	-0.337540
H	1.765802	0.003501	-1.567142
H	1.716187	2.166008	-0.362106
H	1.592711	1.329968	1.176323
H	-0.656264	2.089893	0.494641
H	-0.489736	1.264928	-1.057585
O	-3.150886	-0.127279	-0.295727
H	-3.171325	0.834048	-0.427949

$POST^{9}_{ax}$

T_1= 0.01327695, D_1= 0.05569752

$\tilde{\nu}$: 53.9, 73.1, 128.7, 147.5, 202.6, 239.9, 247.6, 346.5, 395.3, 434.3, 467.4, 539.0, 696.6, 820.4, 837.0, 873.4, 895.7, 926.4, 942.3, 997.9, 1055.9, 1073.2, 1138.1, 1153.8, 1166.8, 1233.6, 1284.9, 1299.8, 1322.2, 1344.6, 1367.7, 1379.9, 1386.4, 1477.2, 1485.0, 1487.0, 1498.6, 1501.7, 1617.2, 3040.3, 3043.1, 3050.3, 3057.2, 3093.2, 3099.8, 3112.0, 3112.9, 3148.4, 3487.4, 3793.1, 3935.2

B: 3.537670 1.648787 1.396868

C	-0.411907	0.167537	1.057709
N	-0.281615	-1.108996	0.452608
C	1.129672	-1.350470	0.094107
C	1.681866	-0.238581	-0.800506
C	1.472238	1.139080	-0.172043
C	-0.007185	1.342218	0.229117
H	-0.207162	0.188383	2.123513
H	-2.602898	0.173556	0.263622
H	-0.830058	-1.117913	-0.404382
H	1.204364	-2.322428	-0.393248
H	1.702146	-1.402827	1.023536
H	2.742170	-0.408472	-0.997316
H	1.165573	-0.278548	-1.764413
H	1.786594	1.926378	-0.858578
H	2.093033	1.225112	0.723976
H	-0.133770	2.282051	0.765688
H	-0.617916	1.383609	-0.678663
O	-2.972627	0.035493	-0.616933
H	-3.737858	-0.528580	-0.486827

PIPĊ[2]

T_1= 0.01546620, D_1= 0.06541086

$\tilde{\nu}$: 225.7, 250.6, 385.8, 437.5, 470.4, 514.8, 664.8, 709.2, 834.7, 846.5, 906.5, 921.2, 932.4, 1050.9, 1075.6, 1083.3, 1107.6, 1176.0, 1190.0, 1249.7, 1281.5, 1306.3, 1351.8, 1367.8, 1383.7, 1385.6, 1455.8, 1470.8, 1485.8, 1493.4, 1504.4, 1516.8, 2955.2, 2987.8, 3051.5, 3058.1, 3102.9, 3104.7, 3107.1, 3110.2, 3200.6, 3612.8

B: 4.759784 4.575280 2.582603

C	0.045985	1.434030	0.153552
N	-1.151463	0.809575	-0.174262
C	-1.297427	-0.595290	0.189626
C	-0.086883	-1.385542	-0.284854
C	1.195420	-0.774716	0.273452
C	1.301676	0.689127	-0.156205
H	0.034614	2.512875	0.086339
H	-1.968971	1.362092	0.030685
H	-2.210747	-0.976190	-0.265778
H	-1.387035	-0.705684	1.279169
H	-0.191718	-2.425725	0.024970
H	-0.055717	-1.365900	-1.377244
H	2.066897	-1.335560	-0.065128
H	1.172832	-0.828722	1.365278
H	2.147892	1.176537	0.327464
H	1.499568	0.713601	-1.239349

PRE^{10}_{ax}

C	-0.040940	0.250034	1.467214

T_1= 0.01416986, D_1= 0.06692537

$\tilde{\nu}$: 57.3, 87.9, 117.4, 269.0, 272.1, 277.1, 387.3, 417.2, 444.1, 464.1, 577.2, 796.1, 824.1, 831.5, 879.1, 885.4, 934.4, 963.9, 1025.1, 1056.7, 1072.6, 1137.1, 1149.2, 1194.1, 1229.8, 1287.9, 1305.1, 1345.8, 1356.9, 1370.0, 1386.3, 1387.7, 1404.8, 1471.4, 1487.2, 1490.4, 1492.7, 1506.9, 1512.4, 2965.3, 2993.2, 2996.1, 2998.8, 3007.6, 3044.1, 3052.8, 3055.0, 3056.4, 3065.1, 3491.5, 3710.0

B: 2.655972 1.953592 1.879093

N	-0.070329	-1.099464	0.914822
C	0.987391	-1.314812	-0.068937
C	1.005413	-0.271019	-1.191851
C	1.090422	1.139453	-0.602113
C	-0.036131	1.361885	0.409861
H	0.870364	0.338630	2.076563
H	-0.890358	0.363652	2.149220
H	-0.977209	-1.272374	0.486485
H	0.878617	-2.324669	-0.476407
H	1.947562	-1.286924	0.466760
H	1.845791	-0.457863	-1.870575
H	0.081102	-0.364569	-1.778707
H	1.054234	1.895829	-1.393399
H	2.056752	1.259613	-0.091220
H	0.054534	2.343997	0.889125
H	-0.998076	1.351949	-0.127170
O	-2.545267	-0.206872	-0.796890
H	-3.105805	0.510714	-0.444352

SP_{ax}^{10}

T_1= 0.02019269, D_1= 0.11134352

$\tilde{\nu}$: -590.4, 68.2, 71.1, 91.8, 234.6, 248.6, 392.3, 439.5, 459.1, 553.7,
663.9, 795.8, 805.9, 832.9, 872.9, 888.7, 926.1, 963.3, 1017.8, 1060.9, 1073.0, 1094.1, 1150.6, 1183.6, 1217.0, 1250.2, 1289.9, 1330.8, 1339.2, 1349.4, 1368.5, 1375.7, 1391.1, 1398.4, 1475.4, 1481.7, 1485.5, 1499.2, 1503.1, 1708.0, 3022.9, 3028.0, 3038.8, 3053.8, 3090.9, 3100.2, 3102.0, 3103.4, 3108.2, 3524.8, 3780.6

B: 2.909630 2.054050 1.853833

C	-0.119383	-1.038594	1.016762
N	0.534157	-1.422005	-0.226689
C	1.569405	-0.466487	-0.611421
C	1.068301	0.974673	-0.706369
C	0.400849	1.394851	0.601829
C	-0.661439	0.384280	1.002247
H	0.616350	-1.126561	1.824741
H	-0.914561	-1.753106	1.229968
H	-0.168543	-1.463587	-0.957734
H	2.000686	-0.788182	-1.559123
H	2.364852	-0.525431	0.138613
H	1.893171	1.646792	-0.948583
H	0.338718	1.044845	-1.517748
H	-0.027284	2.393925	0.519536
H	1.153901	1.434798	1.397788
H	-1.167736	0.647656	1.932145
H	-1.458815	0.412151	0.175777
O	-2.349756	0.085711	-1.001384
H	-3.118188	-0.147291	-0.455772

$POST_{ax}^{10}$

T_1= 0.01158852, D_1= 0.03791839

$\tilde{\nu}$: 35.3, 88.1, 136.0, 149.7, 234.3, 260.8, 375.9, 386.6, 450.1, 465.4, 525.0, 594.8, 674.2, 769.4, 839.1, 844.0, 905.7, 916.5, 934.6, 1053.9, 1072.0, 1082.5, 1112.8, 1177.1, 1195.0, 1234.2, 1286.1, 1309.2, 1350.8, 1368.0, 1379.8, 1392.5, 1449.0, 1470.5, 1489.7, 1491.2, 1501.0, 1513.3, 1637.8, 2974.8, 3008.3, 3051.3, 3068.0, 3105.7, 3108.6, 3109.9, 3117.3, 3197.2, 3587.9, 3639.4, 3930.8

B: 2.773676 1.930918 1.857439

C	0.304819	1.198303	0.906489
N	-0.425889	0.047275	1.225535
C	0.187775	-1.236023	0.861247
C	0.630863	-1.213299	-0.594482
C	1.570988	-0.037756	-0.845868
C	0.873266	1.270860	-0.470675
H	-0.018982	2.099802	1.407570
H	-1.922074	0.115970	-0.093866
H	-0.760786	0.050726	2.178042
H	-0.547534	-2.022189	1.028319
H	1.057360	-1.432757	1.500620
H	1.118478	-2.159751	-0.829200
H	-0.248822	-1.124706	-1.235973
H	1.881754	-0.014573	-1.890227
H	2.471187	-0.155079	-0.236224
H	1.558862	2.114959	-0.538935
H	0.067494	1.450207	-1.198100

O	-2.472468	0.047034	-0.891488
H	-3.302238	0.477684	-0.679145

$PRE_{ax}^{11\text{-}1}$

T_1= 0.02100943, D_1= 0.11743746

$\tilde{\nu}$: -569.4, 50.0, 75.3, 80.2, 235.3, 244.6, 381.1, 428.6, 452.0, 549.8, 655.7, 756.1, 812.0, 839.9, 874.7, 894.7, 936.7, 954.4, 1014.7, 1049.5, 1081.8, 1123.3, 1143.6, 1184.9, 1224.4, 1252.5, 1300.7, 1326.7, 1343.4, 1355.3, 1370.7, 1372.8, 1389.5, 1395.1, 1477.6, 1487.9, 1489.1, 1493.6, 1502.1, 1677.0, 3037.0, 3041.6, 3048.2, 3052.9, 3067.9, 3094.2, 3098.3, 3107.5, 3122.1, 3533.9, 3787.9

B: 4.269848, 1.563273, 1.247116

C	0.019285	-1.305705	-0.077659
N	-1.431006	-1.214743	0.068688
C	-1.959514	0.045849	-0.440198
C	-1.292538	1.281606	0.164202
C	0.225825	1.197290	-0.011626
C	0.741041	-0.118519	0.546249
H	0.244916	-1.339821	-1.147119
H	0.359982	-2.244616	0.355654
H	-1.685410	-1.326132	1.043543
H	-3.035833	0.064026	-0.269424
H	-1.806177	0.048949	-1.523782
H	-1.683395	2.189463	-0.298623
H	-1.532370	1.331937	1.230462
H	0.716174	2.044688	0.469889
H	0.461853	1.246577	-1.079766
H	1.860702	-0.233754	0.317926
H	0.666139	-0.141106	1.637061
O	3.237064	-0.075647	-0.231260
H	3.149355	0.865042	-0.452368

$SP_{ax}^{11\text{-}1}$

T_1= 0.02100943, D_1= 0.11743746

$\tilde{\nu}$: -569.4, 50.0,75.3,80.2, 235.3, 244.6, 381.1, 428.6, 452.0, 549.8, 655.7, 756.1, 812.0, 839.9, 874.7, 894.7, 936.7, 954.4, 1014.7, 1049.5, 1081.8, 1123.3, 1143.6, 1184.9, 1224.4, 1252.5, 1300.7, 1326.7, 1343.4, 1355.3, 1370.7, 1372.8, 1389.5, 1395.1, 1477.6, 1487.9, 1489.1, 1493.6, 1502.1, 1677.0, 3037.0, 3041.6, 3048.2, 3052.9, 3067.9, 3094.2, 3098.3, 3107.5, 3122.1, 3533.9, 3787.9

B: 4.2698481.5632731.247116

C	0.019285	-1.305705	-0.077659
N	-1.431006	-1.214743	0.068688
C	-1.959514	0.045849	-0.440198
C	-1.292538	1.281606	0.164202
C	0.225825	1.197290	-0.011626
C	0.741041	-0.118519	0.546249
H	0.244916	-1.339821	-1.147119
H	0.359982	-2.244616	0.355654
H	-1.685410	-1.326132	1.043543
H	-3.035833	0.064026	-0.269424
H	-1.806177	0.048949	-1.523782
H	-1.683395	2.189463	-0.298623
H	-1.532370	1.331937	1.230462
H	0.716174	2.044688	0.469889
H	0.461853	1.246577	-1.079766
H	1.860702	-0.233754	0.317926
H	0.666139	-0.141106	1.637061
O	3.237064	-0.075647	-0.231260
H	3.149355	0.865042	-0.452368

$PRE_{ax}^{11\text{-}2}$

T_1= 0.01023611, D_1= 0.02487043

$\tilde{\nu}$: 27.2, 47.2, 81.2, 83.1, 239.9, 247.1, 266.7, 394.3, 440.7, 458.2, 556.8, 759.5, 812.1, 833.6, 871.8, 891.8, 928.4, 954.1, 1027.7, 1059.6, 1075.8, 1128.0, 1152.3, 1193.5, 1229.1, 1286.5, 1300.4, 1342.8, 1350.5, 1370.8, 1382.4, 1385.1, 1399.7, 1468.3, 1478.9, 1485.8, 1489.5, 1493.4, 1504.9, 2997.7, 3035.1, 3037.8, 3043.2, 3048.8, 3092.6, 3096.4, 3098.6, 3101.7, 3106.3, 3529.5, 3761.4

B: 3.191162 1.680419 1.472130

C	0.331379	-1.051452	0.558614
N	-0.974490	-1.412150	0.019243
C	-1.482561	-0.395834	-0.898443
C	-1.504270	1.013212	-0.306946
C	-0.121300	1.388959	0.221046
C	0.379666	0.332699	1.203038
H	1.036200	-1.072667	-0.282468
H	0.644322	-1.823843	1.261887
H	-1.630936	-1.540330	0.781199
H	-2.476366	-0.696102	-1.230679
H	-0.833746	-0.402682	-1.780140
H	-1.839254	1.729528	-1.059442
H	-2.229215	1.043933	0.512839

H	-0.141021	2.372963	0.690655
H	0.580658	1.448943	-0.616316
H	1.397990	0.558552	1.523164
H	-0.254690	0.330251	2.095343
O	2.956292	0.124530	-0.744330
H	3.299673	-0.785243	-0.739963

$SP_{ax}^{11\text{-}2}$

T_1= 0.02079706, D_1= 0.11586353

$\tilde{\nu}$: -597.9, 53.5, 67.5, 75.4, 232.9, 243.8, 378.7, 432.8, 448.6, 545.5, 663.2, 759.0, 810.5, 839.2, 872.7, 897.2, 937.9, 956.3, 1017.5, 1049.2, 1098.8, 1124.1, 1143.2, 1177.3, 1209.6, 1258.9, 1300.0, 1331.3, 1343.1, 1353.6, 1370.5, 1379.5, 1385.6, 1394.7, 1478.5, 1487.9, 1490.0, 1493.9, 1501.5, 1651.7, 3037.2, 3040.7, 3046.0, 3050.9, 3066.3, 3097.8, 3104.4, 3107.0, 3112.1, 3532.0, 3782.9

B: 4.242075 1.570140 1.252968

C	0.206030	-1.198186	-0.011838
N	-1.243255	-1.322731	0.111398
C	-1.941785	-0.172372	-0.454275
C	-1.474377	1.169422	0.108791
C	0.042781	1.307961	-0.046722
C	0.735644	0.105213	0.568492
H	0.448100	-1.239705	-1.078948
H	0.673208	-2.061008	0.463496
H	-1.494611	-1.429809	1.087626
H	-3.011866	-0.308224	-0.298044
H	-1.770630	-0.187902	-1.535061
H	-1.987111	1.991254	-0.394106
H	-1.738665	1.223405	1.169201
H	0.397759	2.233608	0.405443
H	0.292902	1.352896	-1.110768
H	1.861531	0.184708	0.352610
H	0.652531	0.108995	1.659043
O	3.226172	0.115170	-0.242038
H	3.160499	-0.802683	-0.550664

$POST_{ax}^{11}$

T_1= 0.01321021, D_1= 0.05568850

$\tilde{\nu}$: 23.1, 45.8, 58.0,116.7,209.9,227.6,239.9,321.1,343.8,434.2, 446.5,497.2,635.4,789.9,835.5,867.2,879.8,917.6,962.9, 1000.5, 1030.0, 1106.5, 1136.8, 1156.7, 1161.1, 1224.5, 1302.3, 1321.6, 1338.6, 1346.9, 1366.4, 1382.7, 1391.5, 1472.7, 1483.1, 1486.1, 1491.0, 1499.5, 1616.2, 3020.1, 3032.9, 3044.5, 3049.5, 3097.2, 3106.2, 3112.8, 3120.5, 3186.7, 3520.8, 3791.8, 3938.9

B: 3.5451121.5365121.307314

C	0.040150	-1.290933	0.263480
N	-1.384829	-1.250493	-0.115017
C	-1.714778	-0.009358	-0.810363
C	-1.362976	1.254945	-0.025383
C	0.124845	1.228743	0.377964
C	0.440673	-0.074053	1.033734
H	0.608952	-1.346131	-0.671278
H	0.228302	-2.208620	0.817777
H	-1.949985	-1.322601	0.724556
H	-2.776737	-0.024724	-1.056410
H	-1.164394	-0.011494	-1.755979
H	-1.579564	2.144970	-0.619201
H	-1.981543	1.301473	0.875124
H	0.368765	2.074666	1.018851
H	0.721607	1.328313	-0.536997
H	2.553914	-0.022037	0.156713
H	0.549524	-0.127599	2.109388
O	3.126462	0.086668	-0.611864
H	3.935779	-0.382176	-0.399111

$PIP\dot{C}^3$

T_1= 0.01158722, D_1= 0.03427274

$\tilde{\nu}$: 170.5, 223.5, 340.2, 383.4, 441.7, 480.6, 631.5, 775.8, 844.2, 872.7, 900.5, 929.3, 944.9, 1028.0, 1069.4, 1099.3, 1117.0, 1171.4, 1196.0, 1202.8, 1273.9, 1298.4, 1341.3, 1359.8, 1364.9, 1399.6, 1423.1, 1471.4, 1481.8, 1485.3, 1492.4, 1509.1, 2867.4, 2943.6, 2956.1, 3068.8, 3097.0, 3098.3, 3103.2, 3116.4, 3207.8, 3562.6

B: 4.801706 4.507795 2.582316

C	0.080678	-1.360242	0.251426
N	1.208510	-0.638732	-0.324674
C	1.253492	0.751762	0.126371
C	-0.034145	1.439805	-0.167637
C	-1.299811	0.727183	0.161043
C	-1.220548	-0.747406	-0.246535
H	0.090318	-1.328586	1.354252
H	0.148003	-2.405458	-0.049290
H	2.076223	-1.101190	-0.089303
H	2.081801	1.258210	-0.368710

H	1.446977	0.807519	1.217362
H	-0.033856	2.511469	-0.303826
H	-2.155638	1.216279	-0.304496
H	-1.474150	0.771893	1.248055
H	-2.072133	-1.297730	0.154941
H	-1.245112	-0.827887	-1.334275

$\mathrm{PRE}_{\mathrm{ax}}^{12\text{-}1}$

T_1= 0.01046675, D_1= 0.02595690

$\tilde{\nu}$: 28.3, 46.5, 79.2, 82.2, 245.6, 247.5, 286.9, 400.2, 437.8, 459.2, 557.6, 765.4, 814.5, 833.9, 868.3, 891.4, 927.8, 950.1, 1026.4, 1058.2, 1073.4, 1124.4, 1149.6, 1192.9, 1227.3, 1284.2, 1294.9, 1344.2, 1350.7, 1366.2, 1379.4, 1383.2, 1401.6, 1473.0, 1476.8, 1488.5, 1491.3, 1495.2, 1502.9, 3010.2, 3035.2, 3038.6, 3047.6, 3049.8, 3090.7, 3091.8, 3092.7, 3105.3, 3106.4, 3527.4, 3760.1

B: 2.603560   1.808229   1.802096

C	-0.560868	-1.208467	-0.693176
N	-1.181584	-0.000067	-1.226375
C	-0.560928	1.208401	-0.693263
C	-0.524126	1.255827	0.834607
C	0.145088	0.000060	1.387491
C	-0.524061	-1.255781	0.834697
H	0.464230	-1.242604	-1.074340
H	-1.084899	-2.075539	-1.095541
H	-2.170898	-0.000084	-1.004396
H	-1.085002	2.075419	-1.095691
H	0.464169	1.242561	-1.074427
H	-0.000955	2.153109	1.170737
H	-1.548424	1.316920	1.216203
H	0.136843	0.000099	2.478273
H	1.197417	0.000078	1.077727
H	-0.000844	-2.153012	1.170891
H	-1.548355	-1.316900	1.216299
O	2.783366	0.000027	-0.580824
H	3.330254	-0.000032	0.223341

$\mathrm{SP}_{\mathrm{ax}}^{12\text{-}1}$

T_1= 0.01986071, D_1= 0.10888845

$\tilde{\nu}$: -518.5, 49.0, 67.3, 137.5, 244.3, 246.1, 394.9, 435.2, 463.1, 552.5, 681.7, 774.7, 809.5, 838.1, 880.8, 894.0, 934.9, 947.0, 1022.2, 1056.6, 1074.3, 1111.0, 1145.9, 1186.7, 1225.0, 1246.4, 1277.4, 1336.8, 1343.5, 1349.7, 1371.5, 1376.8, 1384.4, 1414.3, 1465.6, 1481.3, 1489.5, 1493.7, 1502.0, 1638.4, 3025.7, 3029.4, 3055.4, 3056.5, 3092.5, 3096.0, 3099.3, 3110.7, 3111.3, 3527.6, 3780.8

B: 2.979927   1.941806   1.763176

C	-0.862114	-1.208792	-0.525288
N	-1.656890	0.000037	-0.712945
C	-0.862068	1.208830	-0.525248
C	-0.135492	1.261739	0.820315
C	0.687238	-0.000036	1.021894
C	-0.135541	-1.261773	0.820274
H	-0.119169	-1.240488	-1.326998
H	-1.510153	-2.076238	-0.648089
H	-2.437316	0.000041	-0.065851
H	-1.510075	2.076305	-0.648019
H	-0.119123	1.240525	-1.326957
H	0.498325	2.148181	0.875624
H	-0.873698	1.338269	1.626764
H	1.234475	-0.000062	1.964800
H	1.481224	-0.000036	0.194516
H	0.498241	-2.148242	0.875556
H	-0.873750	-1.338300	1.626720
O	2.488868	-0.000001	-0.934510
H	3.266170	-0.000020	-0.353057

$\mathrm{PRE}_{\mathrm{ax}}^{12\text{-}2}$ see PRE_{ax}^{8}

$\mathrm{SP}_{\mathrm{ax}}^{12\text{-}2}$

T_1= 0.02022389, D_1= 0.11114834

$\tilde{\nu}$: -579.3, 63.5, 88.7, 106.0, 232.0,253.8, 379.9, 434.3,464.5, 542.4, 698.9, 762.0, 806.7, 837.3, 873.3, 894.8, 929.0, 944.2, 1023.5, 1056.3, 1078.9, 1108.2, 1150.7, 1173.2, 1225.5, 1247.8,

C	0.836683	1.211301	-0.517161
N	1.620767	0.000009	-0.726855
C	0.836696	-1.211292	-0.517166
C	0.119059	-1.261155	0.832054
C	-0.697768	-0.000008	1.052125
C	0.119046	1.261149	0.832059
H	0.092431	1.265769	-1.318972

1273.2, 1331.2, 1341.2, 1351.6, 1367.9, 1379.1, 1389.8, 1425.4, 1465.0, 1480.6, 1485.8, 1493.9, 1498.9, 1564.4, 3025.7, 3029.5, 3038.0, 3040.2, 3096.5, 3099.7, 3102.5, 3107.7, 3108.4, 3533.0, 3781.8

B:2.951483, 1.979459, 1.801703

H	1.490198	2.074493	-0.639766
H	2.422961	0.000012	-0.107643
H	1.490220	-2.074477	-0.639777
H	0.092444	-1.265764	-1.318978
H	-0.515767	-2.146210	0.892406
H	0.867310	-1.342877	1.628456
H	-1.214318	-0.000012	2.012228
H	-1.561619	-0.000009	0.288935
H	-0.515791	2.146197	0.892417
H	0.867295	1.342876	1.628462
O	-2.532160	0.000001	-0.844129
H	-1.885753	-0.000044	-1.568216

$POST_{ax}^{12}$

T_1= 0.01216054, D_1= 0.04401320

$\tilde{\nu}$: 32.1, 73.3, 109.5, 128.9, 209.7, 229.6, 230.4, 354.2, 368.4, 416.9, 468.1, 496.2, 645.7, 788.3, 798.1, 841.2, 898.4, 920.4, 970.2, 1012.8, 1036.7, 1098.8, 1113.9, 1135.1, 1174.5, 1246.7, 1264.4, 1329.8, 1333.6, 1348.4, 1372.5, 1402.7, 1403.0, 1458.6, 1470.2, 1490.9, 1496.8, 1504.6, 1614.2, 2961.6, 2963.9, 3064.6, 3065.3, 3088.7, 3089.4, 3115.8, 3116.0, 3184.7, 3526.7, 3773.1, 3932.7

B: 2.663541, 1.834556, 1.779434

C	-0.639065	-1.206749	-0.665425
N	-1.329510	0.000035	-1.099766
C	-0.639032	1.206783	-0.665379
C	-0.434845	1.276275	0.857260
C	0.136100	-0.000033	1.374959
C	-0.434880	-1.276305	0.857211
H	0.335408	-1.222370	-1.159198
H	-1.196543	-2.076607	-1.011507
H	-2.281324	0.000041	-0.750186
H	-1.196488	2.076670	-1.011426
H	0.335441	1.222398	-1.159152
H	0.192988	2.128104	1.123593
H	-1.416955	1.453558	1.322926
H	0.689627	-0.000058	2.304925
H	2.083561	-0.000021	0.145330
H	0.192929	-2.128162	1.123512
H	-1.416995	-1.453579	1.322870
O	2.685742	0.000005	-0.608791
H	3.569315	-0.000091	-0.234751

$PRE_{ax}^{13\text{-}1}$

T_1= 0.01021385, D_1= 0.02502547

$\tilde{\nu}$: 35.4, 75.5, 109.2, 143.9, 247.3, 254.0, 271.2, 400.8, 443.2, 459.0, 561.2, 764.7, 812.6, 833.2, 878.6, 893.1, 929.8, 955.6, 1026.2, 1059.9, 1076.2, 1128.3, 1151.3, 1195.1, 1230.1, 1288.2, 1304.7, 1344.1, 1352.2, 1374.2, 1379.4, 1385.0, 1402.8, 1470.9, 1486.0, 1489.4, 1493.2, 1496.1, 1510.8, 3015.4, 3032.8, 3037.8, 3043.2, 3045.3, 3070.2, 3078.5, 3096.7, 3107.2, 3111.0, 3532.8, 3760.0

B: 4.192192   1.403053   1.154084

C	1.891624	-0.471072	0.494796
N	1.794165	0.917860	0.060342
C	0.425177	1.418123	0.126579
C	-0.584992	0.547155	-0.620368
C	-0.498433	-0.898305	-0.131853
C	0.935768	-1.414254	-0.236110
H	1.667081	-0.493818	1.566000
H	2.924108	-0.800302	0.379039
H	2.142379	1.003350	-0.887765
H	0.409239	2.439712	-0.252056
H	0.144358	1.464424	1.183442
H	-1.592719	0.951668	-0.489475
H	-0.365593	0.578876	-1.691868
H	-1.173798	-1.542244	-0.700953
H	-0.806855	-0.939287	0.919987
H	1.015153	-2.423042	0.172772
H	1.223385	-1.468603	-1.290428
O	-3.524321	0.056388	0.310830
H	-2.966190	-0.736733	0.384004

$SP_{ax}^{13\text{-}1}$

T_1= 0.02019288, D_1= 0.11195169

C	-0.111227	-1.414004	1.208155
N	-0.713777	-1.960313	-0.000000
C	-0.111227	-1.414004	-1.208155

$\tilde{\nu}$: -449.8, 40.6, 55.9, 103.8, 238.8, 261.4, 396.1, 431.4, 456.8, 552.1, 644.0, 777.8, 814.9, 834.9, 889.1, 920.4, 927.4, 953.4, 1011.4, 1059.5, 1067.9, 1118.6, 1136.3, 1188.2, 1213.2, 1269.1, 1278.1, 1323.9, 1326.5, 1353.2, 1353.8, 1378.4, 1379.4, 1405.6, 1474.1, 1488.0, 1492.7, 1497.5, 1500.0, 1728.4, 3042.6, 3045.2, 3049.1, 3052.4, 3061.1, 3104.2, 3105.9, 3109.1, 3110.2, 3536.0, 3787.0

B: 4.446871 1.466720 1.186591

C	-0.111227	0.120659	-1.255489
C	0.542361	0.669032	0.000000
C	-0.111227	0.120659	1.255489
H	0.921283	-1.773504	1.251944
H	-0.630220	-1.822607	2.075050
H	-1.709845	-1.773312	-0.000000
H	-0.630220	-1.822607	-2.075050
H	0.921283	-1.773504	-1.251944
H	0.403581	0.471133	-2.150982
H	-1.143947	0.475530	-1.312198
H	0.440304	1.805464	0.000000
H	1.615668	0.457195	0.000000
H	0.403581	0.471133	2.150982
H	-1.143947	0.475530	1.312198
O	0.444372	3.326084	0.000000
H	1.409213	3.429022	0.000000

$\mathrm{PRE}_{ax}^{13\text{-}2}$

T_1= 0.01025117, D_1= 0.02516600

$\tilde{\nu}$: 49.2, 80.2, 113.7, 147.5, 244.1, 261.0, 286.8, 401.7, 440.3, 458.5, 559.7, 769.2, 813.5, 833.5, 873.5, 889.7, 929.5, 952.7, 1028.5, 1059.1, 1070.5, 1133.3, 1151.3, 1192.8, 1228.3, 1292.8, 1300.5, 1343.9, 1349.1, 1375.7, 1384.3, 1388.4, 1401.7, 1470.0, 1485.6, 1488.6, 1492.9, 1496.8, 1510.1, 3011.8, 3034.3, 3035.9, 3040.8, 3043.0, 3069.3, 3092.3, 3095.3, 3108.0, 3109.4, 3530.1, 3756.7

B: 3.121282 1.694256 1.519431

C	-1.050423	1.344808	0.003178
N	-1.491707	0.319467	-0.935460
C	-1.442151	-1.015178	-0.349442
C	-0.077418	-1.378919	0.236972
C	0.363301	-0.314232	1.241127
C	0.328437	1.075019	0.606373
H	-1.790298	1.390019	0.808959
H	-1.069049	2.310289	-0.501696
H	-0.910115	0.347358	-1.765504
H	-1.742828	-1.739001	-1.106638
H	-2.194631	-1.051143	0.444854
H	-0.120343	-2.362115	0.709217
H	0.647619	-1.440754	-0.581000
H	1.354269	-0.536530	1.647074
H	-0.320032	-0.329874	2.096043
H	0.577794	1.842673	1.340924
H	1.077031	1.141661	-0.192296
O	2.932598	0.051674	-0.840572
H	2.741274	-0.491238	-0.056387

$\mathrm{SP}_{\mathrm{ax}}^{13\text{-}2}$

T_1= 0.02019288, D_1= 0.11195169

$\tilde{\nu}$: -581.5, 51.3, 70.7, 95.2, 239.1, 239.8, 378.0, 426.2, 457.6, 554.8,
646.1, 765.4, 816.8, 834.6, 866.3, 900.7, 928.5, 960.0, 1010.0, 1041.8,
1099.9, 1127.3, 1143.7, 1174.0, 1224.3, 1275.9, 1284.4, 1324.6, 1334.9, 1356.0,
1370.3, 1374.5, 1389.0, 1397.2, 1474.4, 1488.3, 1491.8, 1496.9, 1499.4, 1717.4,
3039.2, 3045.4, 3048.1, 3052.3, 3064.7, 3093.7, 3107.5, 3109.5, 3111.3, 3535.0,
3785.5

B: 4.338549 1.532823 1.232950

C	-1.294287	1.267197	0.149781
N	-2.000836	0.122217	-0.407048
C	-1.490426	-1.140939	0.108993
C	0.025021	-1.309192	-0.067613
C	0.745621	-0.119946	0.540825
C	0.228971	1.191080	-0.023985
H	-1.530013	1.312175	1.217197
H	-1.686548	2.177107	-0.304016
H	-1.928044	0.133196	-1.417851
H	-2.024053	-1.958738	-0.374894
H	-1.734299	-1.183219	1.174669
H	0.357596	-2.242379	0.388271
H	0.256811	-1.365200	-1.134907
H	1.862900	-0.228242	0.320876
H	0.668338	-0.130363	1.631691
H	0.703122	2.044968	0.464326
H	0.466227	1.245520	-1.090997
O	3.258878	-0.106044	-0.241337
H	3.233394	0.858807	-0.342332

$POST^{13}_{ax}$

T_1= 0.02019288, D_1 0.11195169

$\tilde{\nu}$: 45.3, 57.2, 115.1, 124.2, 227.1, 228.8, 257.1, 348.7, 354.2, 443.9, 457.7, 507.6, 634.4, 787.1, 809.4, 846.9, 889.2, 926.7, 971.7, 984.9, 1021.6, 1110.2, 1137.5, 1144.2, 1178.6, 1241.1, 1290.0, 1299.1, 1313.8, 1340.6, 1355.1, 1392.2, 1398.4, 1472.9, 1487.9, 1488.2, 1498.4, 1498.9, 1616.7, 3020.7, 3022.9, 3054.4, 3056.1, 3107.3, 3108.7, 3109.8, 3111.3, 3190.6, 3536.0, 3782.7, 3937.8

B: 3.557111 1.533707 1.317360

C	1.373441	-1.225544	-0.063957
N	1.744261	-0.032276	-0.807325
C	1.420228	1.193594	-0.096178
C	-0.068714	1.278064	0.322284
C	-0.440616	0.034097	1.051465
C	-0.117683	-1.241785	0.354673
H	1.996068	-1.266460	0.833304
H	1.605906	-2.103687	-0.666341
H	1.287519	-0.035640	-1.712164
H	1.685511	2.045914	-0.721730
H	2.044891	1.234575	0.799675
H	-0.243680	2.171889	0.921002
H	-0.663071	1.367523	-0.594726
H	-2.536161	0.009785	0.153055
H	-0.517568	0.049215	2.130641
H	-0.327871	-2.112066	0.976084
H	-0.714526	-1.333675	-0.560494
O	-3.106799	-0.035317	-0.623892
H	-3.972383	0.250532	-0.325624

PIPĊ[4]

T_1= 0.01174001, D_1= 0.03582035

$\tilde{\nu}$: 186.8, 235.2, 359.2, 400.3, 456.8, 475.4, 621.5, 783.8, 808.6, 848.0, 909.1, 921.4, 1007.4, 1048.9, 1063.5, 1084.9, 1106.3, 1181.1, 1189.4, 1204.6, 1257.2, 1322.5, 1333.9, 1347.1, 1372.1, 1400.3, 1420.3, 1460.2, 1469.2, 1482.9, 1501.1, 1511.5, 2962.9, 2964.1, 2985.5, 2986.6, 3099.6, 3100.0, 3103.9, 3104.7, 3204.6, 3568.2

B: 4.726136 4.595880 2.592119

C	0.032376	-0.756088	1.206027
N	-0.666044	-1.183023	0.000000
C	0.032376	-0.756088	-1.206027
C	0.032376	0.770965	-1.275732
C	0.534491	1.353904	-0.000000
C	0.032376	0.770965	1.275732
H	1.075361	-1.108655	1.219522
H	-0.476591	-1.173859	2.074134
H	-0.781580	-2.187055	0.000000
H	-0.476591	-1.173859	-2.074134
H	1.075361	-1.108655	-1.219522
H	0.626670	1.109947	-2.124600
H	-1.002757	1.094780	-1.454394
H	1.014558	2.321835	-0.000000
H	0.626670	1.109947	2.124600
H	-1.002757	1.094780	1.454394

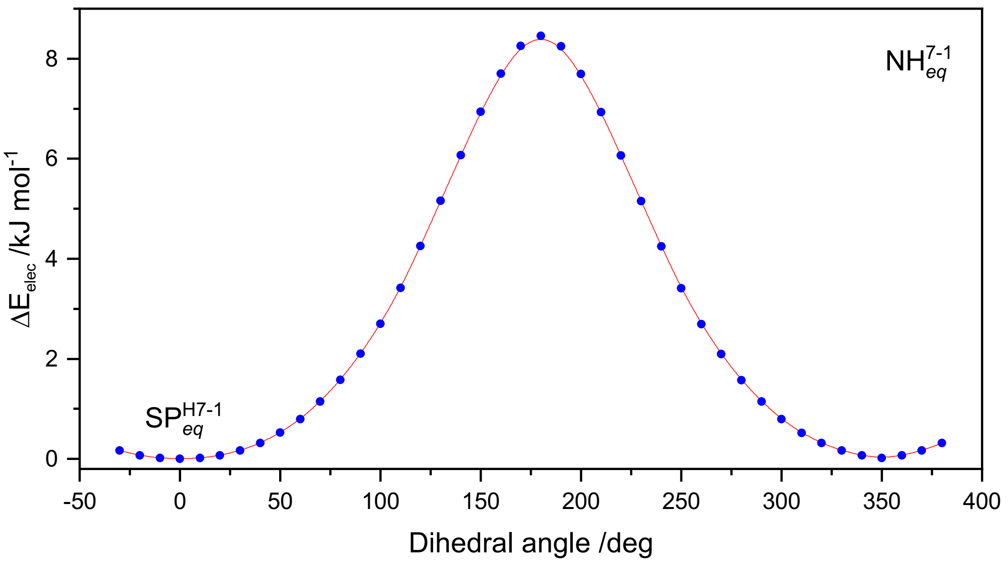

NH_{eq}^{7-1}
SP_{eq}^{H7-1}
ΔE_{elec} /kJ mol^{-1}
Dihedral angle /deg

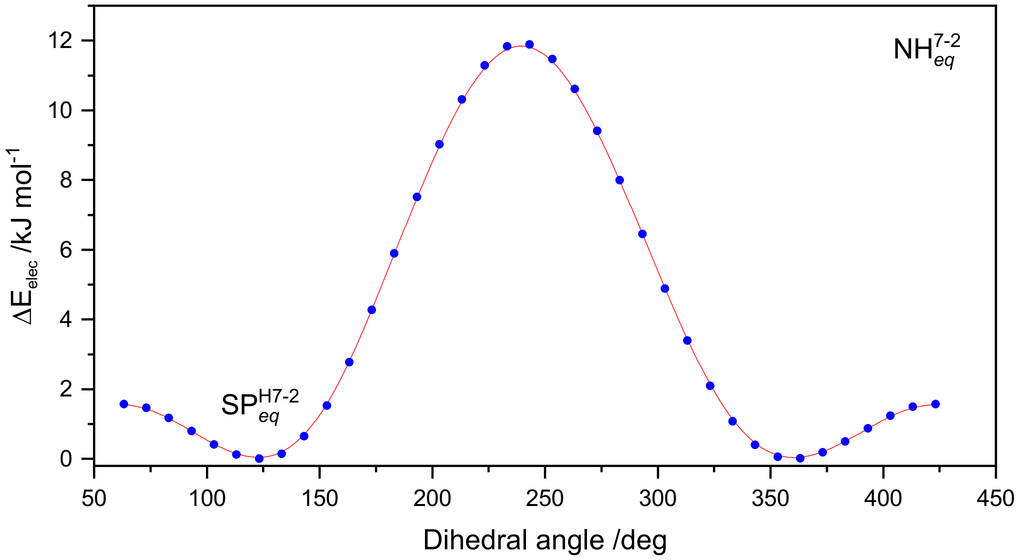

NH_{eq}^{7-2}
SP_{eq}^{H7-2}
ΔE_{elec} /kJ mol^{-1}
Dihedral angle /deg

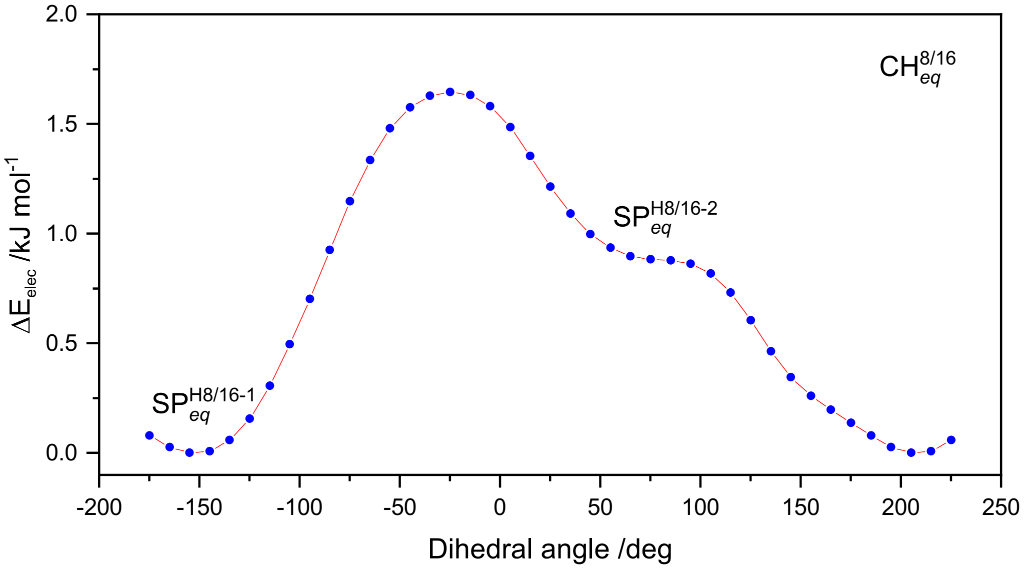

$CH_{eq}^{8/16}$
$SP_{eq}^{H8/16-1}$
$SP_{eq}^{H8/16-2}$
ΔE_{elec} /kJ mol^{-1}
Dihedral angle /deg

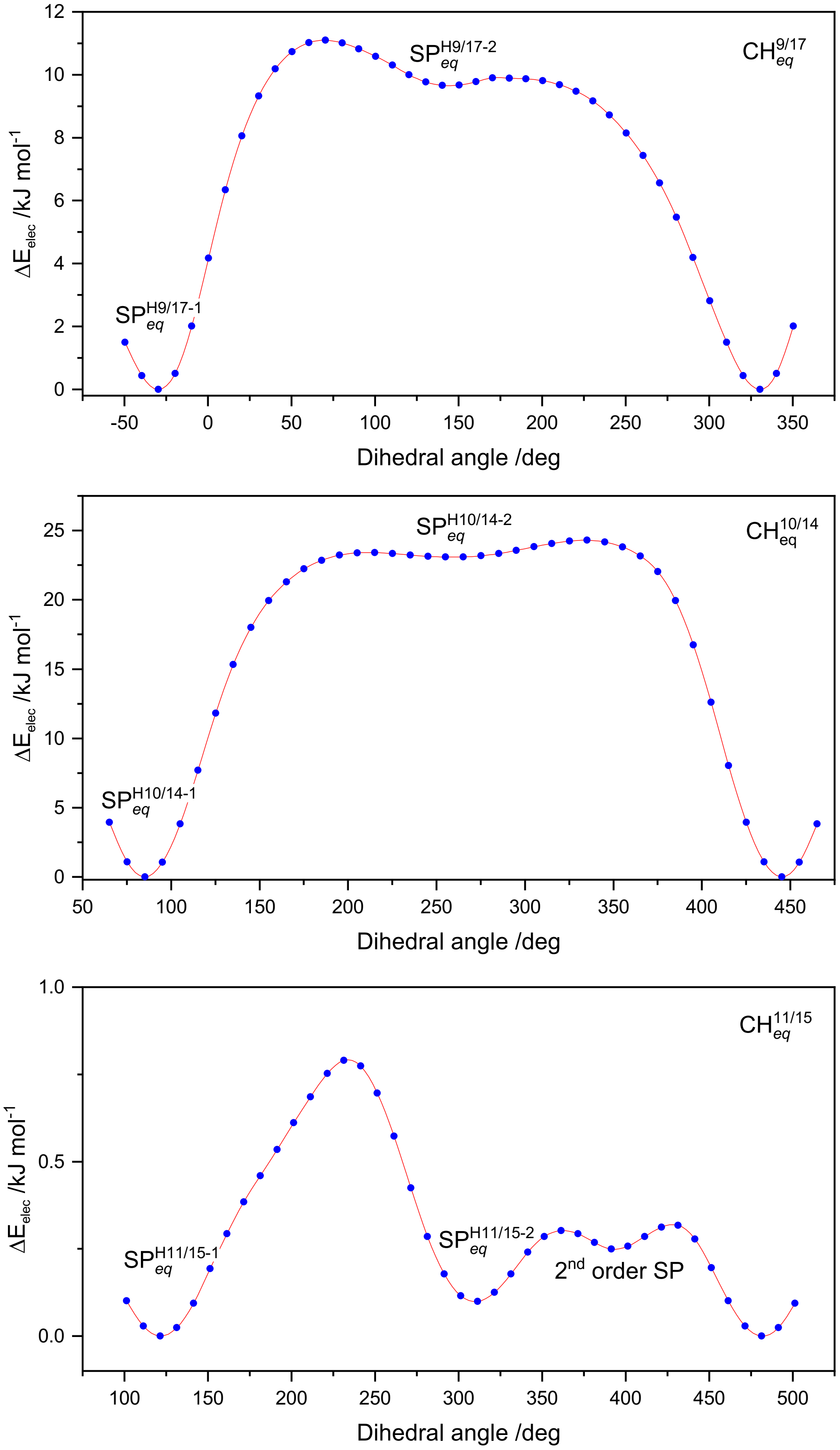

12
10
8
6
4
2
0
ΔE$_{elec}$ /kJ mol^{-1}
-50
0
50
100
150
200
250
300
350
Dihedral angle /deg
SP$_{eq}^{H9/17-1}$
SP$_{eq}^{H9/17-2}$
CH$_{eq}^{9/17}$
25
20
15
10
5
0
ΔE$_{elec}$ /kJ mol^{-1}
50
100
150
200
250
300
350
400
450
Dihedral angle /deg
SP$_{eq}^{H10/14-1}$
SP$_{eq}^{H10/14-2}$
CH$_{eq}^{10/14}$
1.0
0.5
0.0
ΔE$_{elec}$ /kJ mol^{-1}
100
150
200
250
300
350
400
450
500
Dihedral angle /deg
SP$_{eq}^{H11/15-1}$
SP$_{eq}^{H11/15-2}$
2nd order SP
CH$_{eq}^{11/15}$

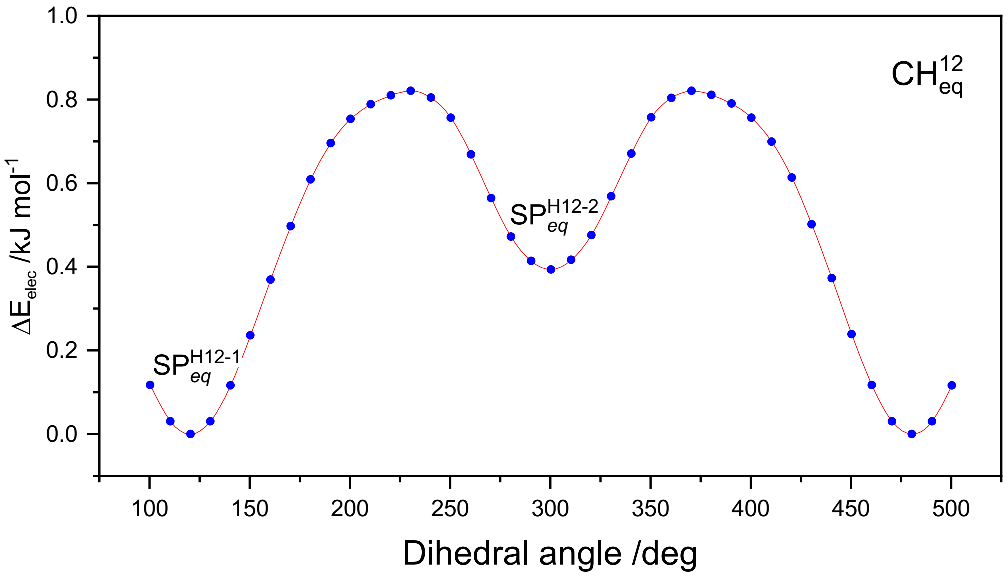
CH$^{12}_{eq}$
SP$^{H12-1}_{eq}$
SP$^{H12-2}_{eq}$
ΔE$_{elec}$ /kJ mol^{-1}
Dihedral angle /deg

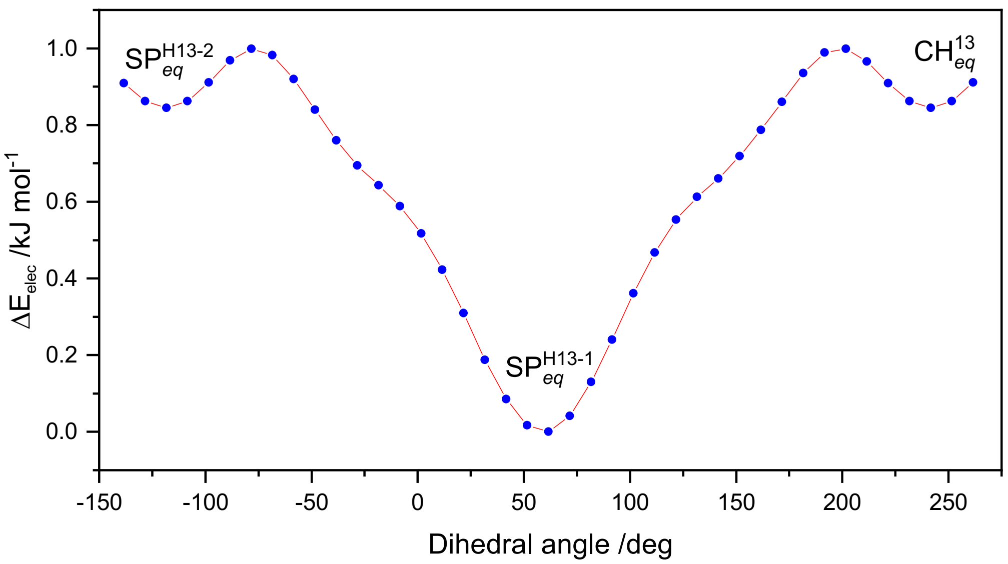
CH$^{13}_{eq}$
SP$^{H13-2}_{eq}$
SP$^{H13-1}_{eq}$
ΔE$_{elec}$ /kJ mol^{-1}
Dihedral angle /deg

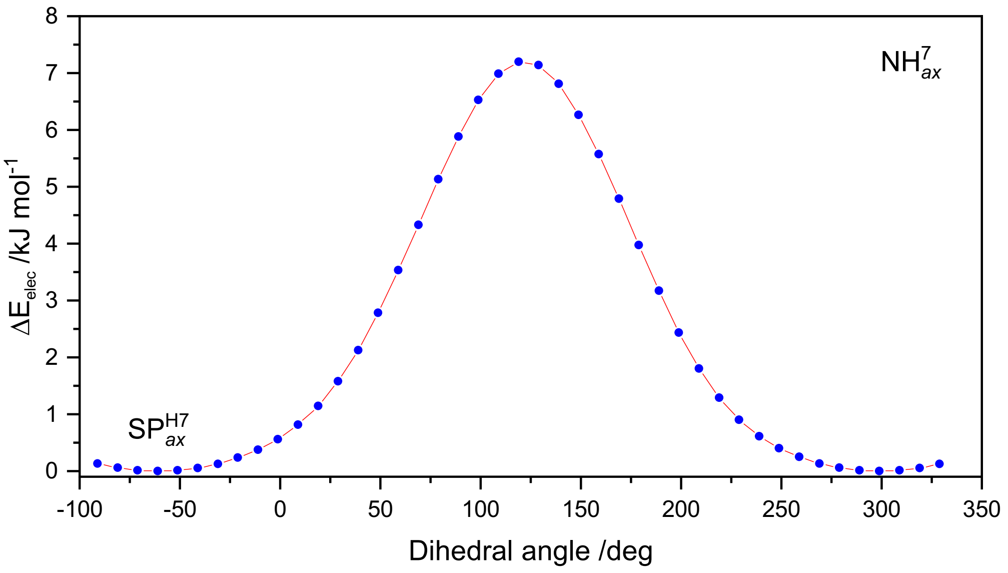
NH$^{7}_{ax}$
SP$^{H7}_{ax}$
ΔE$_{elec}$ /kJ mol^{-1}
Dihedral angle /deg

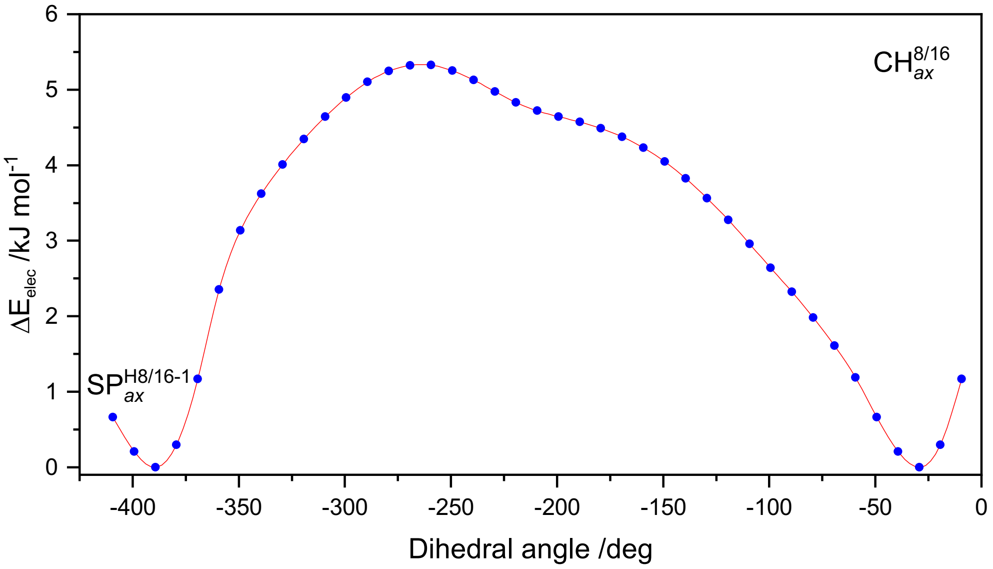

$CH_{ax}^{8/16}$
$SP_{ax}^{H8/16-1}$
ΔE_{elec} /kJ mol^{-1}
Dihedral angle /deg
0
1
2
3
4
5
6
-400
-350
-300
-250
-200
-150
-100
-50
0

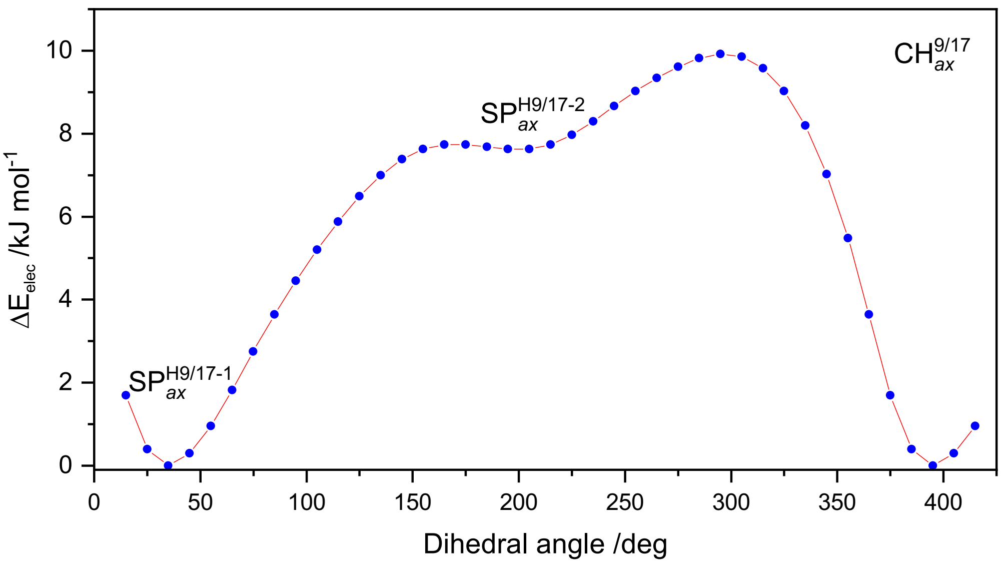

$CH_{ax}^{9/17}$
$SP_{ax}^{H9/17-2}$
$SP_{ax}^{H9/17-1}$
ΔE_{elec} /kJ mol^{-1}
Dihedral angle /deg
0
2
4
6
8
10
0
50
100
150
200
250
300
350
400

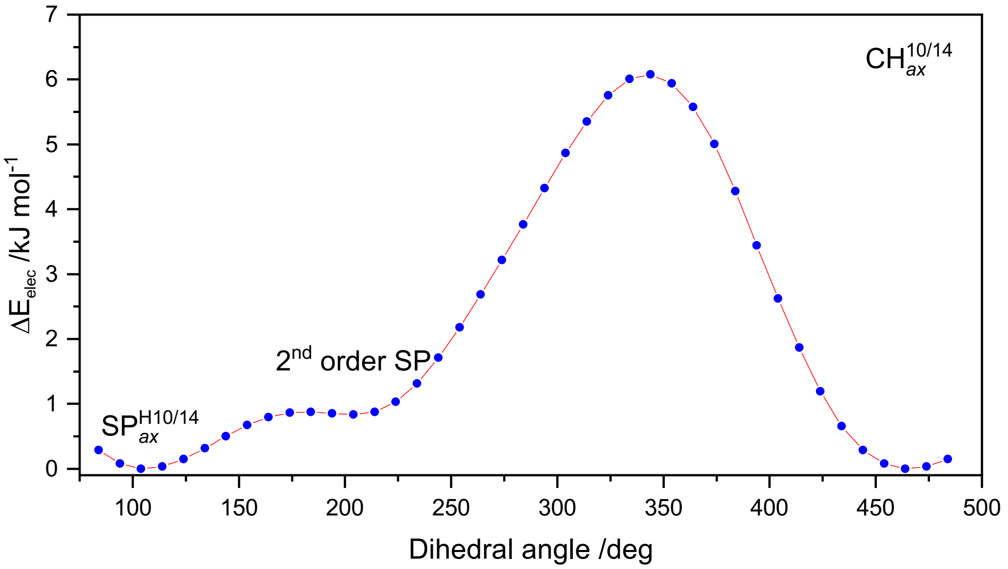

$CH_{ax}^{10/14}$
2nd order SP
$SP_{ax}^{H10/14}$
ΔE_{elec} /kJ mol^{-1}
Dihedral angle /deg
0
1
2
3
4
5
6
7
100
150
200
250
300
350
400
450
500

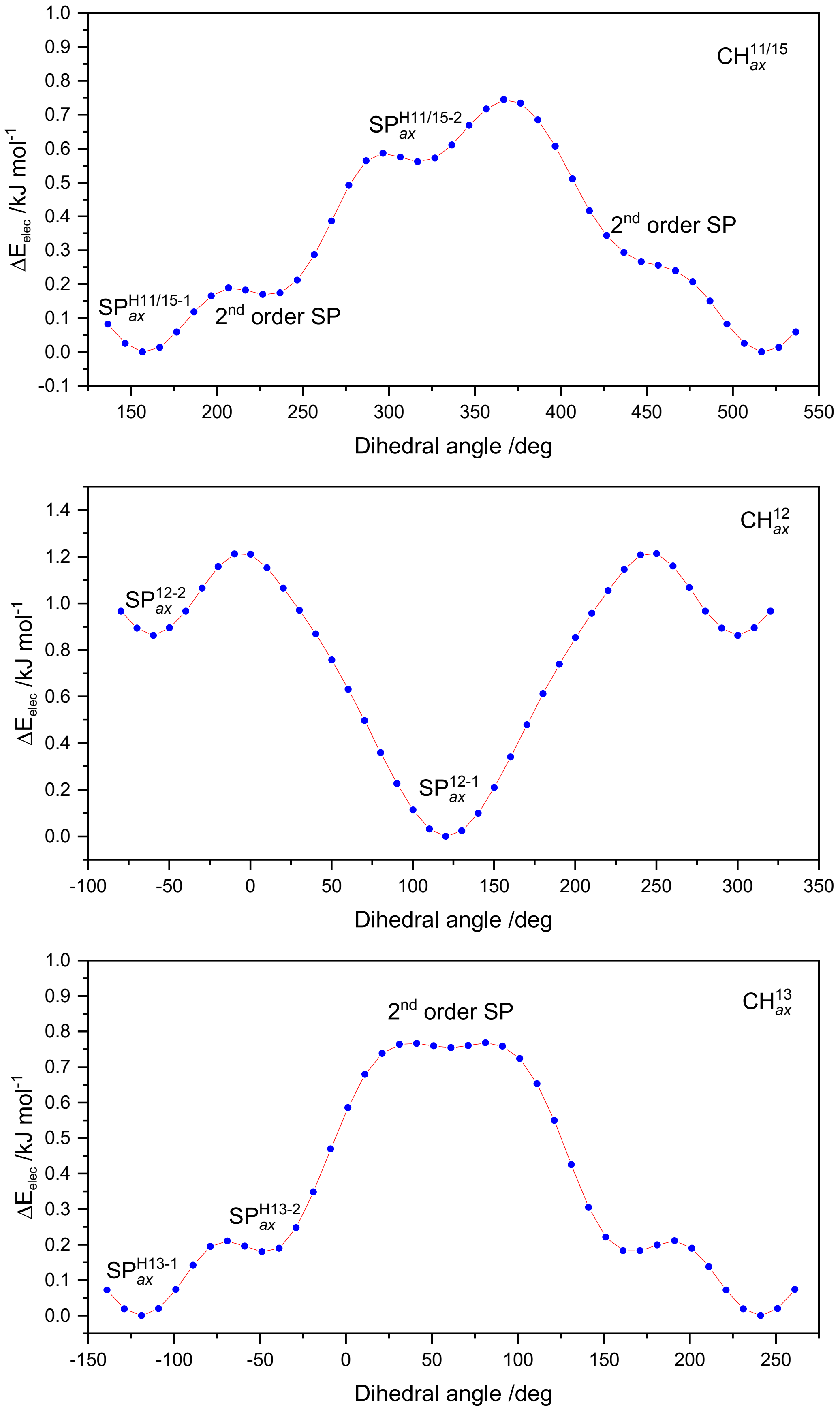

Figure S1. Rotation potential energies of the CH/NH-abstraction saddle points in the piperidine + OH reaction. Results from CCSD(T*)/M06-2X/6-31+G(d,p) calculations.

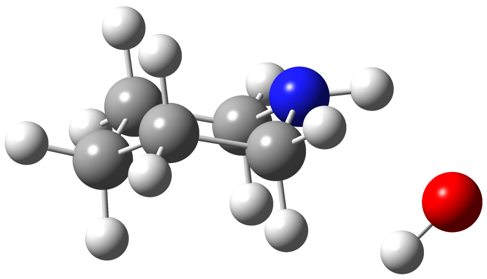

PRE$_{\mathrm{eq}}^{7-1}$

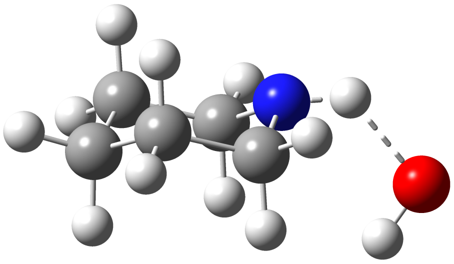

SP$_{eq}^{7-1}$

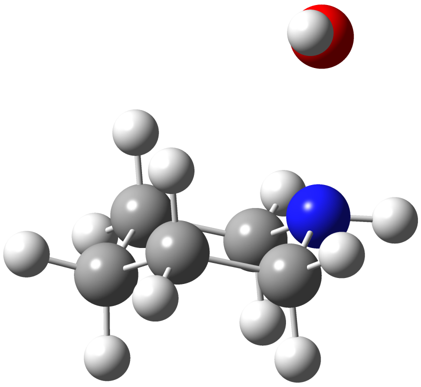

PRE$_{\mathrm{eq}}^{7-2}$

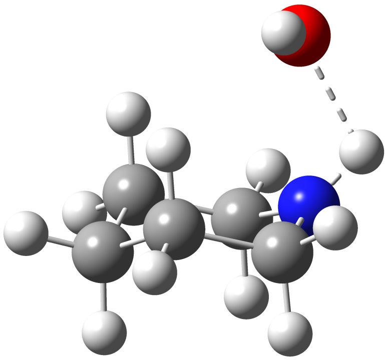

SP$_{eq}^{7-2}$

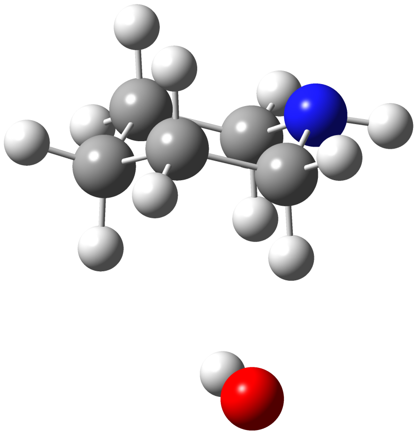

PRE$_{eq}^{8-1}$

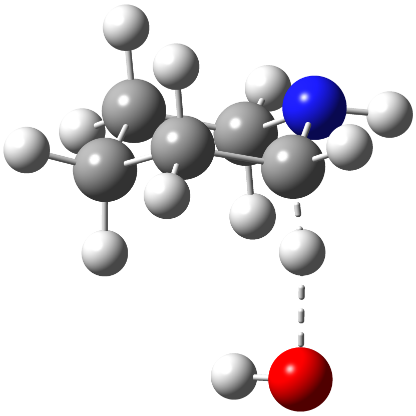

SP$_{eq}^{8-1}$

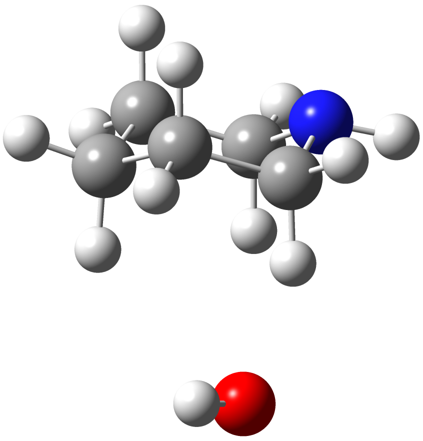

PRE$_{eq}^{8-2}$

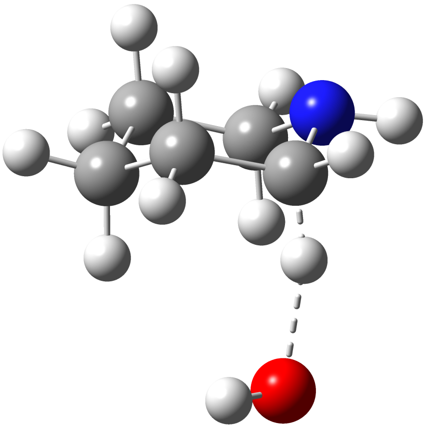

SP$_{eq}^{8-2}$

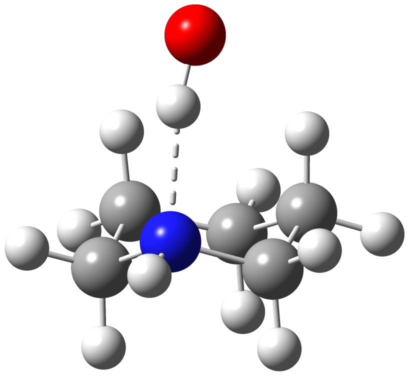	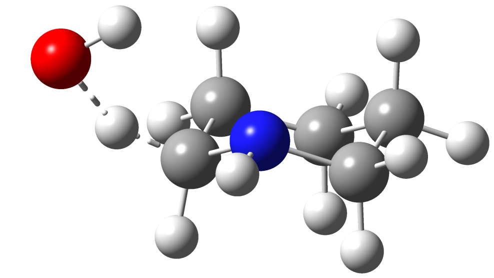
PRE$_{eq}^{9\text{-}1}$	SP$_{eq}^{9\text{-}1}$
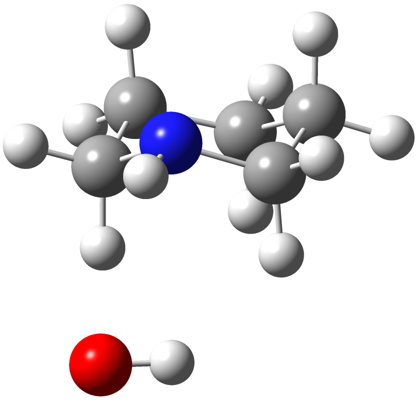	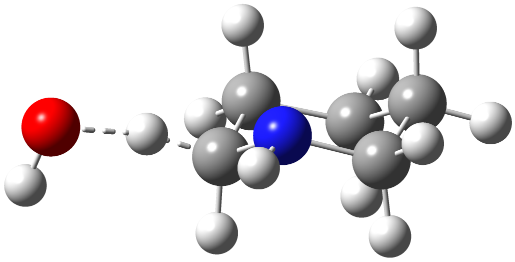
PRE$_{eq}^{9\text{-}2}$	SP$_{eq}^{9\text{-}2}$
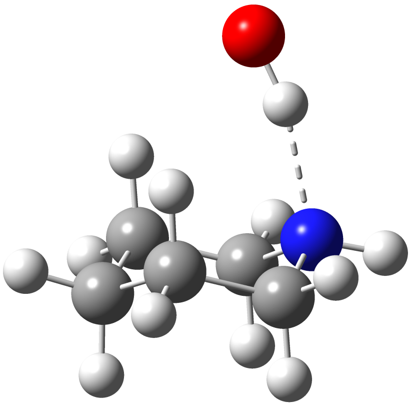	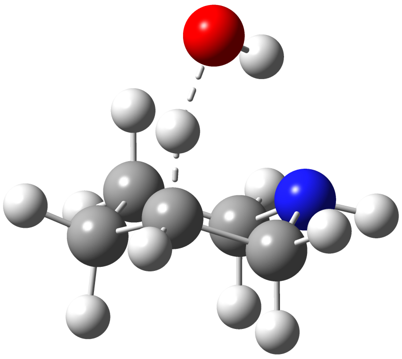
PRE$_{eq}^{10\text{-}1}$ (same as PRE$_{eq}^{9\text{-}1}$)	SP$_{eq}^{10\text{-}1}$
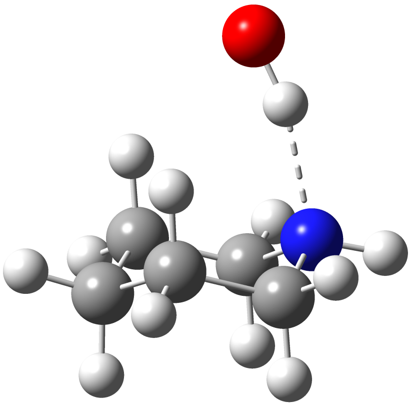	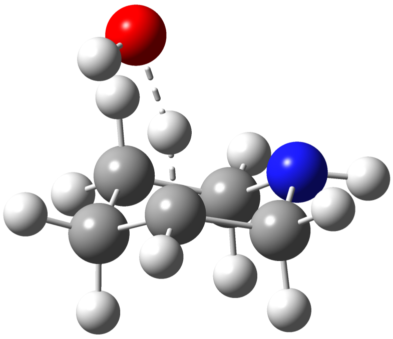
PRE$_{eq}^{10\text{-}2}$(same as PRE$_{eq}^{9\text{-}1}$ and PRE$_{eq}^{10\text{-}1}$)	SP$_{eq}^{10\text{-}2}$

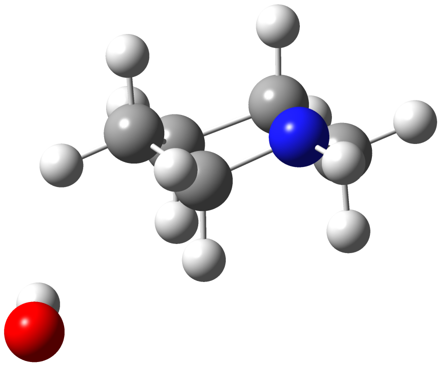<br>$PRE_{eq}^{11\text{-}1}$	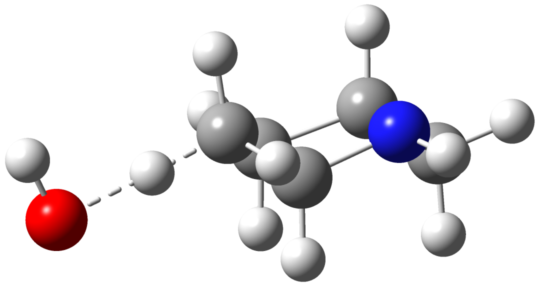<br>$SP_{eq}^{11\text{-}1}$
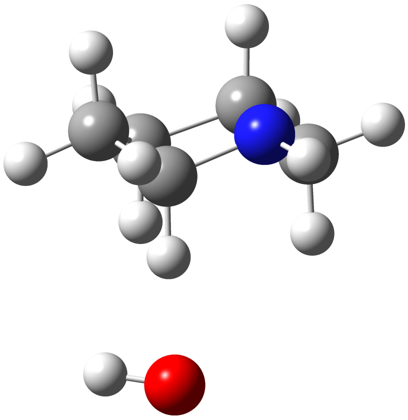<br>$PRE_{eq}^{11\text{-}2}$	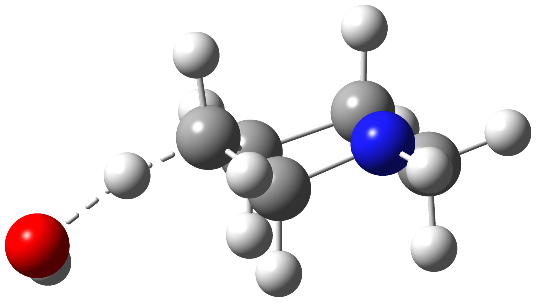<br>$SP_{eq}^{11\text{-}2}$
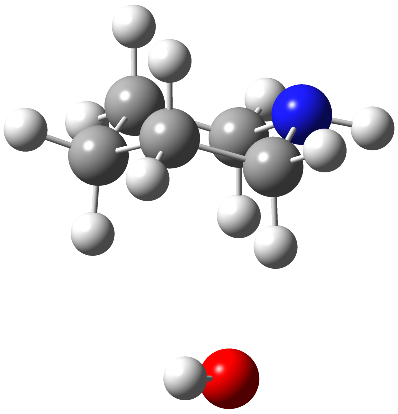<br>$PRE_{eq}^{12\text{-}1}$ (same as PRE_{eq}^{8-2})	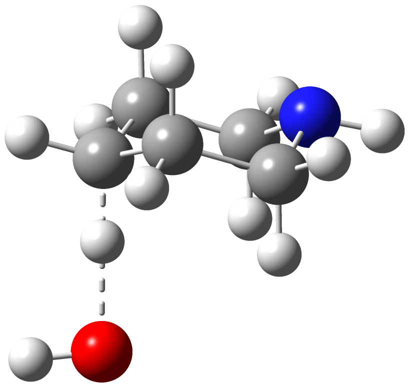<br>$SP_{eq}^{12\text{-}1}$
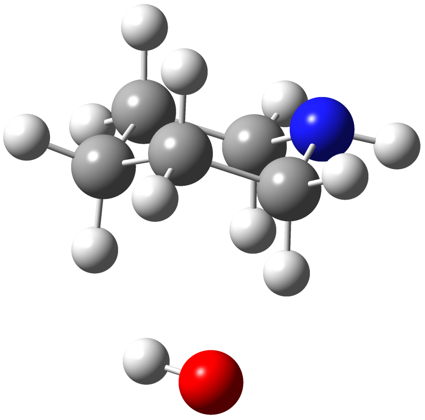<br>$PRE_{eq}^{12\text{-}2}$	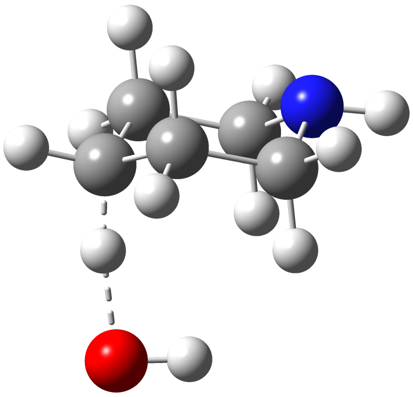<br>$SP_{eq}^{12\text{-}2}$

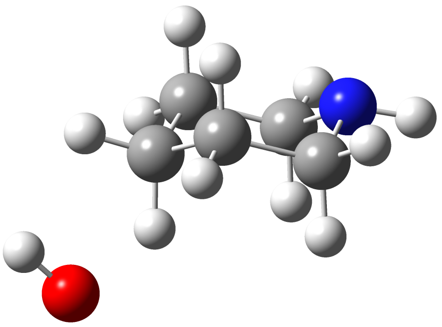

PRE$_{eq}^{13\text{-}1}$

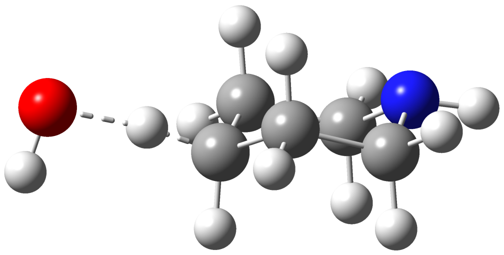

SP$_{eq}^{13\text{-}1}$

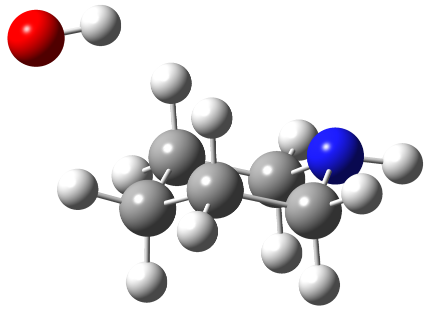

PRE$_{eq}^{13\text{-}2}$

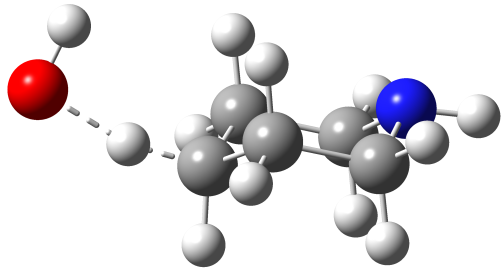

SP$_{eq}^{13\text{-}2}$

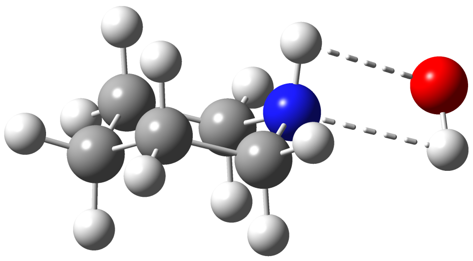

PRE$_{ax}^{7}$

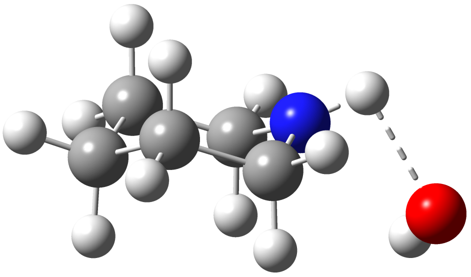

SP$_{ax}^{7}$

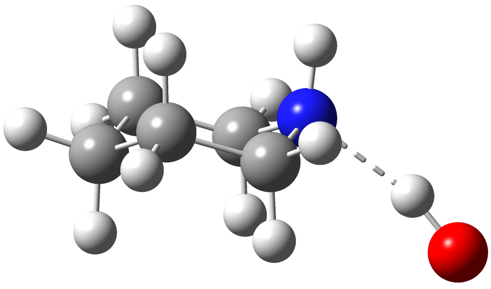

PRE$_{ax}^{8}$

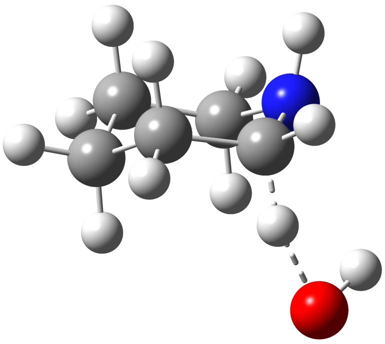

SP$_{ax}^{8}$

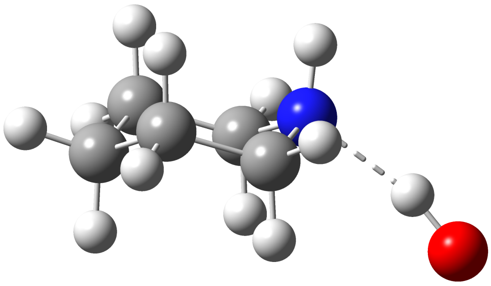

PRE$_{ax}^{9\text{-}1}$ (same as PRE$_{ax}^{8}$)

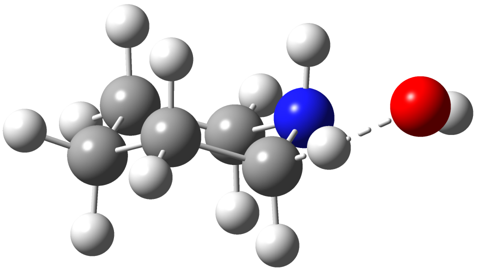

SP$_{ax}^{9\text{-}1}$

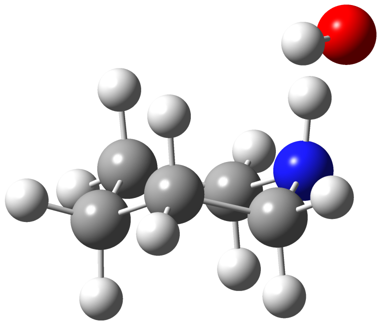	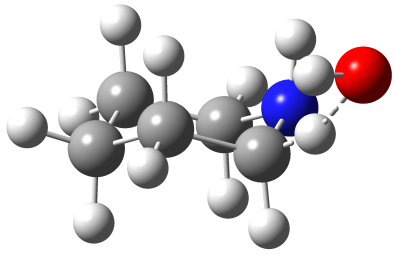
PRE$_{ax}^{9\text{-}2}$	SP$_{ax}^{9\text{-}2}$
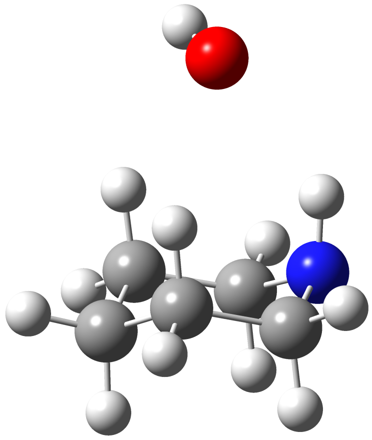	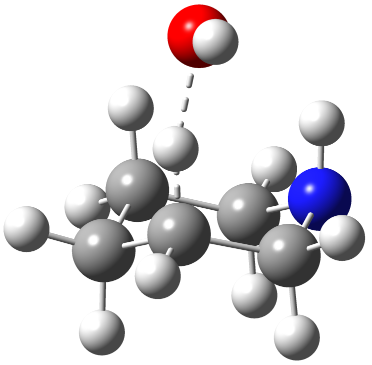
PRE$_{ax}^{10}$	SP$_{ax}^{10}$
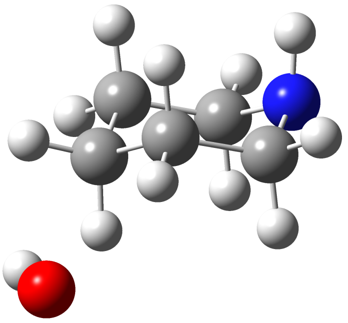	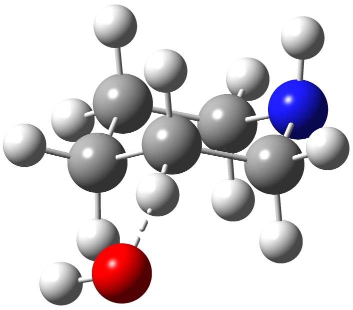
PRE$_{ax}^{11\text{-}1}$	SP$_{\mathrm{ax}}^{11\text{-}1}$
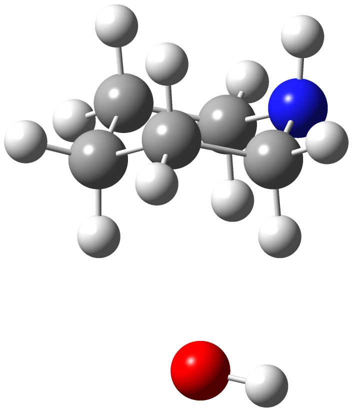	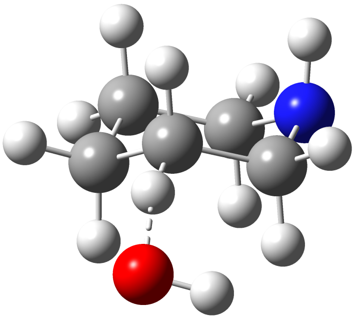
PRE$_{ax}^{11\text{-}2}$	SP$_{\mathrm{ax}}^{11\text{-}2}$

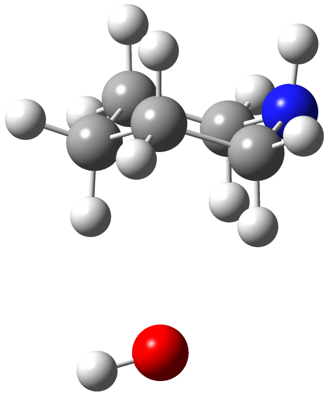

$\mathrm{PRE}_{ax}^{12\text{-}1}$

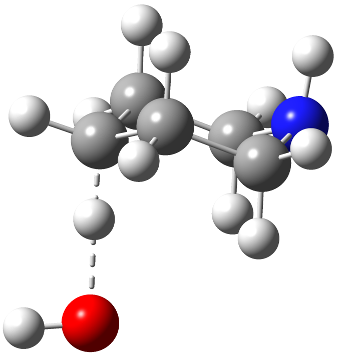

$\mathrm{SP}_{\mathrm{ax}}^{12\text{-}1}$

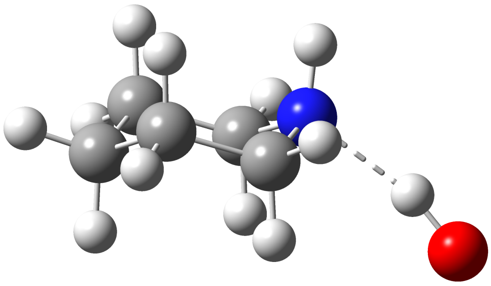

$\mathrm{PRE}_{ax}^{12\text{-}2}$ (same as PRE_{ax}^{8} and $\mathrm{PRE}_{ax}^{9\text{-}1}$)

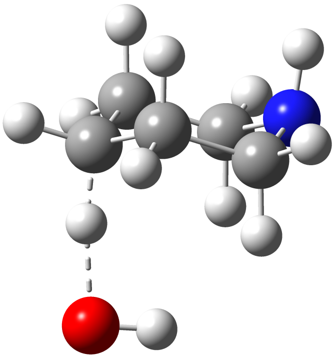

$\mathrm{SP}_{\mathrm{ax}}^{12\text{-}2}$

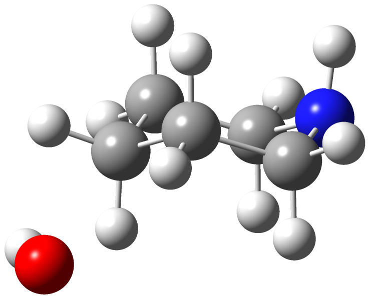

$\mathrm{PRE}_{ax}^{13\text{-}1}$

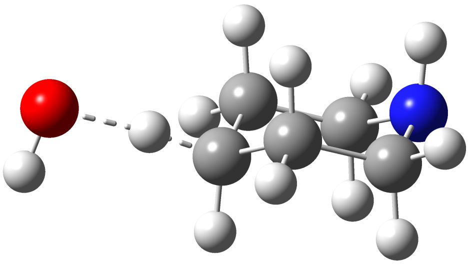

$\mathrm{SP}_{\mathrm{ax}}^{13\text{-}1}$

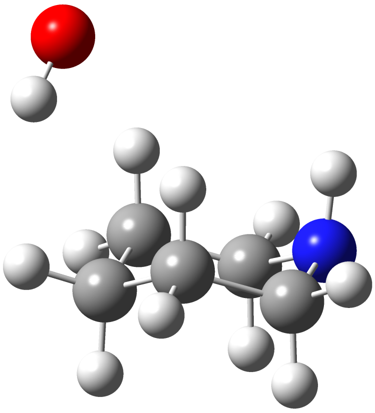

$\mathrm{PRE}_{ax}^{13\text{-}2}$

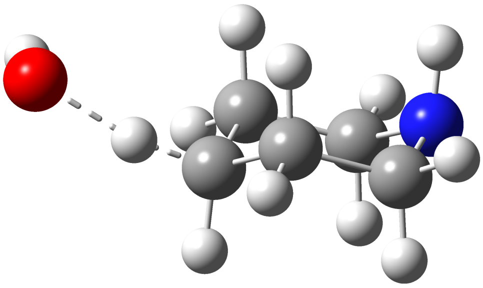

$\mathrm{SP}_{\mathrm{ax}}^{13\text{-}2}$

Figure S2. Structures of pre-reaction complexes/adducts and saddle points in the piperidine + OH reaction
Results from M062X/aug-cc-pVTZ calculations. The $\mathrm{PRE}_{ax}^{9\text{-}2}$ and PRE_{ax}^{10} structures are those of the IRC end points, see main text.

Table S5. Descriptors of saddle points in the piperidine + OH reaction.

Relative saddle point energies including ZPE (ΔE_{DFT}, $\Delta E_{CC//DFT}$, ΔE_{MP2} and $\Delta E_{CC//MP2}$) in kJ mol^{-1}, frequencies ($\tilde{\upsilon}$) in cm^{-1}, C-H and N-H bond elongations ($\Delta r_{X\text{-}H}$) and H··OH distances ($\Delta r_{X\text{-}H}$ and $r_{XH\cdot\cdot OH}$) in pm. Numbering of atoms according to Figure 1 in the main text.

	M062X/aug-cc-pVTZ					ωB97XD/aug-cc-pVTZ					BMK/aug-cc-pVTZ					MP2/aug-cc-pVTZ				
PIP_{eq}	ΔE_{DFT}	$\Delta E_{CC//DFT}$	$\tilde{\upsilon}$	$\Delta r_{X\text{-}H}$	$r_{XH\cdot\cdot OH}$	ΔE_{DFT}	$\Delta E_{CC//DFT}$	$\tilde{\upsilon}$	$\Delta r_{X\text{-}H}$	$r_{XH\cdot\cdot OH}$	ΔE_{DFT}	$\Delta E_{CC//DFT}$	$\tilde{\upsilon}$	$\Delta r_{X\text{-}H}$	$r_{XH\cdot\cdot OH}$	ΔE_{MP2}	$\Delta E_{CC//MP2}$	$\tilde{\upsilon}$ [a]	$\Delta r_{X\text{-}H}$	$r_{XH\cdot\cdot OH}$
NH^{7-1}	-13.7	-12.6	-318	1.3	169.4	-19.0	-4.5	-511	-0.9	189.2	-12.4	-4.7	-719	-0.6	191.9	4.1	-25.8	-1565	5.6	142.0
NH^{7-2}	-11.0	-7.8	-487	0.1	177.5	-22.7	-6.6	-491	-0.6	188.2	-13.8	-5.7	-566	-0.3	-182.8	CP[b]		-533		
CH^{8-1}	-10.6	-8.4	-264	2.6	170.0			Not located					Not located			1.8	-16.0	-653	4.5	154.4
CH^{8-2}	-10.6	-8.1	-258	2.5	173.9			Not located					Not located					Not located		
CH^{9-1}	-3.1	-2.2	-868	6.9	145.1	-10.2	-0.1	-452	5.8	149.1	-3.6	-0.8	-583	7.3	145.4	7.1	-4.0	-1568	8.1	138.2
CH^{9-2}	2.9	2.9	-539	5.5	150.5	-3.9	6.0	-159	4.0	158.1	1.6	5.5	-295	6.0	151.5	11.2	0.1	-1316	7.0	141.2
CH^{10-1}	-12.8	-11.7	-964	9.1	138.8	-20.0	-11.0	-929	10.1	136.7	-9.6	-10.6	-1038	10.2	134.9	-2.9	-12.4	-1795	9.7	134.7
CH^{10-2}	4.2	4.5	-550	6.2	147.5	-4.2	6.3	-272	6.2	147.4	3.8	5.6	-415	7.0	143.2	12.3	2.3	-1351	7.4	140.3
CH^{11-1}	3.4	2.6	-501	6.0	147.4	-4.7	4.8	-256	6.2	146.6	1.3	3.4	-383	8.0	143.2	10.7	0.4	-1282	7.1	140.4
CH^{11-2}	3.3	3.3	639	6.3	146.4	-4.9	4.9	-340	6.5	145.5	2.1	4.0	-460	8.2	142.8			Not located		
CH^{12-1}	-0.1	-0.1	-502	5.1	151.6	-6.7	2.5	-175	4.2	156.0	0.4	1.7	-277	6.3	149.3	7.9	-2.8	-1116	6.6	143.0
CH^{12-2}	-1.2	0.2	-571	5.7	149.6	-8.1	3.2	-231	5.1	151.8	0.0	2.1	-328	7.1	146.7	8.3	-2.7	-1225	6.8	142.3
CH^{13-1}	2.2	1.6	-469	5.6	149.4	-5.1	4.5	-202	5.2	150.7	0.8	3.7	-311	7.1	146.5	10.2	-0.8	-1234	6.9	141.4
CH^{13-2}	1.7	1.9	-629	5.9	148.3	-6.0	3.8	-282	5.5	149.3	0.6	3.0	-378	7.2	146.1	10.5	-0.3	-1301	7.1	141.0
PIP_{ax}	ΔE_{DFT}	$\Delta E_{CC//DFT}$	$\tilde{\upsilon}$	$\Delta r_{X\text{-}H}$	$r_{XH\cdot\cdot OH}$	ΔE_{DFT}	$\Delta E_{CC//DFT}$	$\tilde{\upsilon}$	$\Delta r_{X\text{-}H}$	$r_{XH\cdot\cdot OH}$	ΔE_{DFT}	$\Delta E_{CC//DFT}$	$\tilde{\upsilon}$	$\Delta r_{X\text{-}H}$	$r_{XH\cdot\cdot OH}$	ΔE_{MP2}	$\Delta E_{CC//MP2}$	$\tilde{\upsilon}$ [a]	$\Delta r_{X\text{-}H}$	$r_{XH\cdot\cdot OH}$
NH^{7}	-11.0	-8.3	-392	-0.8	191.7	-21.3	-7.3	-511	-0.8	189.1	-15.1	-7.2	-719	-0.9	191.9			Not located		
CH^{8}	-7.1	-5.7	-687	5.6	150.8	-12.3	-2.3	-164	4.0	160.9	-6.1	-2.1	-239	4.9	156.6	3.6	-8.5	-1345	7.4	140.7
CH^{9-1}	-2.9	-2.4	-685	6.8	144.9	-9.6	-0.1	-292	6.4	147.5	-3.6	-0.2	-403	7.5	144.3	6.4	-4.7	-1498	7.9	138.2
CH^{9-2}	2.7	3.3	-669	5.9	149.1	-4.6	5.6	-228	5.6	151.7			Not located					Not located		
CH^{10}	-2.4	-2.0	-590	5.4	151.2	-8.4	1.0	-199	4.9	154.9	-1.7	0.0	-321	6.4	149.1	6.3	-4.1	-1110	6.5	143.3
CH^{11-1}	1.5	1.2	-569	5.7	149.0	-6.0	3.6	-231	5.7	150.6	-0.5	3.1	-285	6.8	147.4	10.2	-2.2	-1255	6.9	141.3
CH^{11-2}	1.8	1.5	-598	5.8	149.0	-5.4	3.9	-165	5.3	155.2	0.1	3.2	.336	7.0	146,9			Not located		
CH^{12-1}	0.1	-0.2	-519	5.2	151.3	-6.7	2.2	-176	4.7	155.3	0.6	2.2	-289	6.4	149.1	6.9	-3.8	-1152	6.6	142.7
CH^{12-2}	-1.0	0.3	-579	5.8	149.2	-8.3	3.1	-258	5.7	151.0	0.4	1.6	-406	7.2	146.3	CP		-1273		
CH^{13-1}	0.9	0.5	-476	5.0	152.1	-5.7	3.5	-134	4.2	158.0	-1.0	3.3	-223	5.9	151.7	9.2	-2.9	-1194	6.7	142.2
CH^{13-2}	0.8	1.0	-582	5.3	151.0			Not located					Not located					Not located		

[a] MP2 frequencies from MP2/6-31+G(d,p) calculations. [b]CP, Convergence Problems in MP2/aug-cc-pVTZ calculations.

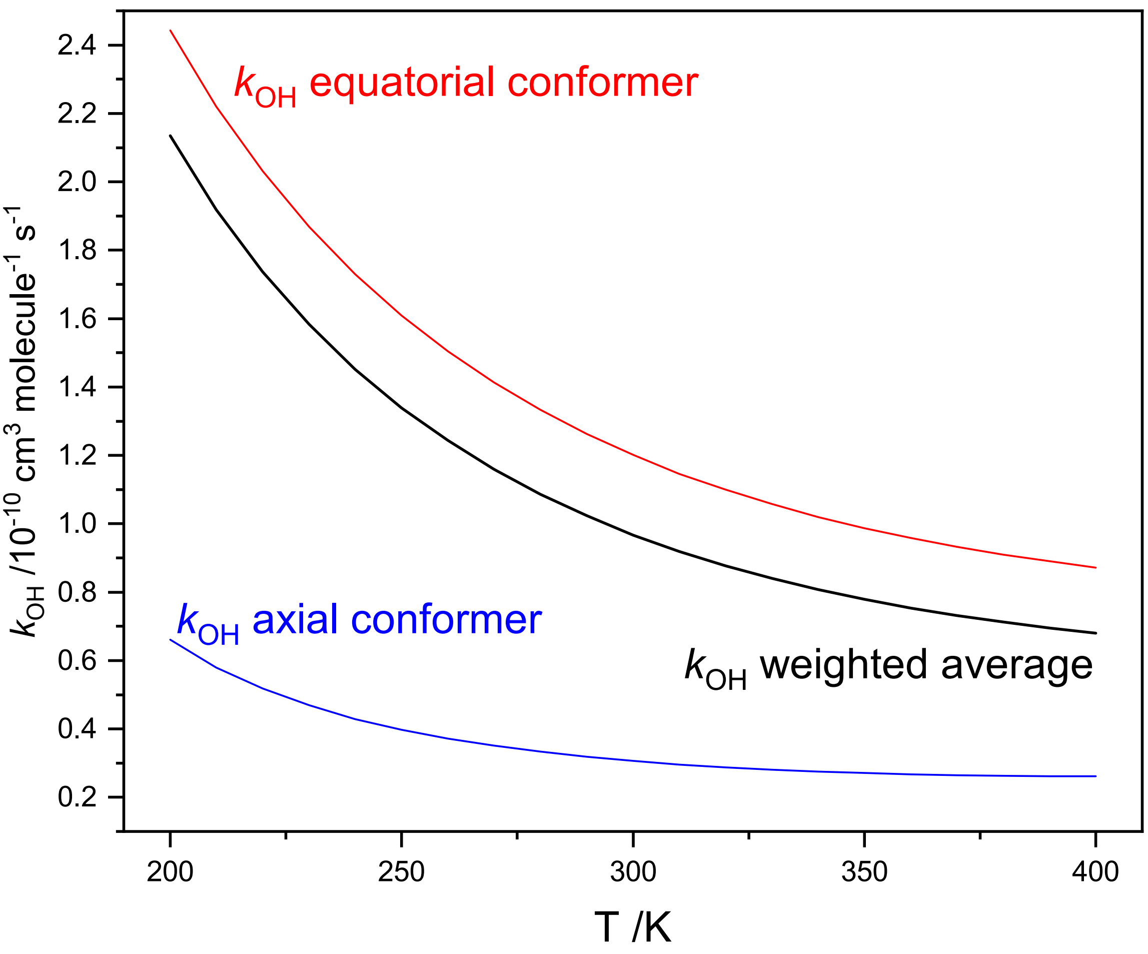

Figure S3. Calculated rate coefficients (200 – 400 K) for the piperidine + OH reaction
Results from MESMER models based on CCSD(T*)/aug-cc-pVTZ//M062X/aug-cc-pVTZ calculations.

Table S6. Site-specific rate coefficients for the OH reaction with piperidine

Results from MESMER models based on CCSD(T*)/aug-cc-pVTZ//M062X/aug-cc-pVTZ calculations (/10^{-12} cm^3 $molecule^{-1}$ s^{-1}). See Figures 1 and 2 in the main text for atom numbering and energetics.

T	k_{eq}^{H7-1}	k_{eq}^{H7-2}	k_{eq}^{H8-1}	k_{eq}^{H8-2}	k_{eq}^{H9-1}	k_{eq}^{H9-2}	k_{eq}^{H10-1}	k_{eq}^{H10-2}	k_{eq}^{H11-1}	k_{eq}^{H11-2}	k_{eq}^{H12-1}	k_{eq}^{H12-2}	k_{eq}^{H13-1}	k_{eq}^{H13-2}	k_{eq}^{Total}
200	59.60	9.56	19.25	34.30	2.57	0.14	25.58	0.03	0.11	0.11	0.60	0.28	0.10	0.41	244.28
210	55.20	8.39	17.83	31.88	2.15	0.15	22.02	0.03	0.13	0.11	0.60	0.28	0.11	0.43	222.01
220	51.28	7.45	16.61	29.77	1.83	0.16	19.05	0.04	0.14	0.12	0.61	0.28	0.11	0.45	203.08
230	47.77	6.67	15.55	27.92	1.60	0.17	16.57	0.04	0.16	0.13	0.61	0.28	0.11	0.48	186.89
240	44.66	6.02	14.64	26.30	1.41	0.19	14.48	0.05	0.18	0.14	0.62	0.29	0.11	0.50	173.00
250	41.82	5.48	13.83	24.86	1.27	0.20	12.71	0.05	0.20	0.15	0.62	0.29	0.12	0.53	160.89
260	39.29	5.03	13.13	23.59	1.16	0.22	11.21	0.06	0.22	0.16	0.63	0.30	0.12	0.56	150.44
270	37.00	4.64	12.51	22.44	1.07	0.23	9.93	0.06	0.24	0.17	0.64	0.30	0.12	0.59	141.25
280	34.93	4.32	11.97	21.45	0.99	0.25	8.83	0.07	0.26	0.18	0.66	0.31	0.13	0.62	133.28
290	33.06	4.04	11.49	20.55	0.93	0.27	7.89	0.07	0.28	0.20	0.67	0.31	0.13	0.66	126.26
300	31.35	3.79	11.07	19.74	0.88	0.29	7.08	0.08	0.30	0.21	0.68	0.32	0.13	0.69	120.06
310	29.82	3.58	10.70	19.03	0.84	0.30	6.37	0.09	0.33	0.22	0.70	0.33	0.14	0.73	114.65
320	28.42	3.40	10.38	18.41	0.81	0.32	5.75	0.09	0.35	0.24	0.72	0.34	0.14	0.76	109.91
330	27.15	3.24	10.09	17.84	0.78	0.34	5.22	0.10	0.38	0.25	0.73	0.35	0.14	0.80	105.67
340	26.00	3.11	9.83	17.33	0.76	0.37	4.75	0.11	0.41	0.27	0.75	0.36	0.15	0.84	101.94
350	24.97	2.99	9.60	16.88	0.74	0.39	4.34	0.12	0.43	0.28	0.77	0.36	0.15	0.89	98.67
360	24.00	2.88	9.40	16.47	0.72	0.41	3.98	0.12	0.46	0.30	0.79	0.37	0.15	0.93	95.75
370	23.13	2.79	9.23	16.11	0.71	0.43	3.66	0.13	0.49	0.32	0.81	0.39	0.16	0.97	93.19
380	22.34	2.71	9.07	15.79	0.69	0.46	3.38	0.14	0.53	0.33	0.83	0.40	0.16	1.02	90.94
390	21.61	2.63	8.94	15.50	0.69	0.48	3.13	0.15	0.56	0.35	0.85	0.41	0.17	1.07	88.97
400	20.95	2.57	8.82	15.25	0.68	0.51	2.90	0.16	0.59	0.37	0.88	0.42	0.17	1.12	87.23

T	k_{ax}^{H7}	k_{ax}^{H8}	$k_{ax}^{\mathrm{H9-1}}$	$k_{ax}^{\mathrm{H9-2}}$	k_{ax}^{H10}	$k_{ax}^{\mathrm{H11-1}}$	$k_{ax}^{\mathrm{H11-2}}$	$k_{ax}^{\mathrm{H12-1}}$	$k_{ax}^{\mathrm{H12-2}}$	$k_{ax}^{\mathrm{H13-1}}$	$k_{ax}^{\mathrm{H13-2}}$	k_{ax}^{Total}
200	19.35	8.83	1.82	0.09	0.14	0.04	0.04	0.44	0.42	0.53	0.36	66.05
210	17.37	7.06	1.53	0.10	0.13	0.04	0.04	0.44	0.40	0.55	0.38	57.97
220	15.73	5.79	1.33	0.10	0.13	0.04	0.04	0.45	0.40	0.57	0.39	51.77
230	14.34	4.86	1.18	0.11	0.12	0.04	0.04	0.46	0.39	0.60	0.41	46.87
240	13.14	4.16	1.06	0.12	0.12	0.04	0.04	0.47	0.39	0.62	0.42	42.94
250	12.13	3.63	0.97	0.12	0.12	0.04	0.04	0.48	0.39	0.65	0.44	39.78
260	11.27	3.21	0.90	0.13	0.12	0.05	0.05	0.49	0.39	0.67	0.46	37.19
270	10.52	2.87	0.85	0.14	0.12	0.05	0.05	0.50	0.40	0.70	0.48	35.05
280	9.86	2.60	0.80	0.15	0.11	0.05	0.05	0.51	0.40	0.73	0.50	33.28
290	9.30	2.38	0.76	0.16	0.11	0.05	0.05	0.52	0.41	0.76	0.52	31.82
300	8.81	2.20	0.73	0.17	0.11	0.05	0.06	0.54	0.42	0.79	0.54	30.59
310	8.38	2.05	0.71	0.18	0.11	0.06	0.06	0.55	0.43	0.82	0.56	29.59
320	8.00	1.92	0.69	0.19	0.11	0.06	0.06	0.57	0.44	0.85	0.59	28.76
330	7.67	1.81	0.67	0.20	0.12	0.06	0.06	0.58	0.45	0.89	0.61	28.07
340	7.37	1.72	0.66	0.21	0.12	0.07	0.07	0.60	0.46	0.92	0.63	27.51
350	7.12	1.64	0.65	0.23	0.12	0.07	0.07	0.62	0.47	0.96	0.66	27.07
360	6.89	1.57	0.64	0.24	0.12	0.07	0.07	0.64	0.48	1.00	0.69	26.73
370	6.68	1.51	0.63	0.25	0.12	0.07	0.08	0.65	0.50	1.03	0.72	26.46
380	6.50	1.46	0.63	0.26	0.12	0.08	0.08	0.67	0.51	1.07	0.74	26.28
390	6.35	1.42	0.62	0.28	0.12	0.08	0.08	0.69	0.52	1.11	0.77	26.17
400	6.21	1.38	0.62	0.29	0.12	0.08	0.09	0.71	0.54	1.16	0.80	26.11

Atmospheric fate of the 1-piperidinyl radical

Table S7. QCC results for the *eq-ax* equilibria in the piperidine C^3 and C^4 radicals

Energies (/Hartree) of the C^2- and C^3-piperidine radicals and connecting saddle points, and energy differences between these (/kJ mol^{-1}).

Species	M06-2X/aTZ		CCSD(T*)-F12a/aTZ	
	E_{Elec}	E_{ZPE}	E_{Elec}	$\Delta E_{v=0}$
Piperidine C^3-radical, *eq*	-251.20842	0.14522	-250.89186	0.0
SP C^3-radical, *eq-ax*	-251.20048	0.14382	-250.88280	20.1
Piperidine C^3-radical, *ax*	-251.20881	0.14525	-250.89232	-1.2
Piperidine C^4-radical, *eq*	-251.21082	0.14546	-250.89424	0.0
SP C^4-radical, *eq-ax*	-251.20319	0.14392	-250.88564	18.5
Piperidine C^4-radical, *ax*	-251.21006	0.14499	-250.89357	0.5

Table S7, continued.

T_1 and D_1 diagnostic values, vibrational frequencies (cm^{-1}), Rotational constants (GHz) and Cartesian coordinates of the species listed above. Results from CCSD(T*)-F12a/aug-cc-pVTZ//M06-2X/aug-cc-pVTZ calculations.

Piperidine C^3-radical, *eq*	C	0.080678	-1.360242	0.251426
	N	1.208510	-0.638732	-0.324674
T_1= 0.01158722 , D_1= 0.03427274	C	1.253492	0.751762	0.126371
	C	-0.034145	1.439805	-0.167637
$\tilde{\nu}$: 170.5, 223.5, 340.2, 383.4, 441.7, 480.6, 631.5, 775.8, 844.2, 872.7, 900.5, 929.3, 944.9, 1028.0, 1069.4, 1099.3, 1117.0, 1171.4, 1196.0, 1202.8, 1273.9, 1298.4, 1341.3, 1359.8, 1364.9, 1399.6, 1423.1, 1471.4, 1481.8, 1485.3, 1492.4, 1509.1, 2867.4, 2943.6, 2956.1, 3068.8, 3097.0, 3098.3, 3103.2, 3116.4, 3207.8, 3562.6	C	-1.299811	0.727183	0.161043
	C	-1.220548	-0.747406	-0.246535
	H	0.090318	-1.328586	1.354252
	H	0.148003	-2.405458	-0.049290
	H	2.076223	-1.101190	-0.089303
	H	2.081801	1.258210	-0.368710
	H	1.446977	0.807519	1.217362
B: 4.801706, 4.507795, 2.582316	H	-0.033856	2.511469	-0.303826
	H	-2.155638	1.216279	-0.304496
	H	-1.474150	0.771893	1.248055
	H	-2.072133	-1.297730	0.154941
	H	-1.245112	-0.827887	-1.334275
SP C^3-radical, *eq-ax*	C	-0.309594	-1.348438	0.256255
	N	0.999288	-0.943403	-0.173079
T_1= 0.01164729 , D_1= 0.03411667	C	1.430907	0.399334	0.113542
	C	0.359868	1.400381	-0.178515
$\tilde{\nu}$: -605.0, 165.0, 195.8, 335.1, 404.4, 452.6, 499.8, 679.5, 837.9, 847.7, 888.5, 913.7, 936.7, 1016.6, 1056.2, 1097.2, 1112.3, 1164.6, 1212.7, 1232.2, 1289.7, 1316.7, 1350.5, 1361.4, 1381.4, 1393.4, 1414.1, 1463.8, 1473.3, 1478.1, 1486.0, 1496.4, 2867.8, 2951.0, 2954.4, 3059.3, 3092.3, 3095.6, 3106.8, 3113.3, 3191.3, 3746.5	C	-1.048310	1.041711	0.156566
	C	-1.380687	-0.393013	-0.267178
	H	-0.370314	-1.368512	1.356364
	H	-0.496450	-2.363762	-0.090708
	H	1.577446	-1.556865	-0.707124
	H	2.328692	0.620489	-0.463893
	H	1.721093	0.497016	1.179497
B: 4.744345, 4.520039, 2.559960	H	0.638951	2.442355	-0.258031
	H	-1.744954	1.751473	-0.290400

H	-1.195417	1.115386	1.246505
H	-2.363136	-0.679058	0.112209
H	-1.404031	-0.454544	-1.356891

Piperidine C^3-radical, *ax*

T_1= 0.01179107, D_1= 0.03716585

$\tilde{\nu}$: 160.7, 221.9, 322.2, 386.7, 448.4, 488.1, 643.5, 787.3, 831.8, 869.3, 881.0, 904.1, 952.8, 1011.4, 1053.3, 1093.0, 1121.7, 1150.0, 1165.3, 1236.6, 1279.3, 1323.4, 1344.5, 1359.4, 1375.4, 1386.4, 1398.8, 1468.6, 1474.4, 1478.8, 1493.0, 1495.8, 2960.5, 2968.7, 3040.0, 3049.4, 3097.5, 3099.0, 3104.3, 3104.9, 3200.8, 3523.1

B: 4.761538, 4.483942, 2.587622

C	-1.380377	0.479659	0.169736
N	-1.120698	-0.914333	-0.206417
C	0.189154	-1.357233	0.261987
C	1.343917	-0.488258	-0.235180
C	1.117581	0.977459	0.155060
C	-0.267877	1.408667	-0.185180
H	-1.540851	0.483570	1.258760
H	-2.320821	0.795720	-0.280573
H	-1.148007	-0.986027	-1.217928
H	0.331789	-2.395335	-0.038675
H	0.166510	-1.340789	1.356493
H	2.291678	-0.848723	0.168493
H	1.400660	-0.566667	-1.324288
H	1.859240	1.625693	-0.311317
H	1.269820	1.065924	1.242450
H	-0.479513	2.445195	-0.407033

Piperidine C^3-radical, *eq*

T_1= 0.01174001, D_1= 0.03582035

$\tilde{\nu}$: 186.8, 235.2, 359.2, 400.3, 456.8, 475.4, 621.5, 783.8, 808.6, 848.0, 909.1, 921.4, 1007.4, 1048.9, 1063.5, 1084.9, 1106.3, 1181.1, 1189.4, 1204.6, 1257.2, 1322.5, 1333.9, 1347.1, 1372.1, 1400.3, 1420.3, 1460.2, 1469.2, 1482.9, 1501.1, 1511.5, 2962.9, 2964.1, 2985.5, 2986.6, 3099.6, 3100.0, 3103.9, 3104.7, 3204.6, 3568.2

B: 4.726136, 4.595880, 2.592119

C	0.032376	-0.756088	1.206027
N	-0.666044	-1.183023	0.000000
C	0.032376	-0.756088	-1.206027
C	0.032376	0.770965	-1.275732
C	0.534491	1.353904	-0.000000
C	0.032376	0.770965	1.275732
H	1.075361	-1.108655	1.219522
H	-0.476591	-1.173859	2.074134
H	-0.781580	-2.187055	0.000000
H	-0.476591	-1.173859	-2.074134
H	1.075361	-1.108655	-1.219522
H	0.626670	1.109947	-2.124600
H	-1.002757	1.094780	-1.454394
H	1.014558	2.321835	-0.000000
H	0.626670	1.109947	2.124600
H	-1.002757	1.094780	1.454394

SP C^4-radical, *eq-ax*

T_1= 0.01259362, D_1= 0.04337560

$\tilde{\nu}$: -609.7, 177.3, 231.1, 299.9, 386.6, 462.8, 489.0, 628.2, 812.7, 847.0, 897.2, 925.0, 996.4, 1018.9, 1032.1, 1090.2, 1116.6, 1167.1, 1213.0, 1238.6, 1261.2, 1328.9, 1332.8, 1354.0, 1363.7, 1406.3, 1409.7, 1454.0, 1471.8, 1473.1, 1489.4, 1497.8, 2973.2, 2974.4, 2986.0, 2986.3, 3099.0, 3099.8, 3112.2, 3112.4, 3214.3, 3745.2

B: 4.755073, 4.523750, 2.578515

C	-1.229059	-0.723914	0.221463
N	0.000000	-1.332188	-0.201283
C	1.229059	-0.723914	0.221463
C	1.276037	0.758907	-0.190011
C	-0.000000	1.430455	0.182931
C	-1.276037	0.758907	-0.190011
H	-1.328913	-0.777050	1.315115
H	-2.067243	-1.268169	-0.211457
H	0.000000	-2.127092	-0.804922
H	2.067243	-1.268169	-0.211457
H	1.328913	-0.777050	1.315115
H	2.133096	1.255354	0.265932
H	1.415378	0.793674	-1.279930
H	-0.000000	2.442155	0.559568
H	-2.133096	1.255354	0.265932
H	-1.415378	0.793674	-1.279930

Piperidine C^4-radical, *ax*	C	-0.031260	-0.764939	-1.209792
	N	0.581676	-1.296947	-0.000000
T_1= 0.01240021, D_1= 0.04243278	C	-0.031260	-0.764939	1.209792
	C	-0.031260	0.772878	1.270366
$\tilde{\nu}$: 122.9, 223.8, 289.6, 381.9, 463.6, 475.5, 611.8, 785.4, 800.3, 845.2, 899.0, 924.1, 973.3, 1011.3, 1026.0, 1106.5, 1111.6, 1141.8, 1171.6, 1247.2, 1269.2, 1323.1, 1327.0, 1341.5, 1360.7, 1398.2, 1403.4, 1462.2, 1474.2, 1486.0, 1494.9, 1497.5, 2960.4, 2963.2, 3054.6, 3056.8, 3098.6, 3099.3, 3108.2, 3108.4, 3215.2, 3529.9	C	-0.562458	1.336252	0.000000
	C	-0.031260	0.772878	-1.270366
	H	-1.064011	-1.121456	-1.237756
	H	0.482665	-1.182326	-2.075654
	H	1.574580	-1.091645	-0.000000
	H	0.482665	-1.182326	2.075654
	H	-1.064011	-1.121456	1.237756
B: 4.685309, 4.562141, 2.593708	H	-0.600217	1.122114	2.132531
	H	1.009440	1.098598	1.423278
	H	-1.177076	2.223625	0.000000
	H	-0.600217	1.122114	-2.132531
	H	1.009440	1.098598	-1.423278

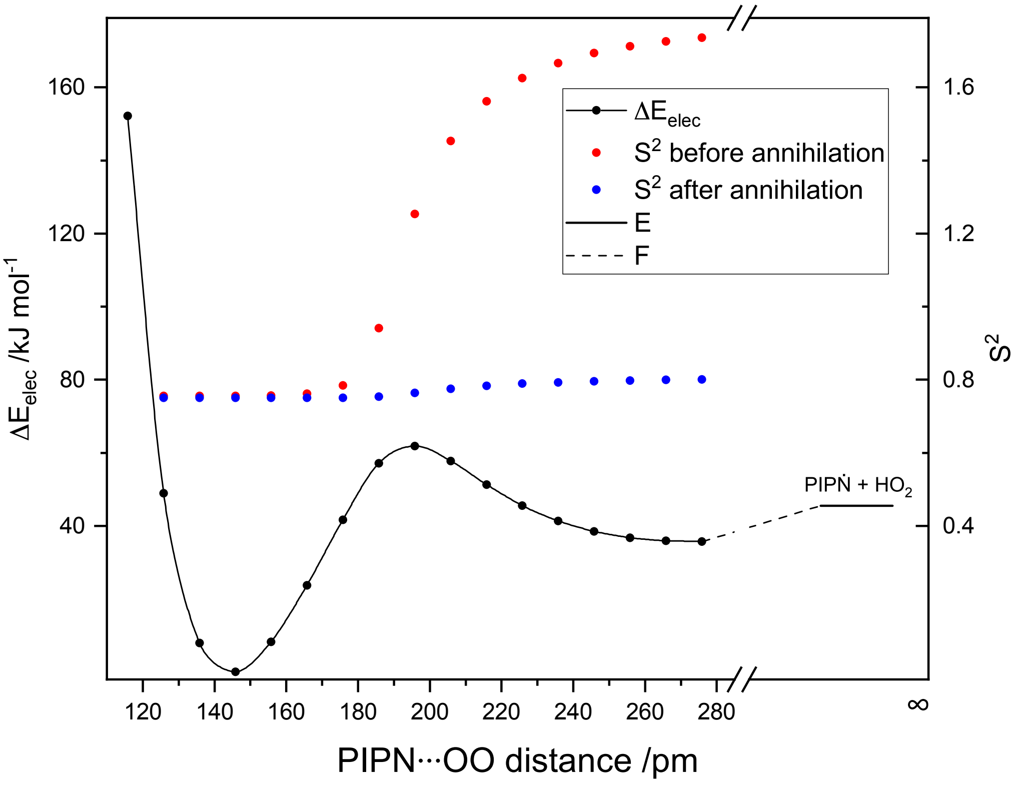

Figure S4. Spin contamination in the PIPNOȮ$_{eq}$ dissociation reaction
Results from M062X/6-31+G(d,p) calculations.

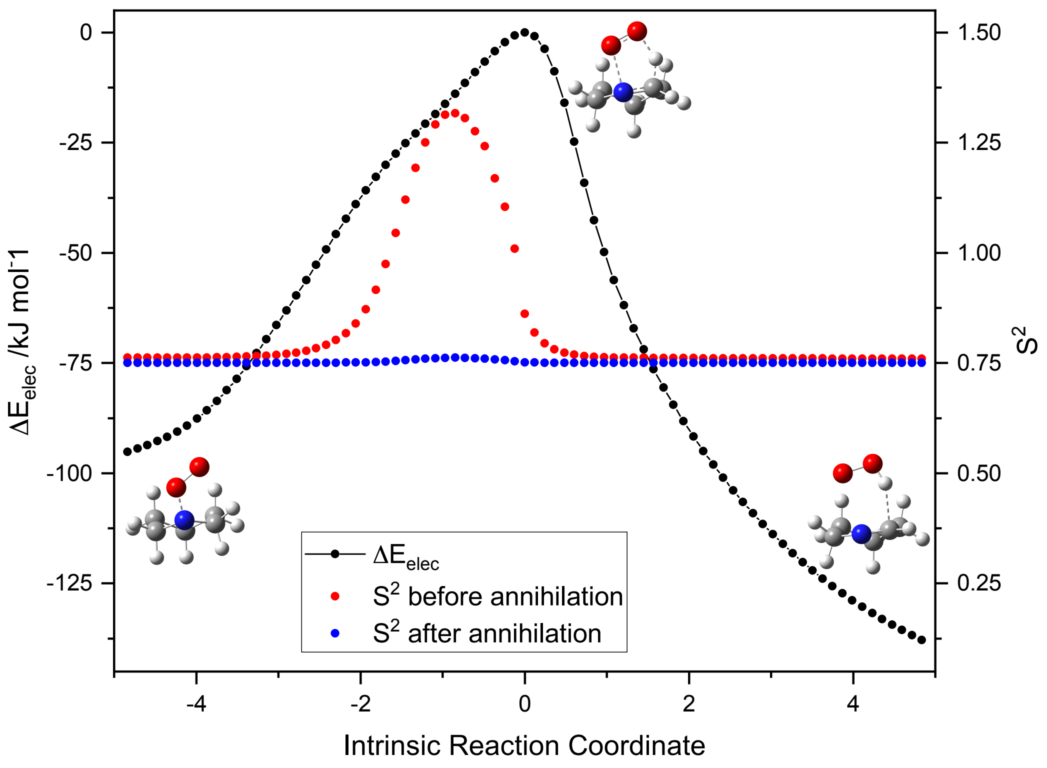

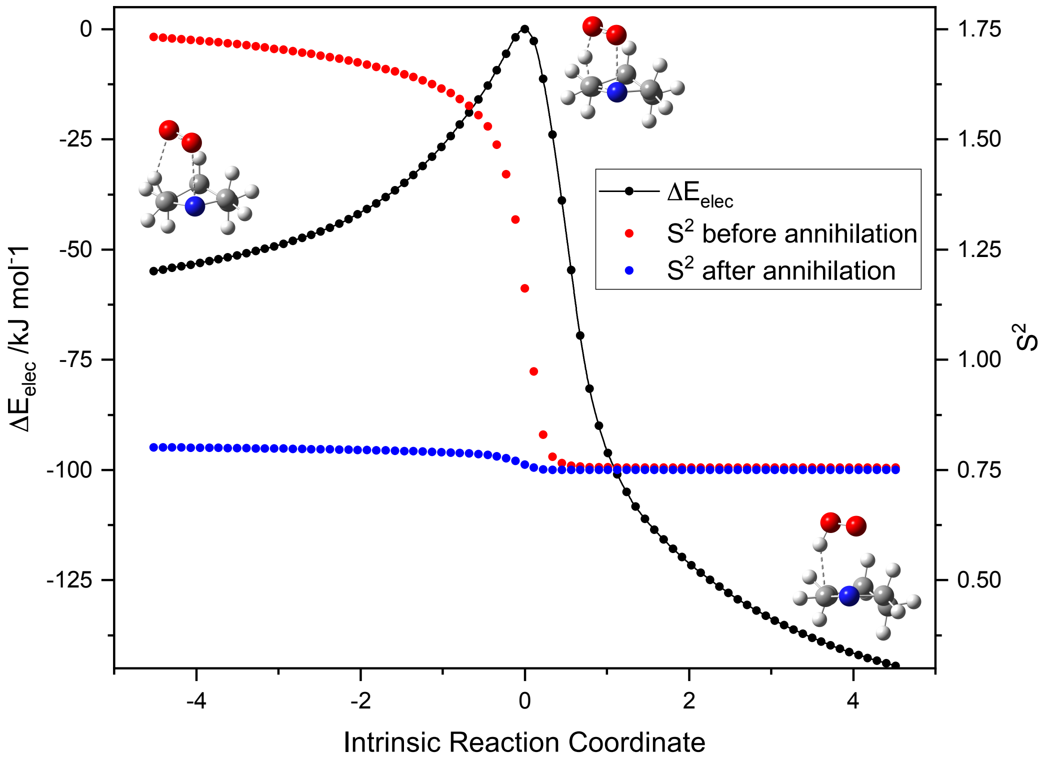

Figure S5. Spin contamination in the PIPNOȮ → PIP-IM + HOO reaction

Top: axial conformation. Bottom: equatorial conformation. Results from M062X/6-31+G(d,p) calculations.

Table S8. QCC results for the 1-piperidinyl radical reactions with O_2

Energies (/Hartree) and relative energy (/kJ mol^{-1}) of the 1-piperidinyl radical and intermediates, connecting saddle points and products in its reaction with O_2.

Species	M06-2X/aTZ [a]			CC//M062X [a]		UUCC//M062X		ωB97XD/aTZ			CC//ωB97XD	
	E_{Elec}	E_{ZPE}	$\Delta E_{v=0}$	E_{Elec}	E_{Elec}	E_{Elec}	E_{Elec}	E_{Elec}	E_{ZPE}	$\Delta E_{v=0}$	E_{Elec}	$\Delta E_{v=0}$
PIP$\dot{N}$	-251.21565	0.14592		-250.90064		-250.81148		-251.25897	0.14590		-250.90052	
O_2	-150.32480	0.00400		-150.19137		-150.14025		-150.33430	0.00388		-150.19163	
Sum reactants	-401.54045	0.14992	0.0	-401.09202	0.0	-400.95173	0.0	-401.59327	0.14978	0.0	-401.09215	0.0
PIPNO$\dot{O}_{eq}$	-401.55632	0.15529	-27.6	-401.10971	-32.4	-400.96823	-29.2	-401.60376	0.15513	-13.5	-401.10978	-32.2
SP^{2a}_{eq}	-401.51498	0.14681	58.7	-401.07727	30.5	-400.93489	36.1	-401.56960	0.14726	55.6	-401.07755	31.7
SP PIPNO$\dot{O}_{eq\text{-}ax}$	-401.53998	0.15420	12.4	-401.09024	15.9	-400.94730	22.8	-401.58989	0.15428	20.7	-401.09038	16.4
PIPNO$\dot{O}_{ax}$	-401.55488	0.15522	-24.0	-401.10747	-26.7	-400.96556	-22.4	-401.60272	0.15492	-11.3	-401.10745	-26.7
SP^{2a}_{ax}	-401.51604	0.14938	62.7	-401.08031	29.3	-400.93758	35.7	-401.57023	0.14872	57.7	-401.08064	27.4
PIP-IM•HO_2	-401.59051	0.15268	-124.2	-401.14474	-131.2	-401.00248	-126.0	-401.63886	0.15284	-111.7	-401.14484	-130.3
PIP-IM	-250.65918	0.13555		-250.34283		-250.25290		-250.69476	0.13555		-250.34277	
HO_2	-150.90809	0.01458		-150.77891		-150.72604		-150.92100	0.01454		-150.77924	
Sum products	-401.56727	0.15013	-69.9	-401.12174	-77.5	-400.97894	-70.9	-401.61576	0.15009	-58.2	-401.12201	-77.6
SP 2b	-401.50314	0.14912	95.8	-401.05832	86.4							
$CH_2CH_2CH_2\dot{C}HCH_2NOOH$	-401.52900	0.15203	35.6	-401.08141	33.4							

[a]Abbreviations: aTZ, aug-cc-pVTZ; CC/M062X, CCSD(T)-F12a/aug-cc/pVTZ//M06-2X/aug-cc-pVTZ; UUCC/M062X, UHF-UCCSD(T)-F12a/aug-cc/pVTZ//M06-2X/aug-cc-pVTZ.

Table S8, continued.

Species	BMK/aTZ			CC//BMK		G4		CBS-QB3		G3X-K	
	E_{Elec}	E_{ZPE}	$\Delta E_{v=0}$	E_{Elec}	$\Delta E_{v=0}$	$E_{v=0}$	$\Delta E_{v=0}$	$E_{v=0}$	$\Delta E_{v=0}$	$E_{v=0}$	$\Delta E_{v=0}$
PIPṄ	-251.15445	0.14529		-250.90043		-251.05371		-250.73641		-251.02943	
O_2	-150.30695	0.00399		-150.19176		-150.27866		-150.16461		-150.26635	
Sum reactants	-401.46140	0.14928	0.0	-401.09218	0.0	-401.33237	0.0	-400.90102	0.0	-401.29578	0.0
PIPNOȮ$_{eq}$	-401.47617	0.15471	-24.5	-401.10982	-32.0	-401.34627	-36.5	-400.91560	-38.3	-401.31051	-38.7
SP^{2a}_{eq}	-401.51498	0.14681	58.7	-401.07748	32.0	-401.30975	59.4	-400.88405	44.5	-401.27728	48.6
SP PIPNOȮ$_{eq-ax}$	-401.46119	0.15363	12.0	-401.09038	16.2	-401.32640	15.7	-400.89759	9.0	-401.29249	8.6
PIPNOȮ$_{ax}$	-401.47469	0.15501	-19.8	-401.10751	-25.2	-401.34337	-28.9	-400.91351	-32.8	-401.30873	-34.0
SP^{2a}_{ax}	-401.43725	0.14861	61.7	-401.08076	28.3	-401.31080	56.6	-400.88449	43.4	-401.28625	25.0
PIP-IM•HO_2	-401.51106	0.15232	-122.4	-401.14485	-130.3	-401.38218	-130.8	-400.95134	-132.1	-401.34758	-136.0
PIP-IM	-250.59667	0.13501		-250.34266		-250.50263		-250.18937		-250.48340	
HO_2	-150.89316	0.01466		-150.77932		-150.85684		-150.74110		-150.84319	
Sum products	-401.48983	0.14967	-73.6	-401.12198	-77.2	-401.35948	-71.2	-400.93048	-77.3	-401.32659	-80.9

Table S8, continued.

T_1 and D_1 diagnostic values, vibrational frequencies (cm^{-1}), Rotational constants (GHz) and Cartesian coordinates of the species listed above. Results from CCSD(T*)-F12a/aug-cc-pVTZ//M06-2X/aug-cc-pVTZ calculations.

$CH_2CH_2CH_2CH_2CH_2\dot{N}$ (PIP$\dot{N}$)

T_1= 0.01389069, D_1= 0.05612892

$\tilde{\nu}$: 217.5, 237.5, 364.2, 445.3, 454.7, 562.1, 811.8, 840.0, 857.9, 891.7, 894.9, 971.2, 980.6, 1037.3, 1075.9, 1106.1, 1137.2, 1140.5, 1243.1, 1251.0, 1284.1, 1294.9, 1332.0, 1336.1, 1351.9, 1383.7, 1387.6, 1482.5, 1488.7, 1494.0, 1499.4, 1517.9, 2978.7, 2987.6, 3047.0, 3063.0, 3063.4, 3100.3, 3106.9, 3108.8, 3110.7, 3113.8

B: 4.652185, 4.648684, 2.646002

C	-0.031830	-0.829670	1.186394
N	-0.715167	-1.280712	0.000000
C	-0.031830	-0.829670	-1.186394
C	-0.031830	0.706571	-1.250223
C	0.620061	1.286733	-0.000000
C	-0.031830	0.706571	1.250223
H	1.011884	-1.176624	1.173678
H	-0.520082	-1.249767	2.064543
H	-0.520082	-1.249767	-2.064543
H	1.011884	-1.176624	-1.173678
H	0.483802	1.030011	-2.154836
H	-1.065924	1.050895	-1.321138
H	0.545041	2.374817	-0.000000
H	1.685317	1.037933	-0.000000
H	0.483802	1.030011	2.154836
H	-1.065924	1.050895	1.321138

O_2

T_1= 0.00775897, D_1= 0.01442723

$\tilde{\nu}$: 1754.5

B: 44.635532

O	0.000000	0.000000	0.594925
O	0.000000	0.000000	-0.594925

$CH_2CH_2CH_2CH_2CH_2NO\dot{O}$ (eq)

T_1= 0.02408685, D_1= 0.13883012

$\tilde{\nu}$: 102.3, 141.7, 243.9, 305.2, 348.1, 363.0, 409.4, 468.9, 520.5, 712.5, 770.2, 822.2, 864.7, 881.7, 929.7, 946.5, 988.9, 1043.5, 1063.5, 1084.1, 1139.4, 1175.7, 1184.8, 1229.2, 1269.5, 1294.9, 1296.6, 1314.1, 1349.9, 1380.7, 1386.6, 1387.1, 1404.1, 1482.0, 1489.7, 1498.6, 1507.6, 1526.0, 3025.6, 3026.8, 3050.6, 3069.6, 3069.7, 3108.9, 3117.4, 3120.0, 3123.1, 3124.8

B: 4.016018, 1.566351, 1.290906

C	-0.062469	1.213786	0.036187
N	-0.569855	-0.000008	-0.602385
C	-0.062456	-1.213791	0.036198
C	1.448615	-1.251146	-0.152123
C	2.093678	0.000009	0.436615
C	1.448602	1.251152	-0.152138
H	-0.312819	1.222281	1.103494
H	-0.542930	2.064996	-0.443787
H	-0.542910	-2.065010	-0.443769
H	-0.312811	-1.222278	1.103505
H	1.841720	-2.151648	0.319486
H	1.670325	-1.313316	-1.219345
H	3.167400	0.000014	0.249800
H	1.955611	0.000015	1.521619
H	1.841700	2.151663	0.319461
H	1.670311	1.313311	-1.219360
O	-2.011374	-0.000024	-0.499947
O	-2.443930	0.000020	0.712092

SP 2a (eq)

T_1= 0.03328787, D_1= 0.19215779

$\tilde{\nu}$: -2033.0, 56.7, 105.4, 124.3, 190.1, 240.0, 330.9, 446.5, 459.6, 522.6, 589.0, 654.1, 805.4, 841.2, 860.5, 894.1, 914.1, 948.7, 1026.9, 1061.1, 1085.1, 1126.7, 1157.4, 1204.0, 1258.1, 1274.8, 1300.2, 1330.6, 1358.8, 1368.3, 1370.3, 1389.7, 1409.8, 1466.8, 1486.1, 1493.9, 1504.9, 1516.2, 1594.0, 3035.1, 3045.8, 3052.7, 3058.6, 3067.1, 3088.1, 3106.4, 3110.0, 3112.0

B: 3.025297, 1.535139, 1.333563

C	0.148276	0.752348	0.953116
N	0.267048	-0.584438	1.077077
C	-0.521717	-1.440265	0.202723
C	-1.812878	-0.834277	-0.338327
C	-1.537527	0.554572	-0.897766
C	-1.016156	1.430507	0.231153
H	1.125605	0.963862	0.212937
H	0.499420	1.279879	1.843658
H	-0.716890	-2.357882	0.759576
H	0.120385	-1.730699	-0.635224
H	-2.228507	-1.498297	-1.096289
H	-2.550541	-0.759296	0.464749

H	-2.434292	0.990532	-1.336994
H	-0.786836	0.487403	-1.690899
H	-0.696962	2.408313	-0.129624
H	-1.814881	1.605574	0.957056
O	2.279019	-0.605231	-0.371559
O	2.227753	0.595777	-0.602677

SP $CH_2CH_2CH_2CH_2CH_2NO\dot{O}$ (eq-ax) (SP $PIPNO\dot{O}_{eq\text{-}ax}$)

T_1= 0.04070376, D_1= 0.26071344

$\tilde{\nu}$: -273.7, 73.9, 169.7, 199.0, 326.3, 354.6, 387.8, 447.5, 518.5, 621.4, 769.9, 817.0, 852.8, 857.0, 925.1, 965.7, 972.1, 1040.6, 1054.6, 1086.8, 1166.6, 1174.0, 1184.4, 1219.5, 1284.7, 1296.5, 1352.4, 1359.8, 1373.2, 1381.2, 1384.4, 1388.5, 1411.5, 1482.8, 1488.9, 1490.6, 1491.6, 1505.5, 3005.6, 3006.6, 3039.2, 3062.6, 3064.0, 3108.5, 3113.9, 3116.2, 3129.8, 3160.9

B: 3.944191, 1.560006, 1.220675

C	-0.210839	1.118702	0.452597
N	-0.597800	-0.251670	0.255293
C	0.312846	-1.359410	0.366690
C	1.612885	-1.057348	-0.375263
C	2.141570	0.319861	0.021595
C	1.102328	1.398160	-0.278987
H	-0.091518	1.314189	1.525655
H	-1.024181	1.741083	0.091078
H	-0.190041	-2.236275	-0.037542
H	0.529541	-1.554490	1.424616
H	2.343340	-1.835102	-0.151318
H	1.422374	-1.077221	-1.450535
H	3.071027	0.533490	-0.505651
H	2.373083	0.325006	1.091828
H	1.468324	2.382049	0.015289
H	0.908738	1.424361	-1.353517
O	-1.846884	-0.596414	-0.204213
O	-2.700470	0.374515	-0.240381

$CH_2CH_2CH_2CH_2CH_2NO\dot{O}$ (ax). ($PIPNO\dot{O}_{ax}$)

T_1= 0.02954239, D_1= 0.18022093

$\tilde{\nu}$: 61.7, 163.6, 191.5, 309.5, 365.6, 409.0, 450.5, 475.0, 593.4, 607.8, 761.0, 799.9, 833.1, 868.2, 902.0, 906.4, 955.4, 1028.6, 1044.1, 1080.1, 1122.9, 1169.9, 1191.5, 1253.5, 1282.7, 1307.3, 1327.8, 1343.9, 1355.8, 1380.4, 1385.5, 1387.9, 1405.4, 1475.6, 1482.7, 1485.9, 1492.4, 1506.3, 3040.1, 3067.0, 3067.5, 3077.8, 3084.8, 3105.9, 3112.4, 3116.0, 3131.4, 3165.2

B: 3.282950, 1.855524, 1.535293

C	0.233372	-0.955167	0.910609
N	0.660154	0.438014	0.756315
C	-0.457671	1.360714	0.542763
C	-1.415737	0.930541	-0.564865
C	-1.893125	-0.501692	-0.339194
C	-0.694977	-1.437679	-0.199487
H	-0.278727	-0.972645	1.874707
H	1.123766	-1.569564	0.998135
H	-0.032935	2.344827	0.350746
H	-0.975698	1.408135	1.502293
H	-2.256850	1.623752	-0.598547
H	-0.902863	0.997903	-1.526627
H	-2.537413	-0.822132	-1.157686
H	-2.493525	-0.544129	0.575220
H	-1.016413	-2.455348	0.023705
H	-0.140083	-1.474982	-1.139445
O	1.462856	0.591746	-0.436210
O	2.319455	-0.339523	-0.600748

SP 2a (ax)

T_1= 0.02954239, D_1= 0.18022093

$\tilde{\nu}$: -273.7, 73.9, 169.7, 199.0, 326.3, 354.6, 387.8, 447.5, 518.5, 621.4, 769.9, 817.0, 852.8, 857.0, 925.1, 965.7, 972.1, 1040.6, 1054.6, 1086.8, 1166.6, 1174.0, 1184.4, 1219.5, 1284.7, 1296.5, 1352.4, 1359.8, 1373.2, 1381.2, 1384.4, 1388.5, 1411.5, 1482.8, 1488.9, 1490.6, 1491.6, 1505.5, 3005.6, 3006.6, 3039.2, 3062.6, 3064.0, 3108.5, 3113.9, 3116.2, 3129.8, 3160.9

B: 3.944191, 1.560006, 1.22067

C	-0.210839	1.118702	0.452597
N	-0.597800	-0.251670	0.255293
C	0.312846	-1.359410	0.366690
C	1.612885	-1.057348	-0.375263
C	2.141570	0.319861	0.021595
C	1.102328	1.398160	-0.278987
H	-0.091518	1.314189	1.525655
H	-1.024181	1.741083	0.091078
H	-0.190041	-2.236275	-0.037542
H	0.529541	-1.554490	1.424616
H	2.343340	-1.835102	-0.151318
H	1.422374	-1.077221	-1.450535

H	3.071027	0.533490	-0.505651
H	2.373083	0.325006	1.091828
H	1.468324	2.382049	0.015289
H	0.908738	1.424361	-1.353517
O	-1.846884	-0.596414	-0.204213
O	-2.700470	0.374515	-0.240381

$CH_2CH_2CH_2CH_2CH{=}N{\bullet}HO_2$ (PIP-IM•HO_2)

T_1= 0.02281903, D_1= 0.13419392

$\tilde{\nu}$: 30.4, 58.3, 82.5, 145.0, 161.8, 270.6, 287.9, 417.5, 467.9, 530.6, 711.0, 810.1, 836.1, 854.5, 876.7, 910.3, 932.5, 992.3, 1038.5, 1069.3, 1089.4, 1126.7, 1197.9, 1219.1, 1279.9, 1291.4, 1308.3, 1358.1, 1377.9, 1387.3, 1395.5, 1422.4, 1465.6, 1489.4, 1493.0, 1508.1, 1667.2, 1774.1, 2860.5, 3050.1, 3054.8, 3060.0, 3066.8, 3099.3, 3107.2, 3117.3, 3121.9, 3144.0

B: 4.290950, 0.987861, 0.830083

C	-0.004489	0.875306	-0.046315
N	-0.318397	-0.347669	0.050885
C	0.717662	-1.378668	0.117418
C	2.087915	-0.919125	-0.355663
C	2.437971	0.407172	0.307236
C	1.386293	1.441790	-0.073242
H	-0.834378	1.576161	-0.113814
H	-1.950307	-0.653693	0.076624
H	0.363122	-2.224987	-0.470477
H	0.764628	-1.720161	1.154961
H	2.828967	-1.685361	-0.130165
H	2.072213	-0.790942	-1.441055
H	3.431056	0.747321	0.018038
H	2.445004	0.275978	1.391751
H	1.411757	2.311842	0.584701
H	1.563361	1.826831	-1.082543
O	-3.236770	0.649493	-0.071406
O	-2.965574	-0.623014	0.066303

$CH_2CH_2CH_2CH_2CH{=}N$ (PIP-IM)

T_1= 0.01114063, D_1= 0.03458031

$\tilde{\nu}$: 151.6, 285.6, 419.0, 458.7, 521.0, 699.6, 812.9, 838.8, 855.1, 901.0, 923.2, 979.9, 1029.2, 1066.0, 1085.9, 1122.3, 1189.9, 1214.2, 1277.2, 1303.8, 1354.9, 1370.3, 1379.2, 1390.0, 1415.9, 1469.6, 1486.7, 1492.0, 1507.3, 1777.2, 3045.8, 3048.7, 3053.5, 3060.9, 3090.7, 3098.3, 3102.0, 3108.6, 3113.2

B: 4.928451, 4.742629, 2.674797

C	0.646387	-1.255141	0.059234
N	-0.605495	-1.367029	-0.056462
C	-1.427923	-0.162245	-0.132889
C	-0.749554	1.118068	0.335625
C	0.626482	1.228491	-0.308583
C	1.455255	0.017239	0.099667
H	1.213377	-2.184222	0.133338
H	-2.331280	-0.357114	0.445397
H	-1.749416	-0.062610	-1.173826
H	-1.373995	1.978445	0.094160
H	-0.638521	1.092403	1.423129
H	1.129195	2.152648	-0.025707
H	0.512826	1.242196	-1.395364
H	2.329965	-0.109427	-0.540656
H	1.842431	0.138412	1.116440

HO_2

T_1= 0.03589638, D_1= 0.12282697

$\tilde{\nu}$: 12528, 1459., 3686.8

B: 628.5431720, 34.6992321, 32.8838526

H	-0.880747	-0.865418	0.000000
O	0.055047	0.708193	0.000000
O	0.055047	-0.600015	0.000000

SP 2b (ax)

T_1= 0.01998546, D_1= 0.10323705

$\tilde{\nu}$: -1882.5, 197.0, 226.8, 274.5, 400.8, 404.2, 459.0, 535.7, 563.0, 624.1, 753.5, 776.6, 806.5, 853.1, 881.1, 896.0, 912.0, 961.2, 993.9, 1043.4, 1047.1, 1081.8, 1115.2, 1152.4, 1186.8, 1235.3, 1249.0, 1286.9, 1318.9, 1342.1, 1346.9, 1366.0, 1388.0,

C	-0.273117	-0.957895	-1.100310
N	-0.861134	0.362765	-0.832445
C	0.162269	1.383279	-0.573545
C	1.279834	1.018125	0.416975
C	1.750167	-0.437212	0.266029
C	0.574683	-1.366385	0.072120
H	0.327940	-0.854270	-2.009399
H	-1.088611	-1.649369	-1.298529
H	-0.361227	2.282247	-0.254357

1392.1, 1466.6, 1474.7, 1486.4, 1501.6, 1560.5, 3010.3, 3045.8, 3068.7, 3083.7, 3111.1, 3132.9, 3138.3, 3149.7, 3153.0

B: 2.901705, 2.147159, 1.975900

H	0.593455	1.584668	-1.556677
H	2.119074	1.695163	0.252850
H	0.927026	1.174409	1.432354
H	2.352288	-0.728341	1.125493
H	2.396147	-0.525155	-0.616705
H	0.751216	-2.430348	0.202107
H	-0.288328	-1.010040	1.009718
O	-1.747381	0.181762	0.217845
O	-1.085627	-0.171486	1.413735

$CH_2CH_2CH_2\dot{C}HCH_2NOOH$

T_1= 0.01500235, D_1= 0.05737253

$\tilde{\nu}$: 116.9, 152.6, 179.4, 246.2, 270.3, 311.4, 361.5, 426.6, 458.2, 523.0, 590.8, 696.6, 749.8, 795.7, 858.2, 871.3, 901.7, 911.6, 1000.4, 1038.5, 1081.8, 1096.6, 1138.4, 1153.8, 1217.4, 1260.4, 1294.7, 1321.4, 1325.7, 1351.8, 1367.2, 1376.7, 1392.6, 1398.6, 1460.1, 1469.1, 1477.8, 1492.8, 2955.6, 2978.7, 3052.8, 3078.8, 3108.0, 3116.2, 3134.3, 3151.0, 3205.3, 3816.7

B: 2.945713, 1.926966, 1.769008

C	0.030274	1.256265	-0.788727
N	0.730291	-0.025910	-0.907385
C	-0.148396	-1.191167	-0.821415
C	-1.122920	-1.192643	0.358640
C	-1.892567	0.132753	0.430765
C	-0.970407	1.297353	0.316848
H	-0.470677	1.403768	-1.756825
H	0.788069	2.034045	-0.704481
H	0.493712	-2.069522	-0.813197
H	-0.714254	-1.198115	-1.757477
H	-1.820462	-2.022175	0.232927
H	-0.575744	-1.348501	1.284655
H	-2.484009	0.186048	1.343820
H	-2.610226	0.160998	-0.405019
H	-1.214860	2.244477	0.776263
H	1.565470	0.812409	1.543622
O	1.828874	-0.106576	-0.113013
O	1.490504	-0.123102	1.316856

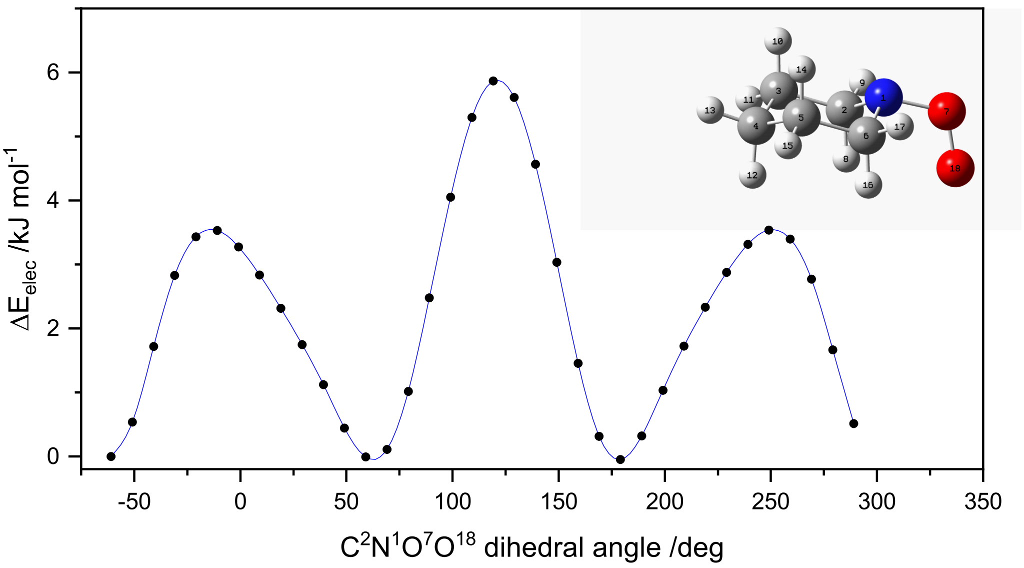

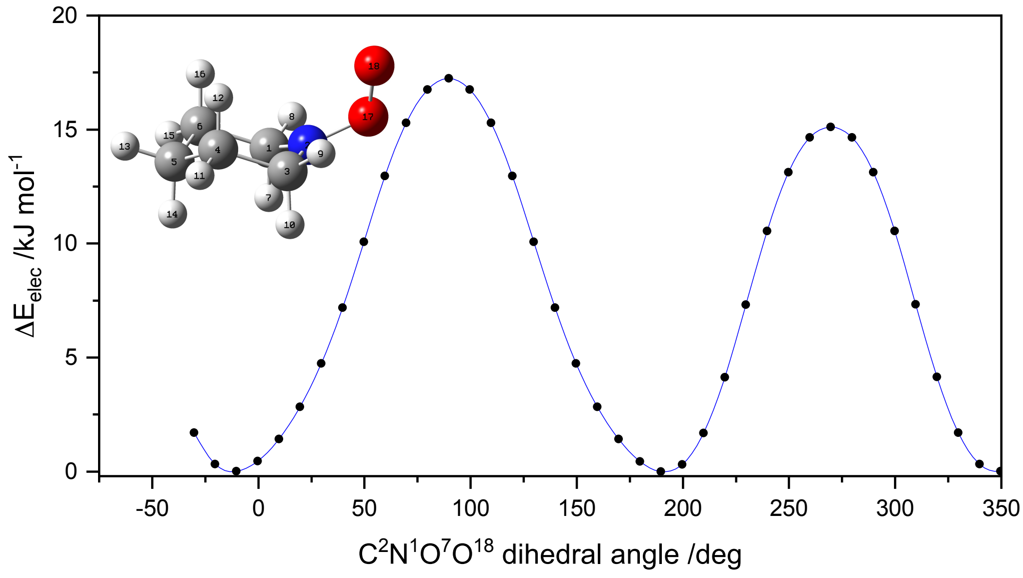

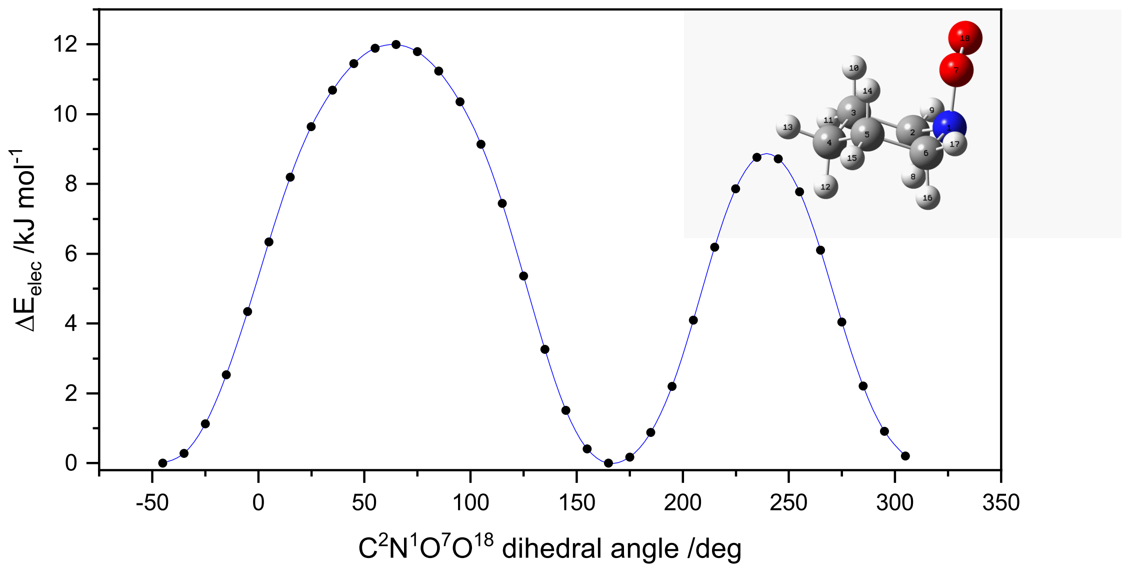

Figure S6. CNOO torsional potentials in PIPNOO.

$PIPNOO_{eq}$ (top), $PIPNOO_{eq\_SP\_aq}$ (middle) and $PIPNOO_{ax}$ (bottom)Results from M062X/6-31+G(d,p) calculations.

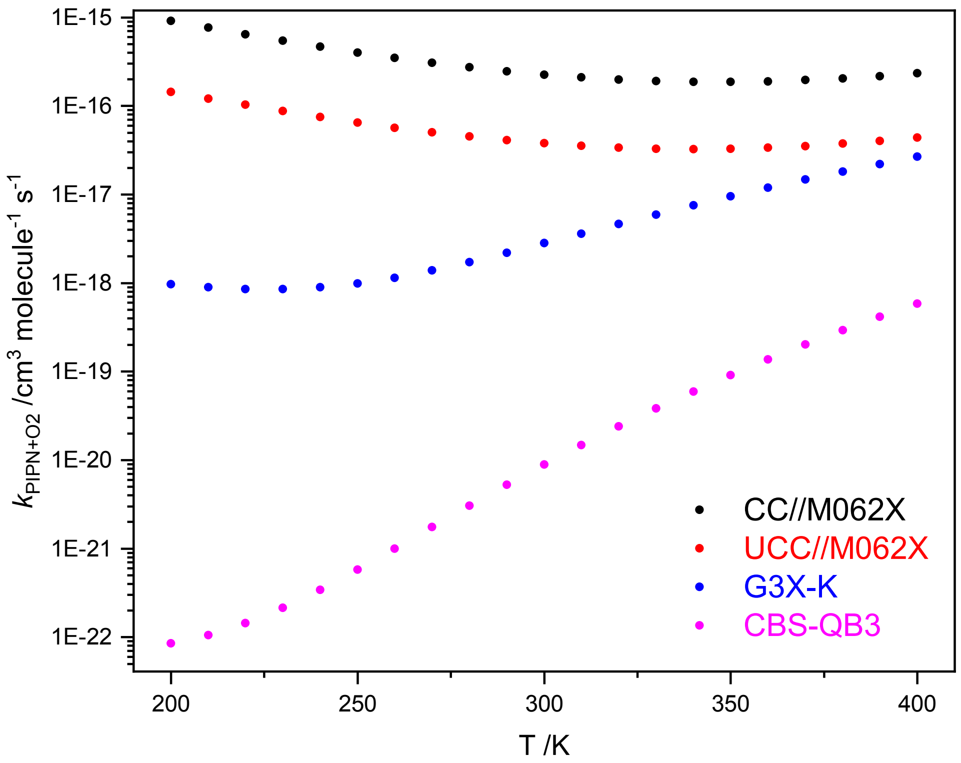

Figure S7. Calculated *k*(T) for the PIPṄ + O_2 ⟶ PIP-Im + HO_2 reaction
Results from MESMER model based on UCCSD(T*)-F12a/aug-cc-pVTZ//M06-2X/aug-cc-pVTZ calculations (CC//M06-2X), UHF-UCCSD(T*)-F12a/aug-cc-pVTZ//M06-2X/aug-cc-pVTZ calculations(UCC//M06-2X), and G3X-K and CBS-QB3 calculations.

Table S9. QCC results for the 1-piperidinyl radical reactions with NO and NO_2
Energies (/Hartree) of the 1-piperidinyl radical and intermediates, connecting saddle points and products in its reactions with O_2, and energy differences between these (/kJ mol^{-1}). Results from CCSD(T*)-F12a/aug-cc-pVTZ//M06-2X/aug-cc-pVTZ calculations

Species	M06-2X/aTZ		CCSD(T*)-F12a/aTZ	
	E_{Elec}	E_{ZPE}	E_{Elec}	$\Delta E_{v=0}$
$CH_2CH_2CH_2CH_2CH_2\dot{N}$	-251.21565	0.14592	-250.90064	
NO	-129.89335	0.00471	-129.76980	
Sum reactants	-381.10900	0.15063	-380.67044	0.0
$CH_2CH_2CH_2CH_2CH_2NNO$	-381.18957	0.15737	-380.75051	-192.5

Species	M06-2X/aTZ		CCSD(T*)-F12a/aTZ	
	E_{Elec}	E_{ZPE}	E_{Elec}	$\Delta E_{v=0}$
$CH_2CH_2CH_2CH_2CH_2\dot{N}$	-251.21565	0.14592	-250.90064	
NO_2	-205.07464	0.00917	-204.88884	
Sum reactants	-456.29029	0.15509	-455.78948	0.0
$CH_2CH_2CH_2CH_2CH_2NNO_2$	-456.38079	0.16352	-455.87605	-205.2

$CH_2CH_2CH_2CH_2CH_2NONO$	-456.34460	0.16064	-455.84313	-126.3
$CH_2CH_2CH_2CH_2CH_2N\dot{O}$	-326.43454	0.15201	-326.05083	
NO	-129.89335	0.00471	-129.76980	
Sum products	-456.32790	0.15672	-455.82063	-77.5
SP 4b	-456.28957	0.15530	-455.79020	-1.3
$CH_2CH_2CH_2CH_2CH{=}N{\bullet}HONO$	-456.38949	0.15843	-455.88810	-250.2
$CH_2CH_2CH_2CH_2CH{=}N$	-250.65918	0.13555	-250.34283	
HONO	-205.70919	0.02095	-205.52470	
Sum products	-456.36836	0.15650	-455.86753	-201.2

Table S9, continued.
T_1 and D_1 diagnostic values, vibrational frequencies (cm^{-1}), Rotational constants (GHz) and Cartesian coordinates of the species listed above. Results from CCSD(T*)-F12a/aug-cc-pVTZ//M06-2X/aug-cc-pVTZ calculations.

$CH_2CH_2CH_2CH_2CH_2\dot{N}$ (PIP$\dot{N}$)

T_1= 0.01389069, D_1= 0.05612892

$\tilde{\nu}$: 217.5, 237.5, 364.2, 445.3, 454.7, 562.1, 811.8, 840.0, 857.9, 891.7, 894.9, 971.2, 980.6, 1037.3, 1075.9, 1106.1, 1137.2, 1140.5, 1243.1, 1251.0, 1284.1, 1294.9, 1332.0, 1336.1, 1351.9, 1383.7, 1387.6, 1482.5, 1488.7, 1494.0, 1499.4, 1517.9, 2978.7, 2987.6, 3047.0, 3063.0, 3063.4, 3100.3, 3106.9, 3108.8, 3110.7, 3113.8

B: 4.652185, 4.648684, 2.646002

C	-0.031830	-0.829670	1.186394
N	-0.715167	-1.280712	0.000000
C	-0.031830	-0.829670	-1.186394
C	-0.031830	0.706571	-1.250223
C	0.620061	1.286733	-0.000000
C	-0.031830	0.706571	1.250223
H	1.011884	-1.176624	1.173678
H	-0.520082	-1.249767	2.064543
H	-0.520082	-1.249767	-2.064543
H	1.011884	-1.176624	-1.173678
H	0.483802	1.030011	-2.154836
H	-1.065924	1.050895	-1.321138
H	0.545041	2.374817	-0.000000
H	1.685317	1.037933	-0.000000
H	0.483802	1.030011	2.154836
H	-1.065924	1.050895	1.321138

NO
T_1= 0.02028868, D_1= 0.04698333
$\tilde{\nu}$: 2066.0
B: 52.3561936

N	0.000000	0.000000	-0.606410
O	0.000000	0.000000	0.530609

$CH_2CH_2CH_2CH_2CH_2NNO$

T_1= 0.01519332, D_1= 0.06812304

$\tilde{\nu}$: 41.5, 197.9, 258.5, 339.5, 358.7, 395.6, 418.0, 466.3, 553.4, 695.7, 812.1, 826.7, 863.5, 869.1, 936.2, 969.7, 1008.3, 1045.5, 1089.6, 1126.4, 1165.4, 1189.8, 1216.9, 1289.0, 1298.4, 1339.2, 1354.8, 1381.4, 1382.4, 1383.5, 1403.7, 1424.8, 1483.4, 1489.5, 1490.6, 1493.0, 1507.1, 1602.7, 3034.0, 3042.9, 3043.2, 3064.3, 3066.6, 3109.8, 3115.4, 3118.0, 3145.2, 3171.0

B: 3.968075, 1.625742, 1.276701

C	-0.226693	1.086082	0.527608
N	-0.667597	-0.269888	0.238166
C	0.286214	-1.349185	0.403587
C	1.553741	-1.060519	-0.393460
C	2.103101	0.320455	-0.041158
C	1.040984	1.395226	-0.264190
N	-1.872521	-0.584019	-0.190868
O	-2.653459	0.333141	-0.323493
H	-0.027913	1.158577	1.601570
H	-1.044667	1.755001	0.280027
H	-0.207572	-2.263240	0.082421
H	0.532149	-1.440831	1.466926

H	2.291981	-1.836282	-0.189740
H	1.316904	-1.101013	-1.459146
H	2.991885	0.537522	-0.633220
H	2.409691	0.328658	1.009591
H	1.414515	2.376238	0.029535
H	0.787456	1.445238	-1.325434

NO_2

T_1= 0.02428737, D_1= 0.06278199

$\tilde{\nu}$: 783.5, 1465.2, 1775.4

B: 253.9654019, 13.2904639, 12.6295376

N	0.000000	0.000000	0.314442
O	0.000000	1.090266	-0.137569
O	0.000000	-1.090266	-0.137569

$CH_2CH_2CH_2CH_2CH_2NNO_2$

T_1= 0.01438068, D_1= 0.06590226

$\tilde{\nu}$: 97.2, 99.9, 185.0, 291.6, 409.7, 411.2, 439.3, 472.2, 558.2, 622.1, 635.3, 813.7, 816.9, 841.6, 864.6, 880.2, 912.5, 964.3, 988.2, 1039.3, 1069.3, 1083.4, 1147.7, 1186.5, 1191.4, 1278.6, 1283.8, 1328.0, 1352.9, 1354.7, 1382.0, 1382.5, 1387.2, 1392.4, 1416.3, 1483.3, 1487.9, 1492.2, 1497.6, 1512.7, 1659.6, 3042.4, 3063.8, 3065.0, 3084.8, 3090.8, 3109.8, 3115.4, 3118.4, 3186.1, 3186.8

B: 2.732156, 1.526566, 1.208041

C	0.355652	1.228053	0.778784
N	-0.443456	0.000000	0.771425
C	0.355652	-1.228053	0.778784
C	1.301111	-1.261564	-0.418178
C	2.164392	0.000000	-0.456034
C	1.301111	1.261564	-0.418178
N	-1.509049	0.000000	-0.100971
O	-1.956286	-1.077500	-0.437215
O	-1.956286	1.077500	-0.437215
H	0.919779	1.200353	1.712062
H	-0.312321	2.080606	0.806364
H	-0.312321	-2.080606	0.806364
H	0.919779	-1.200353	1.712062
H	1.922726	-2.155487	-0.363369
H	0.705183	-1.337389	-1.331066
H	2.794143	0.000000	-1.345304
H	2.835722	0.000000	0.408526
H	1.922726	2.155487	-0.363369
H	0.705183	1.337389	-1.331066

$CH_2CH_2CH_2CH_2CH_2NONO$

T_1= 0.01665859, D_1= 0.07321232

$\tilde{\nu}$: 65.2, 109.6, 219.4, 246.6, 263.9, 353.9, 409.2, 444.2, 450.2, 493.8, 552.9, 731.9, 794.2, 820.5, 868.8, 881.1, 929.6, 955.9, 986.6, 1000.5, 1060.2, 1070.3, 1087.0, 1148.2, 1181.1, 1183.0, 1284.9, 1295.0, 1304.6, 1317.0, 1353.3, 1385.7, 1387.5, 1389.5, 1410.0, 1480.8, 1489.1, 1497.8, 1504.1, 1523.2, 1798.4, 2995.9, 3000.7, 3048.4, 3069.7, 3070.4, 3108.5, 3117.1, 3120.2, 3124.7, 3127.1

B: 3.519331, 1.093562, 0.949918

C	-0.166229	1.003001	-0.594988
N	0.336632	-0.221799	0.027500
C	-0.583289	-1.342537	-0.148526
C	-1.894927	-1.014338	0.550892
C	-2.489734	0.279677	0.002758
C	-1.464264	1.405900	0.090466
O	1.519779	-0.601188	-0.654052
N	2.697546	-0.138391	-0.054296
O	2.545404	0.577068	0.848601
H	-0.332719	0.838671	-1.668530
H	0.592573	1.774403	-0.477078
H	-0.115265	-2.225326	0.282223
H	-0.759561	-1.531027	-1.216860
H	-2.585020	-1.847645	0.418823
H	-1.705260	-0.908312	1.620823
H	-3.396639	0.544085	0.546311
H	-2.773529	0.131184	-1.043261
H	-1.843308	2.317000	-0.372263
H	-1.251320	1.631047	1.137380

$CH_2CH_2CH_2CH_2CH_2N\dot{O}$

T_1= 0.01688316, D_1= 0.07947001

C	0.243056	-0.609781	1.233377
N	-0.388872	-1.073519	0.000000
C	0.243056	-0.609781	-1.233377
C	0.243056	0.918625	-1.259343
C	0.908899	1.472977	-0.000000

$\tilde{\nu}$: 112.9, 210.1, 299.4, 423.4, 431.1, 464.6, 536.1, 594.4, 795.5, 808.6, 856.8, 869.8, 908.7, 965.3, 1009.8, 1039.6, 1079.5, 1109.4, 1152.0, 1178.4, 1258.2, 1268.3, 1284.1, 1315.6, 1353.1, 1366.8, 1370.2, 1380.5, 1382.8, 1475.8, 1480.5, 1483.4, 1489.7, 1503.6, 1514.2, 3040.7, 3060.3, 3063.5, 3067.4, 3070.4, 3106.6, 3113.9, 3116.6, 3156.3, 3157.6

B: 3.956562, 2.769220, 1.99757

C	0.243056	0.918625	1.259343
O	-1.651389	-1.223096	0.000000
H	1.266810	-0.991014	1.253831
H	-0.319347	-1.038824	2.057991
H	-0.319347	-1.038824	-2.057991
H	1.266810	-0.991014	-1.253831
H	0.751651	1.271563	-2.156872
H	-0.794011	1.257775	-1.310189
H	0.868630	2.562188	-0.000000
H	1.967645	1.194216	-0.000000
H	0.751651	1.271563	2.156872
H	-0.794011	1.257775	1.310189

SP 4b

T_1= 0.02115161, D_1= 0.11307041

$\tilde{\nu}$: -1473.0, 89.0, 101.8, 189.5, 228.7, 303.6, 369.2, 454.5, 466.1, 494.6, 523.4, 639.0, 688.3, 827.0, 835.6, 848.3, 885.6, 911.2, 928.3, 970.1, 1031.9, 1071.6, 1086.4, 1127.0, 1181.0, 1203.6, 1251.5, 1285.4, 1307.8, 1337.0, 1367.5, 1369.2, 1382.3, 1384.9, 1390.9, 1465.0, 1486.3, 1492.6, 1505.4, 1523.0, 1611.1, 1811.4, 3033.3, 3042.7, 3047.4, 3058.1, 3093.3, 3111.3, 3112.4, 3116.0, 3127.3

B: 2.740468, 1.286294, 1.079598

C	0.424907	1.297754	0.686464
N	-0.131048	0.023093	1.122458
C	0.422618	-1.132497	0.722907
C	1.556473	-1.211506	-0.300907
C	2.279510	0.121927	-0.464421
C	1.252438	1.242207	-0.584806
N	-1.717714	0.013153	-0.112607
O	-1.789269	-1.158674	-0.504922
O	-2.538887	0.841931	-0.339262
H	1.056107	1.609214	1.526782
H	-0.392654	2.013458	0.624254
H	-0.659819	-1.497425	0.076807
H	0.376073	-1.908256	1.487705
H	2.247301	-1.997757	0.001588
H	1.152983	-1.510800	-1.272681
H	2.933823	0.090828	-1.334661
H	2.910266	0.310808	0.408356
H	1.731556	2.207374	-0.749107
H	0.595268	1.055468	-1.439944

$CH_2CH_2CH_2CH_2CH$=N•HONO

T_1= 0.01516000, D_1= 0.06191814

$\tilde{\nu}$: 25.9, 53.1, 59.8, 120.5, 148.3, 183.5, 203.2, 286.7, 419.9, 466.0, 529.1, 709.0, 775.0, 811.6, 849.6, 859.0, 909.1, 930.5, 987.5, 1029.1, 1038.5, 1052.0, 1070.6, 1089.2, 1126.5, 1197.2, 1218.7, 1280.0, 1307.8, 1357.7, 1378.0, 1387.6, 1394.3, 1426.3, 1466.0, 1489.0, 1492.9, 1507.8, 1574.1, 1777.7, 1795.3, 2951.5, 3050.2, 3055.3, 3061.1, 3067.1, 3099.4, 3108.7, 3118.1, 3121.8, 3126.6

B: 4.120447, 0.639184, 0.566157

C	-1.272924	-1.366936	0.108601
N	-0.191217	-0.383375	0.054588
C	-0.449365	0.851941	-0.037831
C	-1.811312	1.483521	-0.070803
C	-2.910109	0.495970	0.301710
C	-2.619125	-0.842060	-0.366245
N	2.913995	0.374254	-0.037254
O	2.451323	-0.883716	0.090881
O	4.088565	0.424909	-0.052673
H	-1.338497	-1.713239	1.143460
H	-0.953244	-2.223888	-0.483664
H	1.448677	-0.771541	0.087787
H	0.410435	1.518752	-0.095807
H	-1.800977	2.351968	0.589630
H	-1.966003	1.878433	-1.079933
H	-3.885521	0.882550	0.010861
H	-2.926631	0.360749	1.385597
H	-3.395450	-1.574214	-0.146540
H	-2.594333	-0.709884	-1.450975

HONO
T_1= 0.02026557, D_1= 0.05681721
$\tilde{\nu}$: 578.3, 700.4, 904.9, 1342.4, 1841.1, 3830.7
B: 97.129144, 13.024286, 11.484325

N	0.000000	0.507869	0.000000
O	0.882545	-0.567211	0.000000
O	-1.101706	0.141499	0.000000
H	1.753285	-0.149388	0.000000

Table S10. QCC results for the NH_2 and CH_3NH radical reactions with O_3

Energies (/Hartree) of the NH_2 and CH_3NH radicals, O_3 and connecting saddle points in their reactions, and energy differences between these (/kJ mol^{-1}). Results from CCSD(T)/6-311G(3df,2p)//MP2(Full)/6-311+G(d,p).

Species	MP2(Full)/6-311+G(d,p)		CCSD(T)/6-311G(3df,2p)	
	E_{Elec}	E_{ZPE}	E_{Elec}	$\Delta E_{v=0}$
NH_2	-55.756105	0.019470	-55.787546	
O_3	-225.047905	0.009571	-225.123306	
Sum reactants	-280.804010	0.029041	-280.910853	0.0
H_2N-OOO TS1	-280.743847	0.029346	-280.904421	17.7
CH_3NH	-94.963897	0.050032	-95.021371	
O_3	-225.047905	0.009571	-225.123306	
Sum reactants	-320.011801	0.059603	-320.144677	0.0
CH_3NH-OOO TS1	-319.953177	0.058853	-320.139913	10.5

Table S10, continued.

Vibrational frequencies (cm^{-1}), Rotational constants (GHz) and Cartesian coordinates of the species listed above. Results from MP2(Full)/6-311+G(d,p) calculations.

NH_2	N	0.000000	0.141939	0.000000
$\tilde{\nu}$: 1520.1, 3461.7, 3564.5	H	0.800656	-0.496788	0.000000
B: 703.03196 391.12032 251.30879	H	-0.800656	-0.496788	0.000000
O_3	O	0.000000	0.445609	0.000000
$\tilde{\nu}$: 751.0, 1165.0, 2285.2	O	1.092586	-0.222803	0.000000
B: 106.08081 13.23408 11.76619	O	-1.092586	-0.222805	0.000000
H_2N-OOO TS1	N	1.968634	-0.296845	-0.007417
$\tilde{\nu}$: -201.2, 85.5, 126.9, 239.5, 515.8, 598.5, 691.3, 962.3, 1134.5,	H	1.556933	-1.142372	-0.413246
1499.9, 3456.0, 3571.5	H	1.809730	-0.399577	0.999764
B: 18.18656 4.57495 3.99201	O	-0.838468	0.294885	0.411545
	O	0.135087	0.824230	-0.302681
	O	-1.440007	-0.666632	-0.175689
CH_3NH	N	-0.804731	0.153025	0.000000
$\tilde{\nu}$: 147.5, 376., 511.4, 995.6, 1123.6, 1206.8, 1456.9, 1490.2,	H	-1.200201	-0.791090	0.000000
1492.6, 1513.8, 1627.1, 3087.3, 3180.3, 3212.7, 3507.3	C	0.630555	-0.012918	0.000000
B: 52.75937 10.54005 9.31678	H	1.125430	0.958304	0.000000
	H	0.962280	-0.580439	0.880512
	H	0.962280	-0.580440	-0.880512
CH_3NH-OOO TS1	N	-1.492253	0.616457	0.380524
$\tilde{\nu}$: 751.0, 1165.0, 2285.2	H	-1.258448	0.427153	1.358621
B: 106.08081 13.23408 11.76619	O	1.215900	-0.052798	-0.500437
	O	0.600538	1.054427	-0.143553
	O	1.668623	-0.729701	0.483261
	C	-1.762454	-0.656358	-0.250284
	H	-1.798392	-0.536791	-1.333754
	H	-1.050679	-1.448210	0.010749
	H	-2.752472	-0.994634	0.088250

Atmospheric fate of the 2-piperidinyl radical

Table S11. QCC results for the PIP-C²OȮ conformational pathways and internal H-transfer reactions
Energies (/Hartree) of 2-piperidinyl peroxy radical conformations, connecting stationary points, and energy differences of stationary points on the potential energy surface relative to that of the initial reactants.

Species	M06-2X/aTZ		CCSD(T*)-F12a/aTZ	
	E_{Elec}	E_{ZPE}	E_{Elec}	$\Delta E_{v=0}$
PIP-Ċ²	-251.22262	0.14655	-250.90654	
O_2	-150.32480	0.00400	-150.19114	
Sum reactants	-401.54741	0.15055	-401.09768	0.0
PIP-C²OȮ$_{ea}$	-401.60817	0.15668	-401.15968	-146.7
N_{inv} = $SP_{aa\text{-}ea}$	-401.59839	0.15526	-401.14952	-123.7
PIP-C²OȮ$_{aa}$	-401.59942	0.15601	-401.15079	-125.1
PIP-C²OȮ$_{ae}$	-401.59967	0.15651	-401.15119	-124.8
N'_{inv} = $SP_{ae\text{-}ee}$	-401.59072	0.15478	-401.14142	-103.7
PIP-C²OȮ$_{ee}$	-401.60187	0.15648	-401.15354	-131.1
$^3H_4/^3E$ ea-ae	-401.58953	0.15608	-401.14120	-99.7
$^3S_1$ ea-ae	-401.59054	0.15658	-401.14222	-101.1
$^NH_1$ ea-ae	-401.58258	0.15654	-401.13424	-80.3
E_N ea-ae	-401.59301	0.15643	-401.14507	-109.0
$B_{3,N}$ ea-ae	-401.59882	0.15642	-401.15076	-123.9
E_3 ea-ae	-401.59238	0.15588	-401.14315	-105.4
E_3 aa - ee	-401.59126	0.15566	-401.14212	-103.3
$B_{3,N}$ aa - ee	-401.59128	0.15631	-401.14345	-105.0
E_N aa - ee	-401.59335	0.15563	-401.14438	-109.3
$^NH_1$ aa - ee	-401.58833	0.15576	-401.13989	-97.1
$^3S_1$ aa - ee	-401.59531	0.15615	-401.14665	-113.9
$^3H_4/^3E$ aa - ee	-401.59335	0.15563	-401.14438	-109.3
SP^{9a}_{ea}	-401.57132	0.15061	-401.12598	-74.1
$POST^{9a}_{ea}$	-401.59051	0.15265	-401.14474	-118.0
PIP-Im	-250.65918	0.13555	-250.34283	
HOO	-150.90809	0.01458	-150.77891	
Sum products	-401.56727	0.15013	-401.12174	-64.3
SP^{9b}_{ea}	-401.55928	0.14983	-401.11289	-41.8

$POST_{ea}^{9b}$	-401.58213	0.15286	-401.13317	-87.1
1,2,3,4-tetrahydropyridine	-250.65862	0.13612	-250.34020	
HOO	-150.90809	0.01458	-150.77891	
Sum products	-401.56671	0.15070	-401.11911	-55.9
SP_{ea}^{9c}	-401.55555	0.15050	-401.10846	-28.4
$POST_{ea}^{9c}$	-401.57852	0.15382	-401.12889	-73.4
SP_{ea}^{9d}	-401.54659	0.14980	-401.09932	-6.3
$POST_{ea}^{9d}$	-401.58142	0.15386	-401.13275	-83.4
SP_{ea}^{9e}	-401.56844	0.15159	-401.12226	-61.8
$POST_{ea}^{9e}$	-401.59129	0.15520	-401.14241	-105.2
SP_{ee}^{9a}	-401.56969	0.15095	-401.12454	-69.5
$POST_{ee}^{9a}$ (same as SP_{ea}^{9a})	-401.57132	0.15061	-401.12598	-74.1
PIP-Im	-250.65918	0.13555	-250.34283	
HOO	-150.90809	0.01458	-150.77891	
Sum products	-401.56727	0.15013	-401.12174	-64.3
SP_{ee}^{9b}	-401.55971	0.14975	-401.11277	-41.7
$POST_{ee}^{9b}$	-401.57872	0.15423	-401.12957	-74.1
1,2,3,4-tetrahydropyridine	-250.65862	0.13612	-250.34020	
HOO	-150.90809	0.01458	-150.77891	
Sum products	-401.56671	0.15070	-401.11911	-55.9
SP_{ee}^{9d}	-401.54812	0.15021	-401.10101	-9.6
$POST_{ee}^{9d}$	-401.57396	0.15281	-401.12495	-65.7
SP_{ae}^{9a}	-401.56969	0.15095	-401.12454	-69.5
$POST_{ae}^{9a}$	-401.59051	0.15265	-401.14474	-118.0
PIP-Im	-250.65918	0.13555	-250.34283	
HOO	-150.90809	0.01458	-150.77891	
Sum products	-401.56727	0.15013	-401.12174	-64.3
SP_{ae}^{9b}	-401.53797	0.14996	-401.09233	12.5
$POST_{ae}^{9b}$	-401.57343	0.15377	-401.12401	-60.7
1,2,3,4-tetrahydropyridine	-250.65862	0.13612	-250.34020	
HOO	-150.90809	0.01458	-150.77891	
Sum products	-401.56671	0.15070	-401.11911	-55.9
SP_{ae}^{9c}	-401.53192	0.15062	-401.08719	27.7

$POST^{9c}_{ae}$	-401.57804	0.15425		-401.12947	-73.8
SP^{9d}_{ae}	-401.54812	0.15021		-401.10115	-10.0
$POST^{9d}_{ae}$	-401.57396	0.15281		-401.12509	-66.0

Table S11, continued.
T_1 and D_1 diagnostic values, vibrational frequencies (cm^{-1}), Rotational constants (GHz) and Cartesian coordinates of the species listed above. Results from CCSD(T*)-F12a/aug-cc-pVTZ//M06-2X/aug-cc-pVTZ calculations.

PIP-$\dot{C}^2$	C	0.045985	1.434030	0.153552
	N	-1.151463	0.809575	-0.174262
T_1= 0.01546620, D_1= 0.06541086	C	-1.297427	-0.595290	0.189626
	C	-0.086883	-1.385542	-0.284854
$\tilde{\nu}$: 225.7, 250.6, 385.8, 437.5, 470.4, 514.8, 664.8, 709.2, 834.7,	C	1.195420	-0.774716	0.273452
846.5, 906.5, 921.2, 932.4, 1050.9, 1075.6, 1083.3, 1107.6,	C	1.301676	0.689127	-0.156205
1176.0, 1190.0, 1249.7, 1281.5, 1306.3, 1351.8, 1367.8, 1383.7,	H	0.034614	2.512875	0.086339
1385.6, 1455.8, 1470.8, 1485.8, 1493.4, 1504.4, 1516.8, 2955.2,	H	-1.968971	1.362092	0.030685
2987.8, 3051.5, 3058.1, 3102.9, 3104.7, 3107.1, 3110.2, 3200.6,	H	-2.210747	-0.976190	-0.265778
3612.8,	H	-1.387035	-0.705684	1.279169
	H	-0.191718	-2.425725	0.024970
B: 4.759784, 4.575280, 2.582603	H	-0.055717	-1.365900	-1.377244
	H	2.066897	-1.335560	-0.065128
	H	1.172832	-0.828722	1.365278
	H	2.147892	1.176537	0.327464
	H	1.499568	0.713601	-1.239349
O_2	O	0.000000	0.000000	0.594925
T_1= 0.0078, D_1= 0.0144	O	0.000000	0.000000	-0.594925
$\tilde{\nu}$: 1754.5, B: 0.0000000 44.6355338 44.6355338				
PIP-$C^2O\dot{O}_{ea}$	C	-0.732811	1.429595	-0.153951
	N	0.171209	0.932862	0.885172
T_1= 0.02361647, D_1= 0.13958013	C	0.628563	-0.388538	0.708023
	C	-0.494079	-1.387221	0.516101
$\tilde{\nu}$: 81.7, 159.2, 188.3, 312.3, 329.1, 397.7, 443.6, 511.8, 589.3,	C	-1.473167	-0.919712	-0.558807
665.1, 740.1, 786.9, 846.1, 850.3, 909.1, 926.6, 949.7, 1058.7,	C	-1.935273	0.505650	-0.269545
1076.4, 1083.5, 1134.8, 1180.9, 1202.0, 1227.6, 1288.2, 1304.4,	O	1.474582	-0.541485	-0.522288
1312.7, 1346.2, 1356.3, 1380.5, 1390.0, 1393.9, 1447.7, 1475.8,	O	2.441778	0.316395	-0.530713
1488.3, 1501.0, 1519.9, 1522.6, 3013.7, 3060.1, 3066.6, 3080.3,	H	-0.230217	1.497925	-1.126838
3111.4, 3115.5, 3116.4, 3118.5, 3129.5, 3583.1	H	-1.044550	2.433328	0.129885
	H	0.949392	1.558591	1.040760
B: 3.376326, 1.776309, 1.468752	H	1.300793	-0.659251	1.520578
	H	-0.065786	-2.357500	0.266695
	H	-1.006158	-1.483766	1.474506
	H	-2.321996	-1.601227	-0.607498
	H	-0.980691	-0.949042	-1.533450
	H	-2.597586	0.862969	-1.058003
	H	-2.491942	0.530009	0.670241
N_{inv} = $SP_{aa\text{-}ea}$	C	-0.945142	1.384781	-0.245455
	N	0.162328	1.080293	0.648858
T_1= 0.02294672, D_1= 0.13507918	C	0.667427	-0.240577	0.661508
	C	-0.367029	-1.353503	0.554964
$\tilde{\nu}$: -184.1, 85.4, 144.4, 227.1, 317.4, 335.7, 395.3, 449.8, 527.1,	C	-1.434824	-1.043344	-0.490913
600.9, 676.4, 814.1, 842.7, 872.4, 913.3, 928.7, 946.3, 1040.3,	C	-2.045201	0.329606	-0.222736

1068.2, 1081.0, 1147.4, 1172.7, 1196.1, 1246.3, 1287.5, 1291.5, 1320.0, 1331.9, 1362.6, 1384.3, 1389.0, 1396.3, 1435.5, 1470.7, 1485.3, 1493.7, 1495.3, 1507.7, 3039.5, 3050.0, 3060.2, 3066.7, 3105.1, 3110.8, 3120.4, 3121.6, 3124.8, 3673.3

B: 3.546802, 1.665630, 1.373987

O	1.542916	-0.444616	-0.511083
O	2.612993	0.280889	-0.440330
H	-0.553180	1.462467	-1.264247
H	-1.329669	2.367315	0.021464
H	0.152576	1.535836	1.543050
H	1.313274	-0.368691	1.527977
H	0.138888	-2.294889	0.341676
H	-0.836693	-1.445631	1.536976
H	-2.199055	-1.820088	-0.479948
H	-0.984073	-1.050092	-1.486533
H	-2.801903	0.569656	-0.970575
H	-2.535125	0.330110	0.755252

PIP-$C^2O\dot{O}_{aa}$

T_1= 0.02373935, D_1= 0.14133398

$\tilde{\nu}$: 75.4, 136.7, 179.1, 302.6, 346.9, 390.1, 429.7, 485.1, 540.5, 569.8, 674.0, 816.4, 837.9, 886.6, 912.2, 932.1, 958.0, 1033.1, 1072.2, 1078.8, 1140.4, 1166.0, 1195.1, 1242.9, 1283.3, 1301.7, 1318.2, 1334.1, 1360.4, 1379.9, 1386.4, 1394.2, 1429.9, 1472.6, 1486.7, 1493.3, 1495.7, 1507.8, 3043.3, 3057.0, 3061.1, 3064.3, 3104.1, 3111.3, 3113.3, 3124.0, 3131.0, 3624.2

B: 3.487739, 1.704899, 1.401479

C	0.649930	-0.362482	0.655027
N	-0.353053	-1.358783	0.471948
C	-1.434154	-1.022932	-0.453322
C	-2.016871	0.368878	-0.236787
C	-0.894356	1.401843	-0.282591
C	0.160933	1.075387	0.769331
O	1.509779	-0.483468	-0.534828
O	2.559865	0.267638	-0.446556
H	1.281756	-0.631503	1.500203
H	-0.676673	-1.728507	1.351456
H	-2.196658	-1.794767	-0.364535
H	-1.033266	-1.086622	-1.467986
H	-2.767798	0.576801	-1.000000
H	-2.517067	0.411407	0.735441
H	-1.280355	2.407585	-0.119063
H	-0.435018	1.392894	-1.274516
H	1.017443	1.744663	0.708588
H	-0.271036	1.182005	1.767902

PIP-$C^2O\dot{O}_{ae}$

T_1= 0.02353255, D_1= 0.14060796

$\tilde{\nu}$: 119.9, 131.5, 241.9, 307.4, 350.0, 364.3, 443.1, 465.5, 544.8, 689.6, 804.9, 827.1, 866.9, 896.8, 932.2, 954.5, 957.7, 1034.0, 1073.9, 1093.1, 1146.7, 1165.3, 1186.3, 1242.5, 1252.9, 1287.5, 1323.2, 1340.6, 1364.9, 1370.3, 1385.1, 1393.7, 1406.3, 1475.4, 1489.4, 1494.1, 1496.9, 1506.7, 3043.2, 3047.4, 3054.3, 3066.5, 3083.1, 3104.9, 3110.2, 3119.7, 3121.4, 3522.6

B: 3.901807, 1.565505, 1.293482

C	-0.601378	0.037024	0.588026
N	-0.108801	-1.208980	0.061050
C	1.348092	-1.282353	0.179442
C	2.064159	-0.097490	-0.467501
C	1.520601	1.218296	0.085067
C	-0.001635	1.276320	-0.054967
O	-2.046255	0.063485	0.469976
O	-2.424985	-0.089225	-0.761305
H	-0.440187	0.048216	1.668382
H	-0.381752	-1.266504	-0.915505
H	1.674819	-2.225964	-0.254519
H	1.591857	-1.319197	1.245419
H	3.139194	-0.170907	-0.297328
H	1.903926	-0.132694	-1.548953
H	1.974878	2.069872	-0.420368
H	1.787817	1.300413	1.143170
H	-0.409180	2.173689	0.411494
H	-0.288880	1.281076	-1.108918

N'_{inv} = $SP_{ae\text{-}ee}$

T_1= 0.02295355, D_1= 0.13639960

$\tilde{\nu}$: -627.4, 82.4, 103.7, 210.2, 280.4, 339.0, 382.1, 458.8, 512.3, 524.5, 604.5, 833.8, 855.1, 885.3, 944.6, 954.6, 985.2, 1064.9,

C	-1.249177	-1.375847	0.164975
N	0.131958	-1.071484	-0.112402
C	0.646014	0.199831	0.280577
C	-0.244892	1.350533	-0.151198
C	-1.687769	1.080003	0.275069
C	-2.176238	-0.251485	-0.290662

1077.6, 1102.5, 1160.5, 1182.6, 1221.2, 1260.3, 1274.3, 1285.7, 1301.4, 1343.4, 1362.1, 1378.1, 1380.1, 1394.4, 1419.1, 1456.2, 1487.4, 1490.7, 1495.9, 1506.1, 2981.3, 3010.2, 3042.1, 3058.8, 3065.4, 3108.2, 3110.9, 3121.8, 3125.3, 3716.3

B: 4.262836, 1.446267, 1.159515

O	1.914454	0.384041	-0.381677
O	2.861074	-0.292234	0.199092
H	-1.395427	-1.530254	1.243682
H	-1.499188	-2.314221	-0.326031
H	0.692138	-1.648304	-0.708748
H	0.852568	0.246384	1.357794
H	0.127857	2.281490	0.276121
H	-0.187542	1.424067	-1.239650
H	-2.327822	1.898986	-0.051313
H	-1.745118	1.050161	1.367898
H	-3.196401	-0.455215	0.037392
H	-2.176630	-0.205369	-1.382219

PIP-C^{2}OȮ$_{ee}$

T_1= 0.02330508, D_1= 0.13894340

$\tilde{\nu}$: 90.9, 139.0, 233.0, 257.4, 343.6, 383.5, 455.4, 516.0, 522.6, 606.3, 818.5, 840.5, 867.2, 888.7, 945.9, 955.9, 982.8, 1070.8, 1089.6, 1103.0, 1166.2, 1167.2, 1177.6, 1248.0, 1285.9, 1292.6, 1297.9, 1341.8, 1357.3, 1372.3, 1383.7, 1386.8, 1435.4, 1477.9, 1489.7, 1494.5, 1503.8, 1515.1, 2968.4, 2996.7, 3048.7, 3068.9, 3076.0, 3112.7, 3117.1, 3119.1, 3129.5, 3546.8

B: 4.250057, 1.477524, 1.171404

C	1.166889	1.384188	0.168714
N	-0.175185	1.029284	-0.292361
C	-0.616093	-0.221030	0.262770
C	0.291749	-1.354636	-0.155126
C	1.726985	-1.047479	0.268663
C	2.158637	0.315411	-0.265759
O	-1.931159	-0.514082	-0.270657
O	-2.804647	0.350018	0.145273
H	1.209430	1.485664	1.264241
H	1.425095	2.350251	-0.261466
H	-0.843879	1.748509	-0.042249
H	-0.743716	-0.190691	1.354471
H	-0.064409	-2.285392	0.285349
H	0.230159	-1.442911	-1.241295
H	2.394318	-1.833475	-0.082856
H	1.789314	-1.042516	1.360973
H	3.155831	0.570064	0.093582
H	2.191599	0.289284	-1.356717

$^3H_4/^3E$ ea-ae

T_1= 0.02411885, D_1= 0.14365734

$\tilde{\nu}$: -50.4, 84.9, 189.7, 260.0, 334.9, 408.9, 441.4, 492.0, 607.9, 704.7, 724.3, 782.0, 832.7, 857.4, 918.3, 939.9, 968.0, 1022.8, 1078.8, 1095.9, 1141.1, 1168.4, 1203.8, 1238.1, 1253.5, 1290.5, 1314.5, 1351.3, 1354.3, 1367.6, 1389.4, 1401.3, 1405.8, 1483.5, 1488.6, 1504.0, 1508.8, 1516.8, 3061.7, 3065.6, 3071.0, 3079.6, 3083.0, 3103.6, 3122.1, 3124.7, 3130.9, 3543.9

B: 3.304930, 1.759904, 1.577091

N	-0.004064	-1.300150	0.184756
C	0.678096	-0.211596	0.837560
C	-0.118305	1.092708	0.892694
C	-0.884479	1.290380	-0.408866
C	-1.899025	0.149818	-0.606996
C	-1.453982	-1.115614	0.142607
H	1.027971	-0.495665	1.831886
H	-0.807788	1.037161	1.737971
H	0.572260	1.910634	1.096186
H	-0.170485	1.300123	-1.233183
H	-1.382542	2.258447	-0.414832
H	-2.884992	0.447820	-0.246048
H	-2.004514	-0.066863	-1.670355
H	-1.812228	-1.086820	1.173271
H	-1.890383	-2.006214	-0.305412
H	0.365828	-1.384998	-0.755358
O	1.954824	0.065251	0.164990
O	1.930363	-0.071095	-1.121165

$^3S_1$ ea-ae

T_1= 0.02356903, D_1= 0.14077280

$\tilde{\nu}$: 88.8, 134.7, 190.5, 263.9, 325.3, 366.6, 438.7, 490.8, 589.5, 681.4, 782.6, 805.2, 874.2, 892.3, 927.4, 966.2, 969.4, 1010.9,

C	1.403062	-1.291640	-0.066056
C	2.121605	0.045716	0.213037
C	1.354806	1.191553	-0.440629
C	-0.051617	1.301570	0.169512
C	-0.555289	-0.091276	0.562754
N	-0.045853	-1.147650	-0.266606

1077.3, 1100.0, 1150.3, 1165.1, 1198.1, 1227.2, 1255.2, 1288.9, 1324.0, 1341.2, 1351.2, 1374.1, 1380.5, 1390.5, 1415.8, 1482.0, 1493.1, 1498.5, 1505.8, 1514.4, 3052.7, 3065.3, 3069.6, 3077.6, 3098.4, 3109.0, 3118.8, 3127.2, 3134.9, 3545.4

B: 3.961981, 1.569617, 1.303291

H	1.572407	-1.987960	0.757125
H	1.805755	-1.754742	-0.965458
H	2.177035	0.223693	1.289895
H	3.147842	-0.000943	-0.149365
H	1.276022	1.001566	-1.513303
H	1.887434	2.134588	-0.330044
H	-0.042776	1.944991	1.049932
H	-0.749230	1.729810	-0.549571
H	-0.315648	-0.325737	1.600636
O	-2.005373	-0.119962	0.556665
H	-0.252088	-0.924268	-1.233326
O	-2.472275	0.002089	-0.646913

$^{N}H_1$ ea-ae

T_1= 0.02352733, D_1= 0.14040086

$\tilde{\nu}$: -228.1, 61.6, 131.4, 210.1, 332.5, 361.4, 441.8, 492.5, 644.9, 711.5, 737.3, 799.4, 839.7, 879.7, 945.4, 951.6, 975.9, 1018.4, 1117.1, 1125.8, 1165.5, 1179.7, 1209.8, 1252.0, 1292.7, 1298.7, 1315.2, 1340.3, 1348.9, 1372.4, 1388.0, 1396.1, 1416.3, 1484.0, 1493.4, 1505.0, 1512.3, 1529.0, 3064.8, 3075.9, 3081.0, 3092.3, 3097.5, 3104.0, 3118.9, 3131.0, 3141.6, 3529.8

B: 3.919680, 1.532745, 1.248543

N	-0.086879	-1.149641	-0.238325
C	1.329656	-1.340098	0.053959
C	2.206565	-0.066249	-0.047844
C	1.454293	1.284397	-0.193499
C	-0.070227	1.291676	0.084172
C	-0.580004	-0.058438	0.547452
H	-0.614084	1.518444	-0.833575
H	2.881841	-0.168261	-0.895916
H	1.933777	2.012180	0.457772
H	1.386869	-1.742789	1.066873
H	-0.341410	-0.253612	1.594491
H	-0.333131	2.058699	0.811553
H	2.844263	-0.022860	0.834193
H	1.606495	1.655803	-1.204759
H	1.706129	-2.115863	-0.609354
H	-0.236865	-0.946222	-1.221149
O	-2.033167	-0.054475	0.542347
O	-2.500261	-0.022496	-0.667008

E_N ea-ae

T_1= 0.02371945, D_1= 0.13918575

$\tilde{\nu}$: -193.1, 65.1, 125.2, 246.5, 319.3, 367.8, 461.4, 531.8, 611.7, 666.5, 717.7, 759.2, 803.5, 826.3, 913.8, 942.6, 978.1, 1014.3, 1101.8, 1130.3, 1136.1, 1223.5, 1237.0, 1246.3, 1293.1, 1305.3, 1315.6, 1345.1, 1360.1, 1368.5, 1392.8, 1400.9, 1441.0, 1488.3, 1493.1, 1509.6, 1518.6, 1524.6, 3014.5, 3077.3, 3085.8, 3096.9, 3109.2, 3119.7, 3125.3, 3129.6, 3142.7, 3582.5

B: 3.839447, 1.586261, 1.282665

N	-0.169128	-0.873732	0.767534
C	0.694880	-1.361987	-0.302813
C	2.030145	-0.654653	-0.161682
C	1.872459	0.878872	-0.138865
C	0.439542	1.422880	0.087686
C	-0.624536	0.438784	0.571755
H	0.818378	-2.436298	-0.183017
H	0.257076	-1.186561	-1.293326
H	2.474520	-0.991198	0.774428
H	2.702218	-0.951442	-0.966339
H	2.527176	1.284101	0.629816
H	2.231109	1.290030	-1.081081
H	0.476699	2.254360	0.787925
H	0.061966	1.838122	-0.846253
H	-1.164470	0.784693	1.450412
H	-0.943696	-1.487832	0.979189
O	-1.687106	0.491801	-0.507013
O	-2.654396	-0.319955	-0.238109

$B_{3,N}$ ea-ae

T_1 0.02365184, D_1= 0.13923315

$\tilde{\nu}$: -79.9, 89.3, 186.7, 242.2, 320.1, 388.1, 449.2, 541.5, 597.2, 652.9, 746.6, 769.7, 833.2, 853.7, 916.2, 932.0, 960.3, 1018.5, 1080.9, 1108.8, 1143.7, 1201.0, 1210.2, 1241.4, 1292.1, 1299.4,

C	-1.845738	0.616426	-0.553846
C	-0.515189	-1.428013	-0.024075
C	-1.873055	-0.750560	0.124882
C	0.598910	-0.538174	0.526977
C	-0.631006	1.453881	-0.093493
N	0.136009	0.730045	0.921462
H	-2.771812	1.152447	-0.351896

1307.4, 1343.2, 1349.8, 1364.4, 1386.3, 1392.1, 1441.4, 1487.7, 1493.6, 1504.7, 1514.0, 1533.3, 3034.8, 3072.9, 3075.2, 3082.8, 3107.9, 3118.0, 3124.1, 3129.9, 3135.5, 3587.6

B: 3.708956, 1.648726, 1.361816

H	-1.794082	0.470294	-1.633658
H	-0.496596	-2.394026	0.476640
H	-0.311728	-1.611602	-1.081220
H	-2.649385	-1.373383	-0.318173
H	-2.109517	-0.636635	1.182473
H	1.161375	-0.992078	1.339921
H	-0.005348	1.710571	-0.953623
H	-0.958670	2.387986	0.357866
H	0.887671	1.286549	1.303813
O	1.607186	-0.451588	-0.585662
O	2.604375	0.297613	-0.246219

E_3 ea-ae

T_1= 0.02330508, D_1= 0.13894340

$\tilde{\nu}$: -108.1, 98.2, 164.6, 292.7, 322.7, 358.6, 410.2, 458.4, 469.5, 637.6, 704.9, 767.1, 850.6, 851.5, 914.7, 929.7, 961.4, 1037.9, 1084.2, 1103.6, 1147.2, 1191.3, 1205.0, 1238.0, 1283.1, 1310.8, 1319.2, 1353.7, 1373.3, 1381.5, 1393.5, 1414.0, 1445.5, 1485.0, 1497.5, 1503.5, 1536.8, 1549.7, 3006.6, 3049.8, 3064.8, 3065.3, 3078.7, 3089.1, 3112.7, 3120.5, 3137.3, 3650.2

B: 3.332269, 1.689065, 1.457714

N	0.023292	-1.122295	-0.577153
C	-1.299156	-1.305621	0.003836
C	-1.765316	-0.070323	0.752347
C	-1.513782	1.163071	-0.102383
C	-0.008388	1.340437	-0.239130
C	0.686099	0.094267	-0.784217
H	-2.028673	-1.523099	-0.785276
H	-1.278252	-2.166397	0.674367
H	-2.820965	-0.186043	0.995740
H	-1.213953	0.025378	1.690297
H	-1.984223	1.038754	-1.081947
H	-1.945751	2.053935	0.352393
H	0.249162	2.187967	-0.871741
H	0.402809	1.534096	0.751184
H	0.986433	0.224616	-1.826070
H	0.307507	-1.856753	-1.201066
O	2.060213	0.045740	-0.165242
O	2.010552	-0.146663	1.110176

E_3 aa – ee

T_1= 0.02357369, D_1= 0.13917566

$\tilde{\nu}$: -196.9, 54.4, 103.5, 219.0, 317.4, 368.7, 401.3, 474.2, 563.9, 575.8, 701.1, 777.4, 839.8, 859.8, 918.6, 924.4, 963.6, 1024.4, 1077.6, 1104.8, 1139.4, 1202.2, 1210.0, 1254.4, 1296.8, 1304.0, 1309.5, 1353.4, 1363.3, 1373.9, 1392.2, 1396.3, 1454.6, 1480.0, 1500.3, 1509.3, 1536.3, 1551.5, 3022.4, 3064.1, 3073.0, 3074.7, 3078.8, 3081.7, 3119.4, 3127.0, 3130.4, 3659.8

B: 3.741489, 1.585704, 1.293724

C	1.329621	1.350703	0.033113
C	2.125791	0.078242	-0.325620
C	1.233829	-1.142994	-0.517776
C	0.287594	-1.247058	0.669790
C	-0.678710	-0.084770	0.643673
N	-0.097514	1.117699	0.219267
H	1.430886	2.082938	-0.768051
H	1.769554	1.803169	0.927093
H	2.724557	0.258022	-1.216950
H	2.825319	-0.140587	0.482345
H	0.650282	-1.057533	-1.435768
H	1.846429	-2.039529	-0.602571
H	-0.291082	-2.169426	0.657789
H	0.852061	-1.220070	1.604432
H	-1.260479	0.011815	1.563015
H	-0.640978	1.938999	0.417108
O	-1.723864	-0.510883	-0.342281
O	-2.652725	0.383829	-0.430517

$B_{3,N}$ aa – ee

T_1= 0.02337387, D_1= 0.13913067

$\tilde{\nu}$: -63.5, 110.8, 130.7, 262.6, 291.3, 392.6, 445.4, 531.6, 567.5, 647.5, 796.6, 811.1, 870.8, 908.4, 933.6, 950.7, 980.5, 1045.0, 1080.9, 1132.5, 1151.0, 1185.4, 1207.0, 1237.3, 1284.9, 1294.1,

C	-1.184473	-1.415437	0.088511
C	-2.184159	-0.243068	0.178297
C	-1.600340	1.015950	-0.453642
C	-0.296853	1.375260	0.256089
C	0.619722	0.156871	0.382116
N	0.121635	-0.949211	-0.383680
H	-1.532267	-2.158910	-0.626486

1304.5, 1340.2, 1354.8, 1361.8, 1390.2, 1391.2, 1428.6, 1479.9, 1492.7, 1501.2, 1506.4, 1530.4, 2999.0, 3008.8, 3066.7, 3079.0, 3087.2, 3103.7, 3120.2, 3124.5, 3136.1, 3555.8

B: 4.180027, 1.495681, 1.192412

H	-1.110294	-1.911896	1.064113
H	-3.124441	-0.516055	-0.297692
H	-2.411337	-0.032173	1.225259
H	-1.410430	0.846103	-1.512396
H	-2.303452	1.844032	-0.373137
H	0.244261	2.149010	-0.288757
H	-0.510854	1.768688	1.250059
H	0.843009	-0.096679	1.426411
H	0.800069	-1.700702	-0.353822
O	1.906113	0.537070	-0.183845
O	2.786502	-0.397620	-0.008157

E_N aa – ee

T_1= 0.02362825, D_1= 0.13906487

$\tilde{\nu}$: -140.2, 84.0, 108.6, 270.1, 312.8, 368.4, 388.4, 461.1, 509.8, 554.6, 677.1, 797.7, 839.4, 857.7, 913.9, 934.0, 962.2, 1034.3, 1085.7, 1102.6, 1150.7, 1192.1, 1208.8, 1260.0, 1299.2, 1307.8, 1315.4, 1354.5, 1365.8, 1383.0, 1391.7, 1404.9, 1454.0, 1489.1, 1497.8, 1505.4, 1538.4, 1549.8, 3010.3, 3052.6, 3060.9, 3074.4, 3077.5, 3078.4, 3114.5, 3119.2, 3132.0, 3664.2

B: 3.910545, 1.507022, 1.217615

C	-1.205271	-1.421059	0.020893
C	-1.966771	-0.268540	-0.610980
C	-1.758129	0.991918	0.215435
C	-0.294524	1.397023	0.111558
C	0.660634	0.265545	0.478876
N	0.141719	-1.030988	0.402457
H	-1.159756	-2.265037	-0.669982
H	-1.761407	-1.754413	0.904733
H	-1.606818	-0.094212	-1.627851
H	-3.020245	-0.538130	-0.676643
H	-2.388104	1.806285	-0.140485
H	-2.033157	0.800191	1.256176
H	-0.083239	1.676855	-0.922129
H	-0.067722	2.265546	0.726695
H	1.164844	0.434022	1.431282
H	0.679409	-1.724877	0.889880
O	1.795825	0.431833	-0.493878
O	2.787740	-0.329162	-0.166567

$^{N}H_1$ aa – ee

T_1= 0.02417836, D_1= 0.14341804

$\tilde{\nu}$: -160.6, 60.7, 116.1, 266.2, 280.9, 387.6, 397.2, 464.6, 496.7, 537.3, 711.1, 765.5, 830.2, 885.7, 915.4, 936.6, 961.3, 1012.7, 1087.3, 1123.0, 1150.6, 1205.5, 1214.1, 1279.4, 1298.3, 1304.5, 1322.5, 1344.6, 1358.0, 1373.5, 1392.7, 1395.7, 1431.9, 1488.3, 1492.3, 1507.4, 1517.6, 1521.9, 3040.1, 3057.5, 3070.2, 3099.4, 3105.5, 3109.8, 3116.8, 3126.5, 3148.1, 3660.3

B: 4.105490, 1.457982, 1.164058

C	0.139730	1.184444	0.060941
C	-2.204460	0.123435	0.205597
C	-1.383169	1.357673	-0.148783
C	-1.522560	-1.091763	-0.400756
C	0.649859	-0.219444	0.434229
H	0.492858	1.865742	0.830762
H	-2.268910	-0.011340	1.288166
H	-1.563743	1.578895	-1.201697
H	-1.421651	-0.952365	-1.481456
H	0.662052	1.475113	-0.848349
H	-3.220279	0.231732	-0.175663
H	-1.733193	2.222763	0.411898
H	-2.102029	-2.000422	-0.250245
H	1.039053	-0.232708	1.451202
N	-0.213947	-1.303253	0.195902
H	-0.197005	-2.003229	0.915681
O	1.879947	-0.471797	-0.377377
O	2.836813	0.324612	-0.024996

$^{3}S_1$ aa – ee

T_1= 0.02359810, D_1= 0.13937178

$\tilde{\nu}$: 87.0, 132.3, 178.3, 259.6, 319.6, 364.9, 416.5, 475.2, 519.3, 616.8, 733.4, 772.4, 825.0, 863.5, 918.2, 933.7, 979.1, 1002.9, 1071.5, 1092.8, 1166.9, 1173.4, 1191.4, 1242.9, 1257.2, 1289.4,

C	-0.914164	-1.311138	-0.264071
C	-2.085096	-0.316055	-0.309346
C	-1.601873	1.108269	0.021875
C	-0.104772	1.248205	-0.245611
C	0.641431	0.310126	0.691221
N	0.070800	-0.982674	0.755690
H	-0.398120	-1.326141	-1.223638

1309.0, 1337.1, 1365.4, 1367.4, 1391.2, 1391.9, 1427.4, 1489.2, 1495.2, 1504.2, 1510.1, 1519.7, 3058.3, 3068.3, 3084.0, 3090.4, 3101.0, 3105.0, 3122.9, 3136.0, 3140.1, 3645.2

B: 3.708654, 1.636413, 1.402151

H	-1.266612	-2.324605	-0.083800
H	-2.532034	-0.343902	-1.304251
H	-2.861452	-0.616460	0.394858
H	-1.787423	1.327267	1.076011
H	-2.159586	1.848536	-0.549044
H	0.130681	0.971536	-1.274241
H	0.243164	2.267896	-0.091014
H	0.745095	0.742441	1.686429
H	-0.167094	-1.282213	1.685440
O	2.060125	0.250522	0.265115
O	2.182953	-0.328281	-0.886489

$^{3}H_{4}/^{3}E$ aa – ee

T_1= 0.02362830, D_1= 0.13906537

$\tilde{\nu}$: -140.2, 84.0, 108.6, 270.1, 312.8, 368.4, 388.4, 461.1, 509.8, 554.6, 677.1, 797.7, 839.4, 857.7, 913.9, 934.0, 962.2, 1034.3, 1085.7, 1102.6, 1150.7, 1192.1, 1208.8, 1260.0, 1299.2, 1307.8, 1315.4, 1354.5, 1365.8, 1383.0, 1391.7, 1404.9, 1454.0, 1489.1, 1497.8, 1505.4, 1538.4, 1549.8, 3010.3, 3052.6, 3060.9, 3074.4, 3077.5, 3078.4, 3114.5, 3119.2, 3132.0, 3664.2

B: 3.910584, 1.507006, 1.217609

C	-0.294509	-1.397017	-0.111549
C	-1.966790	0.268514	0.610966
C	-1.758116	-0.991932	-0.215460
C	-1.205291	1.421053	-0.020874
C	0.660638	-0.265528	-0.478861
N	0.141700	1.030997	-0.402454
H	-0.067679	-2.265543	-0.726671
H	-0.083242	-1.676833	0.922146
H	-3.020268	0.538092	0.676612
H	-1.606856	0.094175	1.627842
H	-2.388091	-1.806310	0.140435
H	-2.033119	-0.800195	-1.256206
H	-1.761427	1.754431	-0.904706
H	-1.159780	2.265011	0.670025
H	1.164860	-0.433998	-1.431261
H	0.679376	1.724892	-0.889882
O	1.795817	-0.431784	0.493909
O	2.787774	0.329128	0.166530

SP_{ea}^{9a}

T_1= 0.02532170, D_1= 0.11758284

$\tilde{\nu}$: -801.6, 76.9, 136.3, 176.4, 182.3, 310.9, 395.4, 451.8, 472.9, 528.8, 658.7, 756.9, 820.6, 861.6, 879.1, 920.1, 932.8, 985.5, 1055.1, 1078.2, 1088.8, 1129.7, 1193.0, 1218.4, 1280.3, 1311.2, 1326.1, 1358.5, 1371.6, 1391.7, 1396.0, 1401.0, 1458.0, 1490.2, 1495.0, 1510.7, 1513.7, 1682.7, 1942.8, 3040.2, 3051.8, 3059.0, 3071.0, 3112.7, 3115.0, 3119.1, 3124.8, 3178.5

B: 3.316183, 1.587640, 1.324095

C	0.626389	-1.455766	-0.006323
N	-0.329040	-0.715365	0.827485
C	-0.281127	0.579839	0.883513
C	0.818408	1.432325	0.347523
C	1.641392	0.702854	-0.705595
C	1.932741	-0.709624	-0.214171
O	-1.973313	0.687026	-0.599797
O	-2.413568	-0.489033	-0.343902
H	0.146462	-1.643129	-0.971233
H	0.794623	-2.421362	0.468006
H	-1.429614	-0.920555	0.505662
H	-1.021171	1.056844	1.515924
H	0.369790	2.347340	-0.039033
H	1.448324	1.728725	1.193515
H	2.559828	1.249250	-0.913751
H	1.069531	0.652621	-1.634561
H	2.548711	-1.258758	-0.925285
H	2.485025	-0.665138	0.728264

$POST_{ea}^{9a}$

T_1= 0.02281914, D_1= 0.13419459

$\tilde{\nu}$: 29.4, 54.7, 82.6, 145.1, 162.0, 270.8, 288.0, 417.4, 467.9, 530.6, 711.0, 809.8, 832.6, 854.3, 874.1, 910.0, 932.5, 992.1, 1038.6, 1069.2, 1089.3, 1126.8, 1197.9, 1219.1, 1279.9, 1291.5,

C	-0.717665	-1.378668	0.117421
N	0.318396	-0.347672	0.050889
C	0.004491	0.875304	-0.046313
C	-1.386290	1.441791	-0.073244
C	-2.437971	0.407175	0.307234
C	-2.087917	-0.919123	-0.355664
O	3.236770	0.649494	-0.071403

1308.4, 1358.2, 1377.9, 1387.3, 1395.6, 1422.6, 1465.7, 1489.4, 1493.0, 1508.1, 1667.3, 1774.1, 2860.4, 3050.5, 3055.4, 3060.5, 3067.3, 3099.2, 3106.6, 3117.1, 3121.9, 3143.9

B: 4.290951, 0.987860, 0.830083

O	2.965575	-0.623014	0.066297
H	-0.764633	-1.720161	1.154963
H	-0.363126	-2.224988	-0.470474
H	1.950308	-0.653694	0.076621
H	0.834381	1.576157	-0.113810
H	-1.411754	2.311844	0.584698
H	-1.563355	1.826829	-1.082546
H	-3.431055	0.747325	0.018034
H	-2.445005	0.275982	1.391749
H	-2.828971	-1.685357	-0.130165
H	-2.072213	-0.790941	-1.441055

PIP-Im

T_1= 0.01114063, D_1= 0.03458031

$\tilde{\nu}$: 151.6, 285.6, 419.0, 458.7, 521.0, 699.6, 812.9, 838.8, 855.1, 901.0, 923.2, 979.9, 1029.2, 1066.0, 1085.9, 1122.3, 1189.9, 1214.2, 1277.2, 1303.8, 1354.9, 1370.3, 1379.2, 1390.0, 1415.9, 1469.6, 1486.7, 1492.0, 1507.3, 1777.2, 3045.8, 3048.7, 3053.5, 3060.9, 3090.7, 3098.3, 3102.0, 3108.6, 3113.2

B: 4.928451, 4.742629, 2.674797

C	0.646387	-1.255141	0.059234
N	-0.605495	-1.367029	-0.056462
C	-1.427923	-0.162245	-0.132889
C	-0.749554	1.118068	0.335625
C	0.626482	1.228491	-0.308583
C	1.455255	0.017239	0.099667
H	1.213377	-2.184222	0.133338
H	-2.331280	-0.357114	0.445397
H	-1.749416	-0.062610	-1.173826
H	-1.373995	1.978445	0.094160
H	-0.638521	1.092403	1.423129
H	1.129195	2.152648	-0.025707
H	0.512826	1.242196	-1.395364
H	2.329965	-0.109427	-0.540656
H	1.842431	0.138412	1.116440

HO_2
T_1= 0.0358, D_1= 0.1228
$\tilde{\nu}$: 1252.8, 1459.4, 3686.8
B: 628.5431720, 34.6992321, 32.8838526

H	-0.880747	-0.865418	0.000000
O	0.055047	0.708193	0.000000
O	0.055047	-0.600015	0.000000

SP_{ea}^{9b}

T_1= 0.02538451, D_1= 0.12340979

$\tilde{\nu}$: -964.9, 64.4, 98.4, 171.0, 207.5, 305.6, 354.0, 455.4, 477.2, 541.9, 574.0, 722.5, 755.7, 848.9, 857.9, 908.0, 925.0, 968.7, 983.8, 1046.6, 1088.2, 1113.4, 1161.8, 1219.9, 1226.4, 1293.7, 1313.4, 1337.8, 1342.5, 1355.7, 1378.2, 1397.8, 1411.9, 1487.3, 1499.8, 1514.4, 1547.4, 1594.2, 1633.8, 3037.9, 3060.6, 3071.7, 3113.7, 3126.8, 3128.7, 3159.8, 3236.4, 3650.4

B: 3.140638, 1.581248, 1.334314

C	1.340023	-0.979764	-0.683745
N	0.467883	-1.361616	0.416448
C	-0.277859	-0.441461	1.031229
C	-0.146572	0.942699	0.791015
C	1.029742	1.422313	-0.065788
C	2.050806	0.313081	-0.310505
O	-2.182253	-0.584066	-0.294805
O	-2.226929	0.628202	-0.696404
H	0.749999	-0.837670	-1.595127
H	2.047624	-1.788477	-0.854089
H	0.166808	-2.319738	0.472052
H	-0.914248	-0.803354	1.824600
H	-1.185851	1.054709	0.064034
H	-0.396309	1.552869	1.652629
H	1.514609	2.269351	0.417272
H	0.661014	1.782436	-1.028447
H	2.741282	0.593343	-1.104644
H	2.636502	0.133538	0.593013

$POST_{ea}^{9b}$

T_1= 0.02230557, D_1= 0.12976730

C	-0.865308	1.217294	-0.604722
N	-0.310626	1.254157	0.740330
C	-0.008620	0.037942	1.311085
C	-0.452710	-1.140893	0.844575
C	-1.386310	-1.222559	-0.338368

$\tilde{\nu}$: 40.9, 63.7, 77.4, 119.5, 178.6, 244.1, 295.8, 457.3, 464.1, 522.7, 581.1, 593.5, 753.7, 776.8, 861.8, 891.5, 923.4, 982.2, 992.4, 1046.0, 1083.2, 1108.7, 1146.1, 1209.1, 1226.6, 1277.1, 1277.9, 1301.6, 1352.3, 1375.1, 1388.7, 1435.2, 1485.6, 1493.5, 1511.9, 1523.7, 1535.0, 1712.4, 3007.2, 3033.5, 3073.0, 3089.7, 3117.3, 3126.3, 3200.8, 3219.3, 3286.8, 3633.0

B: 2.850055, 1.422931, 1.298237

C	-1.959355	0.158495	-0.661614
O	2.577854	0.406569	-0.218856
O	2.128390	-0.701464	-0.745083
H	-0.085814	0.976525	-1.339201
H	-1.263623	2.201424	-0.845054
H	0.382082	1.967865	0.897255
H	0.611896	0.088943	2.196281
H	1.352596	-0.951591	-0.179640
H	-0.217361	-2.037313	1.401289
H	-2.197483	-1.918403	-0.118632
H	-0.872858	-1.628463	-1.215734
H	-2.426647	0.163135	-1.645348
H	-2.724534	0.416259	0.072257

1,2,3,4-tetrahydropyridine_eq

T_1= 0.01138359, D_1= 0.03555998

$\tilde{\nu}$: 185.3, 289.5, 455.0, 457.8, 517.1, 575.2, 733.4, 767.5, 862.1, 893.6, 926.1, 977.2, 981.9, 1045.1, 1081.4, 1105.0, 1142.2, 1205.6, 1219.5, 1273.3, 1300.6, 1352.0, 1374.9, 1388.1, 1430.6, 1484.7, 1490.7, 1506.2, 1515.5, 1739.5, 2995.1, 3039.9, 3069.1, 3082.8, 3109.2, 3121.3, 3198.6, 3223.0, 3635.7

B: 5.049839, 4.746692, 2.666731

C	0.642321	-1.188310	0.293367
N	1.409864	-0.042311	-0.167312
C	0.767064	1.187700	-0.074244
C	-0.552046	1.328330	0.065220
C	-1.476907	0.142533	0.127219
C	-0.751234	-1.127680	-0.320188
H	0.550439	-1.188386	1.387769
H	1.158681	-2.099814	-0.004575
H	2.372930	-0.032245	0.123993
H	1.415880	2.049416	-0.161712
H	-0.966370	2.325648	0.096979
H	-2.346249	0.313039	-0.510833
H	-1.867352	0.014431	1.141139
H	-1.316952	-2.016899	-0.043510
H	-0.645237	-1.124461	-1.406302

SP_{ea}^{9c}

T_1= 0.01971457, D_1= 0.10894102

$\tilde{\nu}$: -1880.5, 195.0, 224.2, 276.1, 396.1, 419.4, 440.2, 532.2, 558.0, 631.5, 711.7, 775.3, 824.7, 848.1, 872.6, 920.7, 926.6, 960.0, 1043.0, 1067.1, 1071.4, 1085.9, 1115.7, 1160.5, 1165.0, 1223.7, 1252.1, 1255.3, 1318.7, 1338.4, 1361.5, 1362.3, 1374.8, 1428.9, 1465.5, 1483.0, 1512.7, 1528.6, 1574.7, 3024.8, 3048.8, 3065.1, 3104.2, 3116.4, 3117.9, 3138.3, 3153.4, 3590.3

B: 2.889257, 2.131471, 1.950914

C	-1.252057	-1.063124	0.358654
N	-0.197967	-1.322519	-0.627735
C	0.830937	-0.356213	-0.817697
C	0.229512	1.026772	-1.084656
C	-0.625147	1.348665	0.103216
C	-1.767858	0.374437	0.253100
O	1.768440	-0.175248	0.264429
O	1.092949	0.181419	1.426603
H	-0.901070	-1.238566	1.376870
H	-2.069720	-1.755152	0.157691
H	0.189174	-2.248570	-0.517176
H	1.484036	-0.680657	-1.626735
H	1.032879	1.745924	-1.226795
H	-0.367444	0.970538	-1.999726
H	-0.836807	2.395847	0.296894
H	0.262339	0.972756	1.033483
H	-2.379525	0.600937	1.125238
H	-2.411520	0.461992	-0.629554

$POST_{ea}^{9c}$

T_1= 0.01306744, D_1= 0.04165017

$\tilde{\nu}$: 110.4, 127.7, 186.8, 242.0, 273.4, 312.3, 366.0, 426.7, 461.4, 502.9, 592.1, 726.2, 786.0, 795.7, 873.5, 922.8, 928.5, 954.5, 1033.1, 1053.7, 1079.2, 1085.3, 1108.3, 1193.4, 1224.8, 1241.3, 1260.0, 1334.6, 1347.0, 1373.7, 1381.7, 1393.9, 1404.6, 1426.9,

C	-1.112579	-1.193689	0.333183
N	-0.189831	-1.161774	-0.802710
C	0.730920	-0.076515	-0.896742
C	0.014027	1.270441	-0.781223
C	-1.025597	1.285166	0.283906
C	-1.921872	0.100959	0.371992
O	1.873101	-0.154633	-0.024843
O	1.458674	-0.033459	1.329648

1462.2, 1471.5, 1512.3, 1518.5, 2978.1, 2986.3, 3060.6, 3086.7, 3097.3, 3106.2, 3111.1, 3205.8, 3582.9, 3807.8

B: 2.964047, 1.909690, 1.739991

H	-0.595924	-1.327724	1.285949
H	-1.782711	-2.039979	0.183954
H	0.290028	-2.043109	-0.918943
H	1.240600	-0.140240	-1.860455
H	0.758346	2.055351	-0.641414
H	-0.453155	1.453695	-1.759207
H	-1.308140	2.222647	0.740394
H	1.558833	0.912408	1.496096
H	-2.532750	0.131455	1.273538
H	-2.609911	0.094468	-0.486073

SP_{ea}^{9d}

T_1= 0.0358, D_1= 0.1228

$\tilde{\nu}$: -2059.4, 163.0, 219.3, 269.3, 316.2, 374.7, 454.3, 513.7, 561.8, 662.4, 700.8, 760.9, 807.9, 821.7, 845.3, 903.7, 935.1, 966.8, 1030.1, 1056.4, 1079.2, 1087.5, 1099.3, 1168.7, 1183.7, 1216.8, 1248.3, 1290.8, 1314.2, 1342.8, 1349.2, 1372.2, 1381.4, 1417.7, 1473.0, 1488.9, 1501.1, 1503.5, 1533.1, 3002.0, 3045.2, 3083.0, 3094.7, 3109.1, 3117.6, 3142.2, 3147.0, 3599.9

B: 2.642119, 2.266174, 2.112398

C	0.871492	-1.318885	0.506829
N	-0.247038	-0.736365	1.267307
C	-0.657046	0.555506	0.837686
C	0.561954	1.408080	0.524653
C	1.265222	0.855127	-0.728341
C	1.049723	-0.636927	-0.830679
O	-1.499293	0.564750	-0.377332
O	-1.388813	-0.617848	-1.086301
H	0.690722	-2.384556	0.378645
H	1.796721	-1.218494	1.088665
H	-1.034173	-1.366753	1.307773
H	-1.303604	1.013611	1.584653
H	0.258094	2.440907	0.370422
H	1.223845	1.379306	1.391288
H	2.335044	1.080249	-0.694001
H	0.873483	1.343576	-1.621616
H	-0.211485	-0.722598	-1.267213
H	1.657395	-1.163318	-1.561591

POST_{ea}^{9d}

T_1= 0.01261001, D_1= 0.03601786

$\tilde{\nu}$: 96.8, 149.3, 181.9, 206.4, 247.1, 307.6, 381.5, 399.6, 450.5, 580.5, 611.3, 700.2, 766.9, 848.9, 888.3, 913.3, 927.9, 949.3, 1027.6, 1050.0, 1077.9, 1092.0, 1109.4, 1196.7, 1218.4, 1224.9, 1269.7, 1326.8, 1360.4, 1368.4, 1376.9, 1386.9, 1406.6, 1439.5, 1472.4, 1482.1, 1494.2, 1515.4, 2931.0, 2992.4, 3070.1, 3087.4, 3097.3, 3099.7, 3135.1, 3209.3, 3577.4, 3832.8

B: 3.319930, 1.779639, 1.468201

C	-0.750525	1.492494	-0.098584
N	0.124227	0.928789	0.935325
C	0.545345	-0.411685	0.683593
C	-0.642977	-1.352152	0.564221
C	-1.619058	-0.875367	-0.515790
C	-1.893034	0.581910	-0.380857
O	1.257756	-0.574399	-0.558456
O	2.400210	0.271250	-0.502495
H	-0.188305	1.674541	-1.030774
H	-1.111037	2.459418	0.251479
H	0.932835	1.519457	1.074229
H	1.216165	-0.715853	1.490092
H	-0.287841	-2.359190	0.349326
H	-1.136999	-1.361256	1.535400
H	-2.543069	-1.451590	-0.467859
H	-1.175260	-1.079595	-1.499827
H	3.115298	-0.374834	-0.472012
H	-2.793600	1.001377	-0.805223

SP_{ea}^{9e}

T_1= 0.01944377, D_1= 0.10518173

$\tilde{\nu}$: -1733.0, 186.8, 236.6, 265.9, 393.8, 440.8, 458.3, 526.3, 563.4, 638.2, 762.8, 778.8, 819.8, 846.5, 889.9, 933.8, 937.7, 954.3, 1045.2, 1058.7, 1085.1, 1113.8, 1135.0, 1182.3, 1201.3, 1223.0, 1287.5, 1299.5, 1333.1, 1358.5, 1366.3, 1387.3, 1397.8,

C	-0.535216	-1.360551	-0.033055
N	0.247732	-0.889326	-1.114286
C	0.835195	0.396117	-0.795688
C	-0.250764	1.425365	-0.508188
C	-1.347317	0.958464	0.456790
C	-1.755753	-0.495423	0.197338
O	1.769191	0.178674	0.262399
O	1.103124	-0.206549	1.419617

1428.4, 1471.4, 1481.3, 1492.8, 1503.6, 1633.7, 3040.5, 3068.2, 3087.0, 3115.5, 3122.8, 3124.8, 3148.9, 3151.2, 3563.9

B: 2.912618, 2.116550, 1.936797

H	0.271392	-1.053604	0.963749
H	-0.703040	-2.432873	-0.066208
H	0.963884	-1.546480	-1.396497
H	1.468790	0.722736	-1.618387
H	0.227622	2.330572	-0.136340
H	-0.694863	1.662662	-1.477441
H	-2.215380	1.609926	0.355577
H	-0.986687	1.050042	1.477686
H	-2.335874	-0.881653	1.034351
H	-2.385354	-0.556871	-0.695801

POST_{ea}^{9e}

T_1= 0.01540992, D_1= 0.06704297

$\tilde{\nu}$: 117.4, 154.6, 220.7, 274.7, 330.6, 350.9, 437.8, 459.6, 498.5, 510.9, 670.8, 686.4, 763.3, 805.6, 873.1, 898.9, 936.2, 947.0, 1032.1, 1049.3, 1085.3, 1103.4, 1122.0, 1189.3, 1199.9, 1255.2, 1301.5, 1344.8, 1369.5, 1371.0, 1389.0, 1392.8, 1436.3, 1457.0, 1468.7, 1478.7, 1503.9, 1517.3, 2963.0, 3063.8, 3083.3, 3096.4, 3111.2, 3125.0, 3144.0, 3199.1, 3608.5, 3728.0

B: 3.020499, 1.904663, 1.704398

C	1.020204	1.276160	0.321817
N	0.022594	1.259214	-0.652843
C	-0.751557	0.067695	-0.882650
C	0.116408	-1.185570	-0.830370
C	1.146943	-1.210733	0.299481
C	1.935473	0.098295	0.336841
O	-1.902132	0.018393	-0.039916
O	-1.510308	-0.238628	1.300356
H	-1.058461	0.580851	1.557037
H	1.407158	2.259687	0.549496
H	-0.520519	2.105107	-0.735956
H	-1.228417	0.147607	-1.860849
H	-0.549859	-2.045217	-0.773307
H	0.641547	-1.238151	-1.787085
H	1.825298	-2.049981	0.143710
H	0.646084	-1.363773	1.252504
H	2.579321	0.138669	1.214357
H	2.594384	0.137492	-0.544242

SP_{ee}^{9a}

T_1= 0.02541616, D_1= 0.12547616

$\tilde{\nu}$: -628.8, 67.9, 139.5, 162.8, 183.7, 311.8, 379.8, 442.5, 478.9, 541.2, 650.5, 763.9, 817.4, 850.3, 887.6, 911.4, 931.5, 985.9, 1040.6, 1075.5, 1091.6, 1128.0, 1195.5, 1221.4, 1283.1, 1316.3, 1323.5, 1357.6, 1374.8, 1390.3, 1396.3, 1398.3, 1456.8, 1489.2, 1494.6, 1509.9, 1546.5, 1675.0, 2070.7, 3059.9, 3062.8, 3066.6, 3072.3, 3095.3, 3121.1, 3122.7, 3127.7, 3189.5

B: 3.216651, 1.612754, 1.381312

C	-0.550260	-1.461284	0.368853
N	0.361731	-0.496125	1.001733
C	0.211397	0.776366	0.794485
C	-0.972503	1.416949	0.151335
C	-2.021106	0.410717	-0.317180
C	-1.327682	-0.860713	-0.790874
O	1.875482	0.677339	-0.744330
O	2.399036	-0.396901	-0.282322
H	-1.234514	-1.830653	1.135109
H	0.060010	-2.301663	0.042742
H	1.445703	-0.696387	0.726247
H	0.940799	1.427266	1.260653
H	-1.391918	2.145092	0.848684
H	-0.579251	1.995584	-0.688529
H	-2.630574	0.848800	-1.105626
H	-2.692740	0.164024	0.508176
H	-2.048494	-1.588154	-1.162312
H	-0.636357	-0.626746	-1.603766

POST_{ee}^{9a}

T_1= 0.02607240, D_1= 0.12320140

$\tilde{\nu}$: 29.4, 54.7, 82.6, 145.1, 162.0, 270.8, 288.0, 417.4, 467.9, 530.6, 711.0, 809.8, 832.6, 854.3, 874.1, 910.0, 932.5, 992.1, 1038.6, 1069.2, 1089.3, 1126.8, 1197.9, 1219.1, 1279.9, 1291.5,

C	-0.717665	-1.378668	0.117421
N	0.318396	-0.347672	0.050889
C	0.004491	0.875304	-0.046313
C	-1.386290	1.441791	-0.073244
C	-2.437971	0.407175	0.307234
C	-2.087917	-0.919123	-0.355664
O	3.236770	0.649494	-0.071403

1308.4, 1358.2, 1377.9, 1387.3, 1395.6, 1422.6, 1465.7, 1489.4, 1493.0, 1508.1, 1667.3, 1774.1, 2860.4, 3050.5, 3055.4, 3060.5, 3067.3, 3099.2, 3106.6, 3117.1, 3121.9, 3143.9

B: 4.290951 0.987860 0.830083

O	2.965575	-0.623014	0.066297
H	-0.764633	-1.720161	1.154963
H	-0.363126	-2.224988	-0.470474
H	1.950308	-0.653694	0.076621
H	0.834381	1.576157	-0.113810
H	-1.411754	2.311844	0.584698
H	-1.563355	1.826829	-1.082546
H	-3.431055	0.747325	0.018034
H	-2.445005	0.275982	1.391749
H	-2.828971	-1.685357	-0.130165
H	-2.072213	-0.790941	-1.441055

SP$_{ee}^{9b}$

T_1= 0.02607240, D_1= 0.12320140

$\tilde{\nu}$: -1013.7, 95.1, 104.2, 171.2, 195.9, 314.0, 342.9, 461.2, 487.0, 529.1, 561.1, 702.8, 746.7, 854.1, 859.0, 908.0, 924.1, 960.0, 972.1, 1054.3, 1079.9, 1111.9, 1136.4, 1204.0, 1226.8, 1293.0, 1312.2, 1334.6, 1344.1, 1356.1, 1384.1, 1398.9, 1415.2, 1493.3, 1499.6, 1519.0, 1555.2, 1594.4, 1641.3, 3046.3, 3054.5, 3076.8, 3109.4, 3114.9, 3129.1, 3163.5, 3238.4, 3656.2

B: 2.853733, 1.660213, 1.452210

C	-1.672129	-0.816138	-0.543213
N	-0.668204	-1.373824	0.362261
C	0.203864	-0.629059	1.037793
C	0.193216	0.779664	1.027536
C	-0.992990	1.428246	0.335186
C	-1.327248	0.618565	-0.916282
O	2.067982	-0.668558	-0.457298
O	2.148917	0.594204	-0.652475
H	-2.650571	-0.855705	-0.058504
H	-1.714983	-1.445175	-1.431187
H	-0.499519	-2.363874	0.317818
H	0.884004	-1.173010	1.675695
H	0.549297	1.235234	1.945497
H	1.191165	0.933398	0.231485
H	-0.751114	2.454387	0.060719
H	-1.865230	1.468494	0.993634
H	-2.167075	1.052335	-1.457388
H	-0.462018	0.617853	-1.581532

POST$_{ee}^{9b}$

T_1= 0.02230282, D_1= 0.12959867

$\tilde{\nu}$: 36.4, 53.6, 72.6, 118.4, 156.2, 212.0, 291.1, 393.3, 457.7, 511.9, 540.3, 596.7, 751.9, 760.9, 859.9, 882.3, 921.9, 970.8, 987.8, 1037.9, 1076.6, 1107.6, 1139.9, 1197.8, 1240.6, 1277.9, 1278.5, 1319.0, 1351.0, 1372.6, 1396.7, 1437.5, 1487.2, 1489.4, 1506.7, 1515.6, 1534.8, 1704.2, 3041.7, 3045.6, 3067.2, 3087.2, 3121.1, 3126.8, 3195.2, 3216.7, 3255.7, 3648.2

B: 2.676857, 1.434669, 1.312908

C	1.526173	1.052697	-0.532601
N	0.564339	1.380516	0.511492
C	0.041268	0.360304	1.265839
C	0.365762	-0.934507	1.106236
C	1.254842	-1.380801	-0.025184
C	1.259577	-0.325115	-1.131716
O	-2.507176	0.588085	-0.379993
O	-2.242060	-0.686352	-0.489767
H	2.531786	1.067831	-0.102763
H	1.485249	1.827645	-1.295243
H	-0.050010	2.158448	0.343013
H	-0.652913	0.673710	2.035449
H	0.005436	-1.655899	1.826371
H	-1.445980	-0.829001	0.086232
H	0.903954	-2.335134	-0.422336
H	2.275701	-1.554294	0.326912
H	2.015325	-0.553299	-1.882354
H	0.289248	-0.312940	-1.633086

SP$_{ee}^{9d}$

T_1= 0.01919648, D_1= 0.10347875

$\tilde{\nu}$: -2033.7, 148.4, 196.6, 283.0, 347.6, 395.1, 465.2, 507.9, 563.8, 660.9, 732.7, 779.9, 810.0, 847.6, 904.1, 915.3, 926.8,

C	1.126222	1.158730	-0.466264
N	0.350083	1.286200	0.776280
C	-0.725773	0.339058	0.872656
C	-0.157208	-1.055909	1.095826
C	1.118665	-1.265119	0.249834
C	1.139318	-0.286357	-0.898112

963.0, 1027.0, 1053.5, 1078.7, 1092.5, 1105.8, 1184.4, 1201.3, 1210.4, 1254.6, 1265.3, 1297.6, 1342.2, 1356.2, 1360.1, 1382.5, 1424.4, 1472.1, 1489.5, 1499.1, 1512.1, 1530.5, 3021.0, 3041.1, 3047.7, 3104.3, 3119.9, 3122.5, 3151.2, 3156.6, 3583.6

B: 2.646643, 2.252147, 2.081689

O	-1.595255	0.407927	-0.290655
O	-1.279909	-0.558978	-1.238212
H	2.148928	1.504123	-0.283079
H	0.711606	1.773844	-1.271684
H	0.006800	2.229909	0.883711
H	-1.379119	0.639914	1.689464
H	0.076361	-1.147745	2.154543
H	-0.915898	-1.794050	0.848277
H	1.171360	-2.289055	-0.115245
H	2.004257	-1.095044	0.869988
H	1.802965	-0.507221	-1.729027
H	-0.083868	-0.452085	-1.373615

POST_{ee}^{9d}

T_1= 0.0358, D_1= 0.1228

$\tilde{\nu}$: 66.6, 152.4, 183.3, 207.5, 241.5, 271.6, 304.7, 403.1, 427.8, 485.4, 548.9, 679.5, 719.6, 818.9, 888.2, 916.8, 935.5, 963.8, 1005.8, 1054.4, 1057.2, 1084.9, 1103.3, 1197.7, 1211.9, 1239.6, 1251.8, 1332.8, 1340.7, 1356.4, 1384.2, 1388.8, 1406.5, 1414.7, 1477.1, 1487.7, 1493.6, 1508.1, 2938.8, 2993.2, 3060.5, 3062.5, 3090.5, 3103.0, 3139.5, 3227.0, 3612.0, 3834.7

B: 3.401337, 1.720084, 1.481788

C	0.869824	1.408964	-0.089860
N	0.044710	0.828033	0.962896
C	-0.578972	-0.434859	0.733201
C	0.262483	-1.317283	-0.187038
C	1.752577	-0.980791	-0.081338
C	1.954646	0.448880	-0.443074
O	-1.937997	-0.354591	0.266608
O	-1.949859	0.263235	-1.016329
H	1.293134	2.340800	0.292781
H	0.289693	1.675045	-0.988575
H	-0.556741	1.483247	1.437607
H	-0.752231	-0.924947	1.695295
H	0.066456	-2.361205	0.050835
H	-0.067509	-1.143824	-1.211011
H	2.335055	-1.634585	-0.730211
H	2.092656	-1.175196	0.944600
H	2.802755	0.781053	-1.021374
H	-2.276727	1.144764	-0.803792

SP_{ae}^{9a}

T_1= 0.02541663, D_1= 0.12547452

$\tilde{\nu}$: -628.8, 67.9, 139.5, 162.8, 183.7, 311.8, 379.8, 442.5, 478.9, 541.2, 650.5, 763.9, 817.4, 850.3, 887.6, 911.4, 931.5, 985.9, 1040.6, 1075.5, 1091.6, 1128.0, 1195.5, 1221.4, 1283.1, 1316.3, 1323.5, 1357.6, 1374.8, 1390.3, 1396.3, 1398.3, 1456.8, 1489.2, 1494.6, 1509.9, 1546.5, 1675.0, 2070.7, 3059.9, 3062.8, 3066.6, 3072.3, 3095.3, 3121.1, 3122.7, 3127.7, 3189.5

B: 3.216651 1.612755 1.381312

C	-0.211397	0.776366	0.794485
N	-0.361731	-0.496125	1.001733
C	0.550260	-1.461284	0.368853
C	1.327682	-0.860713	-0.790874
C	2.021106	0.410717	-0.317180
C	0.972503	1.416949	0.151335
O	-1.875481	0.677339	-0.744331
O	-2.399036	-0.396900	-0.282322
H	-0.940799	1.427266	1.260653
H	-1.445703	-0.696387	0.726247
H	-0.060010	-2.301663	0.042742
H	1.234514	-1.830653	1.135109
H	2.048493	-1.588154	-1.162312
H	0.636357	-0.626746	-1.603766
H	2.630574	0.848800	-1.105626
H	2.692741	0.164024	0.508176
H	1.391918	2.145092	0.848684
H	0.579251	1.995585	-0.688529

POST_{ae}^{9a} (same as POST_{ee}^{13a})

SP_{ae}^{13b}

T_1= 0.02022743, D_1= 0.11027473

C	0.684602	-0.305679	-0.717568
N	-0.041619	-1.352053	-0.030566
C	-1.487457	-1.151681	-0.058851

$\tilde{\nu}$: -2144.7, 127.0, 182.9, 280.5, 334.0, 404.5, 442.8, 506.4, 541.6, 612.4, 730.4, 777.1, 816.0, 871.8, 877.9, 941.2, 953.5, 972.0, 989.9, 1053.8, 1077.9, 1093.4, 1138.1, 1160.2, 1196.8, 1220.2, 1252.9, 1288.4, 1300.8, 1338.7, 1359.9, 1380.4, 1383.2, 1396.2, 1462.9, 1484.0, 1495.8, 1502.3, 1749.7, 2998.7, 3041.1, 3056.6, 3057.3, 3087.5, 3109.3, 3117.5, 3122.7, 3536.3

B: 3.552576 1.752143 1.444344

C	-1.915151	0.174725	0.564895
C	-1.264489	1.335525	-0.183073
C	0.202734	1.116688	-0.398622
O	2.029734	-0.268215	-0.291620
O	1.950543	0.316301	0.979627
H	0.697562	-0.513665	-1.791790
H	0.286298	-1.370306	0.929695
H	-1.957231	-1.992976	0.448250
H	-1.814195	-1.188859	-1.103215
H	-3.000712	0.276893	0.545806
H	-1.604517	0.187568	1.612869
H	-1.436912	2.289051	0.318794
H	-1.737252	1.429738	-1.170526
H	0.708458	1.877425	-0.991540
H	0.986190	1.067351	0.670877

POST_{ae}^{9b}

T_1= 0.01252660, D_1= 0.03513690

$\tilde{\nu}$: 77.5, 141.0, 204.6, 247.8, 288.6, 340.2, 390.9, 435.2, 473.9, 501.4, 651.7, 704.7, 788.4, 861.9, 876.9, 905.7, 954.5, 972.5, 986.7, 1075.8, 1093.9, 1105.1, 1114.4, 1167.8, 1217.5, 1243.9, 1287.5, 1319.6, 1327.8, 1366.8, 1371.3, 1385.8, 1398.7, 1403.2, 1471.7, 1477.2, 1493.3, 1497.9, 2965.9, 2983.4, 3039.9, 3056.6, 3092.0, 3106.4, 3112.9, 3175.3, 3529.7, 3811.2

B: 3.718603 1.561346 1.319316

C	0.607438	-0.050667	-0.679739
N	0.027847	-1.248335	-0.097672
C	-1.431417	-1.218549	-0.137590
C	-2.030230	-0.008646	0.575943
C	-1.492278	1.284497	-0.040419
C	-0.013035	1.228354	-0.213079
O	2.011433	-0.046796	-0.503724
O	2.284857	-0.009723	0.891407
H	0.515215	-0.135175	-1.773274
H	0.344015	-1.299368	0.865639
H	-1.802216	-2.147584	0.293549
H	-1.737248	-1.211899	-1.188983
H	-3.119278	-0.030344	0.518203
H	-1.755952	-0.051787	1.632999
H	-1.777613	2.155518	0.550472
H	-1.966921	1.420554	-1.024522
H	0.542191	2.142508	-0.386421
H	2.349695	0.938147	1.063882

SP_{ae}^{9c}

T_1= 0.02437448, D_1= 0.14604008

$\tilde{\nu}$: -2259.1, 168.2, 225.9, 301.5, 350.3, 357.0, 405.0, 496.9, 545.7, 596.9, 677.0, 810.5, 844.9, 857.8, 902.1, 940.0, 955.2, 997.6, 1038.7, 1071.7, 1084.1, 1122.7, 1152.9, 1164.5, 1206.9, 1218.7, 1227.9, 1308.6, 1312.8, 1339.6, 1363.5, 1367.4, 1384.6, 1404.8, 1476.1, 1488.1, 1496.3, 1504.2, 1768.6, 3039.1, 3055.0, 3062.2, 3065.7, 3085.3, 3107.3, 3124.0, 3126.4, 3515.5

B: 4.003878 1.620016 1.242737

C	-0.584004	-0.423224	0.260125
N	0.359336	-1.414267	-0.167023
C	1.696412	-0.988114	0.270396
C	2.090421	0.423968	-0.190590
C	1.016803	1.478825	0.178039
C	-0.286498	0.945856	-0.329482
O	-1.899775	-0.680581	-0.177003
O	-2.552294	0.498564	0.181020
H	-0.559484	-0.366567	1.354369
H	0.328658	-1.492455	-1.179419
H	2.420973	-1.717558	-0.087300
H	1.707948	-1.032900	1.362019
H	3.051924	0.699449	0.245235
H	2.216663	0.419698	-1.276271
H	1.275199	2.443806	-0.255127
H	0.982816	1.593682	1.264204
H	-1.546382	1.250936	-0.002925
H	-0.375924	0.934051	-1.418686

POST_{ae}^{9c}

C	0.597738	-0.289650	0.228278
N	0.196137	1.027602	-0.273765

T_1= 0.01265730, D_1= 0.03706097

$\tilde{\nu}$: 114.2, 149.1, 204.6, 243.3, 330.8, 365.4, 385.7, 434.8, 479.1, 524.0, 573.3, 649.7, 829.9, 859.5, 887.4, 941.3, 964.9, 980.2, 1031.4, 1071.5, 1103.8, 1105.2, 1147.4, 1170.6, 1214.6, 1238.9, 1285.9, 1312.9, 1343.0, 1366.7, 1372.1, 1387.6, 1409.6, 1462.7, 1472.8, 1475.5, 1492.4, 1498.9, 2972.0, 2976.3, 3052.8, 3058.9, 3106.8, 3109.6, 3116.3, 3220.7, 3481.7, 3731.3

B: 4.269257 1.452506 1.156555

C	-1.141318	1.371322	0.214735
C	-2.197005	0.353362	-0.216618
C	-1.798933	-1.067150	0.209115
C	-0.374561	-1.355524	-0.126383
O	1.848778	-0.618591	-0.315654
O	2.800449	0.287532	0.223600
H	0.696070	-0.189757	1.320418
H	0.158152	0.949137	-1.288290
H	-1.396817	2.368982	-0.139873
H	-1.091649	1.420397	1.305999
H	-3.169110	0.614604	0.203413
H	-2.292115	0.388459	-1.304917
H	-2.460711	-1.806360	-0.240146
H	-1.938099	-1.150290	1.297693
H	2.430603	1.144075	-0.044722
H	-0.018623	-2.358156	-0.311547

SP_{ae}^{9d}

T_1= 0.01915587, D_1= 0.10310130

$\tilde{\nu}$: -2033.7, 148.4, 196.6, 283.0, 347.6, 395.1, 465.2, 507.9, 563.8, 660.9, 732.7, 779.9, 810.0, 847.6, 904.1, 915.3, 926.8, 963.0, 1027.0, 1053.5, 1078.7, 1092.5, 1105.8, 1184.4, 1201.3, 1210.4, 1254.6, 1265.3, 1297.6, 1342.2, 1356.2, 1360.1, 1382.5, 1424.4, 1472.1, 1489.5, 1499.1, 1512.1, 1530.5, 3021.0, 3041.1, 3047.7, 3104.3, 3119.9, 3122.5, 3151.2, 3156.6, 3583.6

B: 2.646645 2.252147 2.081689

C	0.725773	0.339055	0.872658
N	-0.350084	1.286198	0.776283
C	-1.126221	1.158731	-0.466263
C	-1.139317	-0.286355	-0.898113
C	-1.118667	-1.265118	0.249832
C	0.157208	-1.055913	1.095822
O	1.595256	0.407928	-0.290653
O	1.279910	-0.558975	-1.238212
H	1.379117	0.639909	1.689467
H	-0.006802	2.229906	0.883718
H	-0.711602	1.773846	-1.271680
H	-2.148927	1.504125	-0.283078
H	-1.802962	-0.507217	-1.729030
H	0.083870	-0.452080	-1.373615
H	-1.171367	-2.289054	-0.115247
H	-2.004257	-1.095038	0.869987
H	-0.076358	-1.147755	2.154539
H	0.915897	-1.794052	0.848267

$POST_{ae}^{9d}$

T_1= 0.01268453, D_1= 0.03611240

$\tilde{\nu}$: 66.6, 152.4, 183.3, 207.5, 241.5, 271.6, 304.7, 403.1, 427.8, 485.4, 548.9, 679.5, 719.6, 818.9, 888.2, 916.8, 935.5, 963.8, 1005.8, 1054.4, 1057.2, 1084.9, 1103.3, 1197.7, 1211.9, 1239.6, 1251.8, 1332.8, 1340.7, 1356.4, 1384.2, 1388.8, 1406.5, 1414.7, 1477.1, 1487.7, 1493.6, 1508.1, 2938.8, 2993.2, 3060.5, 3062.5, 3090.5, 3103.0, 3139.5, 3227.0, 3612.0, 3834.7

B: 3.401337 1.720084 1.481788

C	0.578972	-0.434859	0.733201
N	-0.044710	0.828033	0.962896
C	-0.869824	1.408964	-0.089860
C	-1.954646	0.448880	-0.443074
C	-1.752577	-0.980791	-0.081338
C	-0.262483	-1.317283	-0.187038
O	1.937997	-0.354591	0.266608
O	1.949859	0.263234	-1.016329
H	0.752231	-0.924947	1.695295
H	0.556742	1.483248	1.437607
H	-0.289693	1.675044	-0.988575
H	-1.293134	2.340800	0.292781
H	-2.802756	0.781053	-1.021372
H	2.276728	1.144763	-0.803792
H	-2.335055	-1.634585	-0.730211
H	-2.092656	-1.175196	0.944600
H	-0.066456	-2.361205	0.050835
H	0.067509	-1.143823	-1.211011

Atmospheric fate of the 3-piperidinyl radical

Table S12. QCC results for the PIPC^{3}OȮ conformational pathways and internal H-transfer reactions
Energies (/Hartree) of the 3-piperidinyl peroxy radical (PIPC^{3}OȮ) conformations and stationary points on the internal H-transfer reactions in PIPC^{3}OȮ, and energy differences of stationary points on the potential energy surface relative to that of the initial reactants.

Species	M06-2X/aTZ		CCSD(T*)-F12a/aTZ	
	E_{Elec}	E_{ZPE}	E_{Elec}	$\Delta E_{v=0}$
PIPĊ$_a^3$	-251.20881	0.14524	-250.89232	
O_2	-150.32480	0.00400	-150.19114	
Sum reactants	-401.53361	0.14924	-401.08346	0.0
PIPC^{3}OȮ$_{aa}$	-401.59771	0.15646	-401.14899	-153.1
SP_{aa}^{12a}	-401.55938	0.15086	-401.11398	-75.9
$POST_{aa}^{12a}$	-401.57723	0.15474	-401.12950	-106.4
SP_{aa}^{12b}	-401.53622	0.14991	-401.09187	-20.4
$POST_{aa}^{12b}$	-401.58030	0.15256	-401.13166	-117.8
1,2,3,4-tetrahydropyridine	-250.65862	0.13612	-250.34020	
HO_2	-150.90809	0.01458	-150.77891	
Sum products	-401.56671	0.15070	-401.11911	-89.8
SP_{aa}^{12c}	-401.53661	0.15008	-401.09205	-20.4
$POST_{aa}^{12c}$	-401.56972	0.15242	-401.12240	-93.9
1,2,3,6-tetrahydropyridine	-250.64888	0.13603	-250.33142	
HO_2	-150.90809	0.01458	-150.77891	
Sum products	-401.55697	0.15061	-401.11033	-67.0
SP_{aa}^{12d}	-401.54668	0.15032	-401.09965	-39.7
$POST_{aa}^{14d}$	-401.56974	0.15373	-401.11990	-83.9
SP_{aa-ea}	-401.59068	0.15508	-401.14088	-135.4
PIPC^{3}OȮ$_{ea}$	-401.59654	0.15653	-401.14775	-149.7
SP_{ea}^{12b}	-401.53411	0.14979	-401.08985	-15.4
$POST_{ea}^{12b}$	-401.58714	0.15497	-401.13843	-129.3
SP_{ea}^{12c}	-401.53591	0.15024	-401.09135	-18.1
$POST_{ea}^{12c}$	-401.58050	0.15342	-401.13331	-119.9
SP_{ea}^{12d}	-401.53572	0.14965	-401.08778	-10.3

$POST_{ea}^{12d}$	-401.57281	0.15423	-401.13843	-131.2
$PIPC^3O\dot{O}_{ee}$	-401.59736	0.15670	-401.14861	-151.5
SP_{ee}^{12b}	-401.55213	0.15114	-401.10499	-51.5
$POST_{ee}^{12b}$	-401.58551	0.15498	-401.13607	-123.1
SP_{ee}^{12b2}	-401.56058	0.15258	-401.11128	-64.3
$POST_{ee}^{12b2}$	-401.58380	0.15328	-401.13638	-128.3
1,2,3,4-tetrahydropyridine	-250.65862	0.13612	-250.34020	
HO_2	-150.90809	0.01458	-150.77891	
Sum products	-401.56671	0.15070	-401.11911	-89.8
SP_{ee}^{12c}	-401.53482	0.15039	-401.08904	-11.6
$POST_{ee}^{12c}$	-401.57008	0.15369	-401.12213	-89.8
SP_{ee-ae}	-401.59027	0.15522	-401.14046	-134.0
$PIPC^3O\dot{O}_{ae}$	-401.59620	0.15643	-401.14757	-149.5
SP_{ae}^{12b}	-401.53153	0.15079	-401.08628	-3.3
$POST_{ae}^{12b}$	-401.56986	0.15415	-401.12088	-85.4
SP_{ae}^{12c}	-401.53293	0.15011	-401.08719	-7.5
$POST_{ae}^{12c}$	-401.57056	0.15368	-401.12124	-87.6

Table S11, continued.
T_1 and D_1 diagnostic values, vibrational frequencies (cm^{-1}), Rotational constants (GHz) and Cartesian coordinates of the species listed above. Results from CCSD(T*)-F12a/aug-cc-pVTZ//M06-2X/aug-cc-pVTZ calculations.

PIP-$\dot{C}^3{}_a$	C	-1.380377	0.479659	0.169736
	N	-1.120698	-0.914333	-0.206417
T_1= 0.01179107, D_1= 0.03716585	C	0.189154	-1.357233	0.261987
	C	1.343917	-0.488258	-0.235180
$\tilde{\nu}$: 160.7, 221.9, 322.2, 386.7, 448.4, 488.1, 643.5, 787.3, 831.8,	C	1.117581	0.977459	0.155060
869.3, 881.0, 904.1, 952.8, 1011.4, 1053.3, 1093.0, 1121.7,	C	-0.267877	1.408667	-0.185180
1150.0, 1165.3, 1236.6, 1279.3, 1323.4, 1344.5, 1359.4, 1375.4,	H	-1.540851	0.483570	1.258760
1386.4, 1398.8, 1468.6, 1474.4, 1478.8, 1493.0, 1495.8, 2960.5,	H	-2.320821	0.795720	-0.280573
2968.7, 3040.0, 3049.4, 3097.5, 3099.0, 3104.3, 3104.9, 3200.8,	H	-1.148007	-0.986027	-1.217928
3523.1	H	0.331789	-2.395335	-0.038675
	H	0.166510	-1.340789	1.356493
B: 4.761538 4.483942 2.587622	H	2.291678	-0.848723	0.168493
	H	1.400660	-0.566667	-1.324288
	H	1.859240	1.625693	-0.311317
	H	1.269820	1.065924	1.242450
	H	-0.479513	2.445195	-0.407033
$PIP\dot{C}^3{}_e$	C	0.080678	-1.360242	0.251426
	N	1.208510	-0.638732	-0.324674

T$_1$= 0.01158722, D$_1$= 0.03427274

$\tilde{\nu}$: 170.5, 223.5, 340.2, 383.4, 441.7, 480.6, 631.5, 775.8, 844.2, 872.7, 900.5, 929.3, 944.9, 1028.0, 1069.4, 1099.3, 1117.0, 1171.4, 1196.0, 1202.8, 1273.9, 1298.4, 1341.3, 1359.8, 1364.9, 1399.6, 1423.1, 1471.4, 1481.8, 1485.3, 1492.4, 1509.1, 2867.4, 2943.6, 2956.1, 3068.8, 3097.0, 3098.3, 3103.2, 3116.4, 3207.8, 3562.6

B: 4.801706 4.507795 2.582316

C	1.253492	0.751762	0.126371
C	-0.034145	1.439805	-0.167637
C	-1.299811	0.727183	0.161043
C	-1.220548	-0.747406	-0.246535
H	0.090318	-1.328586	1.354252
H	0.148003	-2.405458	-0.049290
H	2.076223	-1.101190	-0.089303
H	2.081801	1.258210	-0.368710
H	1.446977	0.807519	1.217362
H	-0.033856	2.511469	-0.303826
H	-2.155638	1.216279	-0.304496
H	-1.474150	0.771893	1.248055
H	-2.072133	-1.297730	0.154941
H	-1.245112	-0.827887	-1.334275

O_2
T$_1$= 0.00775897, D$_1$= 0.01442723
$\tilde{\nu}$: 1754.5
B: 44.635532

O	0.000000	0.000000	0.594925
O	0.000000	0.000000	-0.594925

PIP-C^{3}OȮ$_{aa}$

T$_1$= 0.02368875, D$_1$= 0.14171092

$\tilde{\nu}$: 83.5, 154.2, 193.1, 300.7, 331.8, 395.2, 438.0, 508.1, 565.2, 685.8, 764.7, 835.7, 849.7, 877.6, 897.5, 936.8, 967.7, 1035.1, 1060.8, 1085.4, 1149.7, 1166.1, 1201.8, 1241.5, 1285.4, 1295.0, 1341.8, 1345.3, 1356.0, 1363.2, 1388.5, 1392.9, 1407.4, 1473.9, 1481.8, 1486.0, 1498.4, 1500.2, 3039.3, 3054.2, 3062.3, 3064.7, 3101.2, 3107.4, 3112.3, 3116.2, 3131.7, 3544.9

B: 3.397387, 1.731270, 1.435735

C	-0.134795	-1.077092	0.799136
N	0.841292	-1.432689	-0.215207
C	1.931240	-0.466108	-0.299328
C	1.454123	0.968077	-0.520733
C	0.453250	1.368495	0.562775
C	-0.664750	0.348545	0.684283
O	-1.451300	0.473840	-0.536641
O	-2.535435	-0.236173	-0.489913
H	0.333535	-1.174274	1.783039
H	-0.971118	-1.773054	0.760782
H	0.383952	-1.520422	-1.115001
H	2.608841	-0.776089	-1.093930
H	2.491029	-0.522597	0.639947
H	2.300951	1.655473	-0.519170
H	0.979192	1.041492	-1.501628
H	0.031245	2.355091	0.370763
H	0.957133	1.413742	1.531588
H	-1.344340	0.576621	1.505692

SP$^{12a}_{aa}$

T$_1$= 0.01164729 , D$_1$= 0.03411667

$\tilde{\nu}$: -1850.5, 190.1, 217.5, 270.6, 373.5, 439.6, 448.9, 527.5, 567.2, 630.2, 768.1, 809.6, 827.9, 874.3, 903.1, 942.4, 950.9, 1022.2, 1030.8, 1060.3, 1093.3, 1107.5, 1148.4, 1187.6, 1232.5, 1241.7, 1286.6, 1301.7, 1331.2, 1357.7, 1360.7, 1371.4, 1377.7, 1405.2, 1471.7, 1487.4, 1495.7, 1513.3, 1731.1, 3038.1, 3049.0, 3058.0, 3087.6, 3104.4, 3112.1, 3121.1, 3138.9, 3154.8

B: 2.906256, 2.164414, 1.968122

C	-0.224513	-0.966778	1.111430
N	0.508939	-1.374566	-0.049524
C	1.691117	-0.538921	-0.217298
C	1.336599	0.940606	-0.463645
C	0.247467	1.438533	0.490323
C	-0.857655	0.425168	0.782028
O	-1.762628	0.182920	-0.260670
O	-1.067821	-0.232438	-1.392604
H	0.431558	-0.858622	1.980242
H	-1.023331	-1.671919	1.329515
H	-0.296918	-1.083697	-0.937481
H	2.283246	-0.924844	-1.045040
H	2.289665	-0.625953	0.694949
H	2.240366	1.538400	-0.342457
H	0.999565	1.053708	-1.489793
H	-0.209804	2.347795	0.099756
H	0.692122	1.690442	1.456718
H	-1.483529	0.761142	1.609424

$POST_{aa}^{12a}$

T_1= 0.01544998, D_1= 0.07032523

$\tilde{\nu}$: 133.9, 154.5, 235.8, 273.8, 313.8, 407.1, 435.1, 441.1, 496.2, 545.2, 752.8, 797.0, 829.9, 874.0, 887.3, 949.2, 961.5, 1019.2, 1037.3, 1059.7, 1096.6, 1112.8, 1149.5, 1192.4, 1237.0, 1255.2, 1289.8, 1301.0, 1332.1, 1350.1, 1372.1, 1384.5, 1395.9, 1461.3, 1472.1, 1490.4, 1497.2, 1518.2, 2985.5, 2996.1, 3055.4, 3086.4, 3089.6, 3113.7, 3116.0, 3116.3, 3152.1, 3701.0

B: 2.970478, 1.956246, 1.794474

C	-0.050981	1.322864	0.692775
N	-0.827369	1.242937	-0.513375
C	-1.847419	0.232880	-0.382764
C	-1.194759	-1.158937	-0.285064
C	-0.161705	-1.203843	0.841362
C	0.750609	0.018769	0.904815
O	1.868868	-0.029373	0.037345
O	1.443062	-0.256236	-1.297898
H	-0.701463	1.454395	1.568830
H	0.645779	2.158587	0.640689
H	1.021863	0.584721	-1.543874
H	-2.515304	0.275942	-1.241174
H	-2.437840	0.411228	0.527300
H	-1.976824	-1.897807	-0.108240
H	-0.722923	-1.393603	-1.235520
H	0.458857	-2.096308	0.761089
H	-0.684852	-1.255761	1.800064
H	1.234378	0.072519	1.882141

SP_{aa}^{12b}

T_1= 0.02351496, D_1= 0.12097872

$\tilde{\nu}$: -1130.0, 99.8, 135.5, 203.2, 250.0, 342.1, 445.4, 475.0, 484.0, 562.4, 687.8, 724.5, 754.1, 834.9, 865.7, 903.7, 946.5, 965.4, 1003.6, 1041.4, 1073.5, 1115.1, 1123.3, 1210.6, 1246.1, 1262.0, 1301.5, 1312.9, 1352.3, 1378.2, 1386.6, 1402.6, 1412.1, 1465.9, 1480.2, 1494.9, 1499.9, 1575.1, 1617.1, 3035.2, 3038.1, 3062.8, 3106.1, 3114.8, 3120.3, 3121.3, 3201.8, 3566.0

B: 3.079170, 1.714755, 1.432369

C	-0.146371	-1.002155	0.791313
N	0.988943	-1.430177	0.003480
C	1.951002	-0.384031	-0.318204
C	1.291964	0.933560	-0.721241
C	0.464562	1.441888	0.452595
C	-0.386861	0.352593	1.021423
O	-1.952284	0.621300	-0.328652
O	-2.153371	-0.559435	-0.711523
H	-0.418185	-1.683986	1.594195
H	-1.210653	-1.101085	0.029053
H	0.691130	-1.908703	-0.835003
H	2.604593	-0.757224	-1.104915
H	2.574527	-0.210361	0.564597
H	2.042092	1.671332	-1.006082
H	0.644204	0.766514	-1.585262
H	-0.157673	2.293664	0.178277
H	1.132486	1.784792	1.250745
H	-1.025654	0.610246	1.856124

$POST_{aa}^{12b}$

T_1= 0.02212913, D_1= 0.12896070

$\tilde{\nu}$: 33.6, 57.6, 84.9, 107.5, 168.4, 201.6, 294.2, 422.1, 462.5, 484.7, 522.6, 551.5, 741.2, 777.0, 860.6, 891.8, 923.3, 981.2, 989.9, 1044.1, 1081.9, 1106.7, 1143.9, 1205.2, 1220.7, 1273.4, 1276.0, 1303.7, 1353.8, 1374.1, 1389.4, 1432.4, 1484.2, 1491.7, 1509.0, 1515.1, 1525.5, 1720.2, 3008.1, 3044.9, 3075.8, 3108.3, 3116.0, 3126.0, 3191.4, 3220.0, 3412.5, 3654.0

B: 2.775341 1.362383 1.256967

C	0.695737	-1.259176	0.635878
N	1.394678	-1.051915	-0.537777
C	1.862088	0.302610	-0.794226
C	0.733795	1.284908	-0.501557
C	0.281267	1.172909	0.955484
C	0.140704	-0.272915	1.354755
O	-2.668831	0.452171	-0.282311
O	-2.209124	-0.723723	-0.621805
H	0.583013	-2.296264	0.927113
H	-1.394051	-0.845331	-0.079904
H	2.010585	-1.795675	-0.816490
H	2.175245	0.371288	-1.834614
H	2.724644	0.543351	-0.160616
H	1.063568	2.298490	-0.725499
H	-0.106116	1.060426	-1.161892
H	-0.671137	1.687122	1.083353
H	0.999170	1.672019	1.612550

H	-0.405578	-0.529611	2.251356

1,2,3,4-tetrahydropyridine

T_1= 0.01138359, D_1= 0.03555998

$\tilde{\nu}$: 185.3, 289.5, 455.0, 457.8, 517.1, 575.2, 733.4, 767.5, 862.1, 893.6, 926.1, 977.2, 981.9, 1045.1, 1081.4, 1105.0, 1142.2, 1205.6, 1219.5, 1273.3, 1300.6, 1352.0, 1374.9, 1388.1, 1430.6, 1484.7, 1490.7, 1506.2, 1515.5, 1739.5, 2995.1, 3039.9, 3069.1, 3082.8, 3109.2, 3121.3, 3198.6, 3223.0, 3635.7

B: 5.049839, 4.746692, 2.666731

C	0.642321	-1.188310	0.293367
N	1.409864	-0.042311	-0.167312
C	0.767064	1.187700	-0.074244
C	-0.552046	1.328330	0.065220
C	-1.476907	0.142533	0.127219
C	-0.751234	-1.127680	-0.320188
H	0.550439	-1.188386	1.387769
H	1.158681	-2.099814	-0.004575
H	2.372930	-0.032245	0.123993
H	1.415880	2.049416	-0.161712
H	-0.966370	2.325648	0.096979
H	-2.346249	0.313039	-0.510833
H	-1.867352	0.014431	1.141139
H	-1.316952	-2.016899	-0.043510
H	-0.645237	-1.124461	-1.406302

HO_2
T_1= 0.03589638, D_1= 0.12282697
$\tilde{\nu}$: 12528, 1459., 3686.8
B: 628.5431720, 34.6992321, 32.8838526

H	-0.880747	-0.865418	0.000000
O	0.055047	0.708193	0.000000
O	0.055047	-0.600015	0.000000

SP_{aa}^{12c}

T_1= 0.02316848, D_1= 0.11425218

$\tilde{\nu}$: -1086.6, 89.0, 124.1, 202.0, 252.9, 355.1, 451.8, 472.1, 492.0, 577.0, 701.0, 739.9, 801.8, 827.7, 866.1, 901.6, 928.7, 963.4, 1028.8, 1040.9, 1061.6, 1128.2, 1146.9, 1204.2, 1232.1, 1264.3, 1287.6, 1331.9, 1352.1, 1372.2, 1390.1, 1403.1, 1407.1, 1465.7, 1482.4, 1494.4, 1501.1, 1571.9, 1632.0, 3023.6, 3045.9, 3055.9, 3100.2, 3110.7, 3115.4, 3157.5, 3190.3, 3531.1

B: 3.1152196, 1.6952048, 1.4191715

C	0.427232	-1.444638	0.397806
N	1.268767	-1.001121	-0.698632
C	1.986447	0.219648	-0.344271
C	1.063056	1.403813	-0.059726
C	-0.130548	0.990962	0.803825
C	-0.402256	-0.358378	1.015587
O	-1.993655	-0.563056	-0.302886
O	-2.141825	0.622012	-0.699355
H	1.073961	-1.848955	1.186257
H	-0.208633	-2.264418	0.065064
H	0.699526	-0.844478	-1.523223
H	2.687345	0.462577	-1.141980
H	2.577399	-0.007813	0.546909
H	1.619727	2.196135	0.441596
H	0.719693	1.822366	-1.009563
H	-1.184465	1.126242	0.013872
H	-0.421318	1.673043	1.595858
H	-1.024351	-0.646938	1.854237

POST_{aa}^{12c}

T_1= 0.01138359, D_1= 0.03555998

$\tilde{\nu}$: 19.4, 52.3, 71.3, 109.5, 173.7, 194.9, 300.8, 407.2, 461.5, 487.5, 528.8, 671.5, 769.4, 818.4, 866.2, 902.4, 923.0, 987.0, 1011.2, 1033.7, 1053.1, 1119.7, 1148.2, 1208.7, 1225.7, 1267.2, 1278.6, 1330.4, 1355.0, 1370.6, 1398.6, 1422.5, 1472.0, 1476.0, 1496.7, 1508.4, 1517.6, 1721.9, 3027.2, 3036.5, 3056.2, 3087.7, 3092.6, 3118.0, 3158.7, 3180.4, 3442.8, 3543.0

B: 2.5863678, 1.4346169, 1.3597199

C	0.630546	-1.096678	1.040826
N	0.912879	-1.291111	-0.372823
C	1.708580	-0.202357	-0.924501
C	1.019412	1.158087	-0.808374
C	0.469750	1.345383	0.579910
C	0.313982	0.325932	1.423350
O	-2.231635	-0.678126	-0.682530
O	-2.476805	0.454604	-0.079494
H	1.490010	-1.432540	1.632190
H	-0.200870	-1.746157	1.319389
H	0.032373	-1.350862	-0.871071
H	1.943832	-0.428068	-1.963613
H	2.654115	-0.172043	-0.376866
H	1.718495	1.965393	-1.036002
H	0.212969	1.235584	-1.545467

H	-1.624621	0.713135	0.341075
H	0.227360	2.352510	0.902433
H	-0.029908	0.506793	2.436625

1,2,3,6-tetrahydropyridine

T_1= 0.01073238 , D_1= 0.03043207

$\tilde{\nu}$: 192.8, 308.2, 406.0, 476.5, 521.6, 664.0, 721.0, 824.1, 871.2, 916.5, 958.9, 998.4, 1024.1, 1034.7, 1073.6, 1098.3, 1155.0, 1206.4, 1222.5, 1252.6, 1299.0, 1349.8, 1368.5, 1416.8, 1427.7, 1474.5, 1487.1, 1496.9, 1511.5, 1749.6, 2949.5, 2966.3, 3055.9, 3077.8, 3092.1, 3111.0, 3176.3, 3199.3, 3574.7

B: 4.992453 4.787257 2.668145

C	-1.453141	0.103459	0.066847
N	-0.717387	-1.069280	-0.384052
C	0.559725	-1.205006	0.304793
C	1.475615	-0.069201	-0.129080
C	0.749292	1.242914	-0.045507
C	-0.569791	1.316130	0.067247
H	-1.874476	-0.023765	1.077699
H	-2.295977	0.269728	-0.606688
H	-1.278834	-1.900040	-0.261085
H	0.996276	-2.169398	0.048807
H	0.443000	-1.167984	1.398944
H	2.364597	-0.043265	0.504396
H	1.818844	-0.239909	-1.152604
H	1.337893	2.152366	-0.068706
H	-1.059822	2.277448	0.161796

SP_{aa}^{12d}

T_1= 0.02108323, D_1= 0.12112183

$\tilde{\nu}$: -1896.2, 190.8, 244.4, 269.1, 385.2, 399.3, 429.5, 523.1, 563.4, 639.2, 759.9, 790.0, 831.0, 861.4, 898.0, 919.1, 937.2, 956.8, 1022.0, 1052.0, 1067.6, 1077.0, 1136.5, 1158.8, 1176.8, 1210.3, 1248.3, 1276.4, 1333.3, 1339.7, 1349.4, 1368.7, 1381.9, 1399.2, 1465.8, 1475.0, 1487.4, 1519.2, 1575.5, 3003.3, 3024.7, 3038.0, 3104.3, 3118.8, 3120.0, 3125.3, 3152.4, 3577.5

B: 2.8815487, 2.1205892, 1.9617334

C	-0.213693	1.408553	0.535491
N	-1.249227	1.054848	-0.429476
C	-1.756767	-0.308180	-0.270379
C	-0.658226	-1.332410	-0.065073
C	0.197713	-1.006105	1.121526
C	0.855167	0.346453	0.812037
O	1.772255	0.121797	-0.253779
O	1.088346	-0.232743	-1.413146
H	-0.692414	1.621013	1.497469
H	0.268516	2.326688	0.202029
H	-0.852298	1.137432	-1.355348
H	-2.370531	-0.547358	-1.137089
H	-2.418918	-0.327456	0.605473
H	-0.912310	-2.374915	-0.233223
H	0.247400	-1.004141	-1.002671
H	0.962141	-1.757297	1.311061
H	-0.415276	-0.896231	2.023947
H	1.498312	0.676034	1.628480

$POST_{aa}^{12d}$

T_1= 0.01301918, D_1= 0.04346640

$\tilde{\nu}$: 108.7, 128.4, 185.6, 244.1, 269.9, 320.8, 381.2, 410.0, 437.3, 507.1, 616.2, 771.5, 799.3, 853.4, 886.6, 900.1, 927.9, 952.1, 1006.5, 1044.0, 1086.4, 1095.1, 1145.0, 1158.0, 1208.0, 1261.5, 1288.5, 1341.5, 1349.1, 1369.6, 1384.5, 1390.7, 1399.8, 1408.8, 1461.0, 1467.9, 1477.9, 1508.4, 2963.6, 2964.8, 3039.0, 3081.4, 3090.5, 3110.6, 3117.2, 3199.3, 3566.8, 3792.7

B: 2.9439122, 1.9136233, 1.7415594

C	0.147549	-1.164319	0.862283
N	1.074538	-1.257051	-0.257116
C	1.888469	-0.047999	-0.393762
C	1.082784	1.209241	-0.409710
C	0.061300	1.362509	0.664430
C	-0.743845	0.079225	0.899999
O	-1.881238	-0.010313	0.044221
O	-1.469577	-0.209477	-1.302267
H	0.736210	-1.170408	1.785716
H	-0.486507	-2.050569	0.872689
H	0.534530	-1.376865	-1.104427
H	2.502154	-0.137340	-1.289104
H	2.584109	-0.031552	0.459989
H	1.441572	2.080586	-0.939831
H	-1.356410	0.694357	-1.625615
H	-0.623663	2.190550	0.474718

H	0.567165	1.595085	1.614653
H	-1.231954	0.131897	1.875961

SP$_{aa-ea}$

T_1= 0.02374455, D_1= 0.14186528

$\tilde{\nu}$: -578.2, 87.6, 154.9, 195.7, 296.1, 328.3, 390.2, 436.5, 511.8, 570.8, 705.4, 839.0, 861.3, 863.7, 902.0, 928.8, 985.3, 1040.5, 1061.4, 1096.0, 1155.0, 1172.1, 1224.6, 1236.5, 1298.8, 1300.3, 1341.1, 1351.9, 1358.5, 1373.8, 1387.9, 1396.0, 1416.7, 1468.0, 1479.9, 1485.1, 1489.3, 1500.2, 2957.3, 2959.7, 3059.6, 3071.2, 3100.4, 3110.4, 3118.1, 3122.2, 3137.1, 3745.8

B: 3.4228237, 1.7178634, 1.4275292

C	0.148900	1.091795	0.782887
N	-0.846064	1.353553	-0.209090
C	-1.950728	0.440570	-0.312755
C	-1.448871	-0.988348	-0.515418
C	-0.443082	-1.352273	0.577994
C	0.679717	-0.333041	0.676809
O	1.466701	-0.483946	-0.538073
O	2.540781	0.240104	-0.510904
H	-0.250577	1.198625	1.804247
H	0.978331	1.788059	0.678578
H	-0.763243	2.139402	-0.818896
H	-2.588755	0.744847	-1.140508
H	-2.566532	0.471463	0.600186
H	-2.282481	-1.691483	-0.498378
H	-0.967901	-1.060469	-1.491875
H	-0.021349	-2.343724	0.412064
H	-0.946093	-1.372332	1.548625
H	1.355582	-0.550736	1.504292

PIP-C^{3}OȮ$_{ea}$

T_1= 0.02366231, D_1= 0.14149920

$\tilde{\nu}$: 88.8, 153.1, 203.0, 307.5, 335.6, 407.7, 426.5, 500.5, 562.5, 664.9, 762.2, 842.7, 867.2, 879.7, 919.4, 938.6, 996.8, 1047.1, 1073.2, 1103.0, 1154.7, 1168.4, 1200.6, 1227.5, 1289.5, 1299.4, 1315.1, 1347.2, 1360.4, 1374.7, 1389.1, 1394.4, 1426.1, 1478.8, 1487.1, 1491.0, 1494.3, 1512.1, 2947.3, 2957.8, 3063.5, 3077.4, 3103.7, 3111.8, 3114.8, 3126.0, 3126.1, 3588.0

B: 3.4608223, 1.7165706, 1.4182485

C	1.953319	0.420888	-0.284800
N	0.818202	1.331672	-0.323346
C	-0.123182	1.090867	0.753771
C	-0.675562	-0.321101	0.665247
C	0.422304	-1.365093	0.565741
C	1.451639	-0.999913	-0.502335
O	-1.487085	-0.468560	-0.532887
O	-2.558668	0.256938	-0.479718
H	2.490689	0.470419	0.677131
H	2.653250	0.701449	-1.070581
H	1.117811	2.295763	-0.324778
H	-0.949159	1.796885	0.685476
H	0.332074	1.188814	1.752939
H	-1.342778	-0.514546	1.505213
H	-0.021509	-2.341934	0.373561
H	0.909171	-1.415587	1.542975
H	2.283493	-1.703620	-0.472827
H	0.994463	-1.060265	-1.490586

SP$^{12b}_{ea}$

T_1= 0.02235339, D_1= 0.12278353

$\tilde{\nu}$: -1180.5, 93.2, 137.6, 207.9, 256.8, 348.5, 438.3, 454.4, 480.6, 582.1, 624.6, 731.9, 762.6, 838.0, 868.1, 904.5, 936.8, 970.4, 1005.4, 1056.0, 1077.2, 1095.3, 1143.5, 1200.0, 1216.7, 1253.3, 1289.4, 1298.4, 1351.1, 1378.7, 1386.0, 1406.6, 1414.6, 1465.3, 1478.4, 1491.3, 1511.0, 1596.2, 1617.3, 2964.4, 3037.2, 3075.3, 3101.9, 3110.1, 3126.0, 3141.9, 3206.6, 3618.3

B: 3.1415290, 1.7012186, 1.4083867

C	-1.967060	-0.391498	-0.303115
N	-0.907208	-1.378829	-0.162584
C	0.153050	-1.007165	0.743254
C	0.401078	0.342488	0.995660
C	-0.463434	1.440774	0.462558
C	-1.345809	0.946772	-0.679907
O	1.946631	0.628798	-0.340813
O	2.213615	-0.549861	-0.679166
H	-2.544246	-0.263915	0.625855
H	-2.651266	-0.730383	-1.079752
H	-1.264398	-2.296375	0.049701
H	1.260254	-1.115883	0.045035
H	0.361866	-1.706375	1.548002
H	1.045773	0.578488	1.831423
H	0.151538	2.288174	0.158517
H	-1.092200	1.789620	1.288998

H	-2.121973	1.678544	-0.900733
H	-0.743808	0.810183	-1.579827

$POST_{ea}^{12b}$

T_1= 0.01562444, D_1= 0.07106334

$\tilde{\nu}$: 86.6, 156.1, 180.0, 280.2, 294.2, 324.9, 406.8, 433.4, 484.9, 542.9, 614.1, 625.8, 740.7, 849.2, 851.9, 905.4, 930.0, 949.2, 1040.6, 1052.5, 1079.0, 1085.2, 1136.8, 1198.4, 1219.0, 1263.0, 1303.9, 1334.3, 1364.4, 1379.7, 1391.3, 1395.4, 1403.9, 1468.6, 1481.8, 1488.1, 1507.8, 1527.2, 2992.0, 3069.9, 3078.9, 3088.7, 3112.3, 3121.5, 3128.8, 3227.1, 3620.4, 3806.1

B: 3.5252857, 1.6531017, 1.3509096

C	2.033882	0.401383	-0.288962
N	0.967018	1.382995	-0.130860
C	-0.092122	1.079711	0.704059
C	-0.634452	-0.302043	0.692856
C	0.470059	-1.345960	0.575273
C	1.441010	-0.975388	-0.542558
O	-1.476160	-0.576077	-0.454758
O	-2.599478	0.285202	-0.400193
H	2.654085	0.364368	0.616811
H	2.666155	0.711133	-1.119640
H	1.289017	2.335519	-0.069716
H	-2.304377	1.036609	-0.929898
H	-0.714032	1.906211	1.011994
H	-1.245199	-0.464520	1.582126
H	0.020458	-2.321837	0.395563
H	0.999083	-1.389106	1.529400
H	2.246294	-1.706797	-0.607571
H	0.914222	-0.971763	-1.497443

SP_{ea}^{12c}

T_1= 0.02312346, D_1= 0.11250351

$\tilde{\nu}$: -1085.2, 100.5, 131.3, 214.8, 259.3, 378.0, 450.1, 473.3, 481.7, 579.9, 686.4, 729.9, 797.5, 831.0, 880.7, 920.9, 956.6, 960.0, 1046.9, 1061.0, 1079.5, 1107.9, 1162.9, 1203.2, 1226.9, 1262.4, 1284.4, 1304.5, 1352.2, 1364.3, 1400.1, 1405.2, 1430.4, 1479.2, 1485.2, 1491.4, 1510.8, 1578.7, 1647.8, 2935.5, 2958.6, 3066.1, 3101.9, 3109.2, 3116.7, 3158.0, 3203.2, 3582.5

B: 3.1715158, 1.6870563, 1.4062368

C	-1.994273	0.237637	-0.307028
N	-1.201764	-0.916244	-0.714945
C	-0.423917	-1.428051	0.396837
C	0.415931	-0.342939	0.989787
C	0.150561	1.005044	0.762149
C	-1.060163	1.417320	-0.075366
O	2.017343	-0.578376	-0.279788
O	2.163914	0.588650	-0.721132
H	-2.558060	0.033026	0.616867
H	-2.712323	0.469886	-1.091764
H	-1.786679	-1.644521	-1.098580
H	0.209281	-2.249421	0.061460
H	-1.059586	-1.813211	1.214225
H	1.024087	-0.631056	1.837163
H	1.196840	1.114290	-0.055124
H	0.461015	1.693566	1.540981
H	-1.598399	2.217740	0.432406
H	-0.742703	1.807149	-1.043945

$POST_{ea}^{12c}$

T_1= 0.02265164, D_1= 0.13443531

$\tilde{\nu}$: 28.4, 66.5, 75.3, 147.7, 202.5, 262.8, 378.2, 417.7, 503.3, 521.7, 671.0, 755.6, 850.4, 868.6, 916.8, 951.3, 980.7, 1011.7, 1030.9, 1042.5, 1075.4, 1100.9, 1147.2, 1201.4, 1228.3, 1258.5, 1288.0, 1307.4, 1349.8, 1371.6, 1415.8, 1426.6, 1476.3, 1482.4, 1499.7, 1514.2, 1655.0, 1750.1, 2778.1, 3000.8, 3014.6, 3051.6, 3097.2, 3098.3, 3122.0, 3184.2, 3207.5, 3556.2

B: 3.0074254, 1.2661681, 1.1972833

C	1.084368	1.188904	0.717317
N	-0.148697	0.411999	0.871239
C	0.117024	-1.028001	0.949825
C	1.026724	-1.449436	-0.165179
C	1.708373	-0.585209	-0.904540
C	1.677227	0.895616	-0.652751
O	-2.943472	-0.123869	-0.075329
O	-2.094712	0.399455	-0.921816
H	1.811736	0.924093	1.494448
H	0.846817	2.246043	0.821489
H	-0.663547	0.707004	1.691506
H	-0.837512	-1.550284	0.869290
H	0.562465	-1.303977	1.914895
H	1.097499	-2.512208	-0.358078
H	-1.251453	0.514541	-0.357062
H	2.320557	-0.944803	-1.722320

H	2.686544	1.305752	-0.713533
H	1.090943	1.393920	-1.430182

SP_{ea}^{12d}

T_1= 0.01798529, D_1= 0.09334412

$\tilde{\nu}$: -2051.8, 147.5, 218.6, 278.1, 343.8, 422.7, 432.1, 487.8, 552.6, 597.8, 699.3, 775.9, 836.2, 864.8, 892.4, 916.9, 948.2, 979.6, 1026.9, 1060.2, 1081.1, 1092.8, 1135.0, 1155.8, 1198.6, 1214.1, 1243.9, 1285.0, 1299.1, 1338.1, 1359.5, 1365.4, 1380.8, 1420.2, 1471.3, 1489.7, 1496.3, 1503.5, 1573.1, 2899.2, 2962.3, 3047.3, 3085.6, 3108.1, 3112.8, 3139.8, 3153.6, 3597.0

B: 2.9121251, 2.1580871, 1.8226897

C	-1.582315	0.815039	-0.306693
N	-1.372148	-0.559957	-0.726484
C	-0.722351	-1.404735	0.260064
C	0.612584	-0.786166	0.671572
C	0.376865	0.596425	1.264483
C	-0.289367	1.411844	0.185526
O	1.425665	-0.715332	-0.516181
O	1.708599	0.590048	-0.904010
H	-2.314528	0.902263	0.519190
H	-1.975752	1.382597	-1.148957
H	-2.224816	-0.978591	-1.066358
H	-0.543456	-2.381715	-0.187434
H	-1.316536	-1.551938	1.176166
H	1.128316	-1.439757	1.376606
H	1.311271	1.049819	1.586008
H	-0.271236	0.485102	2.139516
H	-0.292903	2.488918	0.325217
H	0.658063	1.170834	-0.722742

$POST_{ea}^{12d}$

T_1= 0.01287189, D_1= 0.04138999

$\tilde{\nu}$: 144.1, 150.7, 163.6, 276.1, 316.2, 334.0, 419.3, 424.6, 506.6, 579.1, 632.7, 771.0, 797.9, 868.8, 882.7, 934.0, 943.4, 966.7, 993.9, 1055.1, 1091.9, 1102.7, 1133.5, 1170.6, 1195.6, 1258.7, 1269.3, 1312.3, 1345.6, 1367.5, 1387.0, 1401.6, 1419.4, 1453.1, 1479.6, 1487.5, 1498.0, 1510.5, 2891.3, 2968.6, 2975.0, 3088.9, 3100.6, 3108.6, 3118.5, 3223.5, 3561.6, 3617.1

B: 2.9121190, 1.9872081, 1.8214972

C	0.045163	-1.268994	-0.013080
N	1.499055	-1.222474	-0.052139
C	1.840172	0.040403	0.396762
N	0.953547	-1.130630	0.402156
C	0.180991	-1.197912	-0.837611
C	-0.801570	-0.033575	-0.885877
C	-0.075705	1.309211	-0.767272
C	1.043489	1.285253	0.216050
O	-1.836056	-0.203254	0.073204
O	-1.371888	0.135535	1.375899
H	2.587379	-0.053642	-0.414846
H	2.392053	0.072608	1.335788
H	1.497619	-1.974933	0.520090
H	-0.377576	-2.132400	-0.861546
H	0.827195	-1.153977	-1.728403
H	-1.350573	-0.073607	-1.828352
H	-0.800636	2.083334	-0.516059
H	0.309336	1.551111	-1.769091
H	1.417489	2.211955	0.623122
H	-0.637824	-0.494576	1.499075

PIPC[3]OȮ$_{ee}$

T_1= 0.02371353, D_1= 0.14214839

$\tilde{\nu}$: 124.8, 136.9, 241.2, 314.8, 324.4, 371.6, 433.0, 451.1, 530.3, 672.3, 761.6, 825.2, 885.1, 894.5, 957.4, 970.7, 995.0, 1041.6, 1073.6, 1130.4, 1158.9, 1180.8, 1190.6, 1231.5, 1250.9, 1297.4, 1309.5, 1334.6, 1347.7, 1375.6, 1378.4, 1407.4, 1428.3, 1481.2, 1487.9, 1499.4, 1505.7, 1518.9, 2966.1, 2999.5, 3067.6, 3072.4, 3105.0, 3112.7, 3114.5, 3117.9, 3128.1, 3577.7

B: 3.9042221, 1.5412312, 1.2747997

C	-2.050484	-0.063992	0.453183
N	-1.428371	-1.194930	-0.226315
C	0.007361	-1.243040	0.004578
C	0.617015	0.010948	-0.603649
C	0.027204	1.267422	0.007722
C	-1.495611	1.231739	-0.122743
O	2.060250	-0.019032	-0.461718
O	2.438534	0.016097	0.780053
H	-1.861139	-0.083146	1.537840
H	-3.127625	-0.116554	0.300522
H	-1.858013	-2.065195	0.053867
H	0.426456	-2.126099	-0.476658
H	0.273503	-1.266978	1.070466
H	0.476915	-0.001571	-1.684519

H	0.446444	2.149622	-0.476346
H	0.310960	1.296127	1.061491
H	-1.931978	2.087894	0.391322
H	-1.780109	1.295422	-1.175013

SP_{ee}^{12b}

T_1= 0.03053936, D_1= 0.18381295

$\tilde{\nu}$: -1720.7, 127.9, 220.9, 301.5, 352.3, 389.0, 433.8, 483.8, 492.9, 583.8, 649.9, 716.6, 821.8, 883.3, 889.7, 947.7, 964.1, 988.9, 1008.0, 1074.1, 1086.0, 1111.3, 1158.7, 1187.0, 1226.0, 1244.5, 1289.7, 1298.3, 1312.1, 1343.0, 1370.4, 1384.6, 1401.1, 1444.3, 1486.5, 1495.1, 1508.2, 1517.7, 1797.9, 3004.8, 3061.4, 3075.6, 3080.5, 3118.0, 3121.3, 3126.0, 3131.3, 3630.3

B: 3.6119266. 1.7143441. 1.4104466

C	-1.924911	-0.306694	0.502615
N	-1.155267	-1.291079	-0.240966
C	0.211792	-1.108824	-0.380149
C	0.679477	0.317592	-0.706059
C	-0.126407	1.363041	0.054395
C	-1.623092	1.081606	-0.041857
O	2.038831	0.273618	-0.312066
O	2.014630	-0.287047	0.967664
H	-1.672770	-0.340430	1.570430
H	-2.980547	-0.549717	0.396019
H	-1.471929	-2.244562	-0.175888
H	0.674258	-1.866884	-1.009690
H	0.970757	-1.087802	0.658289
H	0.677864	0.517741	-1.779861
H	0.115527	2.352951	-0.330520
H	0.187586	1.325217	1.098502
H	-2.185790	1.824846	0.522090
H	-1.956934	1.133304	-1.081065

$POST_{ee}^{12b}$

T_1= 0.01569201, D_1= 0.06974811

$\tilde{\nu}$: 96.0, 157.8, 238.6, 253.9, 307.0, 344.6, 369.9, 457.4, 468.2, 495.6, 649.9, 689.2, 729.2, 826.1, 894.9, 912.4, 963.2, 976.5, 1002.9, 1079.9, 1100.7, 1114.6, 1153.4, 1186.0, 1232.0, 1257.7, 1294.5, 1320.8, 1333.8, 1374.5, 1382.2, 1396.2, 1423.4, 1464.3, 1486.8, 1497.3, 1509.2, 1524.5, 2988.2, 2998.0, 3060.7, 3083.7, 3114.6, 3117.2, 3132.8, 3183.7, 3625.6, 3759.9

B: 3.7115237, 1.5467951, 1.3044524

C	2.030692	0.129285	0.535685
N	1.365961	1.259521	-0.096540
C	-0.001656	1.214963	-0.282277
C	-0.616516	-0.089354	-0.683484
C	0.049340	-1.282936	-0.013043
C	1.566769	-1.163090	-0.117723
O	-2.027919	-0.055786	-0.481795
O	-2.308252	0.013970	0.908624
H	1.798946	0.091732	1.608227
H	3.105881	0.259974	0.423983
H	1.733583	2.163343	0.152807
H	-0.447723	2.119619	-0.676482
H	-2.038801	0.917939	1.127295
H	-0.563665	-0.217017	-1.774411
H	-0.312475	-2.201681	-0.473139
H	-0.246479	-1.296689	1.036370
H	2.050458	-2.010385	0.367430
H	1.876150	-1.162158	-1.165882

SP_{ee}^{12b2}

T_1= 0.02884927, D_1= 0.16190884

$\tilde{\nu}$: -390.2, 102.1, 114.7, 150.6, 221.9, 258.7, 315.0, 335.4, 389.4, 446.5, 494.2, 544.3, 740.4, 822.2, 856.9, 871.4, 917.6, 940.8, 971.6, 1026.7, 1034.1, 1080.2, 1106.6, 1145.8, 1207.3, 1226.8, 1275.6, 1315.5, 1351.9, 1370.9, 1382.8, 1394.2, 1425.6, 1478.2, 1487.0, 1504.3, 1537.9, 1623.8, 3063.8, 3071.1, 3077.4, 3115.2, 3118.9, 3127.0, 3214.8, 3234.9, 3682.5, 3801.9

B: 2.876368 1.718216 1.594262

C	1.503818	-0.574692	-0.769679
N	0.873221	-1.329093	0.308603
C	0.058150	-0.717938	1.207791
C	-0.395942	0.563656	1.029566
C	0.305016	1.462988	0.043456
C	1.662194	0.882202	-0.354583
O	-2.114794	0.212008	0.054476
O	-1.752083	-0.368961	-1.151364
H	0.884564	-0.630728	-1.666651
H	2.468893	-1.029585	-0.988070
H	1.216021	-2.248165	0.518846
H	-0.302396	-1.338296	2.015932
H	-1.850007	-1.314199	-0.985042
H	-0.958904	1.006091	1.836729

H	0.434669	2.453498	0.479166
H	-0.319149	1.579132	-0.843490
H	2.095227	1.445649	-1.180061
H	2.354132	0.938577	0.488222

POST$_{ee}^{12b2}$

T_1= 0.02259519, D_1= 0.13292953

$\tilde{\nu}$: 30.3, 51.5, 78.1, 138.3, 179.3, 284.9, 312.3, 449.1, 505.4, 522.9, 716.4, 744.8, 786.4, 858.5, 875.4, 889.0, 932.7, 962.9, 999.6, 1030.7, 1077.0, 1106.3, 1126.4, 1198.4, 1245.4, 1271.1, 1286.3, 1312.9, 1353.3, 1376.2, 1398.4, 1431.9, 1461.9, 1481.3, 1493.9, 1503.9, 1618.2, 1746.1, 2978.3, 3047.3, 3054.6, 3066.8, 3095.8, 3120.8, 3130.0, 3197.8, 3220.0, 3534.5

B: 4.335617 1.002158 0.880717

C	-0.692461	-1.309942	0.144108
N	0.316871	-0.332433	-0.276146
C	-0.099636	1.021401	-0.247250
C	-1.323420	1.411428	0.091830
C	-2.451469	0.441802	0.305582
C	-2.097350	-0.915958	-0.294522
O	3.210740	0.183664	-0.471270
O	2.797724	-0.167220	0.718198
H	-0.663673	-1.368435	1.235051
H	-0.403818	-2.284505	-0.244341
H	0.756271	-0.562393	-1.160803
H	0.690261	1.726092	-0.474578
H	1.809126	-0.315580	0.578743
H	-1.519088	2.471169	0.178741
H	-3.359457	0.834144	-0.154435
H	-2.671033	0.335806	1.371864
H	-2.810872	-1.679518	0.013379
H	-2.127512	-0.853696	-1.384516

SP$_{ee}^{12c}$

T_1= 0.02116004, D_1= 0.11784983

$\tilde{\nu}$: -2193.4, 131.5, 194.5, 288.9, 371.6, 388.7, 436.3, 485.0, 543.8, 580.6, 722.0, 767.3, 817.0, 875.9, 899.5, 954.5, 970.3, 985.8, 1017.5, 1061.3, 1078.0, 1130.3, 1152.0, 1183.2, 1190.7, 1204.5, 1244.0, 1290.2, 1309.4, 1335.4, 1355.5, 1376.8, 1393.9, 1430.8, 1456.3, 1483.1, 1504.5, 1515.0, 1755.3, 2972.9, 3016.6, 3024.9, 3085.8, 3091.9, 3116.5, 3122.5, 3125.4, 3577.1

B: 3.5777203. 1.7256319. 1.4191330

C	-1.902301	0.182139	0.536469
N	-1.546371	-1.057724	-0.140987
C	-0.131132	-1.358520	0.033096
C	0.692194	-0.315762	-0.715312
C	0.217414	1.106644	-0.422413
C	-1.245598	1.344257	-0.196547
O	2.045693	-0.284496	-0.274357
O	1.971004	0.352269	0.970029
H	-1.574795	0.181849	1.587100
H	-2.984825	0.298344	0.522185
H	-2.111350	-1.824319	0.195725
H	0.085736	-2.345513	-0.371699
H	0.176356	-1.340786	1.086174
H	0.719330	-0.539206	-1.783476
H	0.723411	1.855271	-1.029825
H	1.004491	1.089413	0.644723
H	-1.402696	2.284841	0.334280
H	-1.728103	1.449447	-1.175426

POST$_{ee}^{12c}$

T_1= 0.01267132, D_1= 0.03740506

$\tilde{\nu}$: 89.3, 146.3, 195.7, 225.3, 293.8, 365.9, 373.9, 425.3, 461.5, 485.5, 637.8, 667.0, 784.2, 813.8, 902.5, 960.2, 968.9, 997.9, 1019.5, 1076.0, 1082.1, 1120.2, 1149.8, 1190.2, 1198.3, 1227.1, 1293.7, 1310.5, 1329.9, 1341.1, 1372.0, 1398.3, 1407.1, 1424.4, 1465.0, 1480.1, 1504.5, 1515.8, 2973.6, 2993.9, 3003.2, 3021.1, 3092.1, 3112.4, 3122.4, 3178.1, 3574.4, 3815.1

B: 3.7322574, 1.5343247, 1.2957055

C	-2.017050	0.003102	0.553658
N	-1.500492	-1.130652	-0.201566
C	-0.058896	-1.265495	-0.032585
C	0.614856	-0.059940	-0.682538
C	-0.005701	1.223144	-0.252442
C	-1.481590	1.292813	-0.063005
O	2.026268	-0.070541	-0.492418
O	2.308292	0.036266	0.897478
H	-1.716322	-0.039914	1.611143
H	-3.105208	-0.008083	0.510087
H	-1.964867	-1.983934	0.075648
H	0.285833	-2.174574	-0.522518
H	0.241189	-1.300226	1.021785
H	0.535881	-0.171259	-1.772734

H	0.559087	2.132018	-0.420576
H	2.406803	0.989376	1.012929
H	-1.751070	2.153040	0.551493
H	-1.974079	1.430570	-1.035306

SP_{ee-ae}

T_1= 0.02415020, D_1= 0.14511910

$\tilde{\nu}$: -579.8, 122.2, 138.6, 231.8, 308.4, 323.5, 356.7, 425.0, 461.3, 555.7, 673.9, 823.4, 872.5, 886.1, 951.0, 963.3, 991.7, 1028.5, 1056.1, 1131.4, 1153.7, 1184.0, 1216.9, 1239.7, 1252.6, 1303.5, 1330.5, 1348.2, 1353.6, 1375.5, 1377.1, 1406.0, 1418.7, 1462.7, 1487.9, 1493.2, 1494.2, 1508.2, 2976.3, 3001.5, 3057.8, 3069.1, 3098.2, 3107.9, 3120.5, 3122.6, 3127.3, 3744.5

B: 3.9103270, 1.5324749, 1.2673419

C	-2.075145	-0.050648	0.448612
N	-1.409195	-1.209802	-0.081719
C	0.018414	-1.264311	0.020589
C	0.621032	-0.005492	-0.603358
C	0.021195	1.249966	0.004820
C	-1.499937	1.230964	-0.154096
O	2.063493	-0.024970	-0.467461
O	2.447825	0.057236	0.771136
H	-1.954605	-0.006086	1.541180
H	-3.141428	-0.128868	0.243900
H	-1.916191	-1.927142	-0.556041
H	0.397307	-2.151156	-0.484532
H	0.352253	-1.302197	1.066372
H	0.476974	-0.021720	-1.684346
H	0.457847	2.131651	-0.465134
H	0.283519	1.273033	1.064568
H	-1.933315	2.106275	0.331302
H	-1.761903	1.273823	-1.214045

PIPC[3]OȮ$_{ae}$

T_1= 0.02363597, D_1= 0.14156964

$\tilde{\nu}$: 88.3, 143.1, 240.1, 255.2, 339.3, 384.1, 451.5, 468.2, 531.3, 601.9, 754.0, 823.2, 879.8, 891.6, 937.0, 968.3, 982.8, 1054.9, 1061.3, 1125.1, 1151.0, 1184.6, 1190.8, 1239.6, 1288.7, 1300.6, 1323.2, 1334.7, 1343.0, 1377.1, 1385.0, 1392.0, 1405.9, 1481.2, 1490.1, 1491.2, 1498.0, 1509.0, 3044.1, 3050.8, 3054.2, 3056.2, 3079.2, 3106.4, 3116.9, 3122.3, 3124.9, 3544.9

B: 4.2175274, 1.4306304, 1.1469785

C	-0.262306	-1.331393	-0.174827
N	-1.645798	-1.135928	0.230314
C	-2.182483	0.146245	-0.215069
C	-1.329711	1.343988	0.202125
C	0.113095	1.151437	-0.260808
C	0.634571	-0.176814	0.253263
O	1.946913	-0.446149	-0.296395
O	2.854308	0.327794	0.215981
H	-0.229005	-1.408555	-1.265487
H	0.107729	-2.271603	0.231846
H	-1.730486	-1.219580	1.236262
H	-3.199908	0.242359	0.161977
H	-2.245349	0.112113	-1.306874
H	-1.738604	2.265572	-0.213740
H	-1.356827	1.442850	1.291236
H	0.761792	1.956411	0.082254
H	0.152538	1.132693	-1.353939
H	0.749947	-0.154697	1.339470

SP_{ae}^{12b}

T_1= 0.03053936, D_1= 0.18381297

$\tilde{\nu}$: -1720.7, 127.9, 220.9, 301.5, 352.3, 389.0, 433.8, 483.8, 492.9, 583.8, 649.9, 716.6, 821.8, 883.3, 889.7, 947.7, 964.1, 988.9, 1008.0, 1074.1, 1086.0, 1111.3, 1158.7, 1187.0, 1226.0, 1244.5, 1289.7, 1298.3, 1312.1, 1343.0, 1370.4, 1384.6, 1401.1, 1444.3, 1486.5, 1495.1, 1508.2, 1517.7, 1797.9, 3004.8, 3061.4, 3075.6, 3080.5, 3118.0, 3121.3, 3126.0, 3131.3, 3630.3

B: 3.6119266, 1.7143441, 1.4104466

C	-0.211792	-1.108824	-0.380149
N	1.155267	-1.291079	-0.240966
C	1.924911	-0.306694	0.502615
C	1.623092	1.081606	-0.041857
C	0.126407	1.363041	0.054395
C	-0.679477	0.317592	-0.706059
O	-2.038831	0.273618	-0.312066
O	-2.014630	-0.287047	0.967664
H	-0.970757	-1.087802	0.658289
H	-0.674257	-1.866884	-1.009690
H	1.471930	-2.244562	-0.175888
H	2.980547	-0.549717	0.396019
H	1.672770	-0.340430	1.570430
H	2.185790	1.824846	0.522090

H	1.956934	1.133304	-1.081065
H	-0.115527	2.352951	-0.330520
H	-0.187586	1.325217	1.098502
H	-0.677864	0.517741	-1.779861

POST$_{ae}^{12b}$

T_1= 0.01569201, D_1= 0.06974813

$\tilde{\nu}$: 96.0, 157.8, 238.6, 253.9, 307.0, 344.6, 369.9, 457.4, 468.2, 495.6, 649.9, 689.2, 729.2, 826.1, 894.9, 912.4, 963.2, 976.5, 1002.9, 1079.9, 1100.7, 1114.6, 1153.4, 1186.0, 1232.0, 1257.7, 1294.5, 1320.8, 1333.8, 1374.5, 1382.2, 1396.2, 1423.4, 1464.3, 1486.8, 1497.3, 1509.2, 1524.5, 2988.2, 2998.0, 3060.7, 3083.7, 3114.6, 3117.2, 3132.8, 3183.7, 3625.6, 3759.9

B: 3.7115236, 1.5467952, 1.3044525

C	-0.001656	-1.214963	-0.282277
N	1.365961	-1.259521	-0.096540
C	2.030691	-0.129285	0.535685
C	1.566769	1.163090	-0.117723
C	0.049340	1.282936	-0.013043
C	-0.616516	0.089354	-0.683485
O	-2.027919	0.055786	-0.481795
O	-2.308252	-0.013970	0.908624
H	-2.038799	-0.917938	1.127295
H	-0.447723	-2.119619	-0.676482
H	1.733583	-2.163343	0.152807
H	3.105881	-0.259974	0.423983
H	1.798946	-0.091732	1.608227
H	2.050458	2.010385	0.367430
H	1.876150	1.162158	-1.165883
H	-0.312475	2.201681	-0.473139
H	-0.246479	1.296689	1.036370
H	-0.563665	0.217017	-1.774411

SP$_{ae}^{12c}$

T_1= 0.02131292, D_1= 0.11865378

$\tilde{\nu}$: -2214.6, 122.3, 192.4, 282.4, 349.9, 382.2, 441.0, 493.5, 539.4, 584.9, 719.6, 754.4, 803.6, 858.6, 895.8, 948.2, 953.6, 978.5, 997.6, 1042.0, 1066.5, 1122.1, 1152.3, 1176.4, 1191.1, 1199.4, 1257.2, 1288.6, 1327.6, 1344.0, 1354.3, 1381.3, 1396.2, 1407.8, 1458.6, 1486.7, 1490.8, 1498.7, 1754.8, 2999.6, 3058.5, 3063.8, 3082.3, 3083.4, 3117.4, 3121.0, 3133.1, 3538.3

B: 3.5463462, 1.7198999, 1.4186311

C	0.116974	-1.368261	0.045557
N	1.554733	-1.138377	0.022322
C	1.901689	0.169371	0.559126
C	1.261747	1.326052	-0.210055
C	-0.204073	1.103608	-0.431316
C	-0.692157	-0.315857	-0.716051
O	-2.049380	-0.262719	-0.280584
O	-1.964748	0.372033	0.963328
H	-0.210344	-1.351503	1.086015
H	-0.100109	-2.358286	-0.352391
H	1.926206	-1.249510	-0.913907
H	2.985906	0.274146	0.565894
H	1.566643	0.192349	1.599273
H	1.433899	2.281587	0.290009
H	1.748107	1.406042	-1.191926
H	-0.707896	1.854900	-1.037653
H	-0.990194	1.097127	0.638160
H	-0.727407	-0.542198	-1.785250

POST$_{ae}^{12c}$

T_1= 0.01313519, D_1= 0.04350301

$\tilde{\nu}$: 88.4, 145.1, 190.4, 226.7, 287.3, 349.6, 361.3, 413.9, 459.9, 492.9, 635.2, 655.8, 768.6, 810.0, 899.3, 937.0, 965.6, 985.0, 998.4, 1052.9, 1097.8, 1112.4, 1130.9, 1178.9, 1194.5, 1247.4, 1303.5, 1316.0, 1332.3, 1338.9, 1365.4, 1391.2, 1397.4, 1417.9, 1468.1, 1488.3, 1493.0, 1500.3, 2964.6, 2976.6, 3061.2, 3085.1, 3089.0, 3115.4, 3135.1, 3180.3, 3534.3, 3818.4

B: 3.7281553, 1.5245245, 1.2884927

C	0.045163	-1.268994	-0.013080
N	1.499055	-1.222474	-0.052139
C	2.026685	-0.015640	0.566770
C	1.501293	1.274907	-0.077427
C	0.021292	1.222508	-0.228317
C	-0.608242	-0.048876	-0.674673
O	-2.023807	-0.044124	-0.511830
O	-2.324595	0.037211	0.875810
H	-0.268847	-1.299350	1.030478
H	-0.308164	-2.179111	-0.495722
H	1.833861	-1.305290	-1.005103
H	3.115057	-0.044445	0.525322
H	1.737101	-0.035785	1.619996
H	1.803982	2.150406	0.499511

H	1.976713	1.377579	-1.065071
H	-0.551585	2.135508	-0.331976
H	-2.444697	0.986174	1.002659
H	-0.516737	-0.156499	-1.766599

Table S13. QCC results the PIP-C³Ȯ conformational pathways and reactions

Energies (/Hartree) and energies relative to the CHOCH$_2$CH$_2$CH$_2$NHĊH$_2$ radical (/kJ mol^{-1}) of stationary points on the potential energy surface relevant to the internal H-transfer reactions and ring scission in the 3-piperidinyl oxy radical, PIPC³Ȯ.

Species	M06-2X/aTZ		CCSD(T*)-F12a/aTZ	
	E_{Elec}	E_{ZPE}	E_{Elec}	$\Delta E_{v=0}$
PIPC³OȮ$_{ea}$ (reactant)	-401.59654	0.15653	-401.14775	
NO (reactant)	-129.89335	0.00471	-129.76980	
NO_2 (product)	-205.07464	0.00917	-204.88891	
Sum reactants – NO_2	-326.41526	0.15207	-326.02864	67.4
PIPC³OȮ$_{aa}$ (reactant)	-401.59771	0.15646	-401.14899	
NO (reactant)	-129.89335	0.00471	-129.76980	
NO_2 (product)	-205.07464	0.00917	-204.88891	
Sum reactants – NO_2	-326.41643	0.15199	-326.02988	63.9
PIPC³Ȯ$_{ea}$	-326.42701	0.15083	-326.03806	39.4
SP_{aa-ea}	-326.42305	0.14910	-326.03845	33.8
PIP-C³Ȯ$_{aa}$	-326.43151	0.15057	-326.04777	13.2
SP_{aa}^{16a} ($SP_{aa}^{NC\text{-}C}$)	-326.42666	0.14864	-326.04310	20.4
CHOCH$_2$CH$_2$CH$_2$NHĊH$_2$	-326.43143	0.14656	-326.04880	0.0
SP_{aa}^{16b} ($SP_{aa}^{CC\text{-}C}$)	-326.40638	0.14725	-326.02529	63.5
CHOCH$_2$NHCH$_2$CH$_2$ĊH$_2$	-326.41604	0.14499	-326.03347	36.1
SP_{aa}^{15a}	-326.39520	0.14706	-326.01764	83.1
$POST_{aa}^{15a}$	-326.44481	0.15082	-326.06265	-25.2
SP_{aa}^{15b}	-326.39080	0.14674	-326.01041	101.3
$POST_{aa}^{15b}$	-326.43935	0.15011	-326.05541	-8.0
$SP1_{aa}^{15c}$	-326.41402	0.15105	-326.03098	58.6
$Skew_{aa}^{15c}$	-326.42134	0.15063	-326.03757	40.2
$SP2_{aa}^{15c}$	-326.40986	0.14733	-326.02864	55.0
$POST_{aa}^{15c}$	-326.45149	0.15106	-326.06835	-39.5
PIPC³OȮ$_{ee}$ (reactant)	-401.59736	0.15670	-401.14861	
NO (reactant)	-129.89335	0.00471	-129.76980	
NO_2 (product)	-205.07464	0.00917	-204.88891	
Sum reactants – NO_2	-326.41608	0.15224	-326.02950	65.6
PIPC³OȮ$_{ae}$ (reactant)	-401.59620	0.15646	-401.14757	
NO (reactant)	-129.89335	0.00471	-129.76980	
NO_2 (product)	-205.07464	0.00917	-204.88891	
Sum reactants – NO_2	-326.41491	0.15199	-326.02846	67.6

$PIPC^{3}\dot{O}_{ee}$	-326.43023	0.15138	-326.04672	18.1
SP_{ee-ae}	-326.42410	0.14918	-326.03981	30.5
$PIP\text{-}C^{3}\dot{O}_{ae}$	-326.43126	0.15068	-326.04779	13.5
SP_{ee}^{16a} ($SP_{ee}^{\text{CC-C}}$)	-326.40746	0.14732	-326.02458	65.6
$CHOCH_2NHCH_2CH_2\dot{C}H_2$	-326.41604	0.14499	-326.03347	36.1
SP_{ee}^{16b} ($SP_{ee}^{\text{NC-C}}$)	-326.42404	0.14801	-326.04064	25.3
$CHOCH_2CH_2CH_2NH\dot{C}H_2$	-326.43143	0.14656	-326.04880	0.0
$SP1_{ee}^{15c}$	-326.41257	0.15133	-326.02962	62.9
$Skew_{ee}^{15c}$	-326.42050	0.15122	-326.03687	43.6
$SP2_{ee}^{15c}$	-326.40986	0.14733	-326.02864	55.0
$POST_{ee}^{15c}$	-326.45149	0.15106	-326.06835	-39.5
SP_{ae}^{16a} ($SP_{ae}^{\text{NC-C}}$)	-326.42404	0.14801	-326.04064	25.3
$CHOCH_2CH_2CH_2NH\dot{C}H_2$	-326.43143	0.14656	-326.04880	0.0
SP_{ae}^{16b} ($SP_{ae}^{\text{CC-C}}$)	-326.40635	0.14690	-326.02341	67.6
$CHOCH_2NHCH_2CH_2\dot{C}H_2$	-326.41604	0.14499	-326.03347	36.1
$SP1_{ae}^{15c}$	-326.41417	0.15075	-326.03128	57.0
$Skew_{ae}^{15c}$	-326.42264	0.15091	-326.03935	36.2
$SP2_{ae}^{15c}$	-326.40986	0.14733	-326.02864	55.0
$POST_{ae}^{15c}$	-326.45149	0.15106	-326.06835	-39.5
$CH_2CH_2CH_2NHCH_2C{=}O_e$	-325.89355	0.14042	-325.51030	-117.7
HO_2	-150.90809	0.01458	-150.77891	
O_2	-150.32480	0.00400	-150.19114	
Sum products $-O_2$	-326.47685	0.15100	-326.09807	
$CH_2CH_2CH_2NHCH_2C{=}O_a$	-325.89329	0.14044	-325.51009	-117.1
HO_2	-150.90809	0.01458	-150.77891	
O_2	-150.32480	0.00400	-150.19114	
Sum products $-O_2$	-326.47659	0.15102	-326.09786	

Table S13, continued.
T_1 and D_1 diagnostic values, vibrational frequencies (cm^{-1}), Rotational constants (GHz) and Cartesian coordinates of the species listed above. Results from CCSD(T*)-F12a/aug-cc-pVTZ//M06-2X/aug-cc-pVTZ calculations.

$PIPC^3O\dot{O}_{aa}$	C	-0.134795	-1.077092	0.799136
	N	0.841292	-1.432689	-0.215207
T_1= 0.02368875, D_1= 0.14171092	C	1.931240	-0.466108	-0.299328
	C	1.454123	0.968077	-0.520733
$\tilde{\nu}$: 83.5, 154.2, 193.1, 300.7, 331.8, 395.2, 438.0, 508.1, 565.2,	C	0.453250	1.368495	0.562775
685.8, 764.7, 835.7, 849.7, 877.6, 897.5, 936.8, 967.7, 1035.1,	C	-0.664750	0.348545	0.684283
1060.8, 1085.4, 1149.7, 1166.1, 1201.8, 1241.5, 1285.4, 1295.0,	O	-1.451300	0.473840	-0.536641
1341.8, 1345.3, 1356.0, 1363.2, 1388.5, 1392.9, 1407.4, 1473.9,	O	-2.535435	-0.236173	-0.489913
1481.8, 1486.0, 1498.4, 1500.2, 3039.3, 3054.2, 3062.3, 3064.7,	H	0.333535	-1.174274	1.783039
3101.2, 3107.4, 3112.3, 3116.2, 3131.7, 3544.9	H	-0.971118	-1.773054	0.760782
	H	0.383952	-1.520422	-1.115001
B: 3.397387, 1.731270, 1.435735	H	2.608841	-0.776089	-1.093930
	H	2.491029	-0.522597	0.639947
	H	2.300951	1.655473	-0.519170
	H	0.979192	1.041492	-1.501628
	H	0.031245	2.355091	0.370763
	H	0.957133	1.413742	1.531588
	H	-1.344340	0.576621	1.505692
NO	N	0.000000	0.000000	-0.606410
T_1= 0.0203, D_1= 0.0470	O	0.000000	0.000000	0.530609
$\tilde{\nu}$: 2066.0; B: 0.0000000 52.3561936 52.3561936				
NO_2	N	0.000000	0.000000	0.314442
T_1= 0.0251, D_1= 0.0650; $\tilde{\nu}$: 783.5, 1465.2, 1775.4	O	0.000000	1.090266	-0.137569
B: 253.9654019 13.2904639 12.6295376	O	0.000000	-1.090266	-0.137569
$PIPC^3O\dot{O}_{ea}$	C	1.953319	0.420888	-0.284800
	N	0.818202	1.331672	-0.323346
T_1= 0.02366231, D_1= 0.14149920	C	-0.123182	1.090867	0.753771
	C	-0.675562	-0.321101	0.665247
$\tilde{\nu}$: 88.8, 153.1, 203.0, 307.5, 335.6, 407.7, 426.5, 500.5, 562.5,	C	0.422304	-1.365093	0.565741
664.9, 762.2, 842.7, 867.2, 879.7, 919.4, 938.6, 996.8, 1047.1,	C	1.451639	-0.999913	-0.502335
1073.2, 1103.0, 1154.7, 1168.4, 1200.6, 1227.5, 1289.5, 1299.4,	O	-1.487085	-0.468560	-0.532887
1315.1, 1347.2, 1360.4, 1374.7, 1389.1, 1394.4, 1426.1, 1478.8,	O	-2.558668	0.256938	-0.479718
1487.1, 1491.0, 1494.3, 1512.1, 2947.3, 2957.8, 3063.5, 3077.4,	H	2.490689	0.470419	0.677131
3103.7, 3111.8, 3114.8, 3126.0, 3126.1, 3588.0	H	2.653250	0.701449	-1.070581
	H	1.117811	2.295763	-0.324778
B: 3.4608223, 1.7165706, 1.4182485	H	-0.949159	1.796885	0.685476
	H	0.332074	1.188814	1.752939
	H	-1.342778	-0.514546	1.505213
	H	-0.021509	-2.341934	0.373561
	H	0.909171	-1.415587	1.542975
	H	2.283493	-1.703620	-0.472827
	H	0.994463	-1.060265	-1.490586
$PIPC^3\dot{O}_{aa}$	C	-0.395936	-1.230617	0.545683
	N	0.808211	-1.308036	-0.212338
T_1= 0.02235339, D_1= 0.12278353	C	1.682605	-0.154784	-0.033566
	C	0.979089	1.169292	-0.323509
	C	-0.270864	1.301120	0.536364

$\tilde{\nu}$: 170.0, 220.5, 305.5, 365.5, 393.7, 462.7, 510.1, 586.2, 669.4, 742.3, 830.3, 877.2, 888.2, 909.6, 933.0, 1007.0, 1053.0, 1055.5, 1105.4, 1151.9, 1184.8, 1226.0, 1261.7, 1288.0, 1323.0, 1336.9, 1345.0, 1375.8, 1389.9, 1415.2, 1477.3, 1482.2, 1485.0, 1500.6, 1508.8, 2971.0, 3042.8, 3049.9, 3061.0, 3067.2, 3109.0, 3112.7, 3119.1, 3155.3, 3565.5

B: 3.761512, 2.836641, 2.081705

C	-1.236744	0.123483	0.320209
O	-1.714733	0.029486	-0.920058
H	-0.164635	-1.221965	1.613897
H	-1.047526	-2.070928	0.323687
H	0.603146	-1.451038	-1.192705
H	2.557902	-0.284187	-0.668463
H	2.030315	-0.163349	1.004277
H	1.658968	2.000772	-0.132177
H	0.698755	1.209097	-1.377874
H	-0.803035	2.227083	0.319573
H	0.001055	1.321039	1.596080
H	-2.023464	0.102879	1.089447

SP_{aa-ea}

T_1= 0.02570494, D_1= 0.14776991

$\tilde{\nu}$: -539.0, 169.5, 218.9, 306.5, 331.3, 376.4, 439.8, 517.6, 695.1, 710.9, 837.9, 853.8, 886.1, 919.4, 951.3, 1003.7, 1058.4, 1080.0, 1082.8, 1151.4, 1180.0, 1242.7, 1265.9, 1283.7, 1336.0, 1339.0, 1351.2, 1378.9, 1402.1, 1418.4, 1471.6, 1479.1, 1485.5, 1486.0, 1502.9, 2951.2, 2956.1, 2980.4, 3045.7, 3072.4, 3111.9, 3119.5, 3124.4, 3142.7, 3728.6

B: 3.815277, 2.799334, 2.057730

C	-1.702741	-0.111581	-0.040205
N	-0.807833	-1.219273	-0.235775
C	0.387946	-1.244809	0.513135
C	1.241890	0.078094	0.319446
C	0.294829	1.275998	0.539065
C	-0.962912	1.195127	-0.317211
O	1.753694	0.033671	-0.920353
H	-2.086673	-0.089849	0.992431
H	-2.557482	-0.222791	-0.704740
H	-1.087998	-2.019205	-0.764977
H	0.996385	-2.102917	0.239243
H	0.215541	-1.268201	1.601128
H	2.016480	0.059893	1.099998
H	0.848371	2.193079	0.339733
H	0.026465	1.281311	1.600499
H	-1.611690	2.045777	-0.103711
H	-0.688194	1.231461	-1.371734

$PIPC^3\dot{O}_{ea}$

T_1= 0.01695994, D_1= 0.08506423

$\tilde{\nu}$: 151.5, 209.3, 316.7, 386.8, 420.6, 453.0, 500.4, 685.3, 765.8, 807.8, 871.3, 879.6, 923.3, 960.7, 997.3, 1003.3, 1064.9, 1078.3, 1105.5, 1131.1, 1172.7, 1206.5, 1237.4, 1247.5, 1312.7, 1327.2, 1348.5, 1367.9, 1379.3, 1423.0, 1477.7, 1484.2, 1487.7, 1493.8, 1510.7, 2943.9, 2945.7, 2958.0, 3057.0, 3067.9, 3108.1, 3115.6, 3120.4, 3123.3, 3578.4

B: 3.787492, 2.809806, 2.059650

C	-1.685420	-0.044504	-0.063223
N	-0.830967	-1.186169	-0.360594
C	0.320156	-1.260336	0.526144
C	1.171697	-0.008211	0.325095
C	0.352163	1.263022	0.567507
C	-0.905172	1.241801	-0.298886
O	1.799960	0.000825	-0.898501
H	-2.042819	-0.058884	0.980171
H	-2.558675	-0.084883	-0.713206
H	-1.356964	-2.048083	-0.333795
H	0.913369	-2.137676	0.273759
H	0.033073	-1.322067	1.588632
H	2.017541	-0.032621	1.035132
H	0.970891	2.132781	0.350480
H	0.071284	1.301914	1.623488
H	-1.532317	2.106470	-0.080850
H	-0.618835	1.288998	-1.351465

SP_{aa}^{16a} ($SP_{aa}^{NC\text{-}C}$)

T_1= 0.01854016, D_1= 0.08513076

$\tilde{\nu}$: -262.1, 148.6, 217.6, 273.4, 363.3, 395.4, 426.8, 508.6, 630.5, 655.9, 783.8, 833.1, 848.6, 885.5, 925.2, 956.2, 1009.7, 1025.2, 1060.1, 1076.2, 1145.3, 1221.0, 1249.9, 1285.8, 1334.1, 1355.5,

C	-1.676557	0.193851	-0.096470
N	-1.057887	-1.111505	-0.291743
C	-0.074615	-1.457777	0.593266
C	1.396749	-0.015392	0.257346
C	0.529258	1.185896	0.633748
C	-0.680589	1.338568	-0.281043
O	1.718544	-0.211234	-0.921388

1377.1, 1383.4, 1388.3, 1471.8, 1477.3, 1481.0, 1500.8, 1541.3, 1607.3, 2940.5, 3046.3, 3061.6, 3071.8, 3088.2, 3116.6, 3127.3, 3130.4, 3249.7, 3568.9

B: 3.573580, 2.725236, 2.004458

H	-2.081228	0.210893	0.918587
H	-2.512955	0.287507	-0.787103
H	-0.802012	-1.290445	-1.254462
H	0.490472	-2.347137	0.353686
H	-0.305855	-1.284263	1.638994
H	2.033702	-0.399609	1.074037
H	1.172111	2.066312	0.546653
H	0.223525	1.114235	1.680488
H	-1.190445	2.282224	-0.082568
H	-0.335936	1.359807	-1.316094

$CHOCH_2CH_2CH_2NH\dot{C}H_2$

T_1= 0.02351496, D_1= 0.12097872

$\tilde{\nu}$: 83.4, 116.2, 137.4, 165.8, 226.0, 301.4, 330.7, 359.1, 502.7, 573.2, 651.2, 678.4, 720.9, 838.2, 879.1, 956.8, 1003.4, 1016.5, 1094.3, 1113.9, 1175.2, 1233.8, 1286.8, 1291.3, 1304.2, 1370.3, 1388.0, 1402.9, 1423.5, 1447.7, 1472.2, 1486.6, 1518.5, 1535.4, 1850.4, 2955.8, 3009.9, 3043.7, 3053.5, 3075.6, 3086.0, 3123.9, 3172.3, 3287.8, 3586.4

B: 3.907889, 1.854091, 1.450688

C	2.551557	-0.810392	0.258088
H	3.126146	-0.169879	0.910508
H	2.741762	-1.871392	0.253211
N	1.278244	-0.391889	-0.061895
H	0.773503	-0.968504	-0.720643
C	1.031292	1.027999	-0.208052
H	1.622998	1.455424	-1.026296
H	1.365810	1.511836	0.714621
C	-0.445027	1.322289	-0.433194
H	-0.751337	0.942898	-1.409554
H	-0.578240	2.403363	-0.453040
C	-1.336504	0.707930	0.637522
H	-2.280260	1.255220	0.737907
H	-0.867204	0.746510	1.625076
C	-1.729028	-0.721154	0.377890
H	-2.262483	-1.218952	1.208874
O	-1.534018	-1.312917	-0.650116

SP^{16b}_{aa} ($SP^{CC\text{-}C}_{aa}$)

T_1= 0.01890882, D_1= 0.08815482

$\tilde{\nu}$: -460.2, 155.5, 206.8, 264.5, 346.5, 377.9, 396.1, 509.2, 620.1, 688.4, 778.5, 807.6, 848.5, 871.1, 887.2, 915.2, 919.4, 1029.1, 1058.5, 1078.3, 1149.8, 1192.8, 1227.6, 1253.1, 1330.7, 1343.1, 1369.1, 1382.1, 1389.7, 1452.1, 1473.0, 1475.3, 1495.2, 1510.7, 1611.3, 2946.5, 3011.7, 3044.1, 3046.9, 3098.6, 3099.0, 3116.0, 3129.5, 3235.3, 3542.0

B: 3.500197, 2.747962, 1.999133

C	-0.624422	-1.134672	0.622388
N	0.498847	-1.427322	-0.237838
C	1.594317	-0.483176	-0.082037
C	1.227796	0.987322	-0.322062
C	0.179438	1.479449	0.612750
C	-1.400894	0.131200	0.243197
O	-1.655644	0.369738	-0.938964
H	-0.285078	-1.075659	1.658226
H	-1.346202	-1.955173	0.565085
H	0.178835	-1.432274	-1.200019
H	2.394936	-0.774414	-0.760885
H	1.981627	-0.591728	0.935826
H	2.132894	1.595035	-0.195919
H	0.889280	1.123888	-1.350259
H	-0.346798	2.390173	0.363142
H	0.320690	1.301521	1.674121
H	-2.024371	0.571245	1.041845

$CHOCH_2NHCH_2CH_2\dot{C}H_2$

T_1= 0.01266601, D_1= 0.04498746

$\tilde{\nu}$: 46.0, 95.9, 150.5, 161.6, 177.4, 257.0, 278.9, 355.8, 449.6, 506.1, 695.5, 729.1, 763.5, 838.8, 879.5, 921.6, 971.0, 1028.3, 1091.9, 1114.9, 1136.8, 1200.2, 1215.0, 1295.7, 1305.9, 1359.6, 1379.5, 1400.1, 1410.9, 1450.7, 1460.7, 1466.6, 1510.9, 1513.5, 1859.6, 2937.2, 2969.0, 3018.3, 3033.1, 3068.1, 3071.7, 3076.3, 3178.4, 3283.5, 3526.8

C	2.374150	0.023044	0.108573
H	3.337145	0.354728	0.544332
O	2.256749	-1.080807	-0.347307
C	1.270854	1.059400	0.096324
H	1.183773	1.478089	1.103396
H	1.632998	1.881733	-0.532258
N	-0.014565	0.595461	-0.356835
H	0.107700	0.072350	-1.217719
C	-0.695914	-0.254959	0.616411
H	-0.877780	0.349059	1.509516

B: 6.633696, 1.276673, 1.155221

H	-0.087635	-1.117619	0.911952
C	-2.027753	-0.739569	0.058622
H	-2.472016	-1.448006	0.762166
H	-1.823120	-1.327322	-0.849399
C	-2.975771	0.365382	-0.233634
H	-4.028291	0.167268	-0.364298
H	-2.598205	1.348157	-0.469167

SP^{15a}_{aa}

T_1= 0.02184538, D_1= 0.11949536

$\tilde{\nu}$: --1762.4, 235.4, 248.5, 377.5, 408.9, 500.0, 559.3, 662.3, 732.6, 835.6, 847.4, 887.3, 897.6, 946.6, 955.7, 1010.0, 1025.4, 1075.8, 1100.7, 1124.3, 1166.1, 1204.5, 1231.0, 1269.5, 1301.4, 1307.4, 1332.2, 1342.0, 1353.8, 1360.5, 1390.7, 1479.1, 1489.6, 1496.0, 1515.3, 2040.4, 3048.3, 3052.4, 3062.9, 3081.6

B: 3.642069 3.171939 2.416115

C	0.823647	-0.870655	-0.823478
N	-0.090511	-1.370552	0.221852
C	-1.404828	-0.772890	0.053250
C	-1.357179	0.761661	0.217546
C	-0.155943	1.385091	-0.490830
C	1.089280	0.520247	-0.248467
O	1.231509	0.236751	1.128719
H	0.350463	-0.874277	-1.804437
H	1.720267	-1.487615	-0.837003
H	0.474131	-0.894949	1.103785
H	-2.093382	-1.206442	0.776553
H	-1.755892	-1.029531	-0.950315
H	-2.293418	1.193592	-0.134539
H	-1.272052	0.968912	1.283987
H	0.023250	2.384986	-0.094923
H	-0.329991	1.476667	-1.565874
H	1.988265	1.027792	-0.608079

$POST^{15a}_{aa}$

T_1= 0.01459856, D_1= 0.06391108

$\tilde{\nu}$: 155.0, 213.3, 303.9, 357.9, 388.9, 415.6, 497.9, 522.8, 689.3, 816.7, 840.1, 878.4, 885.3, 931.8, 1009.7, 1018.8, 1030.6, 1092.8, 1098.0, 1151.0, 1175.1, 1190.0, 1247.6, 1280.9, 1300.1, 1318.9, 1330.9, 1352.0, 1375.1, 1394.0, 1426.8, 1477.3, 1492.5, 1494.6, 1519.1, 2990.0, 3005.7, 3055.9, 3070.6, 3094.1, 3113.0, 3113.6, 3118.1, 3119.6, 3851.0

B: 3.824589 2.788628 2.121606

C	-0.364992	-1.238553	0.579598
N	0.691380	-1.292321	-0.398270
C	1.692692	-0.295677	-0.117724
C	1.070189	1.101308	-0.304689
C	-0.147012	1.260167	0.599176
C	-1.119258	0.097004	0.419373
O	-1.774031	0.148643	-0.834761
H	0.033221	-1.286932	1.601799
H	-1.056363	-2.065195	0.423002
H	-1.193631	-0.235412	-1.499143
H	2.538909	-0.430710	-0.789277
H	2.048539	-0.383314	0.918665
H	1.820736	1.862473	-0.092825
H	0.778261	1.219064	-1.350001
H	-0.671344	2.191310	0.384439
H	0.172351	1.290280	1.644489
H	-1.907805	0.150048	1.170422

SP^{15b}_{aa}

T_1= 0.02410265, D_1= 0.12819582

$\tilde{\nu}$: -1866.2, 240.1, 255.9, 371.0, 419.0, 482.9, 582.7, 648.0, 748.6, 830.4, 840.1, 857.5, 895.8, 923.1, 954.1, 990.9, 1033.7, 1041.7, 1077.0, 1111.9, 1135.4, 1175.6, 1207.9, 1236.9, 1272.6, 1297.6, 1332.6, 1345.4, 1350.5, 1357.1, 1395.1, 1478.2, 1483.7, 1498.1, 1506.6, 1784.6, 3001.3, 3047.8, 3064.0, 3074.5, 3115.1, 3119.8, 3130.5, 3145.2, 3550.6

B: 3.647099 3.120111 2.393729

C	-0.173074	-1.349712	-0.520212
N	-1.351067	-0.816900	0.159898
C	-1.472859	0.633949	0.040401
C	-0.157858	1.375316	0.199217
C	0.887693	0.898928	-0.800773
C	1.080178	-0.509355	-0.240642
O	1.208075	-0.280078	1.153128
H	-0.358593	-1.377926	-1.598077
H	-0.022845	-2.373950	-0.181358
H	-1.262641	-1.042624	1.143085
H	-2.212214	0.977399	0.762262
H	-1.866305	0.868444	-0.957993

H	-0.237047	2.441000	0.396635
H	0.436377	0.784198	1.165034
H	1.808998	1.472574	-0.719789
H	0.535769	0.925350	-1.834755
H	1.986877	-1.010298	-0.587308

POST_{aa}^{15b}

T_1= 0.01235902, D_1= 0.04128506

$\tilde{\nu}$: 147.4, 189.1, 265.8, 307.8, 367.6, 385.5, 425.8, 516.1, 596.0, 746.3, 812.9, 834.6, 876.1, 899.0, 903.8, 968.6, 1033.9, 1068.7, 1090.4, 1137.9, 1151.0, 1197.5, 1200.3, 1285.3, 1315.2, 1338.4, 1350.8, 1371.7, 1380.3, 1398.9, 1425.4, 1463.4, 1467.9, 1481.6, 1483.1, 2968.3, 2979.1, 3046.3, 3090.6, 3105.9, 3109.7, 3118.5, 3205.9, 3538.4, 3843.0

B: 3.762948 2.778308 2.096416

C	-0.200685	-1.211077	0.632442
N	0.966067	-1.213546	-0.239764
C	1.723622	0.037837	-0.145065
C	0.873045	1.259979	-0.262670
C	-0.373986	1.290966	0.556224
C	-1.140371	-0.026306	0.397267
O	-1.738843	-0.109232	-0.887477
H	0.149015	-1.189365	1.669456
H	-0.751324	-2.139312	0.484146
H	0.667125	-1.360375	-1.196960
H	2.515097	0.028708	-0.893261
H	2.221984	0.029540	0.836592
H	1.281153	2.169559	-0.679790
H	-1.123436	0.255909	-1.530888
H	-1.018569	2.128070	0.288911
H	-0.118387	1.406751	1.620002
H	-1.964130	-0.069202	1.110776

$\mathrm{SP1}_{aa}^{15c}$

T_1= 0.01979059, D_1= 0.10580854

$\tilde{\nu}$: -215.9, 68.9, 268.2, 309.8, 369.1, 466.6, 584.7, 711.4, 737.8, 777.3, 828.4, 859.9, 871.8, 950.0, 976.5, 1003.1, 1040.6, 1106.8, 1151.7, 1165.4, 1199.5, 1244.6, 1287.6, 1297.7, 1332.0, 1343.6, 1359.7, 1366.0, 1382.1, 1408.5, 1486.5, 1488.5, 1497.9, 1503.8, 1520.5, 3016.2, 3057.5, 3058.6, 3081.0, 3092.5, 3106.1, 3114.1, 3124.9, 3139.0, 3546.0

B: 3.866503 2.695052 1.957344

C	0.392479	-1.121420	-0.677127
N	-0.632149	-1.334751	0.315516
C	-1.695366	-0.350903	0.190326
C	-1.243931	1.122259	-0.009117
C	0.271104	1.390059	-0.147191
C	1.202505	0.154566	-0.393831
O	1.884941	-0.002054	0.783046
H	-0.072749	-1.041153	-1.663441
H	1.076851	-1.969188	-0.700521
H	-0.209240	-1.297383	1.235491
H	-2.333147	-0.429882	1.068520
H	-2.304852	-0.650443	-0.664380
H	-1.736844	1.507737	-0.900480
H	-1.615149	1.722674	0.819506
H	0.645078	1.890943	0.744289
H	0.447146	2.078161	-0.971414
H	1.887680	0.380865	-1.218904

$\mathrm{Skew}_{\mathrm{aa}}^{15\mathrm{c}}$

T_1= 0.02327492, D_1= 0.13064679

$\tilde{\nu}$: 87.2, 226.8, 271.5, 349.5, 380.4, 476.7, 483.6, 648.4, 701.6, 743.1, 823.1, 864.7, 893.5, 903.9, 928.0, 1022.0, 1056.8, 1078.4, 1098.6, 1176.7, 1201.6, 1221.1, 1273.7, 1281.4, 1296.0, 1319.8, 1349.1, 1377.3, 1400.5, 1409.3, 1476.6, 1489.2, 1505.3, 1509.0, 1521.5, 2983.8, 3015.4, 3057.7, 3067.2, 3073.6, 3093.7, 3113.3, 3126.2, 3152.9, 3588.2

B: 3.629275 2.889703 2.165679

C	-0.465360	-0.969347	0.838103
N	0.677623	-1.399392	0.080923
C	1.418654	-0.325400	-0.593838
C	1.281281	0.966887	0.193957
C	-0.169982	1.457619	0.170936
C	-1.198319	0.306768	0.233618
O	-1.662338	-0.088560	-0.962335
H	-0.177928	-0.646141	1.841395
H	-1.178932	-1.783374	0.930758
H	0.398407	-2.095208	-0.594471
H	1.027794	-0.160699	-1.605163
H	2.465304	-0.617116	-0.679254
H	1.612489	0.790208	1.219611
H	1.941196	1.725681	-0.224230

H	-0.362206	2.007609	-0.750701
H	-0.353269	2.140900	1.001039
H	-2.015165	0.523200	0.936584

$SP2_{aa}^{15c}$

T_1= 0.03357204, D_1= 0.19710945

$\tilde{\nu}$: -957.9, 110.7, 318.5, 395.4, 404.9, 525.1, 553.4, 668.5, 718.4, 813.0, 837.5, 874.7, 916.4, 917.6, 945.3, 1034.1, 1049.4, 1070.0, 1096.8, 1133.9, 1156.6, 1195.8, 1225.4, 1238.1, 1270.0, 1277.3, 1311.6, 1320.4, 1341.0, 1350.3, 1405.1, 1476.1, 1478.5, 1496.4, 1507.9, 1863.5, 3020.0, 3061.2, 3072.7, 3088.7, 3122.6, 3126.7, 3139.8, 3145.2, 3596.5

B: 3.369290 3.244673 2.517362

C	-0.853906	0.890988	-0.779991
N	-1.509878	-0.300076	-0.227662
C	-0.624998	-1.056053	0.587808
C	0.635605	-1.444553	-0.165195
C	1.399773	-0.152863	-0.552895
C	0.533057	1.032500	-0.112611
O	0.422816	1.053383	1.262950
H	-1.467624	1.770206	-0.590411
H	-0.717601	0.789361	-1.858826
H	-2.358842	-0.068379	0.265667
H	-1.130638	-1.862144	1.113317
H	-0.193677	-0.252837	1.382183
H	1.246644	-2.090798	0.460788
H	0.353552	-2.010154	-1.054735
H	1.591466	-0.112545	-1.624980
H	2.354395	-0.095141	-0.033707
H	0.971755	1.985781	-0.431954

$POST_{aa}^{15c}$

T_1= 0.01509806, D_1= 0.06543331

$\tilde{\nu}$: 175.3, 219.9, 266.6, 338.5, 365.6, 426.4, 467.5, 482.1, 528.8, 660.2, 731.7, 829.0, 896.4, 929.4, 977.9, 1002.9, 1071.7, 1077.4, 1128.7, 1137.8, 1179.5, 1209.3, 1250.9, 1255.1, 1307.8, 1326.6, 1360.1, 1378.0, 1395.2, 1425.7, 1451.4, 1475.4, 1489.5, 1504.3, 1514.3, 2964.2, 2968.9, 3031.4, 3079.5, 3109.3, 3111.2, 3122.7, 3205.5, 3621.1, 3857.9

B: 4.559424 2.368242 1.676367

C	0.289678	-1.244464	-0.155465
N	-1.130179	-1.211594	0.160743
C	-1.838681	-0.071411	-0.196423
C	-1.208639	1.238625	0.140971
C	0.276611	1.250517	-0.226566
C	0.969466	0.024164	0.345183
H	0.456234	-1.310144	-1.241844
H	0.746842	-2.113468	0.314490
H	-1.608154	-2.076708	-0.034216
H	-2.914462	-0.169651	-0.166962
H	-1.735452	2.045787	-0.366479
H	-1.296007	1.435498	1.220249
H	0.767798	2.151671	0.140705
H	0.381456	1.229227	-1.317298
H	0.905647	0.047329	1.435167
O	2.351994	-0.005647	0.043785
H	2.460792	0.102201	-0.905499

$PIPC^3O\dot{O}_{ee}$

T_1= 0.02371353, D_1= 0.14214839

$\tilde{\nu}$: 124.8, 136.9, 241.2, 314.8, 324.4, 371.6, 433.0, 451.1, 530.3, 672.3, 761.6, 825.2, 885.1, 894.5, 957.4, 970.7, 995.0, 1041.6, 1073.6, 1130.4, 1158.9, 1180.8, 1190.6, 1231.5, 1250.9, 1297.4, 1309.5, 1334.6, 1347.7, 1375.6, 1378.4, 1407.4, 1428.3, 1481.2, 1487.9, 1499.4, 1505.7, 1518.9, 2966.1, 2999.5, 3067.6, 3072.4, 3105.0, 3112.7, 3114.5, 3117.9, 3128.1, 3577.7

B: 3.9042221, 1.5412312, 1.2747997

C	-2.050484	-0.063992	0.453183
N	-1.428371	-1.194930	-0.226315
C	0.007361	-1.243040	0.004578
C	0.617015	0.010948	-0.603649
C	0.027204	1.267422	0.007722
C	-1.495611	1.231739	-0.122743
O	2.060250	-0.019032	-0.461718
O	2.438534	0.016097	0.780053
H	-1.861139	-0.083146	1.537840
H	-3.127625	-0.116554	0.300522
H	-1.858013	-2.065195	0.053867
H	0.426456	-2.126099	-0.476658
H	0.273503	-1.266978	1.070466
H	0.476915	-0.001571	-1.684519
H	0.446444	2.149622	-0.476346
H	0.310960	1.296127	1.061491

H	-1.931978	2.087894	0.391322
H	-1.780109	1.295422	-1.175013

PIPC^{3}O$\dot{\mathrm{O}}_{ae}$

T_1= 0.02363597, D_1= 0.14156964

$\tilde{\nu}$: 88.3, 143.1, 240.1, 255.2, 339.3, 384.1, 451.5, 468.2, 531.3, 601.9, 754.0, 823.2, 879.8, 891.6, 937.0, 968.3, 982.8, 1054.9, 1061.3, 1125.1, 1151.0, 1184.6, 1190.8, 1239.6, 1288.7, 1300.6, 1323.2, 1334.7, 1343.0, 1377.1, 1385.0, 1392.0, 1405.9, 1481.2, 1490.1, 1491.2, 1498.0, 1509.0, 3044.1, 3050.8, 3054.2, 3056.2, 3079.2, 3106.4, 3116.9, 3122.3, 3124.9, 3544.9

B: 4.2175274, 1.4306304, 1.1469785

C	-0.262306	-1.331393	-0.174827
N	-1.645798	-1.135928	0.230314
C	-2.182483	0.146245	-0.215069
C	-1.329711	1.343988	0.202125
C	0.113095	1.151437	-0.260808
C	0.634571	-0.176814	0.253263
O	1.946913	-0.446149	-0.296395
O	2.854308	0.327794	0.215981
H	-0.229005	-1.408555	-1.265487
H	0.107729	-2.271603	0.231846
H	-1.730486	-1.219580	1.236262
H	-3.199908	0.242359	0.161977
H	-2.245349	0.112113	-1.306874
H	-1.738604	2.265572	-0.213740
H	-1.356827	1.442850	1.291236
H	0.761792	1.956411	0.082254
H	0.152538	1.132693	-1.353939
H	0.749947	-0.154697	1.339470

PIPC$^3\dot{\mathrm{O}}_{ee}$

T_1= 0.01768328, D_1= 0.09024537

$\tilde{\nu}$: 175.1, 250.3, 337.9, 369.4, 416.9, 436.4, 459.8, 564.6, 755.6, 800.8, 876.0, 889.5, 926.6, 974.4, 977.1, 1057.3, 1071.4, 1125.4, 1131.7, 1159.5, 1192.7, 1216.8, 1267.8, 1284.4, 1293.9, 1330.3, 1336.3, 1371.0, 1375.1, 1419.7, 1479.5, 1484.3, 1492.7, 1500.3, 1513.6, 2961.3, 2973.9, 2996.2, 3069.1, 3070.5, 3110.2, 3114.3, 3116.4, 3135.6, 3584.7

B: 4.481569, 2.437385, 1.729456

C	-1.760685	-0.012195	0.258012
N	-1.073919	-1.167801	-0.300383
C	0.295486	-1.262684	0.172027
C	1.080820	-0.054380	-0.339388
C	0.414012	1.246179	0.184247
C	-1.061294	1.257465	-0.207441
O	2.363880	-0.021081	0.114709
H	-1.767397	-0.031591	1.359575
H	-2.795470	-0.018561	-0.082167
H	-1.580837	-2.020543	-0.111945
H	0.748314	-2.176983	-0.209631
H	0.379189	-1.269617	1.269670
H	1.044424	-0.015113	-1.437210
H	0.945756	2.106158	-0.218106
H	0.519810	1.259098	1.271214
H	-1.543947	2.131624	0.230973
H	-1.153483	1.332470	-1.292093

SP$_{ee-ae}$

T_1= 0.02685848, D_1= 0.15645230

$\tilde{\nu}$: -578.6, 158.0, 200.1, 256.0, 355.4, 410.5, 445.8, 472.6, 599.4, 789.1, 821.8, 868.5, 900.6, 913.2, 975.2, 1010.2, 1047.4, 1099.2, 1146.9, 1176.5, 1210.9, 1219.5, 1244.2, 1290.2, 1324.8, 1333.6, 1348.0, 1351.7, 1373.7, 1406.0, 1463.6, 1482.4, 1489.1, 1490.3, 1503.2, 2971.5, 2977.4, 2995.7, 3062.6, 3066.8, 3109.2, 3122.2, 3129.2, 3131.7, 3739.3

B: 4.499801 2.419801 1.722468

C	-1.785099	-0.049310	0.240728
N	-1.034252	-1.201132	-0.175010
C	0.340798	-1.258639	0.193743
C	1.092461	-0.015371	-0.350493
C	0.369915	1.244346	0.201834
C	-1.096376	1.234833	-0.220663
O	2.369570	0.021935	0.089618
H	-1.872343	-0.022796	1.337389
H	-2.793654	-0.111425	-0.163913
H	-1.456143	-1.928858	-0.713125
H	0.797977	-2.161938	-0.204875
H	0.481993	-1.255942	1.283434
H	1.017882	0.007069	-1.448160
H	0.883905	2.131587	-0.163749
H	0.458683	1.226979	1.290597
H	-1.599097	2.103612	0.207184

H	-1.166202	1.309007	-1.307550

PIPC$^{3}\dot{O}_{ae}$

T_1= 0.02621835, D_1= 0.15072251

$\tilde{\nu}$: 168.2, 252.9, 333.3, 365.1, 413.3, 444.8, 477.5, 567.9, 611.4, 727.0, 815.7, 875.9, 894.9, 934.2, 970.2, 1014.8, 1066.4, 1092.5, 1118.3, 1172.3, 1188.8, 1234.1, 1267.3, 1293.3, 1311.6, 1340.6, 1341.4, 1369.7, 1378.8, 1389.2, 1480.3, 1485.5, 1486.6, 1498.2, 1504.0, 2940.6, 3045.5, 3055.9, 3061.6, 3090.3, 3103.4, 3114.7, 3120.9, 3165.9, 3557.6

B: 4.433826, 2.431154, 1.728931

C	-0.398004	-1.234466	0.198652
N	0.982126	-1.297643	-0.171566
C	1.761942	-0.146221	0.267116
C	1.175011	1.179057	-0.211722
C	-0.290320	1.290816	0.192558
C	-1.103378	0.105829	-0.343419
O	-2.353616	0.036558	0.102277
H	-0.493335	-1.211006	1.284036
H	-0.945591	-2.085347	-0.195747
H	1.091106	-1.454596	-1.165459
H	2.787282	-0.273089	-0.077274
H	1.780129	-0.162093	1.360429
H	1.746087	2.010760	0.202966
H	1.264893	1.240982	-1.300259
H	-0.731048	2.214199	-0.183377
H	-0.390650	1.299438	1.280936
H	-1.026335	0.041697	-1.442615

SP_{ee}^{16a} ($SP_{ee}^{\text{CC-C}}$)

T_1= 0.01852463, D_1= 0.08548979

$\tilde{\nu}$: -451.2, 145.1, 221.2, 304.8, 326.4, 388.3, 397.9, 475.8, 558.9, 649.4, 739.2, 817.1, 854.5, 884.9, 904.2, 929.8, 1002.8, 1035.2, 1085.8, 1098.3, 1156.8, 1199.4, 1221.9, 1246.0, 1307.5, 1324.1, 1368.8, 1376.2, 1415.7, 1458.0, 1474.9, 1478.2, 1491.5, 1506.9, 1616.7, 2945.6, 2965.9, 2998.0, 3012.2, 3087.6, 3099.6, 3115.0, 3143.0, 3242.7, 3594.1

B: 4.117280 2.324378 1.629865

C	-1.745045	-0.235170	0.241220
N	-0.875593	-1.264054	-0.303673
C	0.471373	-1.231233	0.229705
C	1.296684	-0.101521	-0.373385
C	0.069360	1.491752	0.237545
C	-1.299675	1.140681	-0.241028
O	2.386511	0.215987	0.100961
H	-1.748884	-0.239225	1.342635
H	-2.762438	-0.428494	-0.095878
H	-1.284778	-2.178748	-0.185118
H	0.982563	-2.155532	-0.049016
H	0.519595	-1.141729	1.323648
H	1.048783	0.121413	-1.427643
H	0.607038	2.289732	-0.255497
H	0.268620	1.401847	1.299196
H	-2.006206	1.893001	0.131754
H	-1.343416	1.171165	-1.330392

SP_{ee}^{16b} ($SP_{ee}^{\text{NC-C}}$)

T_1= 0.01854446, D_1= 0.08335400

$\tilde{\nu}$: -242.7, 138.6, 234.0, 309.2, 313.1, 380.9, 420.1, 488.4, 550.2, 588.0, 733.5, 790.4, 849.4, 908.3, 916.5, 941.0, 989.9, 1040.3, 1068.4, 1091.1, 1158.0, 1221.7, 1240.4, 1285.9, 1334.3, 1339.2, 1385.8, 1387.1, 1391.0, 1467.6, 1472.0, 1481.3, 1497.7, 1532.6, 1645.4, 2897.5, 3045.0, 3048.3, 3064.5, 3093.4, 3101.2, 3126.6, 3144.2, 3260.5, 3598.0

B: 4.127471 2.256774 1.603911

C	1.789334	0.127102	-0.251143
N	1.260769	-1.152236	0.198927
C	0.002448	-1.485667	-0.233624
C	-1.329904	0.107771	0.366056
C	-0.502620	1.232808	-0.245633
C	0.944476	1.304299	0.230600
O	-2.420612	-0.213240	-0.096640
H	1.805472	0.107584	-1.343544
H	2.818618	0.216372	0.092361
H	1.446555	-1.358085	1.169542
H	-0.469185	-2.335269	0.238299
H	-0.187072	-1.342568	-1.290244
H	-1.088035	-0.108837	1.426467
H	-1.021225	2.158697	0.020859
H	-0.561478	1.147265	-1.332930
H	1.402383	2.230016	-0.121681
H	0.971077	1.338523	1.323958

$SP1_{ee}^{15c}$

T_1= 0.01716176, D_1= 0.08624086

$\tilde{\nu}$: -207.7, 43.4, 333.7, 351.8, 407.0, 441.5, 500.6, 644.9, 735.8, 766.9, 858.6, 882.1, 888.3, 975.9, 1018.3, 1057.0, 1107.3, 1113.6, 1151.5, 1186.8, 1226.6, 1237.1, 1284.9, 1298.8, 1308.3, 1335.7, 1354.3, 1366.1, 1396.1, 1424.8, 1476.9, 1487.3, 1502.1, 1508.4, 1528.0, 2975.7, 2977.6, 3016.0, 3083.9, 3097.1, 3107.5, 3116.2, 3122.5, 3149.9, 3581.3

B: 4.288205 2.430343 1.748301

C	-1.777292	-0.026027	0.265313
N	-1.072323	-1.091410	-0.428385
C	0.232910	-1.276084	0.178499
C	1.148970	-0.173973	-0.341044
C	0.455745	1.237770	-0.324889
C	-1.011274	1.306794	0.157546
O	2.278885	-0.006203	0.410753
H	-1.907168	-0.277154	1.327516
H	-2.770260	0.082435	-0.167761
H	-1.610627	-1.945455	-0.401142
H	0.642579	-2.245341	-0.102095
H	0.203415	-1.220784	1.277176
H	1.410951	-0.388993	-1.383390
H	0.528871	1.620304	-1.338743
H	1.067237	1.892488	0.294067
H	-1.043868	1.799287	1.127465
H	-1.540306	1.961823	-0.532977

$Skew_{ee}^{15c}$

T_1= 0.02621427, D_1= 0.14233931

$\tilde{\nu}$: 122.9, 187.5, 300.3, 350.9, 386.0, 475.1, 508.2, 648.5, 777.4, 799.8, 834.6, 884.9, 896.3, 950.9, 988.6, 1025.4, 1064.1, 1087.1, 1108.4, 1160.2, 1195.8, 1214.5, 1242.1, 1296.0, 1304.6, 1335.3, 1345.4, 1365.4, 1393.4, 1420.4, 1473.5, 1474.0, 1494.2, 1499.7, 1520.2, 2999.2, 3000.8, 3014.6, 3078.2, 3089.9, 3095.2, 3114.1, 3125.7, 3149.2, 3577.6

B: 3.606961 2.935395 2.208889

C	1.412762	-0.160215	0.591361
N	1.183507	0.968129	-0.306672
C	-0.196457	1.434275	-0.224705
C	-1.186042	0.252695	-0.242401
C	-0.492613	-1.003402	-0.833777
C	0.728550	-1.400153	0.000742
O	-1.599358	-0.121606	1.007342
H	1.014586	0.033909	1.595863
H	2.484726	-0.324590	0.683468
H	1.818004	1.727071	-0.102914
H	-0.401241	2.081332	-1.078985
H	-0.398837	2.003913	0.691690
H	-2.056092	0.480533	-0.869488
H	-0.190406	-0.719625	-1.841019
H	-1.214411	-1.813949	-0.903793
H	0.432199	-2.071003	0.806313
H	1.424588	-1.940837	-0.640489

$SP2_{ee}^{15c}$ same as $SP2_{aa}^{15c}$

T_1= 0.03357204, D_1= 0.19710945

$\tilde{\nu}$: -957.9, 110.7, 318.5, 395.4, 404.9, 525.1, 553.4, 668.5, 718.4, 813.0, 837.5, 874.7, 916.4, 917.6, 945.3, 1034.1, 1049.4, 1070.0, 1096.8, 1133.9, 1156.6, 1195.8, 1225.4, 1238.1, 1270.0, 1277.3, 1311.6, 1320.4, 1341.0, 1350.3, 1405.1, 1476.1, 1478.5, 1496.4, 1507.9, 1863.5, 3020.0, 3061.2, 3072.7, 3088.7, 3122.6, 3126.7, 3139.8, 3145.2, 3596.5

B: 3.369290 3.244673 2.517362

C	-0.853906	0.890988	-0.779991
N	-1.509878	-0.300076	-0.227662
C	-0.624998	-1.056053	0.587808
C	0.635605	-1.444553	-0.165195
C	1.399773	-0.152863	-0.552895
C	0.533057	1.032500	-0.112611
O	0.422816	1.053383	1.262950
H	-1.467624	1.770206	-0.590411
H	-0.717601	0.789361	-1.858826
H	-2.358842	-0.068379	0.265667
H	-1.130638	-1.862144	1.113317
H	-0.193677	-0.252837	1.382183
H	1.246644	-2.090798	0.460788
H	0.353552	-2.010154	-1.054735
H	1.591466	-0.112545	-1.624980
H	2.354395	-0.095141	-0.033707
H	0.971755	1.985781	-0.431954

$POST_{ee}^{15c}$ same as $POST_{ae}^{15c}$

C	0.289678	-1.244464	-0.155465

T_1= 0.01509806, D_1= 0.06543331

$\tilde{\nu}$: 175.3, 219.9, 266.6, 338.5, 365.6, 426.4, 467.5, 482.1, 528.8, 660.2, 731.7, 829.0, 896.4, 929.4, 977.9, 1002.9, 1071.7, 1077.4, 1128.7, 1137.8, 1179.5, 1209.3, 1250.9, 1255.1, 1307.8, 1326.6, 1360.1, 1378.0, 1395.2, 1425.7, 1451.4, 1475.4, 1489.5, 1504.3, 1514.3, 2964.2, 2968.9, 3031.4, 3079.5, 3109.3, 3111.2, 3122.7, 3205.5, 3621.1, 3857.9

B: 4.559424 2.368242 1.676367

N	-1.130179	-1.211594	0.160743
C	-1.838681	-0.071411	-0.196423
C	-1.208639	1.238625	0.140971
C	0.276611	1.250517	-0.226566
C	0.969466	0.024164	0.345183
H	0.456234	-1.310144	-1.241844
H	0.746842	-2.113468	0.314490
H	-1.608154	-2.076708	-0.034216
H	-2.914462	-0.169651	-0.166962
H	-1.735452	2.045787	-0.366479
H	-1.296007	1.435498	1.220249
H	0.767798	2.151671	0.140705
H	0.381456	1.229227	-1.317298
H	0.905647	0.047329	1.435167
O	2.351994	-0.005647	0.043785
H	2.460792	0.102201	-0.905499

SP$_{ae}^{16a}$ (SP$_{ae}^{\text{NC-C}}$)

T_1= 0.01854446, D_1= 0.08335400

$\tilde{\nu}$: -242.7, 138.6, 234.0, 309.2, 313.1, 380.9, 420.1, 488.4, 550.2, 588.0, 733.5, 790.4, 849.4, 908.3, 916.5, 941.0, 989.9, 1040.3, 1068.4, 1091.1, 1158.0, 1221.7, 1240.4, 1285.9, 1334.3, 1339.2, 1385.8, 1387.1, 1391.0, 1467.6, 1472.0, 1481.3, 1497.7, 1532.6, 1645.4, 2897.5, 3045.0, 3048.3, 3064.5, 3093.4, 3101.2, 3126.6, 3144.2, 3260.5, 3598.0

B: 4.127472, 2.256774, 1.603911

C	-0.002448	-1.485667	-0.233623
N	-1.260769	-1.152236	0.198927
C	-1.789334	0.127102	-0.251143
C	-0.944476	1.304299	0.230600
C	0.502620	1.232808	-0.245633
C	1.329904	0.107771	0.366056
O	2.420612	-0.213240	-0.096640
H	0.187072	-1.342568	-1.290244
H	0.469185	-2.335269	0.238300
H	-1.446556	-1.358086	1.169541
H	-2.818618	0.216372	0.092361
H	-1.805472	0.107584	-1.343544
H	-1.402383	2.230016	-0.121681
H	-0.971077	1.338523	1.323958
H	1.021225	2.158697	0.020859
H	0.561478	1.147265	-1.332930
H	1.088035	-0.108837	1.426467

SP$_{ae}^{16b}$ (SP$_{ae}^{\text{CC-C}}$)

T_1= 0.01873274, D_1= 0.08658376

$\tilde{\nu}$: -437.1, 139.3, 214.4, 294.3, 321.4, 363.8, 401.3, 473.3, 569.2, 635.4, 704.2, 796.6, 831.4, 880.1, 899.0, 911.0, 991.5, 1039.8, 1052.8, 1072.6, 1159.2, 1190.7, 1228.6, 1249.4, 1323.4, 1333.7, 1376.3, 1380.3, 1392.8, 1457.5, 1462.6, 1475.9, 1493.2, 1500.8, 1625.1, 2892.9, 3002.2, 3049.0, 3053.7, 3078.6, 3104.9, 3115.7, 3144.2, 3244.0, 3557.5

B: 4.062772, 2.310727, 1.621730

C	-0.490476	-1.230681	0.254367
N	0.886555	-1.359160	-0.167907
C	1.748488	-0.264754	0.237902
C	1.311020	1.119903	-0.252171
C	-0.037423	1.500613	0.253536
C	-1.292962	-0.085698	-0.366813
O	-2.389211	0.245168	0.076344
H	-0.536915	-1.135827	1.340919
H	-1.027439	-2.146605	-0.007435
H	0.950968	-1.528229	-1.163596
H	2.759066	-0.476379	-0.109704
H	1.779606	-0.257889	1.331431
H	2.044357	1.860837	0.093211
H	1.331005	1.150755	-1.344150
H	-0.578682	2.299187	-0.234270
H	-0.221381	1.411987	1.317930
H	-1.024660	0.138636	-1.420665

SP1$_{ae}^{15c}$

C	0.439401	-1.320270	0.147772
N	-0.982141	-1.258885	0.301701

T_1= 0.02383978, D_1= 0 0.13418405

$\tilde{\nu}$: -162.4, 67.1, 341.7, 369.3, 400.6, 470.0, 495.5, 586.9, 655.7, 760.1, 804.8, 832.8, 892.8, 937.6, 977.0, 1013.6, 1087.6, 1114.4, 1154.2, 1192.2, 1199.6, 1241.5, 1267.3, 1286.0, 1329.7, 1334.1, 1360.3, 1381.0, 1385.5, 1403.7, 1479.8, 1488.7, 1496.2, 1507.3, 1514.1, 2982.3, 3059.2, 3068.4, 3082.0, 3101.1, 3102.3, 3117.0, 3128.7, 3163.0, 3538.7

B: 3.896767 2.583177 1.860558

C	-1.557471	-0.232099	-0.556497
C	-1.224391	1.168017	-0.049670
C	0.246434	1.330586	0.396963
C	1.188925	0.102251	0.349540
O	2.055034	0.122309	-0.677219
H	0.664984	-1.654402	-0.862366
H	0.879292	-2.022891	0.849411
H	-1.246458	-1.103894	1.267718
H	-2.636857	-0.368679	-0.615275
H	-1.153737	-0.391079	-1.558377
H	-1.471921	1.906050	-0.812257
H	-1.879943	1.374201	0.798468
H	0.264639	1.711909	1.417997
H	0.736722	2.086894	-0.213994
H	1.720603	0.004710	1.305872

Skew_{ae}^{15c}

T_1= 0.02444510, D_1= 0.13802092

$\tilde{\nu}$: 107.2, 198.9, 301.1, 351.7, 380.1, 509.7, 526.0, 587.4, 697.6, 770.8, 801.2, 850.8, 881.1, 927.4, 979.0, 998.2, 1057.5, 1075.2, 1104.6, 1171.3, 1200.2, 1220.4, 1241.3, 1287.4, 1308.1, 1331.0, 1353.4, 1365.7, 1377.8, 1407.5, 1486.8, 1487.7, 1498.1, 1502.1, 1508.8, 2996.4, 3059.8, 3077.0, 3085.8, 3094.4, 3111.7, 3122.3, 3136.7, 3151.5, 3552.6

B: 3.592383 2.913219 2.221290

C	0.387114	1.386116	-0.225421
N	-1.032881	1.186007	-0.237353
C	-1.431999	0.071784	0.616351
C	-0.908053	-1.283124	0.087120
C	0.297415	-1.080622	-0.834605
C	1.225621	-0.002854	-0.275200
O	1.591718	-0.223431	0.996463
H	0.689496	1.904305	0.682094
H	0.692668	1.974456	-1.087957
H	-1.367128	1.038838	-1.181112
H	-2.516787	0.063011	0.705793
H	-1.022916	0.269893	1.606861
H	-0.624738	-1.914713	0.927744
H	-1.695088	-1.803884	-0.459133
H	-0.021927	-0.772193	-1.833464
H	0.852535	-2.010189	-0.955136
H	2.089718	0.188065	-0.925394

$\mathrm{SP2}_{ae}^{15c}$ same as $\mathrm{SP2}_{ee}^{15c}$ and $\mathrm{SP2}_{aa}^{15c}$

T_1= 0.03357204, D_1= 0.19710945

$\tilde{\nu}$: -957.9, 110.7, 318.5, 395.4, 404.9, 525.1, 553.4, 668.5, 718.4, 813.0, 837.5, 874.7, 916.4, 917.6, 945.3, 1034.1, 1049.4, 1070.0, 1096.8, 1133.9, 1156.6, 1195.8, 1225.4, 1238.1, 1270.0, 1277.3, 1311.6, 1320.4, 1341.0, 1350.3, 1405.1, 1476.1, 1478.5, 1496.4, 1507.9, 1863.5, 3020.0, 3061.2, 3072.7, 3088.7, 3122.6, 3126.7, 3139.8, 3145.2, 3596.5

B: 3.369290 3.244673 2.517362

C	-0.853906	0.890988	-0.779991
N	-1.509878	-0.300076	-0.227662
C	-0.624998	-1.056053	0.587808
C	0.635605	-1.444553	-0.165195
C	1.399773	-0.152863	-0.552895
C	0.533057	1.032500	-0.112611
O	0.422816	1.053383	1.262950
H	-1.467624	1.770206	-0.590411
H	-0.717601	0.789361	-1.858826
H	-2.358842	-0.068379	0.265667
H	-1.130638	-1.862144	1.113317
H	-0.193677	-0.252837	1.382183
H	1.246644	-2.090798	0.460788
H	0.353552	-2.010154	-1.054735
H	1.591466	-0.112545	-1.624980
H	2.354395	-0.095141	-0.033707
H	0.971755	1.985781	-0.431954

POST_{ae}^{15c}

T_1= 0.01509806, D_1= 0.06543331

C	0.289678	-1.244464	-0.155465
N	-1.130179	-1.211594	0.160743
C	-1.838681	-0.071411	-0.196423
C	-1.208639	1.238625	0.140971

$\tilde{\nu}$: 175.3, 219.9, 266.6, 338.5, 365.6, 426.4, 467.5, 482.1, 528.8, 660.2, 731.7, 829.0, 896.4, 929.4, 977.9, 1002.9, 1071.7, 1077.4, 1128.7, 1137.8, 1179.5, 1209.3, 1250.9, 1255.1, 1307.8, 1326.6, 1360.1, 1378.0, 1395.2, 1425.7, 1451.4, 1475.4, 1489.5, 1504.3, 1514.3, 2964.2, 2968.9, 3031.4, 3079.5, 3109.3, 3111.2, 3122.7, 3205.5, 3621.1, 3857.9

B: 4.559424 2.368242 1.676367

C	0.276611	1.250517	-0.226566
C	0.969466	0.024164	0.345183
H	0.456234	-1.310144	-1.241844
H	0.746842	-2.113468	0.314490
H	-1.608154	-2.076708	-0.034216
H	-2.914462	-0.169651	-0.166962
H	-1.735452	2.045787	-0.366479
H	-1.296007	1.435498	1.220249
H	0.767798	2.151671	0.140705
H	0.381456	1.229227	-1.317298
H	0.905647	0.047329	1.435167
O	2.351994	-0.005647	0.043785
H	2.460792	0.102201	-0.905499

$CH_2CH_2CH_2NHCH_2C{=}O_e$

T_1= 0.01236929 , D_1= 0.04787050

$\tilde{\nu}$: 56.1, 148.3, 282.5, 394.1, 452.3, 489.0, 557.4, 586.4, 748.0, 753.4, 862.1, 890.6, 938.3, 967.5, 986.2, 1076.1, 1119.3, 1166.4, 1178.3, 1194.4, 1256.5, 1266.4, 1298.7, 1326.0, 1350.8, 1372.3, 1409.4, 1460.4, 1471.9, 1482.9, 1506.8, 1528.2, 1858.1, 2961.2, 2993.6, 3073.1, 3081.1, 3094.9, 3128.1, 3129.3, 3149.9, 3589.9

B: 4.614580, 2.491749, 1.803125

C	-0.306794	-1.257242	0.201598
N	0.976836	-1.093314	-0.455241
C	1.790097	-0.035184	0.140417
C	0.941889	1.236002	0.347487
C	-0.354165	1.191781	-0.467975
C	-1.151755	-0.014508	-0.033749
O	-2.339309	-0.005533	0.159710
H	-0.240547	-1.401522	1.292514
H	-0.839617	-2.113088	-0.208685
H	1.485694	-1.964472	-0.489004
H	2.616557	0.168567	-0.539878
H	2.222809	-0.336282	1.103160
H	0.680558	1.337522	1.401744
H	1.521080	2.119440	0.086038
H	-0.124208	1.078392	-1.529487
H	-0.961338	2.083814	-0.334063

$CH_2CH_2CH_2NHCH_2C{=}O_a$.

T_1= 0.01246791 , D_1= 0.04851778

$\tilde{\nu}$: 68.5, 169.1, 266.0, 390.1, 455.4, 481.1, 564.2, 600.8, 723.5, 759.9, 831.0, 890.2, 911.0, 934.4, 990.2, 1063.3, 1126.6, 1152.1, 1174.4, 1203.7, 1255.9, 1271.5, 1324.9, 1332.8, 1344.6, 1372.5, 1394.9, 1454.3, 1463.3, 1496.2, 1500.8, 1514.5, 1853.4, 3048.5, 3051.6, 3058.8, 3072.9, 3105.5, 3124.6, 3131.6, 3141.5, 3574.1

B: 4.604648, 2.470623, 1.789945

C	0.329236	-1.236078	-0.284116
N	-0.979442	-1.223334	0.345438
C	-1.802676	-0.078573	-0.054119
C	-0.992600	1.194030	-0.375754
C	0.320470	1.215733	0.405884
C	1.151182	0.012228	0.012357
O	2.351643	0.031012	-0.073849
H	0.205982	-1.303859	-1.369973
H	0.898989	-2.111532	0.022991
H	-0.873676	-1.246874	1.349842
H	-2.503336	0.118116	0.756661
H	-2.397931	-0.359749	-0.925133
H	-0.762052	1.226828	-1.442162
H	-1.585986	2.081663	-0.160823
H	0.120931	1.150428	1.480269
H	0.906357	2.116188	0.235547

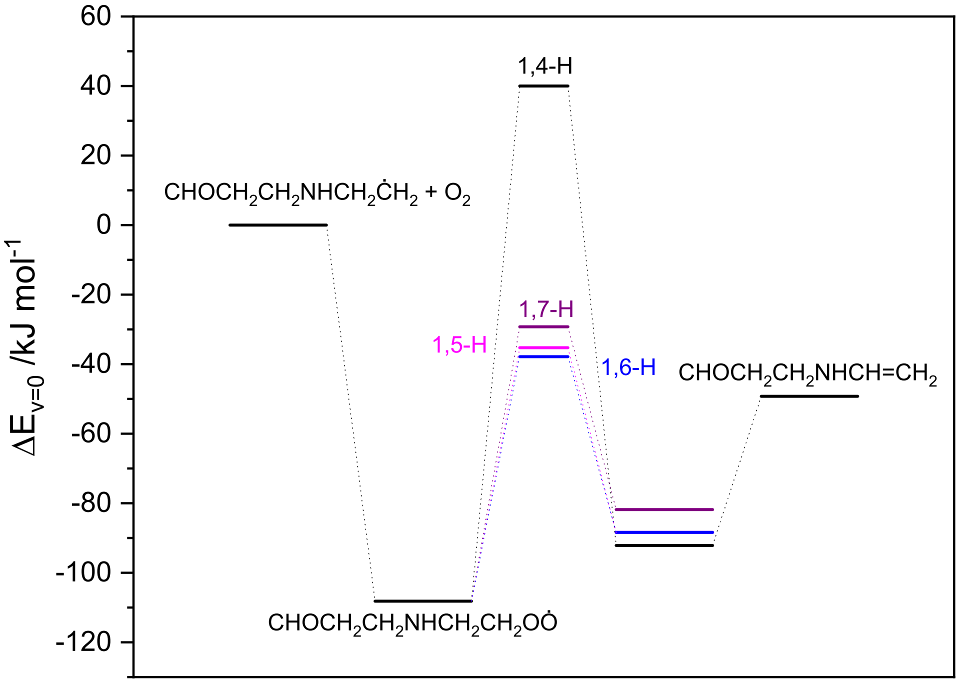

Figure S8. PES of the $CHOCH_2CH_2CH_2NH\dot{C}H_2 + O_2$ reaction.
Stationary points on the potential energy surface relevant to the internal H-transfer reactions in the $CHOCH_2CH_2CH_2NHCH_2O\dot{O}$ oxy radical. Results from CCSD(T*)-F12a/aug-cc-pVTZ//M062X/aug-cc-pVTZ calculations

Table S14. QCC results for the $CHOCH_2CH_2CH_2NHCH_2O\dot{O}$ internal H-transfer reactions
Electronic energies of reactants, intermediates and products (/Hartree), and relative energies including Zero Point Energies, Δ (/kJ mol^{-1}), of stationary points on the potential energy surface of the $CHOCH_2CH_2CH_2NH\dot{C}H_2$ + O_2 reaction.

Species	M06-2X/aTZ		CCSD(T*)-F12a/aTZ	
	E_{Elec}	E_{ZPE}	E_{Elec}	$\Delta E_{v=0}$
$CHOCH_2CH_2CH_2NH\dot{C}H_2$	-326.43143	0.14656	-326.04880	
O_2	-150.32480	0.00400	-150.19114	
Sum reactants	-476.75622	0.15055	-476.23994	0.0
$CHOCH_2CH_2CH_2NHCH_2O\dot{O}$	-476.81384	0.15789	-476.29725	-131.2
SP-18a	-476.77244	0.15130	-476.26115	-53.7
POST-18a	-476.79220	0.15316	-476.28101	-101.0
$CHOCH_2CH_2CH_2N{=}CH_2$	-325.86393	0.13617	-325.48149	
HO_2	-150.90809	0.01458	-150.77891	
Sum Products	-476.77202	0.15075	-476.26040	-53.2
SP-18b	-476.77074	0.15232	-476.25684	-39.7
POST-18b	-476.79959	0.15547	-476.28369	-102.0
SP-18c	-476.76637	0.15132	-476.25186	-29.3
POST-18c	-476.79587	0.15580	-476.27866	-87.9
SP-18d	-476.77544	0.15189	-476.25955	-48.0

POST-18d	-476.80172	0.15648	-476.28436	-101.1
SP-18e	-476.78376	0.15264	-476.26770	-67.4
POST-18e	-476.80661	0.15763	-476.29179	-117.6

Table S14, continued.
T_1 and D_1 diagnostic values, vibrational frequencies (cm^{-1}), Rotational constants (GHz) and Cartesian coordinates of the species listed above. Results from CCSD(T*)-F12a/aug-cc-pVTZ//M06-2X/aug-cc-pVTZ calculations.

$CHOCH_2CH_2CH_2NH\dot{C}H_2$	C	1.729030	-0.721154	0.377890
	O	1.534018	-1.312917	-0.650115
T_1= 0.01605691 , D_1= 0.06519344	C	1.336504	0.707930	0.637523
	C	0.445026	1.322288	-0.433195
$\tilde{\nu}$: 83.4, 116.2, 137.4, 165.8, 226.0, 301.4, 330.7, 359.1, 502.7,	C	-1.031293	1.027999	-0.208052
573.2, 651.2, 678.4, 720.9, 838.2, 879.1, 956.8, 1003.4, 1016.5,	N	-1.278244	-0.391889	-0.061895
1094.3, 1113.9, 1175.2, 1233.8, 1286.8, 1291.3, 1304.2, 1370.3,	C	-2.551557	-0.810392	0.258088
1388.0, 1402.9, 1423.5, 1447.7, 1472.2, 1486.6, 1518.5, 1535.4,	H	2.262487	-1.218950	1.208873
1850.4, 2955.8, 3009.9, 3043.7, 3053.5, 3075.6, 3086.0, 3123.9,	H	2.280258	1.255221	0.737909
3172.3, 3287.8, 3586.4	H	0.867202	0.746508	1.625075
	H	0.751336	0.942897	-1.409554
B: 3.907890 1.854091 1.450687	H	0.578239	2.403362	-0.453041
	H	-1.622998	1.455424	-1.026296
	H	-1.365810	1.511836	0.714621
	H	-0.773503	-0.968505	-0.720643
	H	-3.126146	-0.169880	0.910508
	H	-2.741762	-1.871392	0.253212
$CHOCH_2CH_2CH_2NHCH_2O\dot{O}$	C	-1.594565	-1.160223	0.061336
	H	-1.632979	-2.176880	-0.367242
T_1= 0.02357485, D_1= 0.14073442	O	-1.643083	-1.002517	1.253182
	C	-1.525046	-0.045341	-0.947781
$\tilde{\nu}$: 46.1, 102.8, 110.7, 138.0, 170.4, 188.3, 229.1, 297.8, 327.9,	H	-0.724814	-0.290134	-1.652351
415.5, 479.1, 640.9, 649.6, 668.5, 716.0, 765.7, 829.5, 878.8,	H	-2.453430	-0.122318	-1.523685
952.6, 1002.7, 1016.8, 1094.4, 1119.4, 1164.3, 1223.0, 1246.6,	C	-1.359818	1.351606	-0.358853
1265.9, 1286.0, 1320.6, 1325.4, 1372.4, 1392.4, 1402.2, 1410.6,	H	-1.915966	1.413559	0.578340
1424.5, 1450.5, 1483.9, 1494.5, 1523.3, 1537.8, 1844.5, 2981.5,	H	-1.792809	2.087102	-1.036625
3041.1, 3048.4, 3068.0, 3079.9, 3088.4, 3100.5, 3120.3, 3170.8,	C	0.093035	1.740482	-0.112372
3597.0	H	0.633843	1.697982	-1.061861
	H	0.130234	2.775173	0.236958
B: 2.067510 1.389156 1.090925	N	0.752415	0.864900	0.852481
	H	0.128403	0.236434	1.337498
	C	1.983131	0.315178	0.518617
	H	2.383741	-0.288283	1.328961
	H	2.700417	1.062481	0.182589
	O	1.951014	-0.603912	-0.666707
	O	1.154073	-1.601024	-0.455929
SP-18a	C	1.991543	-0.976320	-0.050957
	H	2.118767	-1.951158	-0.555234
T_1= 0.02568994, D_1= 0.11872348	O	2.138707	-0.882103	1.137544
	C	1.662912	0.184713	-0.952584
$\tilde{\nu}$: -902.4, 36.5, 55.7, 122.1, 140.9, 179.1, 218.4, 235.4, 261.1,	H	2.564874	0.372178	-1.544848
317.0, 377.7, 481.2, 569.5, 663.6, 677.7, 703.4, 771.7, 833.6,	H	0.914799	-0.144845	-1.677303
872.8, 948.5, 988.0, 1045.5, 1080.1, 1097.9, 1134.3, 1201.6,	C	1.239813	1.444826	-0.208543

1234.6, 1258.0, 1323.2, 1324.8, 1370.6, 1396.1, 1407.4, 1424.6, 1428.8, 1463.7, 1476.3, 1500.6, 1504.9, 1666.0, 1852.8, 1882.3, 2969.4, 3053.3, 3068.3, 3080.6, 3094.3, 3118.8, 3130.3, 3130.7, 3239.3

B: 2.217409 1.152141 0.914158

H	1.128964	2.262832	-0.921036
H	2.023414	1.721076	0.496826
C	-0.056533	1.279091	0.569569
H	0.053361	0.486564	1.313755
H	-0.296003	2.204652	1.092682
N	-1.176189	0.953902	-0.328508
H	-1.004397	-0.120335	-0.812083
C	-2.338656	0.798396	0.217907
H	-3.191460	0.570657	-0.407298
H	-2.520212	1.067956	1.254932
O	-2.061003	-1.391610	0.287675
O	-1.146860	-1.417679	-0.599368

POST-18a

T_1= 0.02232698 , D_1= 0.13315697

$\tilde{\nu}$: 24.9, 43.2, 51.7, 87.1, 104.1, 128.3, 143.4, 175.8, 185.4, 263.0, 288.9, 411.3, 518.0, 673.8, 689.1, 750.6, 795.3, 802.9, 912.7, 949.6, 1019.3, 1080.3, 1106.3, 1116.7, 1142.3, 1200.2, 1265.5, 1279.8, 1288.2, 1312.2, 1323.2, 1379.9, 1414.3, 1424.6, 1457.7, 1487.5, 1496.2, 1512.1, 1639.7, 1774.9, 1862.0, 2953.9, 3004.0, 3022.1, 3039.9, 3059.6, 3068.8, 3080.4, 3085.9, 3127.5, 3204.8

B: 2.671191 0.549150 0.485690

C	-3.506834	-0.433281	0.021226
H	-4.525251	-0.231026	0.403127
O	-3.311040	-1.343069	-0.735338
C	-2.435035	0.499870	0.514314
H	-2.740836	1.515202	0.238206
H	-2.462027	0.478821	1.609019
C	-1.053099	0.165955	-0.017663
H	-1.070140	0.157697	-1.108048
H	-0.772151	-0.841488	0.291378
C	-0.010928	1.162050	0.472192
H	-0.256324	2.174289	0.129457
H	0.001104	1.170670	1.564512
N	1.314293	0.768894	0.024529
H	2.111803	-0.692740	0.415883
C	1.964016	1.538849	-0.731841
H	2.948495	1.233910	-1.070648
H	1.566836	2.505061	-1.054857
O	3.815335	-1.020953	-0.165813
O	2.776919	-1.442641	0.508765

$CHOCH_2CH_2CH_2N{=}CH_2$

T_1= 0.01309743, D_1= 0.04745228

$\tilde{\nu}$: 69.1, 87.0, 97.6, 201.9, 296.8, 313.4, 406.9, 500.4, 556.0, 708.7, 745.9, 829.5, 920.0, 958.1, 997.5, 1086.3, 1090.4, 1093.7, 1139.4, 1201.4, 1229.8, 1255.3, 1295.6, 1350.9, 1383.8, 1387.3, 1427.1, 1452.9, 1488.8, 1489.6, 1507.6, 1779.4, 1860.2, 2943.2, 3011.3, 3030.6, 3049.2, 3054.6, 3079.0, 3109.5, 3118.3, 3168.2

B: 3.932819 1.830094 1.347724

C	1.543670	-0.512902	-0.343514
O	2.224466	-1.282815	0.274395
C	1.289586	0.913606	0.079977
C	-0.065250	1.465317	-0.350403
C	-1.223726	0.835353	0.413379
N	-1.238960	-0.596548	0.185642
C	-2.314591	-1.135089	-0.177826
H	1.103182	-0.802958	-1.316912
H	2.084147	1.503099	-0.388896
H	1.434501	0.980151	1.158746
H	-0.209428	1.295193	-1.420878
H	-0.087411	2.544291	-0.196287
H	-2.175171	1.305610	0.136377
H	-1.066012	0.992436	1.483602
H	-2.342009	-2.206207	-0.358196
H	-3.242938	-0.570964	-0.321886

HO_2

T_1= 0.03589638, D_1= 0.12282697

$\tilde{\nu}$: 12528, 1459., 3686.8

B: 628.5431720, 34.6992321, 32.8838526

H	-0.880747	-0.865418	0.000000
O	0.055047	0.708193	0.000000
O	0.055047	-0.600015	0.000000

SP-17b

T_1= 0.01957312, D_1= 0.10313253

C	3.005573	0.379184	0.002903
H	3.762301	1.074198	0.413181
O	3.259525	-0.295730	-0.956592

$\tilde{\nu}$: -1792.7, 38.8, 49.6, 105.9, 178.8, 182.7, 214.8, 285.5, 363.8, 406.1, 539.6, 563.8, 669.3, 690.8, 706.1, 792.3, 839.1, 919.4, 946.7, 965.9, 991.4, 1058.4, 1067.6, 1098.3, 1122.6, 1160.1, 1217.4, 1225.5, 1270.6, 1288.8, 1290.7, 1318.0, 1378.2, 1404.7, 1422.3, 1446.1, 1457.0, 1477.8, 1503.4, 1511.6, 1651.2, 1858.5, 2950.6, 3039.6, 3060.2, 3073.2, 3074.3, 3120.0, 3130.7, 3135.8, 3596.6

B: 3.151312 0.791277 0.764946

C	1.689437	0.358880	0.726660
H	1.897668	0.102243	1.771560
H	1.308089	1.385177	0.749723
C	0.674097	-0.583624	0.106173
H	1.096950	-1.588442	0.025719
H	0.459844	-0.257872	-0.911310
C	-0.597844	-0.638056	0.915353
H	-0.476488	-1.025861	1.924561
H	-1.024882	0.597968	1.009495
N	-1.723082	-1.202821	0.272621
H	-2.458355	-1.382717	0.940304
C	-2.233331	-0.427907	-0.847473
H	-3.323421	-0.452116	-0.812666
H	-1.876248	-0.807446	-1.804298
O	-1.753732	0.907794	-0.827940
O	-1.822229	1.418406	0.454993

POST-18b

T_1= 0.01542640, D_1= 0.06112470

$\tilde{\nu}$: 35.7, 44.4, 92.3, 105.7, 142.7, 185.7, 189.5, 254.2, 333.0, 347.1, 382.3, 452.0, 571.7, 616.9, 666.8, 674.1, 728.8, 867.8, 915.5, 940.4, 1009.2, 1064.4, 1075.8, 1090.4, 1128.3, 1177.2, 1206.0, 1260.4, 1274.0, 1301.6, 1331.6, 1374.7, 1409.7, 1415.8, 1423.5, 1431.0, 1455.7, 1482.3, 1491.5, 1545.5, 1857.8, 2948.7, 3003.4, 3042.6, 3054.5, 3075.8, 3078.4, 3135.8, 3183.5, 3598.5, 3768.8

B: 4.442957 0.542630 0.525158

C	-3.506855	0.458148	0.153622
H	-4.222768	1.122702	0.673609
O	-3.875931	-0.238195	-0.752000
C	-2.093535	0.512229	0.658113
H	-1.752560	1.549410	0.569624
H	-2.119480	0.310296	1.734112
C	-1.153921	-0.439344	-0.061750
H	-1.190545	-0.233422	-1.135754
H	-1.533843	-1.463416	0.046582
C	0.242990	-0.322638	0.438041
H	1.988898	1.586094	-0.647371
H	0.433046	-0.210292	1.498533
N	1.244286	-0.969701	-0.270218
H	1.135178	-0.954099	-1.273329
C	2.595410	-0.856622	0.178738
H	3.252098	-1.304555	-0.566196
H	2.723063	-1.346498	1.145555
O	3.006060	0.472782	0.453353
O	2.878919	1.218044	-0.745656

SP-18c

T_1= 0.01891490, D_1= 0.09994212

$\tilde{\nu}$: -1948.5, 38.5, 53.8, 108.7, 165.2, 210.7, 293.1, 312.6, 356.0, 410.4, 452.8, 573.9, 677.5, 686.6, 708.2, 761.9, 856.4, 905.3, 939.5, 948.8, 987.3, 1047.5, 1079.3, 1102.8, 1113.6, 1151.8, 1168.6, 1190.5, 1240.6, 1266.1, 1294.5, 1338.8, 1367.4, 1403.3, 1409.5, 1420.7, 1438.6, 1487.3, 1500.9, 1508.7, 1512.7, 1861.4, 2959.4, 3001.7, 3046.5, 3052.7, 3078.2, 3098.3, 3129.5, 3135.2, 3570.8

B: 3.051319 0.939525 0.825192

C	2.794173	-0.138916	0.338251
H	3.544559	-0.295550	1.134909
O	3.008076	-0.508650	-0.782283
C	1.533643	0.563601	0.768114
H	1.132509	0.042122	1.644779
H	1.829667	1.556155	1.138238
C	0.495340	0.666616	-0.306684
H	0.049908	-0.576306	-0.454269
H	0.895806	0.857010	-1.300982
C	-0.768831	1.437730	0.031776
H	-0.922745	1.448923	1.112196
H	-0.659349	2.478085	-0.285864
N	-1.973390	0.877857	-0.579822
H	-1.799998	0.641173	-1.547545
C	-2.506902	-0.245279	0.124605
H	-3.166223	-0.808633	-0.534852
H	-3.042968	0.075028	1.019194
O	-1.509155	-1.122043	0.673199
O	-0.665417	-1.527496	-0.341343

POPST-18c

T_1= 0.01426799 , D_1= 0.04814166

$\tilde{\nu}$: 67.6, 79.2, 116.7, 157.7, 180.3, 206.0, 241.6, 286.5, 345.5, 372.7, 436.7, 493.8, 501.3, 625.1, 673.9, 709.7, 732.3, 858.6, 921.6, 933.4, 969.3, 1005.4, 1067.0, 1069.7, 1150.4, 1165.4, 1186.2, 1238.2, 1275.1, 1314.4, 1351.4, 1363.5, 1400.1, 1412.6, 1420.2, 1452.5, 1472.8, 1478.6, 1487.9, 1509.6, 1841.4, 2969.9, 2986.1, 3051.4, 3064.6, 3070.9, 3093.9, 3125.6, 3194.9, 3570.0, 3688.6

B: 2.173109 1.394806 1.001524

C	2.282364	-0.033240	0.341709
H	3.082122	-0.145395	1.095193
O	2.197457	-0.822174	-0.562918
C	1.378853	1.154008	0.541585
H	0.913992	1.025693	1.524753
H	2.052875	2.017460	0.653758
C	0.364288	1.363333	-0.519052
H	0.434462	-1.532069	-0.830168
H	0.669952	1.230845	-1.548376
C	-1.073680	1.645896	-0.227811
H	-1.183399	2.014263	0.795424
H	-1.439196	2.433051	-0.889878
N	-1.988930	0.508358	-0.371822
H	-1.954303	0.123069	-1.306815
C	-1.863023	-0.524766	0.598129
H	-2.618579	-1.286486	0.404529
H	-1.995301	-0.112324	1.600517
O	-0.594442	-1.167909	0.686171
O	-0.424631	-1.879668	-0.536196

SP-18d

T_1= 0.01979941, D_1= 0.10648214

$\tilde{\nu}$: -1982.4, 66.6, 101.6, 162.4, 196.5, 218.7, 279.1, 300.7, 363.7, 437.5, 474.3, 565.5, 621.9, 667.8, 720.1, 816.3, 834.5, 867.7, 898.0, 944.6, 987.4, 1020.7, 1039.4, 1079.7, 1110.6, 1136.7, 1154.9, 1217.2, 1258.3, 1297.3, 1329.6, 1372.7, 1382.1, 1397.2, 1409.8, 1426.6, 1446.4, 1462.0, 1493.4, 1514.6, 1523.5, 1785.3, 2984.9, 3037.9, 3067.1, 3089.0, 3089.9, 3130.0, 3138.2, 3147.9, 3602.5

B: 2.154733 1.408171 1.141788

C	-1.913672	-0.780828	-0.127027
H	-2.230762	-1.805886	0.130268
O	-2.098333	-0.337871	-1.235425
C	-1.246335	-0.026461	0.950713
H	-1.661547	-0.230448	1.936351
H	-0.138331	-0.783720	1.058764
C	-0.845091	1.393916	0.661819
H	-0.528022	1.881240	1.585848
H	-1.731267	1.931565	0.307951
C	0.252576	1.569731	-0.395070
H	-0.044911	1.070476	-1.315789
H	0.338406	2.635934	-0.611852
N	1.552532	1.054125	0.018107
H	1.698541	1.092608	1.015538
C	2.012248	-0.152525	-0.547681
H	2.968387	-0.420731	-0.100679
H	2.100960	-0.079685	-1.631247
O	1.122113	-1.290151	-0.403044
O	0.826528	-1.458631	0.918666

POST-18d

T_1= 0.01678675, D_1= 0.07819733

$\tilde{\nu}$: 37.5, 58.3, 85.1, 137.1, 168.2, 200.0, 206.1, 286.2, 314.0, 364.8, 438.6, 491.1, 573.7, 639.2, 655.7, 711.1, 850.2, 878.4, 904.9, 971.4, 988.9, 1009.4, 1029.9, 1051.1, 1137.0, 1150.1, 1211.8, 1255.9, 1283.4, 1322.8, 1363.8, 1382.4, 1395.0, 1406.1, 1410.2, 1451.8, 1471.8, 1477.5, 1496.1, 1515.8, 1639.2, 3004.1, 3056.1, 3078.8, 3080.6, 3112.3, 3129.8, 3136.4, 3233.7, 3604.0, 3828.4

B: 2.827686 0.925191 0.832762

C	-2.394782	-0.910628	-0.324107
H	-2.641759	-1.855567	-0.832301
O	-3.133877	0.061876	-0.434278
C	-1.190574	-0.887789	0.447164
H	-0.586657	-1.781719	0.487227
H	2.386088	-0.840218	1.372416
C	-0.780322	0.350091	1.151712
H	-0.013753	0.141612	1.898762
H	-1.658565	0.753707	1.661543
C	-0.278899	1.468963	0.196435
H	-0.949676	1.504273	-0.662860
H	-0.378756	2.421378	0.714907
N	1.085311	1.382294	-0.278460
H	1.779121	1.657187	0.400422
C	1.488365	0.267843	-1.050170

H	2.441246	0.475878	-1.535554
H	0.725774	0.033711	-1.795439
O	1.635094	-0.972222	-0.343079
O	2.778411	-0.829301	0.491594

SP-18e

T_1= 0.02858206, D_1= 0.17773742

$\tilde{\nu}$: -1731.9, 85.5, 95.8, 132.4, 234.0, 248.1, 269.9, 300.4, 388.1, 409.1, 473.1, 492.6, 528.9, 622.0, 659.7, 842.4, 879.6, 886.0, 929.7, 961.9, 994.2, 1058.4, 1079.3, 1086.9, 1119.7, 1163.7, 1198.2, 1220.9, 1242.6, 1305.8, 1331.1, 1343.8, 1375.5, 1402.5, 1412.0, 1469.8, 1481.7, 1488.8, 1495.0, 1507.2, 1508.7, 1908.7, 3042.4, 3045.8, 3067.7, 3080.7, 3091.5, 3115.4, 3131.4, 3140.0, 3651.8

B: 1.985896 1.506436 1.065964

C	-1.014493	-1.174237	0.059528
H	0.179400	-1.529203	-0.078488
O	-1.688030	-1.620146	0.926546
C	-1.451699	-0.135680	-0.955466
H	-0.721021	-0.087613	-1.762126
H	-2.407547	-0.480372	-1.351396
C	-1.608402	1.230447	-0.280310
H	-2.213416	1.111739	0.620614
H	-2.144294	1.909570	-0.944986
C	-0.248045	1.826938	0.069736
H	0.284597	2.048435	-0.857963
H	-0.380891	2.773152	0.600873
N	0.568875	0.894930	0.826763
H	0.475460	0.933127	1.826992
C	1.871517	0.580568	0.347970
H	2.469197	0.154181	1.152060
H	2.386393	1.434061	-0.104288
O	1.813821	-0.353003	-0.728299
O	1.473801	-1.589327	-0.240421

POST-18e

T_1= 0.01590324, D_1= 0.06373122

$\tilde{\nu}$: 46.4, 59.5, 94.3, 107.5, 195.8, 219.1, 265.6, 306.1, 321.7, 431.2, 451.9, 506.1, 575.5, 586.1, 682.5, 813.3, 877.6, 882.0, 904.7, 951.2, 988.8, 1060.8, 1077.3, 1097.4, 1151.8, 1165.4, 1190.5, 1245.4, 1300.1, 1319.3, 1334.9, 1373.7, 1405.4, 1411.3, 1442.0, 1466.0, 1481.5, 1491.3, 1497.3, 1511.1, 1964.2, 3023.9, 3039.2, 3069.3, 3074.8, 3093.0, 3116.5, 3118.9, 3136.1, 3606.6, 3657.4

B: 2.127019 1.219075 0.946310

C	-1.330643	-1.243159	-0.039096
H	0.905503	-1.535689	-0.321786
O	-1.897609	-1.555656	0.941496
C	-1.683885	-0.106022	-0.982977
H	-0.983633	-0.097294	-1.816512
H	-2.678761	-0.347322	-1.363134
C	-1.678612	1.236737	-0.249220
H	-2.244753	1.141219	0.679300
H	-2.181393	1.986910	-0.860749
C	-0.253542	1.691942	0.036337
H	0.263015	1.833749	-0.915048
H	-0.262809	2.658937	0.551048
N	0.485202	0.676522	0.769076
H	0.246410	0.623598	1.747665
C	1.895598	0.600072	0.516730
H	2.396254	0.143343	1.370333
H	2.348164	1.570776	0.287367
O	2.156953	-0.154644	-0.644924
O	1.878418	-1.513613	-0.350654

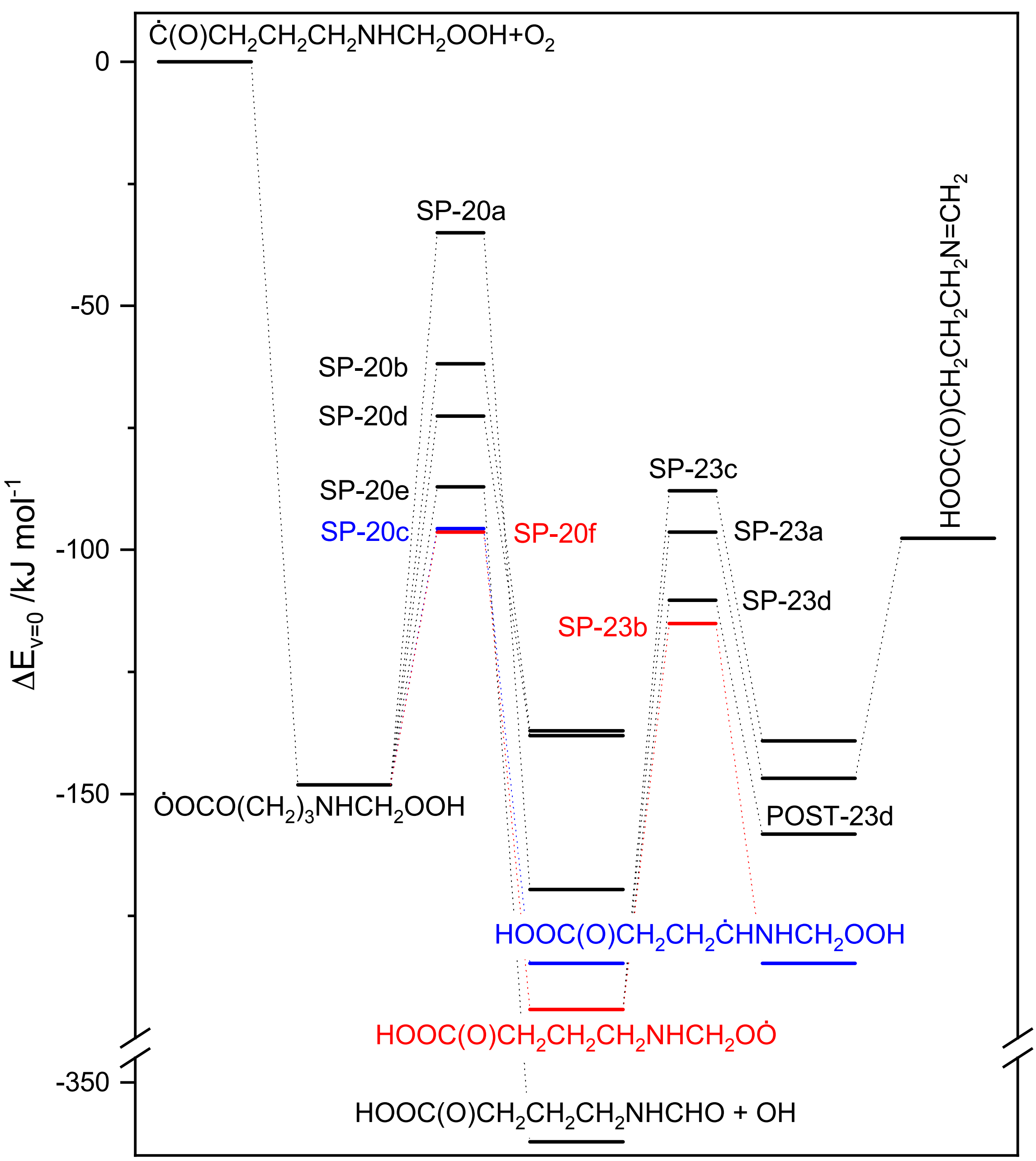

Figure S9. PES of the C(O)CH$_2$CH$_2$CH$_2$NHCH$_2$OOH + O$_2$ reaction.
Stationary points on the potential energy surface relevant to the internal H-transfer reactions in the ȮOC(O)CH$_2$CH$_2$CH$_2$NHCH$_2$OOH oxy radical. Results from CCSD(T*)-F12a/aug-cc-pVTZ//M062X/aug-cc-pVTZ calculations

Table S15. QCC results for the $\dot{O}OC(O)CH_2CH_2CH_2NHCH_2OOH$ internal H-transfer reactions
Electronic energies of reactants, intermediates and products (/Hartree), and relative energies including Zero Point Energies, Δ (/kJ mol^{-1}), of stationary points on the potential energy surface of the $\dot{C}(O)CH_2CH_2CH_2NHCH_2OOH + O_2$ reaction.

Species	M06-2X/aTZ		
	E_{Elec}	E_{ZPE}	$\Delta E_{v=0}$
$\dot{C}(O)CH_2CH_2CH_2NHCH_2OOH$	-476.80661	0.15763	
O_2	-150.32480	0.00400	
Sum reactants	-627.13141	0.16162	0.0
$\dot{O}OCOCH_2CH_2CH_2NHCH_2OOH$	-627.19289	0.16669	-148.1
SP-21a	-627.14543	0.16230	-35.0
$HOOC(O)\dot{C}HCH_2CH_2NHCH_2OOH$	-627.20103	0.16664	-169.6
SP-21b	-627.15449	0.16115	-61.9
$HOOC(O)CH_2\dot{C}HCH_2NHCH_2OOH$	-627.18808	0.16607	-137.1
SP-21c	-627.16871	0.16254	-95.6
$HOOC(O)CH_2CH_2\dot{C}HNHCH_2OOH$	-627.20645	0.16629	-184.7
SP-21d	-627.15830	0.16087	-72.6
$HOOC(O)CH_2CH_2CH_2\dot{N}CH_2OOH$	-627.18769	0.16531	-138.1
SP-21e	-627.16637	0.16341	-87.1
$HOOC(O)CH_2CH_2CH_2NHCHO$	-551.53497	0.15247	
OH	-75.73381	0.00859	
Sum products	-627.26878	0.16106	-362.2
SP-21f	-627.16868	0.16218	-96.4
$HOOC(O)CH_2CH_2CH_2NHCH_2O\dot{O}$	-627.21144	0.16768	-194.2
SP-24a	-627.17031	0.16380	-96.4
POST-24a	-627.18949	0.16377	-146.8
$HOOC(O)CH_2CH_2CH_2N{=}CH_2$	-476.26060	0.14717	
HO_2	-150.90809	0.01458	
Sum products	-627.16870	0.16175	-97.6
SP-24b	-627.17670	0.16306	-115.1
$HOOC(O)CH_2CH_2\dot{C}HNHCH_2OOH$	-627.20645	0.16629	-184.7
SP-23c	-627.16502	0.16176	-87.9
$HOOC(O)CH_2\dot{C}HCH_2NHCH_2OOH$	-627.18820	0.16544	-139.1
SP-24d	-627.17336	0.16156	-110.3
$HOOC(O)\dot{C}HCH_2CH_2NHCH_2OOH$	-627.19592	0.16588	-158.2

Table S15, continued.
Vibrational frequencies (cm^{-1}), Rotational constants (GHz) and Cartesian coordinates of the species listed above. Results from M06-2X/aug-cc-pVTZ calculations.

$\dot{C}(O)CH_2CH_2CH_2NHCH_2OOH$

$\tilde{\nu}$: 46.4, 59.5, 94.3, 107.5, 195.8, 219.1, 265.6, 306.1, 321.7, 431.2, 451.9, 506.1, 575.5, 586.1, 682.5, 813.3, 877.6, 882.0, 904.7, 951.2, 988.8, 1060.8, 1077.3, 1097.4, 1151.8, 1165.4, 1190.5, 1245.4, 1300.1, 1319.3, 1334.9, 1373.7, 1405.4, 1411.3, 1442.0, 1466.0, 1481.5, 1491.3, 1497.3, 1511.1, 1964.2, 3023.9, 3039.2, 3069.3, 3074.8, 3093.0, 3116.5, 3118.9, 3136.1, 3606.6, 3657.4

B: 2.127019 1.219075 0.946310

C	-1.330643	-1.243159	-0.039096
H	0.905503	-1.535689	-0.321786
O	-1.897609	-1.555656	0.941496
C	-1.683885	-0.106022	-0.982977
H	-0.983633	-0.097294	-1.816512
H	-2.678761	-0.347322	-1.363134
C	-1.678612	1.236737	-0.249220
H	-2.244753	1.141219	0.679300
H	-2.181393	1.986910	-0.860749
C	-0.253542	1.691942	0.036337
H	0.263015	1.833749	-0.915048
H	-0.262809	2.658937	0.551048
N	0.485202	0.676522	0.769076
H	0.246410	0.623598	1.747665
C	1.895598	0.600072	0.516730
H	2.396254	0.143343	1.370333
H	2.348164	1.570776	0.287367
O	2.156953	-0.154644	-0.644924
O	1.878418	-1.513613	-0.350654

O_2
$\tilde{\nu}$: 1754.5, B: 0.0000000 44.6355338 44.6355338

O	0.000000	0.000000	0.594925
O	0.000000	0.000000	-0.594925

$\dot{O}OCOCH_2CH_2CH_2NHCH_2OOH$

$\tilde{\nu}$: 28.2, 47.8, 64.7, 107.4, 110.5, 134.3, 183.8, 218.2, 256.5, 280.7, 304.0, 336.9, 412.4, 453.1, 511.7, 571.7, 658.1, 697.3, 771.0, 829.4, 881.3, 903.2, 925.9, 939.4, 1006.8, 1058.1, 1062.5, 1080.1, 1096.6, 1165.4, 1205.4, 1224.9, 1235.3, 1263.0, 1298.0, 1335.0, 1375.8, 1382.9, 1393.3, 1404.7, 1414.9, 1467.7, 1483.5, 1488.3, 1515.3, 1522.9, 1940.5, 3040.0, 3070.7, 3073.0, 3078.0, 3085.3, 3126.6, 3129.3, 3136.6, 3546.2, 3832.6

B: 1.676878 0.591907 0.508667

H	-2.015610	-0.135032	-1.474759
C	-1.660460	1.399484	-0.402493
H	-2.533251	2.031887	-0.226475
C	-0.398348	2.061464	0.146638
H	-0.430698	2.033088	1.237289
H	-0.376983	3.109927	-0.147757
C	0.859961	1.371869	-0.360539
H	1.747341	1.836998	0.078670
H	0.933975	1.498961	-1.444370
N	0.793266	-0.060339	-0.099445
H	0.760715	-0.248773	0.896153
C	1.813926	-0.833291	-0.719395
H	1.754871	-0.737352	-1.804955
H	1.704668	-1.877452	-0.424507
O	3.146138	-0.403342	-0.446090
O	3.346524	-0.554657	0.955239
H	4.009834	-1.254360	0.980122
C	-1.939748	0.102460	0.294147
O	-1.883495	-0.143205	1.447273
O	-2.386852	-0.858962	-0.661821
O	1.676878	-2.015610	-0.135032

SP-21a

$\tilde{\nu}$: -947.2, 42.6, 98.9, 105.8, 113.8, 155.5, 176.9, 209.0, 218.2, 242.1, 292.6, 341.7, 422.2, 495.4, 530.6, 573.0, 603.1, 651.2, 697.5, 767.4, 861.4, 894.6, 924.8, 954.4, 974.4, 1025.1, 1052.7, 1103.9, 1123.4, 1144.2, 1147.1, 1210.9, 1230.5, 1266.3, 1288.6, 1328.6, 1337.4, 1351.1, 1364.3, 1389.2, 1395.7, 1422.6, 1439.1,

H	-1.251931	-0.577344	0.014721
C	-1.773562	0.949708	0.506990
H	-2.459654	1.422138	1.198928
C	-1.301019	1.727834	-0.711069
H	-1.004661	2.751532	-0.469703
H	-2.057072	1.775287	-1.492425
C	-0.099232	0.919788	-1.204967
H	0.623488	1.489085	-1.782982

1496.0, 1505.0, 1518.5, 1604.3, 1915.6, 3054.8, 3114.1, 3119.6, 3126.5, 3181.8, 3189.4, 3203.2, 3428.7, 3816.4

B: 1.601641 0.737117 0.712906

H	-0.424293	0.045484	-1.762137
N	0.531601	0.418281	0.042806
H	1.167625	1.122617	0.426346
C	1.316776	-0.828318	-0.051329
H	0.773851	-1.539773	-0.665300
H	1.454786	-1.194915	0.965063
O	2.530205	-0.576840	-0.696477
O	3.269812	0.309434	0.136626
H	3.947515	-0.273995	0.502005
C	-0.653791	0.337637	1.136696
O	-0.358648	-0.140219	2.184638
O	-1.390135	-1.723114	-0.458954
O	1.601641	-1.251931	-0.577344

$HOOC(O)\dot{C}HCH_2CH_2NHCH_2OOH$

$\tilde{\nu}$: 27.3, 55.0, 75.4, 127.7, 157.3, 171.7, 201.9, 236.3, 293.3, 325.1, 385.4, 419.6, 429.0, 466.9, 491.0, 542.9, 599.1, 626.1, 673.2, 716.4, 769.9, 838.9, 889.2, 935.7, 962.8, 1006.3, 1019.3, 1072.1, 1076.1, 1125.9, 1157.7, 1187.8, 1227.1, 1252.0, 1280.6, 1327.1, 1367.4, 1387.7, 1408.5, 1455.1, 1470.6, 1476.0, 1492.1, 1502.1, 1510.6, 1513.8, 1745.6, 3038.0, 3059.2, 3079.6, 3108.8, 3136.5, 3149.7, 3211.5, 3583.7, 3595.0, 3704.7

B: 1.760800 0.646201 0.526961

C	-1.653633	-0.373789	-0.182719
O	1.383593	-0.870827	-0.654483
O	2.833310	0.439433	0.433954
O	3.635801	-0.716376	0.457265
H	3.075897	-1.364230	-0.016682
C	0.834071	1.400823	-0.195050
H	1.215094	2.268219	0.326803
C	-0.469059	1.450487	-0.886154
H	-0.639935	0.539229	-1.455314
H	-0.458835	2.296204	-1.581838
C	-1.641665	1.683634	0.100451
H	-1.424324	2.574339	0.693676
H	-2.536012	1.900246	-0.485268
N	-1.957106	0.590653	0.990917
H	-1.173419	0.270001	1.542669
C	-2.731714	-0.483858	0.467223
H	-3.638783	-0.087585	0.007758
H	-2.988461	-1.166927	1.276092
O	-2.143335	-1.239100	-0.583703
O	-1.121079	-2.030807	0.010338
H	1.760800	-1.653633	-0.373789

SP-21b

$\tilde{\nu}$: -1722.5, 65.8, 70.4, 112.7, 123.5, 161.6, 213.3, 231.0, 277.3, 341.7, 391.9, 396.7, 481.3, 532.9, 537.7, 564.1, 585.4, 615.0, 645.2, 692.2, 795.0, 872.6, 895.5, 934.2, 959.2, 1017.2, 1044.9, 1077.6, 1089.4, 1105.0, 1148.5, 1206.6, 1213.0, 1229.0, 1251.9, 1286.9, 1313.3, 1349.5, 1370.4, 1385.5, 1422.5, 1443.0, 1462.7, 1475.4, 1491.6, 1515.1, 1561.4, 1867.2, 3016.6, 3052.4, 3059.8, 3079.9, 3112.9, 3134.1, 3145.1, 3608.5, 3702.4

B: 1.592905 0.869044 0.714095

H	0.292048	-0.809997	1.914430
C	1.146192	-0.151317	1.281853
H	2.057770	-0.411438	1.821605
C	1.192797	1.289499	0.840827
H	1.992245	1.116248	-0.154311
H	1.764837	1.950897	1.486874
C	-0.109921	1.864350	0.344904
H	0.050978	2.879661	-0.033140
H	-0.777489	1.951772	1.209614
N	-0.747138	0.983163	-0.609045
H	-0.338846	1.013461	-1.530578
C	-2.169870	0.823364	-0.600324
H	-2.636352	1.133836	-1.537125
H	-2.595313	1.393987	0.228165
O	-2.577988	-0.520601	-0.487194
O	-2.185237	-0.977833	0.799055
H	-1.430133	-1.548134	0.577554
C	1.039404	-1.096134	0.106824
O	0.379834	-2.090593	0.055742
O	1.758870	-0.750526	-1.003141

O	1.592905	0.292048	-0.809997

HOOC(O)$CH_2\dot{C}HCH_2NHCH_2OOH$

$\tilde{\nu}$: 29.0, 54.6, 79.0, 126.5, 146.5, 160.8, 212.7, 235.9, 263.8, 290.2, 348.3, 399.8, 426.4, 444.2, 461.1, 499.7, 546.2, 645.2, 653.7, 772.6, 796.6, 890.0, 914.7, 941.1, 957.1, 979.8, 1037.6, 1052.4, 1072.3, 1134.4, 1164.1, 1200.2, 1211.7, 1259.2, 1278.2, 1314.4, 1349.6, 1384.5, 1401.1, 1407.4, 1451.4, 1471.9, 1484.7, 1497.6, 1507.4, 1522.4, 1804.8, 2939.1, 3063.4, 3072.7, 3081.6, 3139.2, 3158.1, 3219.7, 3565.0, 3624.0, 3751.5

B: 1.759378 0.648940 0.511486

C	-1.692970	0.114335	0.291630
O	0.917962	-0.933212	0.209174
O	2.936787	-0.026878	-0.009104
O	3.333739	-1.312861	-0.434663
H	2.500473	-1.819436	-0.366048
C	1.217632	1.431082	0.676562
H	0.701923	1.352833	1.637293
H	2.101204	2.049194	0.808438
C	0.315469	1.986318	-0.380826
H	0.747455	2.659648	-1.105758
C	-0.983163	1.340607	-0.705308
H	-1.606383	2.046083	-1.259608
H	-0.802646	0.491639	-1.387368
N	-1.722783	0.919304	0.480635
H	-1.131443	0.390014	1.109598
C	-2.878328	0.130585	0.176518
H	-3.549826	0.686715	-0.479246
H	-3.385120	-0.136688	1.103129
O	-2.607070	-1.055733	-0.557486
O	-1.883448	-1.921467	0.305745
H	1.759378	-1.692970	0.114335

SP-21c

$\tilde{\nu}$: -1188.3, 25.6, 74.0, 102.8, 146.3, 163.8, 206.5, 249.9, 266.0, 311.2, 359.1, 389.4, 411.7, 481.5, 503.8, 591.8, 616.2, 684.5, 699.9, 730.9, 787.7, 888.3, 933.7, 945.2, 964.5, 1044.9, 1059.2, 1085.6, 1100.0, 1143.3, 1163.0, 1199.6, 1239.9, 1265.5, 1295.3, 1307.8, 1345.2, 1365.6, 1371.0, 1407.4, 1428.2, 1449.0, 1473.4, 1486.1, 1490.0, 1516.1, 1551.0, 1895.4, 3074.1, 3086.9, 3095.9, 3098.6, 3126.1, 3144.9, 3166.1, 3579.3, 3756.0

B: 2.056871 0.665514 0.557233

H	-1.239990	-0.339688	1.535406
C	1.595162	0.747381	0.855054
H	2.328043	1.297264	1.437933
C	0.886369	1.691651	-0.138801
H	1.557081	1.902721	-0.971077
H	0.686633	2.641310	0.360321
C	-0.420824	1.133928	-0.667379
H	-0.177986	-0.042759	-0.842753
H	-0.713443	1.509341	-1.648955
N	-1.487507	1.224758	0.248992
H	-1.226015	1.018577	1.204060
C	-2.730693	0.611221	-0.117868
H	-3.090760	1.034379	-1.056115
H	-3.454171	0.778311	0.679056
O	-2.636770	-0.769873	-0.395417
O	-2.305302	-1.416809	0.827270
H	-1.411271	-1.737720	0.634749
C	2.376738	-0.321015	0.134060
O	3.564057	-0.382885	0.070698
O	1.662940	-1.245679	-0.596108
O	2.056871	-1.239990	-0.339688

HOOC(O)$CH_2CH_2\dot{C}HNHCH_2OOH$

$\tilde{\nu}$: 64.4, 81.2, 119.9, 131.7, 160.9, 193.7, 199.3, 243.5, 251.8, 289.3, 331.6, 374.9, 421.6, 440.9, 497.2, 503.1, 575.9, 629.3, 642.7, 693.2, 721.2, 876.9, 897.4, 918.0, 956.2, 966.5, 1040.5, 1057.4, 1061.3, 1108.6, 1152.9, 1190.1, 1220.3, 1265.3, 1270.1, 1315.0, 1345.8, 1378.0, 1394.5, 1420.6, 1475.7, 1482.0, 1486.4, 1491.1, 1512.5, 1554.0, 1843.9, 3063.7, 3090.2, 3098.1, 3108.4, 3155.6, 3159.1, 3227.0, 3582.7, 3603.0, 3658.1

C	0.803769	1.242865	0.295085
O	-0.552591	0.855409	1.321144
O	-0.892038	1.554739	-0.798878
O	0.218149	2.404985	-0.612424
H	0.957054	1.788546	-0.773696
C	-2.329673	-0.097530	0.016006
H	-3.124356	0.112064	0.732573
H	-2.702310	0.077066	-0.990421
C	-1.816179	-1.551347	0.166680
H	-2.605589	-2.221173	-0.177098

B: 1.286823 1.138651 0.747633

H	-1.664079	-1.753086	1.228530
C	-0.544373	-1.788814	-0.566911
H	-0.497870	-1.807123	-1.645148
N	0.668816	-1.836864	0.107296
H	0.613601	-1.735761	1.110547
C	1.839052	-1.266935	-0.468181
H	1.872877	-1.493141	-1.533313
H	2.726951	-1.643342	0.035028
O	1.890218	0.159477	-0.417905
O	2.165291	0.526437	0.928437
H	1.286823	0.803769	1.242865

SP-21d

$\tilde{\nu}$: -2211.0, 72.4, 96.8, 107.1, 137.1, 156.8, 198.9, 228.3, 272.0, 299.2, 329.7, 374.7, 427.2, 480.5, 505.4, 558.4, 596.9, 679.6, 723.0, 759.2, 851.2, 891.7, 921.3, 937.6, 989.5, 1051.2, 1059.8, 1075.6, 1101.9, 1114.1, 1163.3, 1172.3, 1244.1, 1275.1, 1303.2, 1323.1, 1360.6, 1372.3, 1377.2, 1381.1, 1384.3, 1419.7, 1442.6, 1447.1, 1481.9, 1483.9, 1497.3, 1879.0, 3027.9, 3048.1, 3085.5, 3100.5, 3101.4, 3108.6, 3157.3, 3165.8, 3812.4

B: 1.460716 0.952961 0.879939

H	0.665571	1.793018	-0.069534
C	1.479503	1.007496	-0.748851
H	2.222606	1.052444	-1.541437
C	0.082563	1.078476	-1.392275
H	-0.176531	0.116825	-1.826512
H	0.139920	1.795271	-2.212357
C	-1.052853	1.592105	-0.486586
H	-1.966824	1.617265	-1.086421
H	-0.811517	2.611548	-0.181634
N	-1.346178	0.857825	0.724835
H	-0.393429	0.774631	1.412908
C	-2.120031	-0.340056	0.631895
H	-3.179848	-0.057068	0.636551
H	-1.906761	-0.960153	1.504827
O	-2.000970	-1.095167	-0.551686
O	-0.703902	-1.673517	-0.559972
H	-0.874883	-2.589425	-0.304356
C	1.761294	-0.303309	-0.054571
O	2.311034	-1.233428	-0.560798
O	1.344617	-0.465565	1.241494
O	1.460716	0.665571	1.793018

HOOC(O)$CH_2CH_2CH_2\dot{N}CH_2$OOH

$\tilde{\nu}$: 49.0, 57.8, 69.6, 112.8, 134.9, 151.0, 190.6, 245.9, 265.3, 329.1, 342.3, 394.3, 433.1, 444.6, 497.1, 519.2, 648.5, 664.8, 744.6, 792.4, 863.5, 908.6, 922.4, 955.4, 972.5, 1029.8, 1053.5, 1059.0, 1093.6, 1111.0, 1165.7, 1170.7, 1183.9, 1269.6, 1291.2, 1296.1, 1364.4, 1369.5, 1393.2, 1401.7, 1418.7, 1445.9, 1454.7, 1477.9, 1483.2, 1493.7, 1849.1, 2990.8, 3007.1, 3026.7, 3086.8, 3107.3, 3125.0, 3135.8, 3167.1, 3562.5, 3768.8

B: 1.422817 0.898906 0.621398

C	-1.734399	-0.197564	0.126770
O	-2.524601	-0.538764	-0.798233
O	-0.970580	-1.044109	0.714853
O	-0.922616	-2.283911	0.036053
H	-1.632878	-2.174368	-0.631334
C	-1.718221	1.178256	0.712457
H	-2.719667	1.574801	0.864587
H	-1.191788	1.122570	1.662718
C	-0.931569	2.051394	-0.277943
H	-0.781819	3.031202	0.173546
H	-1.517515	2.180308	-1.187979
C	0.418344	1.438900	-0.625692
H	0.295592	0.486853	-1.157803
H	0.953183	2.089279	-1.332806
N	1.236566	1.294251	0.546205
C	2.468604	0.596790	0.322059
H	2.850996	0.222758	1.270552
H	3.180701	1.323274	-0.094283
O	2.445468	-0.456344	-0.615285
O	2.025347	-1.629521	0.070241
H	1.422817	-1.734399	-0.197564

SP-21e

$\tilde{\nu}$: -614.5, 80.0, 103.1, 126.0, 150.7, 182.5, 228.6, 249.0, 304.7, 321.1, 352.0, 381.5, 430.7, 435.4, 480.3, 549.6, 612.5, 655.3, 740.8, 793.3, 853.2, 888.3, 912.6, 951.7, 964.1, 1015.7, 1080.7, 1098.1, 1120.6, 1132.1, 1172.7, 1207.1, 1256.8, 1276.5, 1297.8, 1309.5, 1363.3, 1365.1, 1385.8, 1399.4, 1445.4, 1463.3, 1482.3, 1495.4, 1513.1, 1524.7, 1749.0, 1892.6, 3041.7, 3066.7, 3085.9, 3091.2, 3102.6, 3125.3, 3162.4, 3586.8, 3671.0

B: 1.506185 0.971650 0.786253

H	-1.613118	-1.163630	-0.741459
C	-2.367729	-0.240216	0.214467
H	-3.018646	-0.579592	1.016417
C	-2.032815	1.253093	0.403676
H	-1.800410	1.436894	1.454990
H	-2.924583	1.834763	0.174259
C	-0.865978	1.767449	-0.439871
H	-0.900408	2.860100	-0.455258
H	-0.960285	1.422542	-1.472359
N	0.402946	1.321294	0.107349
H	0.390894	0.936011	1.041738
C	1.453544	0.818980	-0.672192
H	1.745667	1.477285	-1.492988
H	1.167767	-0.216341	-1.155792
O	2.603055	0.551384	0.068940
O	2.230718	-0.268396	1.167686
H	1.901134	-1.084940	0.747998
C	-1.110617	-1.064581	0.240205
O	-0.527000	-1.484445	1.190460
O	-0.677641	-1.204854	-1.056792
O	1.506185	-1.613118	-1.163630

$HOOC(O)CH_2CH_2CH_2NHCHO$

$\tilde{\nu}$: 37.1, 64.3, 76.0, 117.2, 187.6, 223.7, 251.7, 299.4, 332.1, 361.4, 441.5, 459.2, 498.1, 567.7, 649.4, 746.1, 757.6, 856.2, 894.3, 930.6, 944.4, 1015.4, 1048.6, 1053.8, 1080.2, 1100.3, 1185.2, 1226.4, 1239.0, 1281.5, 1327.5, 1373.6, 1386.4, 1403.9, 1427.1, 1474.9, 1483.9, 1491.0, 1506.0, 1535.2, 1803.4, 1856.1, 3021.5, 3083.6, 3086.5, 3094.8, 3131.0, 3137.9, 3154.4, 3572.8, 3648.4

B: 1.875774 0.992202 0.838760

C	1.371724	0.377702	0.438811
O	1.405911	-0.206436	1.486816
O	1.816725	-0.196354	-0.704347
O	2.155118	-1.552832	-0.499657
H	2.097881	-1.624377	0.476102
C	0.821542	1.743137	0.167980
H	1.124018	2.383858	0.993751
H	1.250203	2.125143	-0.757853
C	-0.710578	1.693611	0.070137
H	-1.066188	2.705281	-0.124750
H	-1.134576	1.375772	1.022445
C	-1.228837	0.766265	-1.028227
H	-2.288695	0.963145	-1.183955
H	-0.704344	0.946722	-1.966510
N	-1.083868	-0.641881	-0.697463
H	-0.344140	-1.187797	-1.107147
C	-1.922357	-1.235159	0.183078
H	-1.696057	-2.299737	0.343998
O	-2.832753	-0.665400	0.741624

OH
$\tilde{\nu}$: 3769.4
B: 0.000000 564.286338 564.286338

O	-0.000000	-0.000000	0.107992
H	0.000000	0.000000	-0.863937

SP-21f

$\tilde{\nu}$: -2393.1, 40.1, 66.3, 114.5, 154.4, 178.3, 223.7, 272.6, 322.6, 349.0, 355.5, 424.1, 494.0, 507.0, 546.8, 597.6, 627.8, 681.5, 706.0, 771.6, 837.9, 870.6, 892.5, 941.8, 961.0, 1040.8, 1053.7, 1116.8, 1120.8, 1148.3, 1182.4, 1218.6, 1231.9, 1232.7, 1253.9, 1301.3, 1335.8, 1372.5, 1377.9, 1393.5, 1422.3, 1483.2, 1486.1, 1491.0, 1518.2, 1538.6, 1557.3, 1904.2, 3023.9, 3078.2, 3090.1, 3100.2, 3114.0, 3134.3, 3164.6, 3189.2, 3576.5

H	-1.770179	0.870742	1.711930
C	1.702658	0.445744	0.914682
H	2.714651	0.533543	1.307086
C	1.251914	1.804173	0.352583
H	2.025954	2.175168	-0.317718
H	1.153699	2.512435	1.175807
C	-0.053652	1.721614	-0.419836
H	0.056960	0.991844	-1.231109
H	-0.259555	2.687020	-0.885707
N	-1.179802	1.387223	0.449124

B: 1.468289 0.947204 0.691801

H	-0.916193	0.817993	1.242904
C	-2.304653	0.842828	-0.212416
H	-2.565293	1.444742	-1.081836
H	-3.149707	0.742555	0.464633
O	-2.061563	-0.472160	-0.775723
O	-2.099223	-1.379118	0.233123
H	-1.052831	-1.671403	0.464510
C	1.774430	-0.540802	-0.217856
O	2.515886	-0.472657	-1.143523
O	0.883021	-1.615594	-0.246784
O	1.468289	-1.770179	0.870742

$HOOC(O)CH_2CH_2CH_2NHCH_2O\dot{O}$

$\tilde{\nu}$: 29.9, 55.9, 77.4, 106.6, 116.8, 141.4, 185.2, 257.6, 270.2, 340.2, 357.3, 428.4, 430.0, 467.6, 515.4, 596.1, 615.1, 700.8, 741.4, 845.9, 869.6, 903.7, 939.7, 960.2, 1011.4, 1043.8, 1081.8, 1091.9, 1148.1, 1200.5, 1235.8, 1243.7, 1259.0, 1289.8, 1306.8, 1342.3, 1383.5, 1394.7, 1411.4, 1413.3, 1467.6, 1483.0, 1485.6, 1508.6, 1514.3, 1527.7, 1828.2, 3037.5, 3074.5, 3079.8, 3089.4, 3105.5, 3126.6, 3135.1, 3176.2, 3560.0, 3594.0

B: 1.882737 0.561629 0.478262

C	-0.067525	-0.225234	0.111366
O	-1.995296	0.025690	1.294608
O	-2.408385	-0.614535	-0.797534
O	-2.958832	-1.764148	-0.190451
H	-2.835903	-1.563203	0.759419
C	-1.390355	1.488270	-0.538005
H	-1.196372	1.284442	-1.590459
H	-2.193289	2.226253	-0.485449
C	-0.145796	2.008001	0.176335
H	-0.334246	2.018705	1.251381
H	0.041589	3.037378	-0.126407
C	1.090158	1.175180	-0.130768
H	1.324775	1.232789	-1.196404
H	1.954362	1.580012	0.405008
N	0.846768	-0.227980	0.179607
H	0.575773	-0.368908	1.145069
C	1.809268	-1.156913	-0.256917
H	1.991455	-1.061241	-1.325567
H	1.523763	-2.169116	0.017642
O	3.120226	-0.978446	0.383167
O	1.882737	-0.067525	-0.225234

$O_2NOOCOCH_2CH_2CH_2NHCH_2OOH$

$\tilde{\nu}$: 22.6, 51.5, 61.2, 71.6, 82.5, 100.0, 122.7, 142.9, 197.5, 237.9, 298.0, 332.2, 349.0, 365.8, 398.5, 438.8, 471.4, 498.6, 535.3, 552.3, 615.5, 639.5, 712.7, 755.7, 782.6, 823.7, 864.2, 874.9, 924.7, 927.9, 938.0, 1013.1, 1055.8, 1070.3, 1072.8, 1087.9, 1115.5, 1167.5, 1202.3, 1246.1, 1256.2, 1304.9, 1366.8, 1378.3, 1388.9, 1399.0, 1406.8, 1411.9, 1438.7, 1466.8, 1473.6, 1487.0, 1492.2, 1513.9, 1846.7, 1899.8, 3039.7, 3071.2, 3075.1, 3078.2, 3096.8, 3123.8, 3135.2, 3137.4, 3616.8, 3769.5

B: 0.954284 0.516347 0.415964

H	0.633753	0.111333	-0.668854
C	-0.709513	-0.417534	0.272929
H	-0.913762	-1.053305	1.065001
C	-1.386515	-0.110817	0.262145
H	2.094893	1.036855	1.126259
H	2.644748	2.520740	0.358683
C	2.282441	0.929833	-1.029310
H	3.362830	0.778852	-1.068345
H	2.027568	1.568416	-1.880357
N	1.672314	-0.367931	-1.222156
H	0.664051	-0.395652	-1.221953
C	2.294448	-1.496330	-0.624113
H	3.354243	-1.503047	-0.887077
H	1.812318	-2.406827	-0.976589
O	2.325133	-1.536150	0.799546
O	1.043081	-1.962457	1.239957
H	0.622895	-1.132647	1.508699
C	-0.521832	1.251896	0.587449
O	-0.516288	0.417536	1.435278
O	-1.603439	1.432921	-0.258521
O	0.954284	0.633753	0.111333
N	0.516347	-0.709513	-0.417534

O	0.415964	-0.913762	-1.053305
O	-3.408624	-1.386515	-0.110817

$\dot{C}H_2CH_2CH_2NHCH_2OOH$

$\tilde{\nu}$: 62.0, 79.8, 137.0, 148.8, 163.6, 226.0, 280.5, 328.8, 423.6, 458.0, 506.2, 602.2, 728.6, 825.2, 884.2, 910.3, 924.4, 1015.7, 1048.4, 1099.1, 1124.8, 1166.9, 1218.3, 1243.7, 1277.8, 1347.4, 1361.2, 1386.8, 1396.5, 1411.9, 1461.3, 1471.7, 1475.9, 1511.2, 1516.5, 2996.1, 3027.7, 3070.3, 3089.7, 3101.0, 3130.9, 3161.5, 3267.9, 3556.2, 3834.8

B: 5.479767 1.268401 1.191996

C	2.763806	-0.050955	-0.852760
C	2.233875	-0.295451	0.514677
C	0.712331	-0.407801	0.530580
N	0.113701	0.869639	0.165103
C	-1.291820	0.989695	0.296300
O	-2.088523	0.201439	-0.594279
O	-2.097208	-1.138546	-0.106025
H	2.395358	-0.638876	-1.682457
H	3.621073	0.580743	-1.024183
H	2.542629	0.502098	1.191660
H	2.644418	-1.231377	0.916657
H	0.362635	-0.662419	1.532634
H	0.400927	-1.221002	-0.134240
H	0.430362	1.171238	-0.748258
H	-1.596390	2.006149	0.046158
H	-1.588077	0.747216	1.318553
H	-3.032148	-1.257307	0.097409

NO_2

$\tilde{\nu}$: 783.5, 1465.2, 1775.4

B: 253.9654019 13.2904639 12.6295376

N	0.000000	0.000000	0.314442
O	0.000000	1.090266	-0.137569
O	0.000000	-1.090266	-0.137569

CO_2

$\tilde{\nu}$: 694.4, 694.4, 1410.9, 2443.8

B: 0.000000 11.838234 11.838234

O	0.000000	0.000000	1.155205
C	0.000000	0.000000	0.000000
O	0.000000	0.000000	-1.155205

SP-24a

$\tilde{\nu}$: -351.5, 48.7, 75.8, 102.0, 119.9, 128.2, 175.0, 211.5, 257.6, 271.0, 314.5, 356.6, 395.8, 422.0, 450.8, 460.3, 631.2, 691.2, 705.9, 757.4, 786.0, 847.6, 883.8, 939.0, 962.8, 1016.3, 1050.3, 1059.4, 1083.2, 1092.3, 1178.4, 1212.4, 1251.7, 1288.5, 1310.3, 1329.3, 1372.1, 1386.5, 1405.7, 1456.5, 1481.7, 1489.8, 1493.4, 1527.8, 1546.9, 1744.2, 1858.5, 2737.8, 3073.1, 3091.0, 3097.4, 3124.8, 3145.2, 3159.4, 3162.8, 3294.6, 3382.3

B: 1.376492 0.980974 0.703489

C	-1.770018	0.093479	0.274789
O	1.076910	0.523120	1.422780
O	2.100927	-0.039691	-0.516902
O	2.520159	-1.224512	0.121950
H	1.671352	-1.729189	0.123841
C	0.787599	1.883871	-0.527714
H	0.836897	2.776134	0.095258
H	1.379835	2.040627	-1.425871
C	-0.668988	1.591767	-0.912132
H	-0.716203	0.709900	-1.554628
H	-1.035672	2.436208	-1.494703
C	-1.619133	1.393712	0.279925
H	-1.264764	1.938374	1.153423
H	-2.616432	1.750213	0.026162
N	-1.755852	-0.010573	0.662804
H	-0.882440	-0.582414	0.892296
C	-2.684124	-0.759737	0.212616
H	-3.498161	-0.335493	-0.364626
H	-2.685830	-1.806031	0.472906
O	-0.836674	-2.015090	-0.937381
O	1.376492	-1.770018	0.093479

POST-24a

$\tilde{\nu}$: 27.3, 54.4, 89.1, 104.6, 108.0, 132.0, 145.3, 182.5, 193.0, 238.5, 294.8, 312.7, 354.8, 414.8, 425.1, 443.5, 525.5, 629.0, 720.8, 750.6, 853.7, 886.1, 900.8, 947.7, 957.2, 1018.2, 1070.9,

C	1.994821	-0.225095	-0.301878
O	1.080066	-0.341275	-1.392942
O	2.039744	-0.124471	0.635348
O	2.624329	1.062830	0.148643
H	1.847170	1.599098	-0.098515

1088.0, 1100.1, 1112.2, 1183.3, 1228.4, 1250.9, 1283.9, 1306.5, 1313.4, 1367.7, 1387.4, 1407.0, 1481.8, 1488.8, 1493.1, 1507.3, 1526.3, 1619.5, 1775.3, 1867.9, 2796.1, 3041.4, 3065.3, 3078.6, 3095.6, 3107.7, 3125.4, 3155.2, 3203.5, 3648.0

B: 1.149503 0.977846 0.604159

C	0.801265	-2.074556	0.254142
H	0.856042	-2.795921	-0.560431
H	1.443864	-2.408394	1.065583
C	-0.647345	-1.964789	0.746994
H	-0.690998	-1.332245	1.634601
H	-0.963225	-2.961234	1.054023
C	-1.640908	-1.434522	-0.293850
H	-1.280185	-1.658613	-1.299066
H	-2.616104	-1.911582	-0.160772
N	-1.779695	0.012422	-0.194827
H	-0.597850	1.158229	-0.328606
C	-2.921672	0.505037	0.006145
H	-3.815249	-0.117776	0.093857
H	-3.022891	1.581350	0.098807
O	-0.737996	2.809272	0.508419
O	1.149503	1.994821	-0.225095

HOOC(O)$CH_2CH_2CH_2N=CH_2$

$\tilde{\nu}$: 62.7, 107.1, 138.6, 166.0, 177.1, 233.2, 309.3, 380.3, 447.6, 503.8, 534.5, 586.5, 721.0, 733.4, 762.5, 793.2, 851.1, 893.5, 958.0, 1049.2, 1067.8, 1087.4, 1100.8, 1114.3, 1168.0, 1210.4, 1260.6, 1280.8, 1314.2, 1365.7, 1383.3, 1392.7, 1485.4, 1497.7, 1502.5, 1515.2, 1589.8, 1782.1, 1868.9, 3034.7, 3058.0, 3075.8, 3092.6, 3122.4, 3127.5, 3183.1, 3193.8, 3317.6

B: 2.427535 1.269400 0.985694

C	-2.957797	0.460852	-0.111932
H	-3.643532	-0.331726	-0.423167
H	-3.385721	1.396128	0.236445
N	-1.707954	0.324667	-0.149503
C	-1.149545	-0.936089	-0.617386
H	-1.937104	-1.621821	-0.945625
H	-0.519789	-0.706051	-1.480320
C	-0.297474	-1.601651	0.461776
H	0.178803	-2.472975	0.012685
H	-0.940368	-1.952934	1.269463
C	0.787727	-0.689819	1.058431
H	0.359018	0.054512	1.718553
H	1.492298	-1.299016	1.620140
C	1.570312	-0.059641	-0.066059
O	2.351793	-0.658917	-0.745050
O	1.314988	1.231114	-0.417818
O	0.464578	1.936255	0.468522
H	-0.418141	1.551692	0.244139

HO_2

$\tilde{\nu}$: 1252.8, 1459.4, 3686.8

B: 628.54317 34.69923 32.88385

H	-0.880747	-0.865418	0.000000
O	0.055047	0.708193	0.000000
O	0.055047	-0.600015	0.000000

SP-24b

$\tilde{\nu}$: -1623.8, 60.2, 85.3, 126.0, 153.8, 163.0, 237.2, 256.9, 305.9, 328.9, 413.4, 428.5, 452.7, 522.9, 530.6, 552.4, 639.4, 657.3, 715.8, 811.7, 840.2, 892.7, 925.5, 945.9, 976.7, 1016.0, 1045.3, 1066.4, 1081.6, 1122.3 1146.2, 1180.8, 1238.9, 1260.0, 1285.1, 1314.0, 1341.0, 1351.1, 1370.2, 1386.6, 1400.5, 1478.8, 1490.4, 1492.1, 1527.4, 1535.6, 1634.8, 1879.8, 3060.1, 3087.7, 3095.8, 3102.5, 3122.9, 3153.7, 3179.1, 3531.8, 3575.1

B: 1.417908 1.061933 0.798989

C	-1.334219	-0.928793	0.589799
O	1.024816	-0.446490	1.567104
O	1.866143	-0.493927	-0.531018
O	1.748498	-1.897539	-0.457341
H	0.779496	-2.007547	-0.552764
C	1.377768	1.609000	0.366280
H	1.091867	2.054062	1.316968
H	2.371591	1.971753	0.108806
C	0.390171	1.974452	-0.755111
H	0.874252	1.835932	-1.720507
H	0.141239	3.035327	-0.671171
C	-0.873446	1.148205	-0.768710
H	-1.540431	1.379390	-1.600350
H	-0.651799	-0.108940	-1.042368
N	-1.592497	1.005760	0.437592
H	-0.993998	0.987455	1.257678

C	-2.439498	-0.161919	0.422838
H	-3.118924	-0.111695	-0.429173
H	-2.975590	-0.266077	1.361423
O	-1.625014	-1.318250	0.322266
O	1.417908	-1.334219	-0.928793

HOOC(O)$CH_2CH_2\dot{C}HNHCH_2OOH$

$\tilde{\nu}$: 67.6, 88.4, 100.0, 128.7, 136.2, 182.9, 199.5, 211.4, 244.3, 270.8, 322.7, 358.5, 392.7, 436.0, 473.3, 479.8, 585.3, 618.6, 629.7, 684.6, 708.4, 872.7, 888.0, 916.3, 943.3, 972.6, 1045.6, 1059.2, 1064.6, 1114.1, 1149.9, 1176.9, 1223.6, 1265.7, 1290.8, 1332.5, 1354.3, 1377.5, 1385.4, 1414.9, 1448.1, 1477.3, 1484.4, 1487.4, 1512.7, 1553.0, 1868.9, 2990.0, 3091.0, 3095.5, 3099.0, 3144.5, 3164.0, 3214.4, 3595.9, 3679.4, 3733.4

B: 1.292339 1.112880 0.809543

C	-0.602419	-1.027069	0.394110
O	1.199859	-0.990344	1.488558
O	0.924097	-1.142393	-0.735419
O	0.300668	-2.380331	-0.467182
H	-0.365343	-2.137346	0.203418
C	2.080866	0.751605	0.071101
H	2.294033	1.209444	1.035102
H	3.038448	0.515456	-0.393390
C	1.284483	1.704351	-0.842037
H	1.447164	1.432154	-1.885420
H	1.726007	2.701963	-0.719243
C	-0.183186	1.694104	-0.591487
H	-0.871573	2.083345	-1.327288
H	-1.187323	-0.537703	-1.388717
N	-0.698010	1.513755	0.690149
H	-0.023269	1.246602	1.392296
C	-1.926330	0.787980	0.816110
H	-2.712887	1.277221	0.243674
H	-2.198905	0.717695	1.866856
O	-1.848523	-0.566886	0.372973
O	1.292339	-0.602419	-1.027069

SP-24c

$\tilde{\nu}$: -1994.1, 64.4, 109.8, 135.7, 147.7, 171.8, 233.6, 273.2, 304.8, 336.6, 373.3, 407.0, 436.0, 452.8, 503.6, 574.7, 632.6, 641.2, 750.8, 778.0, 830.1, 877.3, 897.7, 912.6, 961.1, 1014.1, 1029.6, 1063.6, 1068.8, 1091.5, 1124.0, 1153.8, 1210.0, 1225.9, 1278.1, 1296.6, 1311.8, 1363.8, 1374.4, 1377.6, 1400.9, 1464.5, 1471.5, 1481.6, 1483.3, 1490.9, 1540.1, 1875.9, 3040.7, 3066.4, 3090.5, 3094.9, 3130.9, 3146.1, 3160.5, 3576.7, 3697.6

B: 1.487611 1.029911 0.868993

C	-1.185766	-1.454846	0.348368
O	1.197899	0.624887	1.525672
O	1.924979	0.115150	-0.553567
O	2.532554	-1.017721	0.021234
H	1.761497	-1.605827	0.108985
C	0.381745	1.840300	-0.406133
H	0.150937	2.648118	0.292419
H	0.976366	2.236576	-1.226362
C	-0.902011	1.232752	-0.945434
H	-0.611767	-0.000464	-1.393801
H	-1.247497	1.707767	-1.860944
C	-2.021884	0.937014	0.034234
H	-2.329729	1.864982	0.525855
H	-2.885864	0.571416	-0.524217
N	-1.717323	-0.039619	1.072236
H	-1.007092	0.286431	1.713932
C	-1.491310	-1.377172	0.668867
H	-2.342431	-1.756064	0.102654
H	-1.293901	-1.996916	1.540855
O	-0.322391	-1.587625	-0.161548
O	1.487611	-1.185766	-1.454846

HOOC(O)$CH_2CH_2\dot{C}HNHCH_2OOH$

$\tilde{\nu}$: 66.1, 84.2, 110.1, 131.3, 158.8, 177.6, 185.1, 223.0, 275.1, 289.6, 339.1, 348.9, 369.7, 422.3, 445.9, 489.4, 557.1, 593.8, 632.8, 678.4, 759.7, 875.2, 884.8, 896.8, 916.9, 961.4, 1029.1, 1047.1, 1065.6, 1127.2, 1134.7, 1185.9, 1217.3, 1274.2, 1298.2,

C	-1.657289	-1.254923	0.332825
O	1.353254	0.686246	1.503202
O	1.720051	0.038905	-0.629228
O	2.384141	-1.078650	-0.086013
H	1.637912	-1.568377	0.308750
C	0.428189	1.968050	-0.342285

1306.8, 1354.2, 1381.5, 1385.8, 1405.4, 1444.8, 1460.2, 1465.7, 1475.4, 1507.7, 1538.3, 1885.1, 2992.1, 3059.6, 3088.6, 3103.8, 3132.8, 3149.4, 3216.5, 3592.9, 3662.8, 3759.6

B: 1.353830 1.083479 0.838808

H	0.168037	2.672604	0.451627
H	1.082387	2.469700	-1.053819
C	-0.788490	1.434583	-1.030816
H	0.071650	-0.971435	-1.527524
H	-0.864413	1.524762	-2.104118
C	-1.987192	1.011245	-0.240280
H	-2.521114	1.908328	0.100993
H	-2.677968	0.466693	-0.884880
N	-1.731049	0.188670	0.933891
H	-1.086613	0.602258	1.592544
C	-1.560801	-1.208280	0.791919
H	-2.394680	-1.633144	0.233550
H	-1.495402	-1.661642	1.779857
O	-0.359592	-1.680839	0.154163
O	1.353830	-1.657289	-1.254923

SP-24d

$\tilde{\nu}$: -1910.1, 46.0, 92.6, 109.3, 180.0, 202.0, 237.8, 250.0, 289.1, 324.9,
375.1, 417.4, 425.4, 457.1, 546.2, 577.3, 654.7, 684.2, 690.8, 778.9,
781.6, 876.9, 878.8, 909.3, 961.3, 1009.6, 1028.9, 1037.3, 1091.0, 1120.5,
1132.9, 1171.2, 1215.5, 1232.5, 1257.5, 1287.8, 1347.1, 1379.1, 1389.6, 1403.5,
1424.0, 1464.3, 1466.5, 1482.4, 1496.1, 1505.3, 1512.7, 1788.4, 3025.1, 3070.1,
3084.6, 3087.3, 3144.3, 3148.5, 3168.8, 3590.4, 3607.5

B: 1.948864 0.674546 0.622173

C	-1.803909	-0.081468	0.125967
O	-1.433774	0.912024	-0.810730
O	-2.584428	-0.585725	0.375945
O	-3.592713	-0.354670	-0.581221
H	-3.173324	0.317708	-1.155704
C	-0.419130	-0.165591	1.090838
H	-0.778060	-0.747318	1.933837
H	0.295974	-1.128418	0.416372
C	0.642868	0.850975	1.407674
H	1.437241	0.325702	1.941469
H	0.235267	1.580947	2.117034
C	1.238811	1.612328	0.209857
H	0.546159	2.384227	-0.116011
H	2.148003	2.112808	0.549913
N	1.567667	0.815316	-0.956837
H	0.746971	0.523559	-1.466836
C	2.549759	-0.182924	-0.832262
H	3.499084	0.231255	-0.490002
H	2.671576	-0.705035	-1.779504
O	2.286053	-1.188420	0.189331
O	1.948864	-1.803909	-0.081468

$HOOC(O)\dot{C}HCH_2CH_2NHCH_2OOH$

$\tilde{\nu}$: 33.6, 45.0, 88.6, 124.1, 145.3, 178.3, 187.7, 207.9, 256.1, 280.6, 324.8, 366.4, 415.4, 434.2, 471.6, 541.5, 586.2, 623.6, 665.3, 729.4, 767.5, 822.0, 878.7, 904.7, 957.4, 997.0, 1033.0, 1037.7, 1052.4, 1139.1, 1159.7, 1197.4, 1222.9, 1250.8, 1281.9, 1349.8, 1390.3, 1399.6, 1407.4, 1413.4, 1449.0, 1473.0, 1480.1, 1496.6, 1509.8, 1515.6, 1773.2, 3015.0, 3061.2, 3077.3, 3090.2, 3133.8, 3152.8, 3217.7, 3574.2, 3608.2, 3816.6

B: 1.554193 0.833020 0.682742

C	2.145920	0.016959	0.150316
O	1.376485	-1.048363	-0.948991
O	2.237637	0.385912	0.544258
O	3.200830	0.612291	-0.457476
H	2.892912	0.012869	-1.168535
C	0.341947	-0.743926	1.186624
H	0.549959	-0.270812	2.135615
H	0.260445	1.887394	0.535993
C	-0.905067	-1.524392	1.013026
H	-1.638311	-1.113674	1.709145
H	-0.734720	-2.559938	1.334183
C	-1.496077	-1.561179	-0.410525
H	-0.896651	-2.214357	-1.038479
H	-2.488562	-2.010836	-0.329663
N	-1.621512	-0.294847	-1.103171
H	-0.775673	-0.013150	-1.576334
C	-2.272451	0.777066	-0.447953
H	-3.185565	0.424162	0.035198

H	-2.509612	1.563131	-1.164038
O	-1.556688	1.391839	0.632773
O	1.554193	2.145920	0.016959

Atmospheric fate of the 4-piperidinyl radical

Under atmospheric conditions, the PIPĊ[4] radical will add O_2 forming a vibrationally excited peroxy radical, PIPC[4]OȮ[‡]. There are four chair conformations of PIPC[4]OȮ (*ee*, *ea*, *ae* and *aa*); the *ea* conformation has the lowest energy followed by *ee* and *ae* (+2 kJ mol^{-1}), and *aa* (+4 kJ mol^{-1}). The *ae* and *ee,* and the *ea* and *aa* conformers are connected via a relatively low barriers of ~20 kJ mol^{-1} to *N*-inversion, whereas the *ea* and *ae*, and the *aa* and *ee* conformers are connected via higher barriers of around 40 kJ mol^{-1} involving a series of linked skew and boat conformers constituting internal pseudo-rotation in PIPC[4]OȮ resembling that in PIPC[2]OȮ. Six pairs of pseudo-enantiomeric skew PIPC[4]OȮ conformations were located with 23 – 27 kJ mol^{-1} higher energy than the *ea* chair form. The energies and conformational pathways involving skew and boat forms of PIPC[4]OȮ were not investigated in detail, but the relative population of the skew conformations is < 0.1% in total, and it is presumed that internal H-transfer reactions involving these conformers can be omitted in modelling the atmospheric fate of the PIPC[3]OȮ radical.

The vibrationally excited PIPC[4]OȮ[‡] may initiate several internal H-transfer reactions before being quenched by collisions, and reaction with NO (the reaction enthalpies given below refer to the *ea* conformation of PIPC[4]OȮ). The 1,4-H transfer turns out to be irreversible and resulting in 1,2,3,6-tetrahydropyridine via a complex with HO_2 on the exit side.

$\Delta H^{\ominus}$ = -159 kJ mol^{-1} (26)

$\Delta H^{\ominus}$ = +49 kJ mol^{-1} (27a)

$\Delta H^{\ominus}$ = +18 kJ mol^{-1} (27b)

$\Delta H^{\ominus}$ = +97 kJ mol^{-1} (27c)

$\Delta H^{\ominus}$ = -43 kJ mol^{-1} (28)

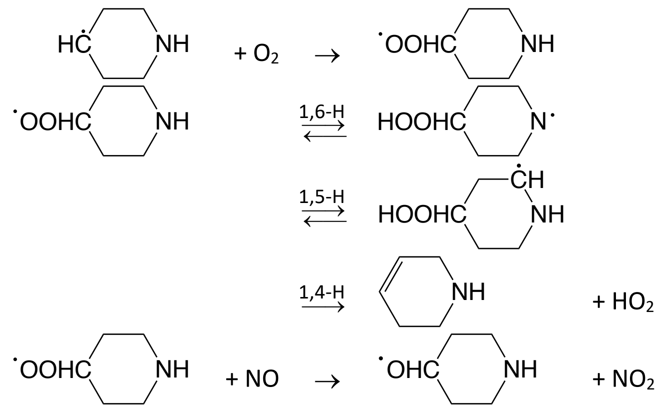

Not all routes of reaction 27 are feasible within each of the four conformational sub-spaces of the PIPC[4]OȮ radical. Route 27a is only possible in the *ae* conformation. A scan of the C[4]OȮ group to uncover the minimum energy path locates an additional saddle point (resembling the E_N ring structure in piperidine, see section 3.1 in the main text and Table S2) and a skew form saddle point to the H-transfer. The energy of the intermediate saddle point is much lower than that of the H-transfer. Further, route 27b is only within reach in the *aa* and *ea* conformations. The barriers to routes 27a – 27c are calculated for the *ea* conformer to be 104, 92 and 134 kJ mol^{-1}, respectively.

Figure S10 illustrates the energetics of reactions relevant to the atmospheric fate of the PIPC[4]OȮ radical. The atmospheric fate of the PIPC[4]OȮ radical was simulated in master equation calculations based on the PES illustrated in Figure 10 and including O_2 sinks for QOOH radicals, $k_{QOOH+O2}$ = 1.3 × 10^{-11} cm^3 molecule^{-1} s^{-1} and an O_2 sink for aminyl radicals, $k_{aminyl+O2}$ = 1 × 10^{-17} cm^3 molecule^{-1} s^{-1} (see section3.2.1.2 in the main text). The rate coefficient of reaction 26 is in the order of 10^{-11} cm^3 molecule^{-1} s^{-1} and the PIPĊ[4] radical will consequently not be fully thermalised at the time of peroxy radical formation. Assuming equipartitioning of the reaction enthalpy in reaction 1d and ~10 quenching collisions before reaction, PIPĊ[4] could bring as much as an additional ~40 kJ mol^{-1} into reaction 26. Taking k_{28} = 6.7 × 10^{-12} cm^3 molecule^{-1} s^{-1}, the calculations predict a PIPC[4]Ȯ yield of >99% for NO > 20 ppt. It makes essentially no difference to the model results should the PIPĊ[4] radical carry an additional 40 kJ mol^{-1} internal energy when entering reaction 26.

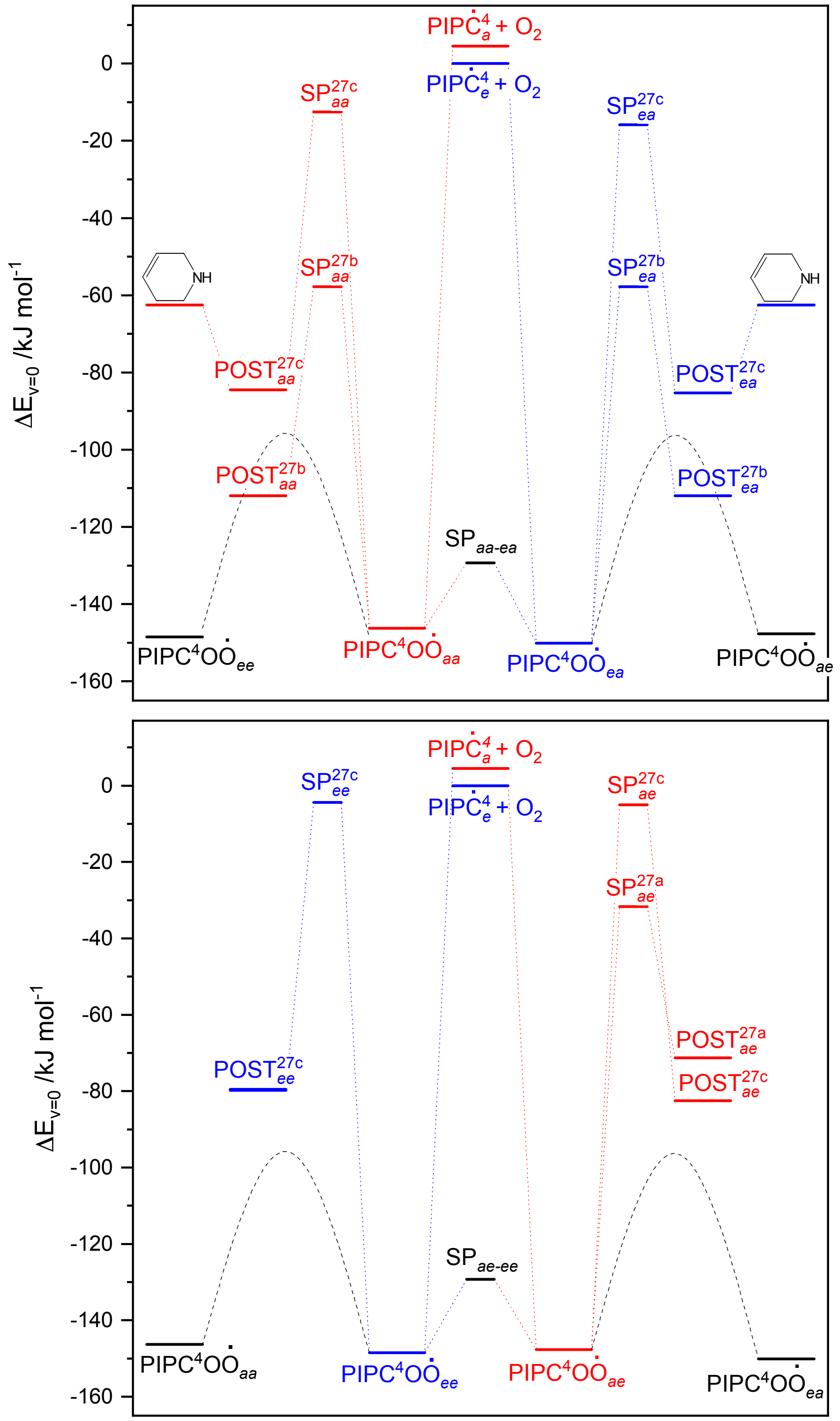

Figure S10. PES of the PIP$\dot{C}^4$ + O_2 reaction.
Relative energies of stationary points on the potential energy surfaces of relevance to internal H-transfer reactions in the 4-piperidinyl peroxy radical, PIPC^4O$\dot{O}$. Top: *ea* and *aa* manifold. Bottom: *ae* and *ee* manifold. The dashed curves mimic the complex conformational links between the *ea* and *ae*, and the *ee* and *aa* conformers. Results from CCSD(T*)-F12a/aug-cc-pVTZ//M06-2X/aug-cc-pVTZ calculations. The underlying quantum chemistry data are collected in Table S16.

There are four chair conformations of the PIPC^{4}Ȯ radical – *aa*, *ae*, *ea* and *ee* – of which the *ee* and *ae*, and the *ea* and *aa* conformations are pairwise linked by relatively low barriers of around 20 kJ mol^{-1}, whereas the *ee* and *aa*, and the *ae* and *ea* conformer pairs are expected to be connected via barriers of around 40 kJ mol^{-1}.

The PIPC^{4}Ȯ radical may either undergo ring opening, internal H-transfer reactions, or undergo H-abstraction by O_2. The reaction with O_2 leads to the corresponding ketone, piperidin-4-one:

$\Delta H^{\ominus}$ =+29 kJ mol^{-1} (29)

$\Delta H^{\ominus}$ = -62 kJ mol^{-1} (30a)

$\Delta H^{\ominus}$ = -45 kJ mol^{-1} (30b)

$\Delta H^{\ominus}$ = -152 kJ mol^{-1} (31)

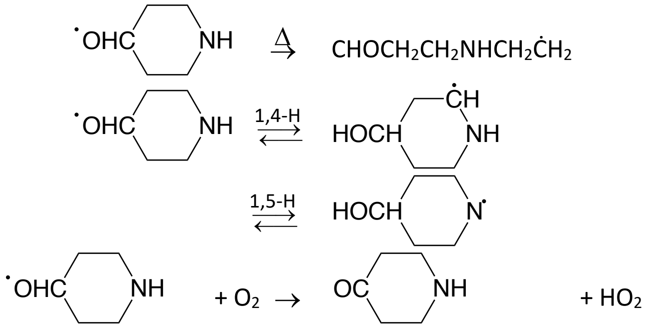

The barriers to ring-opening and to the 1,4- and 1,5-hydrogen shift reactions are calculated to be in the region 42 – 53 kJ mol^{-1}. Route 30a is only accessible within the *ea* and *aa* conformational sub-spaces, whereas route 30b is possible within the *ea* and *ae* conformational sub-spaces. The saddle point to route 30b has a boat structure, and scans of the oxy radical C^{4}Ȯ groups in the *ea* and *ae* conformations to uncover the minimum energy paths to reaction 30b locates additional envelope-like saddle points to intermediate skew forms. Figure S11 illustrates the stationary points on the PES related to the atmospheric fate of the PIPC^{4}Ȯ radical.

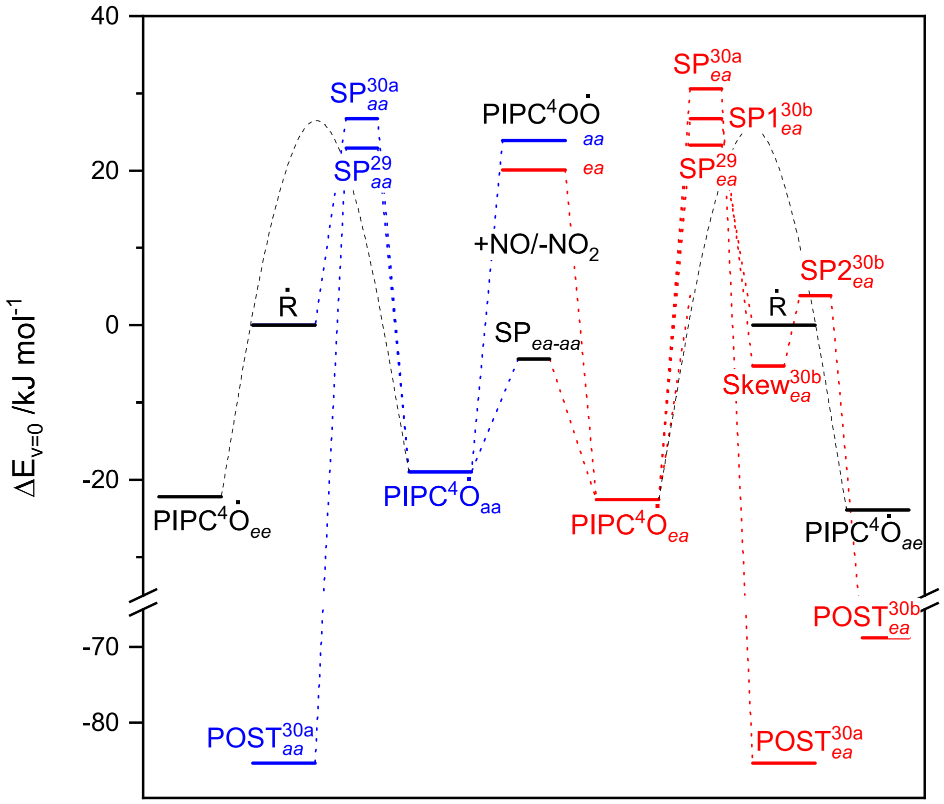

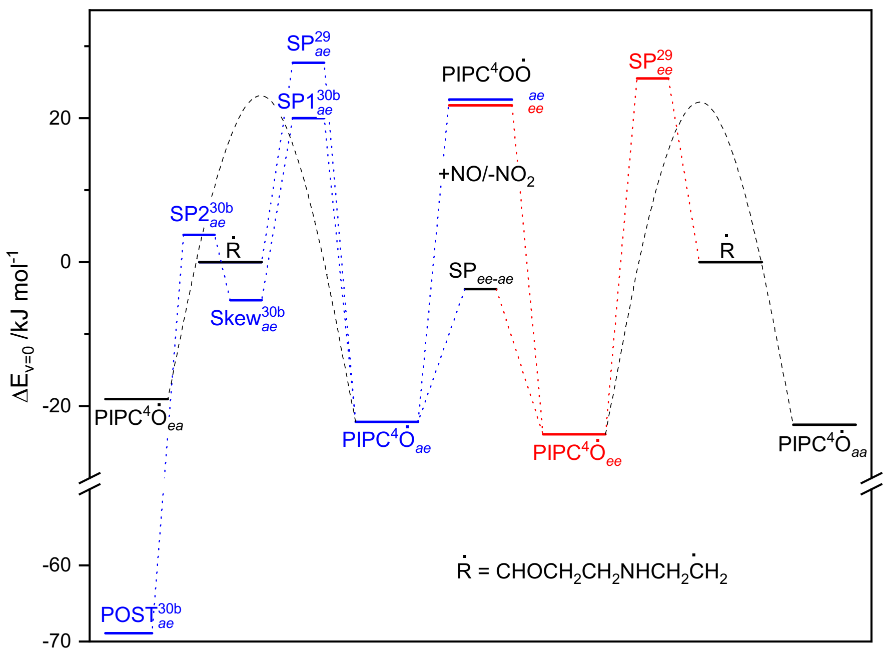

Figure S11. PES of PIPC⁴Ȯ reactions.

Relative energies of stationary points on the potential energy surfaces of relevance to the atmospheric fate of the 4-piperidinyl oxy radical, PIPC⁴Ȯ. Top: *ea* and *aa* manifold. Bottom: *ae* and *ee* manifold. The dashed curves mimic the complex conformational links between the *ea* and *ae*, and the *ee* and *aa* conformers. Results from CCSD(T*)-F12a/aug-cc-pVTZ//M06-2X/aug-cc-pVTZ calculations. The underlying quantum chemistry data are collected in Table S17.

The branching in reactions 29 – 31 was examined using a master equation model based on the potential energy surface indicated in Figure 11 and O_2 loss reactions for the alkyl and aminyl radicals formed in reactions 30 and 31. The calculations reveal that the branching between reactions 29 – 31 is essentially independent on the energy partitioning in reaction 28. Assuming PIPC⁴OȮ to be thermalised when entering reaction 28, results in an average branching of 29 : 30a : 30b : 31 = 18% : 1% : 31% : 51%.

The alkyl radical formed in 30a will add O_2 forming a vibrationally excited peroxy radical that will initiate internal H-transfer reactions similar to those elaborated in section **Error! Reference source not found.**. It is assumed that the by far dominating product will be the corresponding imine, 2,3,4,5-tetrahydropyridin-4-ol:

ΔH^{Θ} = -149 kJ mol^{-1} 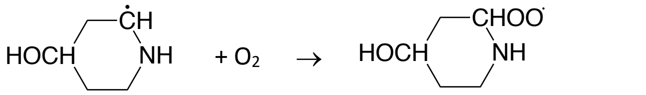 (32)

ΔH^{Θ} = +92 kJ mol^{-1} 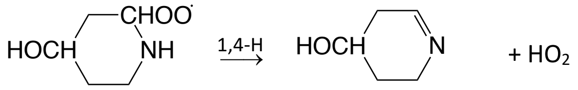 (33)

Neglecting the minor reactions with NO, NO_2 and O_3, the aminyl radical formed in 30b will add O_2 forming a vibrationally excited peroxy radical that will initiate internal H-transfer reactions similar to those elaborated in section 3.1.2.1 in the main text. It is assumed that the by far dominating product will be the imine, 2,3,4,5-tetrahydropyridin-4-ol:

ΔH^{Θ} = -40 kJ mol^{-1} 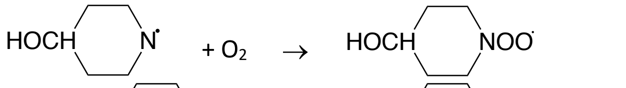 (34)

ΔH^{Θ} = -34 kJ mol^{-1} 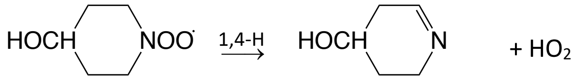 (35)

In summary, the major products following H-abstraction from C⁴ are projected to be ~20% $CHOCH_2CH_2NHCH_2\dot{C}H_2$, ~30% 2,3,4,5-tetrahydropyridin-4-ol and ~50% piperidin-4-one. The alkyl radical, $CHOCH_2CH_2NHCH_2\dot{C}H_2$, is expected to undergo autoxidation reactions similar to those outlined for $CHOCH_2CH_2CH_2NH\dot{C}H_2$ in section 3.1.2.3 in the main text.

Table S16. QCC results for the PIP-C⁴OȮ conformational pathways and internal H-transfer reactions
Energies (/Hartree) of the 4-piperidinyl peroxy radical (PIPC⁴OȮ) conformations and stationary points on the internal H-transfer reactions in PIPC⁴OȮ, and energy differences of stationary points on the potential energy surface relative to that of the initial reactants.

Species	M06-2X/aTZ		CCSD(T*)-F12a/aTZ	
	E_{Elec}	E_{ZPE}	E_{Elec}	$\Delta E_{v=0}$
PIP-$\dot{C}_e^4$	-251.21082	0.14546	-250.89424	
O_2	-150.32480	0.00400	-150.19114	
Sum reactants	-401.53561	0.14946	-401.08537	0.0
PIP-$\dot{C}_a^4$	-251.20881	0.14524	-250.89232	
O_2	-150.32480	0.00400	-150.19114	
Sum reactants	-401.53361	0.14924	-401.08346	4.5
PIP-C⁴OȮ$_{ea}$	-401.59848	0.15675	-401.14984	-150.1
SP_{ea}^{27b}	-401.55711	0.15145	-401.10936	-57.8
$POST_{ea}^{27b}$	-401.58318	0.15533	-401.13386	-111.9
SP_{ea}^{27c}	-401.53675	0.15020	-401.09213	-15.8
$POST_{ea}^{27c}$	-401.56882	0.15266	-401.12106	-85.3
1,2,3,6-tetrahydropyridine	-250.64888	0.13603	-250.33142	
HO_2	-150.90809	0.01458	-150.77891	
Sum products	-401.55697	0.15061	-401.11033	-62.5
SP_{aa-ea}	-401.59009	0.15519	-401.14034	-129.3
PIPC⁴OȮ$_{aa}$	-401.59678	0.15650	-401.14815	-146.3
SP_{aa}^{27b}	-401.55711	0.15145	-401.10936	-57.8
$POST_{aa}^{27b}$	-401.58318	0.15533	-401.13386	-111.9
SP_{aa}^{27c}	-401.53586	0.15064	-401.09133	-12.5
$POST_{ea}^{27c}$	-401.56825	0.15254	-401.12064	-84.5
1,2,3,6-tetrahydropyridine	-250.64888	0.13603	-250.33142	
HO_2	-150.90809	0.01458	-150.77891	
Sum products	-401.55697	0.15061	-401.11033	-62.5
PIPC⁴OȮ$_{ee}$	-401.59785	0.15667	-401.14914	-148.5
SP_{ee}^{27c}	-401.53348	0.15011	-401.08770	-4.4
$POST_{ee}^{27c}$	-401.56961	0.15376	-401.12007	-79.8
SP_{ee-ae}	-401.59009	0.15519	-401.14034	-129.3
PIP-C⁴OȮ$_{ae}$	-401.59620	0.15643	-401.14757	-149.5

SP^{27a}_{ea}	-401.54864	0.15042	-401.10282	-43.3
$POST^{27a}_{ea}$	-401.56789	0.15458	-401.11992	-77.2
SP^{27c}_{ae}	-401.53361	0.15007	-401.08790	-5.0
$POST^{27c}_{ae}$	-401.57051	0.15379	-401.12113	-82.5

Table S16, continued.
T_1 and D_1 diagnostic values, vibrational frequencies (cm^{-1}), Rotational constants (GHz) and Cartesian coordinates of the species listed above. Results from CCSD(T*)-F12a/aug-cc-pVTZ//M06-2X/aug-cc-pVTZ calculations.

$PIP\dot{C}^4{}_e$	C	0.032376	-0.756088	1.206027
	N	-0.666044	-1.183023	0.000000
T_1= 0.01174001 , D_1= 0.03582035	C	0.032376	-0.756088	-1.206027
	C	0.032376	0.770965	-1.275732
$\tilde{\nu}$: 186.8, 235.2, 359.2, 400.3, 456.8, 475.4, 621.5, 783.8, 808.6, 848.0, 909.1, 921.4, 1007.4, 1048.9, 1063.5, 1084.9, 1106.3, 1181.1, 1189.4, 1204.6, 1257.2, 1322.5, 1333.9, 1347.1, 1372.1, 1400.3, 1420.3, 1460.2, 1469.2, 1482.9, 1501.1, 1511.5, 2962.9, 2964.1, 2985.5, 2986.6, 3099.6, 3100.0, 3103.9, 3104.7, 3204.6, 3568.2	C	0.534491	1.353904	-0.000000
	C	0.032376	0.770965	1.275732
	H	1.075361	-1.108655	1.219522
	H	-0.476591	-1.173859	2.074134
	H	-0.781580	-2.187055	0.000000
	H	-0.476591	-1.173859	-2.074134
	H	1.075361	-1.108655	-1.219522
B: 4.726136 4.595880 2.592119	H	0.626670	1.109947	-2.124600
	H	-1.002757	1.094780	-1.454394
	H	1.014558	2.321835	-0.000000
	H	0.626670	1.109947	2.124600
	H	-1.002757	1.094780	1.454394
$PIP\dot{C}^4{}_a$	C	-0.031260	-0.764939	-1.209792
	N	0.581676	-1.296947	-0.000000
T_1= 0.01240021 , D_1= 0.04243278	C	-0.031260	-0.764939	1.209792
	C	-0.031260	0.772878	1.270366
$\tilde{\nu}$: 122.9, 223.8, 289.6, 381.9, 463.6, 475.5, 611.8, 785.4, 800.3, 845.2, 899.0, 924.1, 973.3, 1011.3, 1026.0, 1106.5, 1111.6, 1141.8, 1171.6, 1247.2, 1269.2, 1323.1, 1327.0, 1341.5, 1360.7, 1398.2, 1403.4, 1462.2, 1474.2, 1486.0, 1494.9, 1497.5, 2960.4, 2963.2, 3054.6, 3056.8, 3098.6, 3099.3, 3108.2, 3108.4, 3215.2, 3529.9	C	-0.562458	1.336252	0.000000
	C	-0.031260	0.772878	-1.270366
	H	-1.064011	-1.121456	-1.237756
	H	0.482665	-1.182326	-2.075654
	H	1.574580	-1.091645	-0.000000
	H	0.482665	-1.182326	2.075654
	H	-1.064011	-1.121456	1.237756
B: 4.685309 4.562141 2.593708	H	-0.600217	1.122114	2.132531
	H	1.009440	1.098598	1.423278
	H	-1.177076	2.223625	0.000000
	H	-0.600217	1.122114	-2.132531
	H	1.009440	1.098598	-1.423278
O_2	O	0.000000	0.000000	0.594925
T_1= 0.00775897, D_1= 0.01442723	O	0.000000	0.000000	-0.594925
$\tilde{\nu}$: 1754.5				
B: 44.635532				
$PIPC^4O\dot{O}_{ea}$	C	-1.451353	-0.959942	-0.494241
	N	-1.960248	0.370742	-0.182798

T_1= 0.02364182, D_1= 0.14139103

$\tilde{\nu}$: 87.6, 156.2, 203.6, 315.7, 337.0, 398.3, 446.5, 507.6, 558.1, 658.8, 792.3, 813.4, 845.8, 898.1, 916.3, 933.8, 1020.1, 1058.0, 1069.5, 1108.8, 1145.7, 1172.2, 1195.7, 1219.1, 1270.0, 1298.3, 1325.7, 1358.0, 1359.9, 1371.9, 1380.0, 1397.0, 1435.2, 1464.1, 1478.0, 1487.0, 1506.9, 1518.1, 2985.7, 2989.0, 3078.1, 3079.6, 3106.1, 3112.0, 3113.8, 3127.5, 3134.1, 3569.4

B: 3.484863 1.699231 1.418605

C	-0.902443	1.373101	-0.257933
C	0.144172	1.080711	0.808887
C	0.679096	-0.336129	0.682601
C	-0.429845	-1.365988	0.561853
O	1.464005	-0.456212	-0.543087
O	2.549418	0.250260	-0.494641
H	-0.978926	-1.000027	-1.486159
H	-2.281041	-1.665500	-0.492222
H	-2.713644	0.611146	-0.812275
H	-1.335523	2.357607	-0.087478
H	-0.416660	1.386929	-1.244508
H	0.975978	1.781197	0.745927
H	-0.312127	1.176511	1.795530
H	1.365863	-0.571543	1.495077
H	-0.000671	-2.340860	0.329846
H	-0.926660	-1.433552	1.530672

SP_{ea}^{27b}

T_1= 0.02554919, D_1= 0.14848986

$\tilde{\nu}$: -1709.9, 185.4, 237.5, 277.3, 372.6, 418.8, 427.3, 525.5, 556.3, 634.4, 707.1, 777.7, 832.1, 871.8, 892.9, 923.3, 962.4, 1016.0, 1041.7, 1062.4, 1078.3, 1112.3, 1142.6, 1167.9, 1212.0, 1229.9, 1259.9, 1306.0, 1330.9, 1352.2, 1362.6, 1383.6, 1390.1, 1436.7, 1462.4, 1476.7, 1502.2, 1523.0, 1606.6, 3062.8, 3066.7, 3069.2, 3106.4, 3109.2, 3125.6, 3136.2, 3147.5, 3595.5

B: 2.886846 2.103448 1.941700

C	-0.630027	-1.337189	0.004396
N	-1.704008	-0.437234	-0.167470
C	-1.333590	0.959324	-0.425032
C	-0.221225	1.429092	0.514412
C	0.879607	0.402201	0.776120
C	0.263464	-0.945407	1.153619
O	1.767886	0.173974	-0.311866
O	1.066703	-0.252969	-1.436409
H	0.214185	-1.117810	-0.933933
H	-0.941423	-2.377231	-0.032290
H	-2.393622	-0.767486	-0.826104
H	-2.220258	1.574543	-0.274115
H	-1.003328	1.081649	-1.457388
H	0.221180	2.342369	0.117735
H	-0.653189	1.667048	1.489193
H	1.544206	0.768500	1.558368
H	1.053480	-1.678653	1.303374
H	-0.319265	-0.848448	2.072561

POST_{ea}^{27b}

T_1= 0.01577828, D_1= 0.07054027

$\tilde{\nu}$: 124.9, 139.1, 239.8, 279.7, 349.8, 369.6, 410.8, 422.9, 499.8, 522.7, 670.3, 686.9, 770.7, 818.4, 882.9, 912.1, 936.8, 966.3, 1027.7, 1060.8, 1079.2, 1119.6, 1132.7, 1182.1, 1232.5, 1269.5, 1283.4, 1343.5, 1363.0, 1381.5, 1394.2, 1397.2, 1442.9, 1459.9, 1461.1, 1473.2, 1508.5, 1530.3, 2961.0, 3062.3, 3066.5, 3086.7, 3107.3, 3107.6, 3125.8, 3196.1, 3618.9, 3704.7

B: 2.895266 1.942269 1.784050

C	-1.034751	1.226928	0.379872
N	-1.802278	0.073623	0.360927
C	-1.141579	-1.218601	0.183513
C	-0.098002	-1.150266	-0.928320
C	0.808436	0.076979	-0.876152
C	-0.014401	1.353425	-0.704343
O	1.868421	-0.013750	0.069370
O	1.358130	-0.262783	1.372802
H	0.930396	0.577611	1.604745
H	-1.550777	2.114538	0.719535
H	-2.538810	0.053712	1.048182
H	-1.904074	-1.950450	-0.082143
H	-0.665808	-1.539711	1.111294
H	0.513301	-2.052016	-0.910055
H	-0.614600	-1.116305	-1.890561
H	1.372453	0.138096	-1.808966
H	0.654305	2.193171	-0.515650
H	-0.511067	1.547462	-1.667666

SP_{ea}^{27c}

T_1= 0.02354010, D_1= 0.11515195

$\tilde{\nu}$: -1089.3, 95.8, 128.3, 205.2, 261.7, 349.6, 455.3, 457.7, 489.7, 575.6, 682.5, 734.7, 798.0, 844.9, 873.9, 924.3, 959.7, 1002.9, 1011.0, 1048.7, 1077.1, 1117.0, 1155.7, 1211.2, 1231.9, 1250.3, 1285.6, 1312.5, 1343.7, 1372.4, 1394.0, 1411.0, 1430.5, 1457.3, 1486.7, 1508.9, 1519.0, 1579.4, 1635.1, 2967.2, 2976.4, 3054.2, 3100.3, 3109.1, 3118.0, 3161.4, 3196.3, 3570.4

B: 3.151762 1.681177 1.413379

C	1.266703	0.938898	-0.693054
N	1.984422	-0.240158	-0.223297
C	1.089352	-1.379076	-0.050371
C	-0.130548	-0.994652	0.782401
C	-0.415775	0.345698	1.024690
C	0.408365	1.452385	0.451319
O	-2.005119	0.565856	-0.304333
O	-2.132639	-0.610554	-0.733607
H	0.624344	0.721193	-1.560000
H	1.986913	1.697769	-0.993560
H	2.723436	-0.483447	-0.868315
H	1.633172	-2.171872	0.461957
H	0.763624	-1.784048	-1.019983
H	-1.171338	-1.118727	-0.033400
H	-0.423423	-1.700450	1.552135
H	-1.064252	0.598581	1.853696
H	-0.231576	2.277296	0.137275
H	1.061631	1.822881	1.246884

$POST_{ea}^{27c}$

T_1= 0.02169285, D_1= 0.12778153

$\tilde{\nu}$: 30.2, 63.5, 85.5, 103.4, 185.1, 223.1, 316.9, 415.0, 480.0, 480.9, 526.5, 677.2, 725.9, 830.5, 870.7, 914.5, 958.4, 1003.0, 1029.6, 1035.9, 1073.5, 1100.7, 1154.3, 1210.1, 1223.9, 1252.9, 1270.0, 1298.7, 1349.2, 1369.5, 1419.1, 1428.2, 1469.8, 1490.9, 1496.2, 1512.4, 1517.6, 1729.6, 2939.7, 2973.3, 3061.2, 3083.2, 3115.5, 3118.4, 3170.9, 3191.6, 3454.8, 3579.6

B: 2.796273 1.358791 1.247751

C	0.903071	1.146637	-0.669341
N	1.936215	0.116471	-0.667540
C	1.393748	-1.199191	-0.370753
C	0.511057	-1.145967	0.843925
C	0.072982	-0.001294	1.363894
C	0.390442	1.331684	0.751327
O	-2.586819	0.500024	-0.309249
O	-2.279781	-0.740405	-0.583877
H	0.055303	0.884538	-1.321004
H	1.333395	2.073765	-1.043675
H	2.426795	0.099626	-1.550369
H	2.217495	-1.889930	-0.182720
H	0.814623	-1.621090	-1.210159
H	-1.460029	-0.912727	-0.066593
H	0.254766	-2.092364	1.307020
H	-0.535251	-0.017442	2.261183
H	-0.502886	1.956382	0.752272
H	1.147284	1.835780	1.357523

1,2,3,6-tetrahydropyridine

T_1= 0.00775897, D_1= 0.01442723

$\tilde{\nu}$: 173.0, 293.1, 405.5, 487.9, 528.8, 657.9, 756.3, 805.5, 866.1, 903.5, 928.0, 984.6, 1012.6, 1019.4, 1057.6, 1114.8, 1145.0, 1201.6, 1222.7, 1266.3, 1332.1, 1355.5, 1372.6, 1398.6, 1420.6, 1476.9, 1483.6, 1492.7, 1497.7, 1743.2, 3030.2, 3036.3, 3057.4, 3080.6, 3090.5, 3112.0, 3168.0, 3190.5, 3536.2

B: 4.940573 4.754902 2.664883

C	-1.449742	-0.166939	0.090310
N	-0.558942	-1.261168	-0.279430
C	0.761783	-1.096866	0.319338
C	1.460868	0.190322	-0.115902
C	0.509754	1.353586	-0.055727
C	-0.801922	1.192182	0.063122
H	-1.845014	-0.361883	1.093354
H	-2.310950	-0.181521	-0.579469
H	-0.464462	-1.284346	-1.288082
H	1.368954	-1.969824	0.081714
H	0.624716	-1.082972	1.403580
H	2.325149	0.384816	0.523334
H	1.853709	0.081862	-1.132194
H	0.928042	2.352388	-0.099173
H	-1.451999	2.055948	0.146104

HO_2

T_1= 0.03589638, D_1= 0.12282697

H	-0.880747	-0.865418	0.000000
O	0.055047	0.708193	0.000000

$\tilde{\nu}$: 12528, 1459., 3686.8
B: 628.5431720, 34.6992321, 32.8838526

O	0.055047	-0.600015	0.000000

SP$_{aa-ea}$

T_1= 0.02368137, D_1= 0.14139453

$\tilde{\nu}$: -604.9, 86.0, 152.0, 196.9, 295.9, 334.8, 383.0, 450.8, 529.4, 562.2,
665.7, 807.5, 840.1, 886.6, 905.8, 941.1, 1003.0, 1030.6, 1051.7, 1113.7,
1144.9, 1175.4, 1208.5, 1252.4, 1270.1, 1298.8, 1347.0, 1359.1, 1365.4, 1369.6,
1388.5, 1399.4, 1427.0, 1452.7, 1475.4, 1477.9, 1494.5, 1502.5, 2997.3, 2999.9,
3069.5, 3071.6, 3102.5, 3117.7, 3122.4, 3123.4, 3126.6, 3744.8

B: 3.414033 1.717314 1.428178

C	0.882614	1.400278	-0.265453
N	1.895774	0.382889	-0.321099
C	1.459501	-0.973672	-0.497039
C	0.443704	-1.366738	0.579990
C	-0.668337	-0.336039	0.689573
C	-0.149291	1.088544	0.820070
O	-1.444189	-0.461757	-0.541531
O	-2.541093	0.226909	-0.495291
H	0.363624	1.482213	-1.229989
H	1.348251	2.364515	-0.070740
H	2.858610	0.602357	-0.175950
H	2.320040	-1.638955	-0.456521
H	0.994797	-1.106691	-1.482876
H	0.011529	-2.346378	0.371202
H	0.943680	-1.414235	1.548955
H	-1.360658	-0.574568	1.496664
H	-0.993717	1.776325	0.775384
H	0.316531	1.179740	1.803302

PIPC^{4}OȮ$_{aa}$

T_1= 0.02368875, D_1= 0.14171092

$\tilde{\nu}$: 85.9, 154.7, 196.1, 308.8, 333.4, 391.7, 449.9, 513.7, 562.8, 650.5, 784.8, 799.4, 846.9, 878.2, 903.0, 949.5, 985.5, 1020.1, 1053.9, 1098.0, 1141.1, 1160.4, 1202.0, 1238.9, 1275.9, 1297.7, 1340.4, 1356.3, 1358.7, 1368.5, 1387.8, 1395.4, 1411.2, 1465.9, 1480.9, 1490.1, 1494.8, 1501.6, 3059.1, 3062.6, 3067.7, 3068.8, 3102.7, 3110.8, 3117.6, 3120.2, 3122.1, 3530.7

B: 3.454894 1.698483 1.421722

C	0.891862	1.377965	-0.290709
N	1.967385	0.394973	-0.357246
C	1.448940	-0.957650	-0.517515
C	0.443249	-1.360398	0.564801
C	-0.669013	-0.334131	0.692042
C	-0.137821	1.086205	0.802174
O	-1.462418	-0.463578	-0.526628
O	-2.551591	0.236656	-0.471430
H	0.394482	1.387033	-1.263518
H	1.325167	2.366492	-0.144847
H	2.537756	0.450227	0.479049
H	2.283818	-1.657112	-0.532854
H	0.967357	-1.011395	-1.496389
H	0.015349	-2.341693	0.354777
H	0.949235	-1.422009	1.531313
H	-1.349129	-0.567324	1.510880
H	-0.974413	1.783555	0.756202
H	0.327449	1.190848	1.785810

SP$_{aa}^{27b}$

T_1= 0.02554919, D_1= 0.14848986

$\tilde{\nu}$: -1709.9, 185.4, 237.5, 277.3, 372.6, 418.8, 427.3, 525.5, 556.3, 634.4, 707.1, 777.7, 832.1, 871.8, 892.9, 923.3, 962.4, 1016.0, 1041.7, 1062.4, 1078.3, 1112.3, 1142.6, 1167.9, 1212.0, 1229.9, 1259.9, 1306.0, 1330.9, 1352.2, 1362.6, 1383.6, 1390.1, 1436.7, 1462.4, 1476.7, 1502.2, 1523.0, 1606.6, 3062.8, 3066.7, 3069.2, 3106.4, 3109.2, 3125.6, 3136.2, 3147.5, 3595.5

B: 2.886846 2.103448 1.941700

C	-0.630027	-1.337189	0.004396
N	-1.704008	-0.437234	-0.167470
C	-1.333590	0.959324	-0.425032
C	-0.221225	1.429092	0.514412
C	0.879607	0.402201	0.776120
C	0.263464	-0.945407	1.153619
O	1.767886	0.173974	-0.311866
O	1.066703	-0.252969	-1.436409
H	0.214185	-1.117810	-0.933933
H	-0.941423	-2.377231	-0.032290
H	-2.393622	-0.767486	-0.826104
H	-2.220258	1.574543	-0.274115
H	-1.003328	1.081649	-1.457388
H	0.221180	2.342369	0.117735
H	-0.653189	1.667048	1.489193

H	1.544206	0.768500	1.558368
H	1.053480	-1.678653	1.303374
H	-0.319265	-0.848448	2.072561

POST$^{27b}_{aa}$

T_1= 0.01577828, D_1= 0.07054024

$\tilde{\nu}$: 124.9, 139.1, 239.8, 279.7, 349.8, 369.6, 410.8, 422.9, 499.8, 522.7, 670.3, 686.9, 770.7, 818.4, 882.9, 912.1, 936.8, 966.3, 1027.7, 1060.8, 1079.2, 1119.6, 1132.7, 1182.1, 1232.5, 1269.5, 1283.4, 1343.5, 1363.0, 1381.5, 1394.2, 1397.2, 1442.9, 1459.9, 1461.1, 1473.2, 1508.5, 1530.3, 2961.0, 3062.3, 3066.5, 3086.7, 3107.3, 3107.6, 3125.8, 3196.1, 3618.9, 3704.7

B: 2.895266 1.942269 1.784050

C	-1.034750	1.226929	0.379868
N	-1.802278	0.073625	0.360927
C	-1.141580	-1.218600	0.183516
C	-0.098003	-1.150268	-0.928317
C	0.808436	0.076976	-0.876152
C	-0.014399	1.353423	-0.704346
O	1.868421	-0.013752	0.069371
O	1.358129	-0.262777	1.372803
H	0.930392	0.577617	1.604739
H	-1.550775	2.114540	0.719530
H	-2.538810	0.053716	1.048182
H	-1.904076	-1.950449	-0.082137
H	-0.665809	-1.539707	1.111298
H	0.513299	-2.052019	-0.910050
H	-0.614601	-1.116309	-1.890558
H	1.372453	0.138089	-1.808966
H	0.654308	2.193168	-0.515654
H	-0.511064	1.547459	-1.667671

SP$^{27c}_{aa}$

T_1= 0.02329009, D_1= 0.11445451

$\tilde{\nu}$: -1085.5, 91.9, 125.9, 201.0, 259.0, 345.8, 455.2, 468.4, 495.0, 585.3, 684.2, 741.1, 798.2, 822.5, 868.4, 904.7, 926.2, 984.7, 1016.0, 1025.2, 1061.4, 1124.8, 1147.1, 1188.6, 1231.1, 1265.5, 1280.5, 1347.4, 1351.1, 1376.3, 1395.0, 1396.3, 1411.1, 1461.9, 1488.8, 1494.7, 1502.8, 1575.4, 1633.6, 3029.1, 3046.6, 3061.1, 3099.3, 3113.0, 3117.0, 3151.6, 3191.5, 3528.9

B: 3.143513 1.677210 1.413886

C	1.080236	1.385973	-0.089648
N	1.994516	0.287178	-0.380312
C	1.268771	-0.931531	-0.718830
C	0.421807	-1.440169	0.444289
C	-0.404374	-0.336817	1.027256
C	-0.130201	1.004725	0.773885
O	-2.002477	-0.580238	-0.272513
O	-2.144373	0.588705	-0.714740
H	0.745966	1.794899	-1.046824
H	1.642680	2.177035	0.403898
H	2.580377	0.110978	0.427833
H	1.976099	-1.694894	-1.039994
H	0.622967	-0.706866	-1.571897
H	-0.216455	-2.272843	0.145928
H	1.079408	-1.815937	1.237062
H	-1.032158	-0.592955	1.871402
H	-1.180664	1.114504	-0.029871
H	-0.422471	1.715012	1.540968

POST$^{27c}_{aa}$

T_1= 0.02167079, D_1= 0.12781121

$\tilde{\nu}$: 36.6, 61.8, 83.5, 102.8, 175.5, 210.6, 302.0, 414.6, 473.3, 489.5, 531.8, 670.2, 762.8, 803.0, 864.6, 902.8, 922.6, 986.1, 1011.8, 1029.0, 1057.5, 1114.6, 1145.2, 1202.6, 1223.4, 1266.5, 1270.5, 1333.0, 1354.2, 1371.5, 1398.4, 1421.4, 1472.2, 1480.6, 1491.9, 1500.2, 1511.1, 1722.5, 3019.4, 3038.9, 3059.4, 3094.9, 3106.4, 3119.3, 3160.5, 3184.6, 3461.8, 3541.6

B: 2.766994 1.369425 1.259942

C	-1.360127	-1.204441	-0.418291
N	-1.882797	0.089097	-0.834682
C	-0.885103	1.145609	-0.690401
C	-0.406779	1.323320	0.749356
C	-0.103476	-0.011276	1.370389
C	-0.518205	-1.157627	0.833090
O	2.559999	0.522127	-0.299149
O	2.292020	-0.734037	-0.541202
H	-0.769458	-1.625653	-1.240718
H	-2.195874	-1.889729	-0.271451
H	-2.695552	0.317552	-0.274866
H	-1.296489	2.075220	-1.080712
H	-0.032163	0.881504	-1.321575
H	0.484888	1.950418	0.779531

H	-1.172061	1.833210	1.343632
H	0.473613	-0.024634	2.288274
H	1.474729	-0.916727	-0.024499
H	-0.266071	-2.103069	1.303113

PIPC^{4}OȮ$_{ee}$

T_1= 0.02362667, D_1= 0.14109890

$\tilde{\nu}$: 120.7, 134.7, 246.7, 309.6, 329.4, 370.8, 415.4, 471.3, 545.9, 669.9, 784.8, 836.2, 850.8, 904.6, 931.8, 966.2, 1003.3, 1054.0, 1075.0, 1136.8, 1159.4, 1167.2, 1193.2, 1234.6, 1249.8, 1296.3, 1311.7, 1331.3, 1355.4, 1358.9, 1390.4, 1416.3, 1429.5, 1479.2, 1485.6, 1499.3, 1506.1, 1516.2, 2955.2, 2955.4, 3079.8, 3082.1, 3083.3, 3112.7, 3113.7, 3136.5, 3139.9, 3574.0

B: 3.967633 1.518174 1.263691

C	-1.491588	1.206679	0.108174
N	-1.995245	-0.000001	-0.531985
C	-1.491587	-1.206680	0.108175
C	0.026772	-1.255721	-0.025734
C	0.618758	0.000001	0.585753
C	0.026771	1.255721	-0.025735
O	2.068533	0.000002	0.474230
O	2.468559	-0.000002	-0.759892
H	-1.758952	1.249585	1.176636
H	-1.934115	2.075621	-0.376403
H	-3.005683	-0.000001	-0.544499
H	-1.934113	-2.075622	-0.376402
H	-1.758951	-1.249585	1.176637
H	0.428923	-2.138894	0.471057
H	0.298732	-1.291833	-1.080425
H	0.471720	0.000001	1.668162
H	0.428921	2.138896	0.471055
H	0.298731	1.291833	-1.080426

SP$^{27c}_{ee}$

T_1= 0.02230770, D_1= 0.12874508

$\tilde{\nu}$: -2237.9, 132.8, 195.6, 284.3, 356.0, 395.3, 417.5, 502.0, 538.3, 596.9, 716.9, 790.5, 815.1, 850.7, 906.7, 938.7, 954.3, 997.6, 1016.6, 1059.2, 1076.5, 1115.8, 1144.4, 1175.2, 1198.6, 1214.7, 1236.6, 1284.8, 1304.5, 1342.0, 1349.1, 1369.5, 1405.2, 1423.5, 1477.5, 1484.4, 1490.4, 1511.5, 1762.5, 2895.7, 2949.0, 3065.3, 3088.3, 3089.4, 3116.3, 3132.2, 3143.3, 3578.0

B: 3.617750 1.706422 1.411223

C	-1.598187	1.060733	0.012537
N	-1.831916	-0.240725	-0.600843
C	-1.247561	-1.316653	0.179210
C	0.215063	-1.092775	0.404457
C	0.695600	0.327102	0.704820
C	-0.109763	1.375571	-0.051610
O	2.058606	0.272435	0.291908
O	1.992903	-0.348663	-0.958331
H	-1.941626	1.089153	1.060106
H	-2.161346	1.813220	-0.536776
H	-2.820259	-0.403715	-0.732446
H	-1.409877	-2.269003	-0.327552
H	-1.718655	-1.406309	1.177581
H	0.696491	-1.848662	1.021947
H	1.014000	-1.067571	-0.663743
H	0.717929	0.542100	1.776498
H	0.098766	2.362858	0.359363
H	0.205007	1.358966	-1.094187

POST$^{27c}_{ee}$

T_1= 0.00775897, D_1= 0.01442723

$\tilde{\nu}$: 86.0, 144.0, 197.6, 221.1, 289.9, 349.6, 375.4, 410.0, 475.1, 500.2, 639.3, 669.4, 799.0, 844.0, 911.6, 925.6, 953.6, 975.4, 1020.2, 1073.7, 1086.4, 1115.0, 1155.7, 1192.5, 1201.7, 1230.9, 1280.3, 1316.8, 1324.5, 1345.4, 1368.8, 1404.7, 1410.2, 1423.4, 1484.0, 1487.8, 1492.4, 1511.3, 2875.2, 2945.9, 2977.4, 3089.0, 3089.8, 3112.2, 3147.6, 3185.6, 3570.2, 3806.9

B: 3.784925 1.511061 1.283347

C	1.545096	-1.151358	0.088134
N	1.961424	0.050096	-0.623492
C	1.487493	1.262359	0.033362
C	0.011944	1.213866	0.232036
C	-0.620184	-0.063213	0.666397
C	0.029292	-1.276746	0.013429
O	-2.036456	-0.038152	0.506902
O	-2.344625	0.017923	-0.878593
H	1.861298	-1.136297	1.144734
H	2.013658	-2.014968	-0.381394
H	2.968065	0.079201	-0.709830
H	1.758795	2.128837	-0.570746
H	1.970746	1.401830	1.021431

H	-0.513993	2.135510	0.447931
H	-2.341931	0.967549	-1.053698
H	-0.533152	-0.161661	1.759521
H	-0.314066	-2.183098	0.511478
H	-0.272590	-1.315194	-1.031609

SP_{ee-ae}

T_1= 0.02373582, D_1= 0.14143238

$\tilde{\nu}$: -593.7, 119.2, 132.5, 239.3, 292.9, 332.3, 349.6, 420.4, 477.9, 580.2, 688.8, 830.5, 837.7, 908.9, 923.4, 961.5, 995.5, 1034.9, 1054.5, 1133.7, 1159.0, 1159.3, 1230.3, 1250.9, 1254.8, 1291.2, 1326.5, 1326.8, 1366.5, 1373.7, 1390.4, 1408.5, 1423.0, 1462.0, 1486.3, 1495.4, 1497.4, 1504.6, 2964.7, 2965.2, 3073.8, 3074.9, 3076.4, 3123.3, 3124.1, 3130.9, 3134.1, 3744.1

B: 3.942064 1.517157 1.253833

C	1.494887	-1.227405	0.107671
N	2.037018	0.000001	-0.398745
C	1.494886	1.227406	0.107672
C	-0.030157	1.259555	-0.043128
C	-0.610203	-0.000001	0.572949
C	-0.030156	-1.259556	-0.043129
O	-2.062976	-0.000002	0.479866
O	-2.478018	0.000002	-0.749062
H	1.742459	-1.352849	1.173032
H	1.940584	-2.066606	-0.422698
H	2.732335	0.000001	-1.115047
H	1.940582	2.066607	-0.422696
H	1.742458	1.352849	1.173033
H	-0.450449	2.139267	0.446271
H	-0.296863	1.284467	-1.099839
H	-0.450512	-0.000001	1.654089
H	-0.450447	-2.139268	0.446269
H	-0.296862	-1.284466	-1.099840

PIPC[4]OȮ$_{ae}$

T_1= 0.02371841, D_1= 0.14156750

$\tilde{\nu}$: 124.0, 131.8, 234.1, 310.4, 327.8, 358.7, 421.2, 479.1, 557.9, 682.2, 775.7, 820.9, 836.6, 911.0, 917.5, 967.1, 980.1, 1029.4, 1047.8, 1119.0, 1137.3, 1180.2, 1202.9, 1251.4, 1253.7, 1289.3, 1324.1, 1329.5, 1360.7, 1370.7, 1389.2, 1397.2, 1417.2, 1477.6, 1491.8, 1498.6, 1499.2, 1505.3, 3046.6, 3048.4, 3069.1, 3069.8, 3081.6, 3115.6, 3117.1, 3121.7, 3125.3, 3538.8

B: 3.946766 1.507928 1.258818

C	-1.502203	1.206650	0.116170
N	-2.100416	-0.000000	-0.437724
C	-1.502203	-1.206650	0.116171
C	0.023975	-1.254807	-0.017421
C	0.617788	0.000000	0.591875
C	0.023975	1.254807	-0.017423
O	2.068946	-0.000000	0.472743
O	2.461492	0.000000	-0.763406
H	-1.776210	1.253442	1.174516
H	-1.948944	2.075411	-0.364903
H	-2.003370	-0.000001	-1.446417
H	-1.948944	-2.075411	-0.364900
H	-1.776210	-1.253440	1.174518
H	0.429861	-2.139987	0.474349
H	0.304227	-1.293657	-1.071686
H	0.476909	0.000001	1.675000
H	0.429861	2.139988	0.474345
H	0.304228	1.293655	-1.071687

SP_{ae}^{27a}

T_1= 0.04312515, D_1= 0.28180013

$\tilde{\nu}$: -2118.6, 164.0, 199.5, 291.2, 340.8, 364.6, 456.4, 507.8, 563.7, 668.9, 745.5, 813.3, 831.3, 888.2, 906.3, 928.8, 965.3, 978.9, 1041.5, 1045.6, 1097.8, 1114.5, 1132.0, 1208.6, 1224.0, 1253.9, 1286.5, 1312.7, 1344.6, 1354.8, 1365.4, 1375.4, 1387.1, 1397.0, 1474.0, 1477.4, 1489.0, 1505.7, 1613.4, 3042.3, 3047.1, 3074.2, 3091.3, 3108.5, 3121.7, 3130.3, 3138.2, 3156.1

B: 2.670089 2.268732 2.139121

C	-1.186461	0.929063	-0.692268
N	-0.990577	-0.487827	-0.967671
C	-1.025461	-1.276917	0.257675
C	0.168023	-0.942791	1.189145
C	0.682114	0.460007	0.891973
C	-0.497213	1.384046	0.612242
O	1.562757	0.502343	-0.249949
O	1.342678	-0.560779	-1.119822
H	-2.264260	1.111680	-0.642855
H	-0.800499	1.483560	-1.545483
H	0.143324	-0.590196	-1.366285
H	-1.023997	-2.332034	-0.005638

H	-1.968847	-1.054222	0.764882
H	-0.155550	-0.978453	2.229225
H	0.972660	-1.661440	1.057789
H	1.290210	0.834056	1.714454
H	-1.189250	1.334887	1.455096
H	-0.159243	2.414002	0.518076

$POST_{ae}^{27a}$

T_1= 0.01467240, D_1= 0.06108280

$\tilde{\nu}$: 79.6, 122.1, 195.2, 255.0, 323.5, 373.1, 452.1, 501.4, 532.7, 584.1, 742.5, 807.1, 829.4, 875.0, 888.3, 912.0, 960.3, 974.8, 1034.8, 1065.6, 1102.1, 1114.9, 1141.7, 1177.1, 1222.3, 1253.1, 1276.8, 1297.7, 1340.6, 1357.2, 1369.9, 1382.7, 1392.0, 1459.7, 1473.6, 1480.5, 1491.5, 1517.8, 2998.0, 3001.3, 3071.7, 3080.3, 3100.9, 3117.8, 3122.5, 3135.2, 3171.3, 3696.1

B: 2.761897 2.102045 1.910748

C	1.308838	-0.768596	-0.797933
N	0.915713	0.597649	-1.065071
C	0.976096	1.389961	0.138369
C	-0.123238	0.932264	1.133988
C	-0.484990	-0.527364	0.907772
C	0.778855	-1.300893	0.551563
O	-1.410312	-0.765769	-0.175444
O	-1.900407	0.418999	-0.776634
H	2.406853	-0.804311	-0.808389
H	0.958460	-1.388912	-1.619992
H	-1.204454	0.612052	-1.427086
H	0.852266	2.441491	-0.110709
H	1.959495	1.262831	0.609900
H	0.255582	1.024373	2.151543
H	-1.007089	1.555320	1.046278
H	-0.957551	-0.931872	1.804183
H	1.507459	-1.166093	1.353211
H	0.571378	-2.366492	0.470620

SP_{ae}^{27c}

T_1= 0.02191360, D_1= 0.12511623

$\tilde{\nu}$: -2223.5, 121.1, 184.9, 283.4, 341.1, 391.6, 422.6, 509.1, 549.3, 604.9, 728.0, 777.3, 806.4, 831.7, 912.9, 923.8, 948.8, 979.5, 1005.6, 1046.3, 1055.3, 1116.8, 1145.0, 1178.3, 1188.5, 1215.0, 1251.1, 1294.2, 1322.9, 1341.1, 1353.9, 1366.9, 1394.0, 1406.6, 1462.1, 1483.3, 1497.0, 1499.6, 1755.3, 2994.5, 3045.9, 3068.1, 3075.7, 3094.9, 3115.3, 3120.4, 3125.9, 3536.8

B: 3.588272 1.699179 1.409267

C	1.615946	1.052166	-0.014816
N	1.933219	-0.260637	0.534779
C	1.251177	-1.323218	-0.186837
C	-0.215985	-1.093037	-0.423528
C	-0.691037	0.331647	-0.711694
C	0.122527	1.375304	0.041711
O	-2.052896	0.284521	-0.288324
O	-1.982083	-0.345293	0.957171
H	1.969733	1.067899	-1.050470
H	2.186639	1.806638	0.524632
H	1.673730	-0.288987	1.514151
H	1.413330	-2.272853	0.324488
H	1.739655	-1.424461	-1.166182
H	-0.704910	-1.842190	-1.045182
H	-1.019185	-1.078850	0.640669
H	-0.720679	0.551511	-1.781990
H	-0.083965	2.364881	-0.366536
H	-0.202808	1.369878	1.083172

$POST_{ae}^{27c}$

T_1= 0.01273462, D_1= 0.03768218

$\tilde{\nu}$: 85.8, 143.6, 187.5, 223.6, 291.9, 340.6, 371.2, 416.0, 473.7, 503.1, 666.2, 675.3, 795.1, 829.8, 872.0, 917.7, 968.4, 971.1, 1008.0, 1043.4, 1098.3, 1109.0, 1135.2, 1172.1, 1210.9, 1243.0, 1306.2, 1314.9, 1327.7, 1358.4, 1368.8, 1383.8, 1398.3, 1410.1, 1472.2, 1483.1, 1497.8, 1499.2, 2973.3, 2978.5, 3044.5, 3076.1, 3099.7, 3111.5, 3126.7, 3176.4, 3529.9, 3816.8

C	-1.565424	-1.147458	-0.082866
N	-2.062553	0.064421	0.558114
C	-1.498479	1.261998	-0.060217
C	-0.013351	1.215807	-0.223642
C	0.612491	-0.058791	-0.671999
C	-0.041807	-1.276593	-0.030431
O	2.029861	-0.050419	-0.512447
O	2.333988	0.016050	0.874510
H	-1.904841	-1.130092	-1.123333
H	-2.033203	-2.011178	0.388104
H	-1.806054	0.048129	1.539037

B: 3.768799 1.503638 1.280493	H	-1.796762	2.136952	0.517454
	H	-1.978664	1.370884	-1.044168
	H	0.537066	2.135093	-0.384895
	H	2.446922	0.964518	1.012058
	H	0.524561	-0.146794	-1.765922
	H	0.296744	-2.181149	-0.536666
	H	0.280732	-1.332129	1.009955

Table S17. QCC results for the PIP-C$^{4}\dot{O}$ conformational pathways and internal H-transfer reactions
Energies (/Hartree) of the 4-piperidinyl oxy radical (PIPC$^{4}\dot{O}$) conformations and stationary points on the internal H-transfer reactions in PIPC$^{4}\dot{O}$, and energy differences of stationary points on the potential energy surface relative to that of the product from ring opening.

Species	M06-2X/aTZ		CCSD(T*)-F12a/aTZ	
	E_{Elec}	E_{ZPE}	E_{Elec}	$\Delta E_{v=0}$
PIPC^{4}O$\dot{O}_{aa}$ (reactant)	-401.59678	0.15650	-401.14815	
NO (reactant)	-129.89335	0.00471	-129.76980	
NO_2 (product)	-205.07464	0.00917	-204.88891	
Sum reactants – NO_2	-326.41549	0.15204	-326.02904	23.9
PIPC^{4}O$\dot{O}_{ea}$ (reactant)	-401.59848	0.15675	-401.14984	
NO (reactant)	-129.89335	0.00471	-129.76980	
NO_2 (product)	-205.07464	0.00917	-204.88891	
Sum reactants – NO_2	-326.41719	0.15228	-326.03073	20.1
PIPC$^{4}\dot{O}_{aa}$	-326.42764	0.15049	-326.04385	-19.0
SP$_{aa-ea}$	-326.42121	0.14924	-326.03704	-4.4
PIPC$^{4}\dot{O}_{ea}$	-326.42934	0.15081	-326.04555	-22.6
SP$^{29}_{aa}$	-326.40599	0.14731	-326.02328	26.7
$CHOCH_2CH_2NHCH_2\dot{C}H_2$	-326.41602	0.14534	-326.03146	0.0
SP$^{30a}_{aa}$	-326.40319	0.14756	-326.02203	30.6
POST$^{30a}_{aa}$	-326.45370	0.15155	-326.07021	-85.4
SP$^{29}_{ea}$	-326.40758	0.14767	-326.02494	23.3
$CHOCH_2CH_2NHCH_2\dot{C}H_2$	-326.41602	0.14534	-326.03146	0.0
SP$^{30a}_{ea}$	-326.40319	0.14756	-326.02203	30.6
POST$^{30a}_{ea}$	-326.45370	0.15155	-326.07021	-85.4
$SP1^{30b}_{ea}$	-326.41070	0.15084	-326.02678	26.7
SKEW$^{30b}_{ea}$	-326.42220	0.15068	-326.03882	-5.3
$SP2^{30b}_{ea}$	-326.41012	0.14672	-326.03140	3.8
POST$^{30b}_{ea}$	-326.44494	0.15064	-326.06297	-68.8

$PIPC^4O\dot{O}_{ae}$ (reactant)	-401.59745	0.15661	-401.14877	
NO (reactant)	-129.89335	0.00471	-129.76980	
NO_2 (product)	-205.07464	0.00917	-204.88891	
Sum reactants – NO_2	-326.41616	0.15215	-326.02966	22.6
$PIPC^4O\dot{O}_{ee}$ (reactant)	-401.59785	0.15667	-401.14914	
NO (reactant)	-129.89335	0.00471	-129.76980	
NO_2 (product)	-205.07464	0.00917	-204.88891	
Sum reactants – NO_2	-326.41657	0.15221	-326.03003	21.8
$PIPC^4\dot{O}_{ae}$	-326.42818	0.15004	-326.04462	-22.2
SP_{aa-ea}	-326.42170	0.14882	-326.03637	-3.7
$PIPC^4\dot{O}_{ee}$	-326.42916	0.15038	-326.04559	-23.9
SP_{ae}^{29}	-326.40527	0.14696	-326.02253	27.7
$CHOCH_2CH_2NHCH_2\dot{C}H_2$	-326.41602	0.14534	-326.03146	0.0
$SP1_{ae}^{30b}$	-326.41254	0.15111	-326.02961	20.0
$SKEW_{ae}^{30b}$	-326.42220	0.15068	-326.03882	-5.3
$SP2_{ae}^{30b}$	-326.41012	0.14672	-326.03140	3.8
$POST_{ae}^{30b}$	-326.44494	0.15064	-326.06297	-68.8
SP_{ee}^{29}	-326.40638	0.14725	-326.02367	25.5
$CHOCH_2CH_2NHCH_2\dot{C}H_2$	-326.41602	0.14534	-326.03146	0.0

Table S17, continued.
T_1 and D_1 diagnostic values, vibrational frequencies (cm^{-1}), Rotational constants (GHz) and Cartesian coordinates of the species listed above. Results from CCSD(T*)-F12a/aug-cc-pVTZ//M06-2X/aug-cc-pVTZ calculations.

$PIPC^4O\dot{O}_{aa}$	C	0.891862	1.377965	-0.290709
	N	1.967385	0.394973	-0.357246
T_1= 0.02359876, D_1= 0.14104097	C	1.448940	-0.957650	-0.517515
	C	0.443249	-1.360398	0.564801
$\tilde{\nu}$: 85.9, 154.7, 196.1, 308.8, 333.4, 391.7, 449.9, 513.7, 562.8,	C	-0.669013	-0.334131	0.692042
650.5, 784.8, 799.4, 846.9, 878.2, 903.0, 949.5, 985.5, 1020.1,	C	-0.137821	1.086205	0.802174
1053.9, 1098.0, 1141.1, 1160.4, 1202.0, 1238.9, 1275.9, 1297.7,	O	-1.462418	-0.463578	-0.526628
1340.4, 1356.3, 1358.7, 1368.5, 1387.8, 1395.4, 1411.2, 1465.9,	O	-2.551591	0.236656	-0.471430
1480.9, 1490.1, 1494.8, 1501.6, 3059.1, 3062.6, 3067.7, 3068.8,	H	0.394482	1.387033	-1.263518
3102.7, 3110.8, 3117.6, 3120.2, 3122.1, 3530.7	H	1.325167	2.366492	-0.144847
	H	2.537756	0.450227	0.479049
B: 3.454894 1.698483 1.421722	H	2.283818	-1.657112	-0.532854
	H	0.967357	-1.011395	-1.496389
	H	0.015349	-2.341693	0.354777
	H	0.949235	-1.422009	1.531313

H	-1.349129	-0.567324	1.510880
H	-0.974413	1.783555	0.756202
H	0.327449	1.190848	1.785810

NO

T_1= 0.0203, D_1= 0.0470

$\tilde{\nu}$: 2066.0; B: 0.0000000 52.3561936 52.3561936

N	0.000000	0.000000	-0.606410
O	0.000000	0.000000	0.530609

NO_2

T_1= 0.0251, D_1= 0.0650; $\tilde{\nu}$: 783.5, 1465.2, 1775.4

B: 253.9654019 13.2904639 12.6295376

N	0.000000	0.000000	0.314442
O	0.000000	1.090266	-0.137569
O	0.000000	-1.090266	-0.137569

PIPC^{4}O$\dot{O}_{ea}$

T_1= 0.02364182, D_1= 0.14139103

$\tilde{\nu}$: 87.6, 156.2, 203.6, 315.7, 337.0, 398.3, 446.5, 507.6, 558.1, 658.8, 792.3, 813.4, 845.8, 898.1, 916.3, 933.8, 1020.1, 1058.0, 1069.5, 1108.8, 1145.7, 1172.2, 1195.7, 1219.1, 1270.0, 1298.3, 1325.7, 1358.0, 1359.9, 1371.9, 1380.0, 1397.0, 1435.2, 1464.1, 1478.0, 1487.0, 1506.9, 1518.1, 2985.7, 2989.0, 3078.1, 3079.6, 3106.1, 3112.0, 3113.8, 3127.5, 3134.1, 3569.4

B: 3.484863 1.699231 1.418605

C	-1.451353	-0.959942	-0.494241
N	-1.960248	0.370742	-0.182798
C	-0.902443	1.373101	-0.257933
C	0.144172	1.080711	0.808887
C	0.679096	-0.336129	0.682601
C	-0.429845	-1.365988	0.561853
O	1.464005	-0.456212	-0.543087
O	2.549418	0.250260	-0.494641
H	-0.978926	-1.000027	-1.486159
H	-2.281041	-1.665500	-0.492222
H	-2.713644	0.611146	-0.812275
H	-1.335523	2.357607	-0.087478
H	-0.416660	1.386929	-1.244508
H	0.975978	1.781197	0.745927
H	-0.312127	1.176511	1.795530
H	1.365863	-0.571543	1.495077
H	-0.000671	-2.340860	0.329846
H	-0.926660	-1.433552	1.530672

PIPC$^4\dot{O}_{aa}$

T_1= 0.01680410, D_1= 0.08362481

$\tilde{\nu}$: 156.8, 218.9, 329.4, 395.0, 412.2, 456.2, 511.8, 672.8, 783.5, 812.5, 812.7, 897.1, 900.7, 963.8, 986.0, 1057.2, 1064.8, 1073.5, 1096.5, 1104.4, 1177.9, 1201.5, 1215.4, 1236.7, 1310.2, 1324.0, 1352.9, 1357.7, 1378.1, 1426.5, 1463.4, 1479.2, 1484.9, 1502.9, 1513.9, 2933.0, 2962.9, 2964.1, 3072.8, 3078.4, 3110.4, 3111.5, 3130.9, 3132.4, 3570.0

B: 3.820966 2.791075 2.071097

C	0.913239	-1.205884	0.277929
N	1.664025	-0.000002	-0.051502
C	0.913243	1.205881	0.277930
C	-0.351483	1.262713	-0.570661
C	-1.180006	0.000003	-0.322262
C	-0.351487	-1.262711	-0.570662
O	-1.759257	0.000000	0.925659
H	0.617723	-1.229891	1.339005
H	1.539699	-2.075626	0.084634
H	2.554088	-0.000004	0.427462
H	1.539705	2.075623	0.084637
H	0.617726	1.229888	1.339006
H	-0.945910	2.139295	-0.315697
H	-0.076055	1.314199	-1.625248
H	-2.060158	0.000002	-0.991263
H	-0.945916	-2.139291	-0.315699
H	-0.076059	-1.314196	-1.625249

SP$_{aa-ea}$

T_1= 0.01789621, D_1= 0.09155005

$\tilde{\nu}$: -595.6, 153.0, 210.1, 307.6, 398.3, 402.1, 471.5, 535.2, 677.8, 801.2, 808.4, 894.5, 897.1, 962.0, 987.0, 1028.0, 1050.9, 1060.3, 1094.3, 1109.9, 1167.1, 1212.3, 1237.7, 1254.0, 1306.5,

C	-0.912976	-1.225929	-0.281158
N	-1.634180	0.000009	-0.086385
C	-0.912963	1.225938	-0.281154
C	0.350429	1.267899	0.584031
C	1.170916	-0.000010	0.324671
C	0.350414	-1.267906	0.584028
O	1.744714	-0.000003	-0.923845

1343.5, 1345.6, 1374.9, 1376.2, 1413.4, 1451.0, 1474.0, 1477.9, 1491.1, 1498.2, 2920.7, 2973.8, 2975.4, 3064.5, 3070.4, 3119.6, 3120.3, 3123.1, 3124.7, 3744.2

B: 3.752379 2.820133 2.075121

H	-0.600408	-1.324201	-1.331762
H	-1.563117	-2.069218	-0.055287
H	-2.579230	0.000014	0.234953
H	-1.563093	2.069234	-0.055279
H	-0.600393	1.324211	-1.331758
H	0.955695	2.142106	0.343599
H	0.072085	1.307223	1.638976
H	2.057353	-0.000007	0.986937
H	0.955669	-2.142120	0.343594
H	0.072068	-1.307233	1.638972

PIPC^{4}Ȯ$_{ea}$

T_1= 0.01680410, D_1= 0.08362481

$\tilde{\nu}$: 156.8, 218.9, 329.4, 395.0, 412.2, 456.2, 511.8, 672.8, 783.5, 812.5, 812.7, 897.1, 900.7, 963.8, 986.0, 1057.2, 1064.8, 1073.5, 1096.5, 1104.4, 1177.9, 1201.5, 1215.4, 1236.7, 1310.2, 1324.0, 1352.9, 1357.7, 1378.1, 1426.5, 1463.4, 1479.2, 1484.9, 1502.9, 1513.9, 2933.0, 2962.9, 2964.1, 3072.8, 3078.4, 3110.4, 3111.5, 3130.9, 3132.4, 3570.

B: 3.820966 2.791075 2.071097

C	0.913239	-1.205884	0.277929
N	1.664025	-0.000002	-0.051502
C	0.913243	1.205881	0.277930
C	-0.351483	1.262713	-0.570661
C	-1.180006	0.000003	-0.322262
C	-0.351487	-1.262711	-0.570662
O	-1.759257	0.000000	0.925659
H	0.617723	-1.229891	1.339005
H	1.539699	-2.075626	0.084634
H	2.554088	-0.000004	0.427462
H	1.539705	2.075623	0.084637
H	0.617726	1.229888	1.339006
H	-0.945910	2.139295	-0.315697
H	-0.076055	1.314199	-1.625248
H	-2.060158	0.000002	-0.991263
H	-0.945916	-2.139291	-0.315699
H	-0.076059	-1.314196	-1.625249

SP$_{aa}^{29}$

T_1= 0.01847171, D_1= 0.08471638

$\tilde{\nu}$: -472.1, 154.2, 211.1, 260.0, 359.6, 372.9, 401.9, 518.8, 628.0, 648.7, 763.0, 803.8, 842.1, 864.2, 890.6, 909.4, 974.6, 1031.1, 1054.5, 1067.7, 1127.4, 1186.9, 1215.1, 1253.6, 1328.7, 1359.6, 1370.1, 1387.3, 1394.7, 1459.2, 1463.4, 1468.3, 1489.7, 1499.4, 1601.2, 2950.7, 3020.6, 3049.8, 3076.3, 3095.5, 3097.4, 3122.1, 3124.3, 3227.4, 3537.8

B: 3.579276 2.679043 1.967569

C	0.614417	1.356034	-0.296981
N	1.659992	0.357591	-0.120007
C	1.194283	-1.002788	-0.332863
C	0.123311	-1.468223	0.604907
C	-1.401557	-0.079765	0.265988
C	-0.606914	1.173139	0.605056
O	-1.716479	-0.337531	-0.899942
H	0.285182	1.314104	-1.336527
H	1.052659	2.339398	-0.131579
H	2.080040	0.445254	0.797992
H	2.053138	-1.678883	-0.250695
H	0.827529	-1.081366	-1.357927
H	-0.405233	-2.379381	0.355821
H	0.272704	-1.293102	1.666027
H	-1.988398	-0.512414	1.094639
H	-1.292305	2.014666	0.467874
H	-0.314665	1.158458	1.657318

CHOCH$_2$CH$_2$NHCH$_2$ĊH$_2$

T_1= 0.01288593, D_1= 0.04648321

$\tilde{\nu}$: 66.7, 108.9, 115.4, 195.0, 208.3, 234.0, 339.1, 378.0, 488.3, 545.7, 665.2, 726.0, 756.9, 852.1, 876.0, 897.1, 969.4, 980.1, 1068.0, 1078.2, 1163.5, 1179.1, 1248.9, 1257.8, 1324.9, 1363.6, 1392.5, 1395.0, 1423.9, 1442.0, 1460.7, 1472.2, 1485.8, 1513.5,

C	1.654359	-0.603859	0.250771
O	1.761927	-0.677793	-0.944945
C	0.928950	0.497355	0.968675
C	0.120749	1.408878	0.042616
N	-0.908422	0.778663	-0.766596
C	-1.890139	-0.011224	-0.034985
C	-1.477238	-1.401071	0.323152
H	2.114925	-1.376011	0.896093
H	1.685993	1.078970	1.507359

1847.7, 2945.4, 2984.2, 3042.2, 3061.1, 3062.8, 3082.7, 3118.0, 3155.4, 3258.6, 3565.9

B: 3.675572 2.045765 1.744851

H	0.307169	0.037138	1.741239
H	0.805470	1.911510	-0.639977
H	-0.350370	2.184721	0.650208
H	-0.473924	0.219326	-1.489662
H	-2.803901	-0.056468	-0.643310
H	-2.169817	0.545159	0.864336
H	-1.973615	-1.933621	1.122052
H	-0.898482	-1.979497	-0.383983

SP_{aa}^{30a}

T_1= 0.03594232, D_1= 0.21245499

$\tilde{\nu}$: -1404.0, 244.2, 301.8, 382.3, 426.8, 496.9, 565.5, 597.3, 712.3, 777.9, 839.0, 888.9, 903.3, 924.3, 992.5, 1010.7, 1058.9, 1081.5, 1090.6, 1115.4, 1142.5, 1199.8, 1212.2, 1243.5, 1280.9, 1311.2, 1316.9, 1337.6, 1364.2, 1374.5, 1430.6, 1471.0, 1489.6, 1494.8, 1507.6, 1839.0, 3021.8, 3040.7, 3069.5, 3084.2, 3118.0, 3123.3, 3143.9, 3150.7, 3594.7

B: 3.68141 3.09385 2.36161

C	-0.839195	-1.183404	-0.138573
N	-1.640333	-0.046937	0.089506
C	-0.953584	1.232013	-0.098136
C	0.414372	1.232994	0.579550
C	1.153146	-0.065552	0.233690
C	0.360803	-1.240709	0.805006
O	1.170619	-0.298917	-1.146559
H	-0.110696	-0.965832	-1.122294
H	-1.428953	-2.087098	-0.260120
H	-2.528344	-0.077758	-0.389364
H	-1.586404	2.031354	0.283200
H	-0.790172	1.398068	-1.169182
H	0.978312	2.094323	0.222204
H	0.310072	1.318791	1.663086
H	2.187822	-0.035189	0.589818
H	0.905662	-2.170674	0.654524
H	0.078795	-1.135479	1.853115

$POST_{aa}^{30a}$

T_1= 0.01371614, D_1= 0.05631066

$\tilde{\nu}$: 169.9, 200.5, 285.7, 290.4, 393.9, 408.0, 501.3, 515.7, 702.1, 802.8, 833.3, 875.9, 892.6, 975.8, 991.8, 1005.6, 1051.7, 1076.9, 1114.9, 1128.9, 1155.2, 1223.8, 1238.9, 1273.3, 1300.7, 1318.9, 1329.9, 1364.1, 1386.7, 1392.5, 1430.6, 1471.6, 1488.1, 1491.2, 1515.6, 3006.8, 3016.0, 3029.3, 3059.1, 3077.8, 3103.6, 3112.1, 3114.1, 3127.6, 3878.6

B: 3.805483 2.790745 2.109368

C	-1.016698	-1.156803	-0.325773
N	-1.758265	0.046949	-0.040877
C	-0.952240	1.211465	-0.310452
C	0.278907	1.232521	0.609596
C	1.101997	-0.039029	0.437084
C	0.211140	-1.259978	0.593205
H	-0.667295	-1.146915	-1.365918
H	-1.663744	-2.020895	-0.184630
H	-1.550155	2.108911	-0.159818
H	-0.606263	1.193439	-1.352263
H	0.886364	2.115157	0.395656
H	-0.049052	1.305757	1.648437
H	1.894531	-0.069508	1.191897
H	0.775668	-2.161628	0.357186
H	-0.121045	-1.326612	1.630426
O	1.679091	-0.113736	-0.861708
H	2.237484	0.654482	-1.003137

SP_{ea}^{29}

T_1= 0.01844113, D_1= 0.08460941

$\tilde{\nu}$: -472.2, 154.7, 219.1, 264.7, 368.5, 380.1, 401.7, 514.0, 633.2, 658.6, 782.0, 823.6, 842.9, 868.4, 910.3, 928.1, 987.9, 1036.3, 1082.1, 1095.3, 1132.9, 1190.2, 1218.1, 1231.8, 1313.4, 1356.8, 1370.1, 1386.6, 1420.2, 1449.6, 1467.4, 1476.1, 1496.5, 1504.5, 1601.7, 2954.8, 2999.3, 3010.7, 3040.5, 3059.5, 3112.2, 3118.0, 3138.3, 3246.0, 3573.9

C	0.632725	1.346366	-0.268886
N	1.624671	0.322973	0.048818
C	1.179385	-1.017997	-0.306357
C	0.091768	-1.471632	0.605128
C	-1.408499	-0.061862	0.245288
C	-0.602669	1.179887	0.602833
O	-1.707035	-0.314435	-0.926224
H	0.320664	1.310145	-1.320568
H	1.081583	2.320611	-0.080440
H	2.491965	0.530345	-0.426625
H	2.030051	-1.700219	-0.199422

B: 3.602894 2.691501 1.969174

H	0.825990	-1.090648	-1.343712
H	-0.448929	-2.367873	0.334157
H	0.235997	-1.296040	1.665107
H	-2.015823	-0.485481	1.063626
H	-1.274050	2.029595	0.452296
H	-0.320125	1.155666	1.655611

SP_{ea}^{30a}

T_1= 0.03594228, D_1= 0.21245472

$\tilde{\nu}$: -1404.0, 244.2, 301.8, 382.3, 426.8, 496.9, 565.5, 597.3, 712.3, 777.9, 839.0, 888.9, 903.3, 924.3, 992.5, 1010.7, 1058.9, 1081.5, 1090.6, 1115.4, 1142.5, 1199.8, 1212.2, 1243.5, 1280.9, 1311.2, 1316.9, 1337.6, 1364.2, 1374.5, 1430.6, 1471.0, 1489.6, 1494.8, 1507.6, 1839.0, 3021.8, 3040.7, 3069.5, 3084.2, 3118.0, 3123.3, 3143.9, 3150.7, 3594.7

B: 3.681261 3.093775 2.361652

C	0.224078	-1.363056	0.159402
N	1.434560	-0.665436	-0.023285
C	1.343735	0.780354	0.186600
C	0.121753	1.368358	-0.515837
C	-1.103337	0.495286	-0.218415
C	-0.862785	-0.894200	-0.806905
O	-1.254801	0.267372	1.154472
H	-0.372071	-0.875908	1.135367
H	0.375939	-2.432916	0.266846
H	2.213460	-1.074848	0.469146
H	2.263870	1.245819	-0.160959
H	1.236297	0.981164	1.258545
H	-0.037716	2.380557	-0.146289
H	0.281840	1.421231	-1.594588
H	-2.019334	0.964124	-0.591927
H	-1.751825	-1.511074	-0.691580
H	-0.534632	-0.899527	-1.846408

$POST_{ea}^{30a}$

T_1= 0.01541265, D_1= 0.06856767

$\tilde{\nu}$: 155.2, 224.2, 330.2, 382.4, 395.7, 399.8, 494.9, 526.6, 617.8, 679.2, 769.1, 826.6, 883.7, 913.9, 918.5, 1006.8, 1039.9, 1070.7, 1102.1, 1128.7, 1171.7, 1208.8, 1233.1, 1267.7, 1309.6, 1348.1, 1358.0, 1382.7, 1398.1, 1421.7, 1459.3, 1462.8, 1478.5, 1504.0, 1524.1, 2976.4, 3006.7, 3066.2, 3093.0, 3110.9, 3115.2, 3123.6, 3210.2, 3612.4, 3812.2

B: 3.765591 2.793237 2.114610

C	0.856497	1.268920	-0.221480
N	1.660056	0.143747	-0.108884
C	1.013638	-1.161608	-0.223469
C	-0.221898	-1.219371	0.662114
C	-1.160044	-0.049405	0.365925
C	-0.408781	1.264827	0.574096
O	-1.683658	-0.134565	-0.949993
H	-1.055613	0.290339	-1.544892
H	1.378972	2.198331	-0.395899
H	2.529026	0.206081	-0.615643
H	1.728439	-1.929243	0.068257
H	0.717588	-1.355308	-1.262109
H	-0.746598	-2.159159	0.492427
H	0.077575	-1.176916	1.711785
H	-2.021139	-0.095422	1.032651
H	-1.043150	2.102292	0.285074
H	-0.192702	1.369117	1.647361

$SP1_{ea}^{30b}$

T_1= 0.01735840, D_1= 0.08805018

$\tilde{\nu}$: -258.8, 79.2, 201.0, 335.0, 373.3, 465.5, 537.6, 603.3, 749.6, 795.5, 808.8, 857.7, 895.1, 953.3, 982.7, 1029.8, 1076.8, 1085.3, 1120.5, 1167.0, 1213.0, 1249.0, 1268.5, 1289.3, 1314.3, 1330.2, 1353.9, 1384.2, 1408.5, 1440.6, 1482.6, 1498.9, 1512.7, 1529.1, 1533.4, 3008.7, 3030.4, 3040.8, 3063.4, 3076.5, 3085.4, 3089.2, 3117.7, 3135.0, 3638.8

B: 3.468131 2.947633 2.202140

C	-0.955099	1.285484	-0.050068
N	-1.571003	0.001542	0.290971
C	-0.913835	-1.285533	0.050821
C	0.386371	-1.181918	-0.729726
C	1.247794	-0.049180	-0.178003
C	0.516236	1.275201	-0.503964
O	1.383496	-0.082684	1.181338
H	-1.038126	1.929869	0.824453
H	-1.534775	1.770068	-0.840630
H	-1.898346	0.033253	1.240458
H	-1.594053	-1.953944	-0.482871
H	-0.685463	-1.756686	1.008754
H	0.915905	-2.132355	-0.668281

H	0.200470	-0.980468	-1.787207
H	2.229946	-0.006741	-0.664630
H	1.055518	2.112413	-0.064636
H	0.589181	1.370949	-1.587270

SKEW$_{ea}^{30b}$

T_1= 0.02089348, D_1= 0.11407283

$\tilde{\nu}$: 102.3, 170.4, 298.4, 363.7, 377.1, 538.6, 542.4, 734.7, 765.5, 815.3, 831.2, 870.2, 894.2, 961.7, 973.7, 991.6, 1047.8, 1070.6, 1088.7, 1125.0, 1160.7, 1189.5, 1210.5, 1266.5, 1284.9, 1324.3, 1342.9, 1348.2, 1361.1, 1389.3, 1476.9, 1485.3, 1488.0, 1497.2, 1508.8, 2939.4, 3059.2, 3060.7, 3073.5, 3077.3, 3114.1, 3121.8, 3132.5, 3139.7, 3524.8

B: 3.478421 3.021595 2.350375

C	-1.094581	1.131345	-0.124788
N	-1.318077	-0.052377	0.688095
C	-0.925352	-1.266933	-0.019716
C	0.379302	-1.101760	-0.838546
C	1.161834	0.058971	-0.230432
C	0.404379	1.375097	-0.434392
O	1.445453	-0.163234	1.094998
H	-1.527049	1.998122	0.371809
H	-1.647690	0.987258	-1.055715
H	-0.820611	0.021366	1.568782
H	-1.741332	-1.556617	-0.685041
H	-0.806650	-2.059121	0.716075
H	0.967014	-2.017872	-0.815067
H	0.161604	-0.869615	-1.882905
H	2.161721	0.137375	-0.693099
H	0.825171	2.118124	0.242202
H	0.537249	1.733172	-1.456447

SP2$_{ea}^{30b}$

T_1= 0.02136921, D_1= 0.11570461

$\tilde{\nu}$: -1399.4, 61.3, 300.9, 404.5, 409.7, 552.4, 571.0, 664.2, 813.4, 825.2, 853.2, 874.4, 893.1, 933.9, 958.9, 1001.7, 1053.4, 1057.0, 1093.3, 1142.5, 1174.5, 1203.9, 1218.6, 1266.9, 1279.1, 1295.8, 1310.1, 1323.2, 1336.5, 1364.4, 1397.0, 1479.2, 1493.0, 1496.3, 1517.6, 1889.2, 3026.2, 3064.4, 3070.5, 3084.2, 3090.2, 3129.7, 3131.0, 3147.7, 3150.4

B: 3.384162 3.314946 2.618632

C	1.191454	0.987568	-0.129192
N	0.000001	1.174768	0.684990
C	-1.191452	0.987570	-0.129192
C	-1.244206	-0.470780	-0.690428
C	-0.000001	-1.159774	-0.137383
C	1.244205	-0.470782	-0.690428
O	-0.000001	-1.048089	1.250177
H	2.065010	1.194908	0.483231
H	1.162760	1.713023	-0.944767
H	0.000000	0.256596	1.391333
H	-1.162756	1.713025	-0.944768
H	-2.065008	1.194912	0.483230
H	-2.138631	-0.973076	-0.327698
H	-1.255188	-0.490232	-1.780429
H	-0.000002	-2.227318	-0.388616
H	2.138630	-0.973080	-0.327700
H	1.255186	-0.490234	-1.780430

POST$_{ea}^{30b}$ same as POST$_{aa}^{30b}$

PIPC^{4}O$\dot{\mathrm{O}}_{ae}$

T_1= 0.02371841, D_1= 0.14156750

$\tilde{\nu}$: 124.0, 131.8, 234.1, 310.4, 327.8, 358.7, 421.2, 479.1, 557.9, 682.2, 775.7, 820.9, 836.6, 911.0, 917.5, 967.1, 980.1, 1029.4, 1047.8, 1119.0, 1137.3, 1180.2, 1202.9, 1251.4, 1253.7, 1289.3, 1324.1, 1329.5, 1360.7, 1370.7, 1389.2, 1397.2, 1417.2, 1477.6, 1491.8, 1498.6, 1499.2, 1505.3, 3046.6, 3048.4, 3069.1, 3069.8, 3081.6, 3115.6, 3117.1, 3121.7, 3125.3, 3538.8

B: 3.946766 1.507928 1.258818

C	-1.502203	1.206650	0.116170
N	-2.100416	-0.000000	-0.437724
C	-1.502203	-1.206650	0.116171
C	0.023975	-1.254807	-0.017421
C	0.617788	0.000000	0.591875
C	0.023975	1.254807	-0.017423
O	2.068946	-0.000000	0.472743
O	2.461492	0.000000	-0.763406
H	-1.776210	1.253442	1.174516
H	-1.948944	2.075411	-0.364903
H	-2.003370	-0.000001	-1.446417
H	-1.948944	-2.075411	-0.364900
H	-1.776210	-1.253440	1.174518

H	0.429861	-2.139987	0.474349
H	0.304227	-1.293657	-1.071686
H	0.476909	0.000001	1.675000
H	0.429861	2.139988	0.474345
H	0.304228	1.293655	-1.071687

PIPC^{4}O$\dot{O}_{ee}$

T_1= 0.02362667, D_1= 0.14109890

$\tilde{\nu}$: 120.7, 134.7, 246.7, 309.6, 329.4, 370.8, 415.4, 471.3, 545.9, 669.9, 784.8, 836.2, 850.8, 904.6, 931.8, 966.2, 1003.3, 1054.0, 1075.0, 1136.8, 1159.4, 1167.2, 1193.2, 1234.6, 1249.8, 1296.3, 1311.7, 1331.3, 1355.4, 1358.9, 1390.4, 1416.3, 1429.5, 1479.2, 1485.6, 1499.3, 1506.1, 1516.2, 2955.2, 2955.4, 3079.8, 3082.1, 3083.3, 3112.7, 3113.7, 3136.5, 3139.9, 3574.0

B: 3.967633 1.518174 1.263691

C	-1.491588	1.206679	0.108174
N	-1.995245	-0.000001	-0.531985
C	-1.491587	-1.206680	0.108175
C	0.026772	-1.255721	-0.025734
C	0.618758	0.000001	0.585753
C	0.026771	1.255721	-0.025735
O	2.068533	0.000002	0.474230
O	2.468559	-0.000002	-0.759892
H	-1.758952	1.249585	1.176636
H	-1.934115	2.075621	-0.376403
H	-3.005683	-0.000001	-0.544499
H	-1.934113	-2.075622	-0.376402
H	-1.758951	-1.249585	1.176637
H	0.428923	-2.138894	0.471057
H	0.298732	-1.291833	-1.080425
H	0.471720	0.000001	1.668162
H	0.428921	2.138896	0.471055
H	0.298731	1.291833	-1.080426

PIPC$^4\dot{O}_{ae}$

T_1= 0.01605331, D_1= 0.07709925

$\tilde{\nu}$: 157.9, 229.2, 254.7, 346.5, 409.6, 442.6, 466.7, 594.3, 765.0, 806.8, 814.1, 860.9, 905.6, 917.8, 993.2, 1026.9, 1057.7, 1063.1, 1114.8, 1148.6, 1163.4, 1221.2, 1243.5, 1248.2, 1281.7, 1339.1, 1339.2, 1355.4, 1373.8, 1398.1, 1471.2, 1486.9, 1490.8, 1494.7, 1499.7, 2895.7, 3038.6, 3042.0, 3053.5, 3056.6, 3112.1, 3113.2, 3114.6, 3115.7, 3535.9

B: 4.522697 2.349344 1.690528

C	-1.119411	1.207349	0.216023
N	-1.810438	0.000007	-0.215676
C	-1.119423	-1.207342	0.216024
C	0.353181	-1.262041	-0.200605
C	1.081817	-0.000009	0.259576
C	0.353194	1.262036	-0.200603
O	2.414452	-0.000003	-0.062996
H	-1.184413	1.248067	1.307994
H	-1.656008	2.075079	-0.165645
H	-1.906774	0.000007	-1.224476
H	-1.656030	-2.075067	-0.165641
H	-1.184425	-1.248057	1.307995
H	0.847331	-2.142492	0.211258
H	0.426345	-1.318904	-1.290171
H	1.101559	-0.000000	1.368807
H	0.847354	2.142479	0.211263
H	0.426360	1.318900	-1.290169

SP$_{ae-ee}$

T_1= 0.01633066, D_1= 0.07856220

$\tilde{\nu}$: -596.3, 159.3, 237.5, 276.2, 325.1, 410.6, 445.1, 479.6, 613.7, 811.2, 813.5, 878.9, 908.6, 926.5, 994.2, 1031.9, 1063.2, 1079.1, 1126.1, 1146.9, 1178.1, 1224.4, 1240.4, 1263.5, 1280.9, 1332.6, 1343.8, 1371.4, 1372.0, 1415.2, 1455.4, 1480.7, 1488.1, 1491.7, 1500.8, 2884.3, 2958.4, 2959.1, 3063.5, 3066.4, 3118.9, 3120.1, 3121.2, 3122.1, 3744.1

B: 4.487913 2.377385 1.689494

C	1.112720	-1.226473	0.202308
N	1.737745	0.000008	-0.200293
C	1.112706	1.226481	0.202308
C	-0.358490	1.267188	-0.221705
C	-1.073318	-0.000010	0.250573
C	-0.358475	-1.267195	-0.221706
O	-2.412312	-0.000003	-0.042507
H	1.156465	-1.339722	1.297133
H	1.655800	-2.066937	-0.226142
H	2.543793	0.000013	-0.789199
H	1.655775	2.066952	-0.226146
H	1.156449	1.339733	1.297132
H	-0.863012	2.141866	0.189617

H	-0.424795	1.310946	-1.310819
H	-1.069292	0.000000	1.361071
H	-0.862985	-2.141881	0.189613
H	-0.424779	-1.310952	-1.310820

PIPC$^{4}\dot{O}_{ee}$

T_1= 0.01611192, D_1= 0.07759684

$\tilde{\nu}$: 161.3, 243.4, 287.9, 359.5, 404.4, 442.6, 477.8, 576.9, 785.1, 816.7, 818.9, 895.8, 904.6, 938.1, 1003.5, 1060.4, 1066.9, 1076.7, 1136.7, 1151.7, 1181.1, 1202.3, 1235.8, 1241.4, 1284.2, 1321.6, 1339.4, 1350.4, 1374.0, 1424.6, 1473.6, 1484.3, 1489.1, 1502.1, 1513.8, 2890.1, 2947.8, 2949.5, 3072.0, 3074.5, 3109.5, 3110.7, 3127.3, 3128.8, 3573.2

B: 4.550546 2.369311 1.697136

C	-1.111419	1.205972	0.201421
N	-1.720493	0.000004	-0.341658
C	-1.111427	-1.205967	0.201421
C	0.356464	-1.263888	-0.203772
C	1.081732	-0.000006	0.258762
C	0.356473	1.263884	-0.203771
O	2.415862	-0.000002	-0.052580
H	-1.175891	1.238119	1.301801
H	-1.642828	2.074766	-0.184768
H	-2.716960	0.000008	-0.173393
H	-1.642843	-2.074758	-0.184765
H	-1.175899	-1.238112	1.301801
H	0.844801	-2.140678	0.220557
H	0.428320	-1.322330	-1.291235
H	1.093768	0.000000	1.368560
H	0.844816	2.140670	0.220559
H	0.428330	1.322326	-1.291234

SP$^{29}_{ae}$

T_1= 0.01832200, D_1= 0.08309283

$\tilde{\nu}$: -456.0, 138.5, 207.1, 292.1, 322.4, 364.0, 407.4, 485.3, 575.2, 643.6, 751.1, 795.3, 824.1, 859.1, 897.5, 918.3, 995.3, 1039.0, 1055.6, 1075.1, 1148.2, 1194.3, 1215.4, 1253.4, 1313.9, 1356.8, 1367.0, 1389.4, 1399.5, 1454.7, 1465.7, 1469.0, 1488.9, 1498.2, 1614.1, 2912.3, 3019.3, 3045.0, 3047.6, 3075.2, 3101.6, 3113.8, 3140.0, 3239.3, 3540.7

B: 4.141556 2.278345 1.617422

C	0.969371	-1.289638	0.207785
N	1.795467	-0.168292	-0.214613
C	1.292924	1.115107	0.244135
C	-0.076211	1.481564	-0.244274
C	-1.299669	-0.119083	0.364184
C	-0.489682	-1.249057	-0.243826
O	-2.388251	0.227120	-0.095167
H	1.005323	-1.327238	1.301074
H	1.436256	-2.206820	-0.149290
H	1.895351	-0.165819	-1.222816
H	1.998801	1.893354	-0.069040
H	1.310284	1.113144	1.336986
H	-0.603807	2.288397	0.247723
H	-0.269463	1.393940	-1.307033
H	-1.055852	0.105281	1.421847
H	-0.985941	-2.174142	0.066955
H	-0.573613	-1.192369	-1.330803

SP1$^{30b}_{ae}$

T_1= 0.01713796 , D_1= 0.08544628

$\tilde{\nu}$: -152.4, 58.4, 327.1, 368.4, 393.3, 473.3, 496.6, 650.7, 750.9, 789.2, 803.2, 883.6, 900.8, 966.7, 992.0, 998.0, 1059.9, 1114.0, 1166.1, 1169.0, 1205.9, 1242.0, 1270.3, 1286.3, 1335.2, 1347.8, 1356.6, 1373.6, 1396.9, 1402.0, 1479.1, 1485.3, 1498.9, 1499.9, 1512.1, 3012.2, 3056.5, 3060.0, 3066.3, 3077.5, 3105.0, 3108.8, 3128.3, 3129.6, 3530.8

B: 3.915890 2.578079 1.873112

C	-1.058221	1.206666	0.254513
N	-1.568118	0.140646	-0.587284
C	-1.218002	-1.155790	-0.025270
C	0.262479	-1.332619	0.390005
C	1.168919	-0.080315	0.377050
C	0.462979	1.309865	0.204523
O	2.039891	-0.087870	-0.687724
H	-1.399975	1.010928	1.273940
H	-1.490740	2.159624	-0.051576
H	-1.194049	0.234209	-1.525113
H	-1.500018	-1.926628	-0.739898
H	-1.855287	-1.296216	0.849971
H	0.302051	-1.765904	1.389716
H	0.748837	-2.049491	-0.271116
H	1.742160	-0.039738	1.309680

H	0.854689	2.002101	0.947493
H	0.741109	1.702710	-0.775238

$SKEW_{ae}^{30b}$

T_1= 0.02089349 , D_1= 0.11407292

$\tilde{\nu}$: 102.3, 170.4, 298.4, 363.7, 377.1, 538.6, 542.4, 734.7, 765.5, 815.3, 831.2, 870.2, 894.2, 961.7, 973.7, 991.6, 1047.8, 1070.6, 1088.7, 1125.0, 1160.7, 1189.5, 1210.5, 1266.5, 1284.9, 1324.3, 1342.9, 1348.2, 1361.1, 1389.3, 1476.9, 1485.3, 1488.0, 1497.2, 1508.8, 2939.4, 3059.2, 3060.7, 3073.5, 3077.3, 3114.1, 3121.8, 3132.5, 3139.7, 3524.8

B: 3.478421 3.021595 2.350376

C	1.094580	1.131346	-0.124788
N	1.318077	-0.052376	0.688095
C	0.925354	-1.266932	-0.019717
C	-0.379302	-1.101761	-0.838544
C	-1.161834	0.058969	-0.230432
C	-0.404380	1.375096	-0.434394
O	-1.445452	-0.163233	1.094999
H	1.647691	0.987261	-1.055714
H	1.527046	1.998124	0.371811
H	0.820610	0.021366	1.568781
H	0.806655	-2.059121	0.716073
H	1.741334	-1.556613	-0.685043
H	-0.161606	-0.869618	-1.882905
H	-0.967013	-2.017874	-0.815063
H	-2.161722	0.137372	-0.693097
H	-0.537250	1.733168	-1.456450
H	-0.825174	2.118125	0.242197

$SP2_{ae}^{30b}$ same as $SP2_{ea}^{30b}$

$POST_{ae}^{30b}$ same as $POST_{ea}^{30b}$

SP_{ee}^{29}

T_1= 0.01836209, D_1= 0.08375905

$\tilde{\nu}$: -460.0, 144.7, 219.8, 293.7, 330.5, 381.1, 405.8, 488.5, 562.2, 655.9, 763.2, 822.4, 844.4, 870.4, 909.1, 932.6, 991.0, 1045.5, 1094.0, 1111.4, 1136.5, 1197.1, 1215.6, 1231.1, 1304.5, 1349.7, 1359.0, 1392.5, 1423.9, 1451.3, 1461.5, 1479.7, 1497.5, 1505.0, 1612.7, 2908.9, 2957.6, 2970.3, 3032.0, 3055.7, 3112.0, 3124.0, 3154.3, 3256.8, 3577.7

B 4.176019 2.295181 1.624349

C	0.965983	-1.282559	0.193971
N	1.710452	-0.147750	-0.334519
C	1.277039	1.118622	0.232204
C	-0.085752	1.480976	-0.254582
C	-1.295232	-0.119646	0.363929
C	-0.487849	-1.249874	-0.250051
O	-2.394195	0.213778	-0.080077
H	0.999531	-1.316261	1.295257
H	1.434214	-2.195215	-0.171804
H	2.699883	-0.277125	-0.176011
H	1.979283	1.896998	-0.087660
H	1.286249	1.115172	1.333082
H	-0.611310	2.283286	0.244564
H	-0.268012	1.386762	-1.316998
H	-1.039105	0.109480	1.417889
H	-0.980629	-2.173982	0.066558
H	-0.564838	-1.190196	-1.335460

Experimental Results

Piperidine + OH reaction kinetics

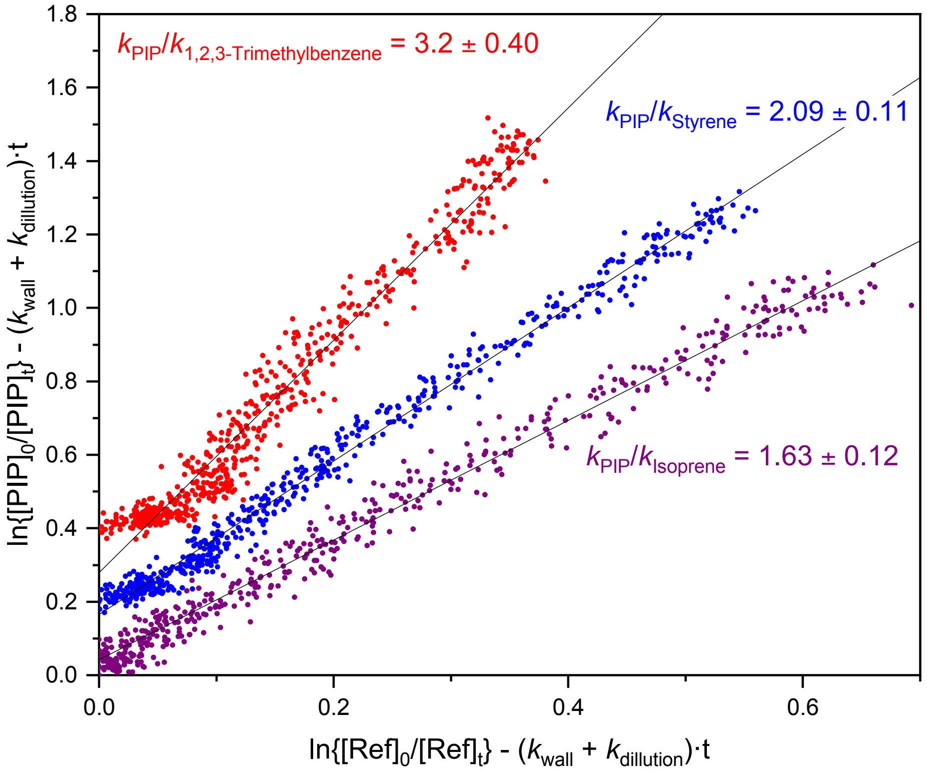

Figure S12. Piperidine + OH relative rate plot, 2016.07.19 experiment 2.
Relative rate plots showing the decays of piperidine, styrene, isoprene and 1,2,3-trimethylbenzene in the presence OH radicals. For the sake of clarity, the data have been displaced along the abscissa. The data have been corrected for dilution due to chamber air replenishment and wall loss. Uncertainties given are 3 times the standard error of the least squares fit.

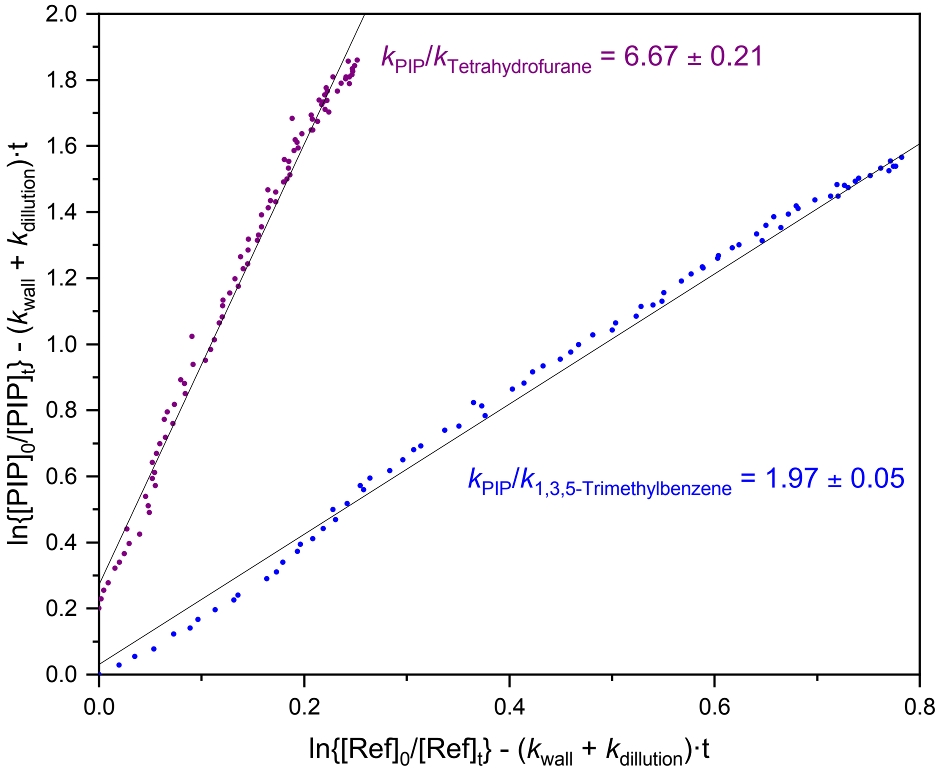

Figure S13. Piperidine + OH relative rate plot, 2011.06.08.
Relative rate plots showing the decays of piperidine, tetrahydrofurane and 1,3,5-trimethylbenzene in the presence OH radicals. For the sake of clarity, the data have been displaced along the abscissa. The data have been corrected for dilution due to chamber air replenishment and wall loss. Uncertainties given are 3 times the standard error of the least squares fit.

Table S18. Summary of relative rate results for the piperidine + OH reaction.

Reference compound	k_{rel}	k_{Ref} /10^{-11} cm^3 molecule^{-1} s^{-1}	k_{OH} /10^{-10} cm^3 molecule^{-1} s^{-1}
Tetrahydrofurane	6.67 ± 0.21	1.70 ± 0.26	1.13 ± 0.17
1,3,5-trimethylbenzene	1.97 ± 0.05	5.86 ± 0.88	1.15 ± 0.18
1,2,3-trimethylbenzene	3.61 ± 0.20	3.27 ± 0.82	1.18 ± 0.30
1,2,3-trimethylbenzene	3.20 ± 0.40	3.27 ± 0.82	1.05 ± 0.29
Styrene	2.25 ± 0.04	5.80 ± 0.58	1.31 ± 0.28
Styrene	2.24 ± 0.04	5.80 ± 0.58	1.30 ± 0.27
Isoprene	1.82 ± 0.04	10.0 ± 1.5	1.82 ± 0.28*
Isoprene	1.63 ± 0.12	10.0 ± 1.5	1.63 ± 0.27*
Average			1.19 ± 0.27

* Not included in the average value.

1-Nitrosopiperidine photolysis studies

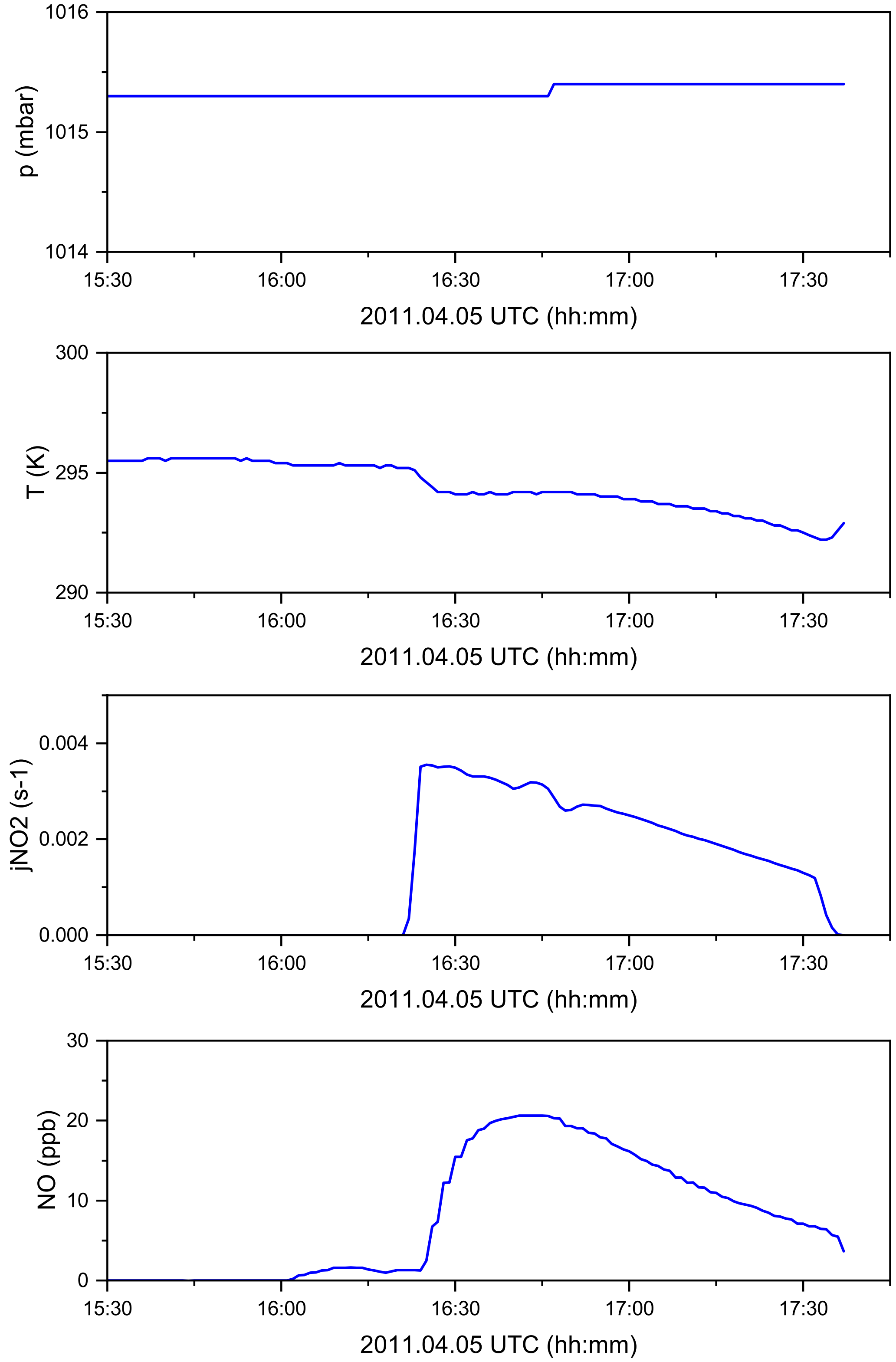

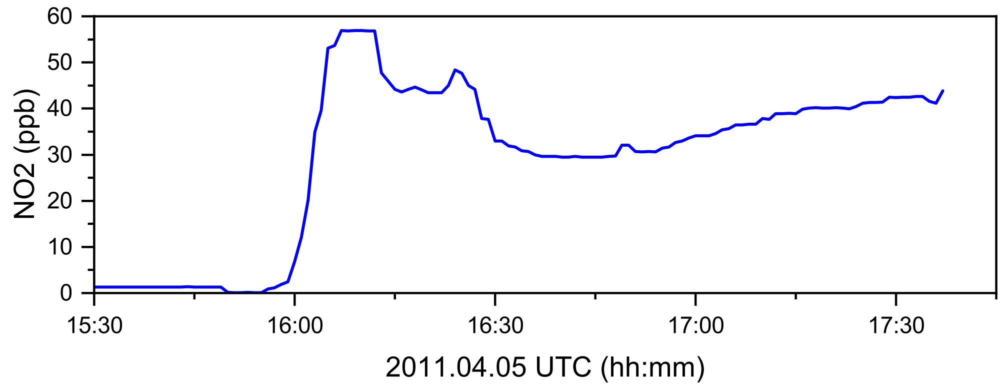

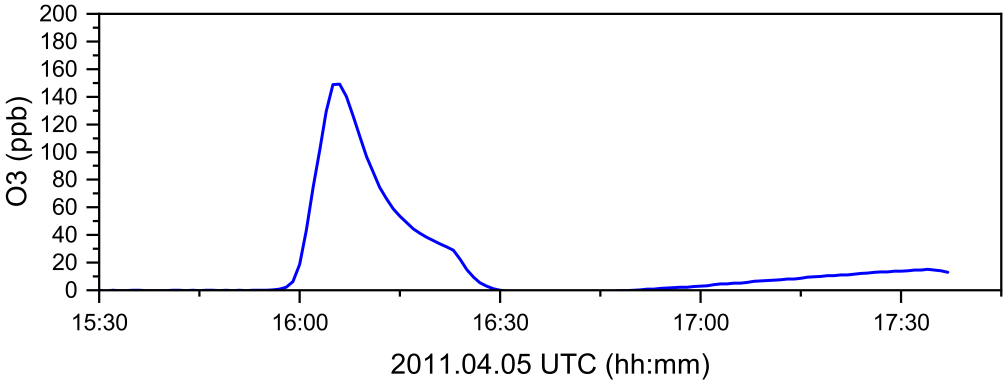

Figure S14. p, T, j_{NO2}, NO, NO_2 and O_3 during the PIP-NO photolysis experiment on 2011.04.05.

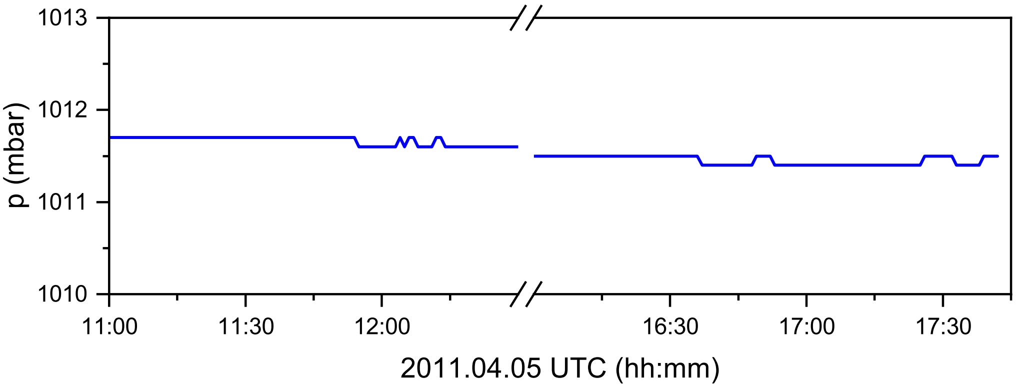

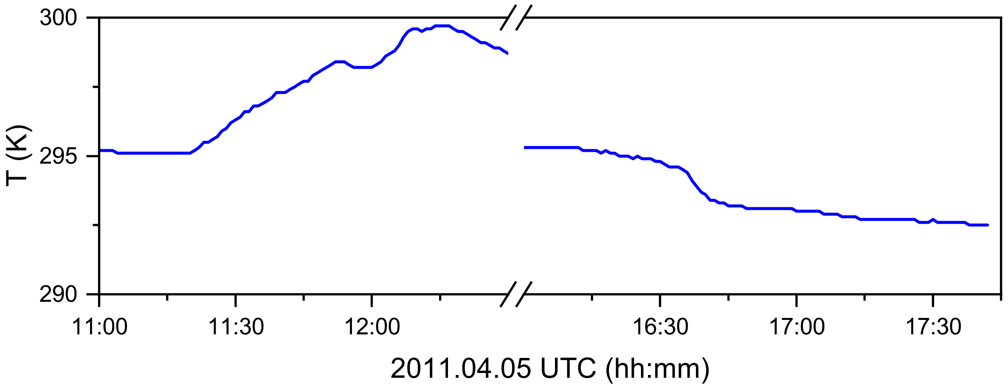

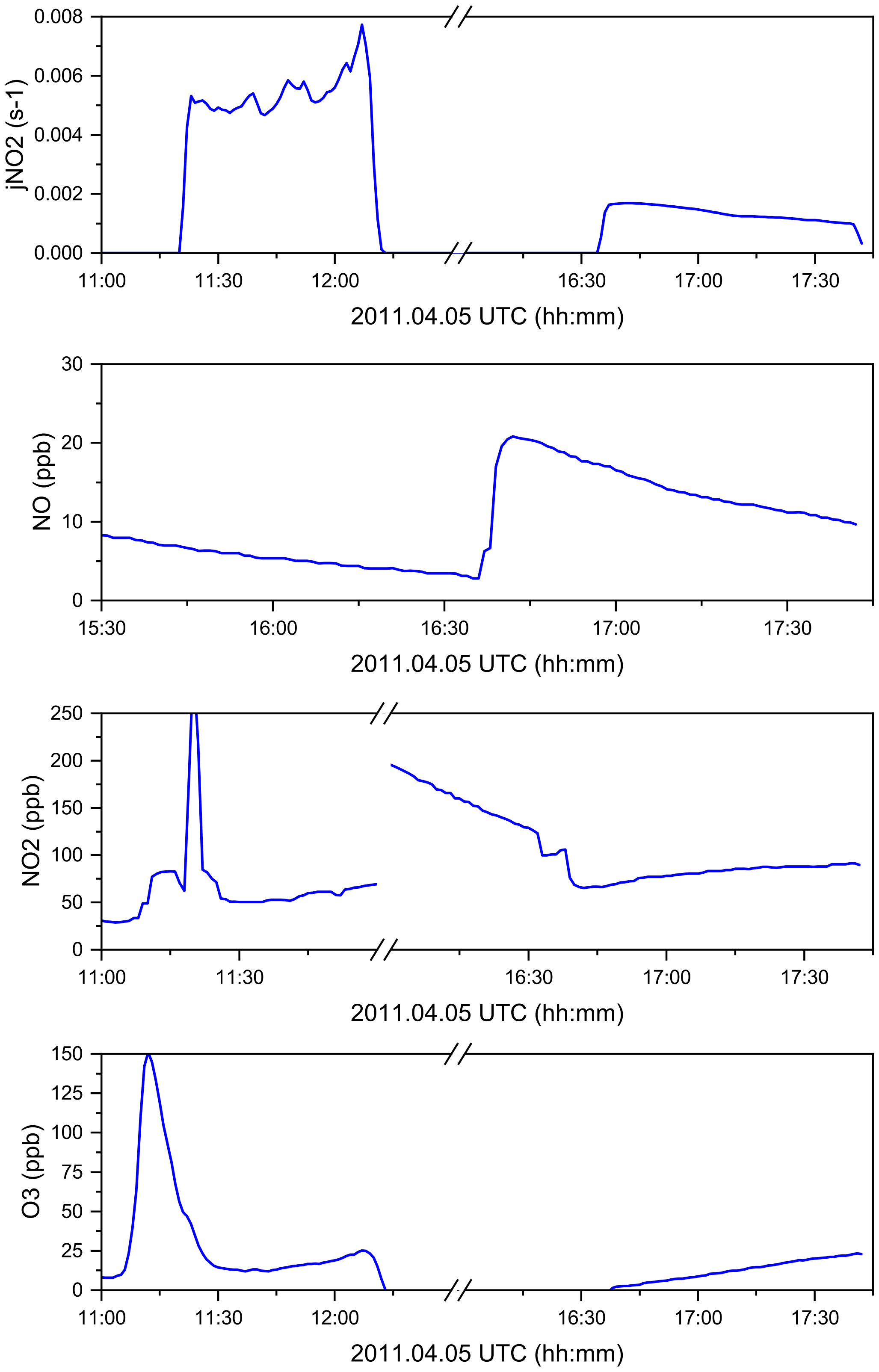

Figure S15. p, T, j_{NO2}, NO, NO_2 and O_3 during the PIP-NO photolysis experiment on2011.04.05

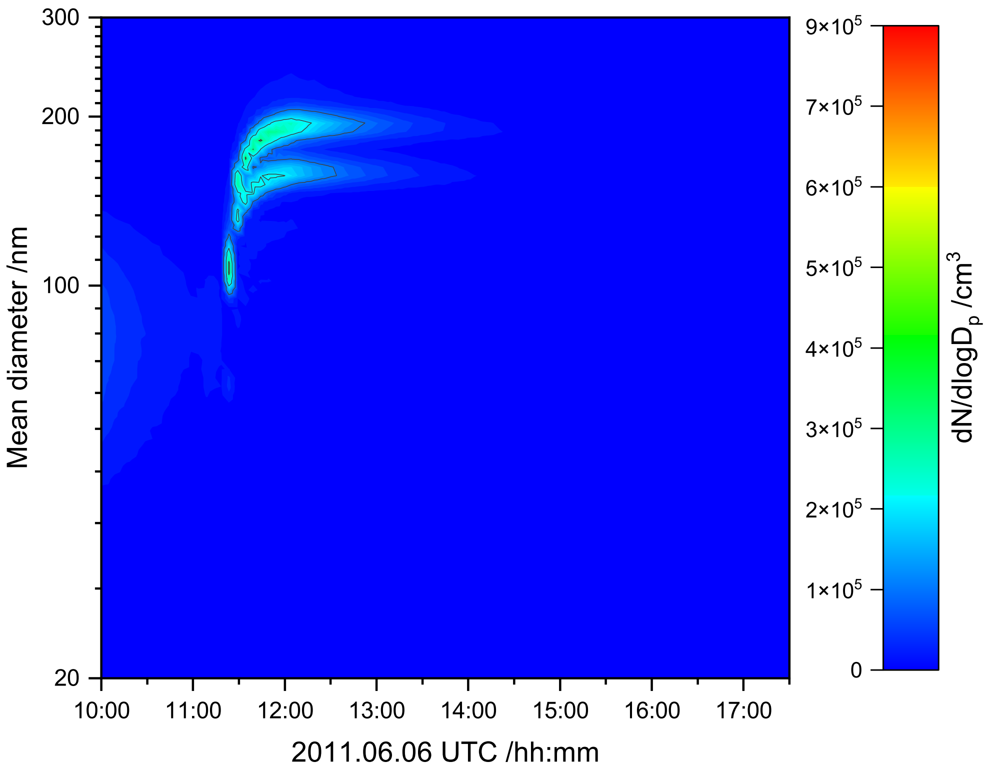

Figure S16. SMPS results 2011.06.06.
Particle number concentration and particle size distribution from SMPS measurements during the 2011.06.06 photolysis experiment in the EUPHORE atmospheric simulation chamber B.

Piperidine photo-oxidation studies

Table S19. Initial experimental conditions in piperidine photo-oxidation experiments.

Date	PIP [ppbv]	IPN (rate, total) [µl min^{-1}; µl]	NO [ppbv]	NO_2 [ppbv]	RH [%]	T [K]
2016.07.11	170	0	100	95	1.4	300
2016.07.12	160	0.1; 25.6	<1	<2.5	n.a.	297
2016.07.15	180	0.1; 20.1	145	<2.5	0.6	300
2016.07.18	180	0.1 (32 min), 0.2 (28 min), 0.4 (93 min)	<1	100	1.0	297

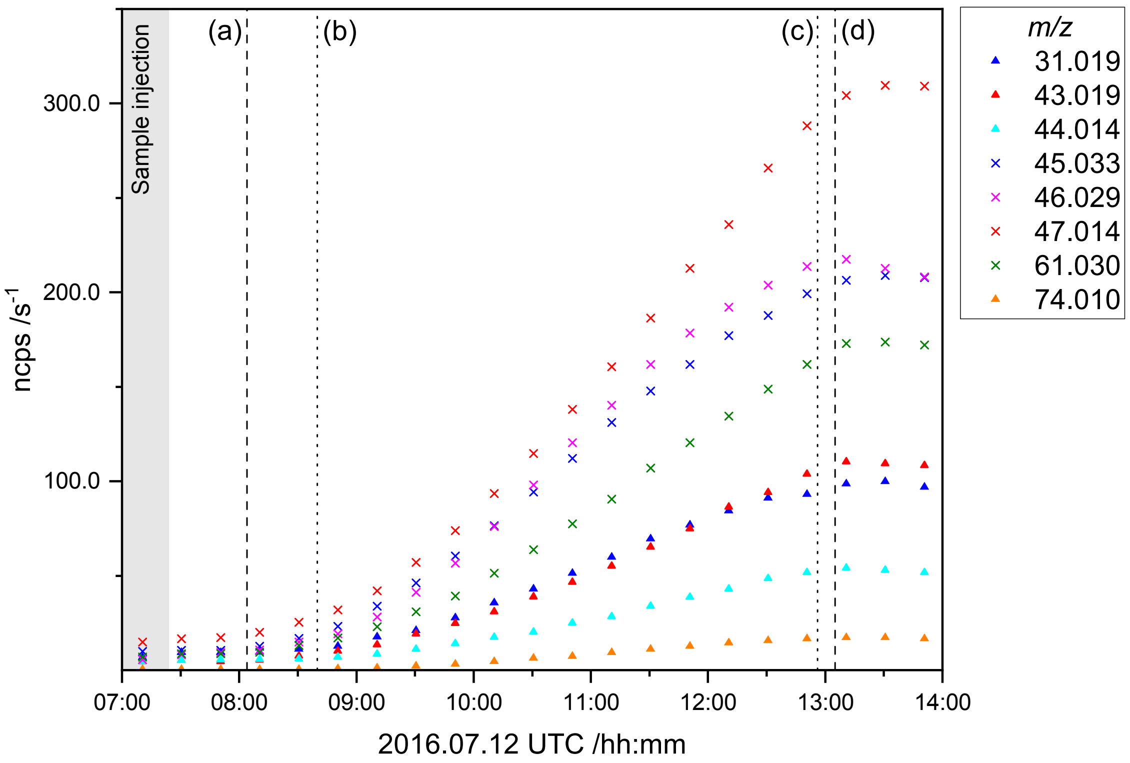

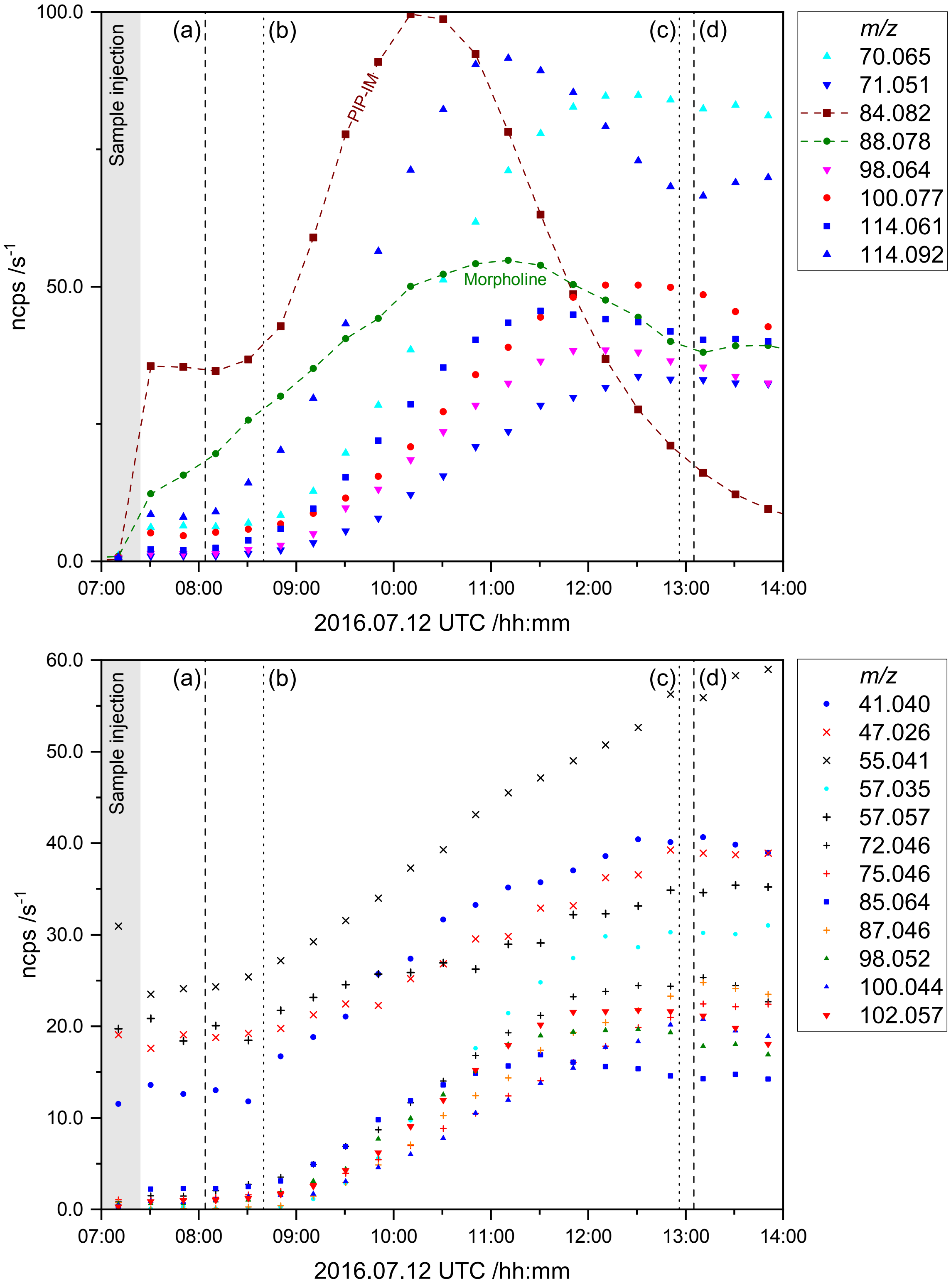

Figure S17. Small ion signals observed by PTR-TOF-MS during the experiment on 2016.07.12.
Top: Ion signals that continuously increase in total by > 2% of the decrease in m/z 86.097 (protonated PIP). Middle: Ion signals that first increase in total by > 2% of the decrease in m/z 86.097, and then decrease in intensity during the experiment. A scaled ion signal of protonated PIOP-IM is included for comparison of the time profiles. Bottom: Ion signals that increase between 1 and 2 % of the decrease in m/z 86.097.

Table S20. Ion signals observed by PTR-TOF-MS during the experiment on 2016.07.12.

Observed m/z	Ion sum formula	Exact m/z	Growth[a]	Interpretation & comments
31.019	CH_3O^+	31.018		CH_2O (chamber artefact)
41.040	$C_3H_5^+$	41.039	<2%	General fragment
43.019	$C_2H_3O^+$	43.018		Fragment from $[CH_3COOH]H^+$
44.014	CH_2NO^+	44.014		HNCO, product from $CHONH_2$+OH (chamber artefact)
45.033	$C_2H_5O^+$	45.034		CH_3CHO (chamber artefact)
45.992	NO_2^+	45.993		From HNO_3 and NO_2-containing organics
46.029	CH_4NO^+	46.029		$CHONH_2$, chamber artefact
47.004	HNO_2^+	47.001		NO_2
47.014	$CH_3O_2^+$	47.013		HCOOH (chamber artefact)
47.026	$H_3N_2O^+$	47.025	<2%	
55.041	$C_3H_5N^+$	55.042	<2%	
57.035	$C_3H_5O^+$	57.034	<2%	
57.057	$C_3H_7N^+$	57.058	<2%	
57.071	$C_4H_9^+$	57.070		General fragment
61.030	$C_2H_5O_2^+$	61.029		CH_3COOH (chamber artefact)
69.071	$C_5H_9^+$	69.070		General fragment, also observed in PIP-NO photolysis experiments
70.065	$C_4H_8N^+$	70.066		
71.051	$C_4H_7O^+$	71.050		Also observed in PIP-NO photolysis experiments
72.046	$C_3H_6NO^+$	72.045	<2%	
73.030	$C_3H_5O_2^+$	73.029		
74.010	$CH_2N_2O_2^+$	74.012	<2%	
74.025	$C_2H_4NO_2^+$	74.024		
75.046	$C_3H_7O_2^+$	75.045	<2%	
84.082	$C_5H_{10}N^+$	84.081		2,3,4,5-tetrahydro pyridine (PIP-IM)
85.064	$C_5H_9O^+$	85.065	<2%	
86.065	$C_4H_8NO^+$	86.061		Also observed in PIP-NO photolysis experiments<br>3,6-dihydro-2*H*-1,4-oxazine MOR-IM
86.097	$C_5H_{12}N^+$	86.097		Piperidine (PIP)
87.046	$C_4H_7O_2^+$	87.045	<2%	
88.078	$C_4H_{10}NO^+$	88.076		Morpholine (MOR, desorbing from chamber walls)
98.052	$C_4H_6N_2O$	98.048	<2%	
98.064	$C_5H_8NO^+$	98.061		
100.044	$C_4H_6NO_2^+$	100.040	<2%	
100.077	$C_5H_{10}NO^+$	100.076		4-Piperidin-one ($PIPC^4$=O) and $CHOCH_2CH_2CH_2N=CH_2$
102.057	$C_4H_8NO_2^+$	102.056	<2%	Products from MOR: $MORC^2$=O, $MORC^3$=O.
114.061	$C_5H_8NO_2^+$	114.056		
114.092	$C_6H_{12}NO^+$	114.092		Condensation product from PIP-IM + CH_2O
115.094	$C_5H_{11}N_2O^+$	115.087		1-Nitrosopiperidine (PIP-NO)
131.083	$C_5H_{11}N_2O_2^+$	131.082		1-Nitropiperidine (PIP-NO_2)

[a] Only ion signals increasing by more than 1% of the *m/z* 86.097 ion signal decrease during the time of photo-oxidation are included. Signals due to isotopes are not included.

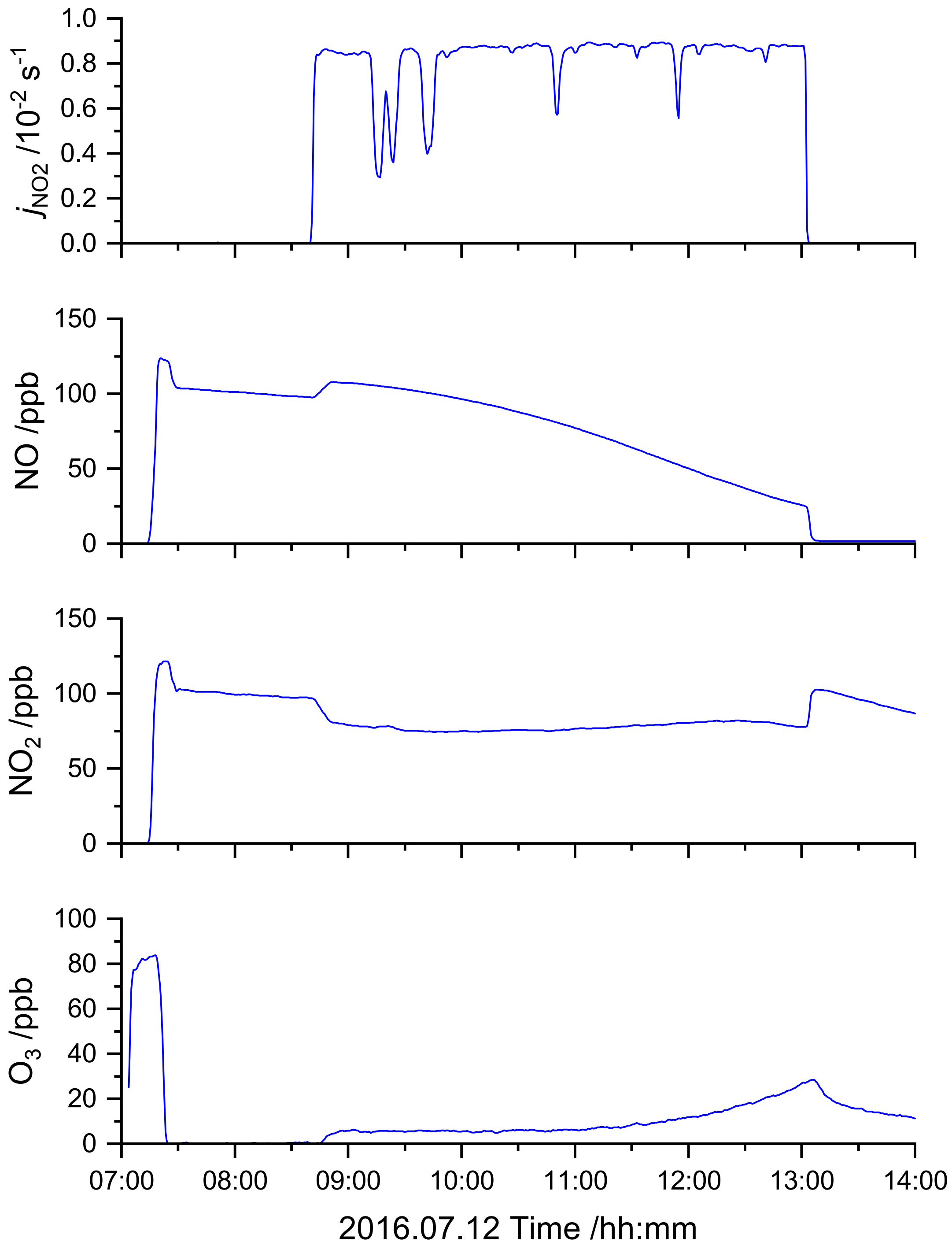

Figure S18. ***j*$_{NO2}$, NO, NO$_2$ and O$_3$ mixing ratios during the 2016.07.12 photo-oxidation experiment.**

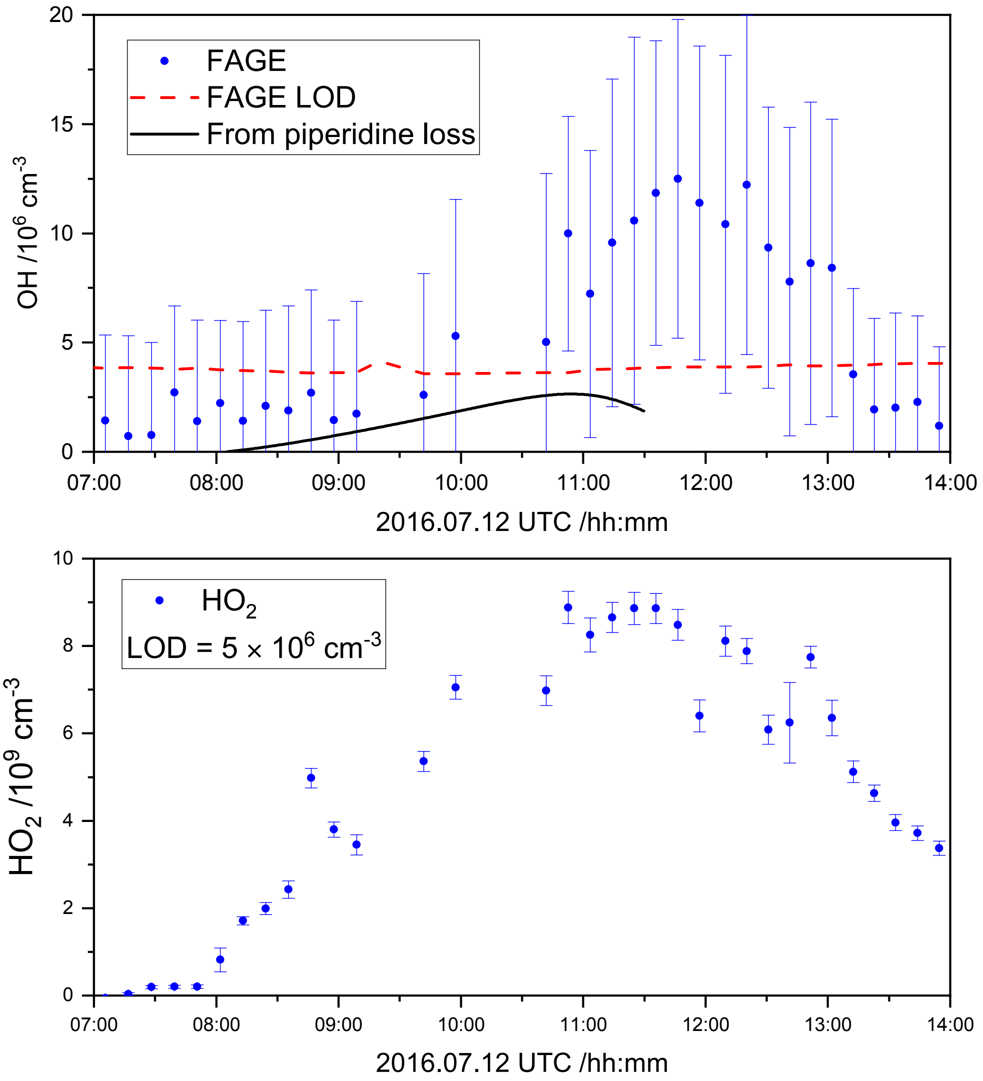

Figure S19. OH and HO_2 concentrations during the 2016.07.12 photo-oxidation experiment.
Top: Comparison of OH concentrations from FAGE (Fluorescence Assay by Gas Expansion) and from modeling the piperidine loss during the photo-oxidation. Bottom: HO_2 concentration from FAGE.
Notes: The OH concentration derived from the tracer decays is around 50% lower than for the measured OH. There are several possible explanations for this. Most likely, there is an unrecognised interference in the FAGE cell under the operational conditions in Valencia caused by an unknown species in the chamber that is contributing towards the OH signal. These are species that may generate OH in the detection cell either when photolyzed by the 308 nm laser light, or following their decomposition within the cell after they are sampled and drawn into the low-pressure cell.[101] No testing has been performed for the Leeds aircraft cell for any potential interferences from either gaseous or aerosol phase amine oxidation products, and the aircraft-based instrument is not equipped with an inlet pre-injector (IPI),[102] so it is not possible to rule out or quantify any such interference.

(101) Mao, J.; Ren, X.; Zhang, L.; Van Duin, D. M.; Cohen, R. C.; Park, J. H.; Goldstein, A. H.; Paulot, F.; Beaver, M. R.; Crounse, J. D., et al. Insights into hydroxyl measurements and atmospheric oxidation in a California forest. *Atmos. Chem. Phys.* **2012,** *12*, 8009-8020. DOI: 10.5194/acp-12-8009-2012

(102) Woodward-Massey, R.; Slater, E. J.; Alen, J.; Ingham, T.; Cryer, D. R.; Stimpson, L. M.; Ye, C.; Seakins, P. W.; Whalley, L. K.; Heard, D. E. Implementation of a chemical background method for atmospheric OH measurements by laser-induced fluorescence: characterisation and observations from the UK and China. *Atmos. Meas. Tech.* **2020,** *13*, 3119-3146. DOI: 10.5194/amt-13-3119-2020

Particle analysis during the piperidine + OH reaction

Table S21. Results from filter sampling

Average N-nitropiperidine and N-nitrosopiperidine concentrations (and the corresponding average percentage yields from the parent amine) detected in aerosol collected throughout the piperidine photo-oxidation experiments. The nitramine: nitrosamine mass ratios are also reported. All reported concentrations have been corrected for particle wall loss and chamber dilution.

Date	Sampling time (hh:mm, UTC)	[N-nitropiperidine] / μg m^{-3}	Average % nitramine yield	[N-nitrosopiperidine] / μg m^{-3}	Average % nitrosamine yield	Nitramine: nitrosamine
11-07-2016	06:22 – 06:52	0.000	n/a	0.000	n/a	n/a
	09:41 - 10:41*	1.125	0.433	0.467	0.180	2.41
	10:46 - 11:46*	1.491	0.361	0.135	0.033	11.0
	11:52 – 12:52*	2.152	0.416	0.166	0.032	12.9
	13:37 – 14:37	0.058	0.009	0.000	0.000	n/a
12-07-2016	06:20 – 06:50	0.095	n/a	0.000	n/a	n/a
	09:48 – 10:48*	0.380	0.089	0.050	0.012	7.69
	10:55 – 11:55*	0.775	0.146	0.015	0.003	52.0
	12:01 – 13:01*	1.018	0.183	0.011	0.002	91.2
	13:42 – 14:42	0.034	0.006	0.013	0.002	2.55
15-07-2016	11:04 – 13:06*	0.340	0.109	0.133	0.043	2.56
	14:01 – 15:01	0.075	0.012	0.045	0.007	1.68
18-07-2016	06:28 – 06:58	0.007	n/a	0.000	n/a	n/a
	09:44 – 10:44*	0.000	0.000	0.040	0.012	n/a
	10:50 – 11:50*	3.256	0.577	0.124	0.022	26.3
	12:41 – 13:41	0.050	0.008	0.073	0.011	0.68

* Denotes sampling intervals during which the chamber canopy was open.

Table S22. Results from filter sampling

Average NO_3^- and $C_5H_{12}N^+$ concentrations (and the corresponding average percentage yields from the parent amine) detected in aerosol collected throughout the piperidine photo-oxidation experiments. The NO_3^- : $C_5H_{12}N^+$ molar ratios are also reported. All reported concentrations have been corrected for particle wall loss and chamber dilution.

Date	Sampling time (hh:mm, UTC)	NO_3^- / µg m^{-3}	Average % NO_3^- yield	$C_5H_{12}N^+$ / µg m^{-3}	Average % $C_5H_{12}N^+$ yield	Molar NO_3^- : $C_5H_{12}N^+$
11-07-2016	06:22 – 06:52	0.571	n/a	0.000	n/a	n/a
	09:41 - 10:41*	2.868	1.104	42.658	16.415	0.09
	10:46 - 11:46*	6.063	1.469	37.735	9.142	0.22
	11:52 – 12:52*	12.884	2.491	37.715	6.131	0.56
	13:37 – 14:37	19.724	3.114	25.872	4.085	1.06
12-07-2016	06:20 – 06:50	0.090	n/a	0.000	n/a	n/a
	09:48 – 10:48*	2.420	0.569	24.531	5.770	0.14
	10:55 – 11:55*	6.939	1.309	9.653	1.822	1.00
	12:01 – 13:01*	9.159	1.650	7.893	1.422	1.61
	13:42 – 14:42	9.030	1.572	8.632	1.502	1.45
15-07-2016	11:04 – 13:06*	3.105	0.994	24.793	7.933	0.17
	14:01 – 15:01	7.704	1.205	9.234	1.444	1.16
18-07-2016	06:28 – 06:58	0.000	n/a	0.000	n/a	n/a
	09:44 – 10:44*	7.633	2.392	32.659	10.240	0.32
	10:50 – 11:50*	19.692	3.491	20.470	3.629	1.33
	12:41 – 13:41	20.272	3.050	16.643	2.504	1.69

* Denotes sampling intervals during which the chamber canopy was open.

N-H/C-H branching in the piperidine + OH reaction

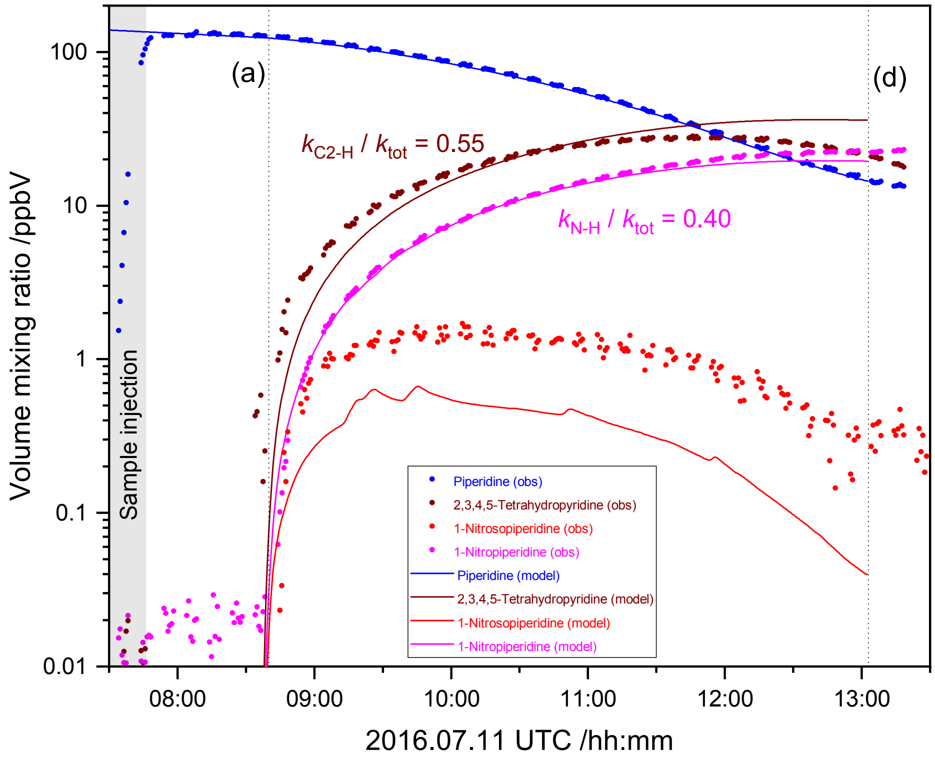

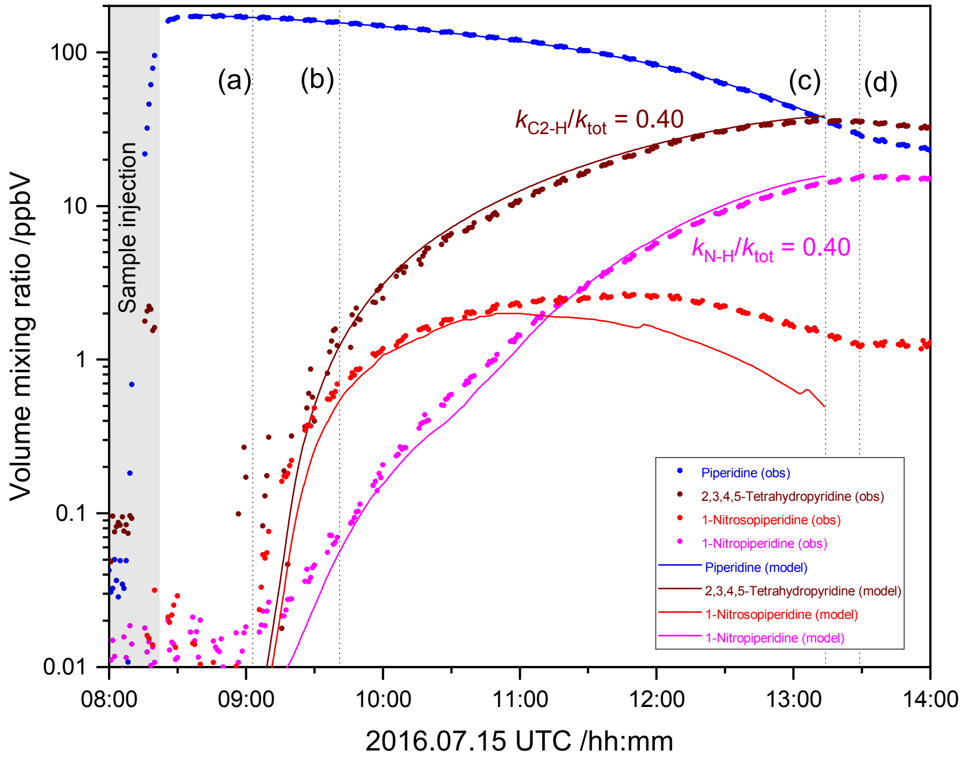

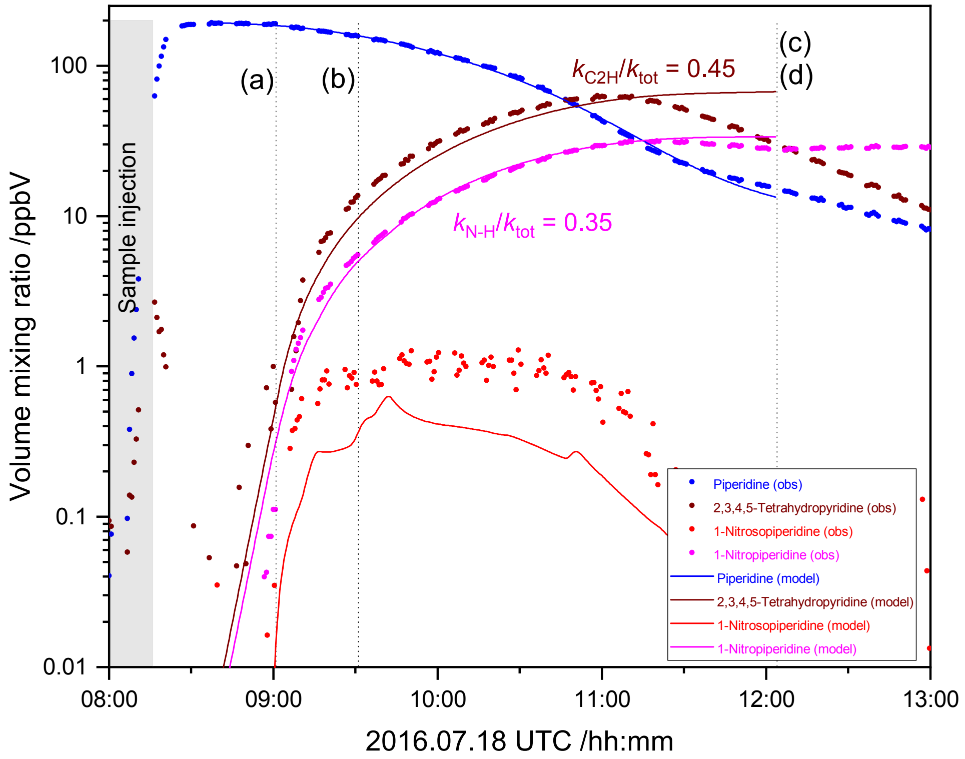

Figure S20. Observed and modelled PIP, PIP-IM, PIP-NO and PIP-NO_2 during photo-oxidation experiments. (a): Chamber canopy opened to solar radiation. (b): IPN injection started. (c) IPN injection stopped. (d): Chamber canopy closed.

Synthesis of experimental and theoretical results

DISCUSSION & CONCLUSIONS

Table S23. Parameters for box-modelling of nitrosamine and nitramine formation
Rate coefficients (/10^{-11} cm^3 $molecule^{-1}$ s^{-1}) for amine, nitrosamine and nitramine reactions with OH, and relative rate coefficients relevant to the branching in aminyl radical reactions with O_2, NO and NO_2.

	k_{OH} (k_1)	k_{1a}/k_1	k_2/k_{4a} / 10^{-8}	k_{4b}/k_{4a}	k_3/k_{4a}	$j_{rel} = j_{nitrosamine} / j_{NO2}$
Me_2NH	6.8 [93]	0.41 [110]	39.0 [89]	0.22 [89]	0.26 [89]	0.34
Me_2NNO	0.30 [95]					
Me_2NNO_2	0.35 [95, 96]					
PIP [a]	12	0.38	7.66	0	0.53	0.34
PIP-NO [b]	1.2 [b]					
PIP-NO_2	1.2 [b]					

[a] This work; [b] assumed value.

(89) Lindley, C. R. C.; Calvert, J. G.; Shaw, J. H. Rate Studies of the Reactions of the $(CH_3)_2N$ Radical with O_2, NO, and NO_2. *Chem. Phys. Lett.* **1979,** *67*, 57-62. DOI: 10.1016/0009-2614(79)87105-5

(93) M. R. McGillen, W. P. L. Carter, A. Mellouki, J. J. Orlando, B. Picquet-Varrault and T. J. Wallington, Database for the Kinetics of the Gas-Phase Atmospheric Reactions of Organic Compounds. *Earth Syst. Sci. Data*, 2020, **12**, 1203-1216. DOI: 10.5194/essd-12-1203-2020

(95) E. C. Tuazon, W. P. L. Carter, R. Atkinson, A. M. Winer and J. N. Pitts, Atmospheric Reactions of N-Nitrosodimethylamine and Dimethylnitramine. *Environmental Science & Technology*, 1984, **18**, 49-54. DOI: 10.1021/es00119a011

(96) S. S. Zabarnick, J. W. Fleming, A. P. Baronavski and M. C. Lin, Reaction kinetics of hydroxyl with nitromethane, dimethylnitrosamine, and 1,3,5-trioxane; photolytic production of hydroxyl from nitromethane at 266 nm *NBS Special Publication (United States)*, 1986, **716**, 731-756

(110) Onel, L.; Blitz, M.; Dryden, M.; Thonger, L.; Seakins, P. Branching Ratios in Reactions of OH Radicals with Methylamine, Dimethylamine, and Ethylamine. *Environ. Sci. Technol.* **2014,** *48*, 9935-9942. DOI: 10.1021/es502398r

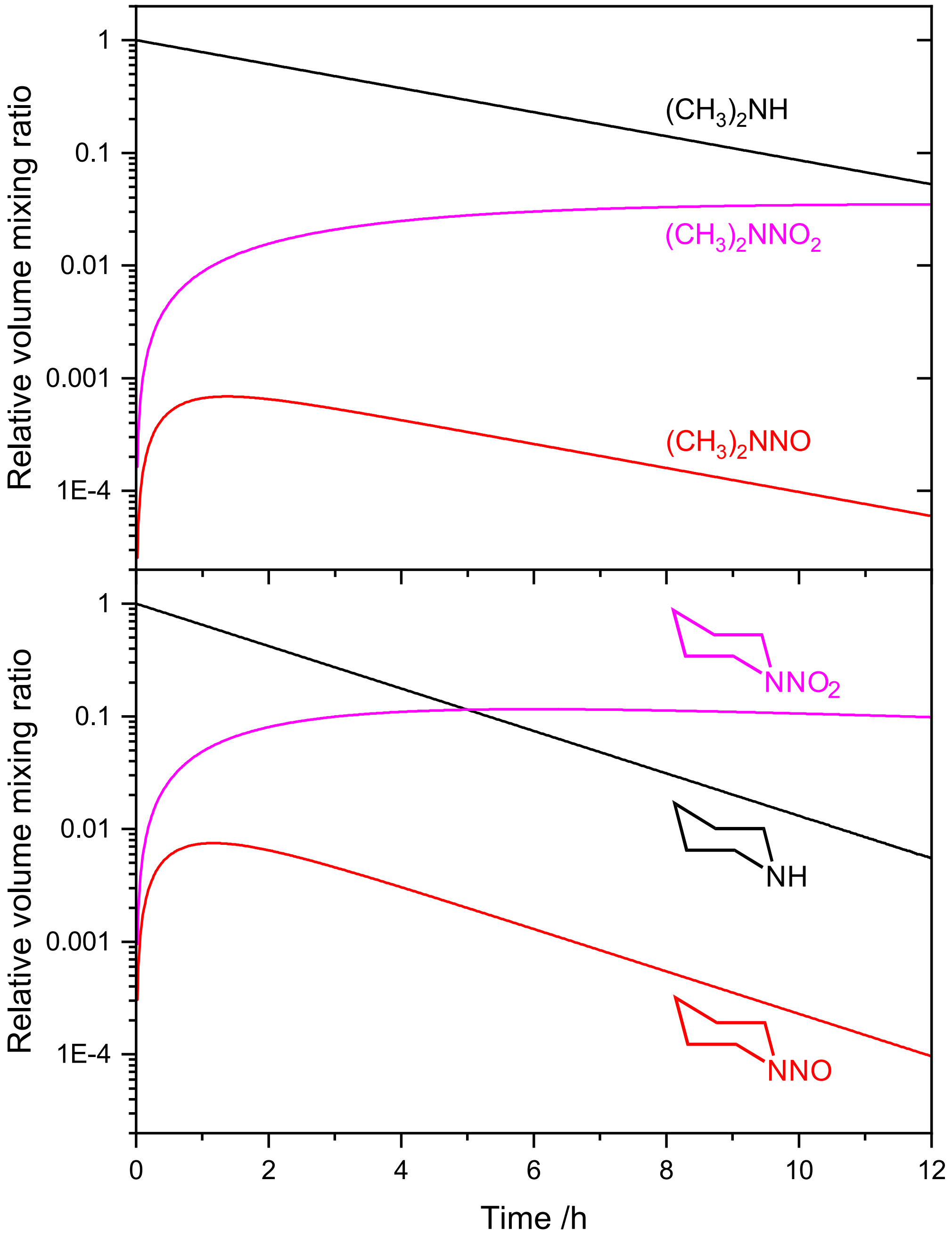

Figure S21. Box model results for nitrosamine and nitramine formation from $(CH_3)_2NH$ and PIP emissions
